# Evaluation of the MGISEQ-2000 Sequencing Platform for Illumina Target Capture Sequencing Libraries

**DOI:** 10.3389/fgene.2021.730519

**Published:** 2021-10-27

**Authors:** Jidong Lang, Rongrong Zhu, Xue Sun, Siyu Zhu, Tianbao Li, Xiaoli Shi, Yanqi Sun, Zhou Yang, Weiwei Wang, Pingping Bing, Binsheng He, Geng Tian

**Affiliations:** ^1^ Bioinformatics and R and D Department, Geneis (Beijing) Co. Ltd., Beijing, China; ^2^ Qingdao Geneis Institute of Big Data Mining and Precision Medicine, Qingdao, China; ^3^ Academician Workstation, Changsha Medical University, Changsha, China; ^4^ Vascular Surgery Department, Tsinghua University Affiliated Beijing Tsinghua Changgung Hospital, Beijing, China; ^5^ Department of Medicine, School of Medicine, University of California at San Diego, La Jolla, CA, United States

**Keywords:** illumina sequencing platform, MGISEQ-2000 sequencing platform, next generation sequencing, DNA nanoball, target capture library

## Abstract

Illumina is the leading sequencing platform in the next-generation sequencing (NGS) market globally. In recent years, MGI Tech has presented a series of new sequencers, including DNBSEQ-T7, MGISEQ-2000 and MGISEQ-200. As a complex application of NGS, cancer-detecting panels pose increasing demands for the high accuracy and sensitivity of sequencing and data analysis. In this study, we used the same capture DNA libraries constructed based on the Illumina protocol to evaluate the performance of the Illumina Nextseq500 and MGISEQ-2000 sequencing platforms. We found that the two platforms had high consistency in the results of hotspot mutation analysis; more importantly, we found that there was a significant loss of fragments in the 101–133 bp size range on the MGISEQ-2000 sequencing platform for Illumina libraries, but not for the capture DNA libraries prepared based on the MGISEQ protocol. This phenomenon may indicate fragment selection or low fragment ligation efficiency during the DNA circularization step, which is a unique step of the MGISEQ-2000 sequence platform. In conclusion, these different sequencing libraries and corresponding sequencing platforms are compatible with each other, but protocol and platform selection need to be carefully evaluated in combination with research purpose.

## Introduction

With the launch of the Human Genome Project, next-generation sequencing (NGS) technology has had a huge impact on the biological field in the past 20 years (Consortium, 2015; Yang et al., 2015; Goodwin et al., 2016). Different companies and research institutions have developed various sequencing approaches and platforms, such as Roche’s 454 sequencing platform, Illumina’s sequencing by synthesis (SBS) technology, and PacBio’s single-molecule nanopore sequencing technology (Rivas et al., 2015; Goodwin et al., 2016). Among them, the sequencers or sequencing platforms developed by the Illumina Company have a dominant position in the sequencing market due to their high throughput and high sequencing accuracy. Over time, the development of machine hardware and the diversification of bioinformatics analysis software tools have led to drastic reductions in sequencing costs and increases in convenience and usability, even for new developed techniques like single cell sequencing (Yang et al., 2020a; Xu et al., 2020). For example, NGS technology plays a vital role in analyzing somatic mutations that occur in multiple tumor types. The Cancer Genome Atlas (TCGA) (Weinstein et al., 2013) and International Cancer Genome Consortium (ICGC) (Hudson et al., 2010) have sequenced thousands of tumors from more than 50 cancer types and summarized the significant genetic somatic mutations that occur during the process of tumorigenesis (Alexandrov et al., 2013). These data have played an extremely important role in promoting cancer genome research and development (He et al., 2020a; He et al., 2020b; Liu et al., 2021).

Recently, MGI Tech Co., Ltd (referred to MGI) launched a series of NGS sequencers and platforms based on DNA nanoball (DNB) and probe-anchor synthesis (cPAS) technology, such as MGISEQ-200, MGISEQ-2000, and DNBSEQ-T7 (Fehlmann et al., 2016). They have gradually achieved a certain sales volume and have become another option for high-throughput sequencing. For example, MGISEQ-2000 can generate approximately 1.44 TB sequencing data per run with a running cost of only 10 USD/GB. Several studies have compared the performance between MGI and the Illumina sequencing platform, and the results showed that they were highly consistent for different types of sequencing libraries, including whole-exome sequencing (WES) (Xu et al., 2019), whole-genome sequencing (WGS) (Patch et al., 2018), transcriptome sequencing (Zhu et al., 2018; Jeon et al., 2019; Patterson et al., 2019; Zeng et al., 2020), single-cell transcriptome sequencing (Natarajan et al., 2019; Peng et al., 2020a; Senabouth et al., 2020; Zhuang et al., 2021), metagenome sequencing (Fang et al., 2018) and small RNA sequencing (Huang et al., 2017) libraries.

When MGI launched their sequencers, they indicated that they were compatible with the sequencing libraries constructed based on Illumina protocols, that is, that the MGISEQ platform could sequence the Illumina libraries. In our study, we used the same capture DNA libraries constructed based on the Illumina protocol for sequencing with the Illumina NextSeq 500 and MGISEQ-2000 sequencing platforms. We found that the two platforms had high consistency in the hotspot mutation analysis and that there was a significant loss of the 101–133 bp fragments on the MGISEQ-2000 sequencing platform but not in the capture DNA libraries based on the MGISEQ protocol. We hypothesized that this might be related to fragment selection or low ligation efficiency during the DNA circularization step, a step that is unique to the MGISEQ-2000 sequence platform. Hence, although the selection of sequencers and platforms is becoming increasingly diversified and all theoretically compatible and applicable to each other, the choice of platform for practical applications may need to be further evaluated according to the research purpose and library characteristics.

## Materials and Methods

### Sample Collection and Experimental Groups

Our research was approved by the Qingdao Geneis Institute of Big Data Mining and Precision Medicine in November 2019, and the research ID was Ethics-QD-[2020] No. 001. A total of 272 samples (patient age: 29–91 years old) were collected at Qingdao Geneis Institute of Big Data Mining and Precision Medicine from December 2019 to March 2020, including 79 plasma samples, 21 white blood cell samples and 172 formalin-fixed and paraffin-embedded (FFPE) samples. Informed written consent forms were obtained from patients, and identifying information was removed. The clinical information of the samples is shown in Table 1.

**TABLE 1:** Clinical information for collected samples.

Clinical characteristics		All samples (*n* = 272)
Unknown		46
Age, Median (Range)-yrs		62.5 (29.0–91.0)
Age groups-No.%	15–49 years	24/226 (10.62)
	50–64 years	97/226 (42.92)
	≥65 years	105/226 (46.46)
Sex-No.%	Female	103/226 (45.58)
	Male	123/226 (54.42)
Disease-No.%	Lung cancer	166/226 (73.45)
	Colon cancer	13/226 (5.75)
	Rectal cancer	11/226 (4.87)
	Gastric cancer	6/226 (2.65)
	Breast cancer	5/226 (2.21)
	Esophageal cancer	5/226 (2.21)
	Colorectal cancer	4/226 (1.77)
	Nasopharyngeal carcinoma	2/226 (0.88)
	Liver cancer	1/226 (0.44)
	Ovarian cancer	1/226 (0.44)
	Tongue cancer	1/226 (0.44)
	Unknown	11/226 (4.87)

We randomly selected 204 (75%: 204/272) samples to construct capture libraries based on the Illumina protocol and performed data analysis. The remaining samples were divided into two groups of 34 samples (12.5%: 34/272) using different capture panels and constructing capture libraries based on the MGISEQ protocol for sequencing and data analysis, respectively.

### Library Preparation Based on Illumina Platform and Sequencing

DNA for NGS-based analysis was extracted using the GeneRead Kit (Qiagen, Hilden, Germany) for FFPE tissue and the QIAamp DNA Blood Mini Kit (Qiagen, Hilden, Germany) for white blood cell samples. DNA (200 ng) was used to build the library by using the NEBNext Ultra II DNA library Prep Kit for Illumina (96 reactions) (NEB, Ipswich, MA, United States). Cell-free DNA was extracted using a QIAamp Circulating Nucleic Acid Kit (Qiagen, Hilden, Germany) according to the manufacturer’s instructions. The extracted DNA (20 ng/sample) was then used to build libraries using Accel-NGS^®^ 2S Plus DNA Library Kits (96 reactions; Swift BioSciences, Ann Arbor, MI, United States). Integrated DNA Technologies (IDT, Skokie, IL, United States) or Agilent Technologies (Santa Clara, CA, United States) custom probes were used for hybridization capture. We used the IDT 38-hotspot gene panel or Agilent 519 gene panel (Supplementary Table S5) for all 272 libraries. Quantification was performed with an Illumina/Universal Library Quantification Kit (Kapa Biosystems, Wilmington, MA, United States) on an ABI 7500 Real Time Polymerase Chain Reaction (PCR) System (Applied Biosystems, Waltham, MA, United States). The quality control for Agilent 2,100 Bioanalyzer used a High-Sensitivity DNA Kit (Agilent Technologies, Santa Clara, CA, United States). Next-generation sequencing-based analysis was performed on a NextSeq500 or MiSeqDX instrument according to the manufacturer’s instructions (Illumina, San Diego, CA, United States). With the NextSeq500/550 High Output V2 Kit or MiSeqTMDX Reagent V3 Kit, Illumina NextSeq500 or MiSeqDX (Illumina, San Diego, CA, United States) was used for DNA sequencing in 302 cycles for 151 bp paired-end sequencing. All 272 libraries were also analyzed on a MGISEQ2000 instrument according to the manufacturer’s instructions (BGI, Shenzhen, Guangdong, China). With the MGISEQ-2000RS High Output kit (BGI, Shenzhen, Guangdong, China), MGISEQ-2000 (BGI, Shenzhen, Guangdong, China) was used for DNA sequencing in 200 cycles and 300 cycles for 100 bp and 150 bp paired-end sequencing, respectively.

### Library Preparation Based on the MGISEQ Platform and Sequencing

DNA libraries were prepared with the MGIEasy FS DNA Library Prep Set (BGI, Shenzhen, Guangdong, China). DNA (50–200 ng) was fragmented physically with a Covaris S220 instrument (Covaris, Woburn, MA, United States), followed by A-tailing, adapter ligation and PCR amplification. DNA library quality was assessed using a Qubit and Agilent 2,100 Bioanalyzer with a High Sensitivity DNA Kit. Cot-1 DNA blocking reagent (Thermo Fisher Scientific, Waltham, MA, United States), IDT universal blocking oligonucleotides and IDT adapter-specific blocking oligonucleotides were added to the pooled libraries and dried in a SpeedVac. The dried mixture was redissolved in mixed liquids of IDT hybridization buffer, IDT hybridization enhancer and BOKE capture probes (BOKE bioscience, Bejing, China). After hybridization at 65°C for 4 h, the target regions were captured with M270 streptavidin beads by incubation at 65°C for 45 min and then washed 3 times at 65°C and another 3 times at room temperature with IDT xGen lockdown reagents. Then, 15 postcapture amplification cycles were performed to obtain the captured libraries. Final libraries were pooled and sequenced using the MGISEQ-2000 sequencing platform with a 150 bp paired-end cycle kit.

### Data Normalization and Statistics

As the volume of sequencing data and read length of the Illumina and MGISEQ-2000 platforms were different (Supplementary Table S1), we “normalized” all 272 sample sequencing datasets, that is, each sample had the same read length and read number. We used seqtk (version: 1.0-r73-dirty) (https://github.com/lh3/seqtk) to “normalize” the raw sequencing data. We used a in-house perl program to caculate the number of reads, Q20 ratio and GC content (Supplementary Table S2).

### Data Preprocessing and Analysis

The normalized data were cleaned by Trimmomatic (version: 0.39) (Bolger et al., 2014), which filtered out the adapter contamination reads and low-quality reads and the parameter’s setting was ILLUMINACLIP:adapter sequence:2:30:10 LEADING:3 TRAILING:3 SLIDINGWINDOW:4:15 MINLEN:36 (adapter sequences for Illumina Nextseq 500 and MGISEQ-2000 were AGA​TCG​GAA​GAG​CAC​ACG​TCT​GAA​CTC​CAG​TCA​C/AGA​TCG​GAA​GAG​CGT​CGT​GTA​GGG​AAA​GAG​TGT​A and AAG​TCG​GAG​GCC​AAG​CGG​TCT​TAG​GAA​GAC​AA/AAG​TCG​GAT​CGT​AGC​CAT​GTC​GTT​CTG​TGA​GCC​AAG​GAG​TTG, respectively). BWA-ALN algorithm (version: 0.7.12) (Li and Durbin, 2009) was applied for alignment with the reference genome hg19 (parameters: -o 1 -e 50 -t 4 -i 15 -q 10). The output SAM file was sorted and deduplicated with Samtools (version: 0.1.19) (Li et al., 2009), and the BAM format file was obtained. We used FreeBayes (version: 1.0.2) (Garrison and Marth, 2012) to detect SNP/InDel mutations (parameters: -j -m 10 -q 20 -F 0.001 -C 1). The mutations were annotated from the ANNOVAR database (Wang et al., 2010). Fragment size distribution was summarized from the paired-end alignment information (column ninth) in the BAM format file. Statistical analysis used the statistical functions in Microsoft Excel 2019 and R software (version 3.2.5).

## Results

### Data Quality Control Parameters Were Significantly Different Between the Illumina and MGISEQ-2000 Sequencing Platforms

**FIGURE 1:**
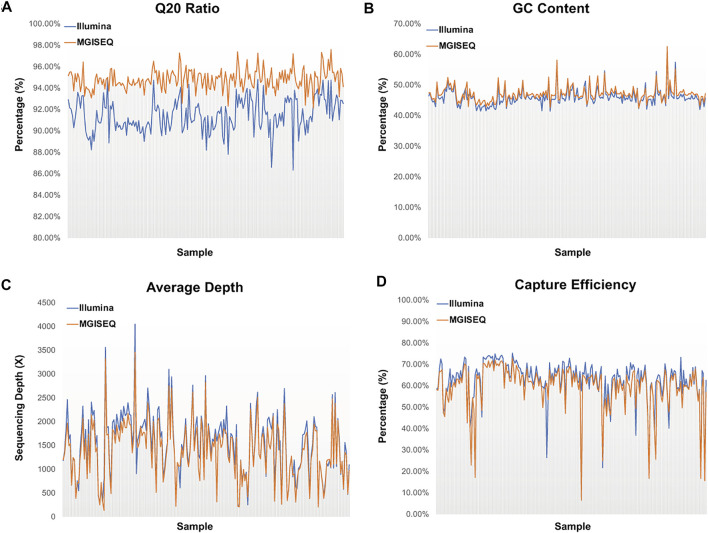
Comparison of Sequencing Data Quality Control Parameters between Illumina and MGISEQ-2000 Platforms. **(A)** Distribution of Q20 ratio by each sample. **(B)** Distribution of GC content by each sample. **(C)** Distribution of average depth by each sample. **(D)** Distribution of probe capture efficiency by each sample.

We compared the Q20 rate, GC content, mean depth and capture efficiency of 204 samples generated based on the Illumina library protocol, which were captured by the IDT 38-hotspot gene panel and sequenced on the Illumina and MGISEQ-2000 sequencing platforms (Figure 1, details in Supplementary Table S3), respectively. We found that all of the quality control parameters had significant differences, with *p*-values of 4.87e-85, 1.15e-4, 0.0326 and 0.0035, respectively, in the two-tailed heteroscedasticity *t*-test analysis. We thought that these differences could be due to the sequencing principles, the algorithm used for base recognition or the sequencing platform characteristics. For example, the Nextseq500 platform treated all unrecognized bases as G, while HiSeq-2000, MGISEQ-2000 and other previous four-color imaging sequencers treated these bases as N. Therefore, the GC content tended to be higher in the Illumina NextSeq500 results than in the others.

### Hotspot Mutations Showed High Consistency Between the Illumina and MGISEQ-2000 Sequencing Platforms.

**FIGURE 2:**
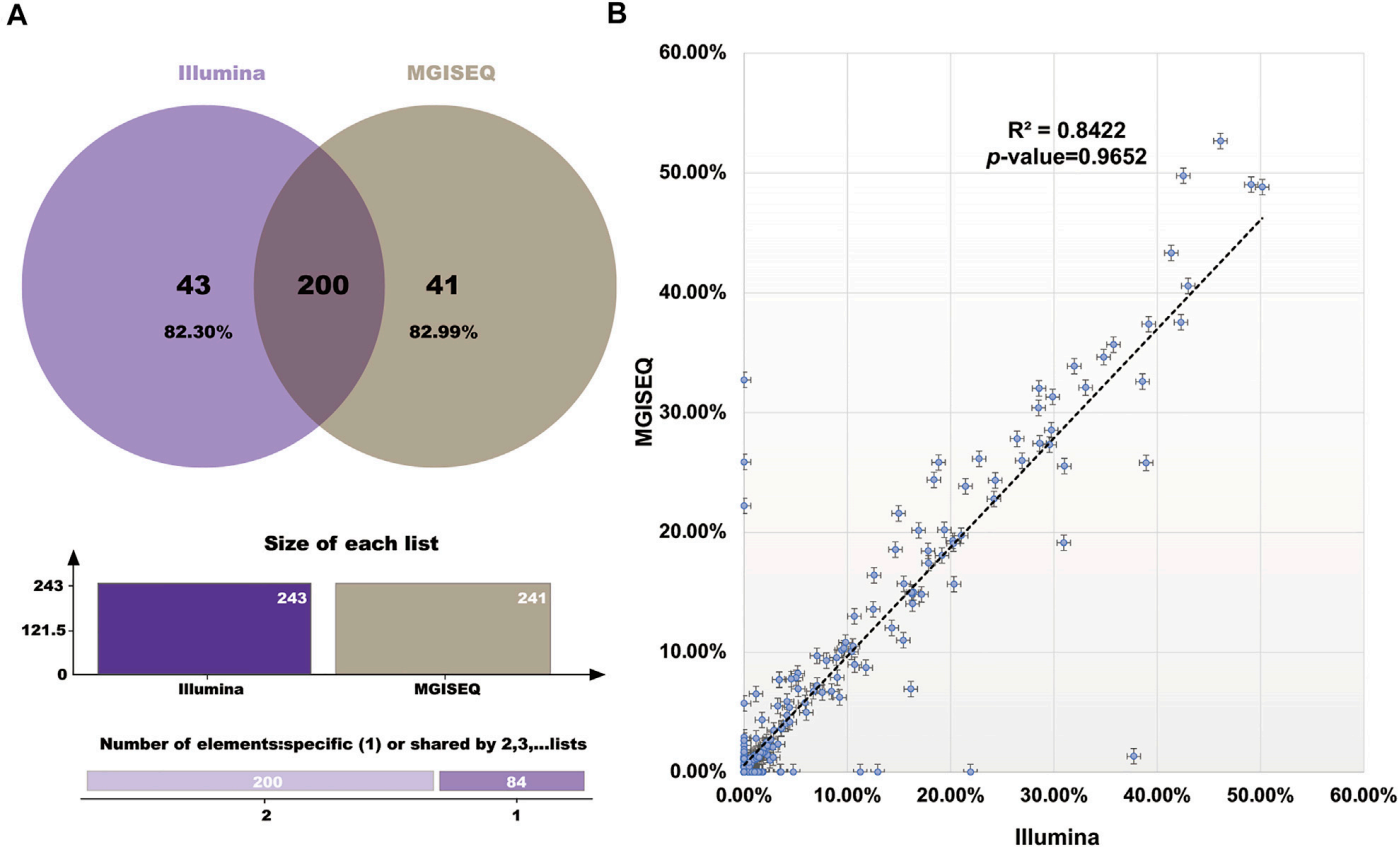
Hotspot Mutation Results Comparison between the Illumina and MGISEQ-2000 Platforms. **(A)** The Venn digram of the detected hotspot mutations comparision. **(B)** The correlation comparison of the detected hotspot mutation frequency values.

The hotspot mutations (SNPs and InDels) detected in 204 sample datasets were compared between the Illumina and MGISEQ-2000 platforms (Supplementary Table S4). We defined a positive detection filter condition as mutation frequency ≥ 0.4% for plasma samples and mutation frequency ≥ 1% for FFPE samples. We found that the hotspot mutation detection results had high consistency rates of 82.30% (Illumina: 200/243) and 82.99% (MGISEQ-2000: 200/241) (Figure 2A). Furthermore, no significant difference (*R* ^2^ = 0.8422, *p*-value = 0.9652) in mutation frequency was observed between the Illumina and MGISEQ-2000 platform data. (Figure 2B).

MGISEQ-2000 sequencing platform data based on Illumina libraries showed a significant loss of the 101–133 bp fragment.

**FIGURE 3:**
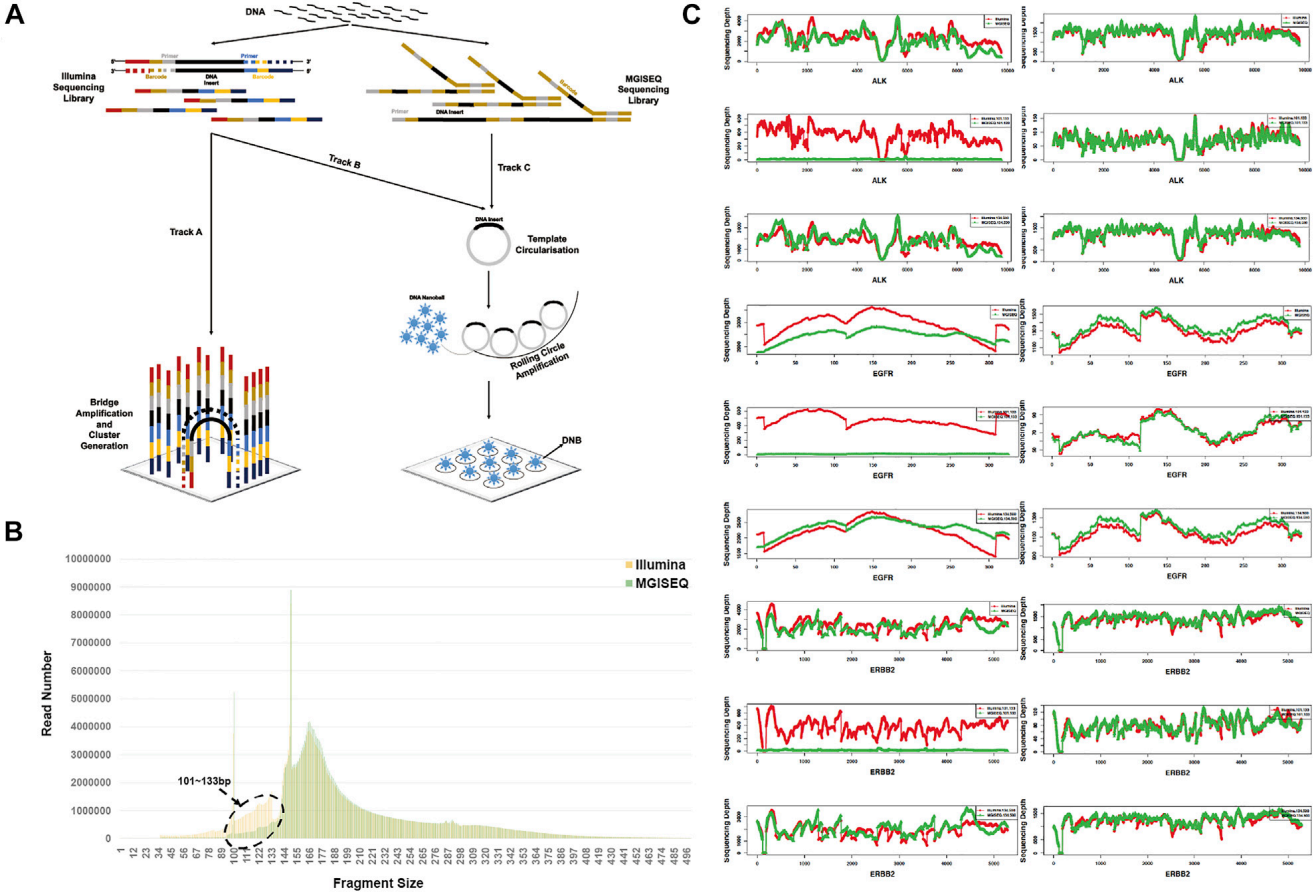
The Insert Fragment Size Analysis of Illumina-based Capture Library On the Illumina and MGISEQ-2000 Platforms. **(A)** The principles of Illumina and MGISEQ sequencers. Track A represents the Illumina library combined with bridge PCR amplification of the Illumina platform. Track B represents the Illumina library combined with DNA circularization for DNB of the MGISEQ-2000 platform. Track C represents the MGISEQ library combined with DNA circularization for DNB of the MGISEQ-2000 platform. **(B)** Compared with the Illumina platform data, the MGISEQ-2000 platform data had a significant loss of 101–133 bp fragments. **(C)** Statistical analysis of sequencing depth distribution in ALK, EGFR and ERBB2 with one sample. Sequencing depth distribution of 101–133 bp (left panel) and 134–500 bp (right panel) in ALK (top), EGFR (middle), and ERRBB2 (bottom). In each figure panel, the top panel shows the sample total sequencing depth distribution, the middle panel shows the sequencing depth of 101–133 bp fragment size, and the bottom panel shows the 134–500 bp fragment size sequencing depth distribution.

Insert fragment size and distribution were evaluated and analyzed for all 204 samples. As we used the same sample library for sequencing, the theoretical difference only existed in Illumina’s bridge PCR amplification and MGISEQ-2000s DNB circularization. (Figure 3A) (Goodwin et al., 2016; Chen et al., 2019; Korostin et al., 2020). Combining all 204 sample data for fragment size analysis, our results revealed a significant loss of 101–133 bp fragments in the MGISEQ-2000 platform data, with a *t*-test *p*-value of 3.3072e-17 (Figure 3B), while other fragment sizes, such as 134–500 bp (*t*-test *p*-value = 0.7264), did not show a difference. Although significant differences were found in the Q20 rate, GC content and other quality control statistics, these should be attributable to the sequencer system characteristics and should not have a great impact on the fragment size distribution. Therefore, the loss of the 101–133 bp fragment size may be related to the DNA cyclization step, that is, there may be fragment size selection in the circularization step or enrichment bias for longer DNA molecules and low ligation efficiency for shorter DNA molecules.

Then, we extracted 101–133 bp and 134–500 bp fragment size information from BAM files for each sample and analyzed the sequencing depth distribution of three common cancer genes, ALK receptor tyrosine kinase (*ALK*), epidermal growth factor receptor (*EGFR*) and erb-b2 receptor tyrosine kinase 2 (*ERBB2*). The results showed that 69.12% (141/204) of samples had 101–133 bp fragment size loss, while the sequencing depth distribution of 134–500 bp fragments was consistent with the overall total sequencing depth, indicating that the phenomenon was not due to stochasticity in specific genes (Figure 3C). The sequencing depth distribution of all samples was in the Supplementary Figures by each sample.

**FIGURE 4:**
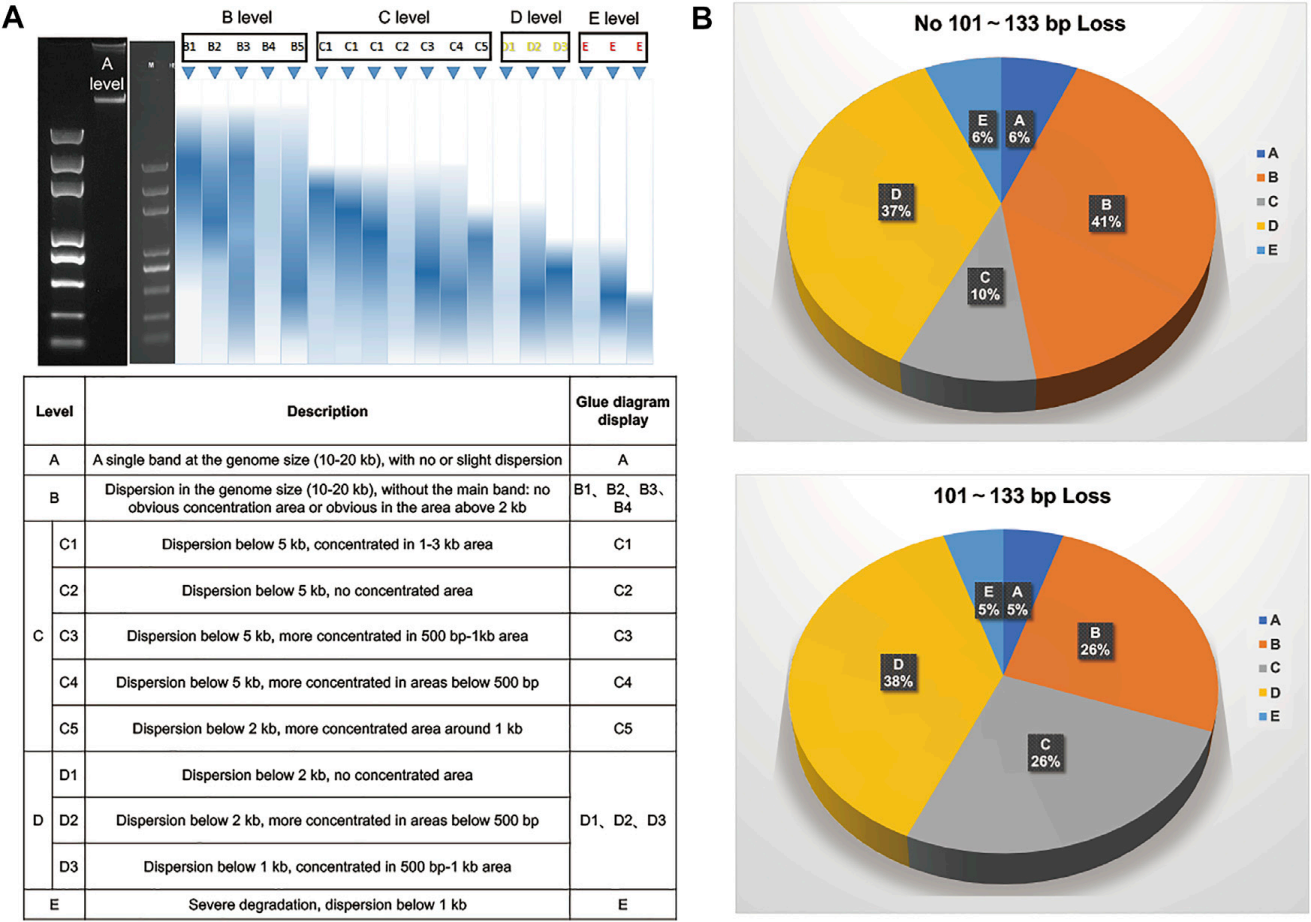
Statistical Analysis On The Quality of 204 Samples. **(A)** Sample quality grading table of gDNA agarose gel electrophoresis. **(B)** The distribution of different sample quality levels in samples with and without loss of 101–133 bp fragment size. The top figure represented sample quality grade distribution of samples without 101–133 bp fragment size loss. The bottom figure represented sample quality grade distribution of samples with 101–133 bp fragment size loss.

As we know, the use of FFPE or hemolyzed samples may have a great influence on the distribution of DNA fragment size. Therefore, we performed statistical analysis on the quality of 204 samples with and without 101–133 bp loss. First, we defined the sample quality levels with DNA agarose gel electrophoresis as A, B, C, D or E (Figure 4A). Then, all samples in each grade were subgrouped according to whether the 101–133 bp fragment size was lost. We found that the sample proportions of A, D and E levels were consistent in the two groups, while B and C levels were quite different. The proportions of B [C] level samples in the 101–133 bp loss group and 101–133 bp nonloss group were 25.53% (36/141) [26.24% (37/141)] and 41.27% (26/63: 6) [9.52% (6/63)], respectively (Figure 4B). Therefore, our results showed that the circularization step of MGISEQ-2000 not only biased the selection of DNA fragment size but also may have a greater impact on samples with quality grade B or C.

### Fragment Size Loss had no Probe Preference and was not Obvious in the Database of MGISEQ-2000 Libraries.

**FIGURE 5:**
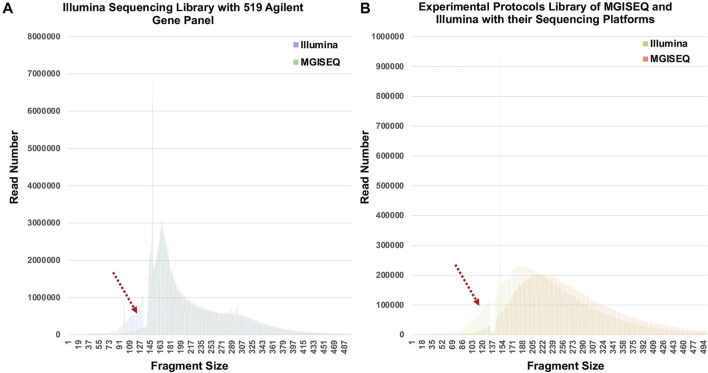
Fragment Distribution in Illumina Nextseq500 and MGISEQ-2000. **(A)** The libraries were constructed following the instructions of Illumina and captured with an Agilent 519 gene panel. (**B)** Fragment distribution when experiments were performed according to the experimental systems and kits recommended by Illumina and MGISEQ, respectively.

To verify whether the phenomenon was related to capture-probe preference, we analyzed the fragment size distribution of the sequencing data from 34 samples that were captured with an Agilent 519 gene panel and sequenced separately by Illumina Nextseq500 and MGISEQ-2000. As shown in Figure 5A, the same 101–133 bp fragment size loss was found. In addition, we constructed 34 other libraries according to the experimental protocols of MGISEQ and Illumina and generated data on their sequencing platforms. We also analyzed the fragment size distribution and found that the fragment size (peak 183 bp) distribution on the Illumina platform had a “left offset” compared to that (peak 214 bp) on the MGISEQ-2000 platform. The fragment size distribution curve of the MGISEQ data was smooth, and there was no obvious 101–133 bp fragment size loss (Figure 5B).

## Discussion

In recent decades, next-generation sequencing technology has undergone rapid development. With the greatly reduced sequencing cost, increasing scientific research and technical product development are being applied to NGS. In particular, to meet the needs of precision medicine and big data mining, the number and scale of cancer omics research and clinical projects are constantly increasing (Yang et al., 2020b; Zeng et al., 2020). For a large number of samples, the expenses and costs borne are unaffordable; thus, sequencing costs are still the bottleneck for large-scale NGS applications. At present, Illumina sequencers dominate the high-throughput sequencing market, but MGI sequencers based on DNB technology have gradually become more popular worldwide. Recently, several studies have compared the performance of BGI-500 and the Illumina HiSeq machine and showed that both of them could produce high-quality data in various applications. However, a comparison of their quality for capture panel sequencing (except WES), which is widely used in tumor research, has not been published.

In this study, we compared the data produced from the same library by different sequencing platforms. For the library preparation step, Illumina used bridge PCR technology, while MGI achieved single-molecule template amplification by DNB circularization amplification. We applied both the Illumina (Nextseq500 and MiSeqDx) platform and MGISEQ (MGISEQ-2000) platform to the same library constructed by the Illumina protocol. Theoretically, any difference in sequencing data should have been caused by the differences between bridge PCR and circularization amplification or the consequent sequencing system differences. Comparison of the data analysis results revealed the disadvantage of fragment size selection and short fragment size ligation efficiency in the circularization step. These results suggest that the sequencing data based on Illumina library preparations and in which sample types with shorter fragment sizes (such as hemolyzed plasma samples) or a more complex distribution of DNA fragment sizes (such as FFPE samples with longer storage times) are used may encounter short DNA fragment size loss on the MGISEQ sequencing platform. Therefore, we should evaluate the compatibility of sequencing libraries and sequencing platforms for scientific research that focuses on the distribution of fragment size, especially for small RNA (Fehlmann et al., 2016), cell-free DNA (cfDNA) and circulating tumor DNA (ctDNA) research (Underhill et al., 2016; Liu et al., 2020). Although the sequencing library is basically compatible with different sequencing platforms, appropriate experimental systems and sequencing platforms should be selected based on the research purpose and sample type. Otherwise, there may be an unexpected impact on the sequencing results. Our data showed the results of only target capture panel sequencing; the assessment of other sequencing applications requires further investigation.

Considering that the alignment algorithm may also have an impact on the fragment size distribution analysis, we replaced the BWA “aln” algorithm mentioned in the article with the BWA “mem” algorithm. The “mem” algorithm is much looser than the “aln” algorithm, and it can perform local alignment and splicing. The “mem” algorithm allows multiple different parts of the sequencing reads to have their own optimal matches, resulting in multiple optimal alignment positions for the reads and greatly improving the alignment rate. After comparing and analyzing the combined data with 204 samples of the IDT 38-hotspot gene panel and 34 samples of the Agilent 519 gene panel by using the “mem” algorithm, we found that the number of reads in the 101–133 bp fragment size from the MGISEQ-2000 platform data was significantly improved (Supplementary Figure S1), but there were still significant differences, with *t*-test *p*-values of 0.0277 and 0.0252, respectively. The conclusion was consistent with that based on the “aln” algorithm.

We also found that the data without the 101–133 bp fragment size loss were derived from different sequencing read lengths of the Illumina Nextseq500 and MGISEQ-2000 platforms, while the data with the same sequencing read length showed the 101–133 bp fragment size loss. To investigate whether the data with or without the phenomenon were related to the sequencing read length, we reanalyzed and compared data with the same number of sequencing reads but not read length, and found that the results were consistent with the previous conclusion. Since the 101–133 bp fragment size loss was concentrated in the data with long read length (150 bp) but not in the data with short read length (100 bp), we hypothesized that the phenomenon may also be related to the sequencing read length. We will conduct more in-depth research on this point in our future work.

In summary, the MGISEQ-2000 platform has good compatibility with Illumina sequencing libraries, but the DNB circularization step may cause fragment size selection or have low ligation efficiency for short DNA fragment sizes. For the accuracy of downstream data analysis, we recommend that different sequencing platforms should be used with their official experimental systems and kits. If the experiment needs to change between different platforms, for cost considerations or other reasons, the selected platform should be evaluated carefully with respect to the purpose of the research or actual needs, as it may have a significant impact on outcomes. In the future, it would be interesting to compare the performances of two platforms in specific applications like cancer diagnosis (He et al., 2020b; Peng L.-H. et al., 2020), prognosis (Peng et al., 2020c; Song et al., 2020; Zhou et al., 2020), evolution inference (Yang et al., 2013; Yang et al., 2014), drug repositioning (Peng et al., 2015; Zhou et al., 2019; Liu et al., 2020), and so on. However, it is out of the scope of this study.

## Data Availability

The data has been uploaded to NCBI - BioProject 744584.
