# Supplementary figures and images for: Evaluation of the MGISEQ-2000 Sequencing Platform for Illumina Target Capture Sequencing Libraries (part 1 of 6)
Source: Front Genet. 2021 Oct 27;12:730519. doi: 10.3389/fgene.2021.730519 (PMC8578046; doi:10.3389/fgene.2021.730519)

Sequencing Depth

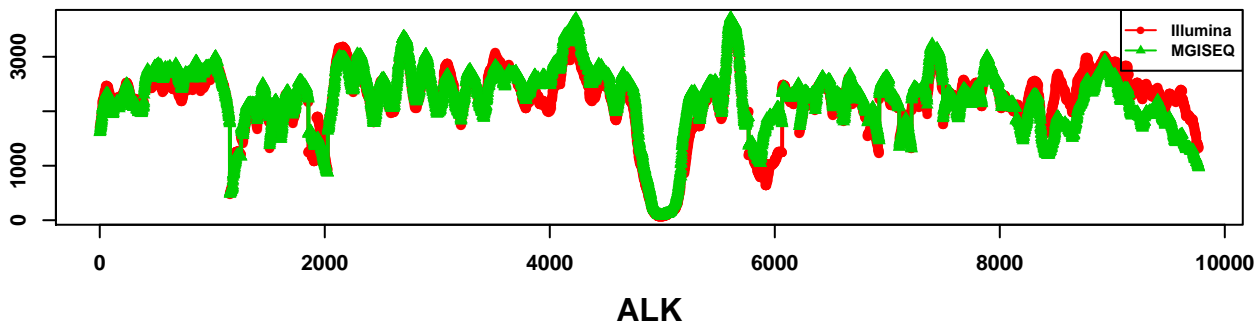

Sequencing Depth

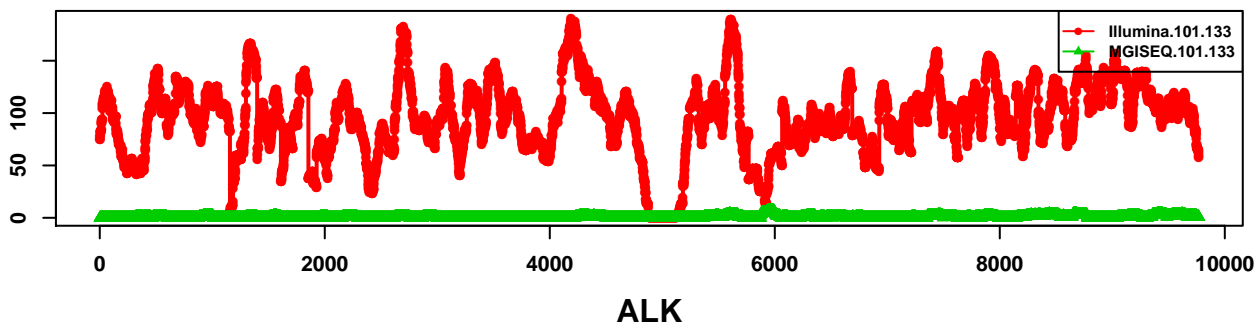

Sequencing Depth

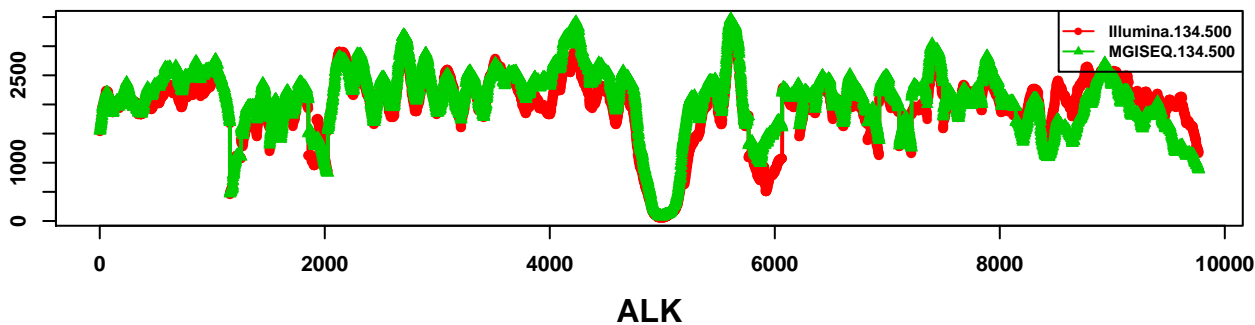

Supplement: Supplementary file 1 [file Presentation4.zip › ALK/19JS48180P.pdf]

Sequencing Depth

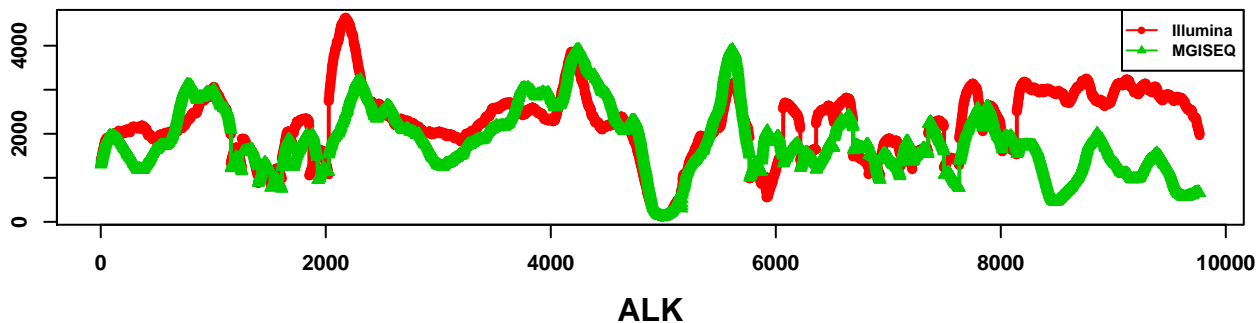

Sequencing Depth

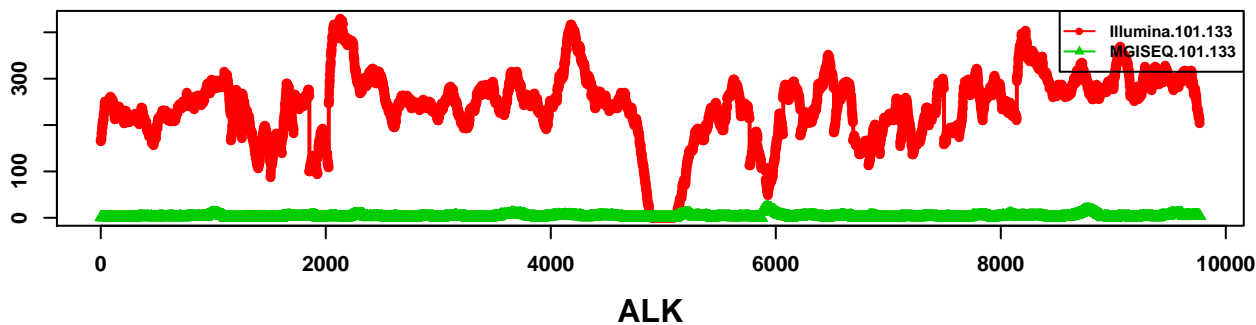

Sequencing Depth

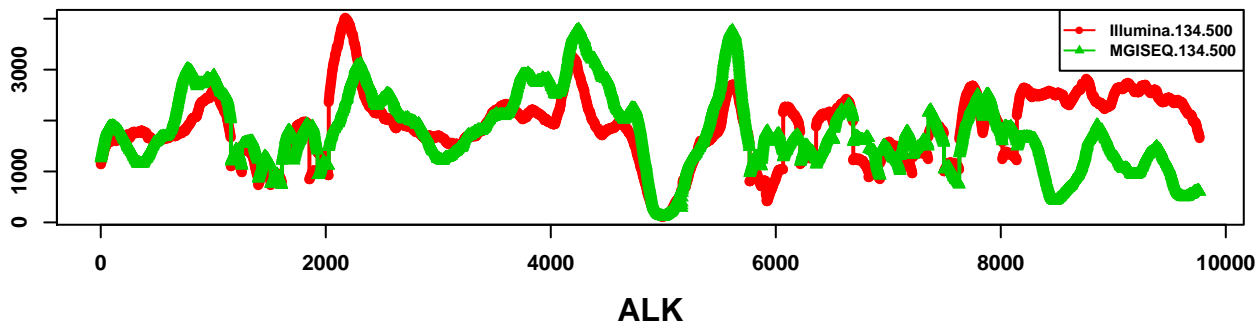

Supplement: Supplementary file 1 [file Presentation4.zip › ALK/19HE22185F.pdf]

Sequencing Depth

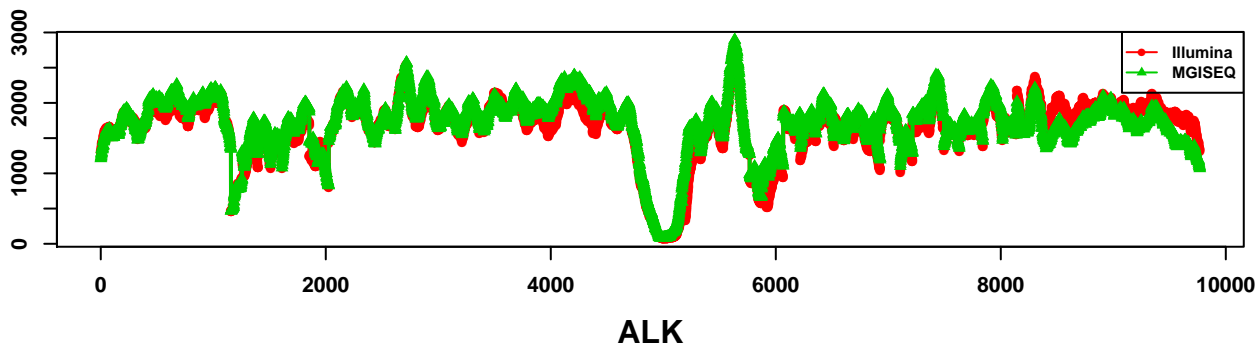

Sequencing Depth

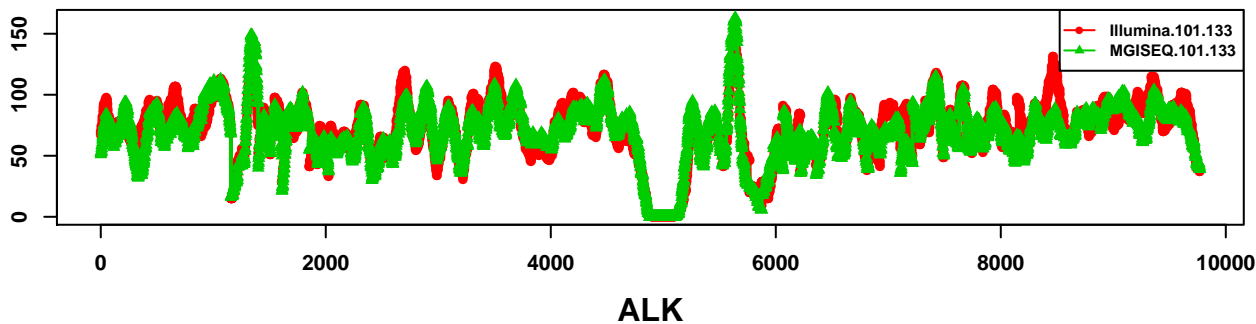

Sequencing Depth

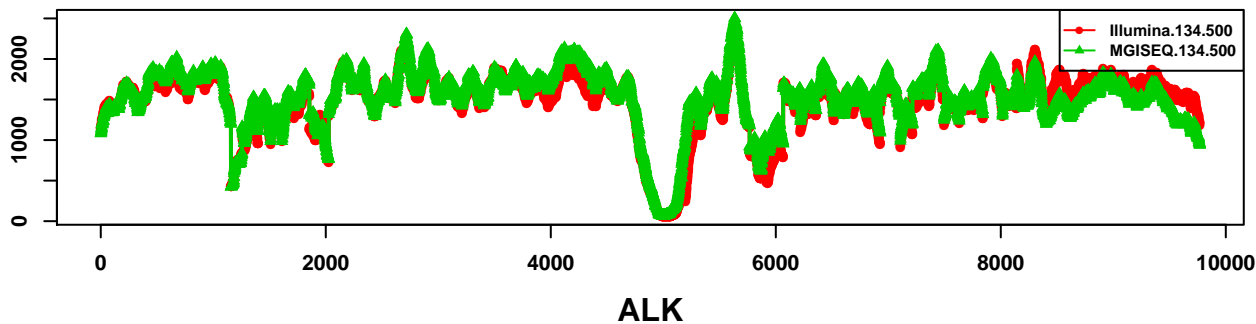

Supplement: Supplementary file 1 [file Presentation4.zip › ALK/19JS48246P.pdf]

Sequencing Depth

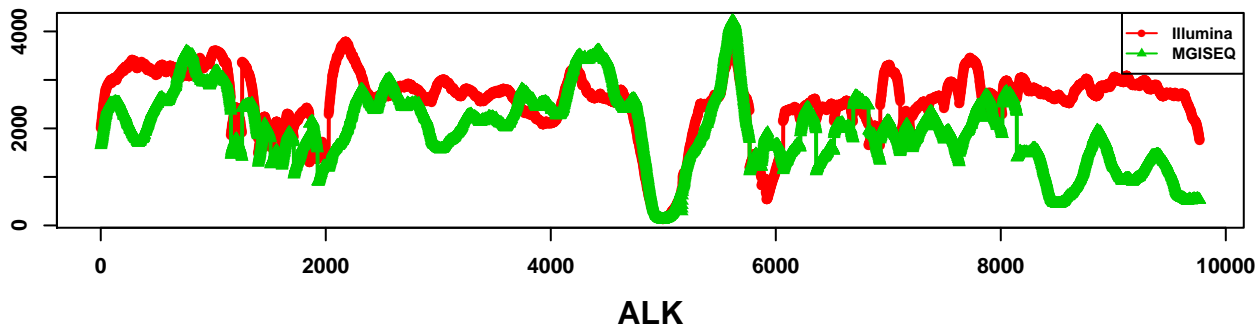

Sequencing Depth

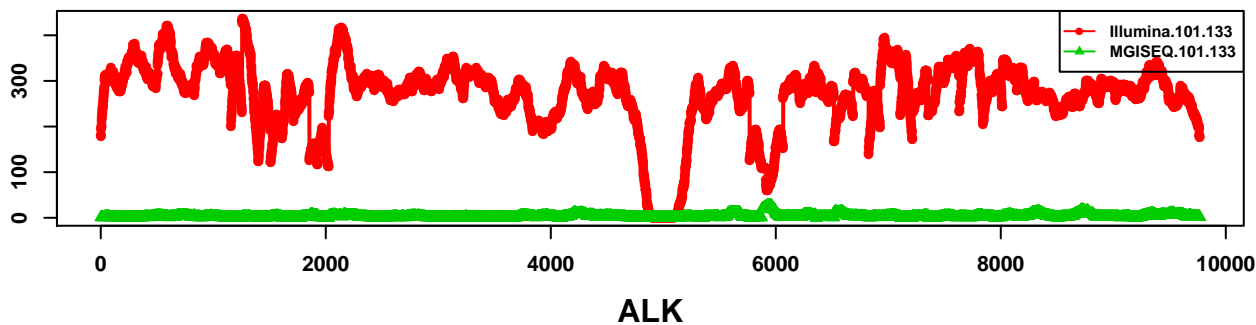

Sequencing Depth

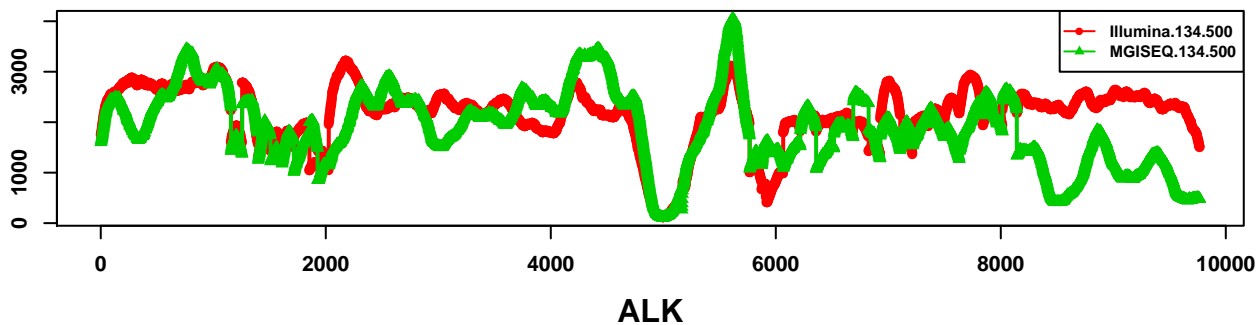

Supplement: Supplementary file 1 [file Presentation4.zip › ALK/19ZN12297F.pdf]

Sequencing Depth

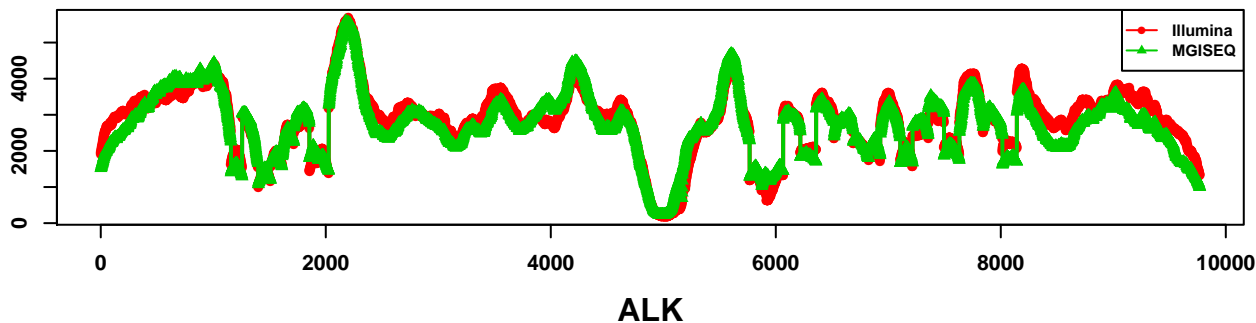

Sequencing Depth

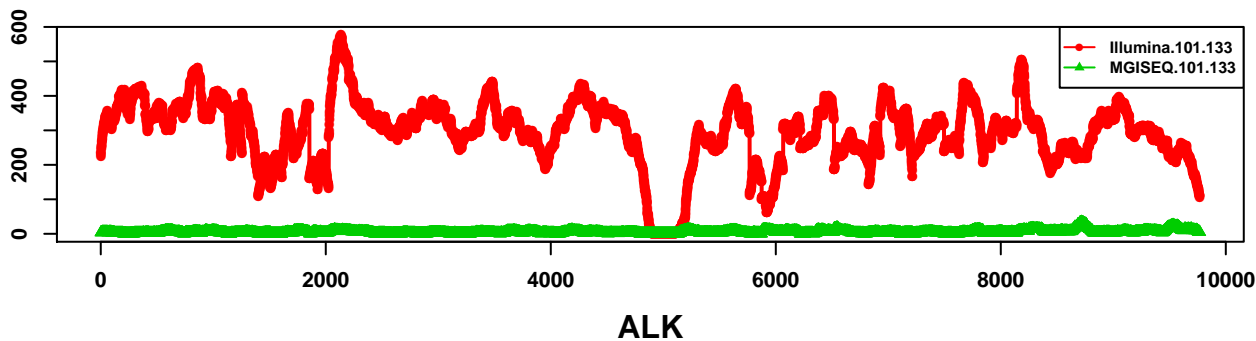

Sequencing Depth

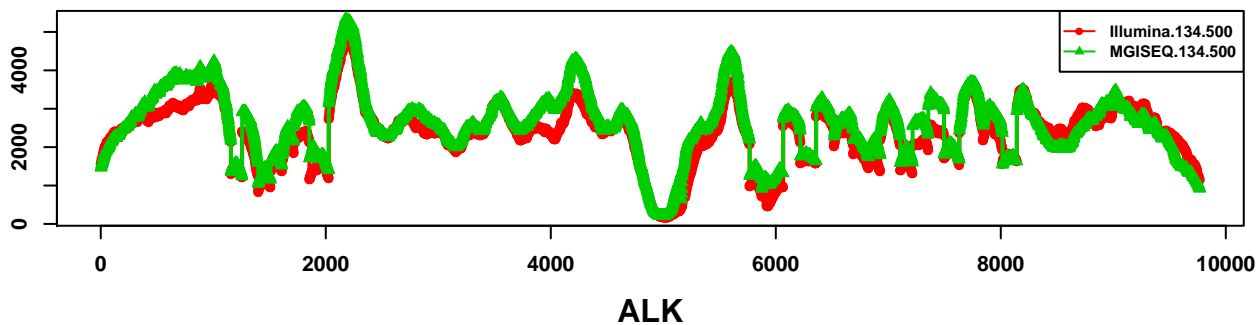

Supplement: Supplementary file 1 [file Presentation4.zip › ALK/19FC40253-IIF.pdf]

Sequencing Depth

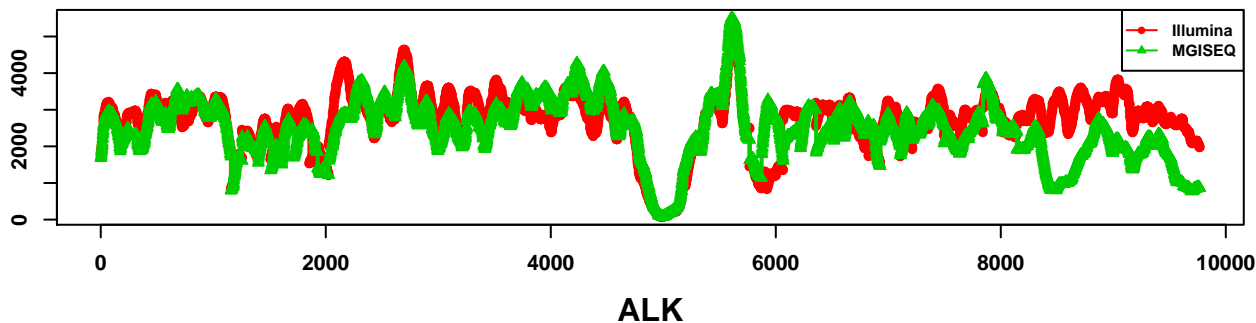

Sequencing Depth

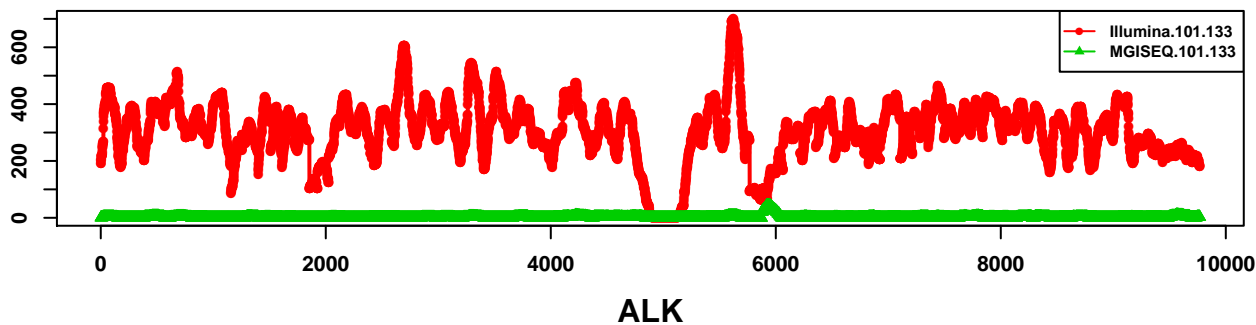

Sequencing Depth

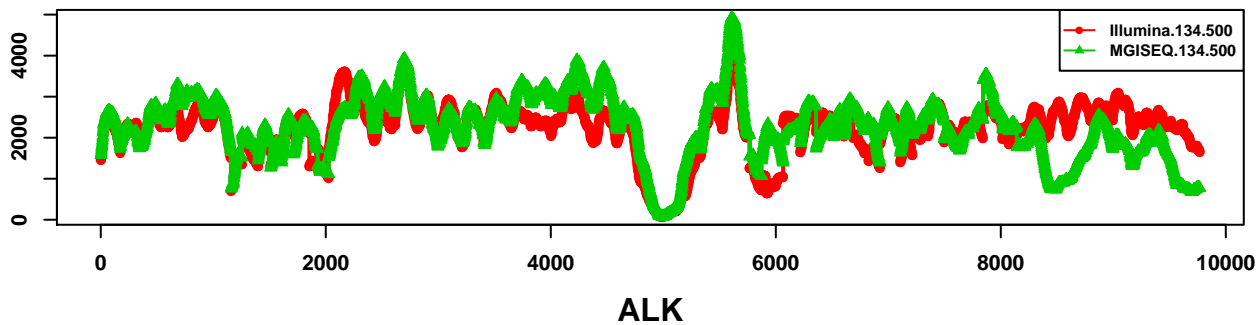

Supplement: Supplementary file 1 [file Presentation4.zip › ALK/19ZN13097P.pdf]

Sequencing Depth

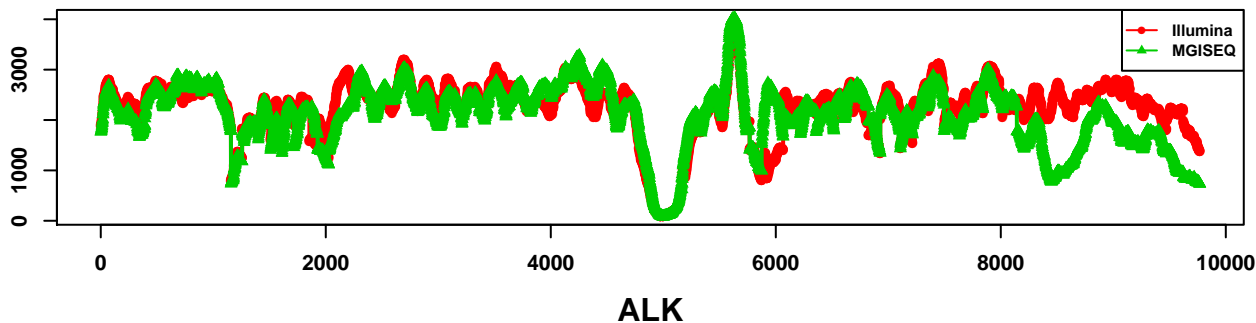

Sequencing Depth

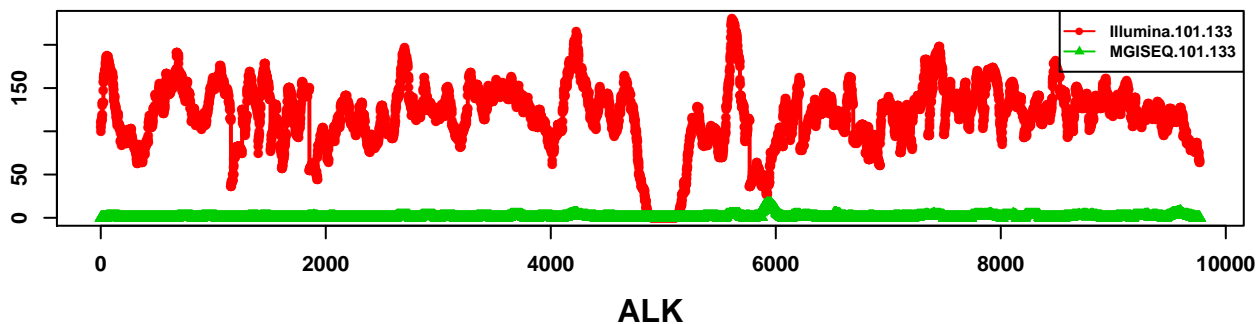

Sequencing Depth

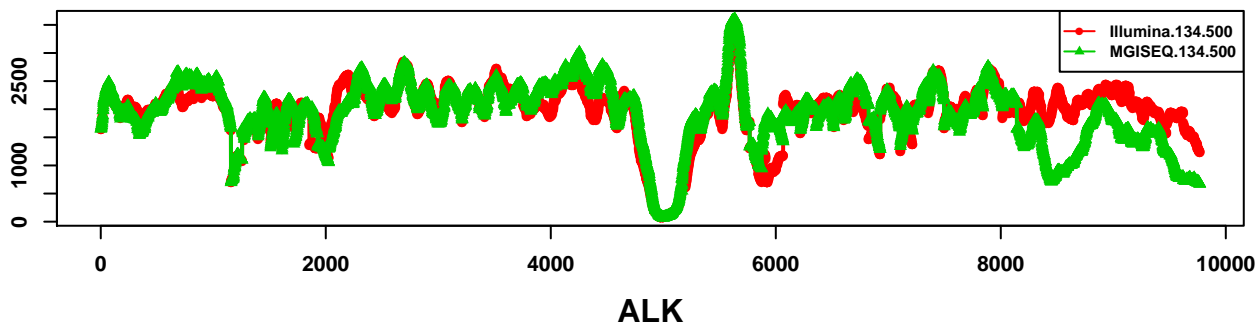

Supplement: Supplementary file 1 [file Presentation4.zip › ALK/19GY94044P.pdf]

Sequencing Depth

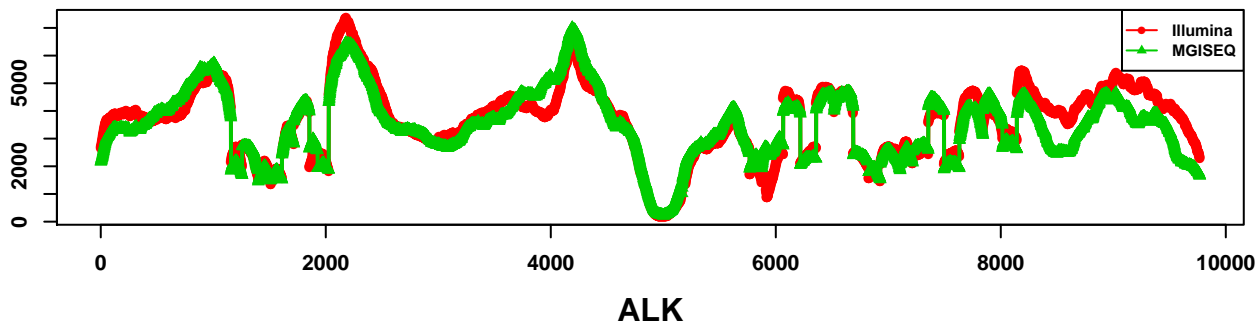

Sequencing Depth

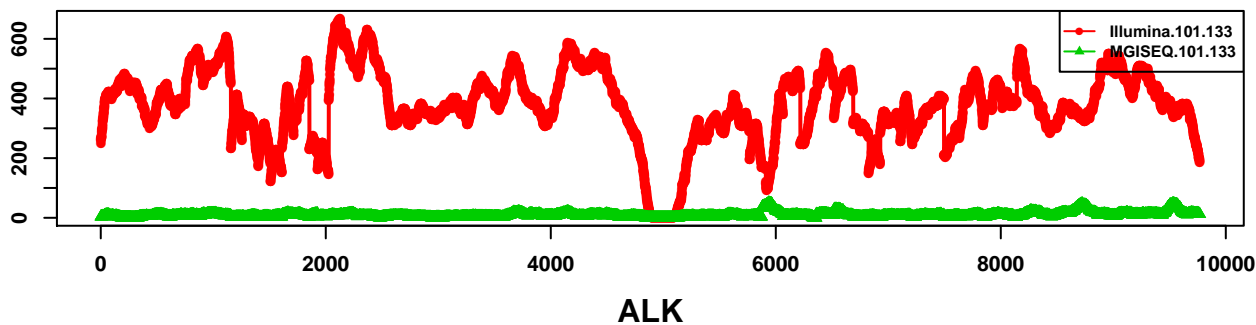

Sequencing Depth

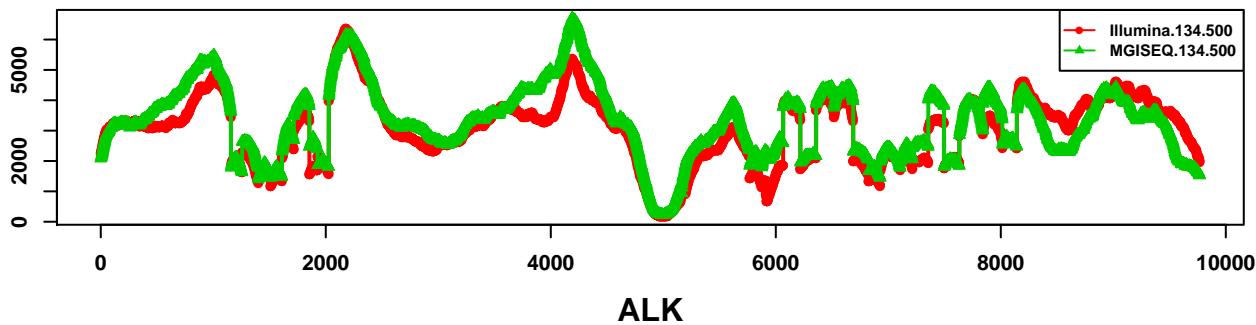

Supplement: Supplementary file 1 [file Presentation4.zip › ALK/19JS48179F.pdf]

Sequencing Depth

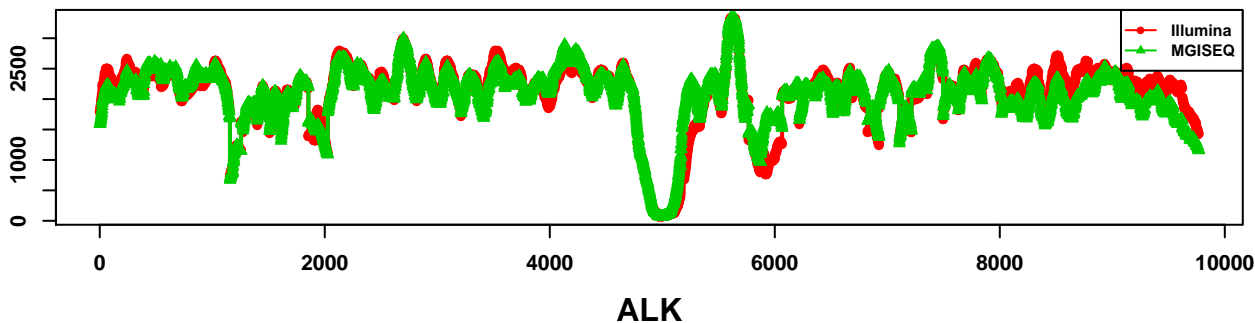

Sequencing Depth

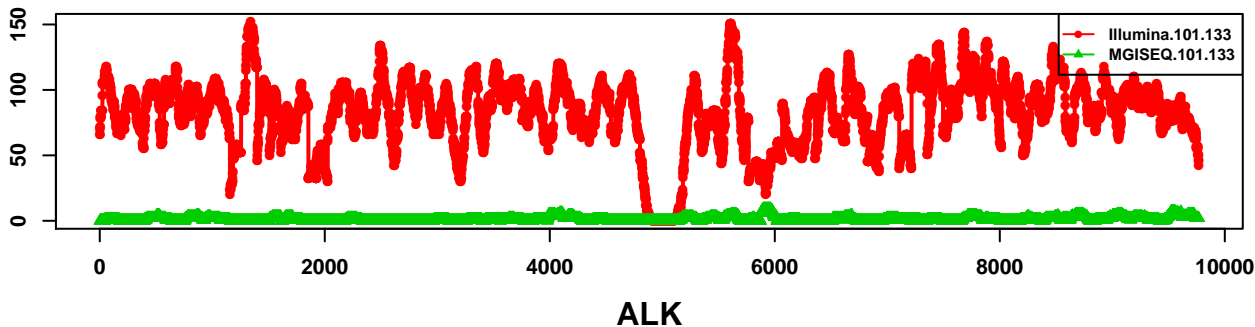

Sequencing Depth

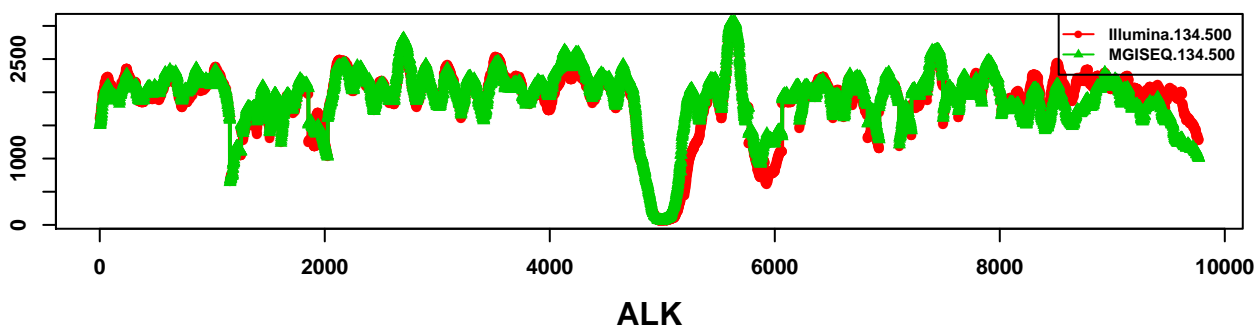

Supplement: Supplementary file 1 [file Presentation4.zip › ALK/19N01682P.pdf]

Sequencing Depth

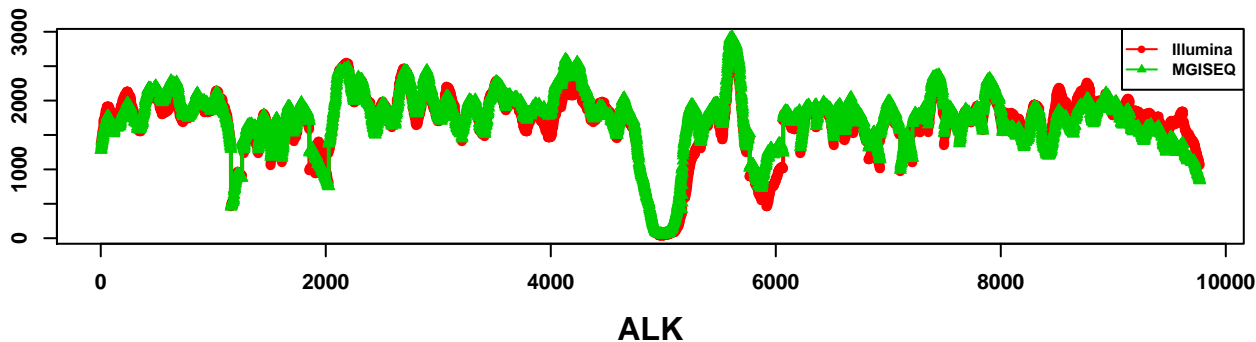

Sequencing Depth

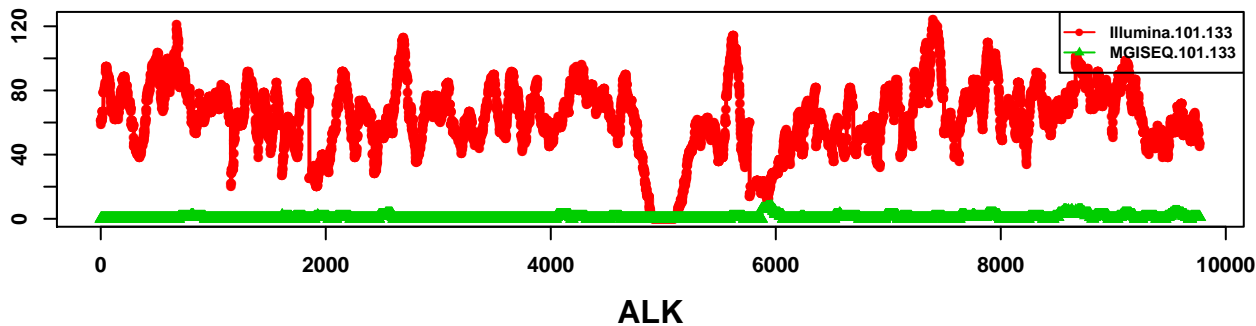

Sequencing Depth

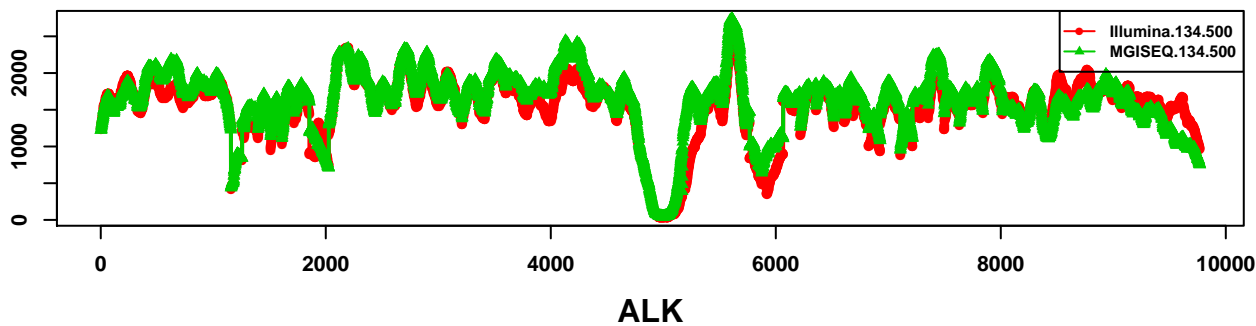

Supplement: Supplementary file 1 [file Presentation4.zip › ALK/19N01378P.pdf]

Sequencing Depth

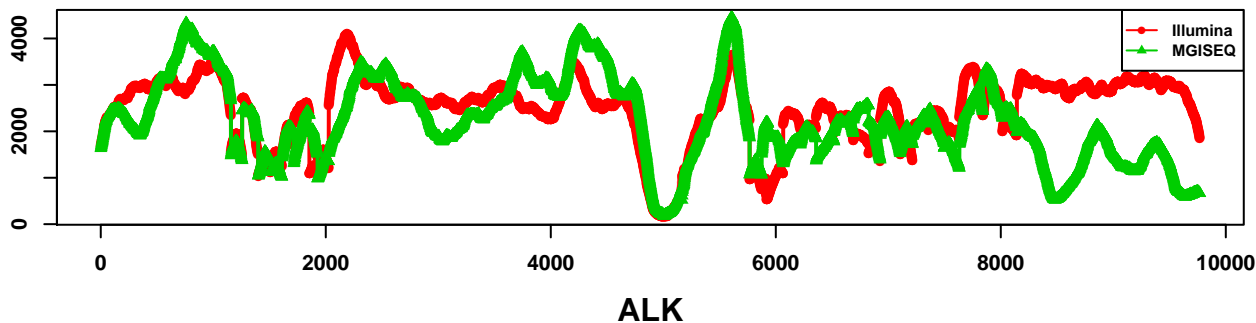

Sequencing Depth

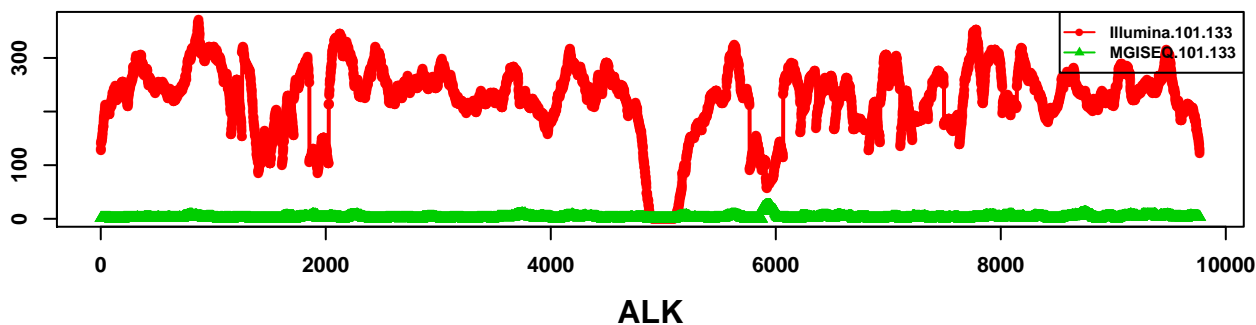

Sequencing Depth

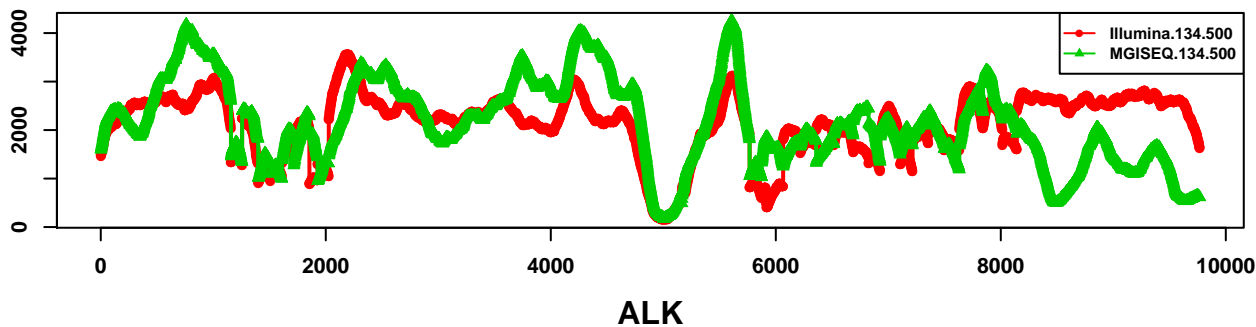

Supplement: Supplementary file 1 [file Presentation4.zip › ALK/19HE22170F.pdf]

Sequencing Depth

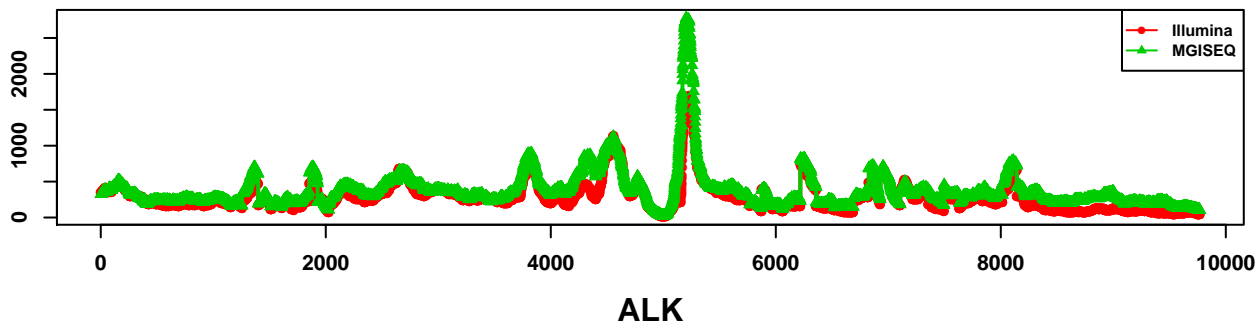

Sequencing Depth

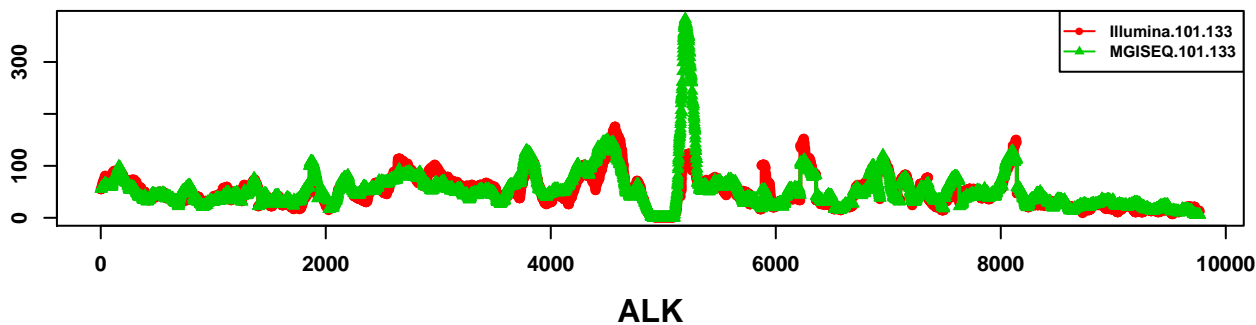

Sequencing Depth

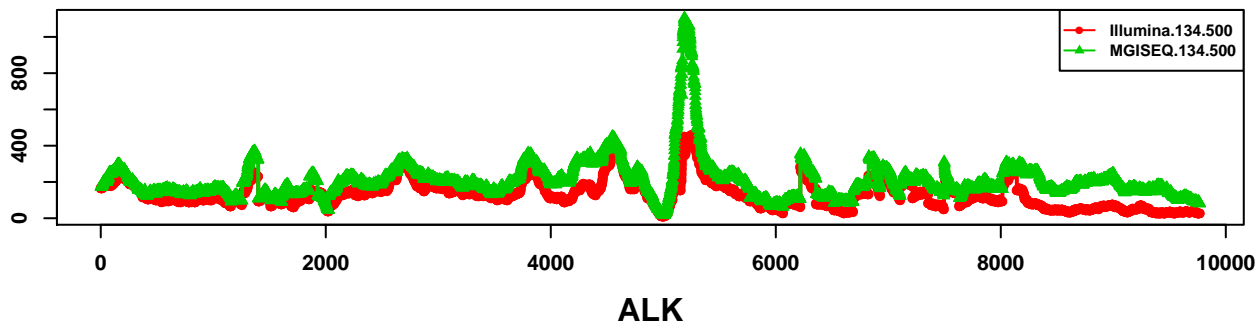

Supplement: Supplementary file 1 [file Presentation4.zip › ALK/19FC40390F.pdf]

Sequencing Depth

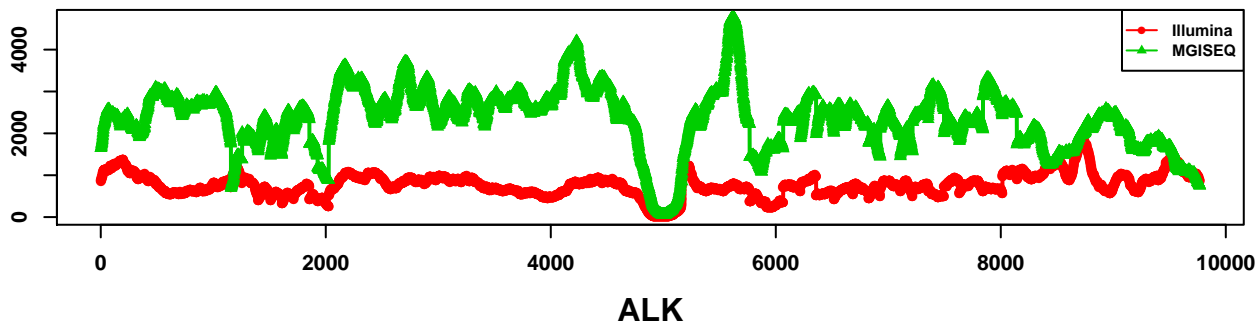

Sequencing Depth

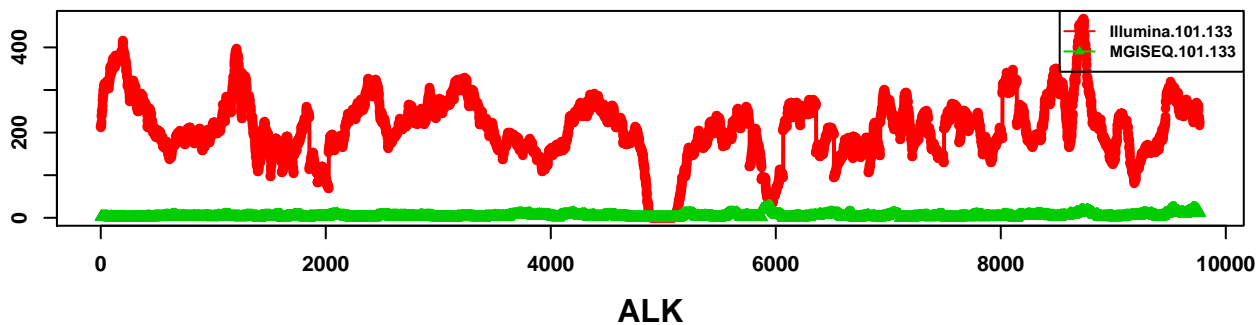

Sequencing Depth

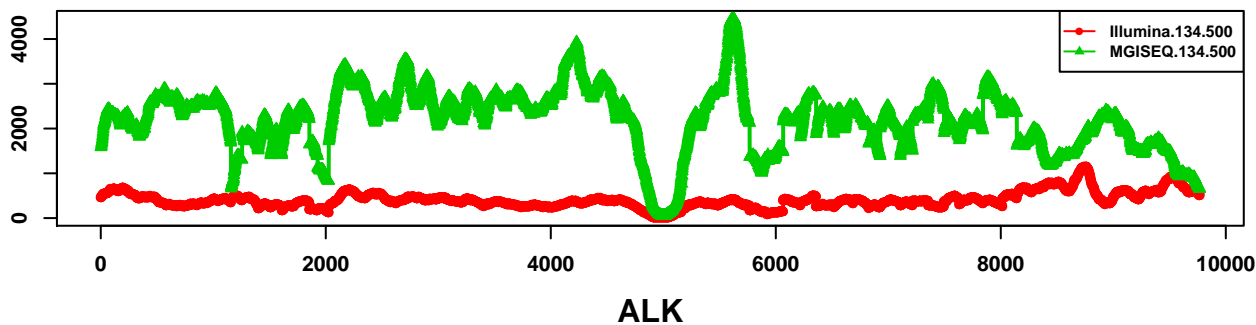

Supplement: Supplementary file 1 [file Presentation4.zip › ALK/19HE22131F.pdf]

Sequencing Depth

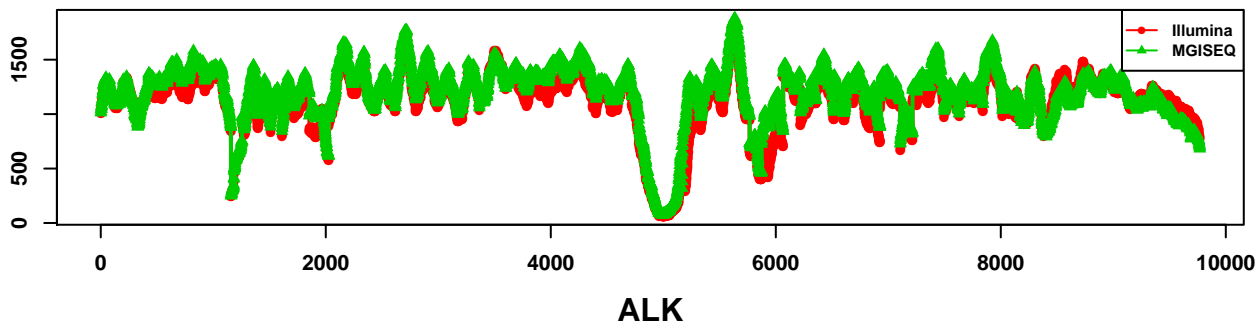

Sequencing Depth

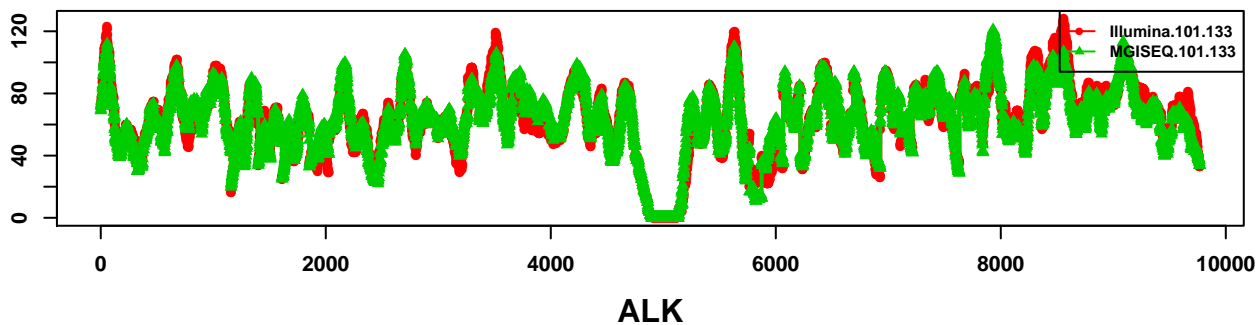

Sequencing Depth

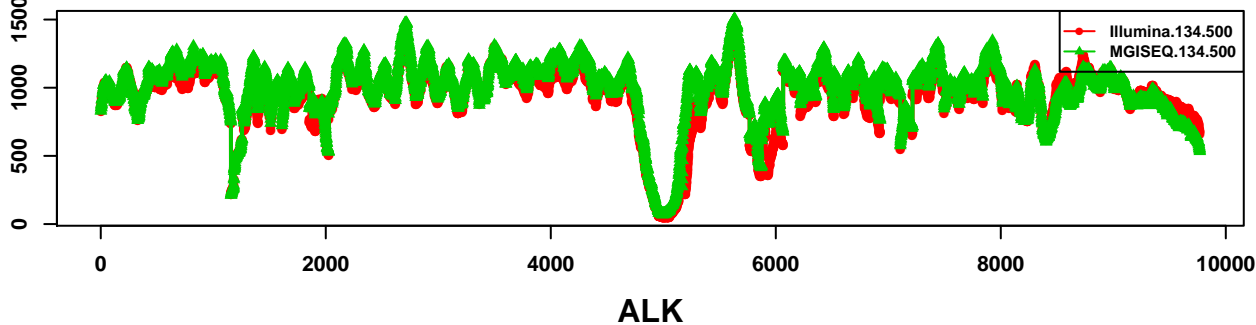

Supplement: Supplementary file 1 [file Presentation4.zip › ALK/M1901223P.pdf]

Sequencing Depth

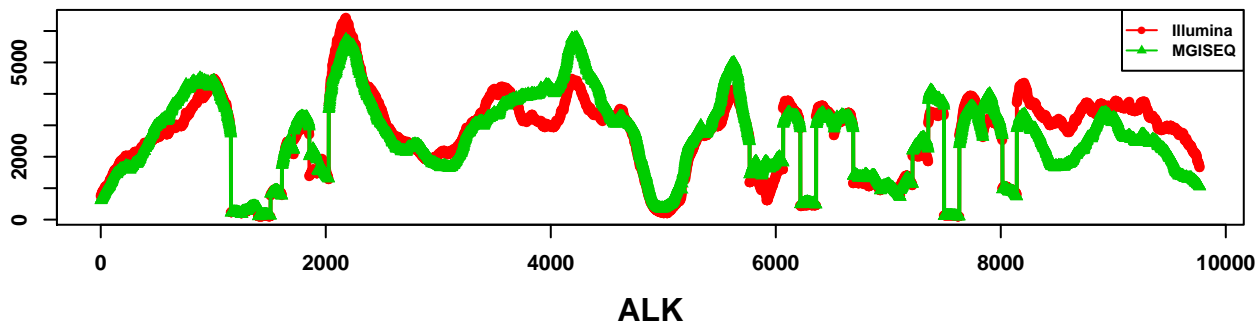

Sequencing Depth

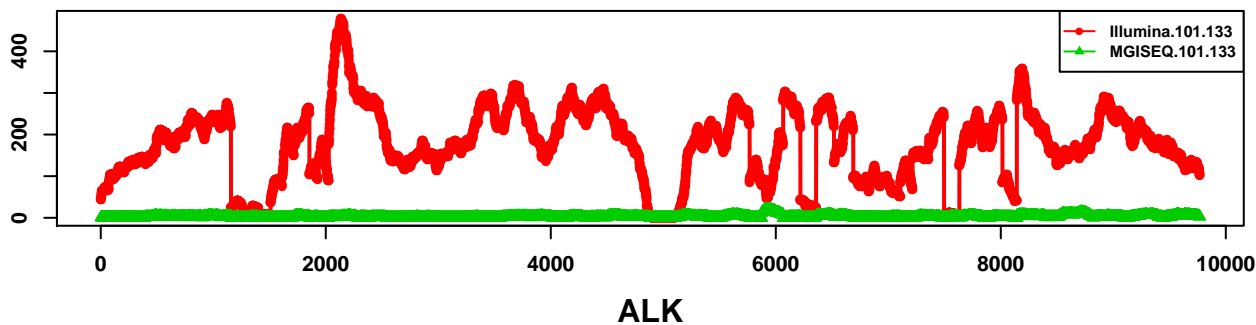

Sequencing Depth

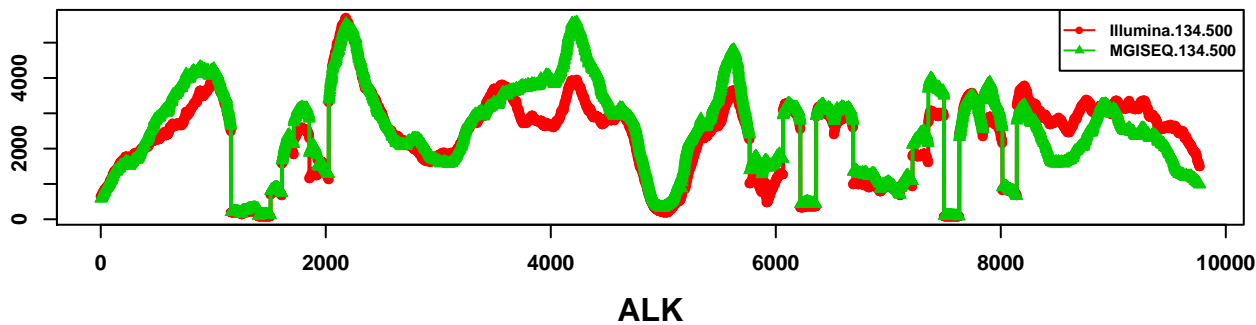

Supplement: Supplementary file 1 [file Presentation4.zip › ALK/19N01654H.pdf]

Sequencing Depth

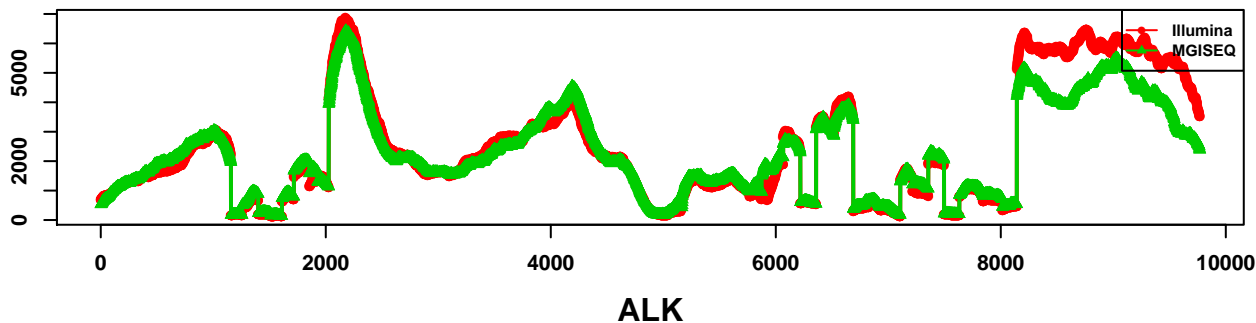

Sequencing Depth

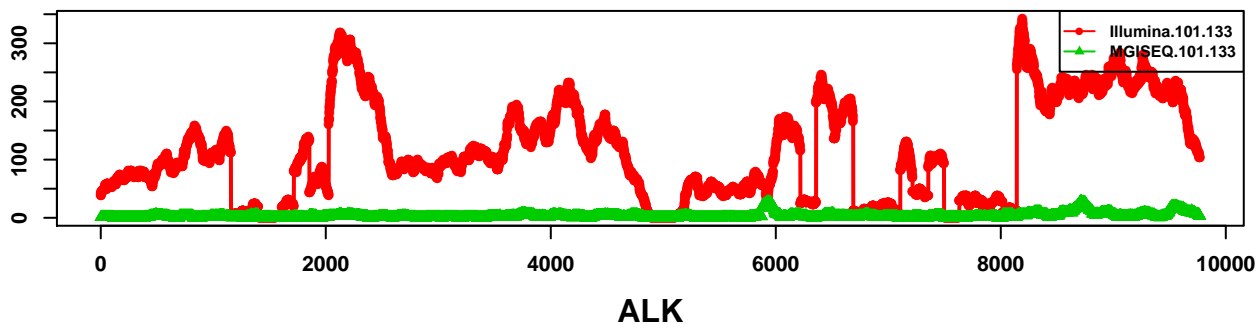

Sequencing Depth

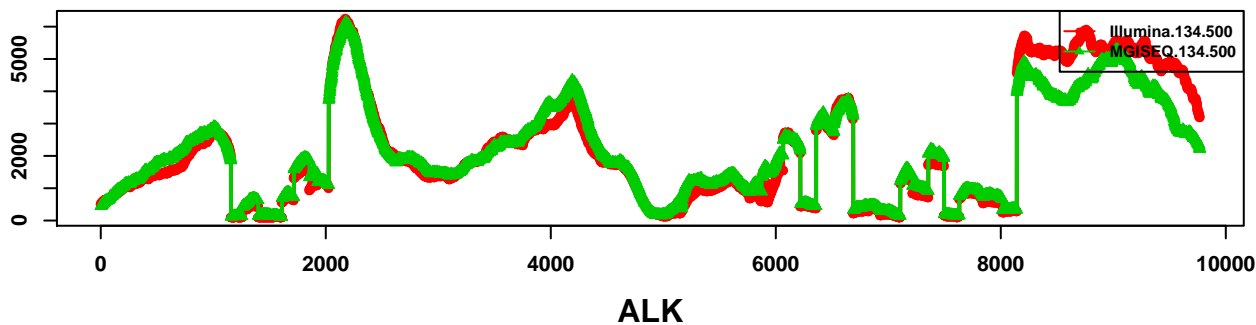

Supplement: Supplementary file 1 [file Presentation4.zip › ALK/19N01676T.pdf]

Sequencing Depth

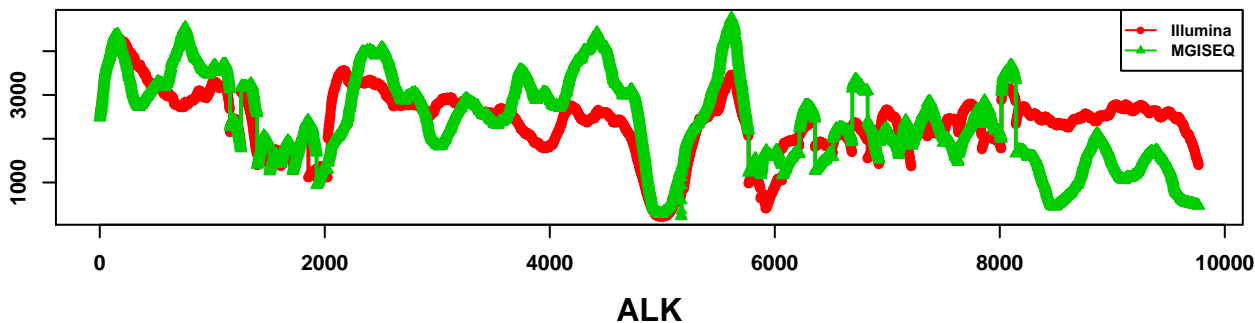

Sequencing Depth

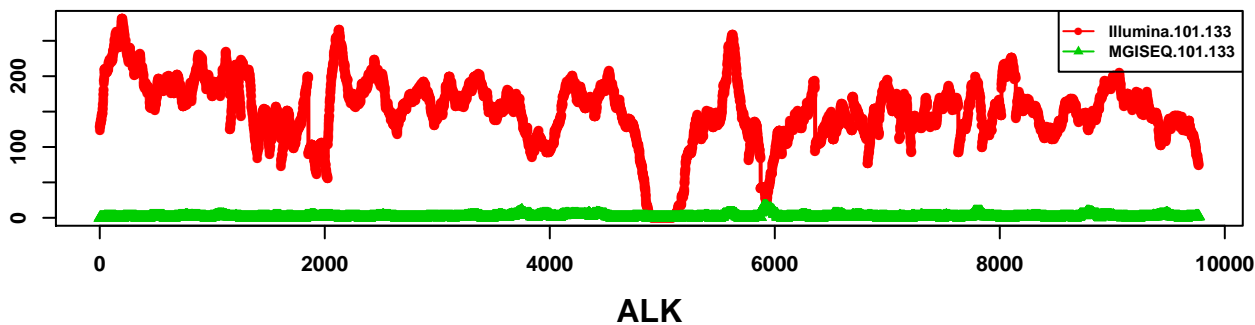

Sequencing Depth

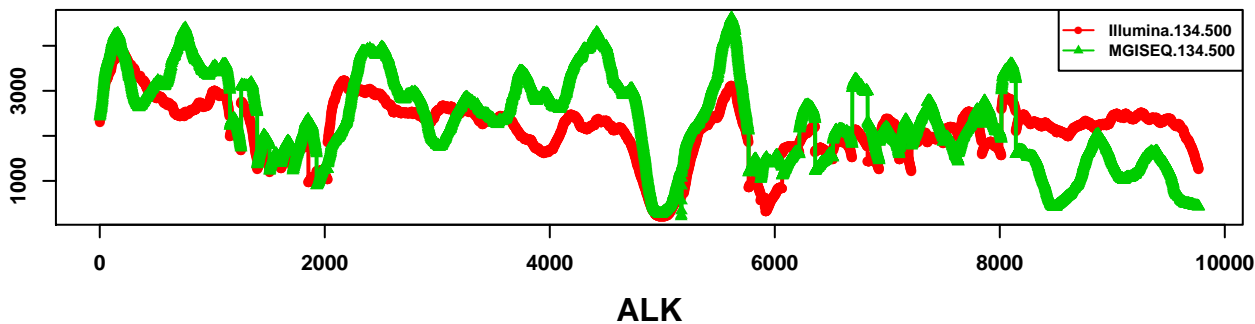

Supplement: Supplementary file 1 [file Presentation4.zip › ALK/19FC40257F.pdf]

Sequencing Depth

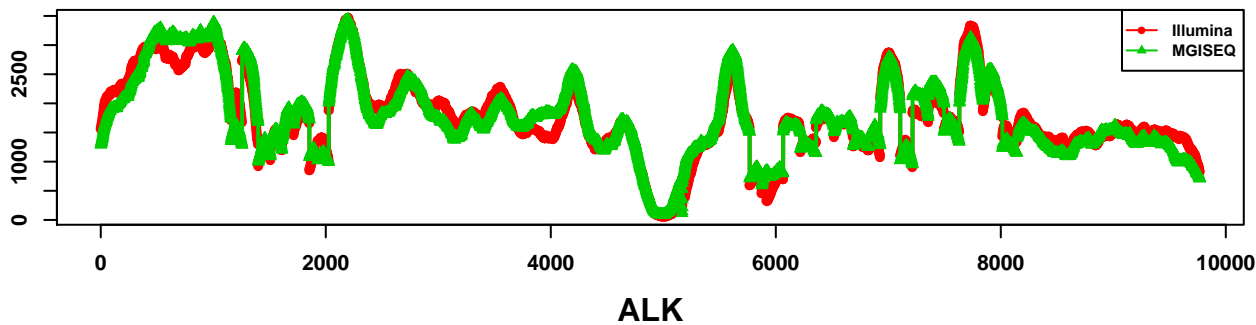

Sequencing Depth

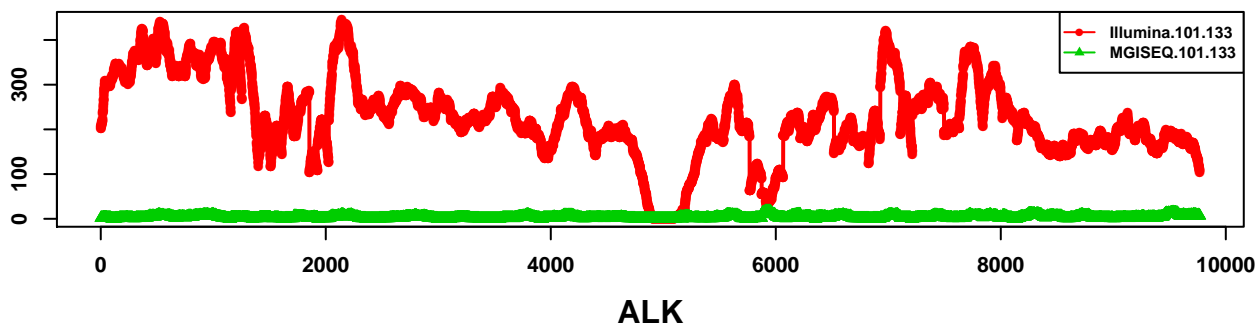

Sequencing Depth

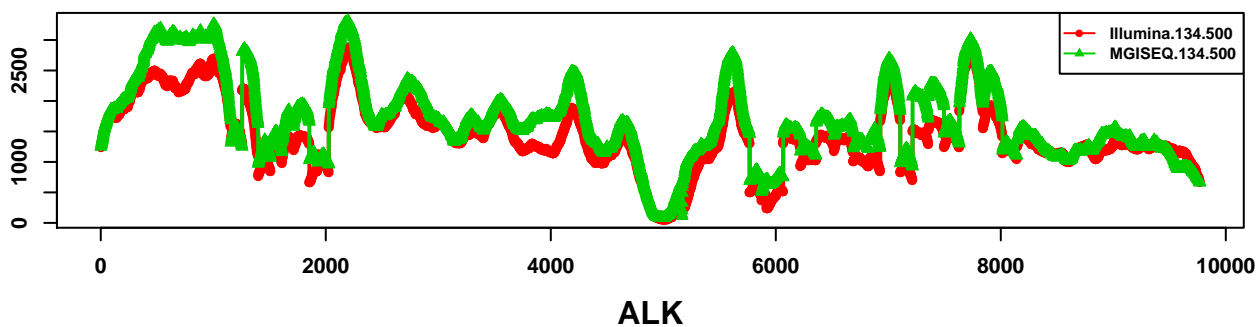

Supplement: Supplementary file 1 [file Presentation4.zip › ALK/19CF15540F.pdf]

Sequencing Depth

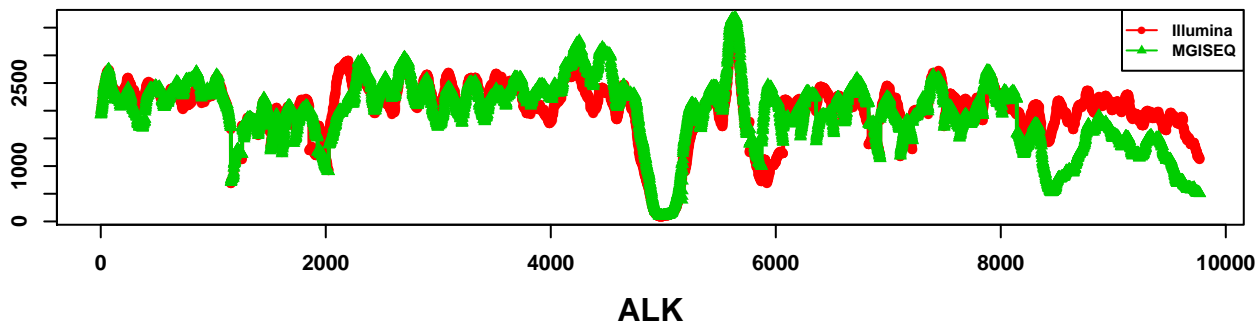

Sequencing Depth

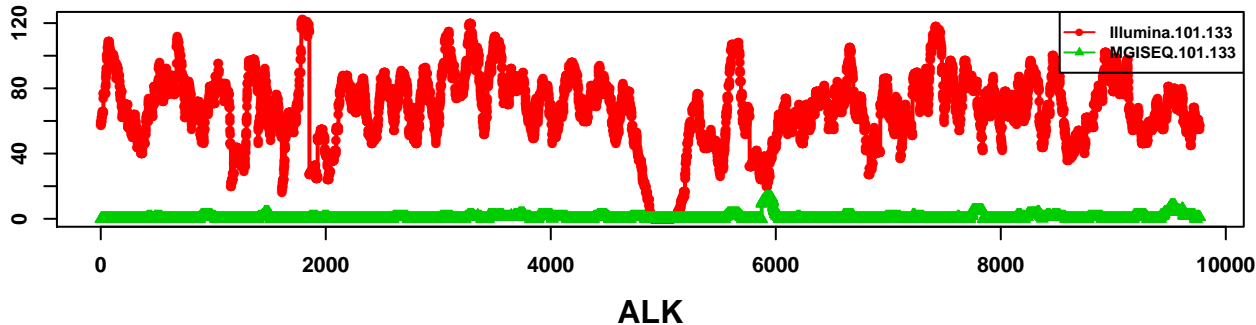

Sequencing Depth

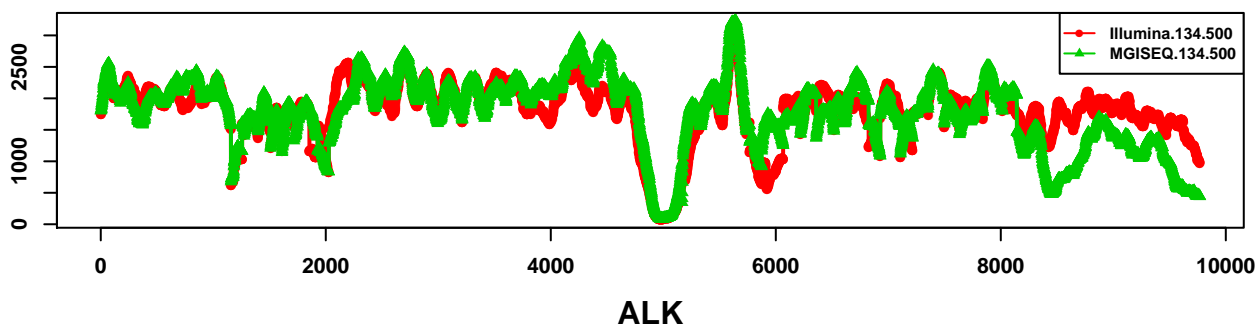

Supplement: Supplementary file 1 [file Presentation4.zip › ALK/19ZN12366P.pdf]

Sequencing Depth

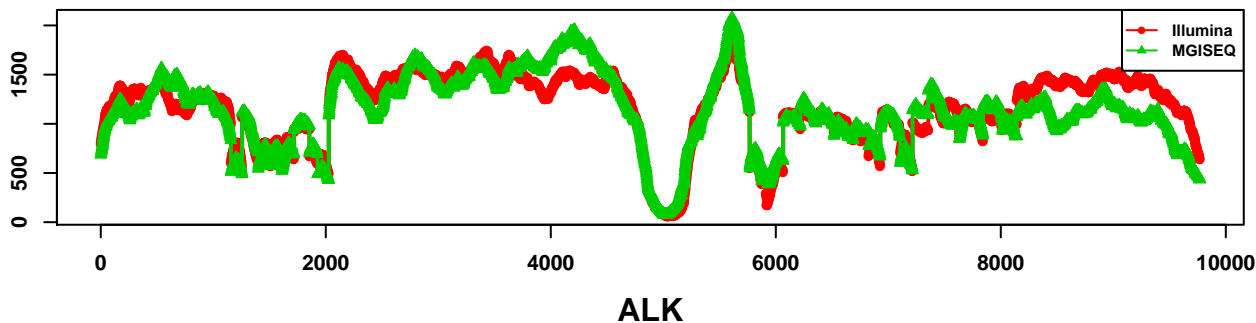

Sequencing Depth

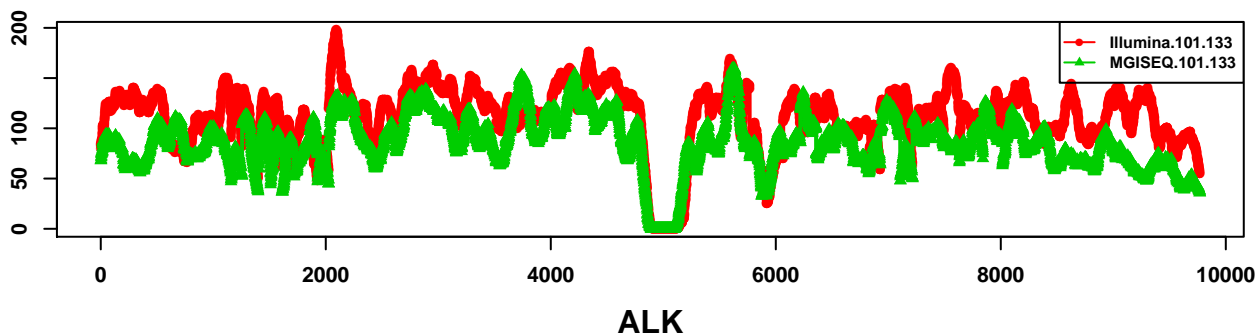

Sequencing Depth

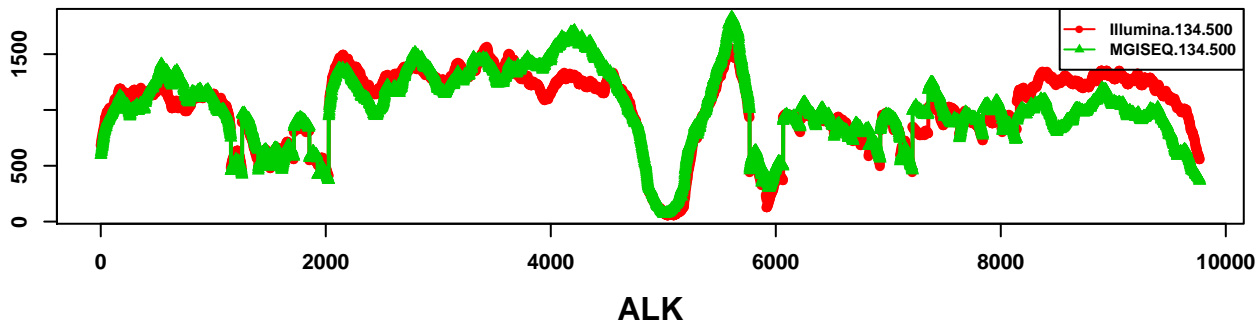

Supplement: Supplementary file 1 [file Presentation4.zip › ALK/19GY94073B.pdf]

Sequencing Depth

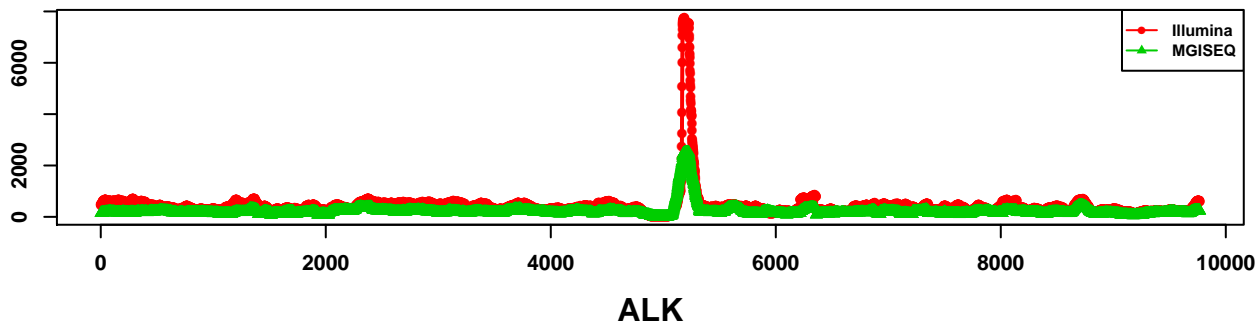

Sequencing Depth

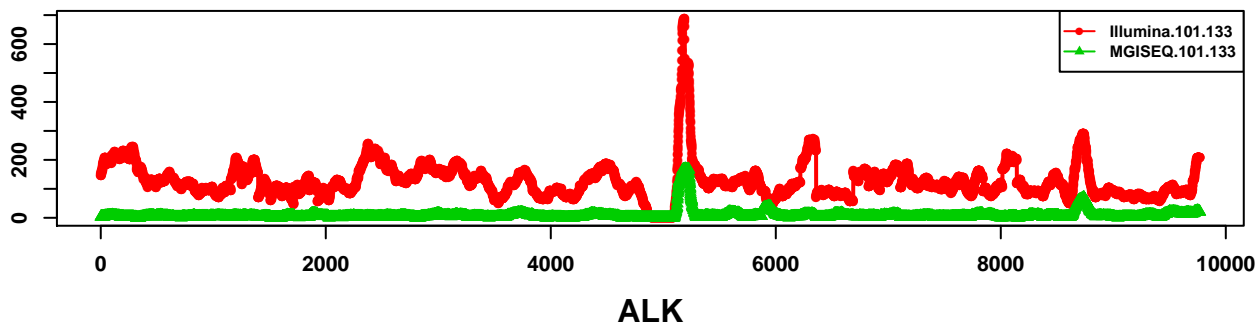

Sequencing Depth

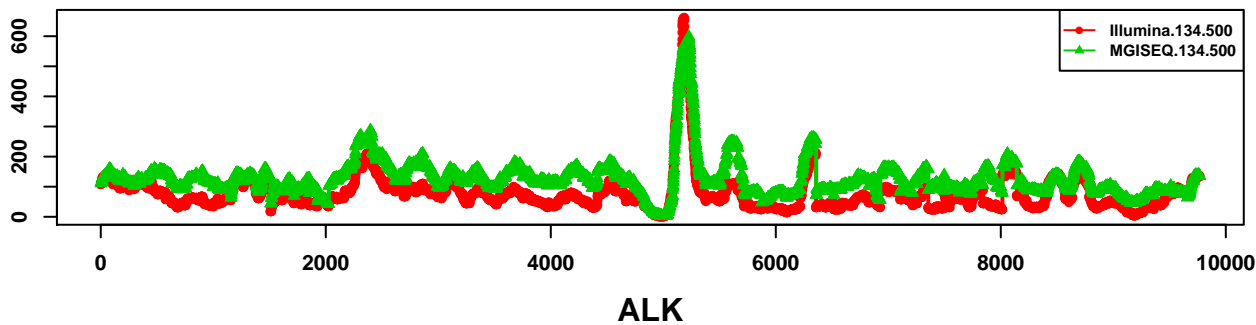

Supplement: Supplementary file 1 [file Presentation4.zip › ALK/19ZN12357F.pdf]

Sequencing Depth

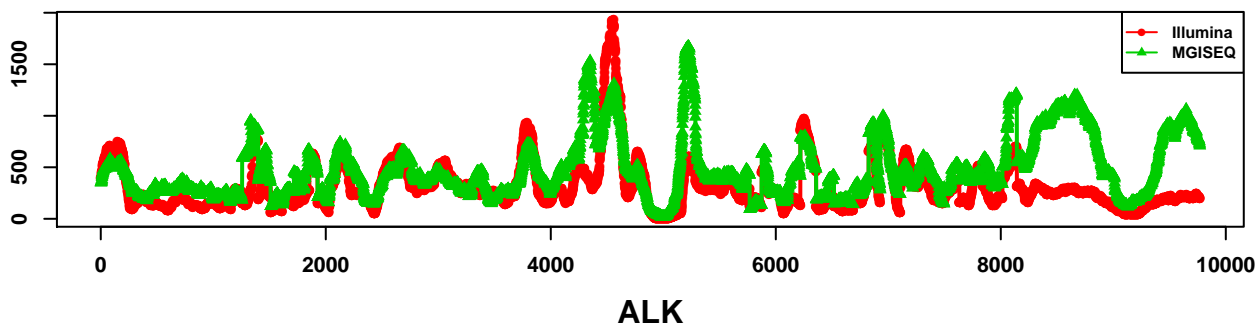

Sequencing Depth

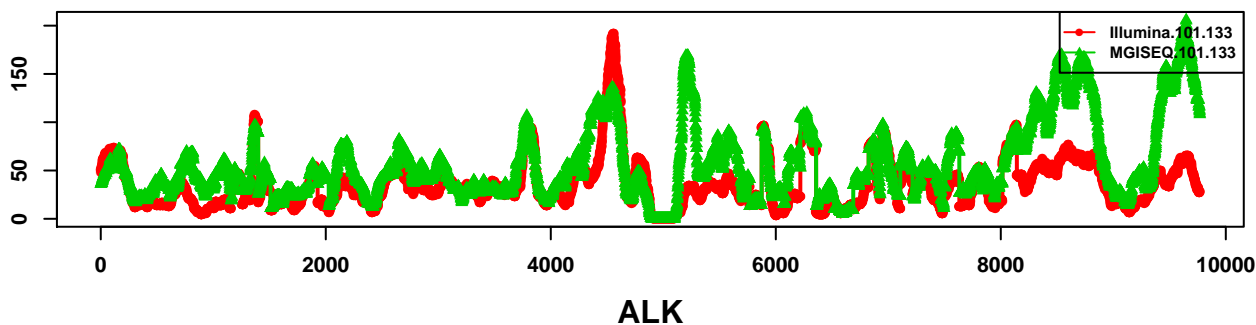

Sequencing Depth

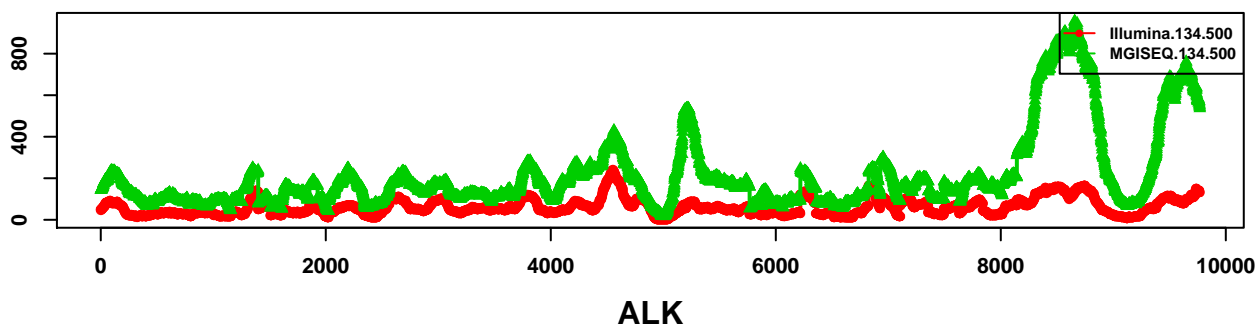

Supplement: Supplementary file 1 [file Presentation4.zip › ALK/19N02343F.pdf]

Sequencing Depth

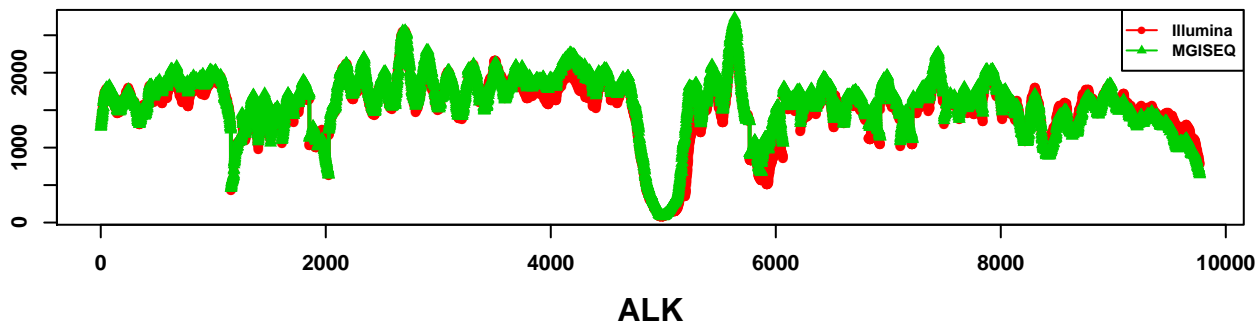

Sequencing Depth

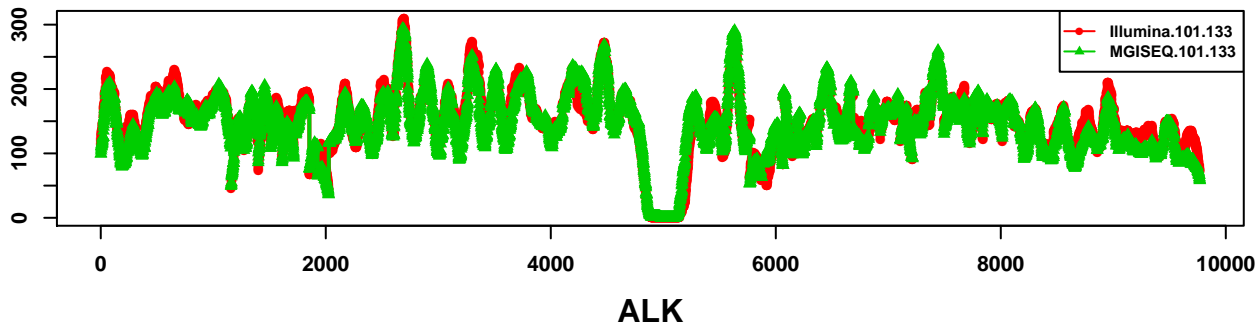

Sequencing Depth

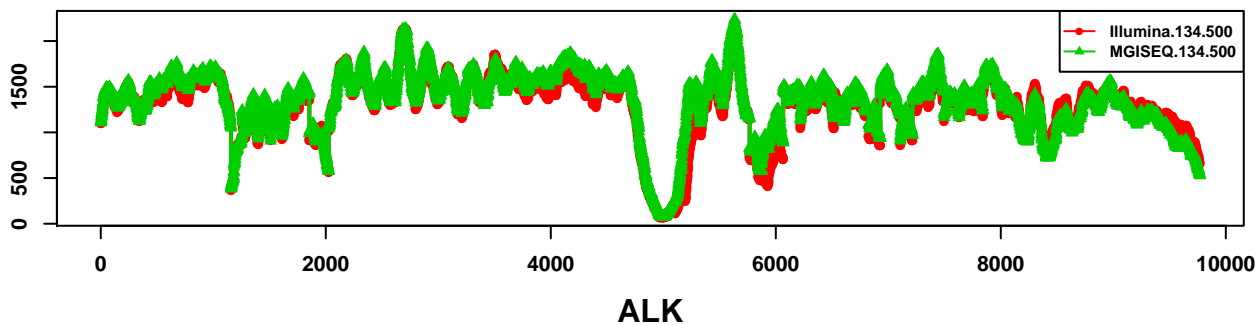

Supplement: Supplementary file 1 [file Presentation4.zip › ALK/19ZN12547P.pdf]

Sequencing Depth

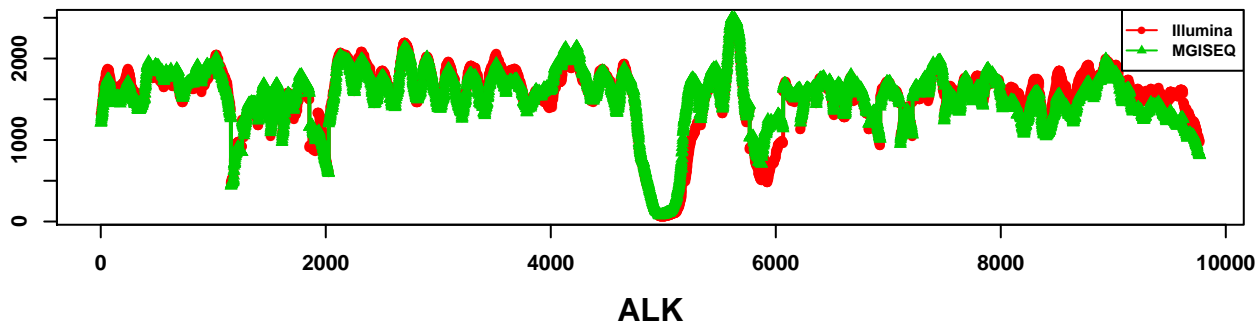

Sequencing Depth

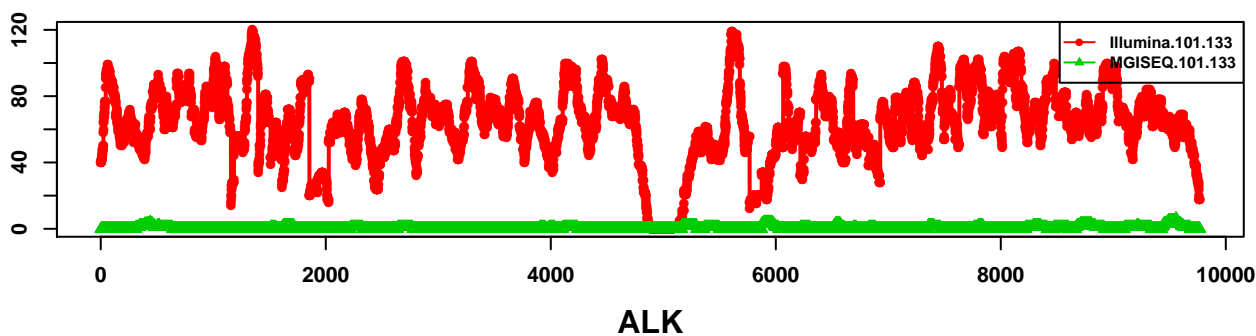

Sequencing Depth

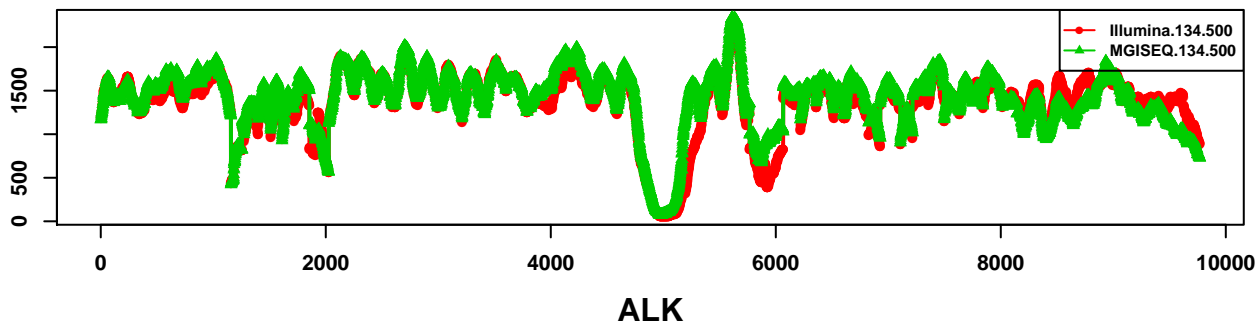

Supplement: Supplementary file 1 [file Presentation4.zip › ALK/19ZN13100P.pdf]

Sequencing Depth

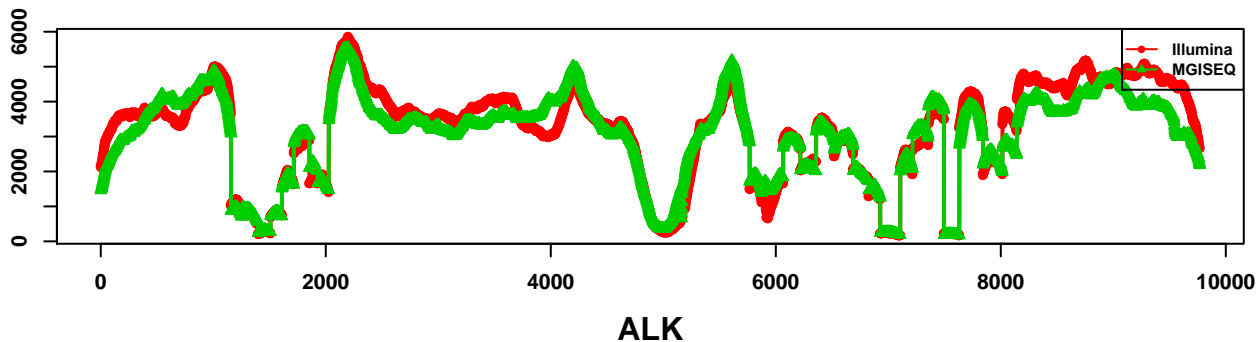

Sequencing Depth

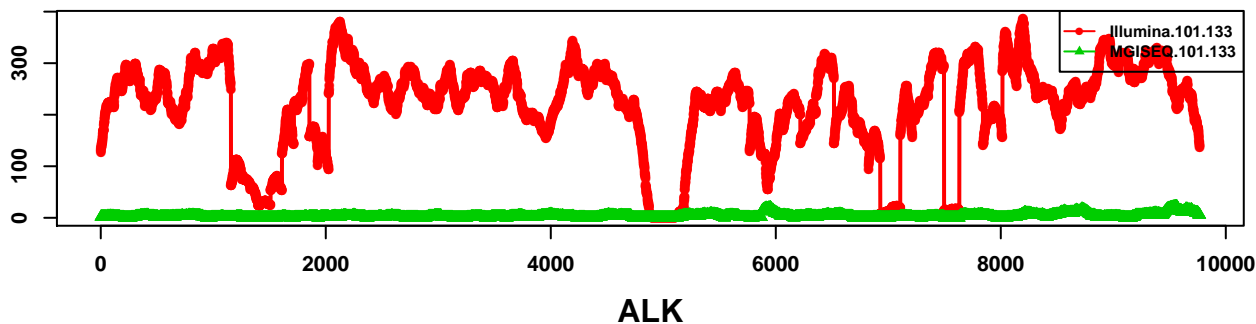

Sequencing Depth

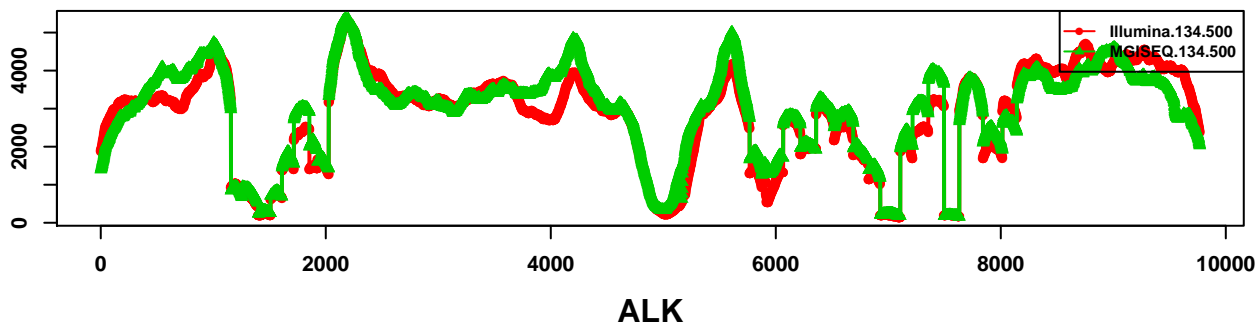

Supplement: Supplementary file 1 [file Presentation4.zip › ALK/19ZN13101T.pdf]

Sequencing Depth

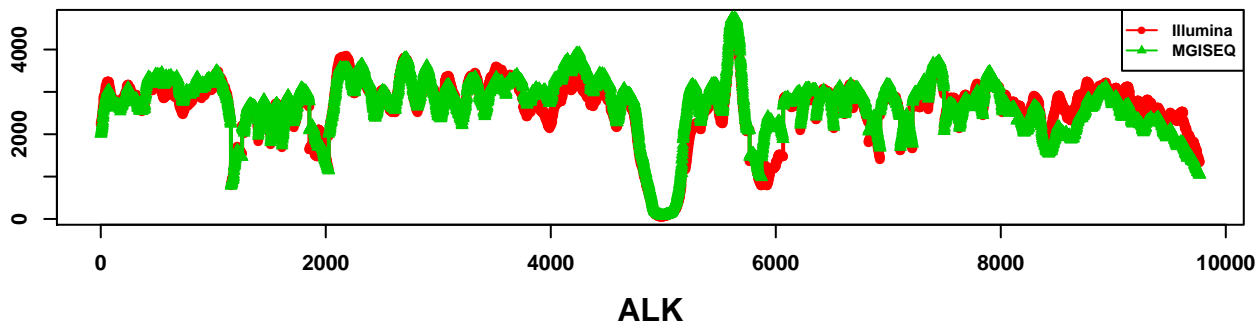

Sequencing Depth

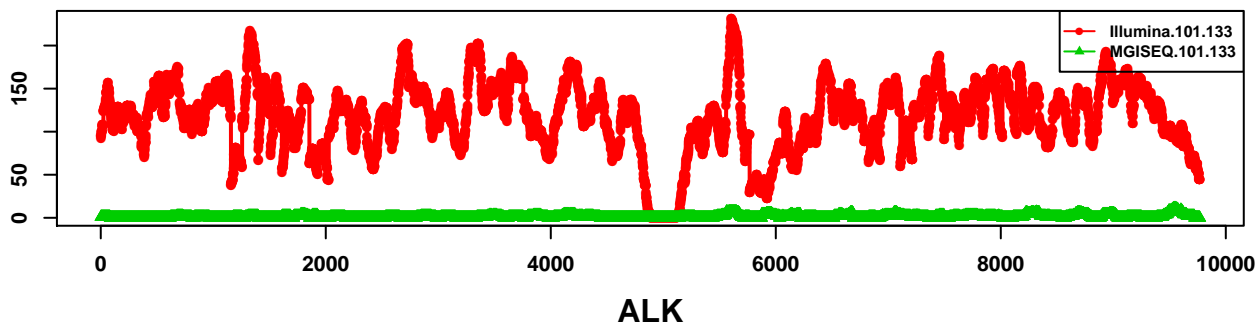

Sequencing Depth

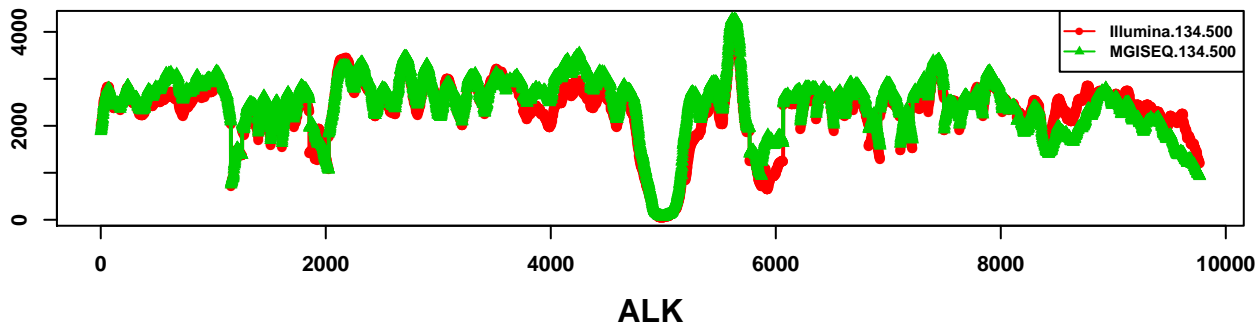

Supplement: Supplementary file 1 [file Presentation4.zip › ALK/19N01650P.pdf]

Sequencing Depth

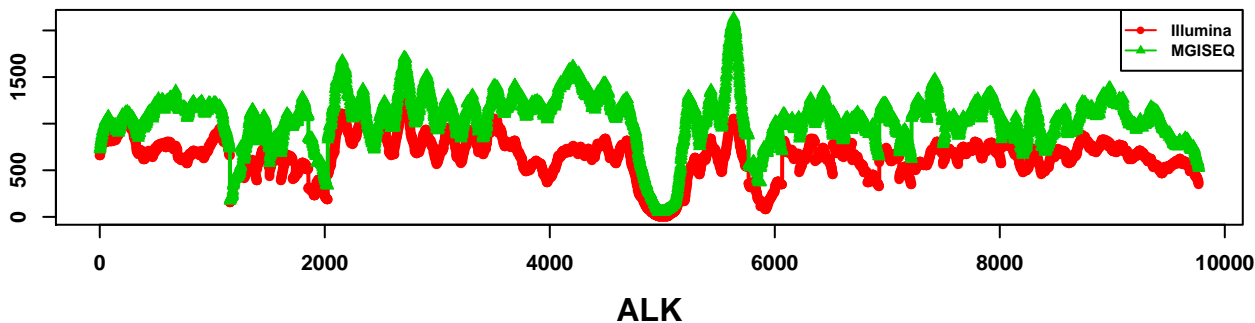

Sequencing Depth

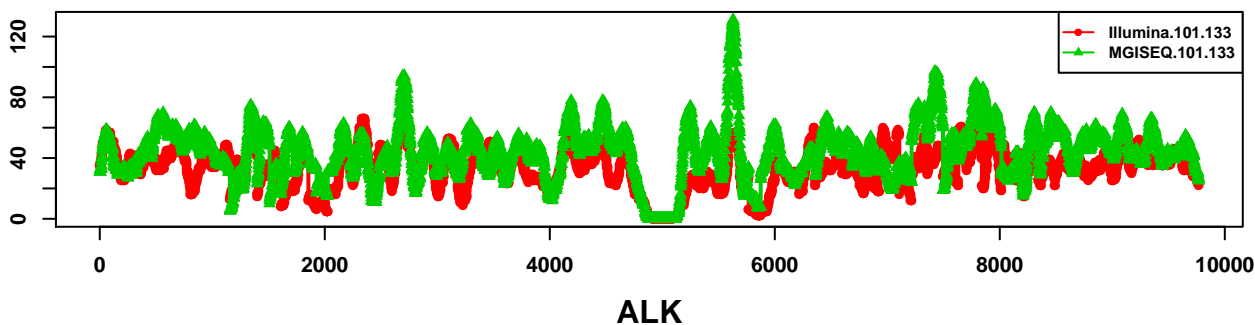

Sequencing Depth

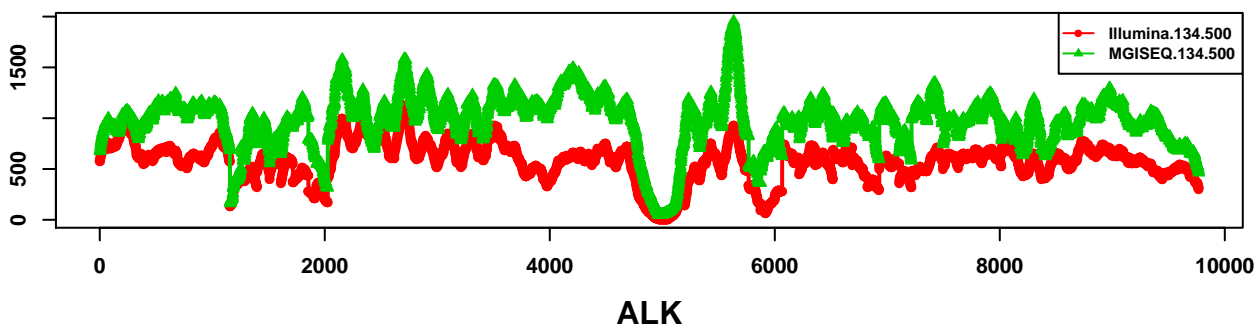

Supplement: Supplementary file 1 [file Presentation4.zip › ALK/19ZN12584P.pdf]

Sequencing Depth

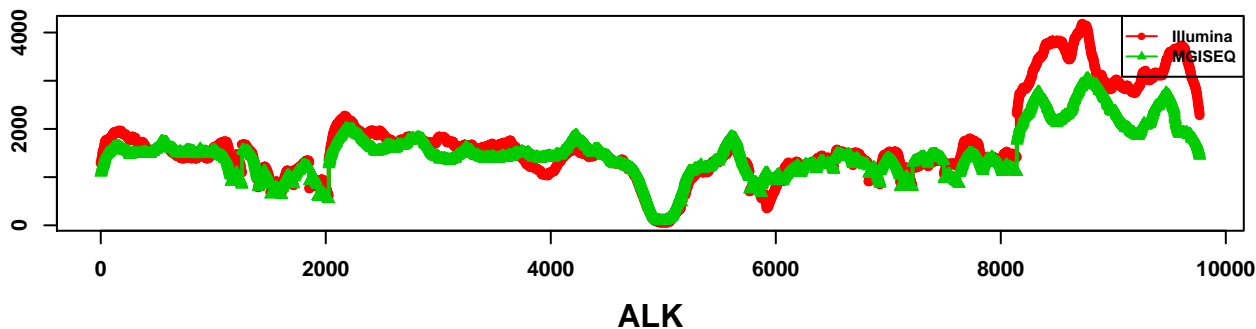

Sequencing Depth

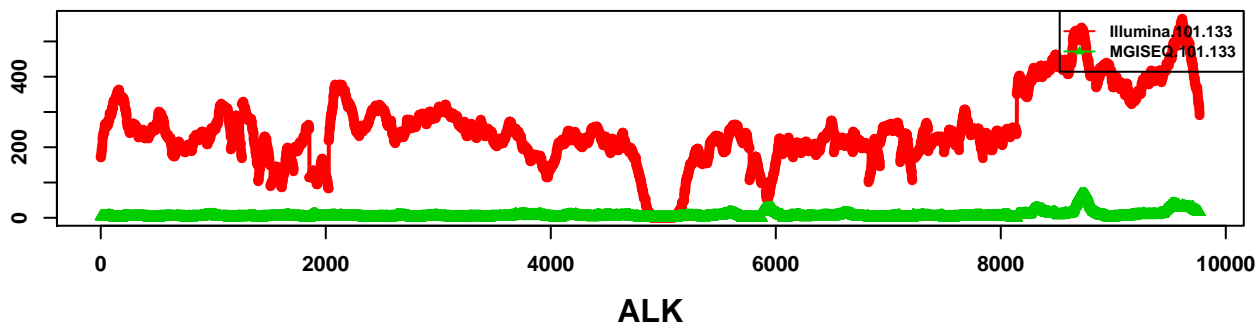

Sequencing Depth

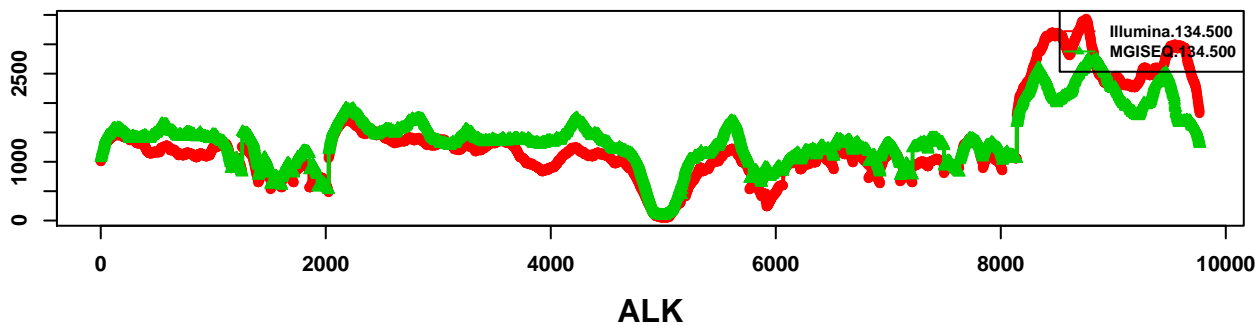

Supplement: Supplementary file 1 [file Presentation4.zip › ALK/19ZN11296F.pdf]

Sequencing Depth

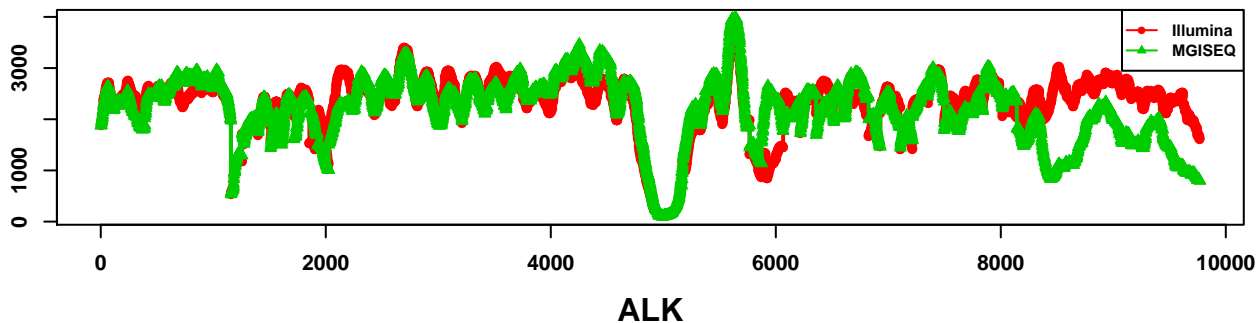

Sequencing Depth

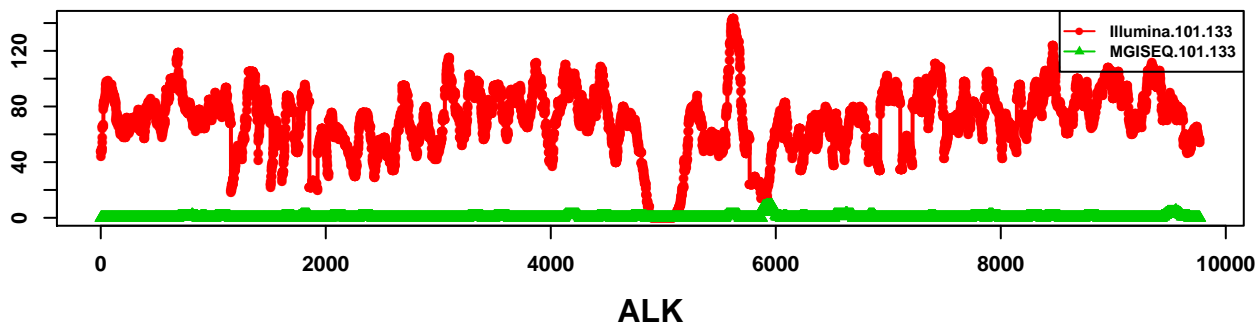

Sequencing Depth

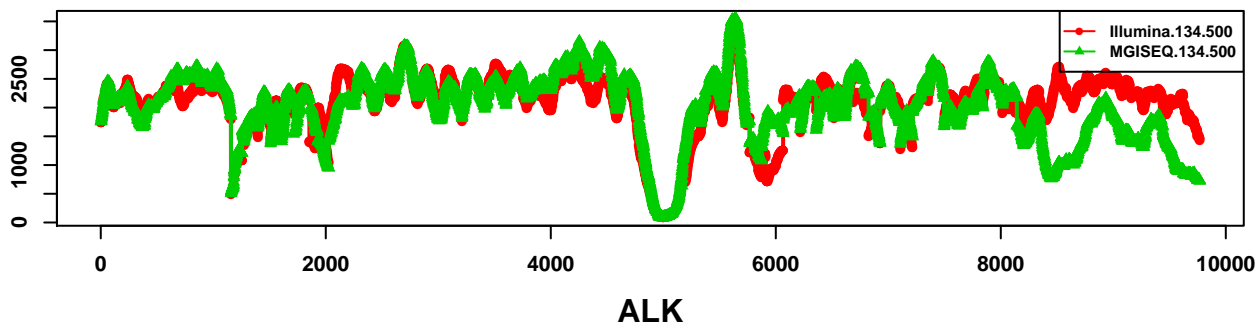

Supplement: Supplementary file 1 [file Presentation4.zip › ALK/19N01666P.pdf]

Sequencing Depth

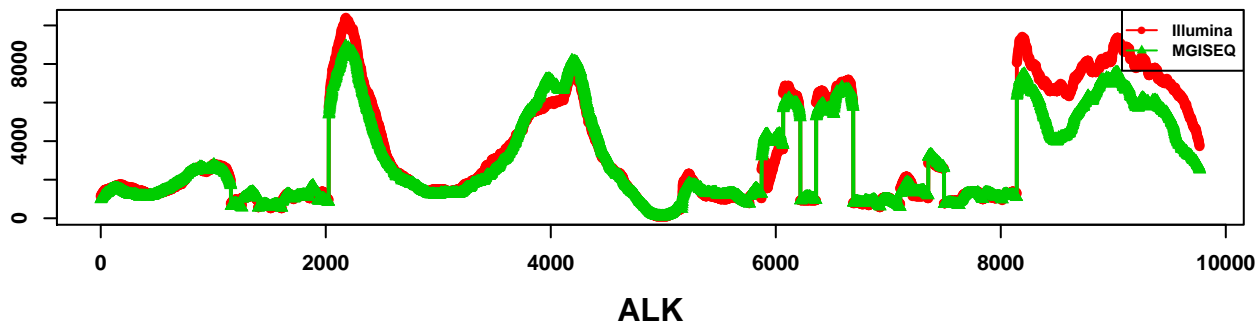

Sequencing Depth

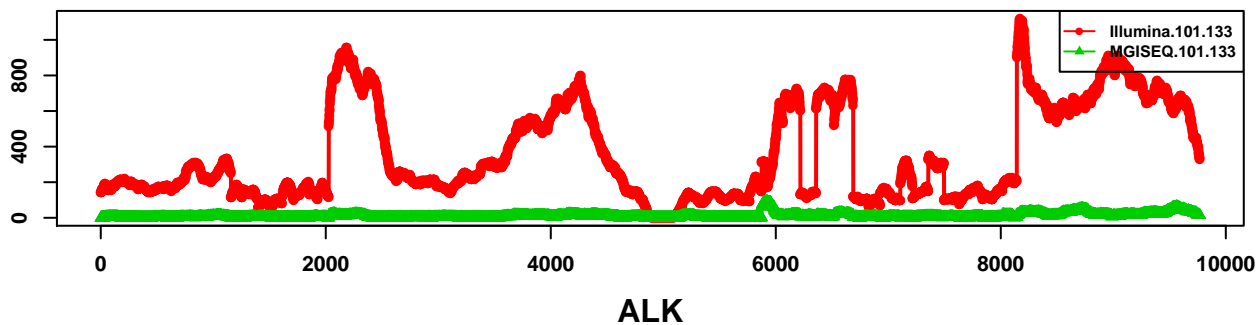

Sequencing Depth

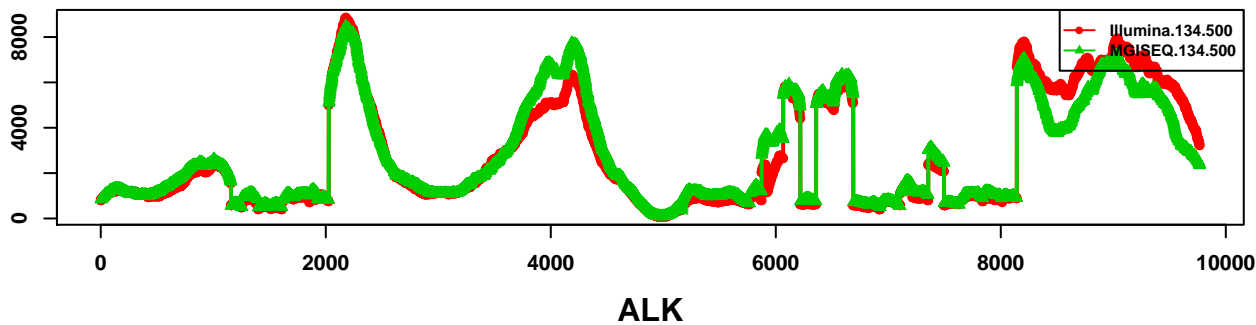

Supplement: Supplementary file 1 [file Presentation4.zip › ALK/19FC40246F.pdf]

Sequencing Depth

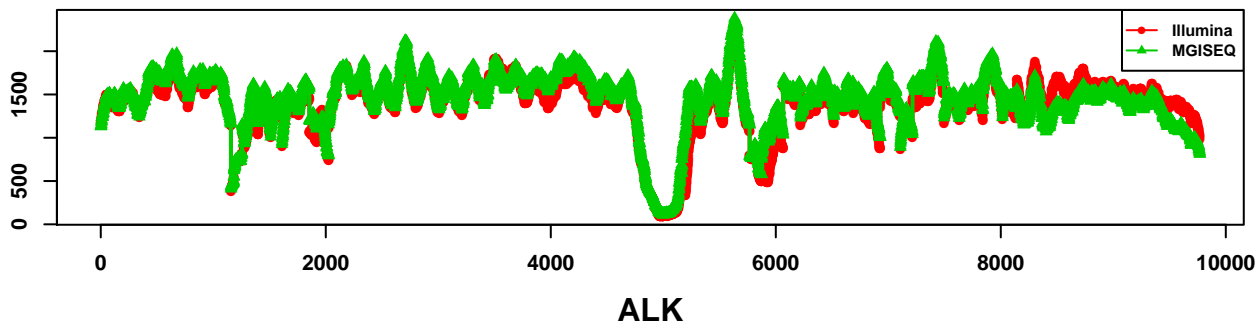

Sequencing Depth

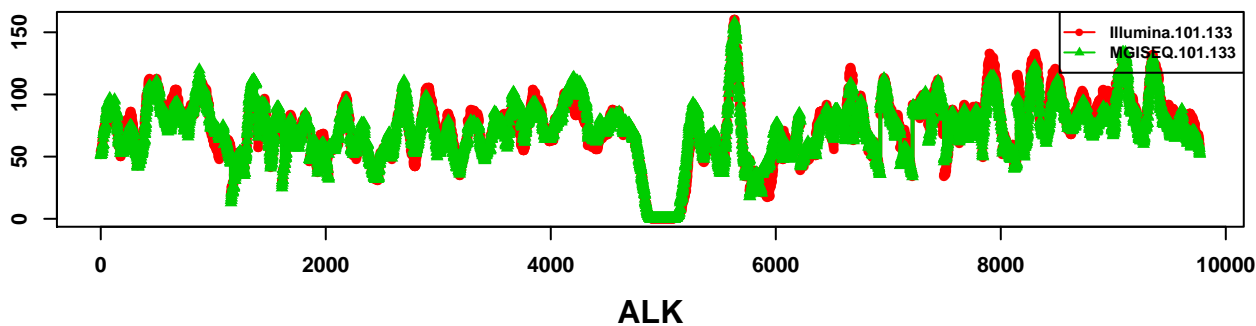

Sequencing Depth

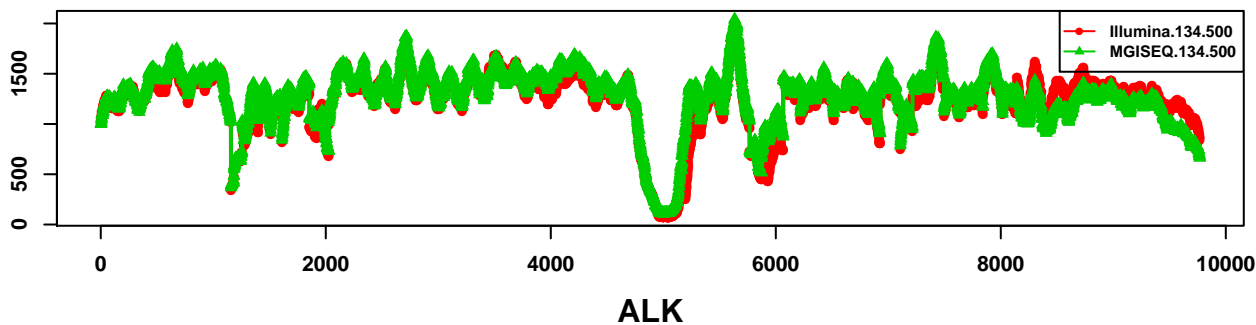

Supplement: Supplementary file 1 [file Presentation4.zip › ALK/19CF15711P.pdf]

Sequencing Depth

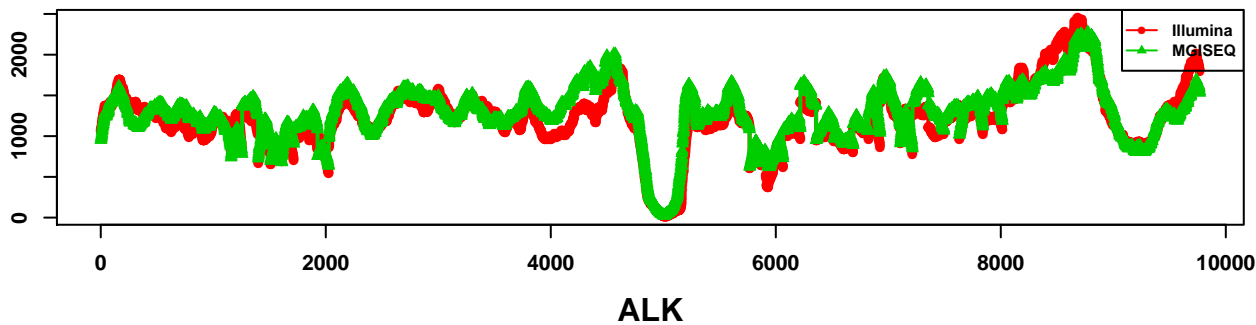

Sequencing Depth

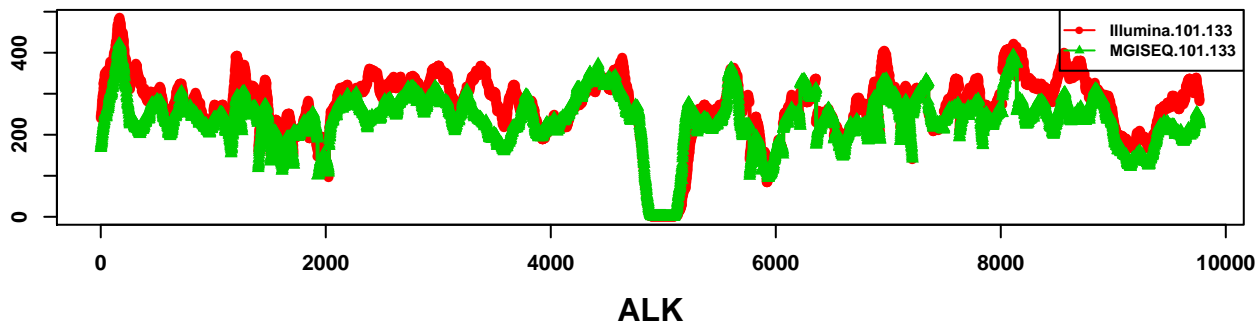

Sequencing Depth

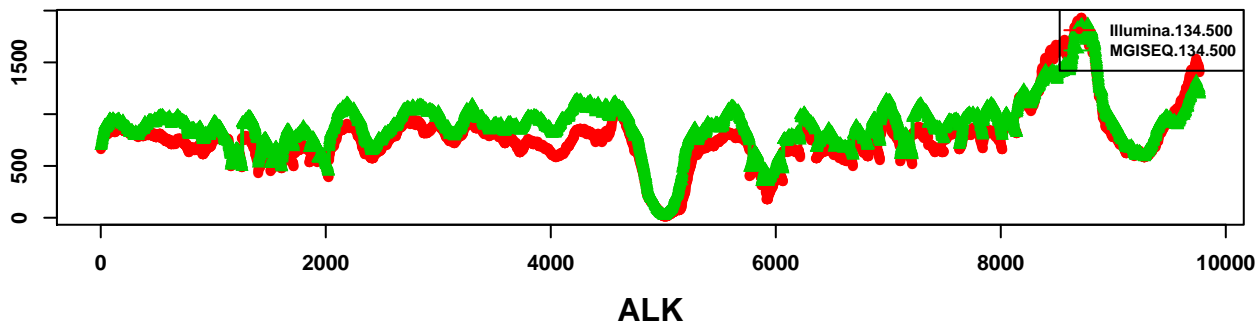

Supplement: Supplementary file 1 [file Presentation4.zip › ALK/19Q06297F.pdf]

Sequencing Depth

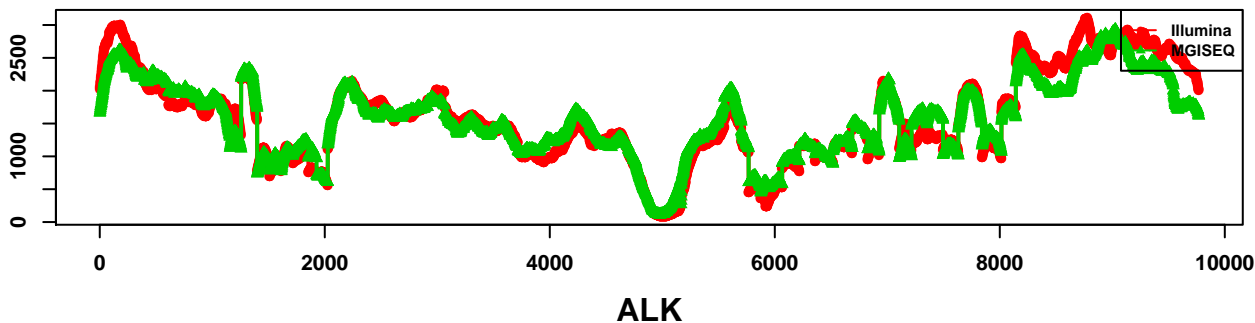

Sequencing Depth

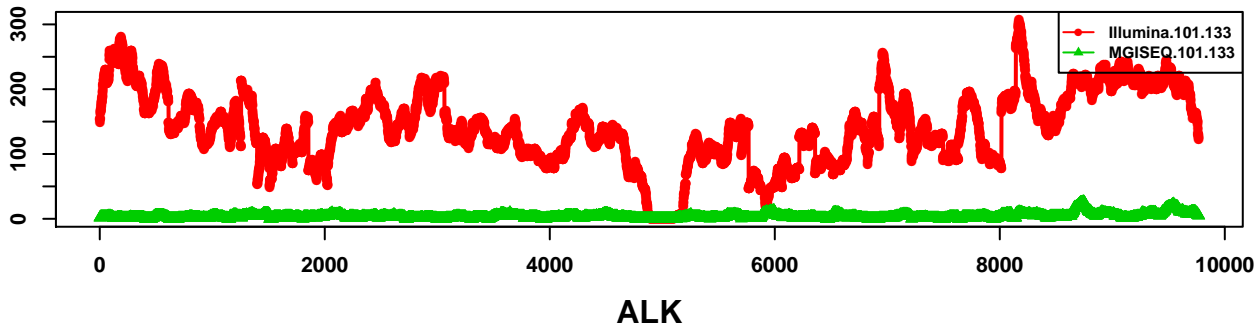

Sequencing Depth

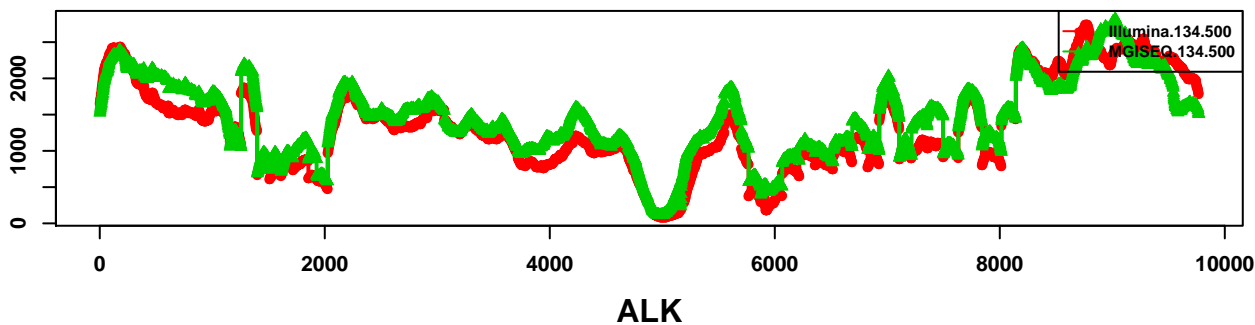

Supplement: Supplementary file 1 [file Presentation4.zip › ALK/19HE22008F.pdf]

Sequencing Depth

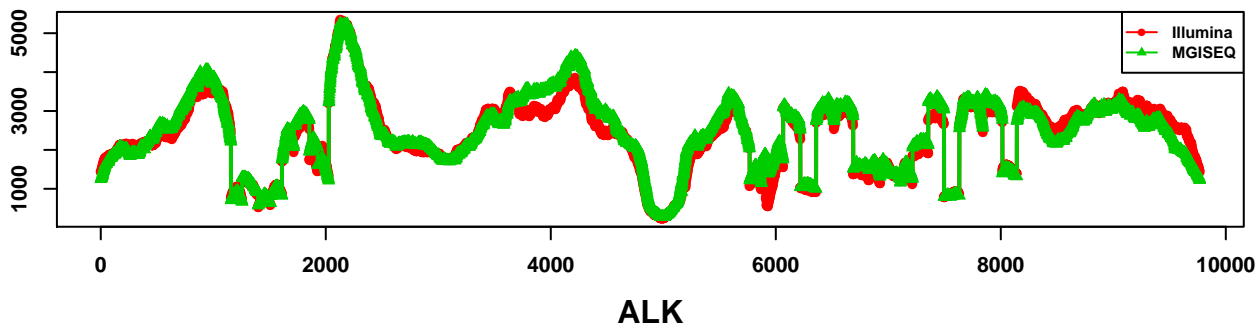

Sequencing Depth

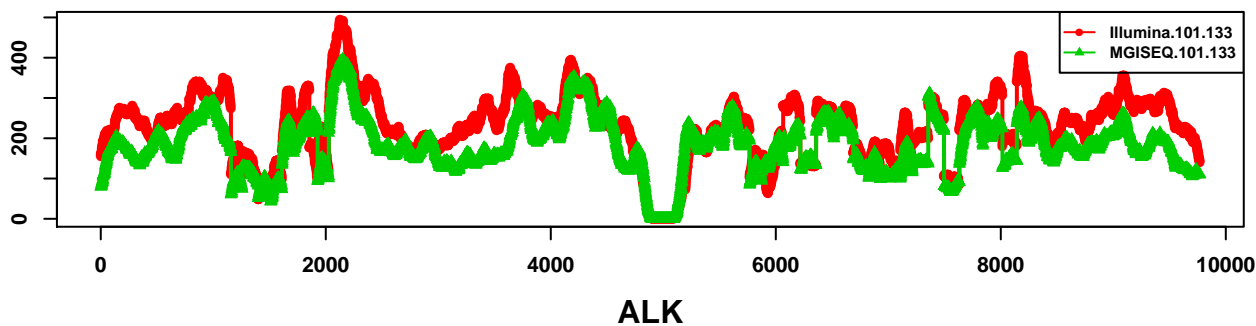

Sequencing Depth

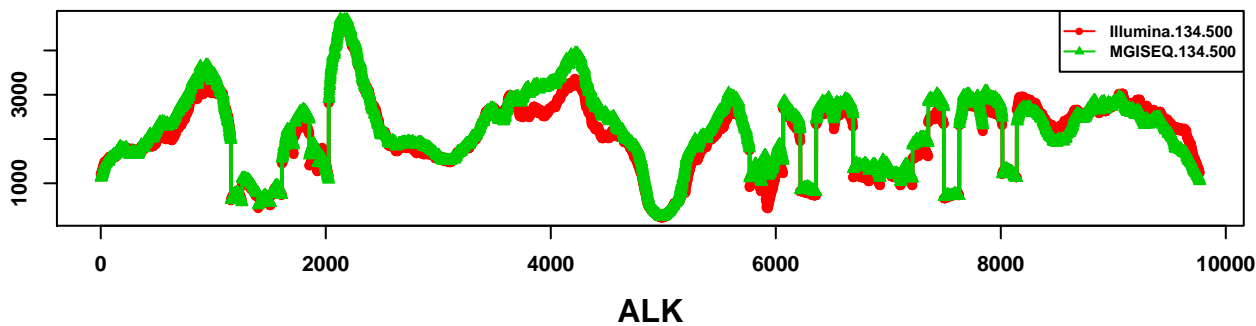

Supplement: Supplementary file 1 [file Presentation4.zip › ALK/19Q06147F.pdf]

Sequencing Depth

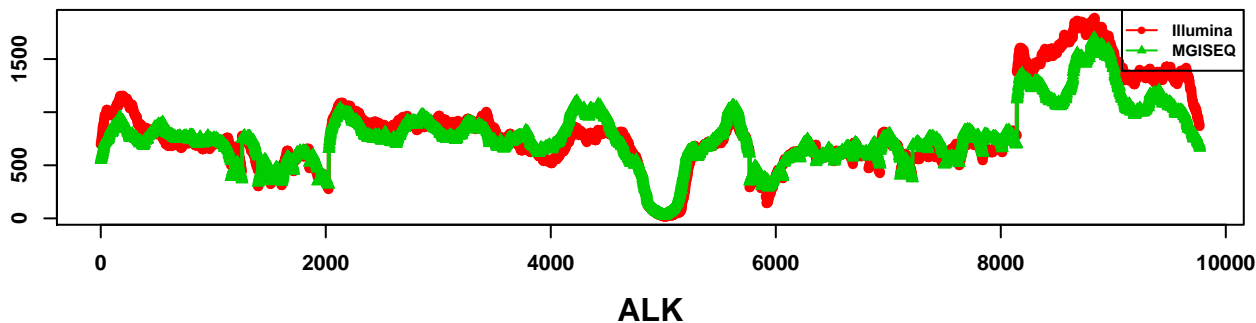

Sequencing Depth

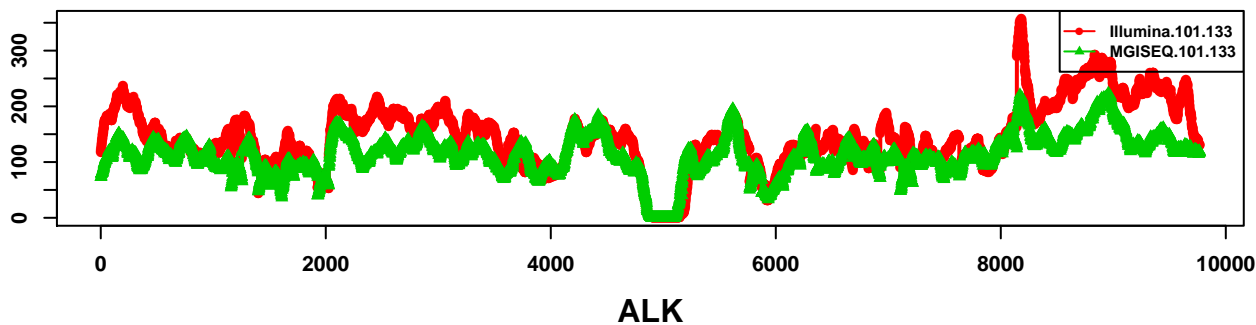

Sequencing Depth

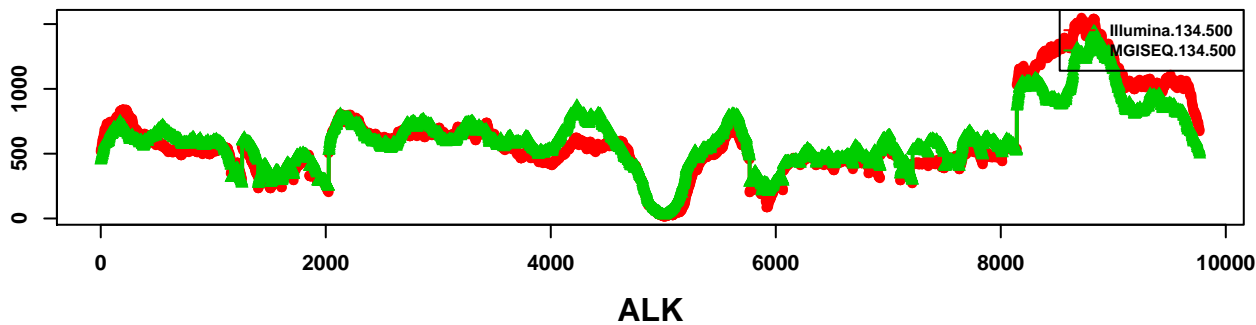

Supplement: Supplementary file 1 [file Presentation4.zip › ALK/19ZN13675F.pdf]

Sequencing Depth

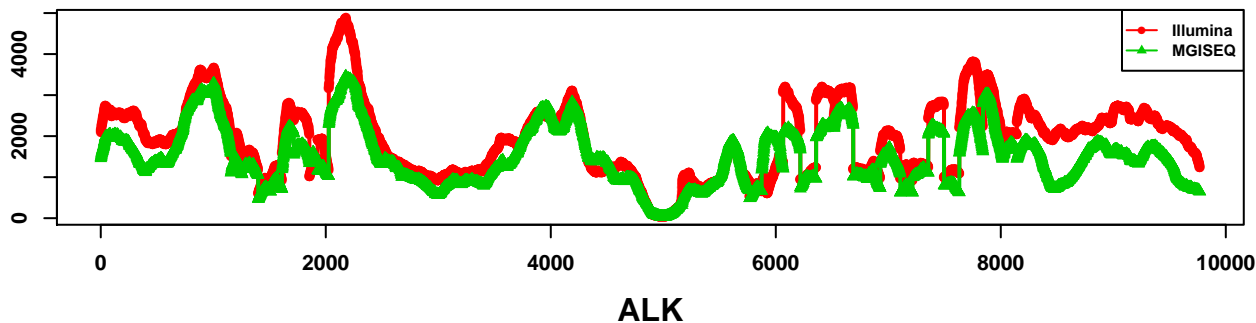

Sequencing Depth

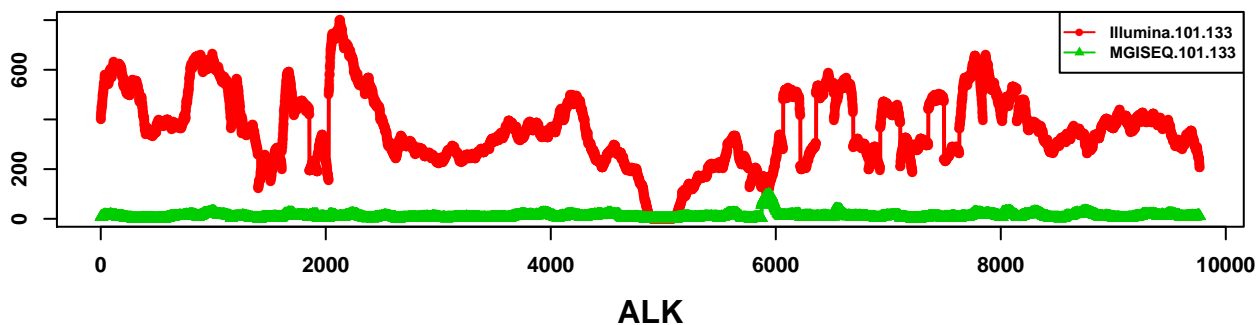

Sequencing Depth

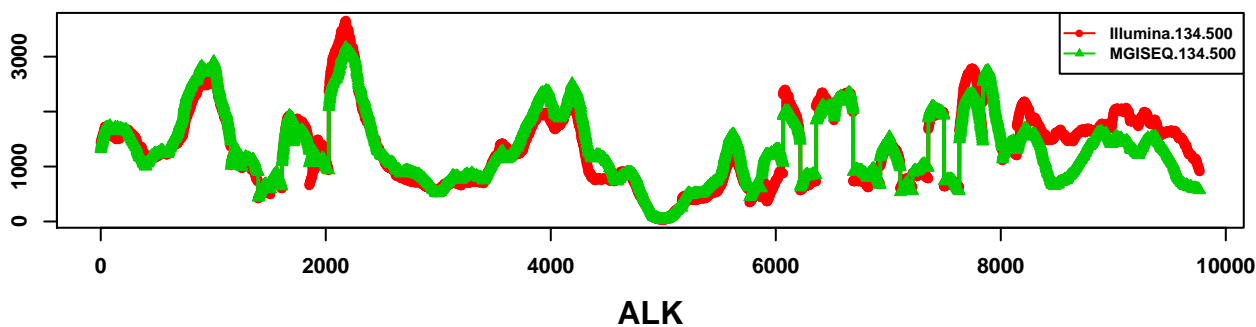

Supplement: Supplementary file 1 [file Presentation4.zip › ALK/19N01671T.pdf]

Sequencing Depth

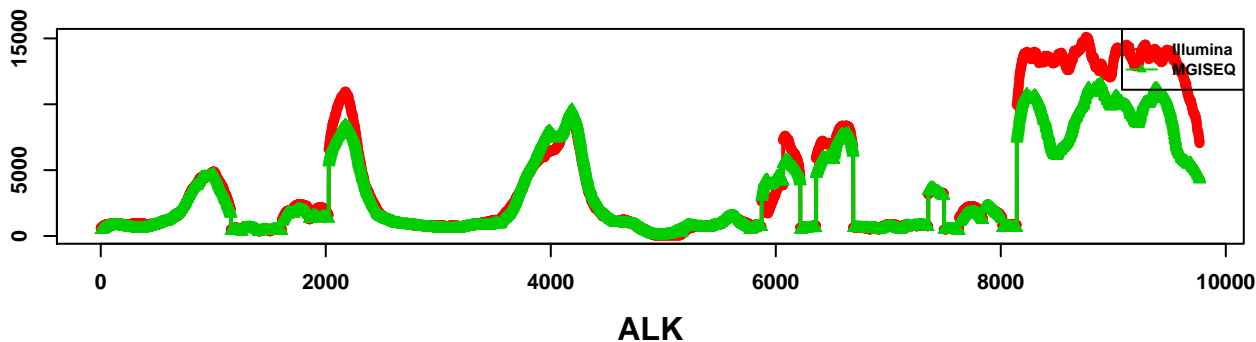

Sequencing Depth

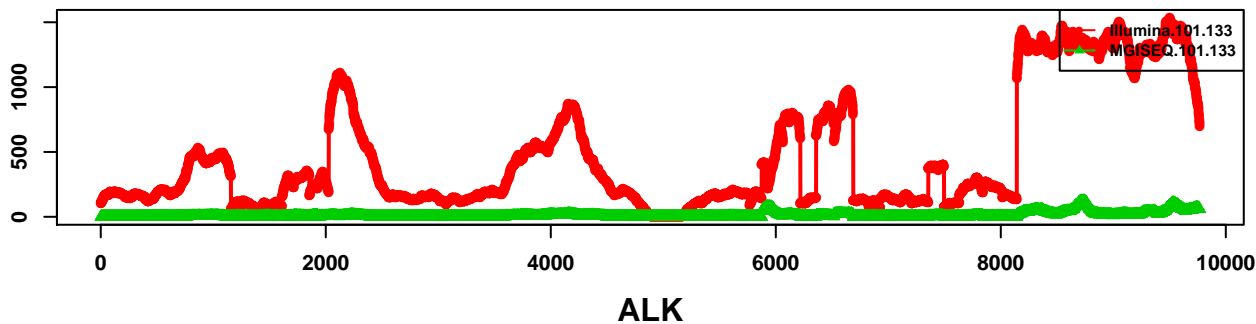

Sequencing Depth

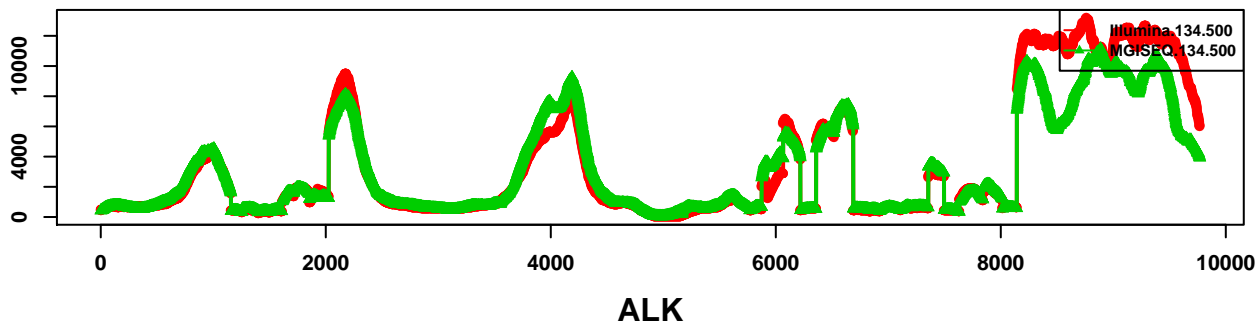

Supplement: Supplementary file 1 [file Presentation4.zip › ALK/19N01467QC.pdf]

Sequencing Depth

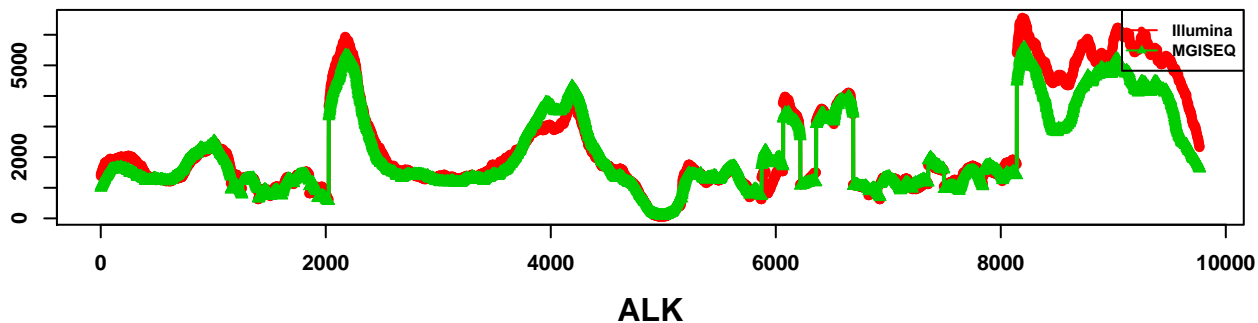

Sequencing Depth

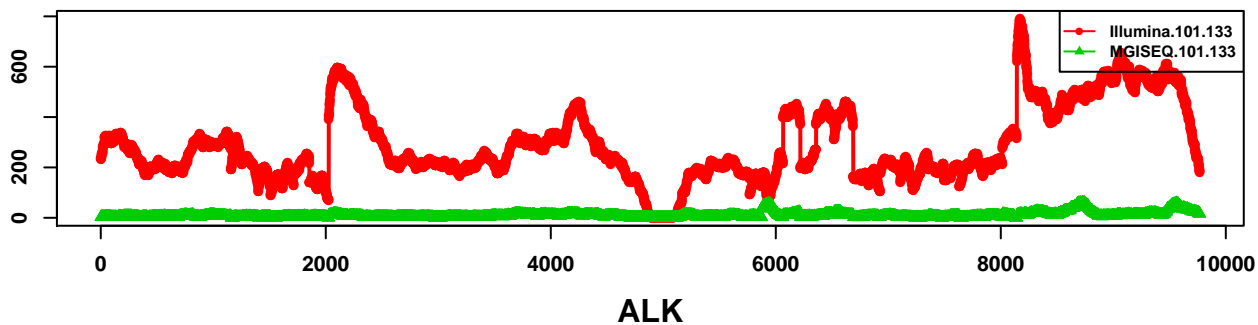

Sequencing Depth

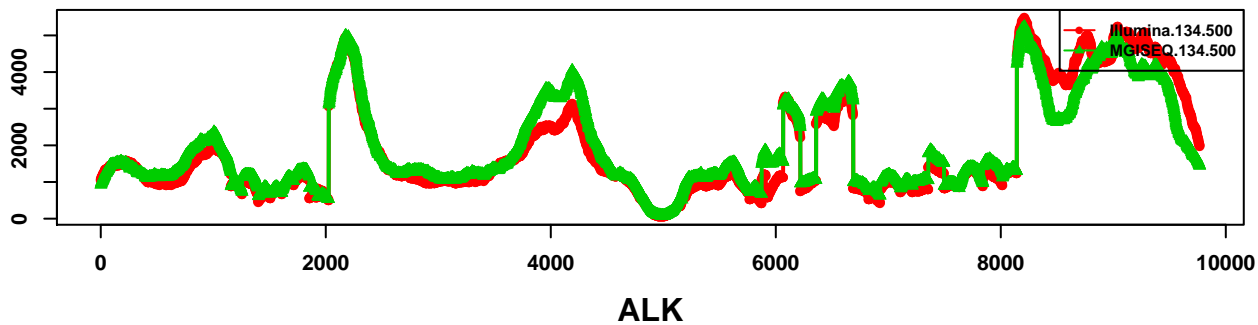

Supplement: Supplementary file 1 [file Presentation4.zip › ALK/19FC40250F.pdf]

Sequencing Depth

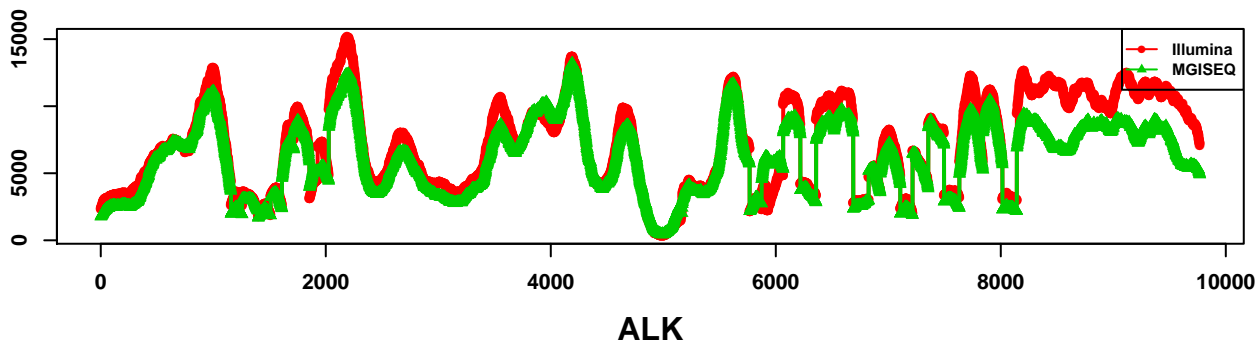

Sequencing Depth

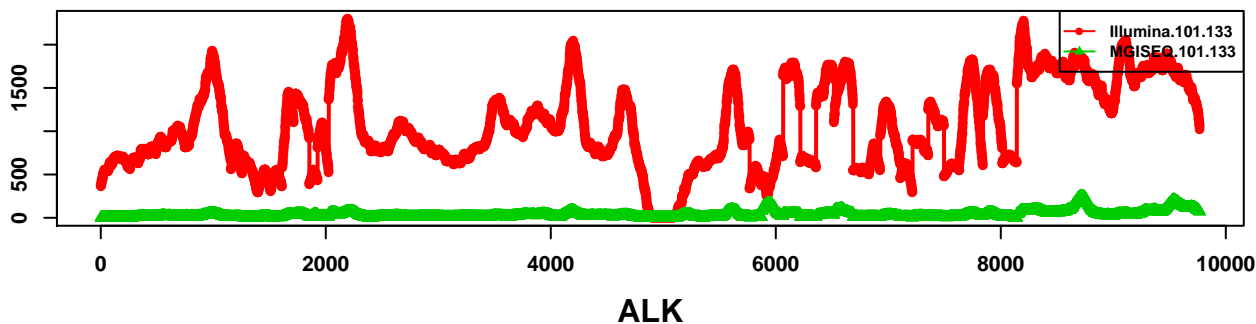

Sequencing Depth

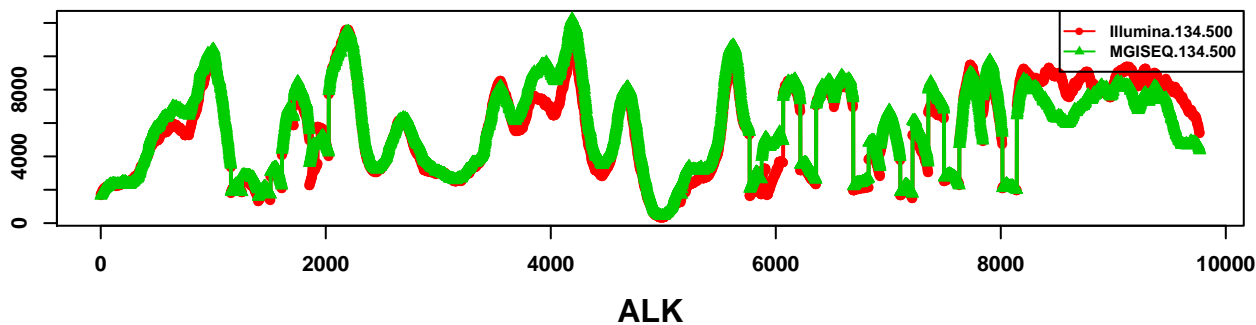

Supplement: Supplementary file 1 [file Presentation4.zip › ALK/19HE22120F.pdf]

Sequencing Depth

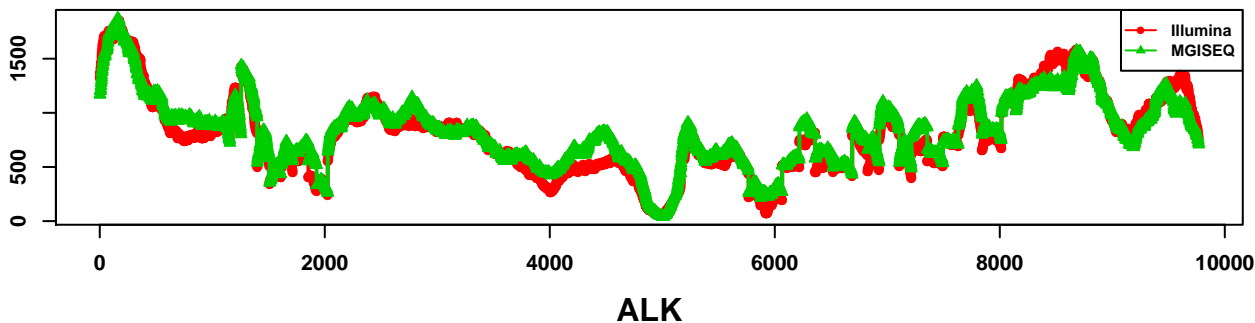

Sequencing Depth

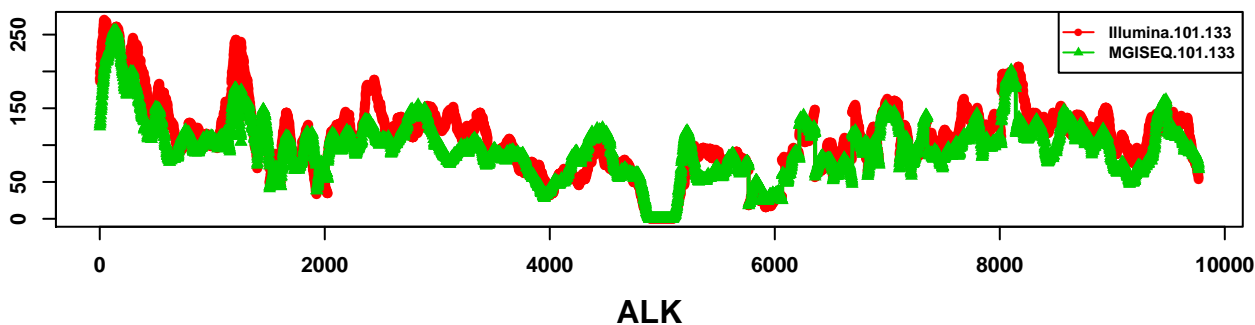

Sequencing Depth

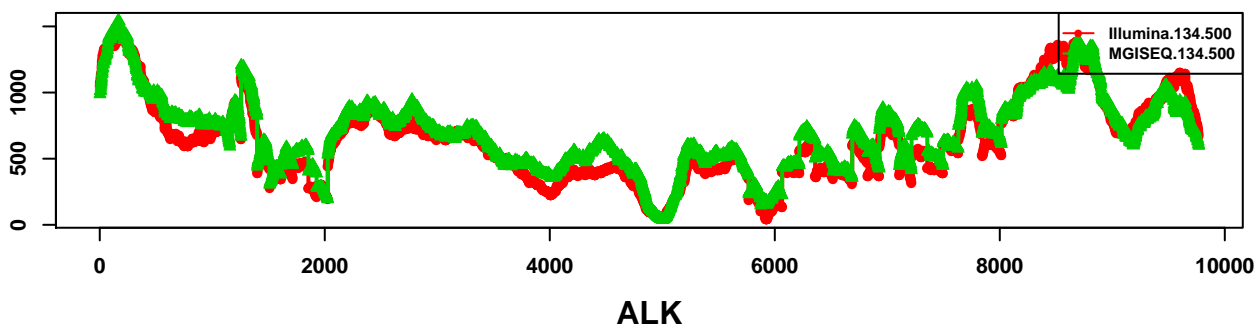

Supplement: Supplementary file 1 [file Presentation4.zip › ALK/19N01373F.pdf]

Sequencing Depth

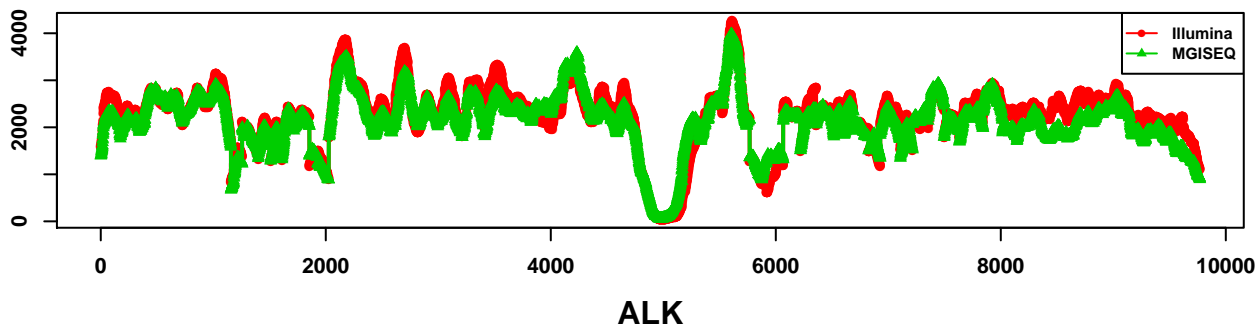

Sequencing Depth

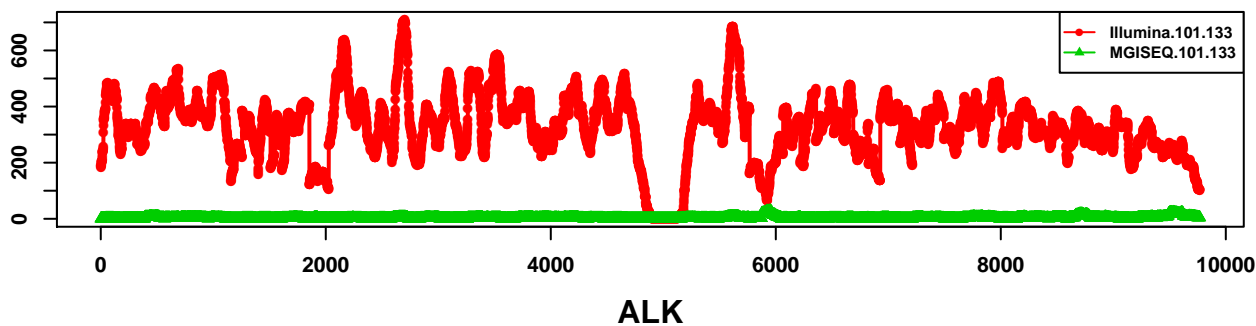

Sequencing Depth

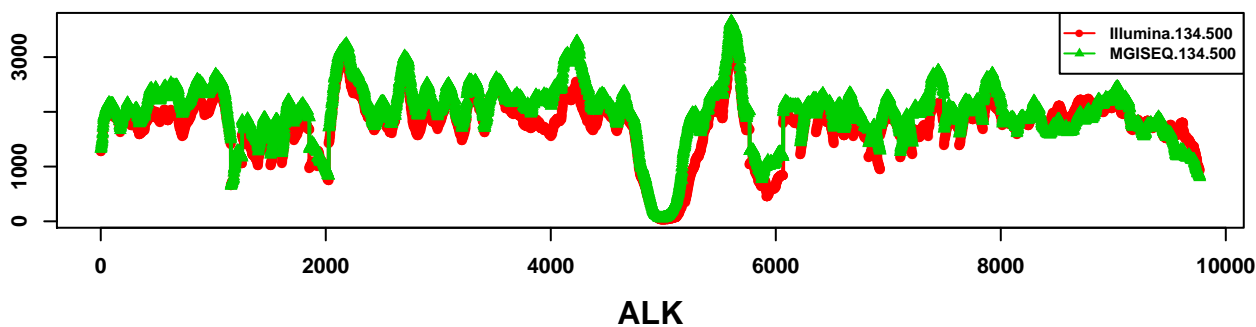

Supplement: Supplementary file 1 [file Presentation4.zip › ALK/19HE22225P.pdf]

Sequencing Depth

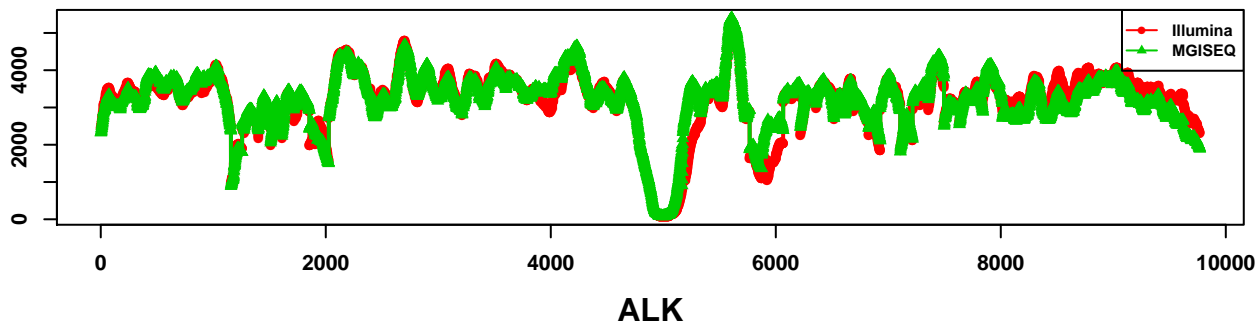

Sequencing Depth

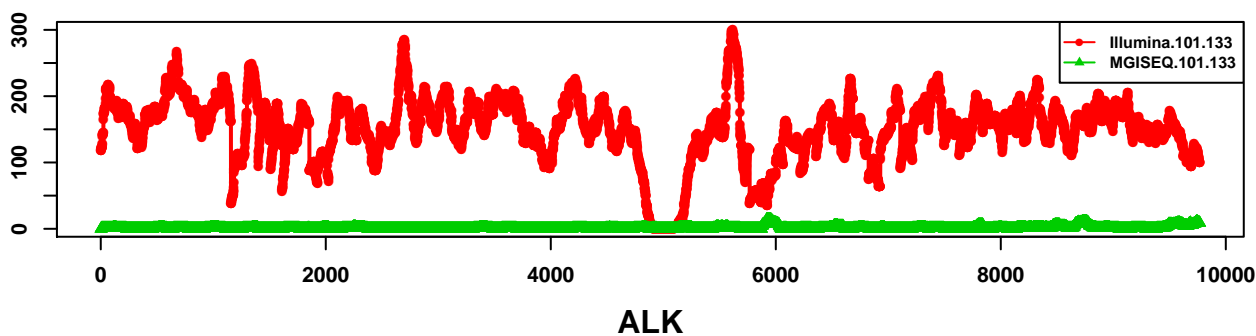

Sequencing Depth

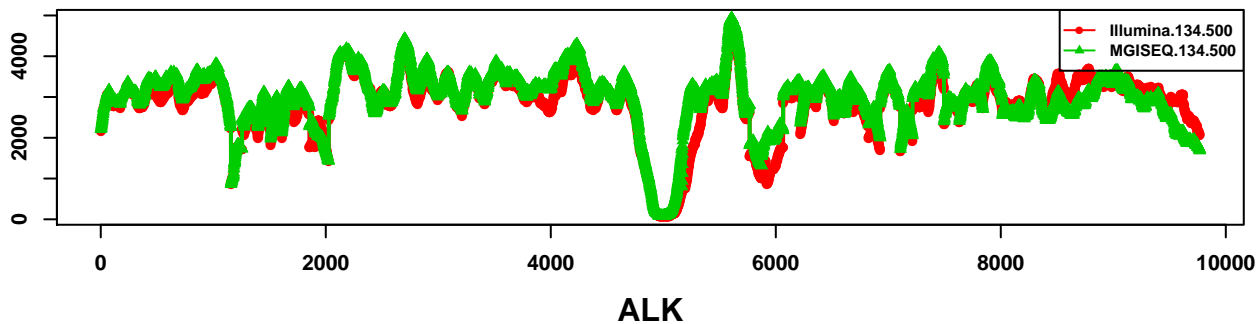

Supplement: Supplementary file 1 [file Presentation4.zip › ALK/19YT52688P.pdf]

Sequencing Depth

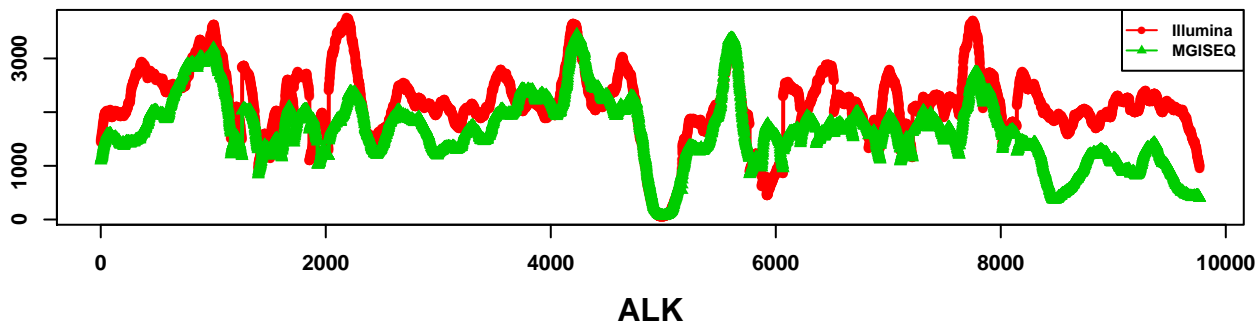

Sequencing Depth

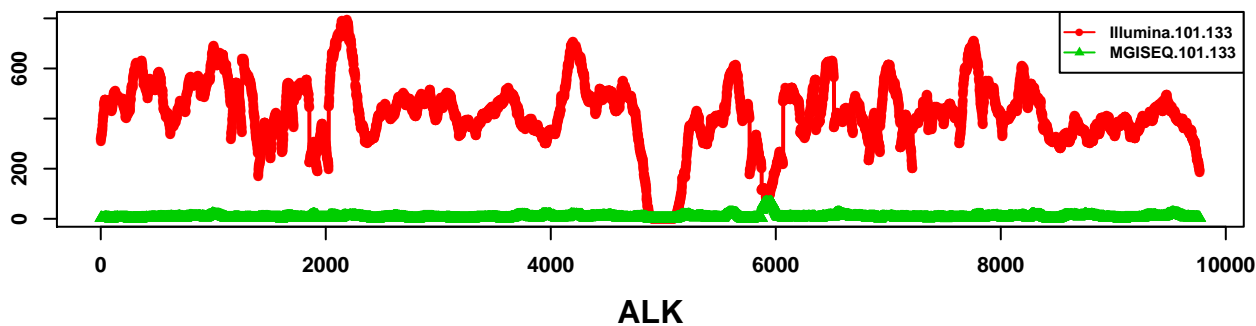

Sequencing Depth

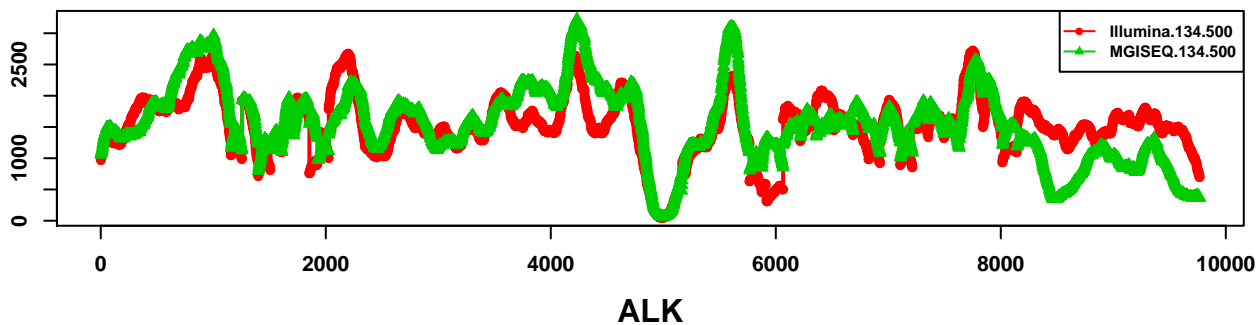

Supplement: Supplementary file 1 [file Presentation4.zip › ALK/19N01667F.pdf]

Sequencing Depth

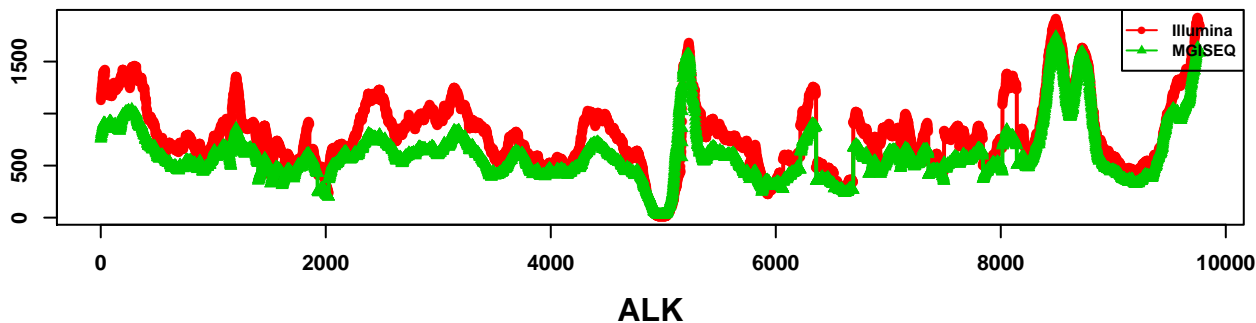

Sequencing Depth

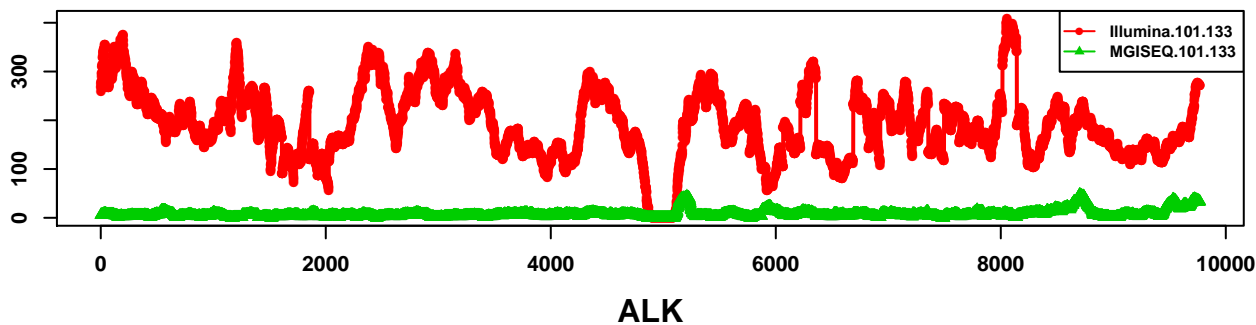

Sequencing Depth

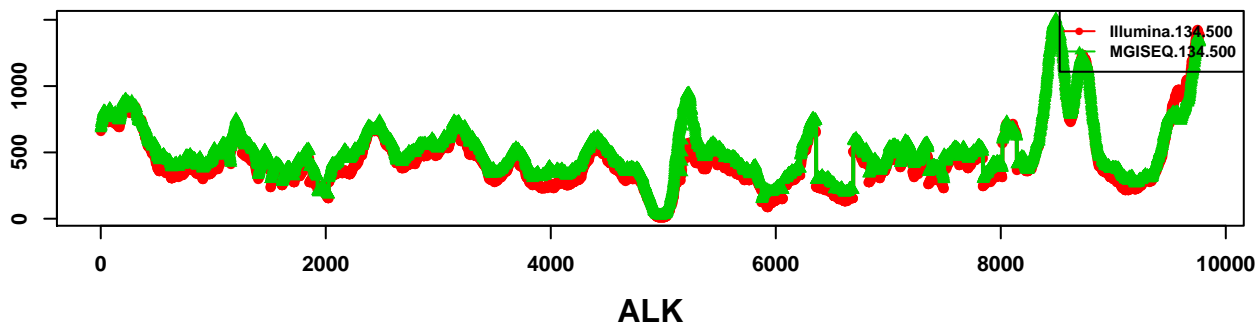

Supplement: Supplementary file 2 [file Presentation1.zip › ALK/19N01687F.pdf]

Sequencing Depth

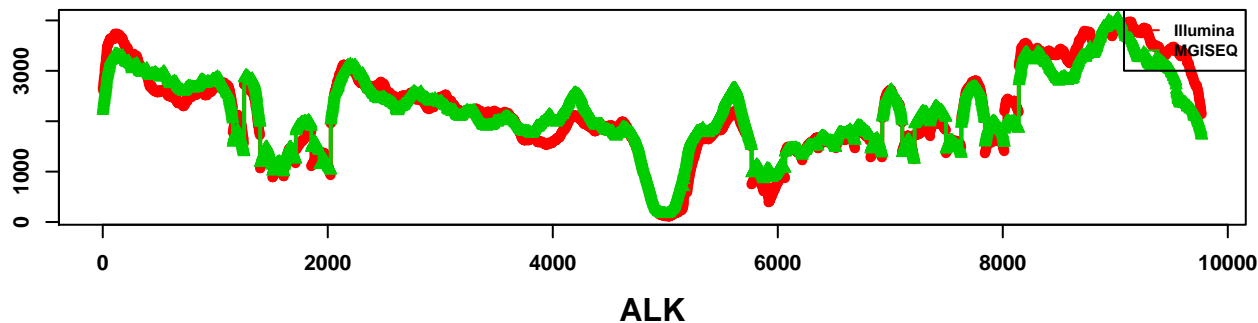

Sequencing Depth

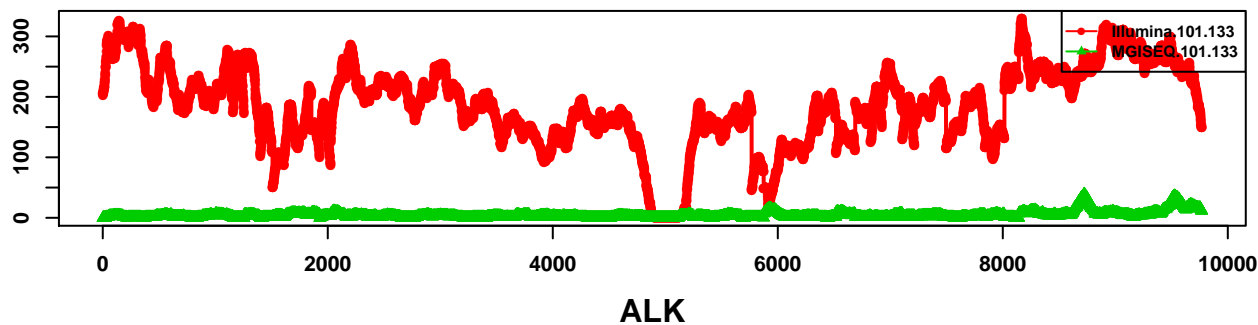

Sequencing Depth

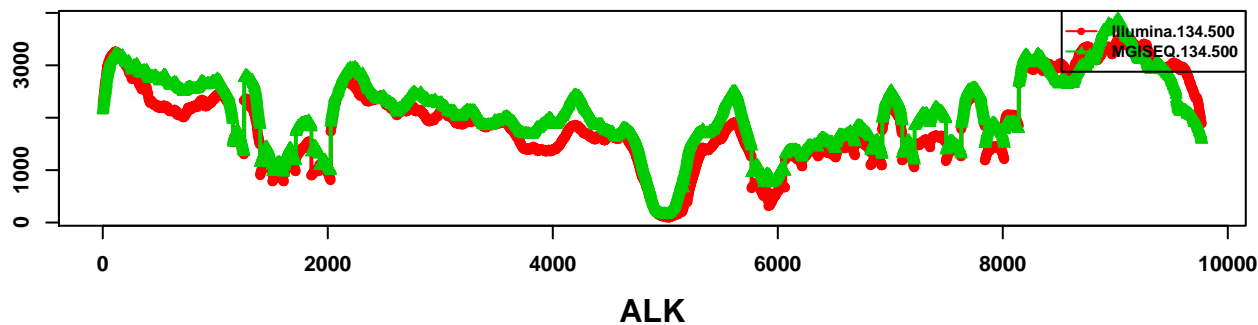

Supplement: Supplementary file 2 [file Presentation1.zip › ALK/19HE22084F.pdf]

Sequencing Depth

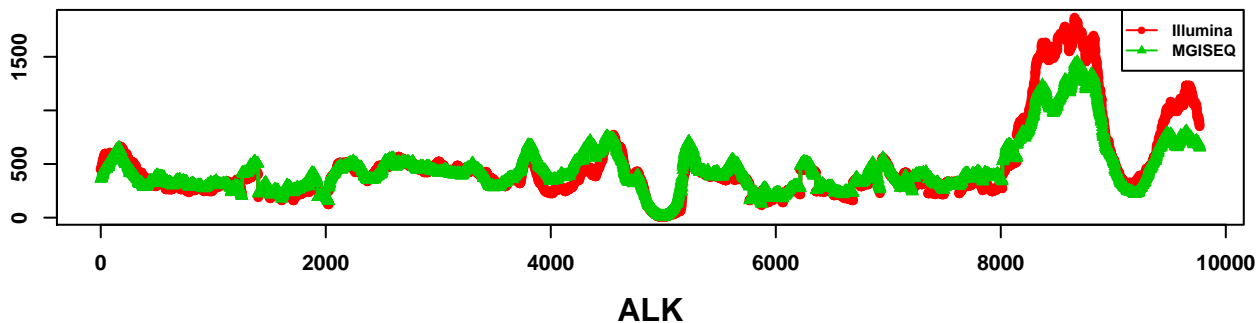

Sequencing Depth

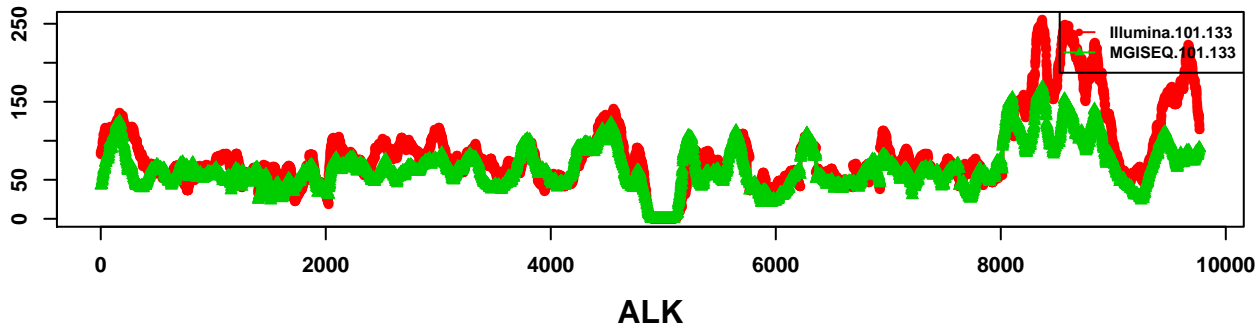

Sequencing Depth

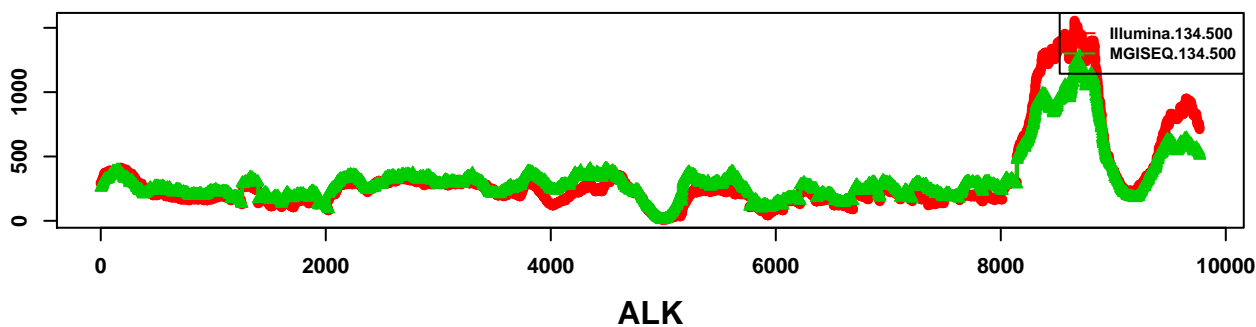

Supplement: Supplementary file 2 [file Presentation1.zip › ALK/19ZN13587F.pdf]

Sequencing Depth

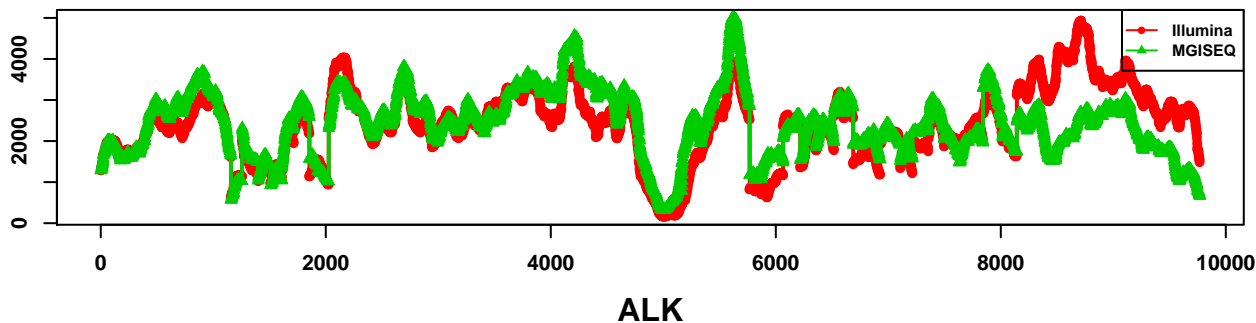

Sequencing Depth

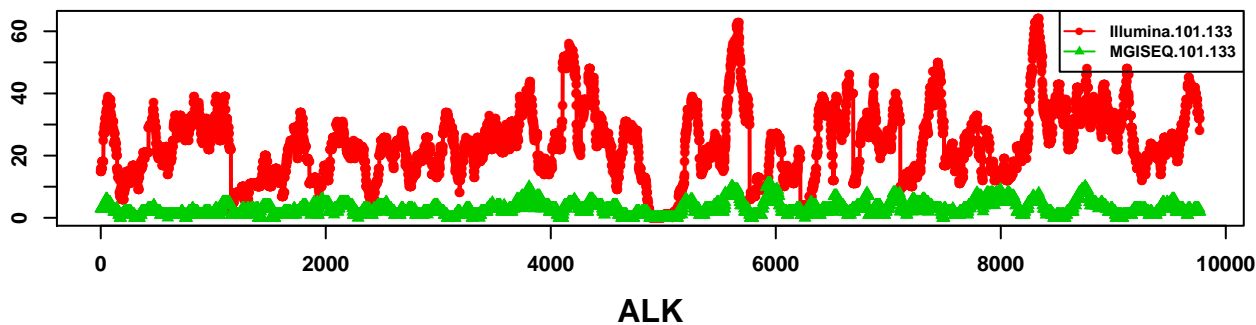

Sequencing Depth

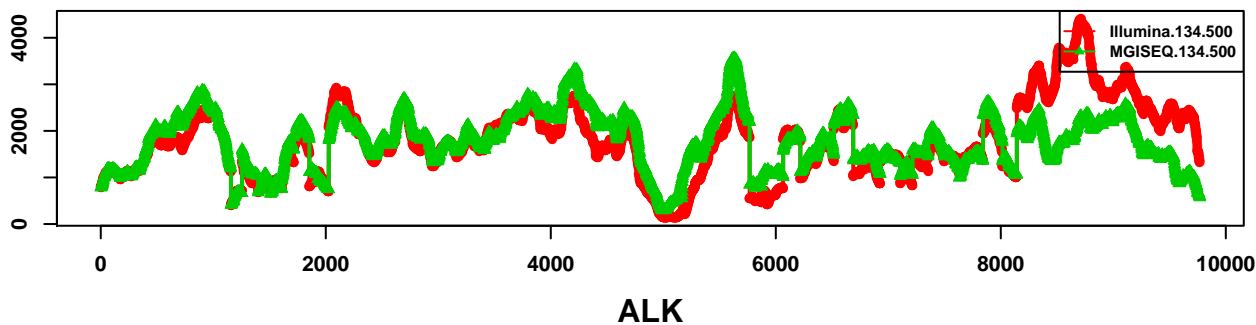

Supplement: Supplementary file 2 [file Presentation1.zip › ALK/19KS38324-IIP.pdf]

Sequencing Depth

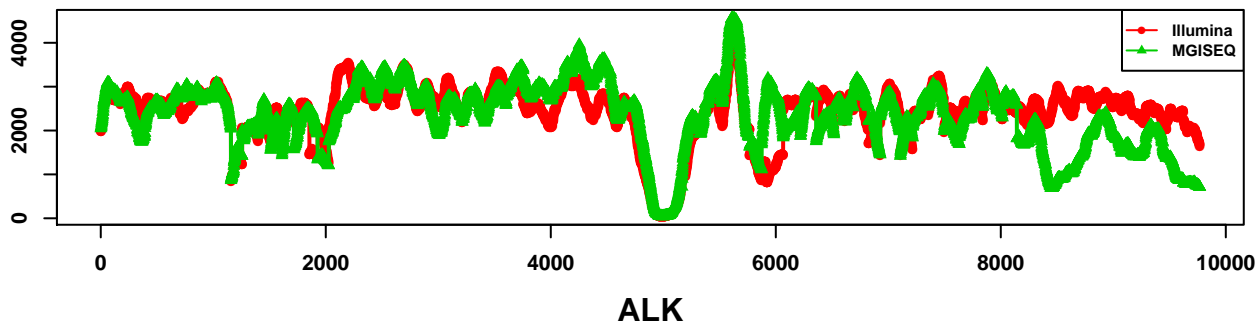

Sequencing Depth

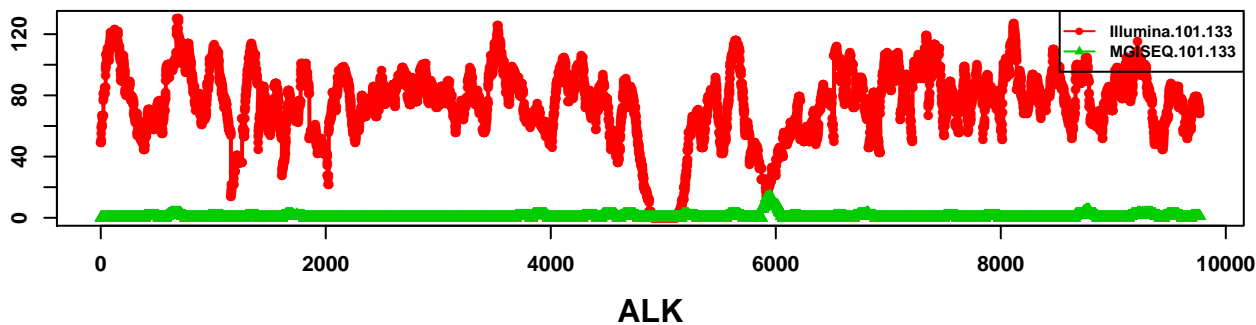

Sequencing Depth

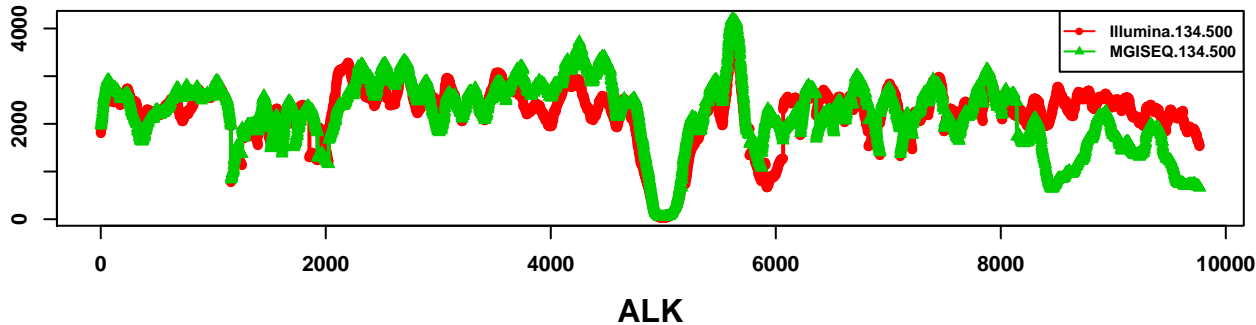

Supplement: Supplementary file 2 [file Presentation1.zip › ALK/19LN70358P.pdf]

Sequencing Depth

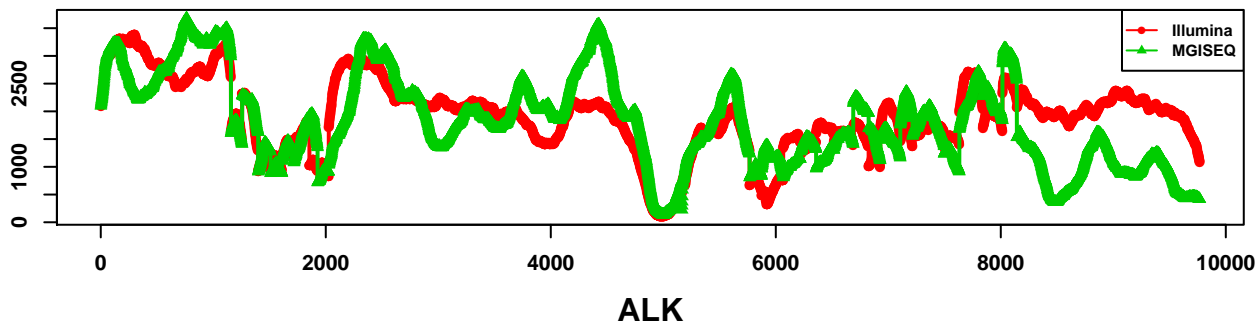

Sequencing Depth

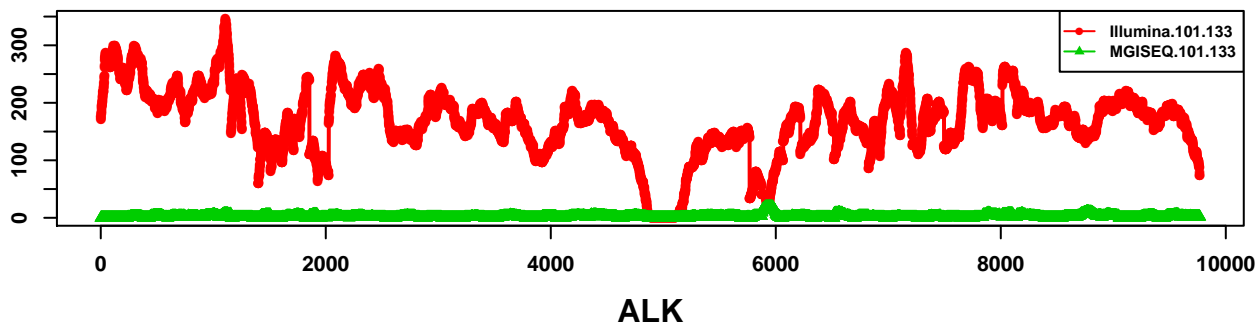

Sequencing Depth

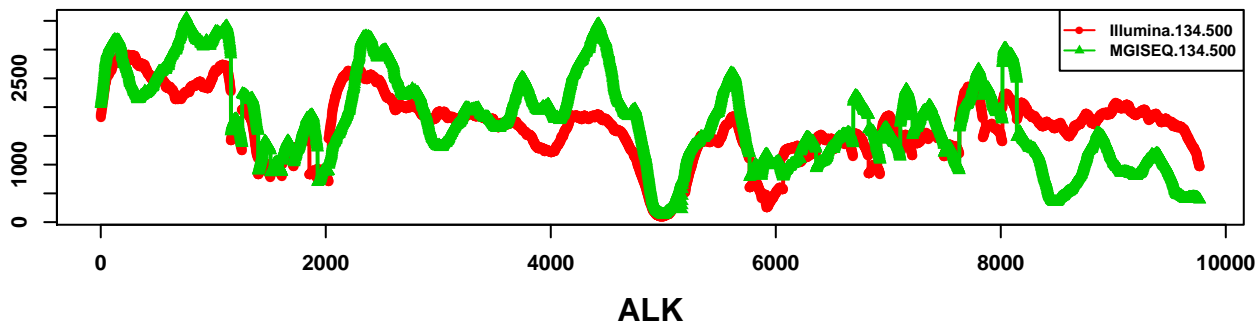

Supplement: Supplementary file 2 [file Presentation1.zip › ALK/19HE22051F.pdf]

Sequencing Depth

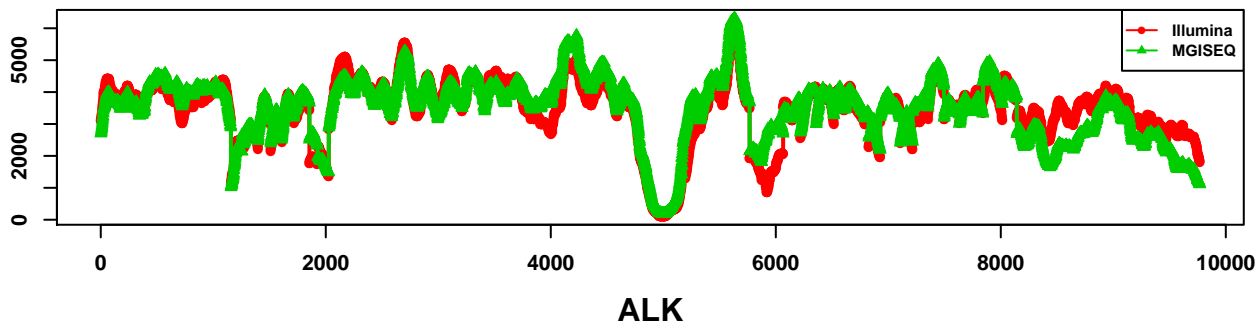

Sequencing Depth

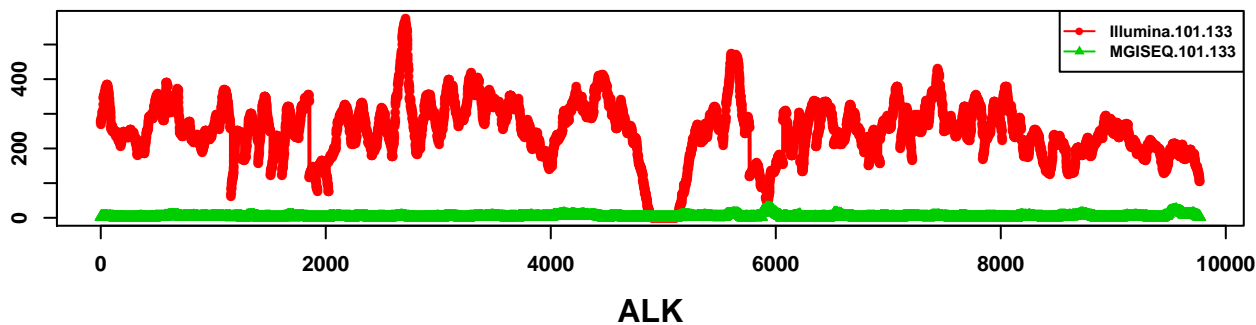

Sequencing Depth

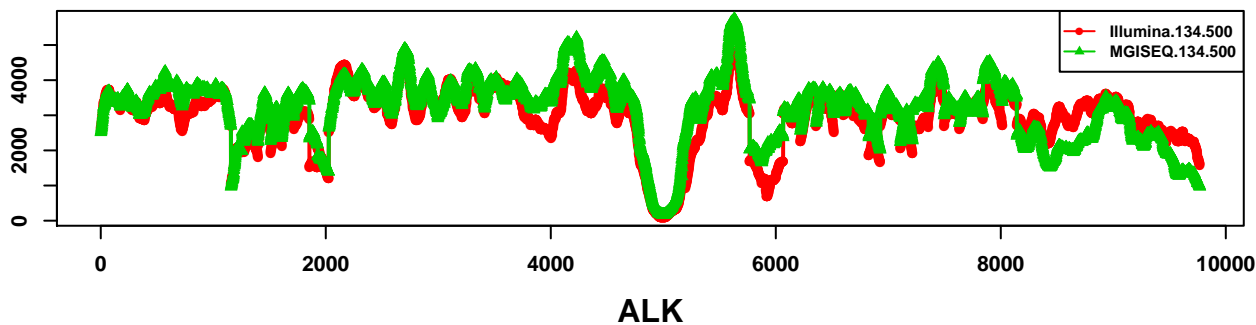

Supplement: Supplementary file 2 [file Presentation1.zip › ALK/19N01384P.pdf]

Sequencing Depth

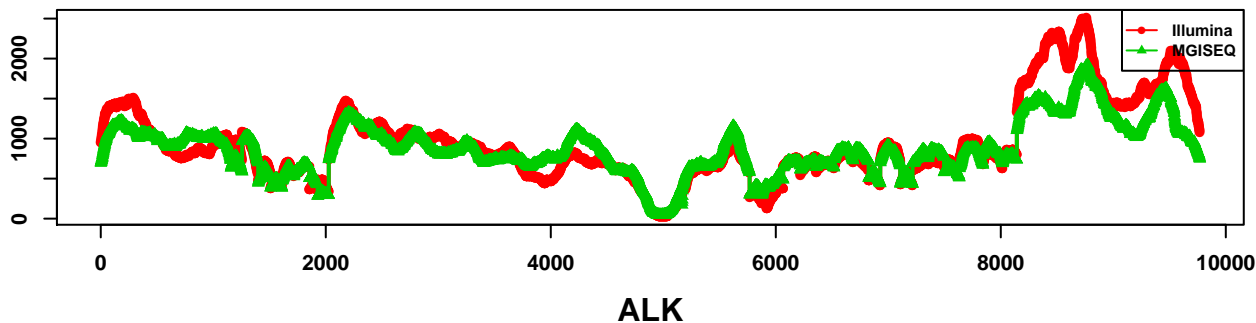

Sequencing Depth

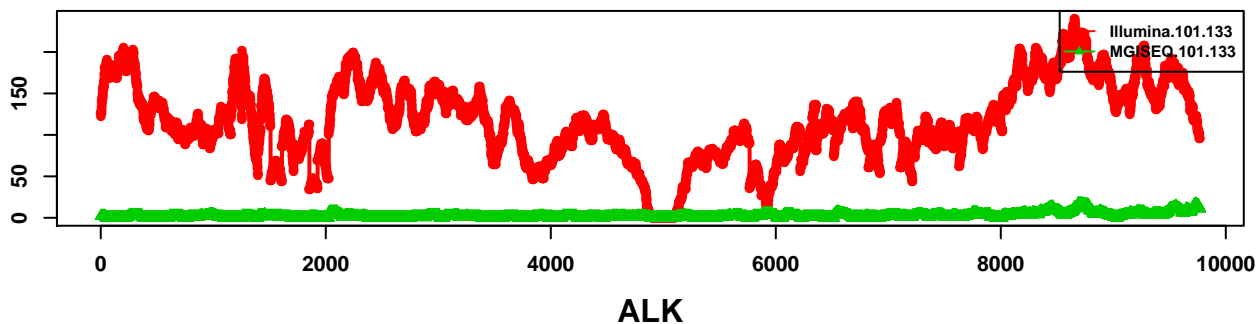

Sequencing Depth

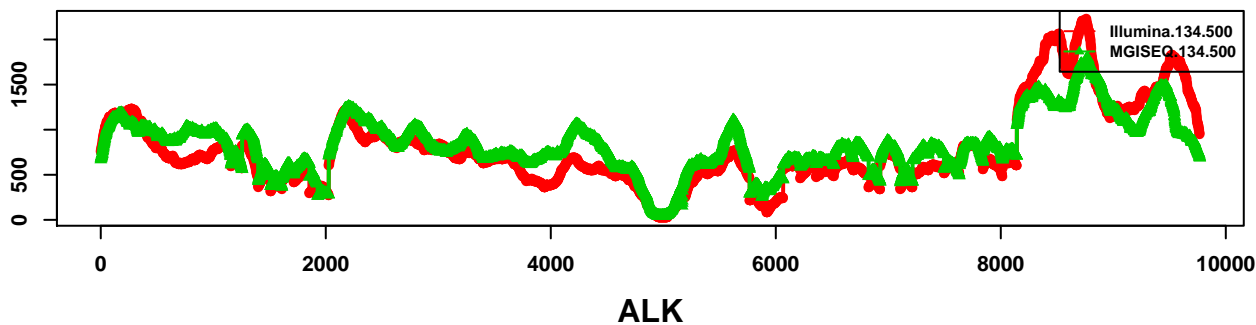

Supplement: Supplementary file 2 [file Presentation1.zip › ALK/19N01652F.pdf]

Sequencing Depth

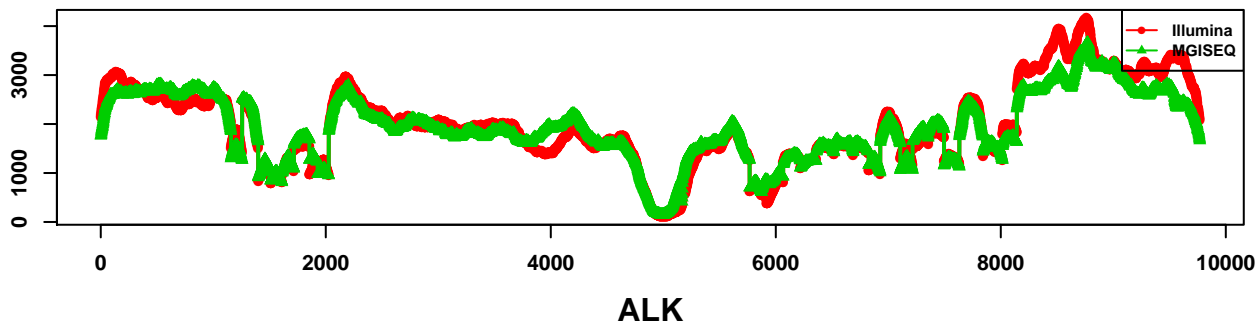

Sequencing Depth

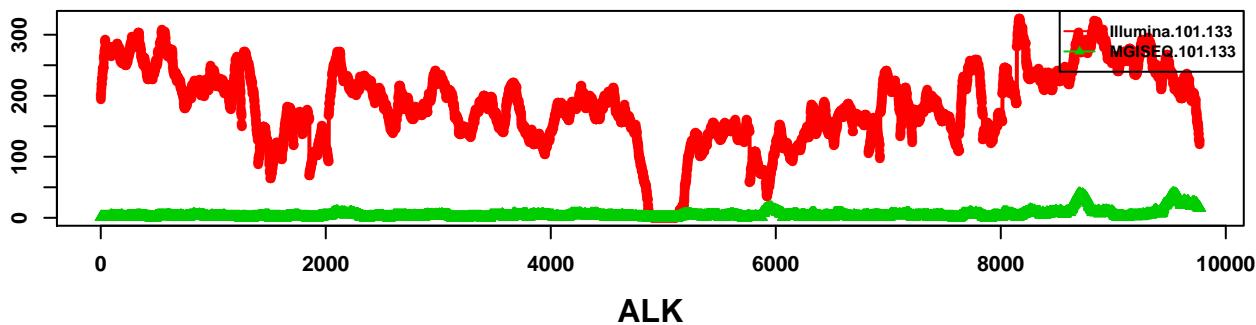

Sequencing Depth

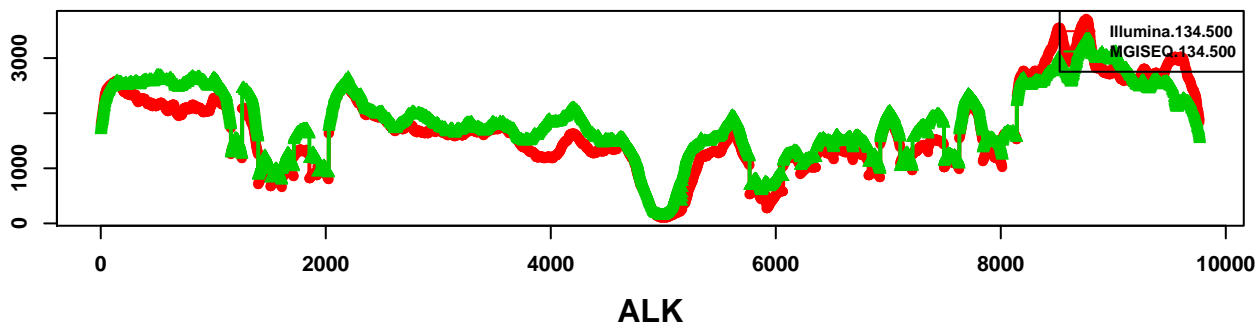

Supplement: Supplementary file 2 [file Presentation1.zip › ALK/19HE22006F.pdf]

Sequencing Depth

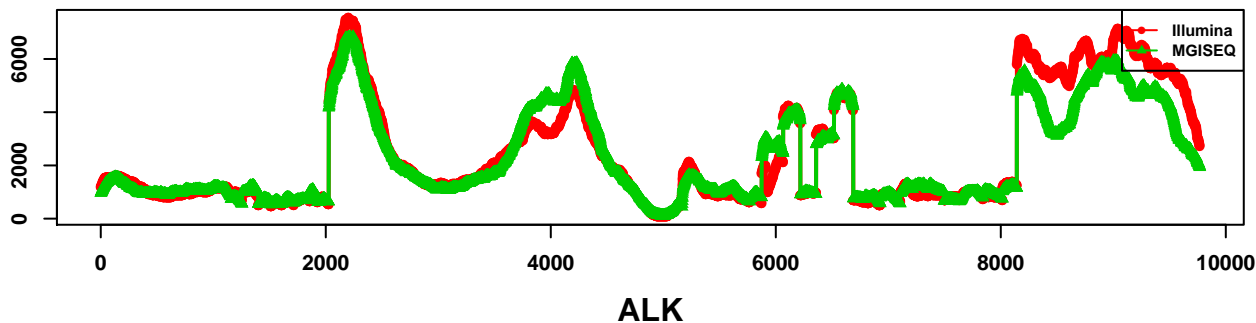

Sequencing Depth

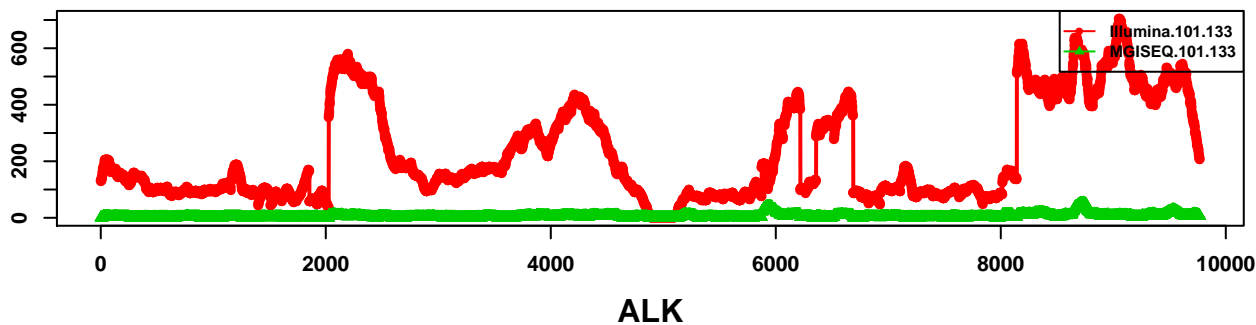

Sequencing Depth

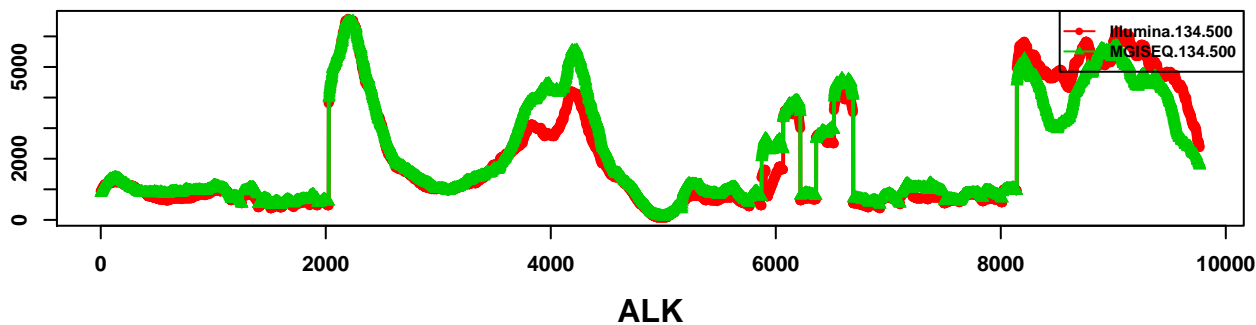

Supplement: Supplementary file 2 [file Presentation1.zip › ALK/19FC40248F.pdf]

Sequencing Depth

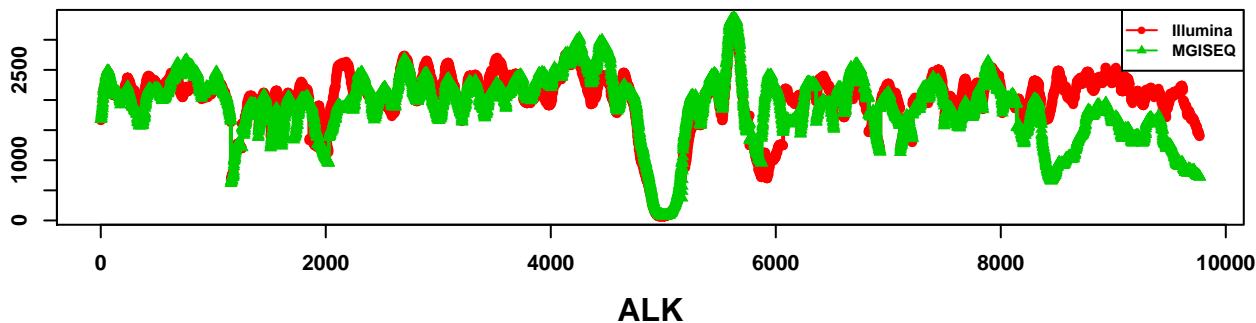

Sequencing Depth

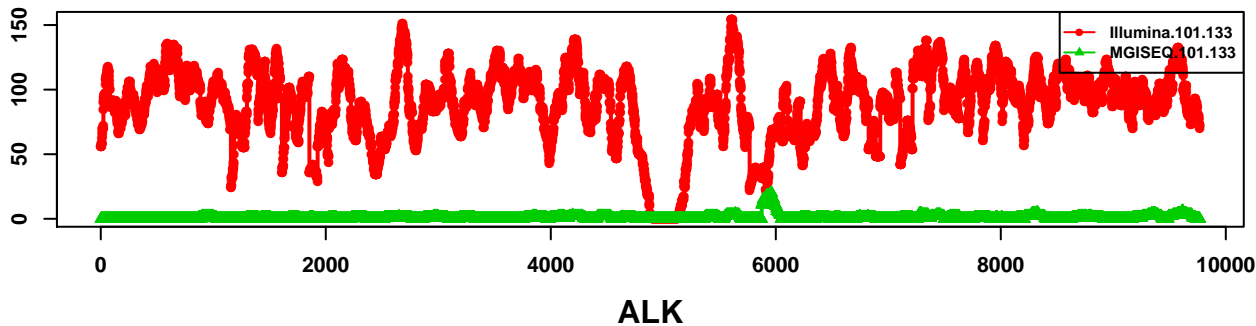

Sequencing Depth

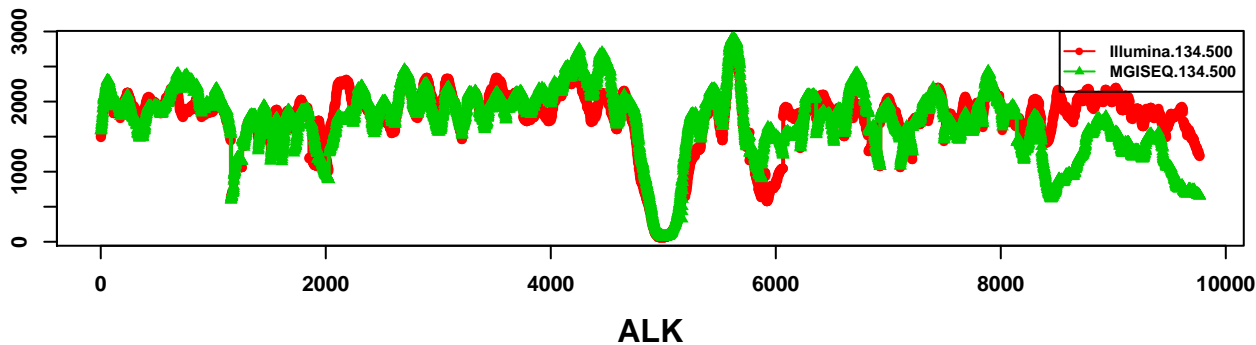

Supplement: Supplementary file 2 [file Presentation1.zip › ALK/19N01668P.pdf]

Sequencing Depth

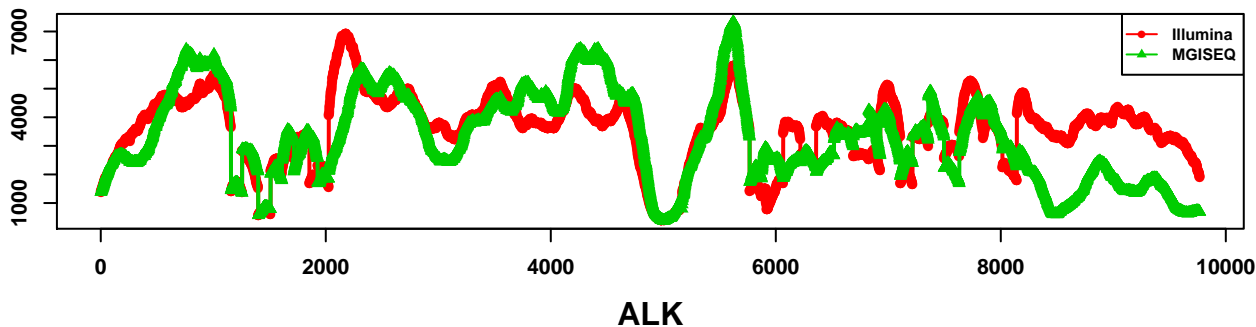

Sequencing Depth

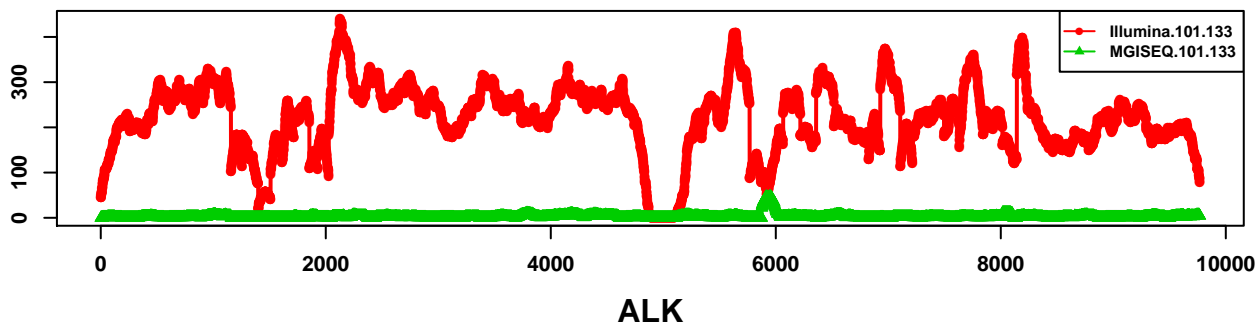

Sequencing Depth

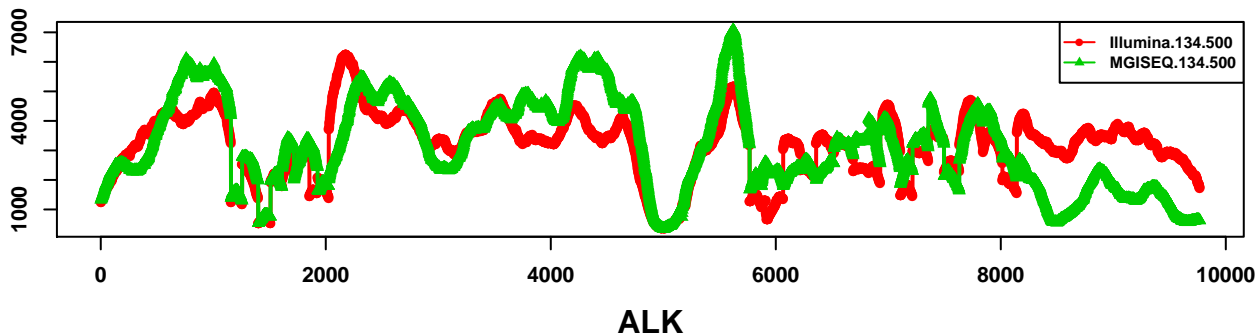

Supplement: Supplementary file 2 [file Presentation1.zip › ALK/19N01669T.pdf]

Sequencing Depth

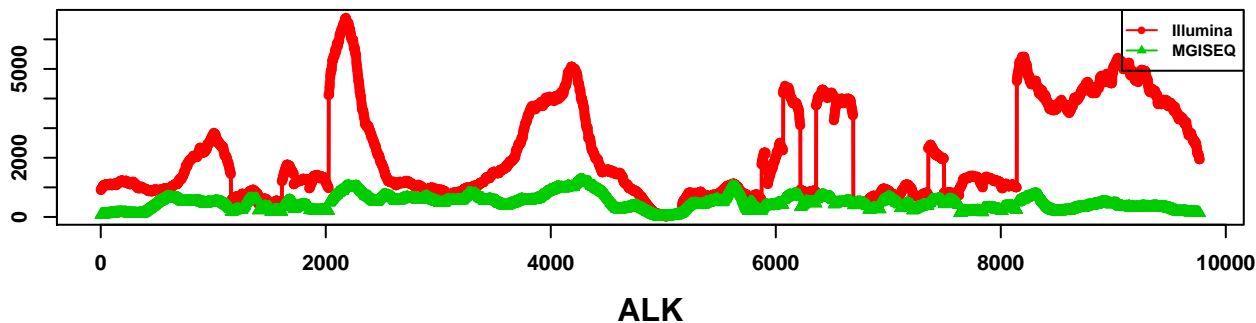

Sequencing Depth

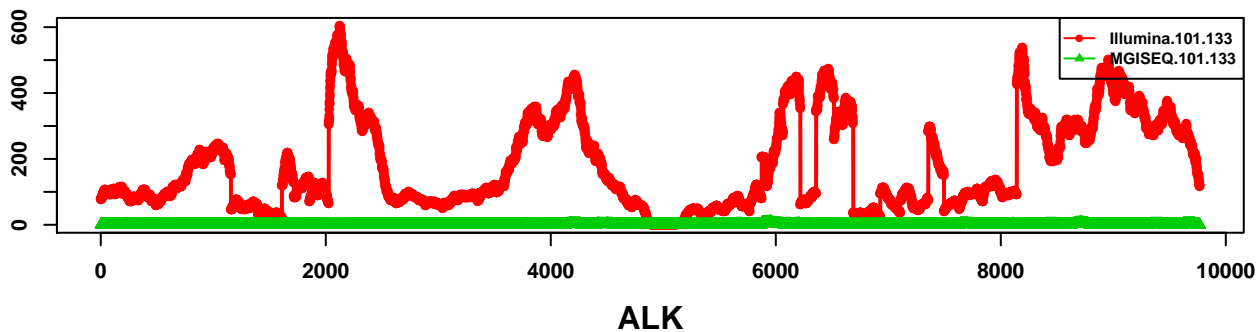

Sequencing Depth

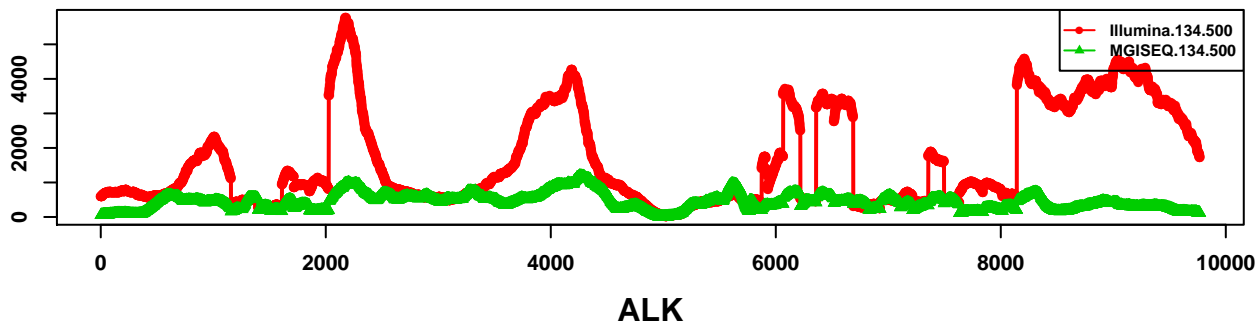

Supplement: Supplementary file 2 [file Presentation1.zip › ALK/19N01664F.pdf]

Sequencing Depth

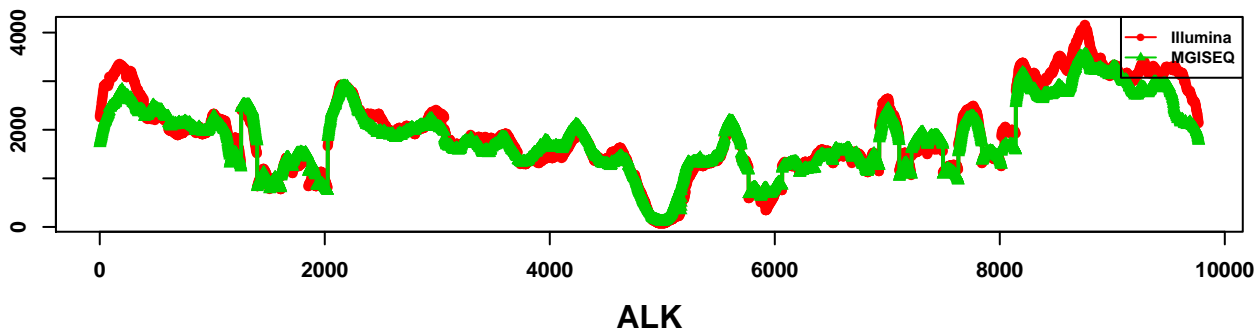

Sequencing Depth

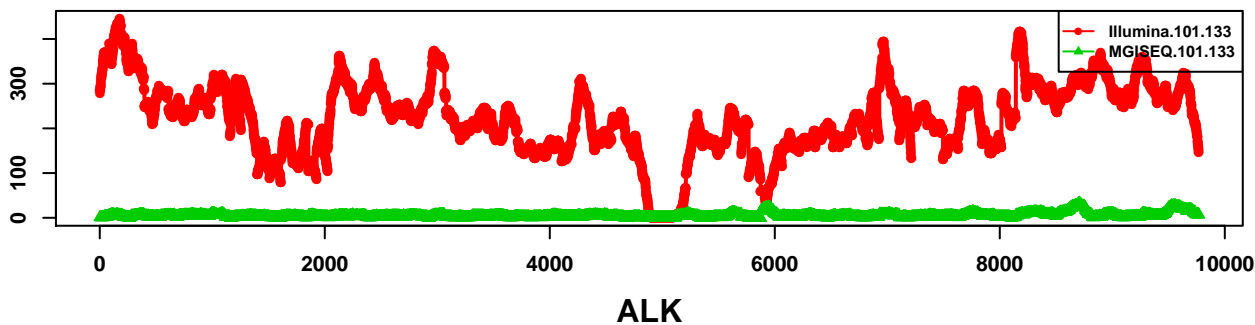

Sequencing Depth

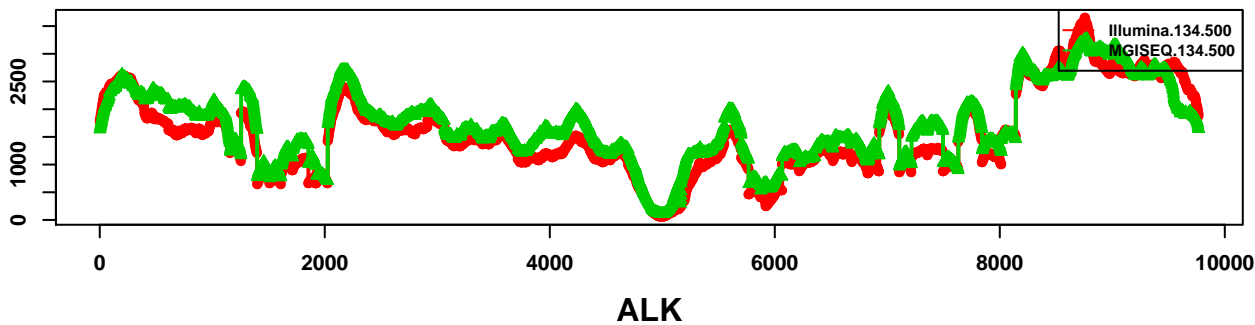

Supplement: Supplementary file 2 [file Presentation1.zip › ALK/19HE22067F.pdf]

Sequencing Depth

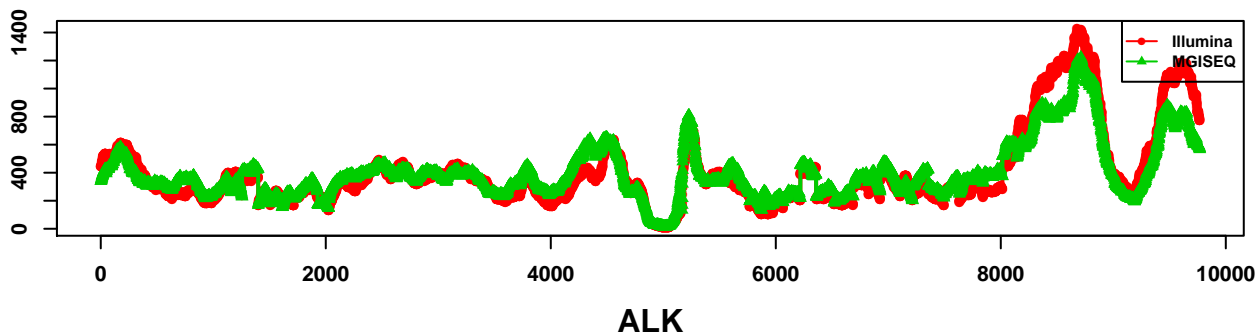

Sequencing Depth

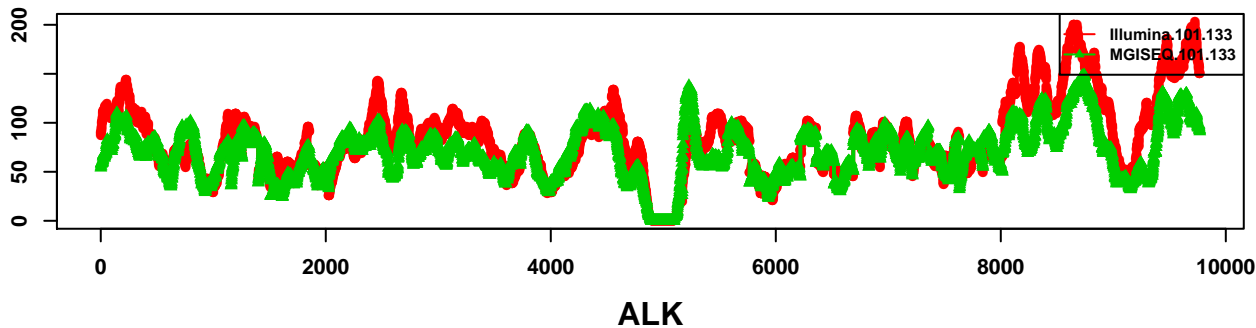

Sequencing Depth

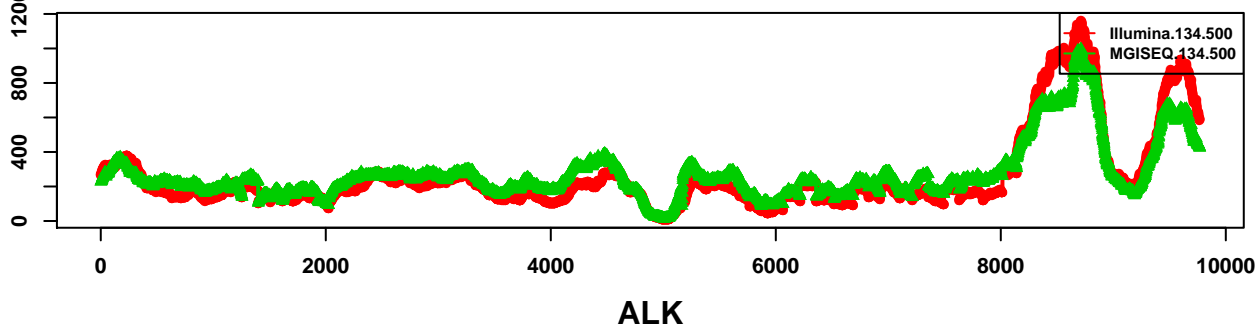

Supplement: Supplementary file 2 [file Presentation1.zip › ALK/19CF15713F.pdf]

Sequencing Depth

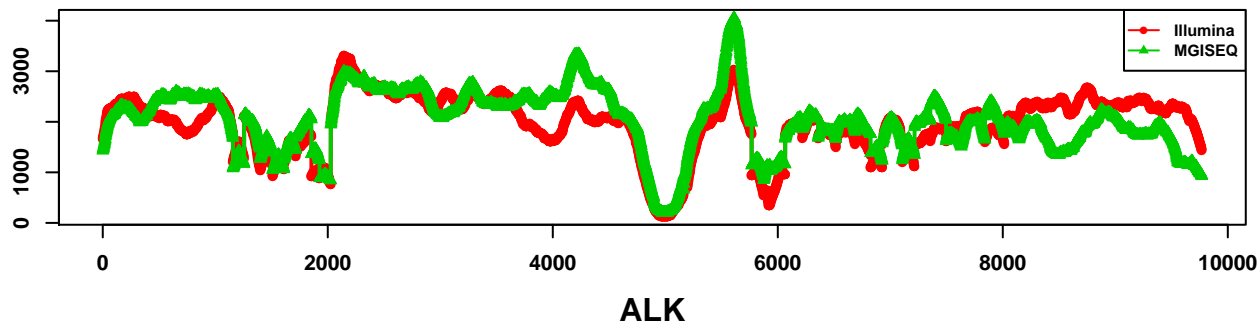

Sequencing Depth

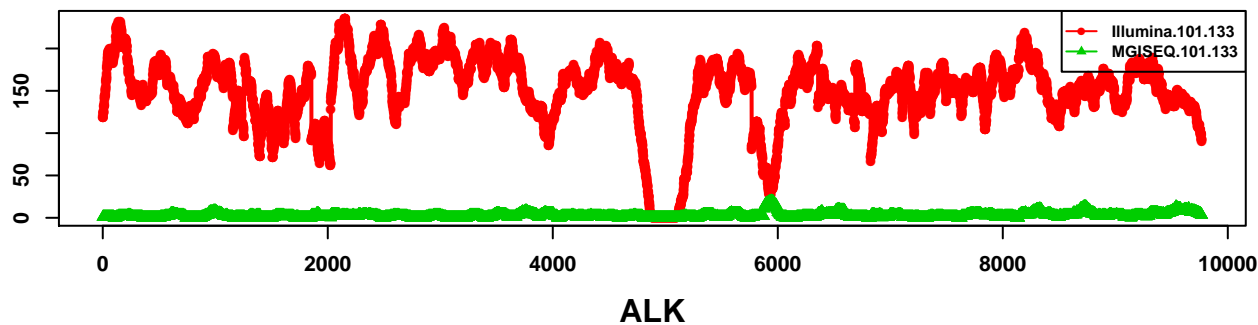

Sequencing Depth

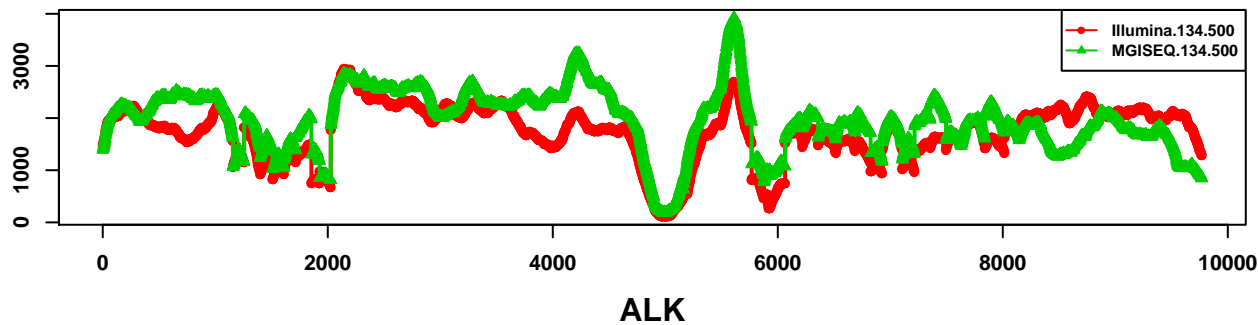

Supplement: Supplementary file 2 [file Presentation1.zip › ALK/19ZN12352H-R.pdf]

Sequencing Depth

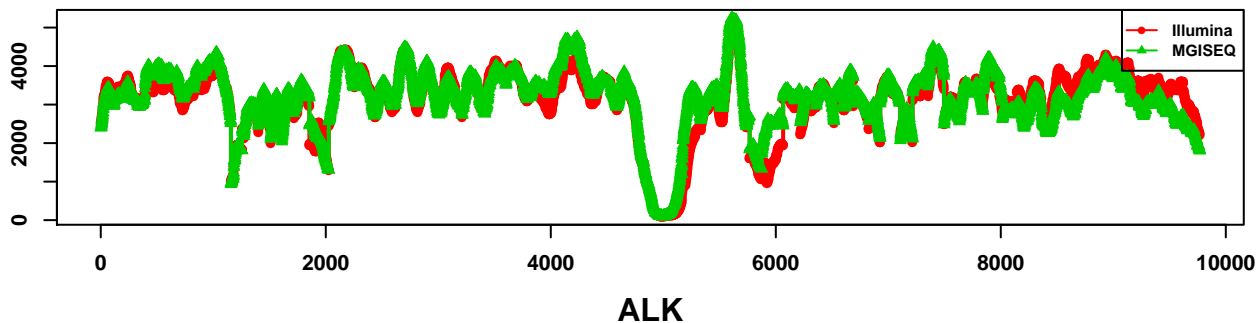

Sequencing Depth

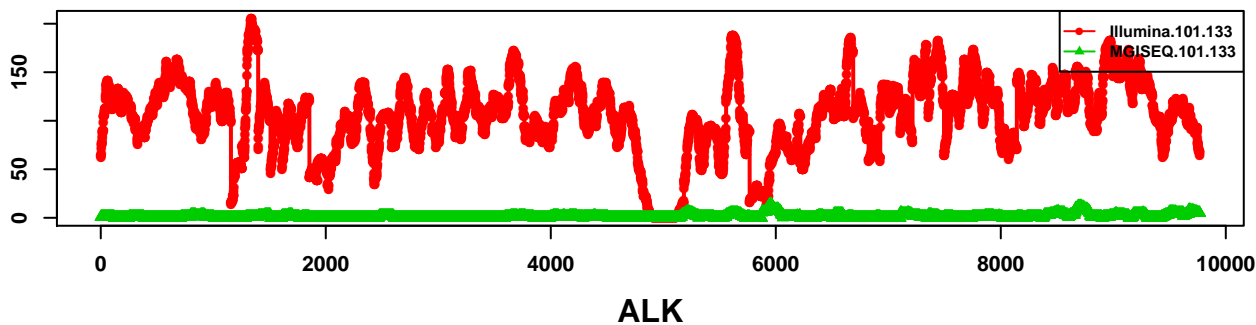

Sequencing Depth

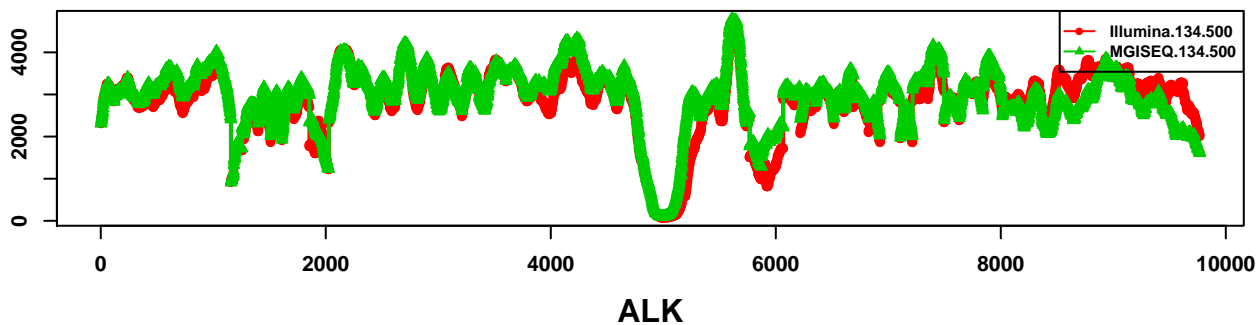

Supplement: Supplementary file 2 [file Presentation1.zip › ALK/19ZY39353P.pdf]

Sequencing Depth

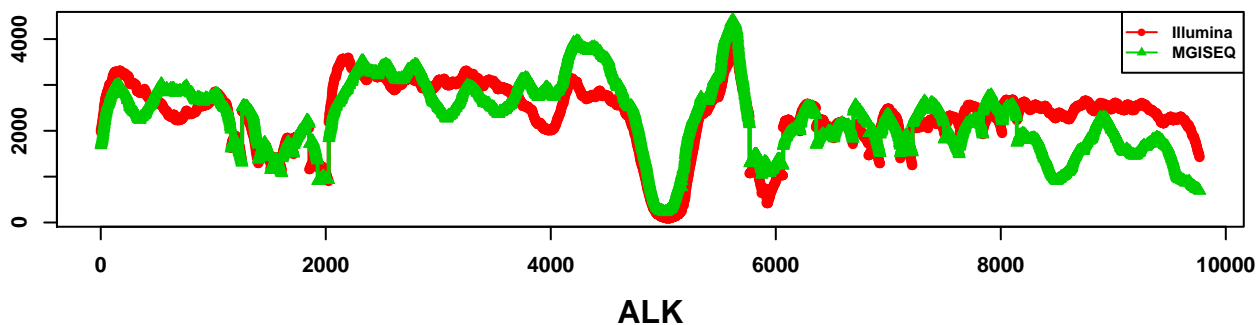

Sequencing Depth

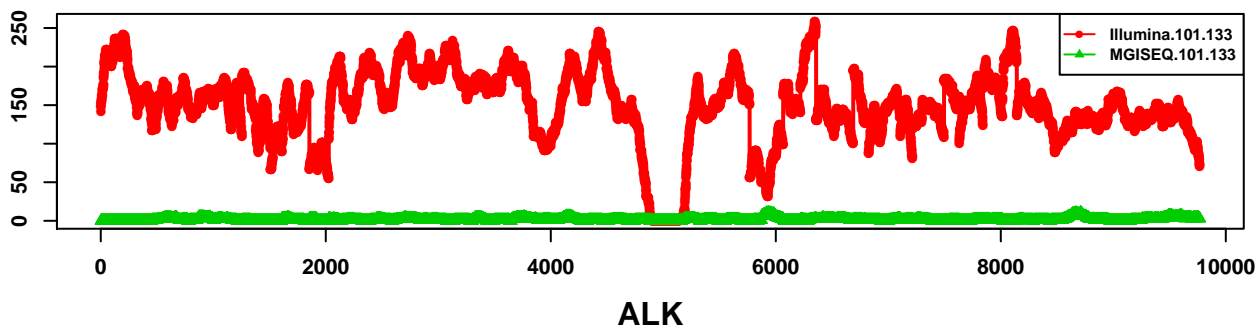

Sequencing Depth

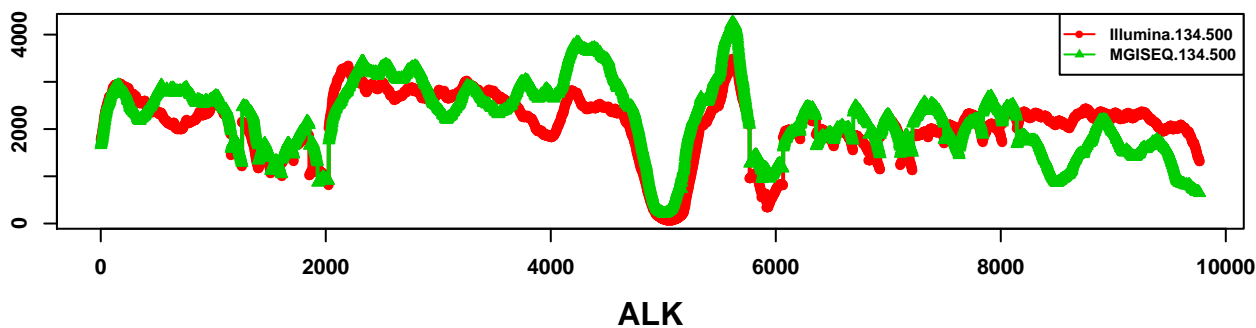

Supplement: Supplementary file 2 [file Presentation1.zip › ALK/19ZN12362B.pdf]

Sequencing Depth

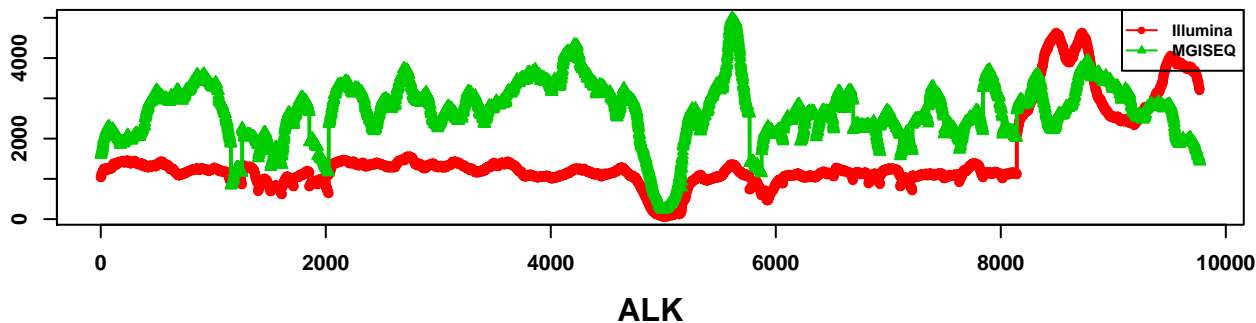

Sequencing Depth

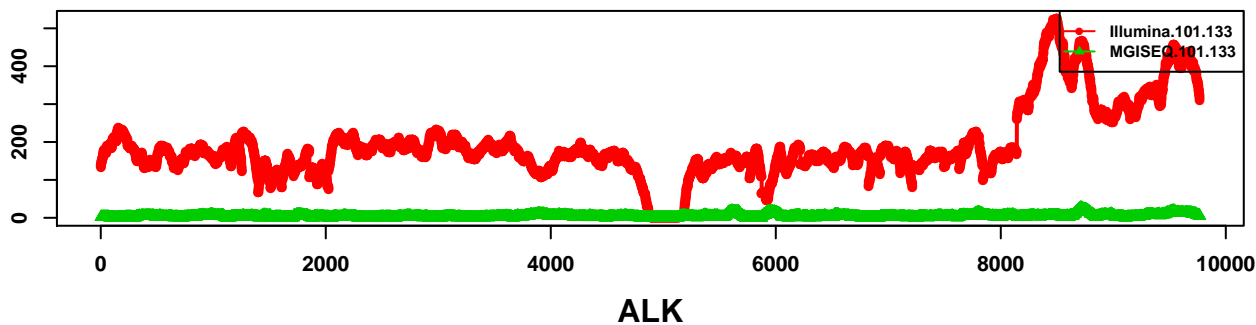

Sequencing Depth

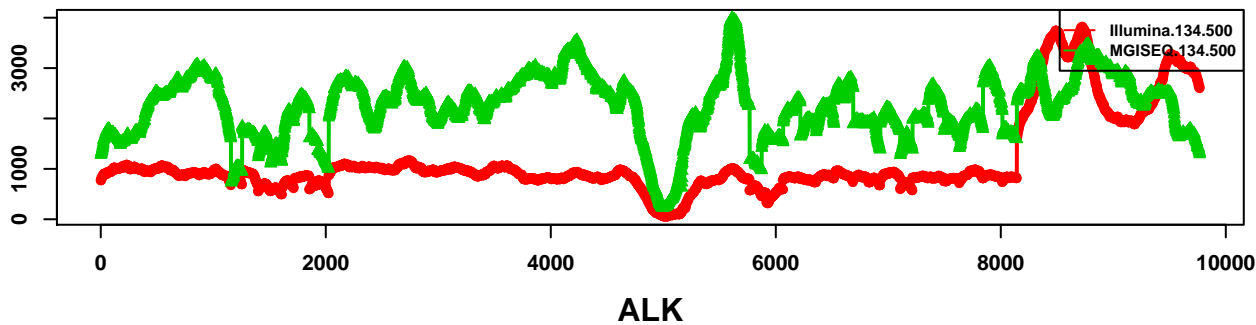

Supplement: Supplementary file 2 [file Presentation1.zip › ALK/19ZN12363F.pdf]

Sequencing Depth

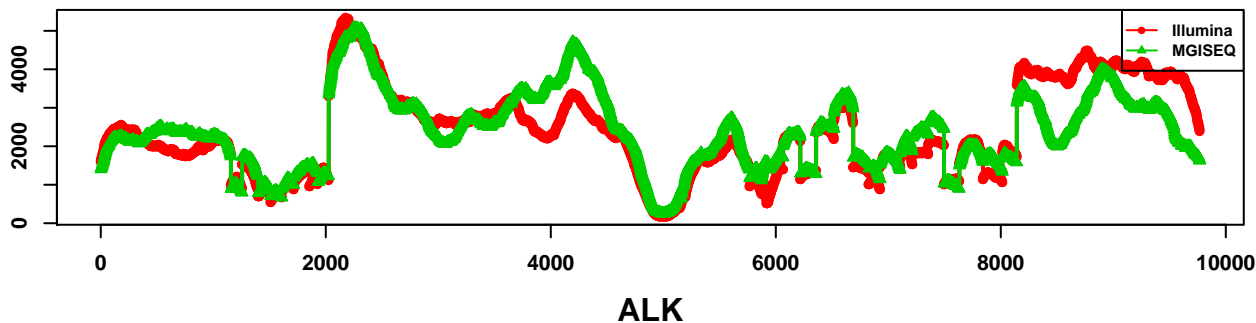

Sequencing Depth

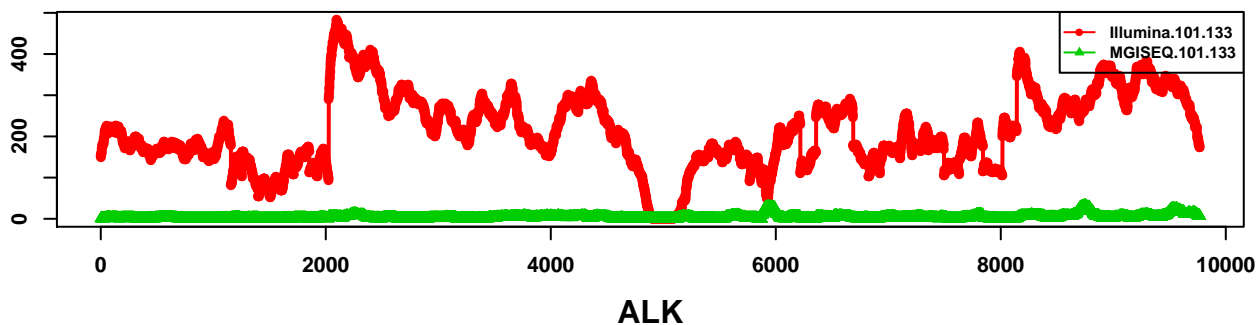

Sequencing Depth

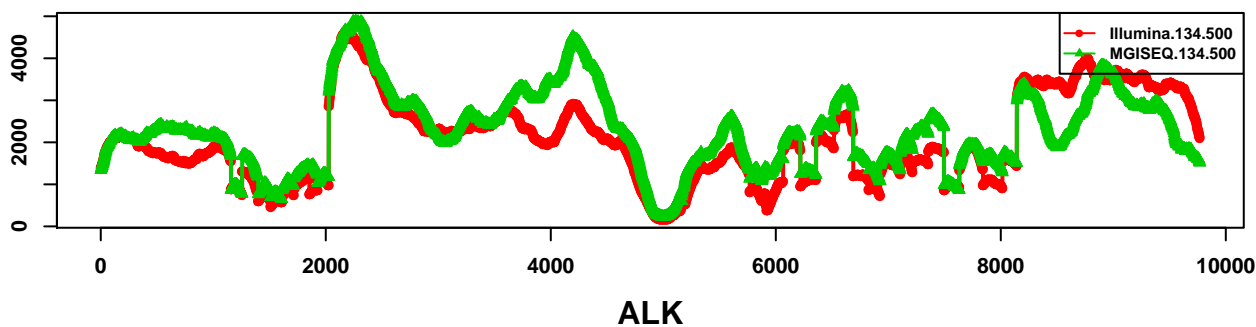

Supplement: Supplementary file 2 [file Presentation1.zip › ALK/19HE22134F.pdf]

Sequencing Depth

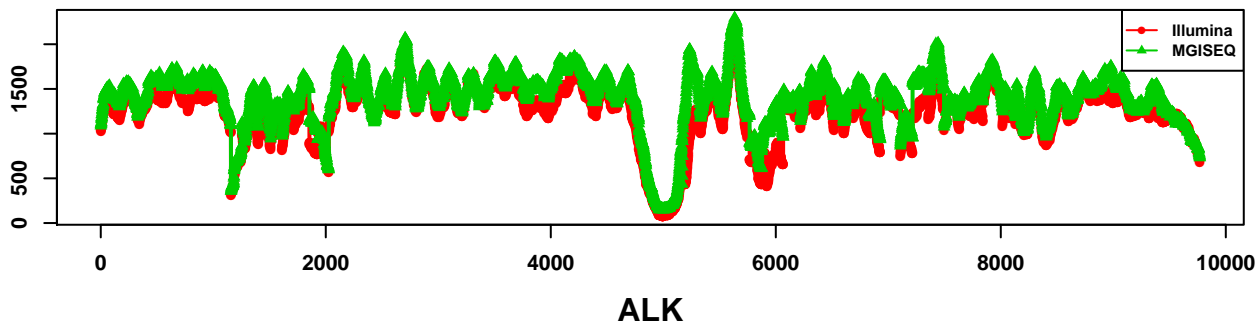

Sequencing Depth

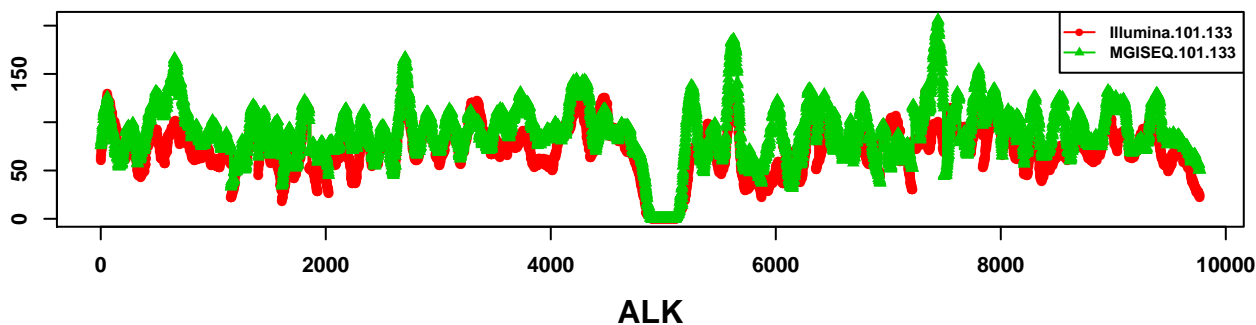

Sequencing Depth

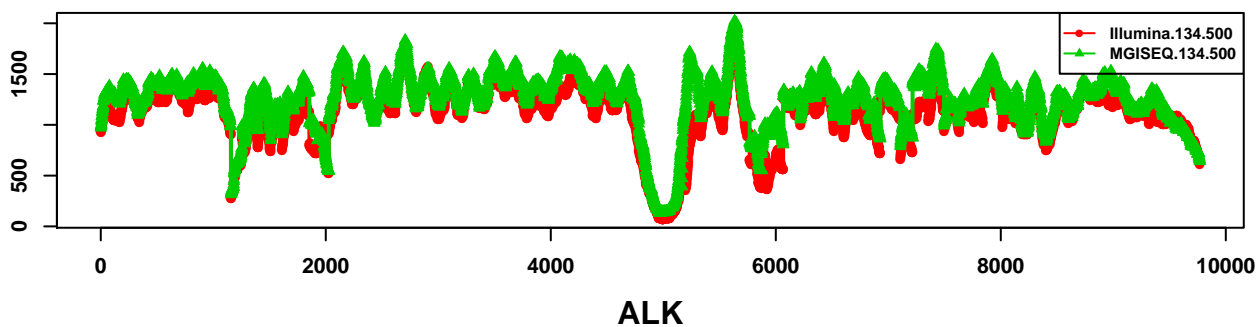

Supplement: Supplementary file 2 [file Presentation1.zip › ALK/19ZY39607P.pdf]

Sequencing Depth

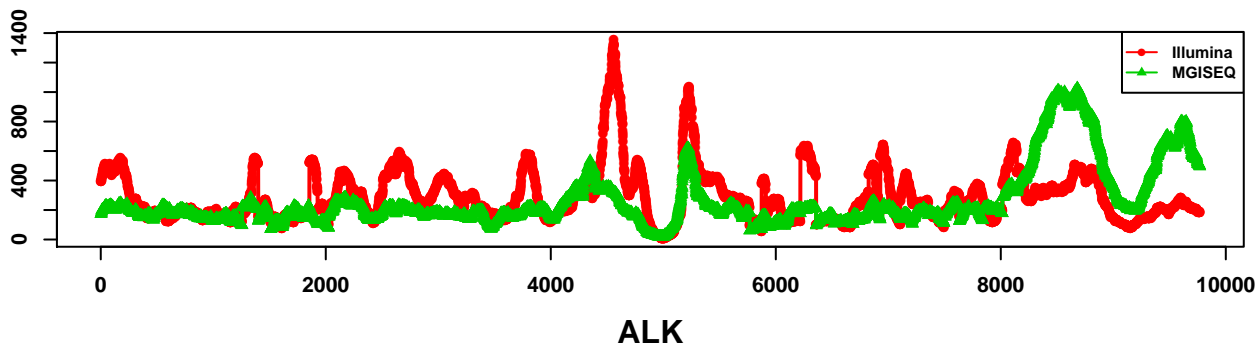

Sequencing Depth

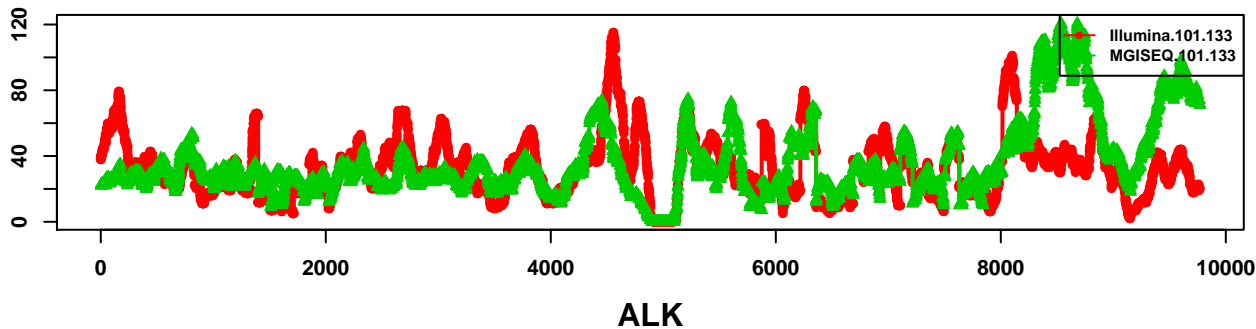

Sequencing Depth

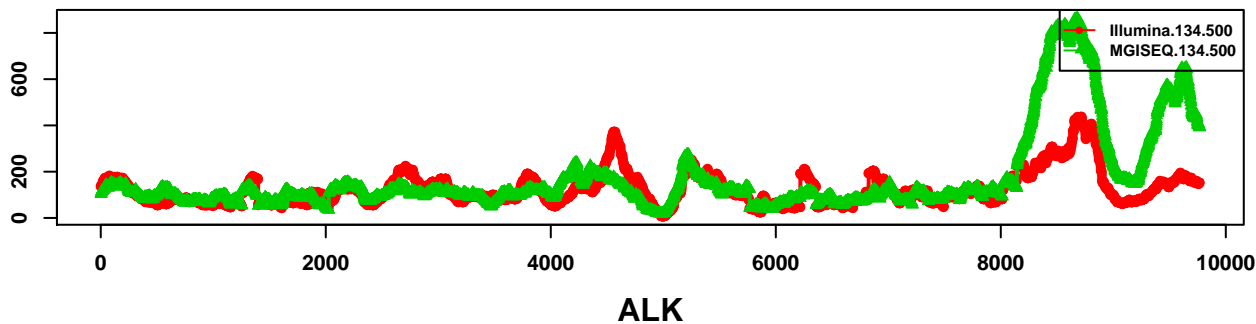

Supplement: Supplementary file 2 [file Presentation1.zip › ALK/19FC40415F.pdf]

Sequencing Depth

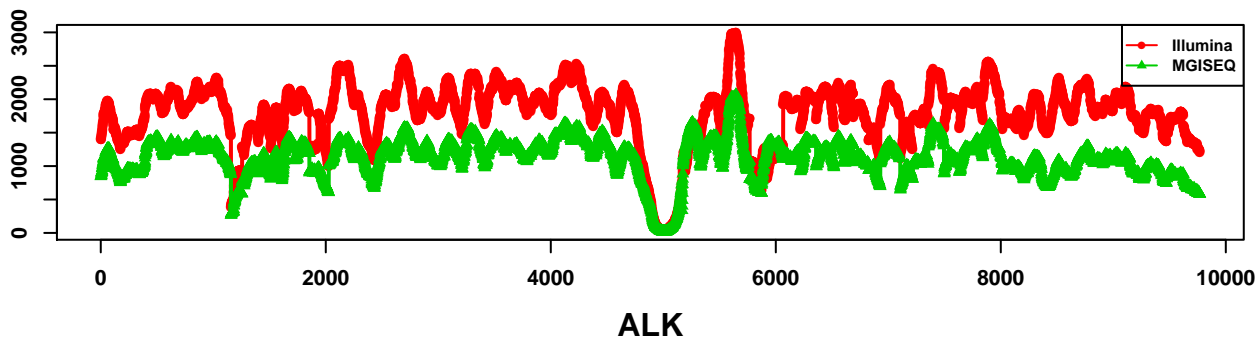

Sequencing Depth

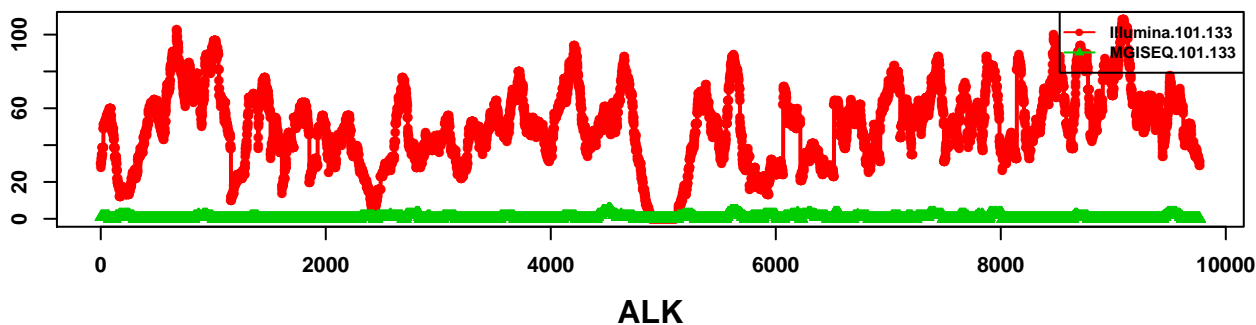

Sequencing Depth

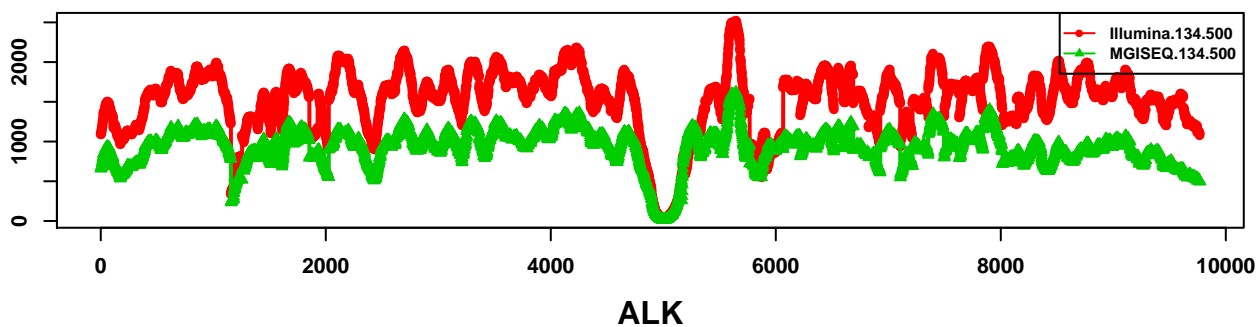

Supplement: Supplementary file 2 [file Presentation1.zip › ALK/19N01627-IIP.pdf]

Sequencing Depth

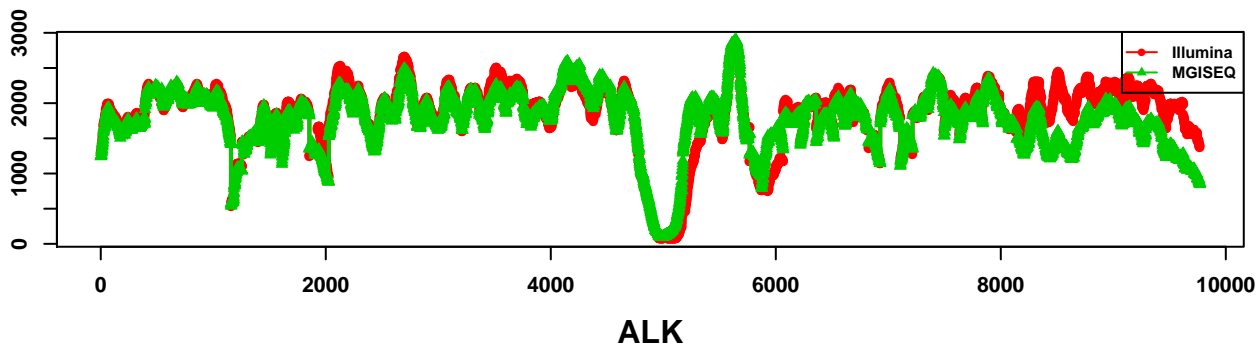

Sequencing Depth

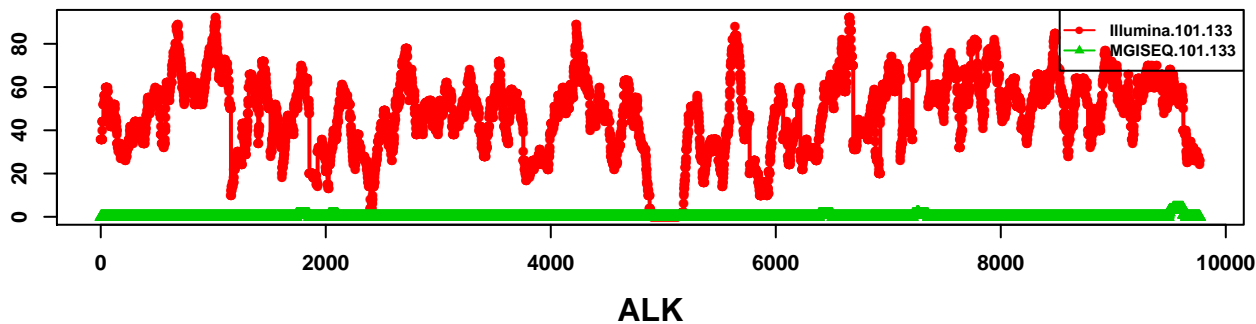

Sequencing Depth

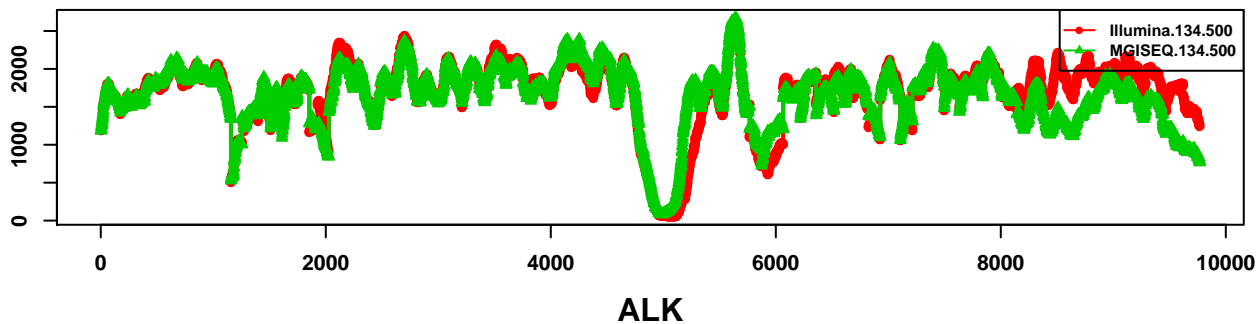

Supplement: Supplementary file 2 [file Presentation1.zip › ALK/19CF15528P.pdf]

Sequencing Depth

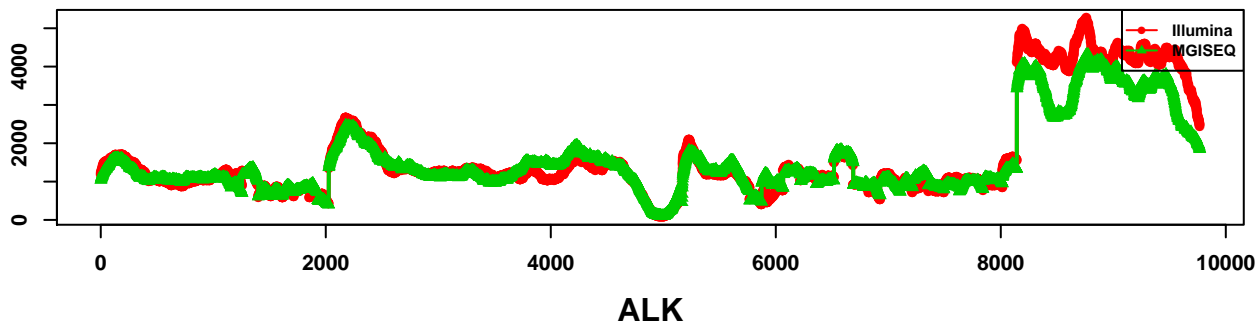

Sequencing Depth

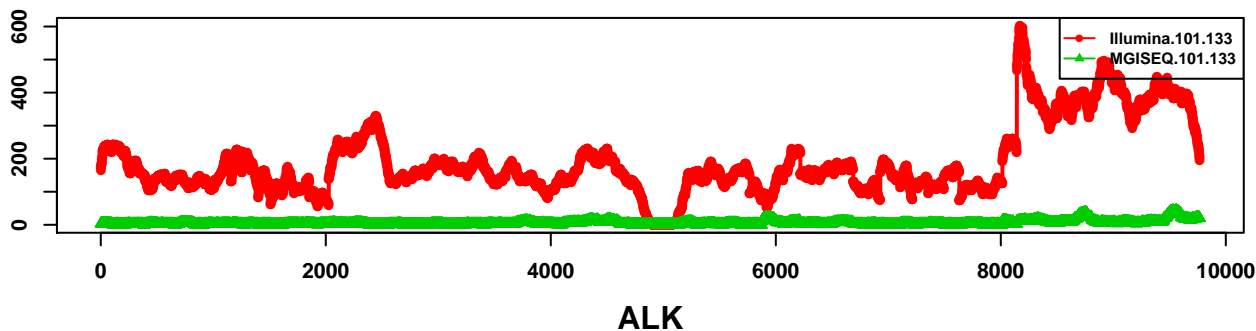

Sequencing Depth

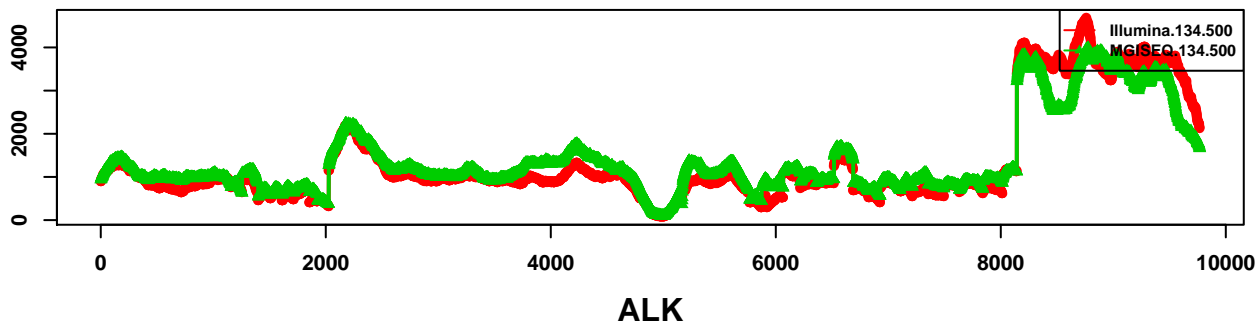

Supplement: Supplementary file 2 [file Presentation1.zip › ALK/19FC40245F.pdf]

Sequencing Depth

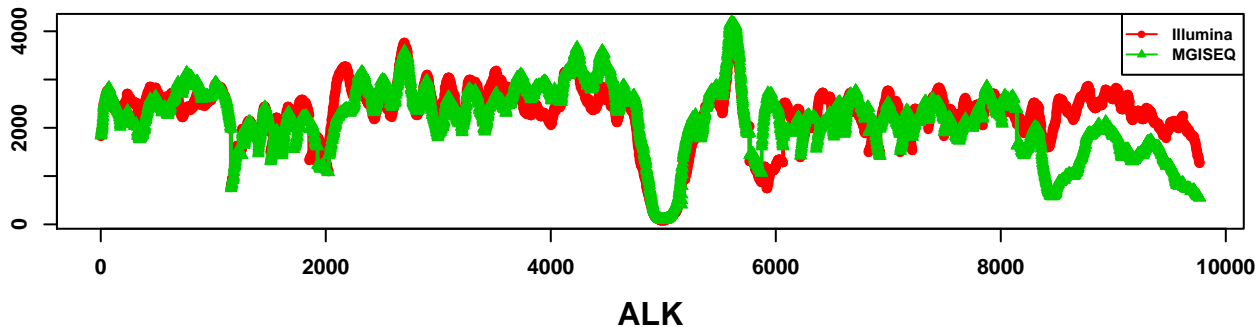

Sequencing Depth

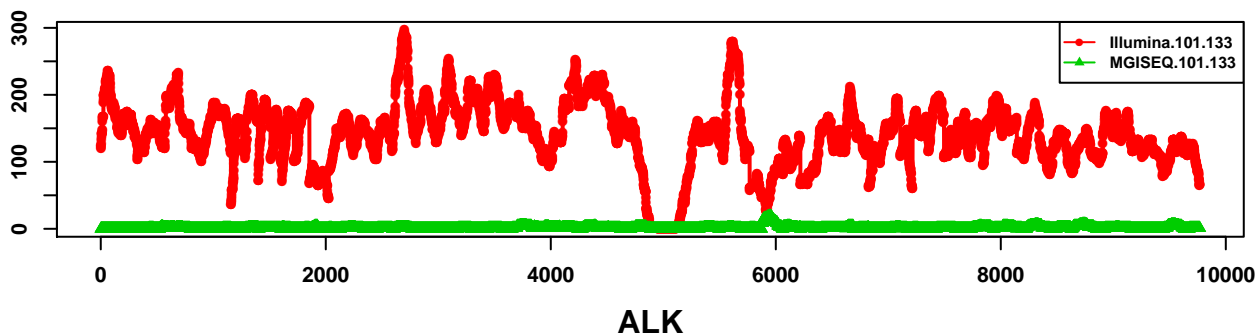

Sequencing Depth

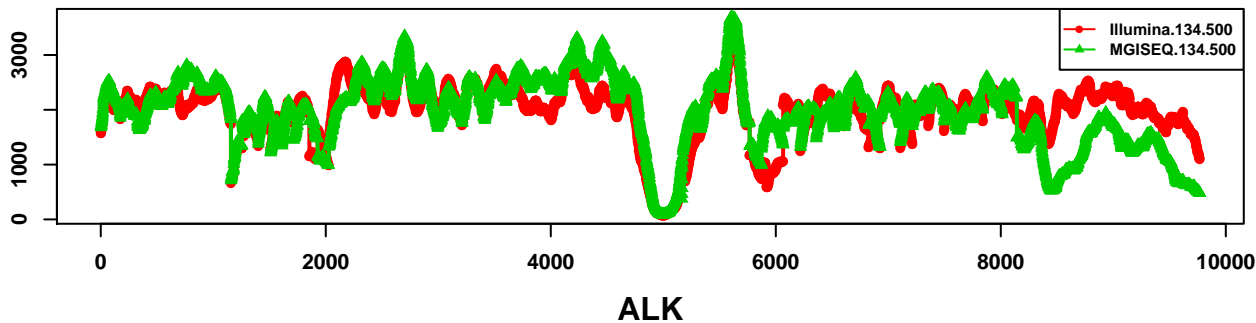

Supplement: Supplementary file 2 [file Presentation1.zip › ALK/19CF15529P.pdf]

Sequencing Depth

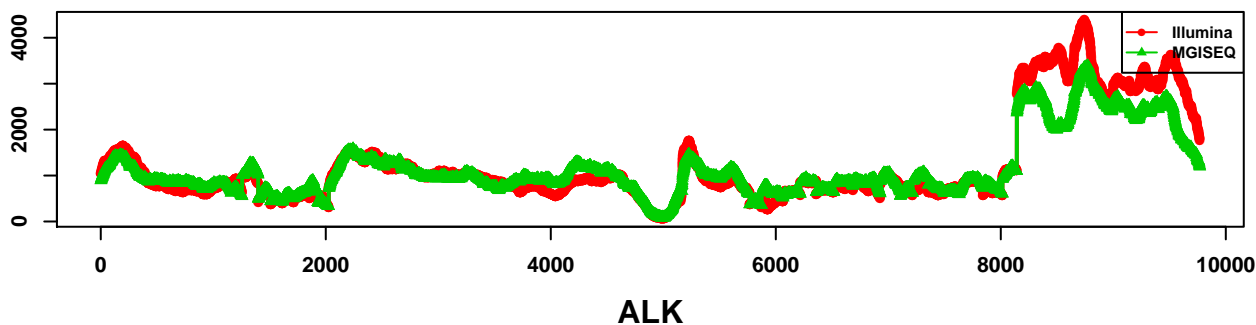

Sequencing Depth

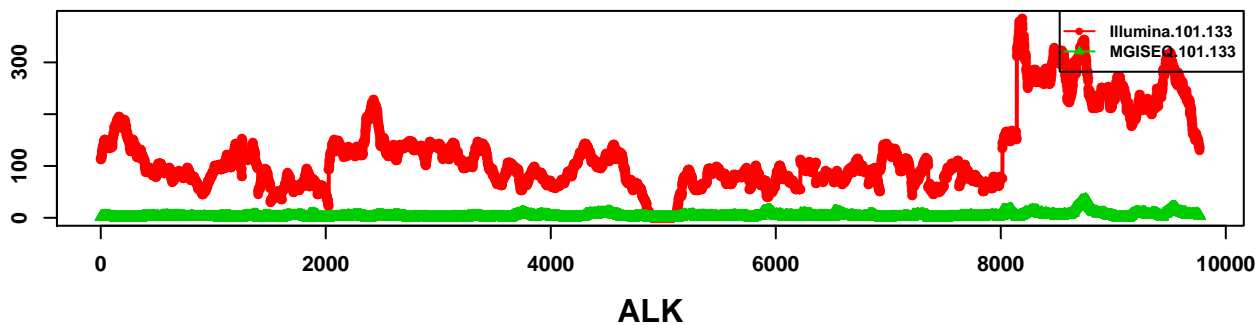

Sequencing Depth

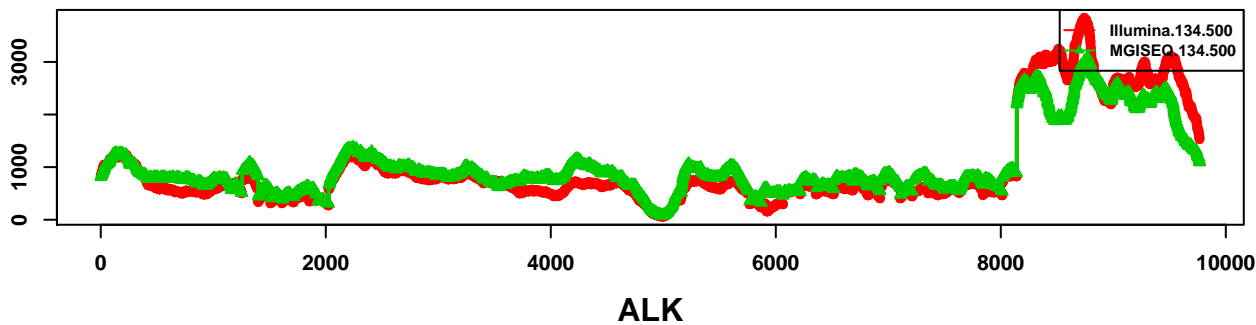

Supplement: Supplementary file 2 [file Presentation1.zip › ALK/19ZN12358F.pdf]

Sequencing Depth

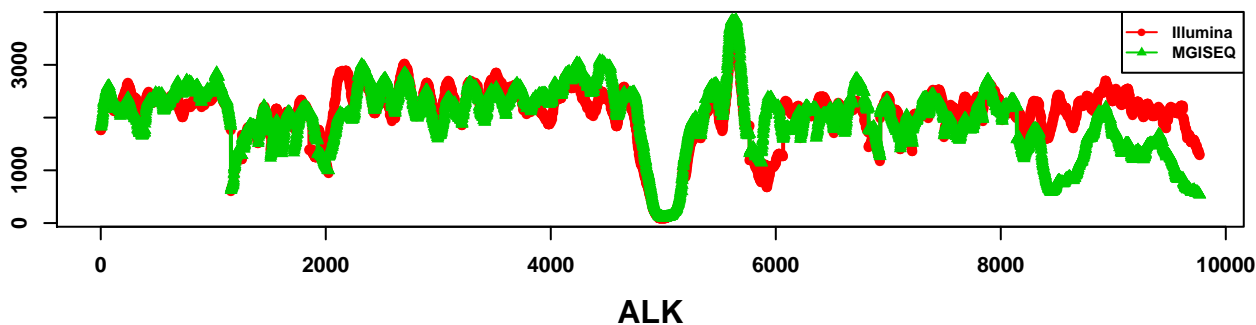

Sequencing Depth

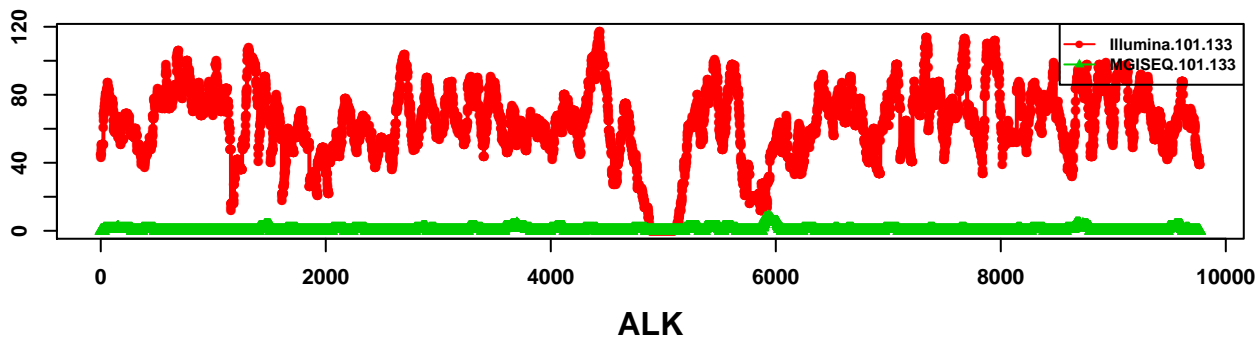

Sequencing Depth

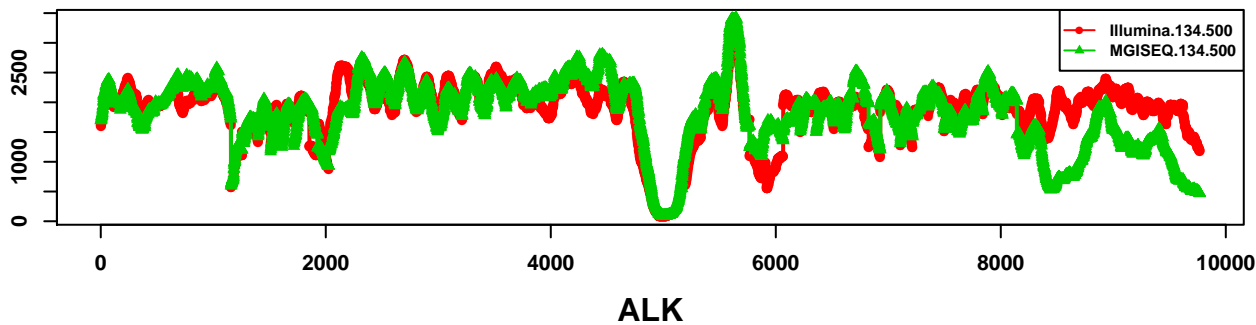

Supplement: Supplementary file 2 [file Presentation1.zip › ALK/19N01665P.pdf]

Sequencing Depth

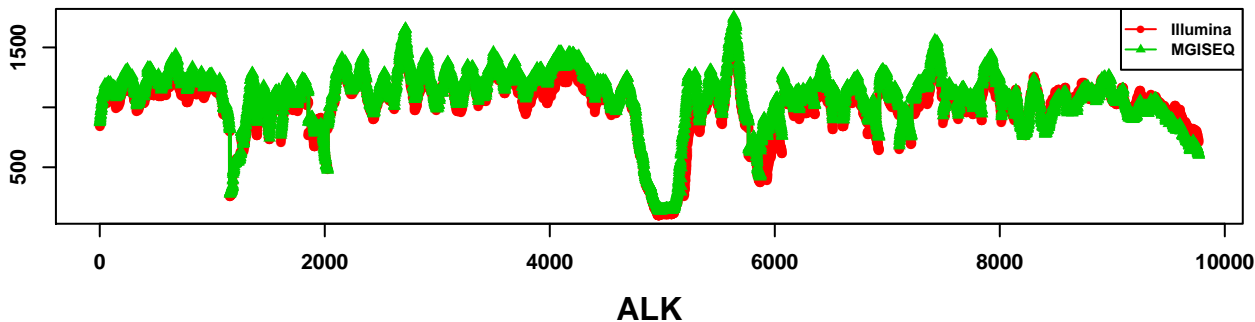

Sequencing Depth

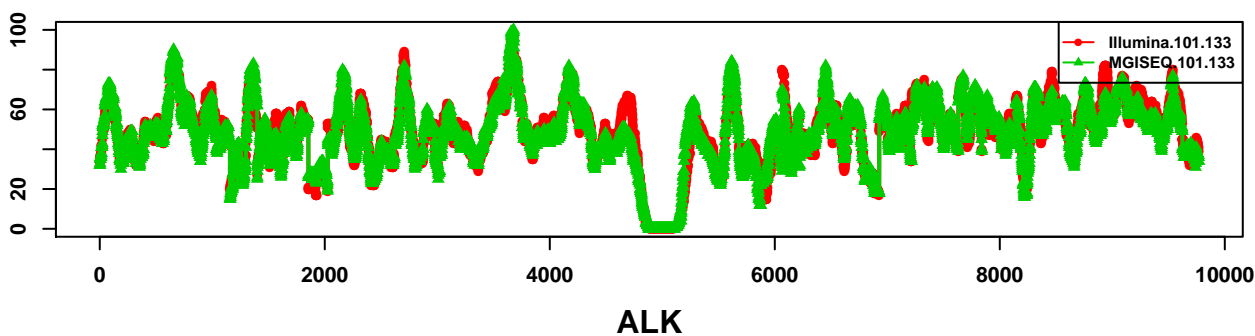

Sequencing Depth

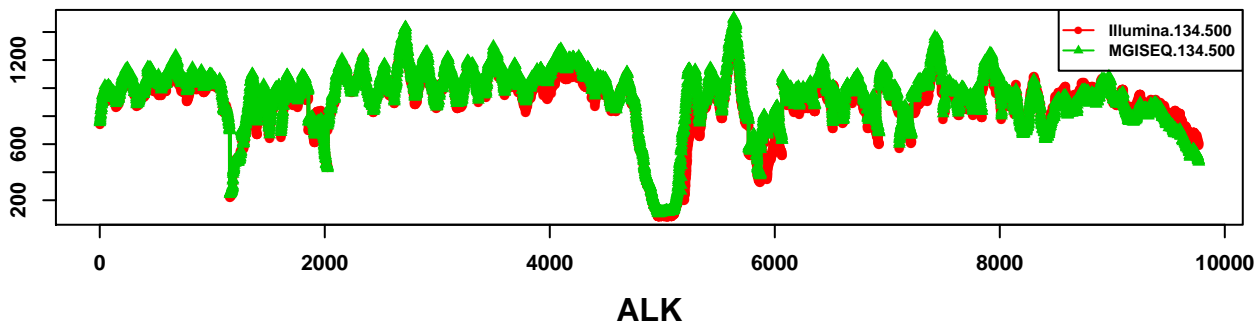

Supplement: Supplementary file 2 [file Presentation1.zip › ALK/19ZN12548P.pdf]

Sequencing Depth

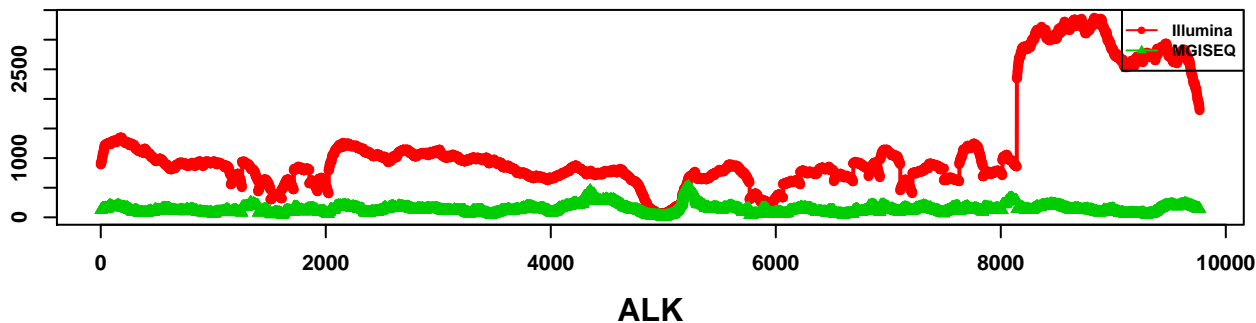

Sequencing Depth

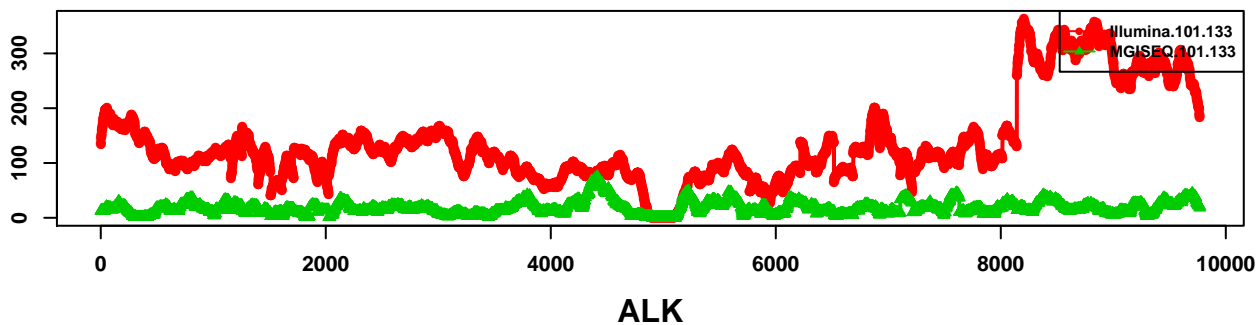

Sequencing Depth

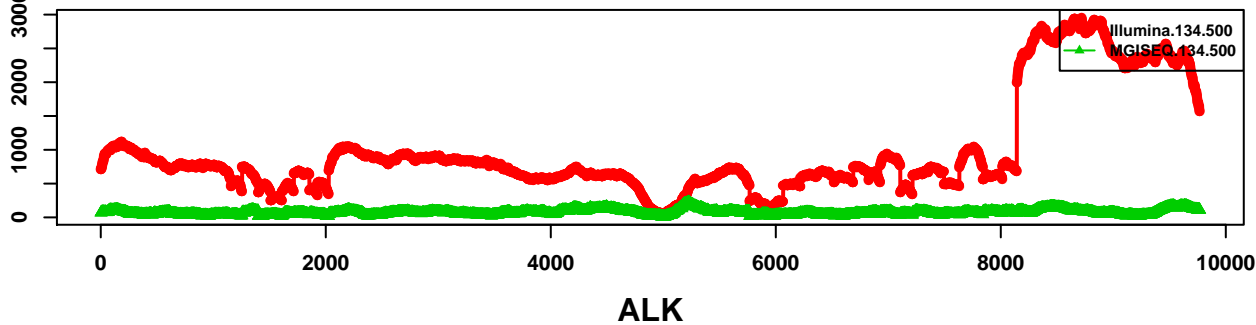

Supplement: Supplementary file 2 [file Presentation1.zip › ALK/19FC40438F.pdf]

Sequencing Depth

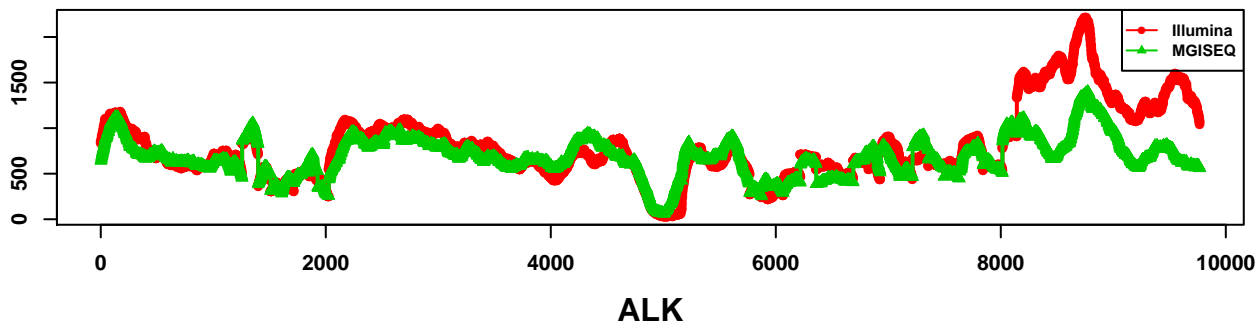

Sequencing Depth

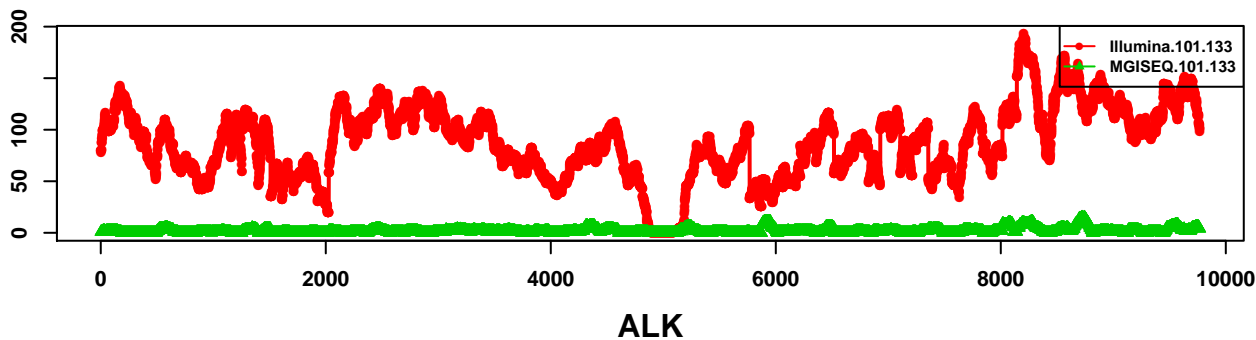

Sequencing Depth

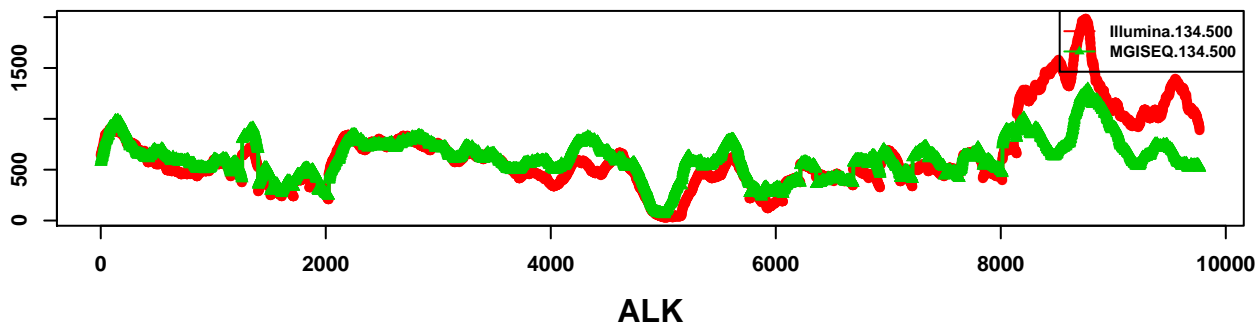

Supplement: Supplementary file 2 [file Presentation1.zip › ALK/19HS86178F.pdf]

Sequencing Depth

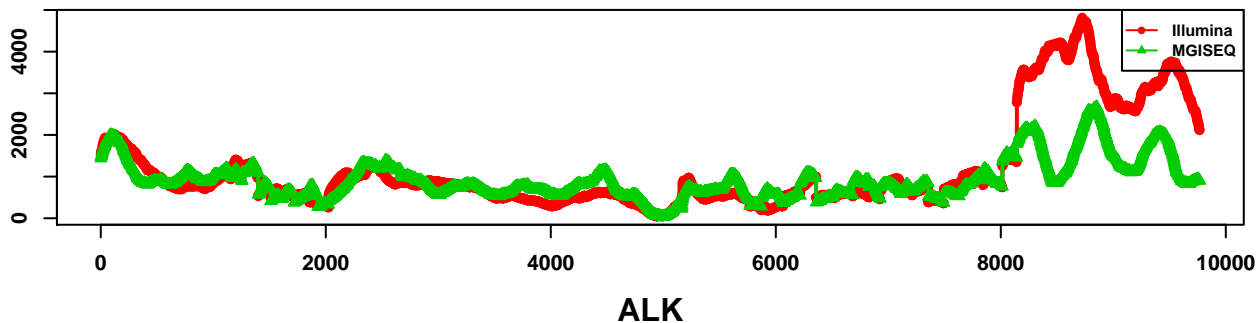

Sequencing Depth

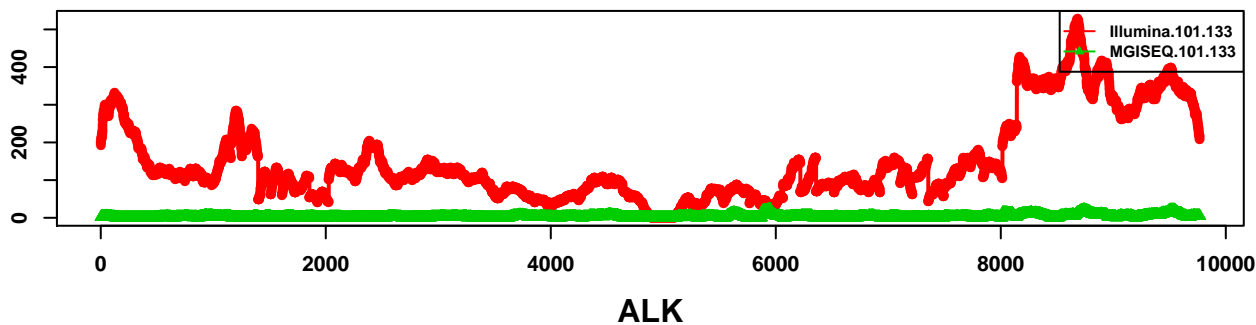

Sequencing Depth

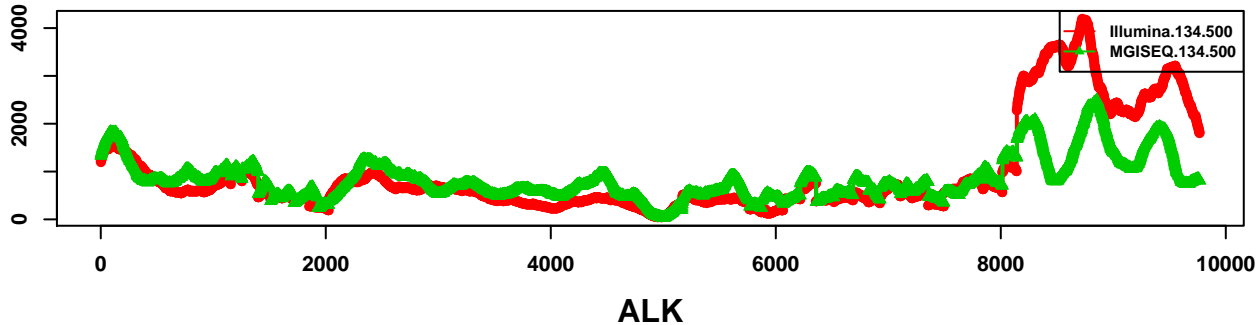

Supplement: Supplementary file 2 [file Presentation1.zip › ALK/19N01673F.pdf]

Sequencing Depth

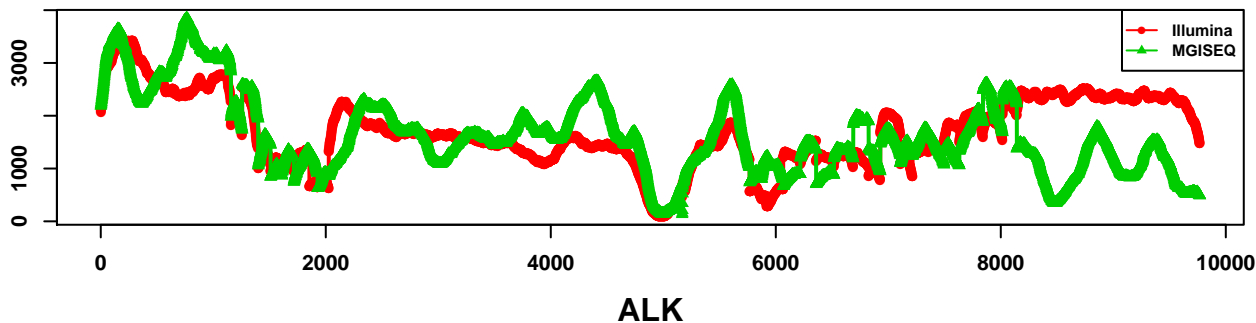

Sequencing Depth

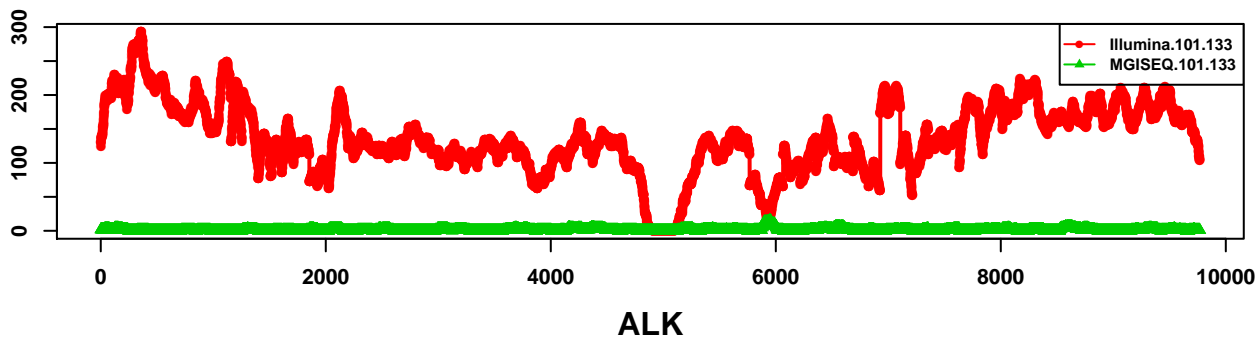

Sequencing Depth

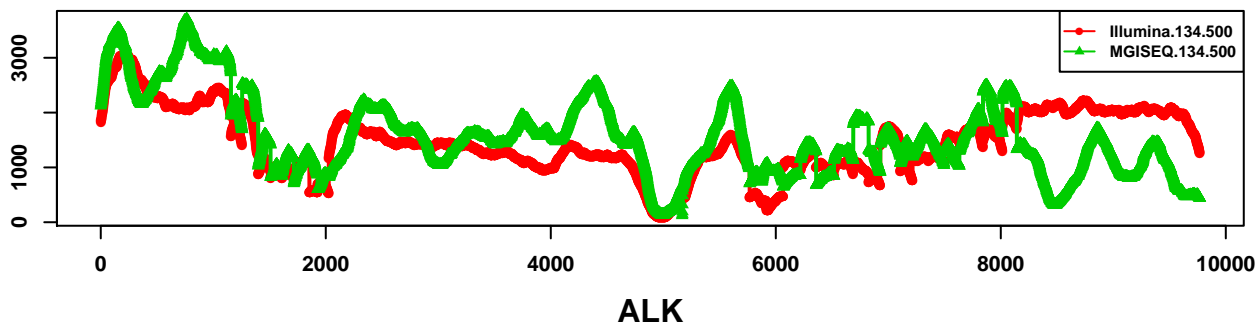

Supplement: Supplementary file 2 [file Presentation1.zip › ALK/19HE22162F.pdf]

Sequencing Depth

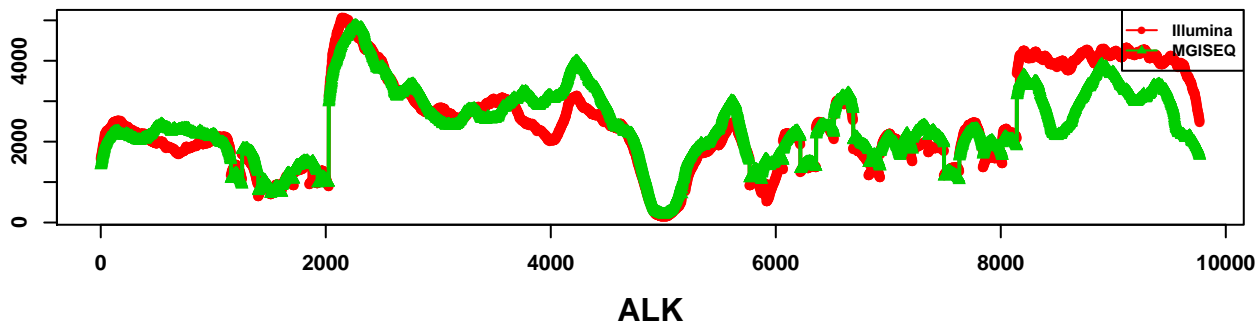

Sequencing Depth

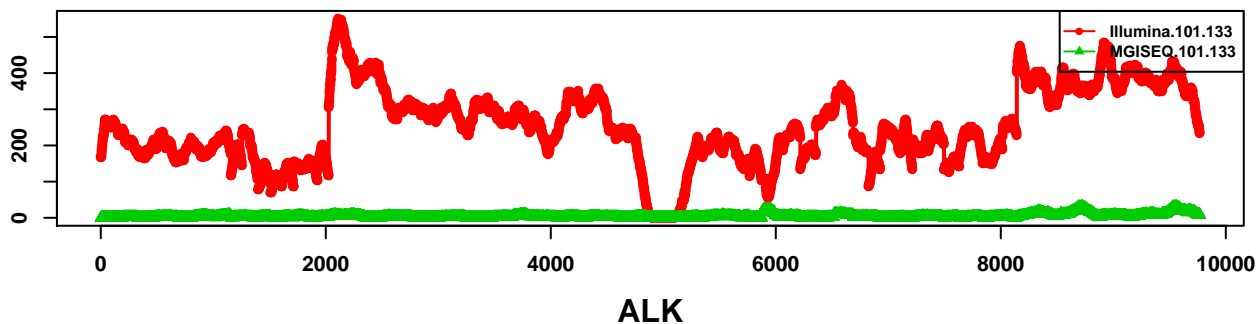

Sequencing Depth

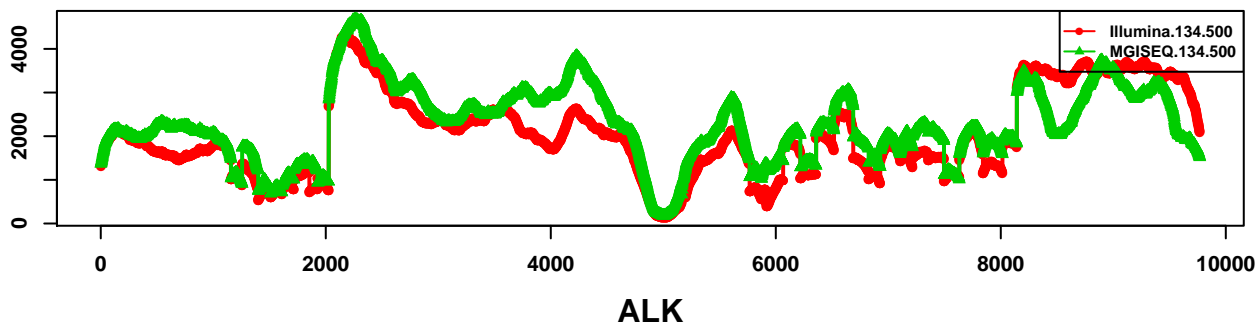

Supplement: Supplementary file 2 [file Presentation1.zip › ALK/19HE22135F.pdf]

Sequencing Depth

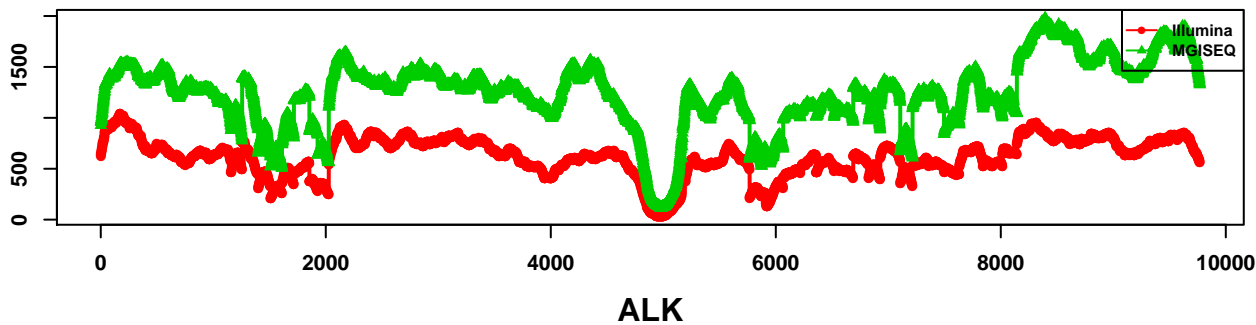

Sequencing Depth

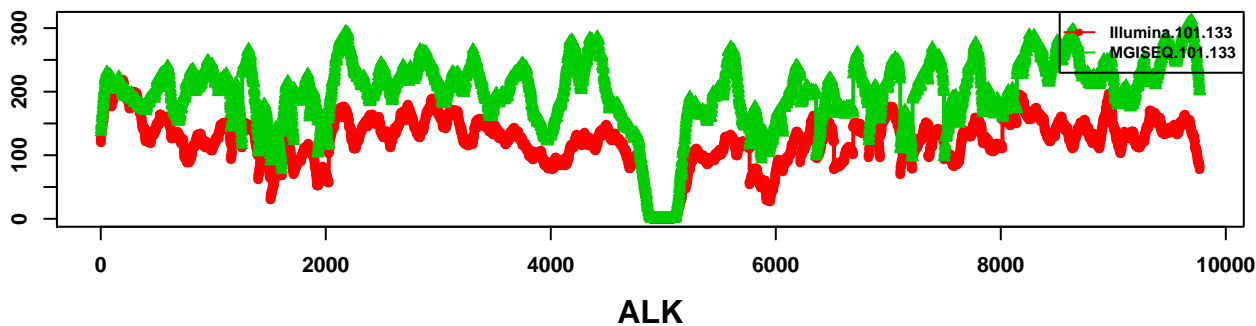

Sequencing Depth

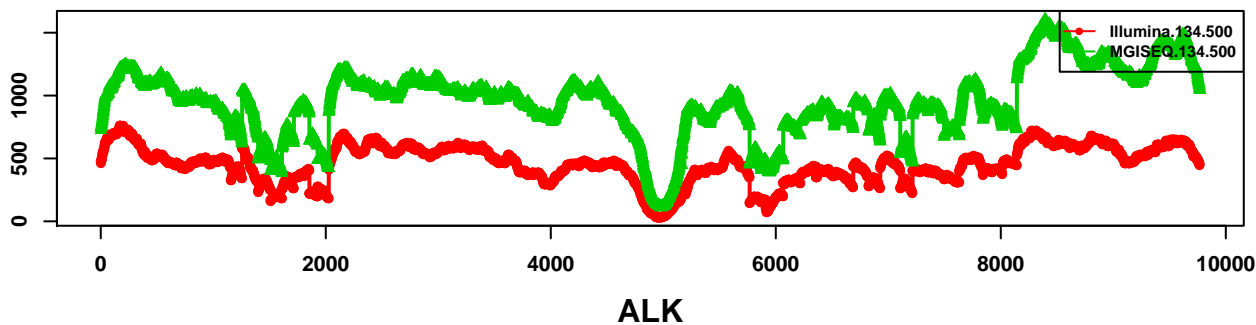

Supplement: Supplementary file 2 [file Presentation1.zip › ALK/19JS48274F.pdf]

Sequencing Depth

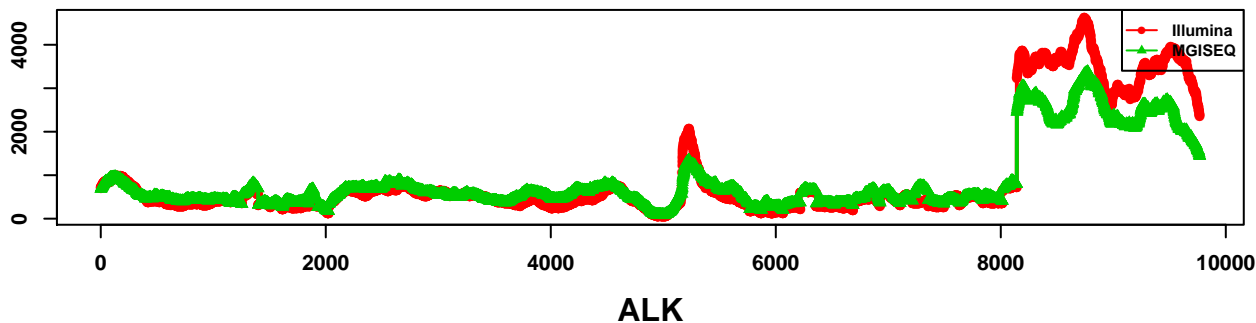

Sequencing Depth

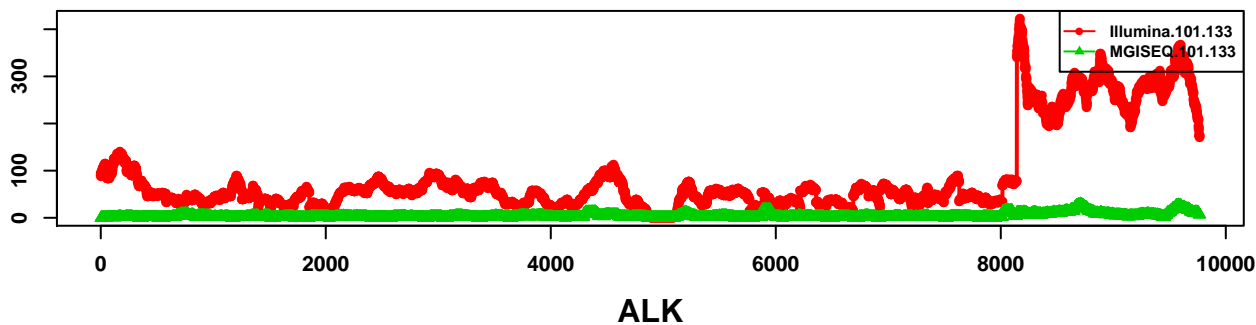

Sequencing Depth

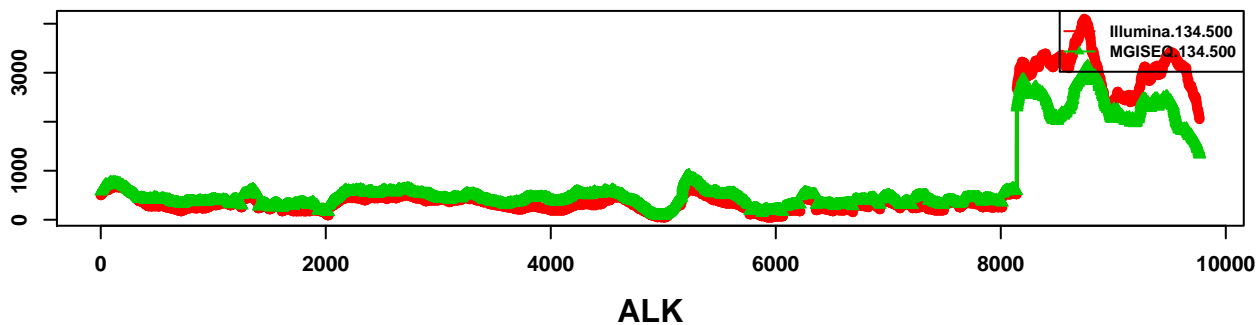

Supplement: Supplementary file 2 [file Presentation1.zip › ALK/19FC40249F.pdf]

Sequencing Depth

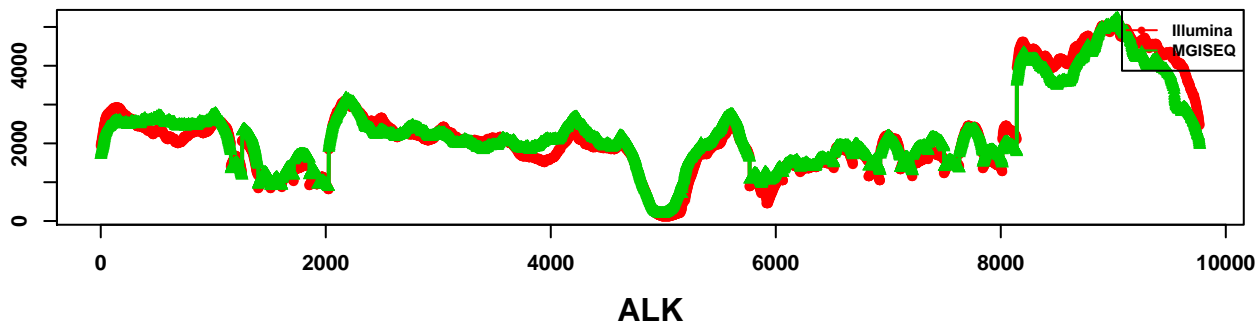

Sequencing Depth

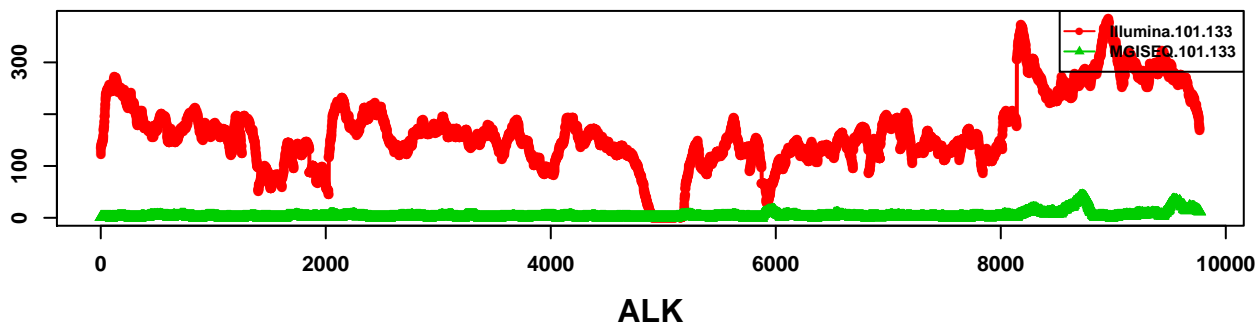

Sequencing Depth

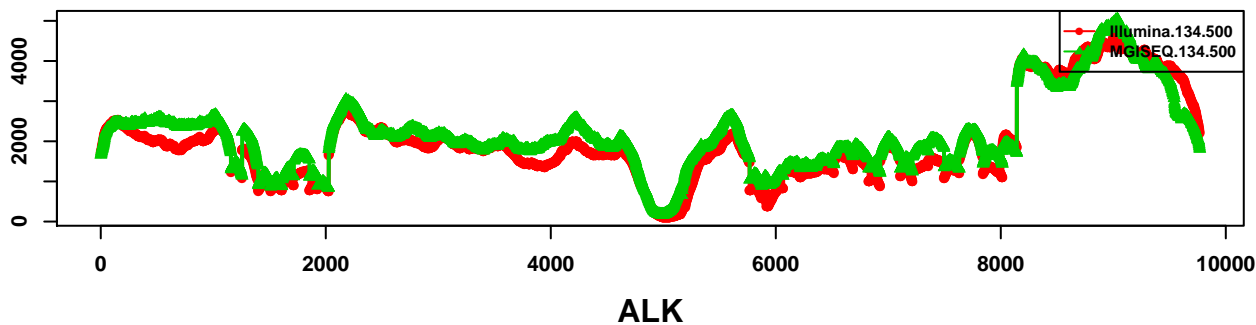

Supplement: Supplementary file 2 [file Presentation1.zip › ALK/19HE22103F.pdf]

Sequencing Depth

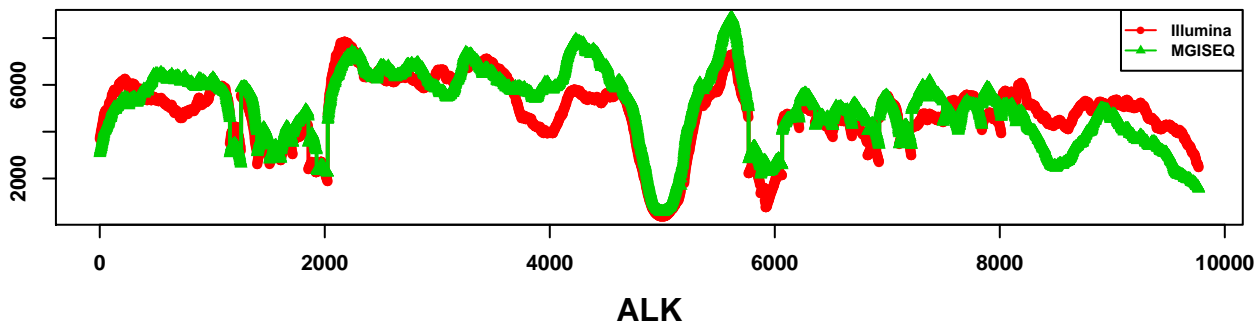

Sequencing Depth

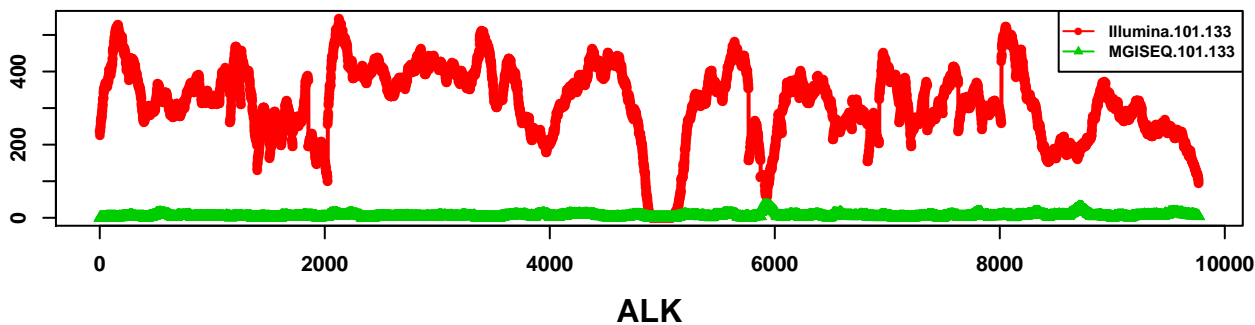

Sequencing Depth

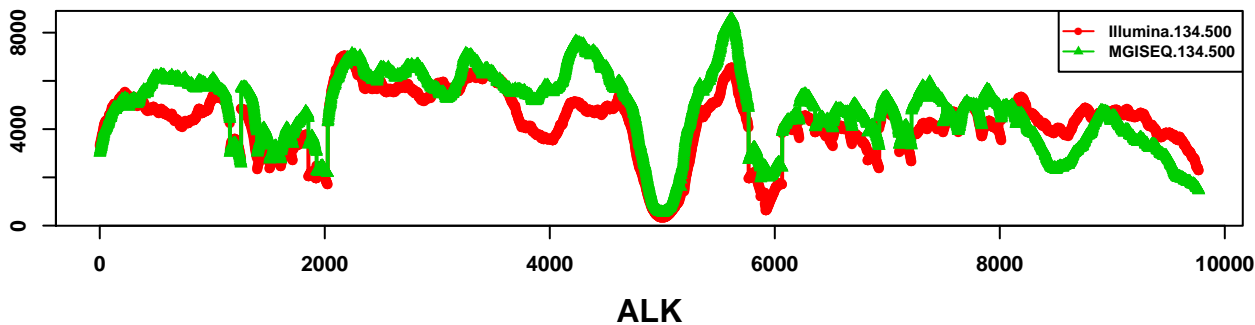

Supplement: Supplementary file 2 [file Presentation1.zip › ALK/19GY94041T.pdf]

Sequencing Depth

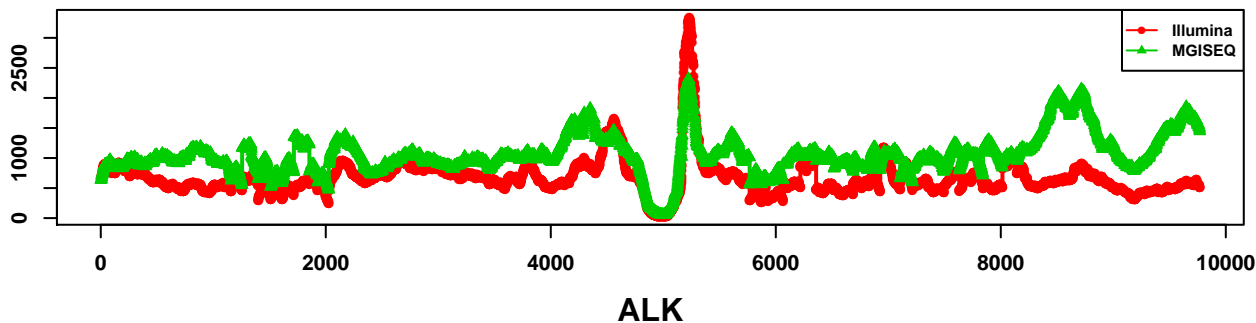

Sequencing Depth

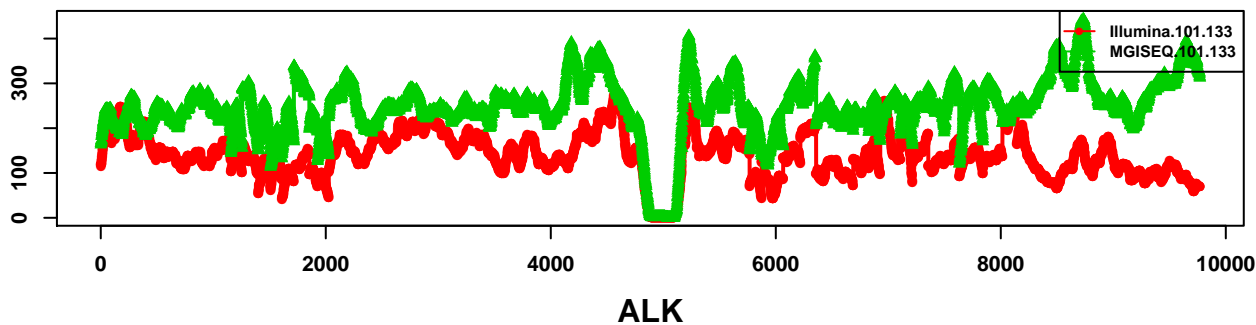

Sequencing Depth

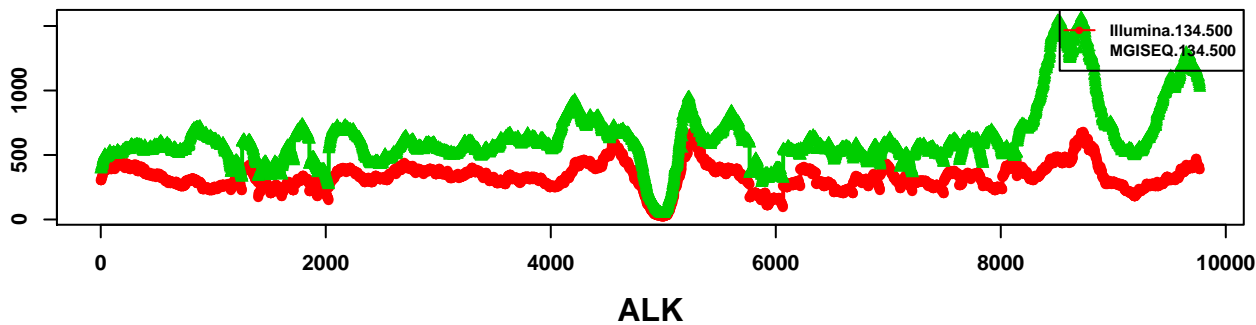

Supplement: Supplementary file 2 [file Presentation1.zip › ALK/19CF15765F.pdf]

Sequencing Depth

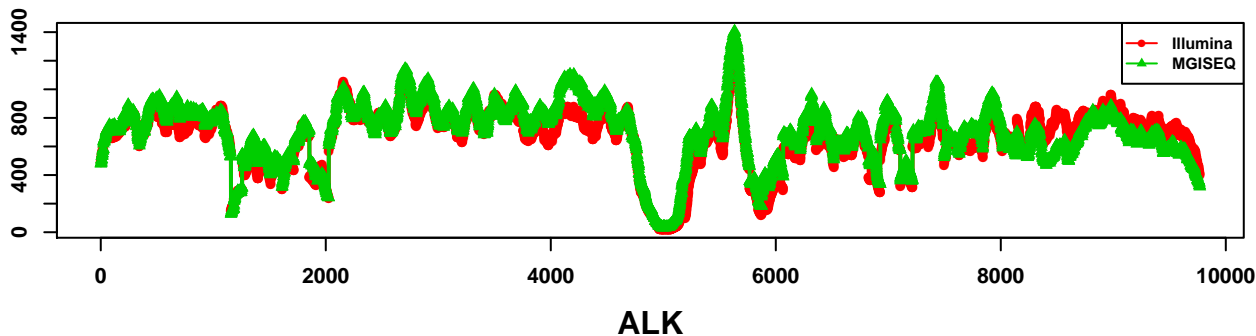

Sequencing Depth

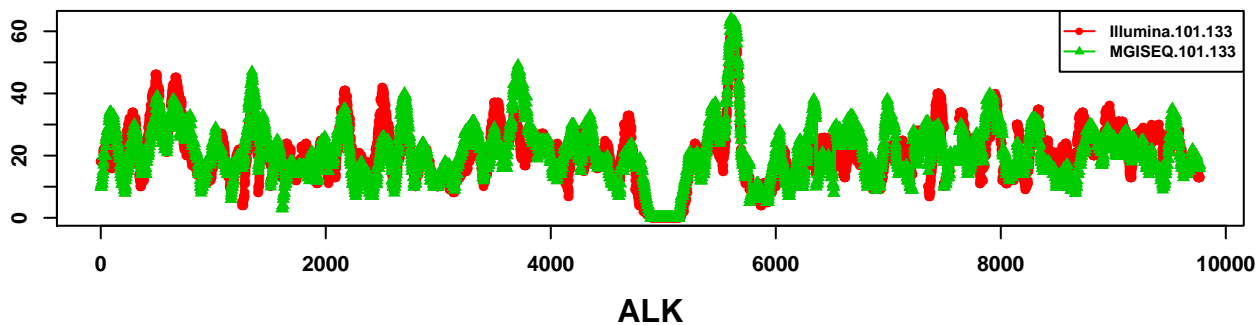

Sequencing Depth

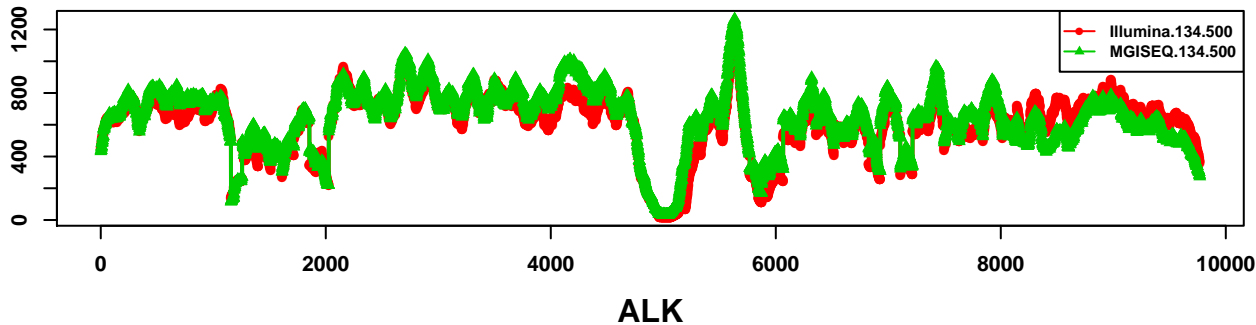

Supplement: Supplementary file 2 [file Presentation1.zip › ALK/M1901246P.pdf]

Sequencing Depth

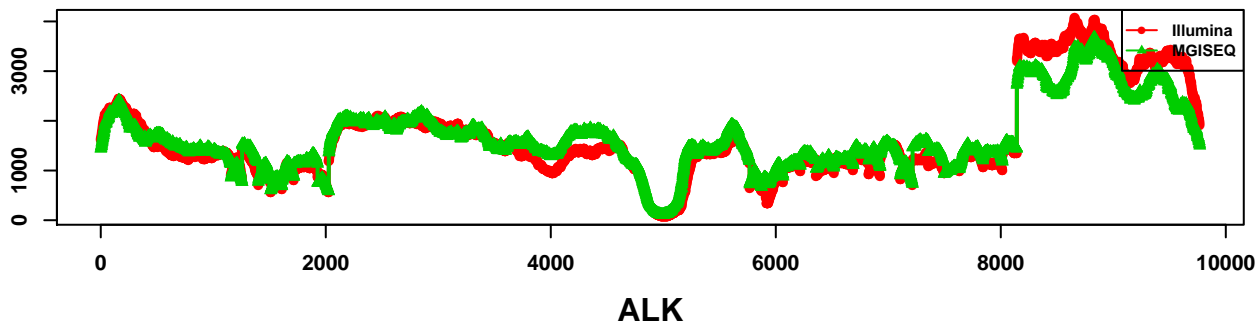

Sequencing Depth

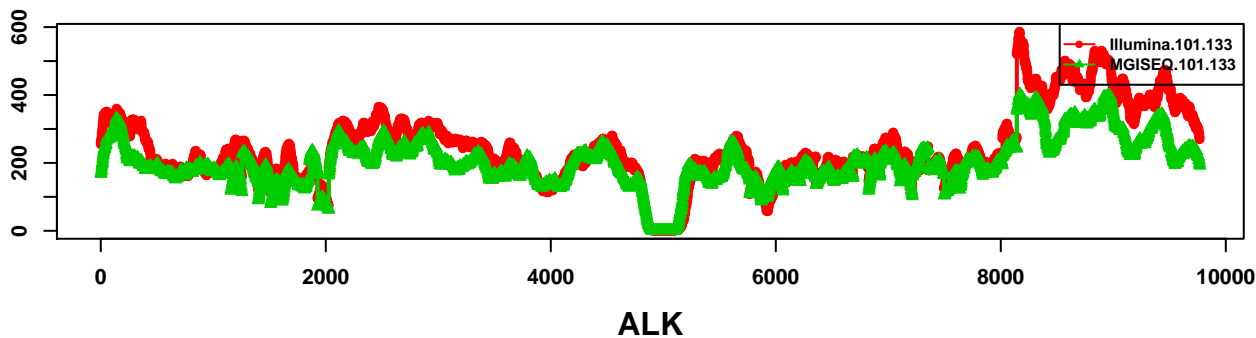

Sequencing Depth

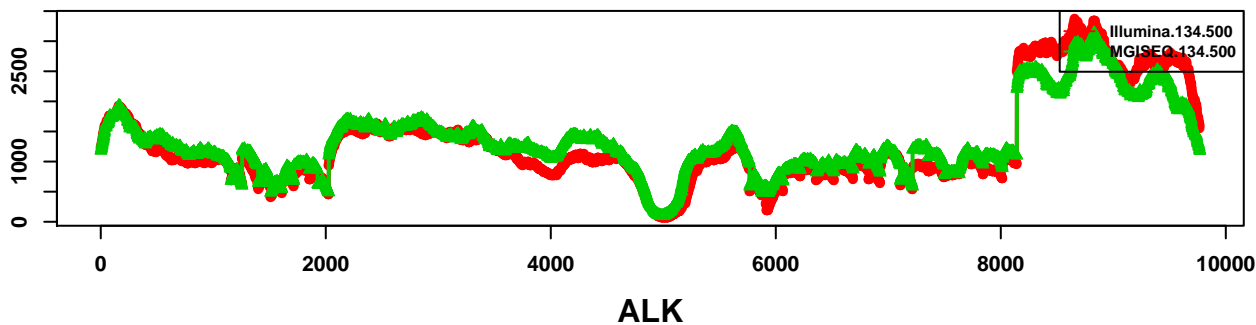

Supplement: Supplementary file 2 [file Presentation1.zip › ALK/19Q06298F.pdf]

Sequencing Depth

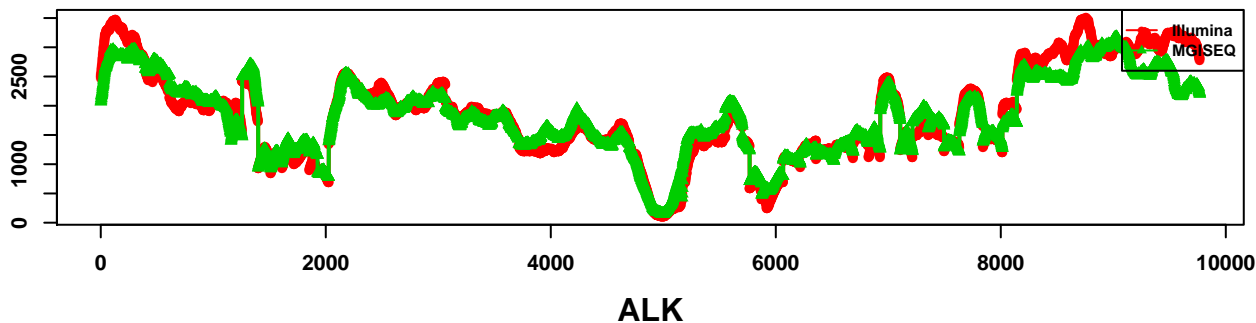

Sequencing Depth

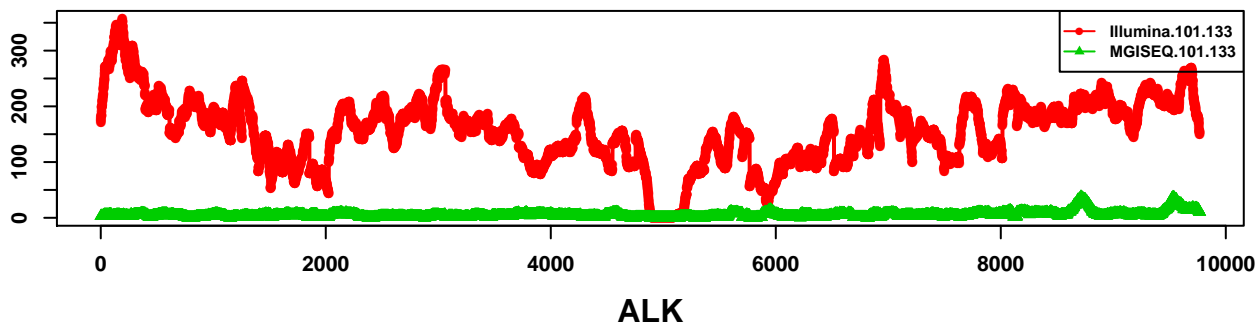

Sequencing Depth

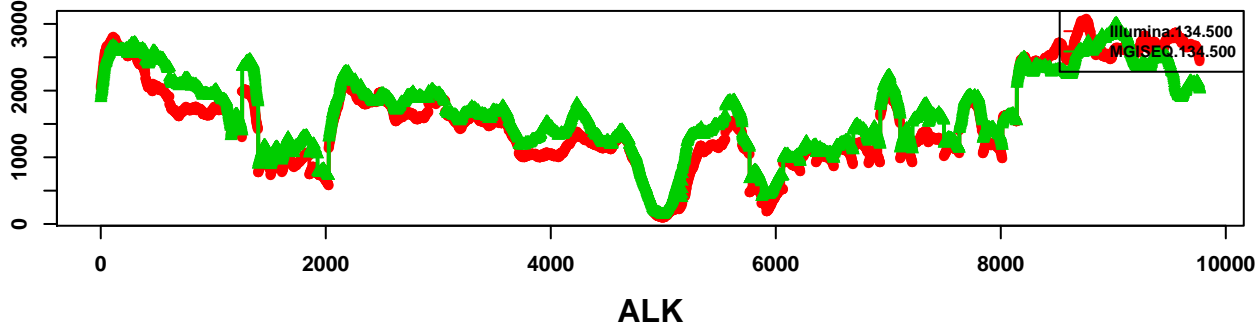

Supplement: Supplementary file 2 [file Presentation1.zip › ALK/19HE22050F.pdf]

Sequencing Depth

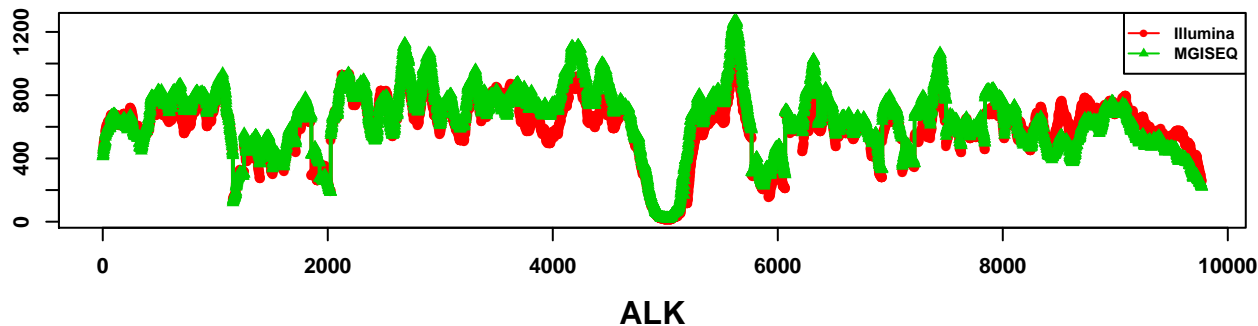

Sequencing Depth

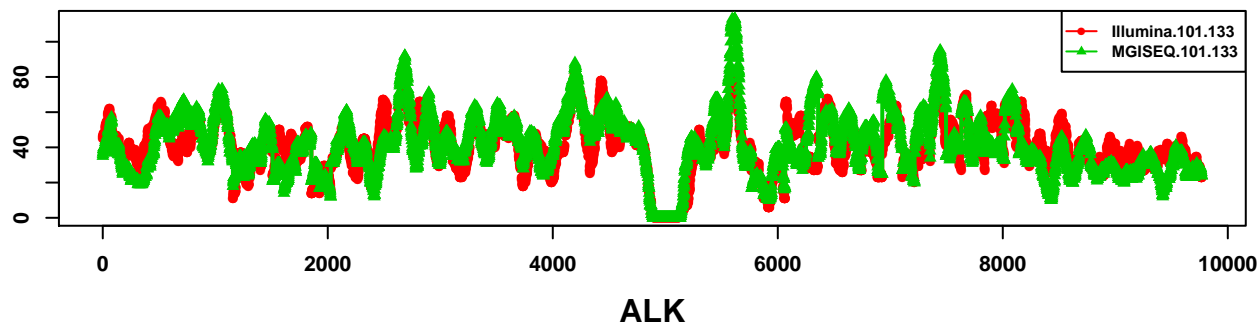

Sequencing Depth

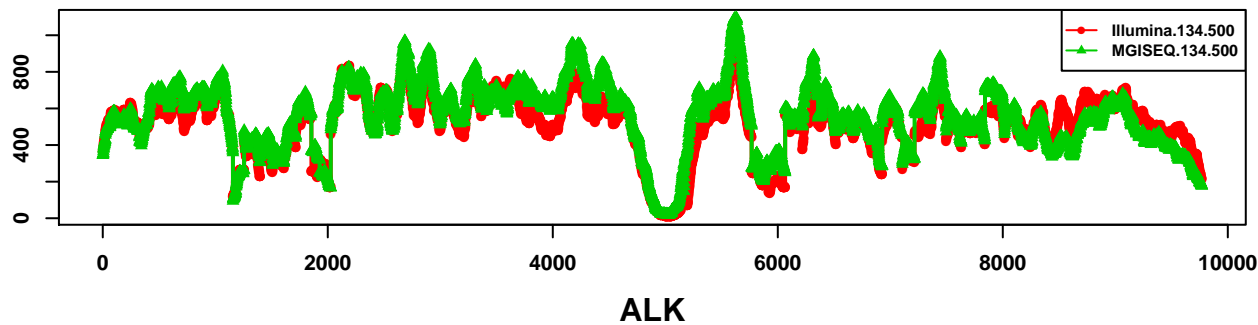

Supplement: Supplementary file 2 [file Presentation1.zip › ALK/ZK190805-CF.pdf]

Sequencing Depth

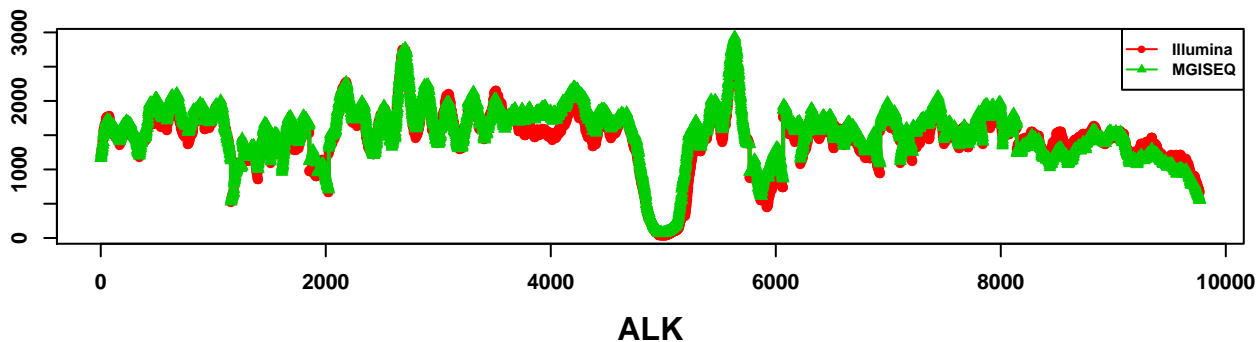

Sequencing Depth

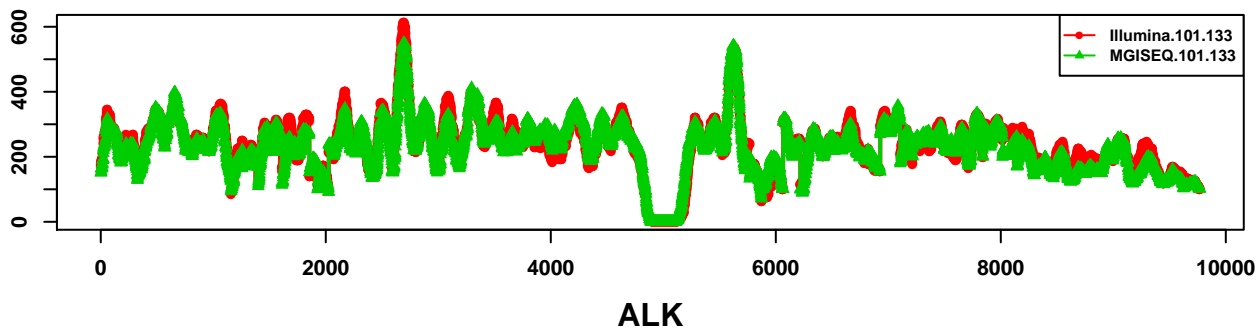

Sequencing Depth

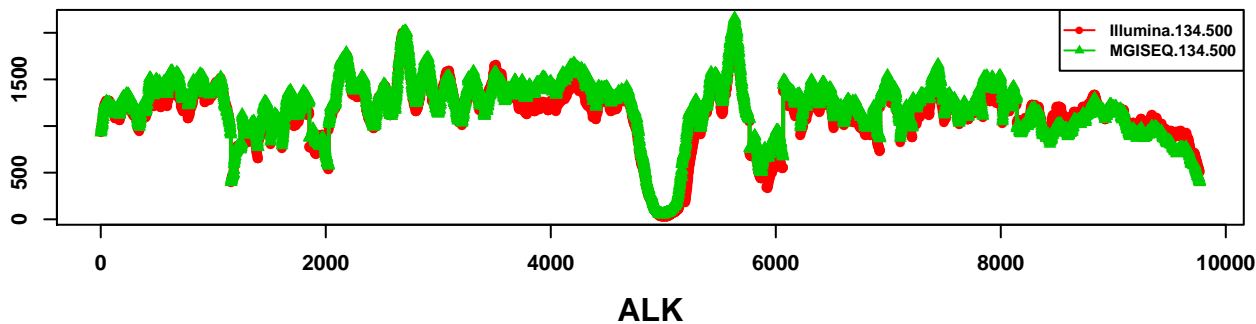

Supplement: Supplementary file 2 [file Presentation1.zip › ALK/19YT53595P.pdf]

Sequencing Depth

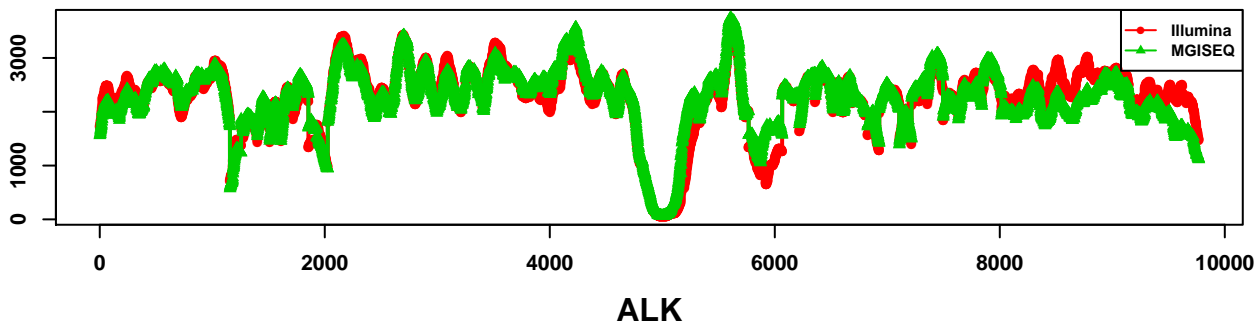

Sequencing Depth

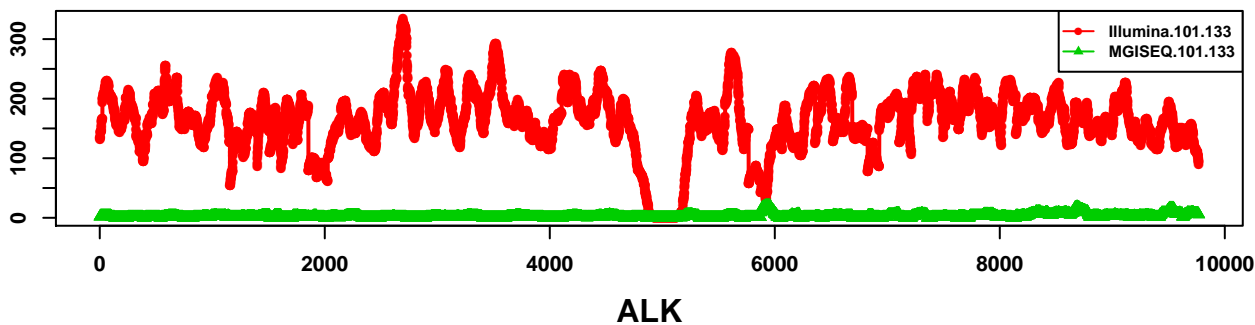

Sequencing Depth

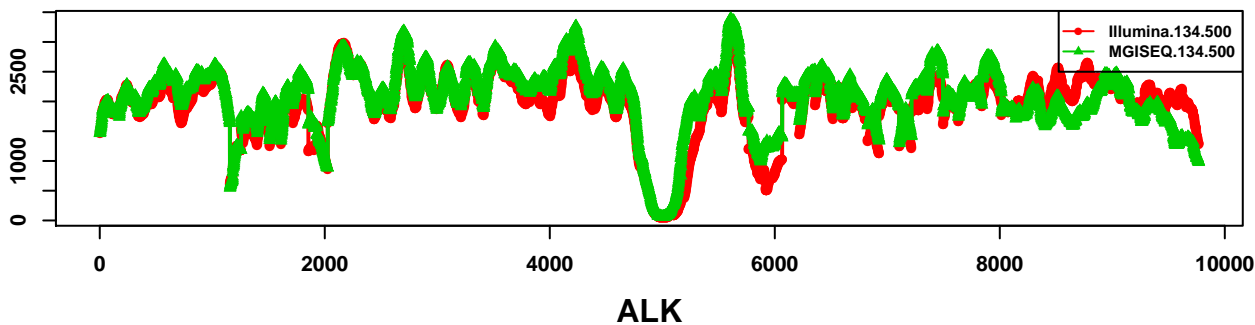

Supplement: Supplementary file 3 [file Presentation2.zip › ALK/19ZN13103P.pdf]

Sequencing Depth

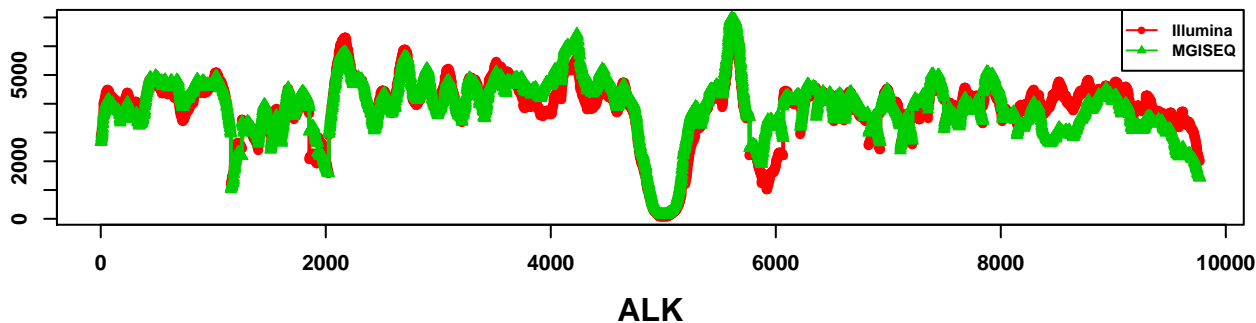

Sequencing Depth

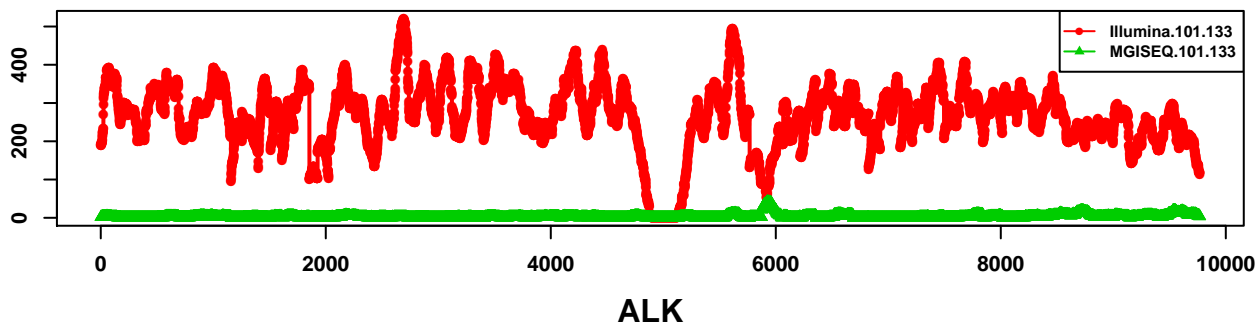

Sequencing Depth

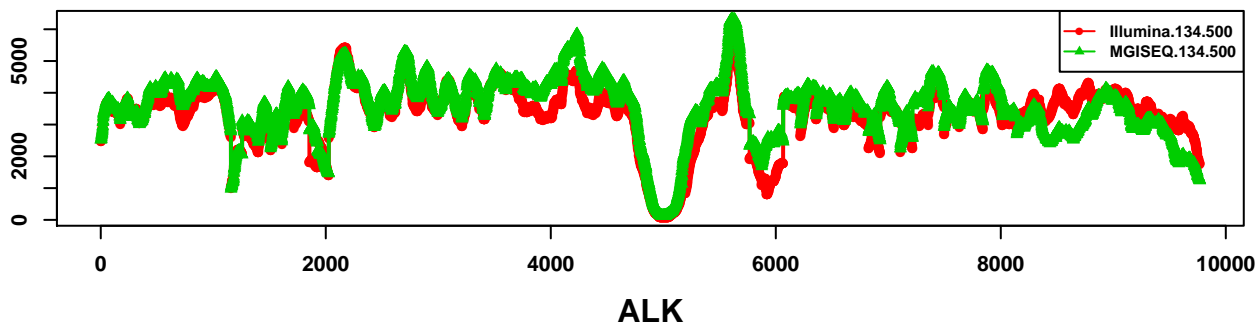

Supplement: Supplementary file 3 [file Presentation2.zip › ALK/19N01653P.pdf]

Sequencing Depth

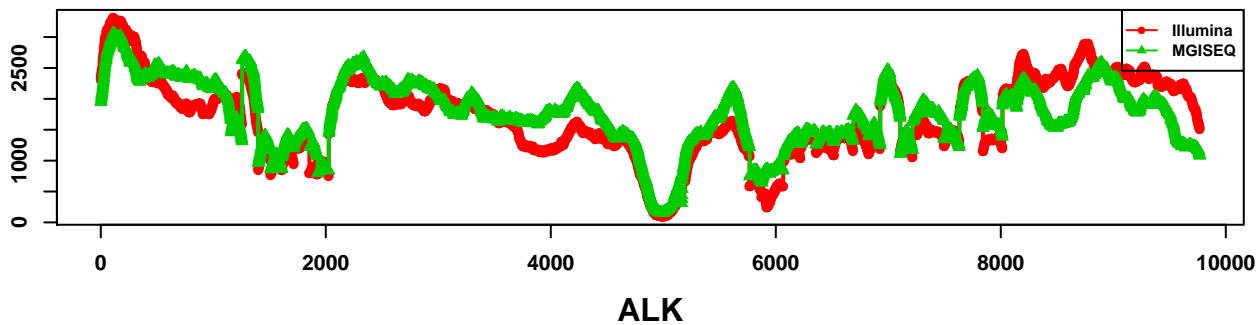

Sequencing Depth

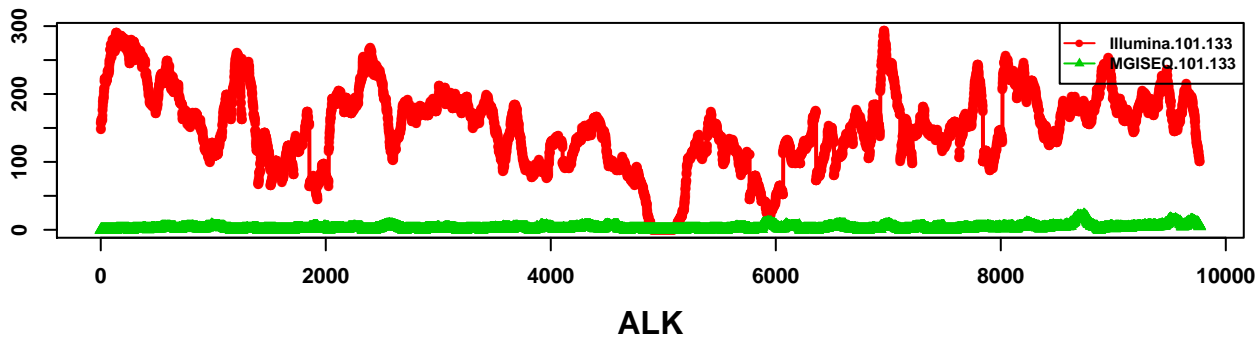

Sequencing Depth

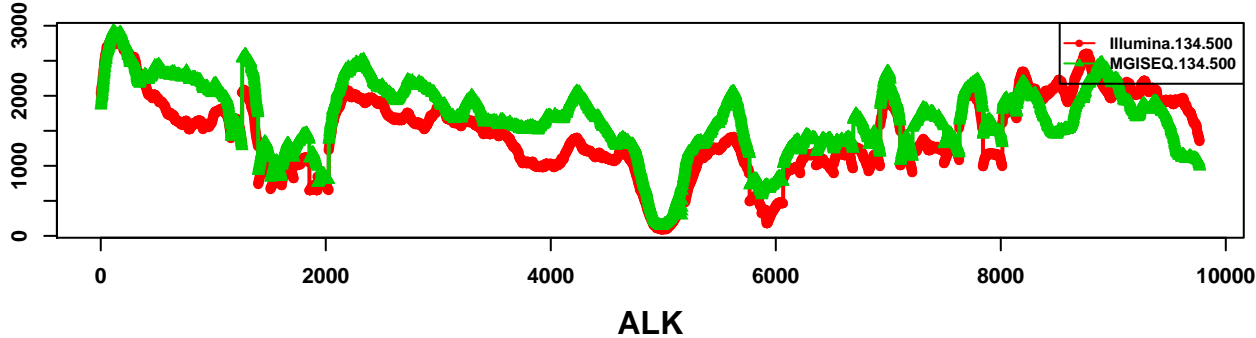

Supplement: Supplementary file 3 [file Presentation2.zip › ALK/19HE21980F.pdf]

Sequencing Depth

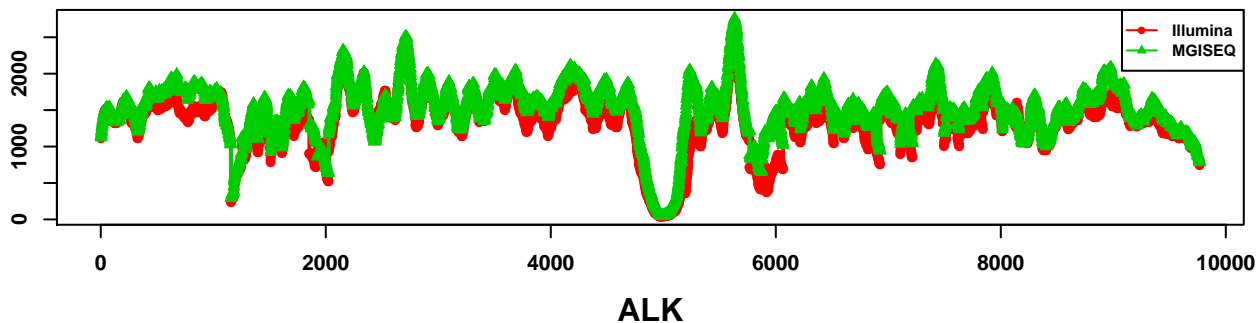

Sequencing Depth

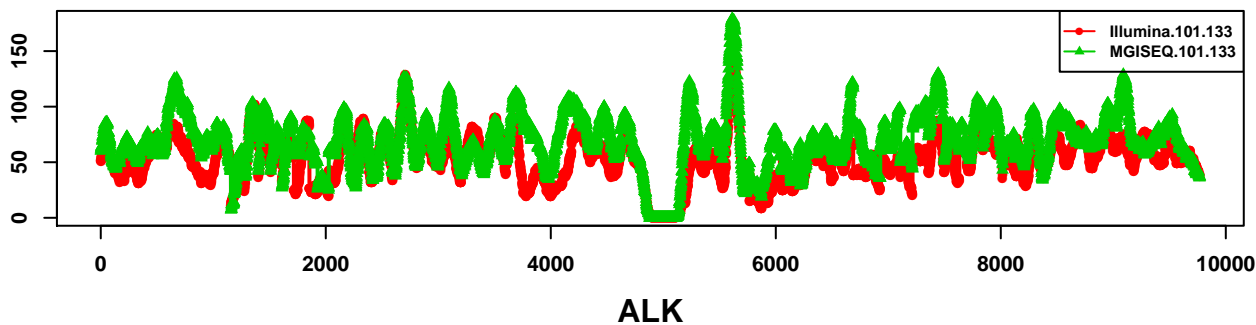

Sequencing Depth

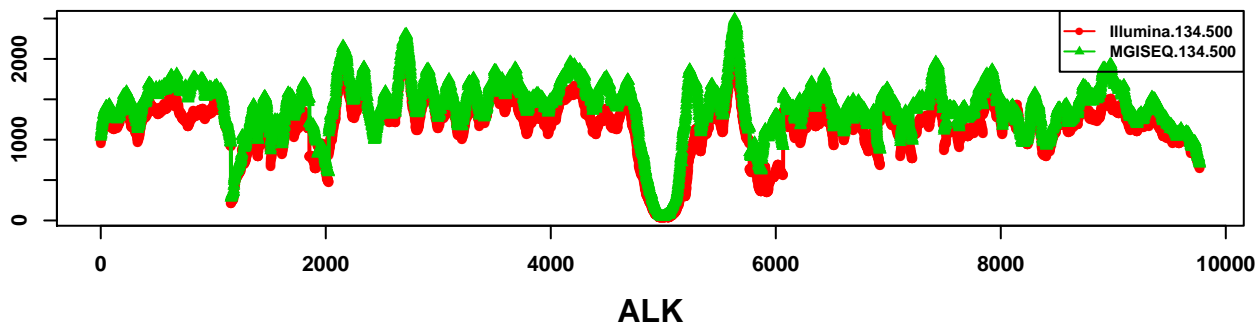

Supplement: Supplementary file 3 [file Presentation2.zip › ALK/FZ19-04447P.pdf]

Sequencing Depth

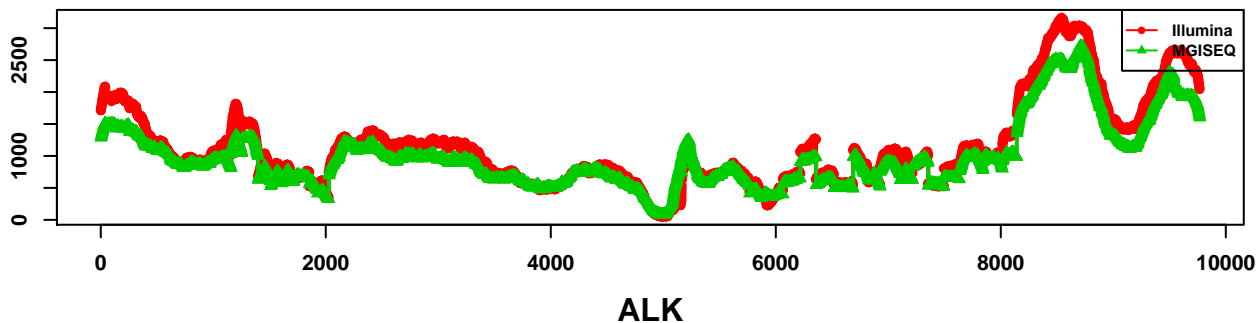

Sequencing Depth

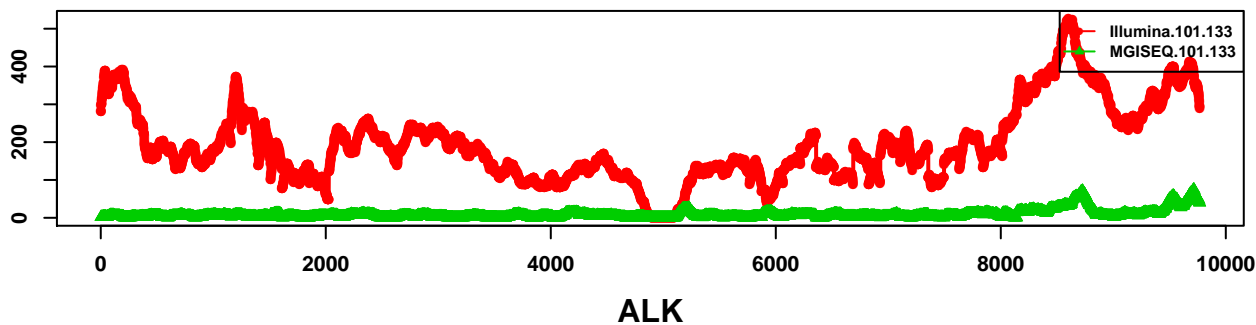

Sequencing Depth

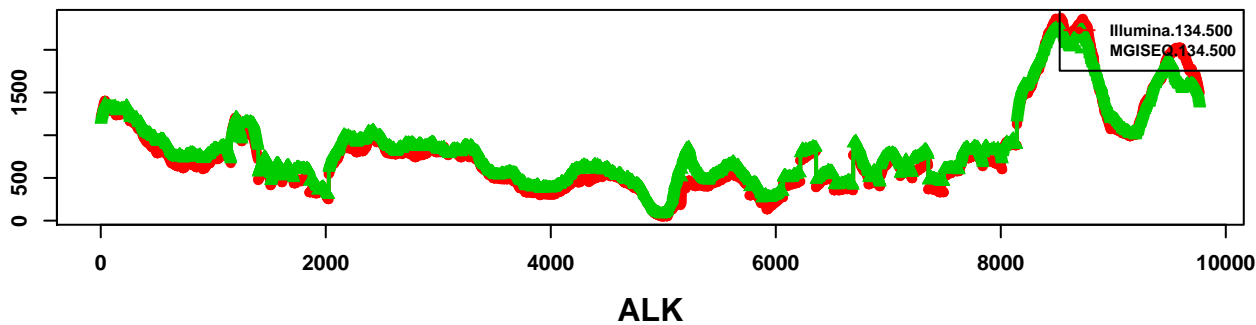

Supplement: Supplementary file 3 [file Presentation2.zip › ALK/19N01686F.pdf]

Sequencing Depth

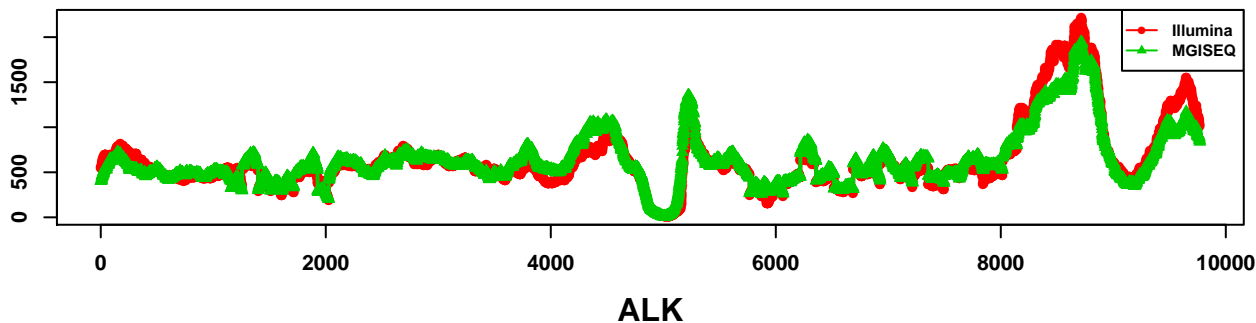

Sequencing Depth

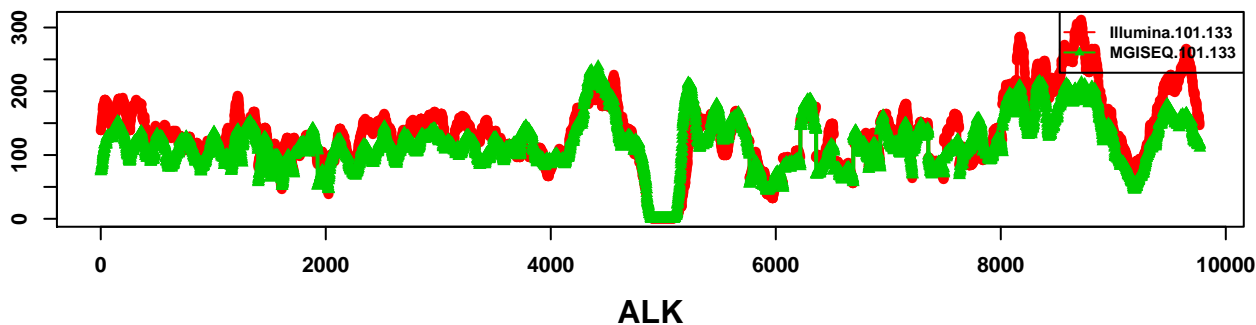

Sequencing Depth

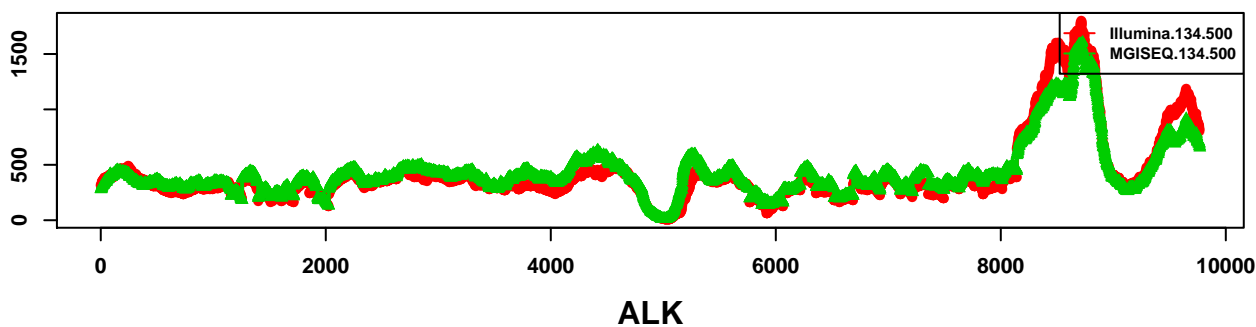

Supplement: Supplementary file 3 [file Presentation2.zip › ALK/19HS86145F.pdf]

Sequencing Depth

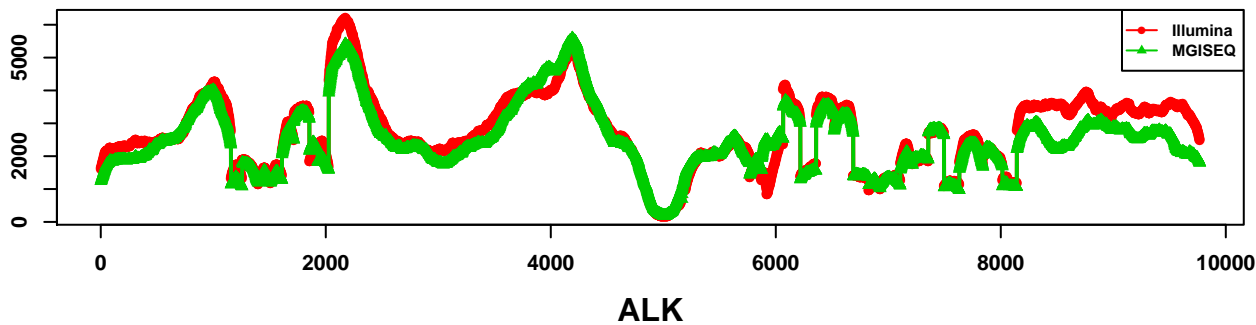

Sequencing Depth

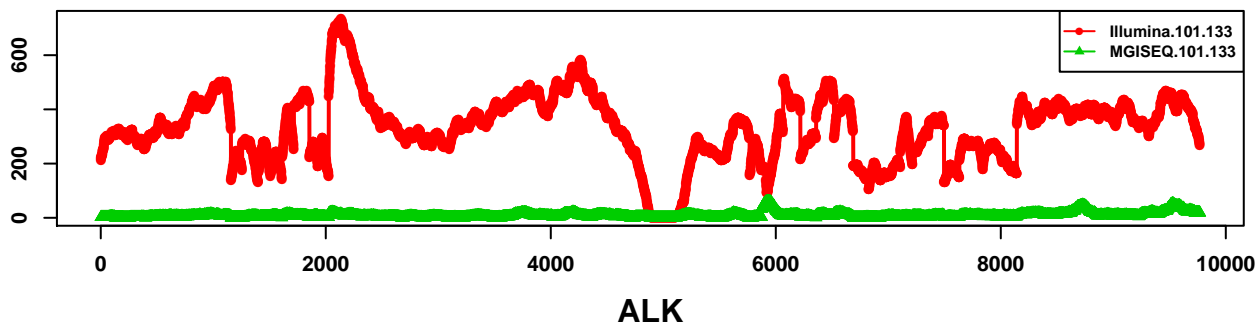

Sequencing Depth

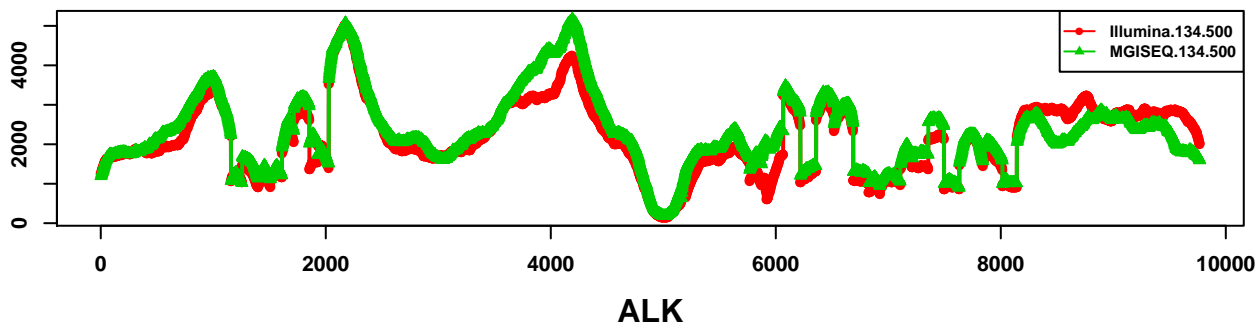

Supplement: Supplementary file 3 [file Presentation2.zip › ALK/19WN60001F.pdf]

Sequencing Depth

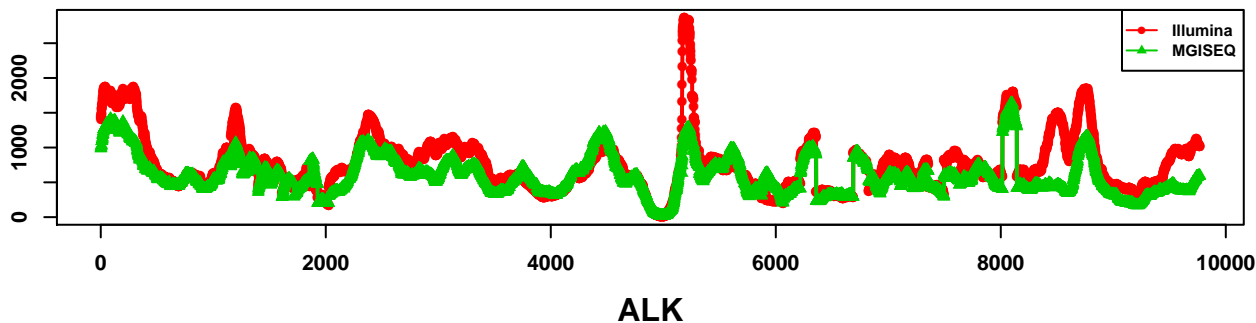

Sequencing Depth

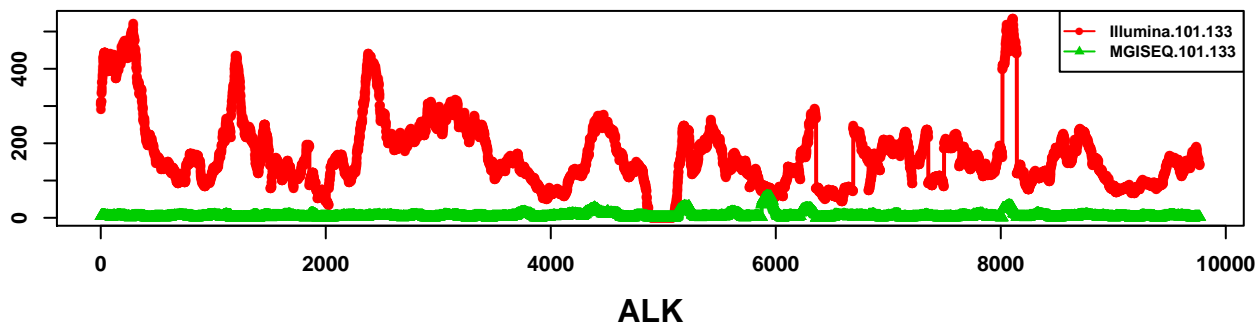

Sequencing Depth

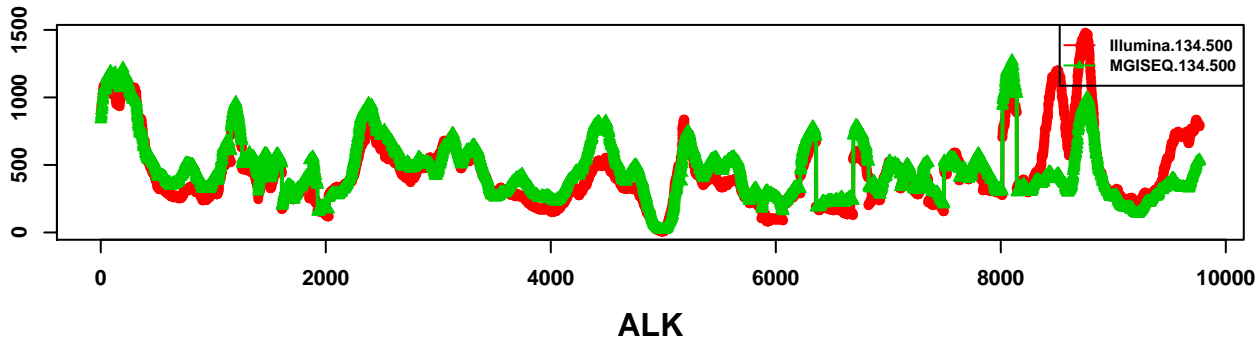

Supplement: Supplementary file 3 [file Presentation2.zip › ALK/19ZN13098F.pdf]

Sequencing Depth

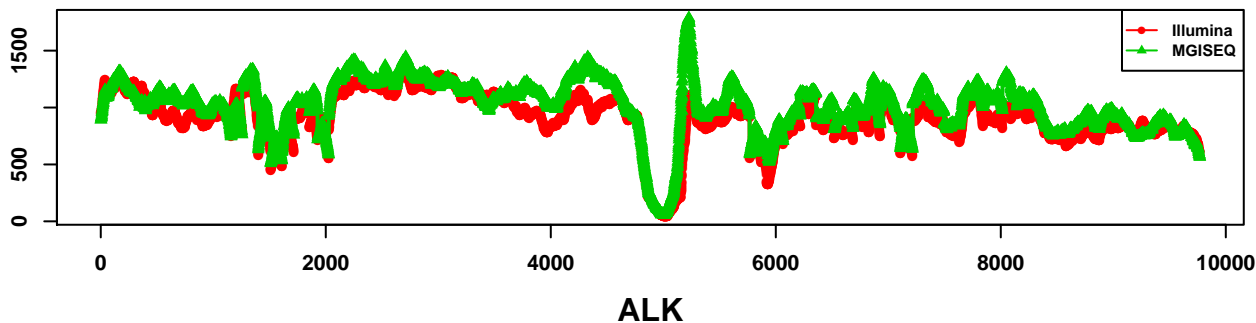

Sequencing Depth

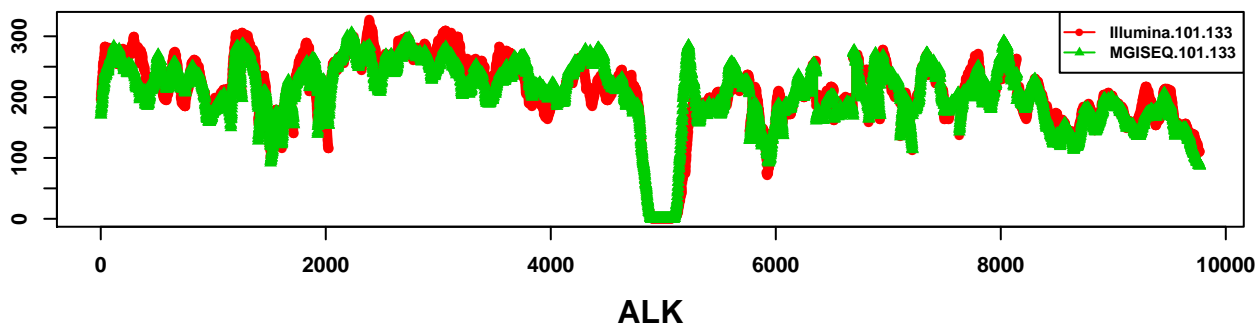

Sequencing Depth

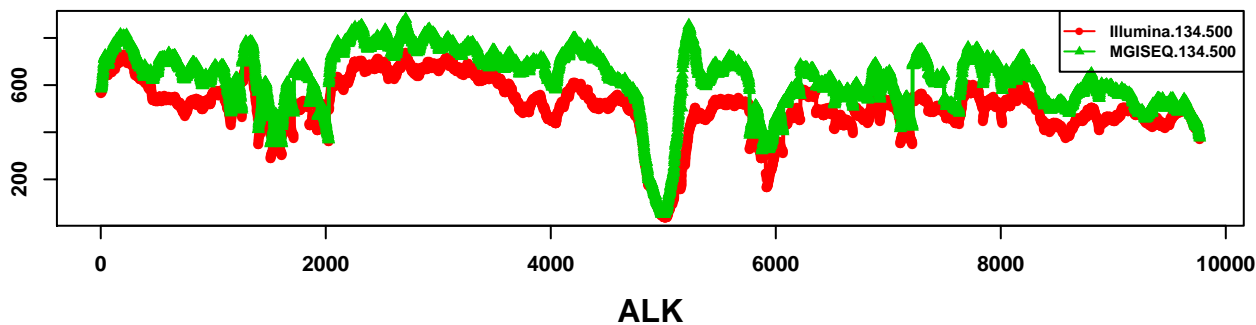

Supplement: Supplementary file 3 [file Presentation2.zip › ALK/19N02330F.pdf]

Sequencing Depth

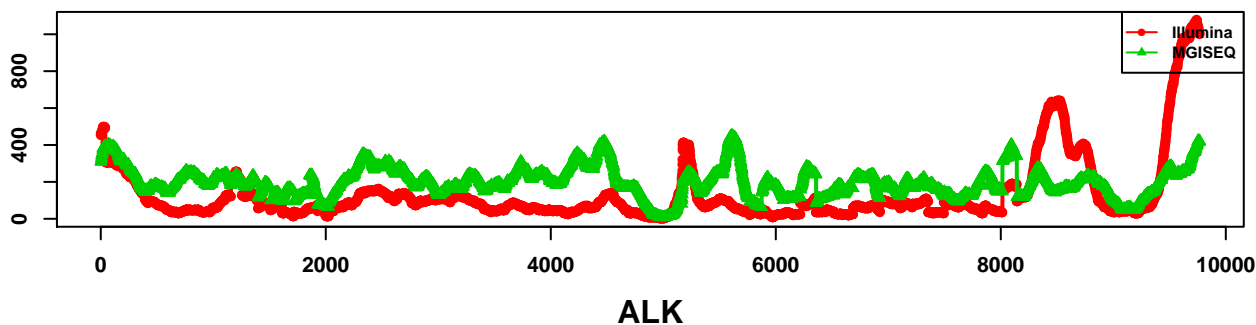

Sequencing Depth

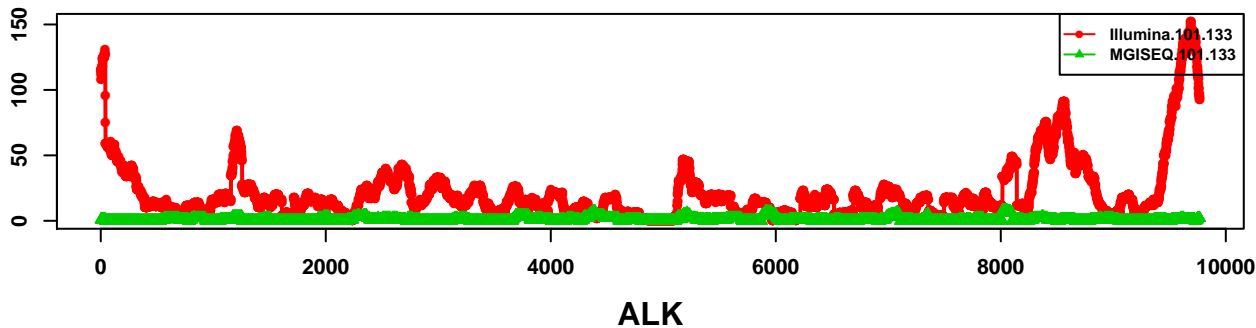

Sequencing Depth

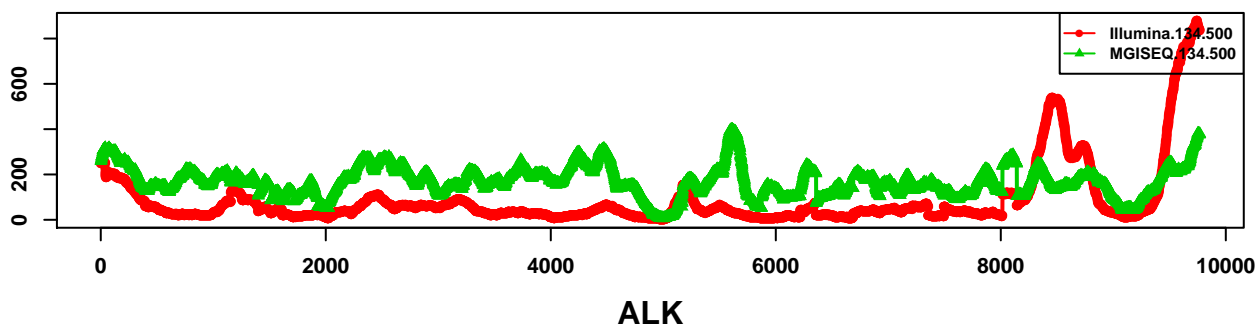

Supplement: Supplementary file 3 [file Presentation2.zip › ALK/19ZN12365F.pdf]

Sequencing Depth

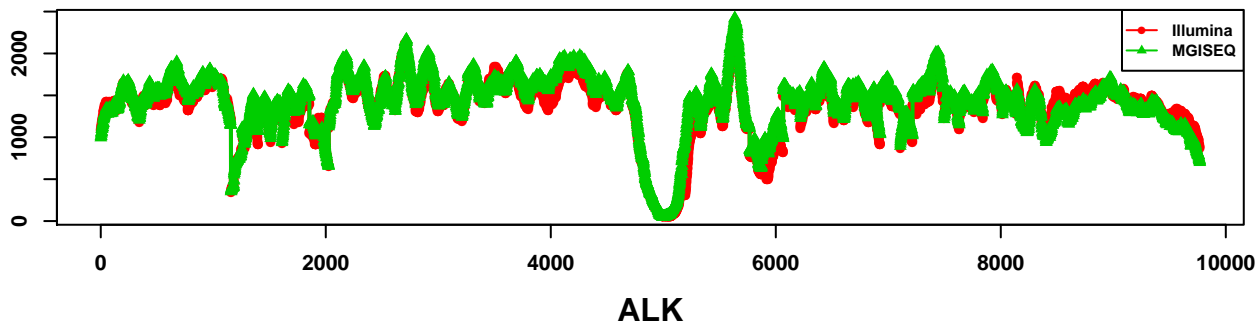

Sequencing Depth

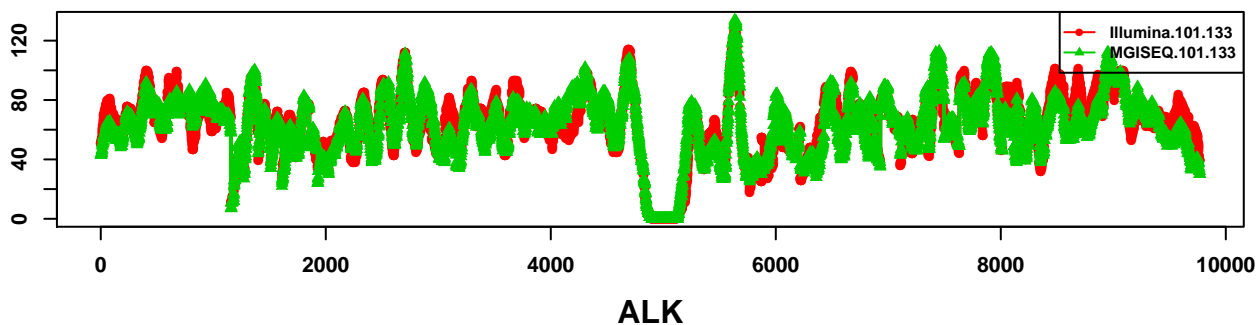

Sequencing Depth

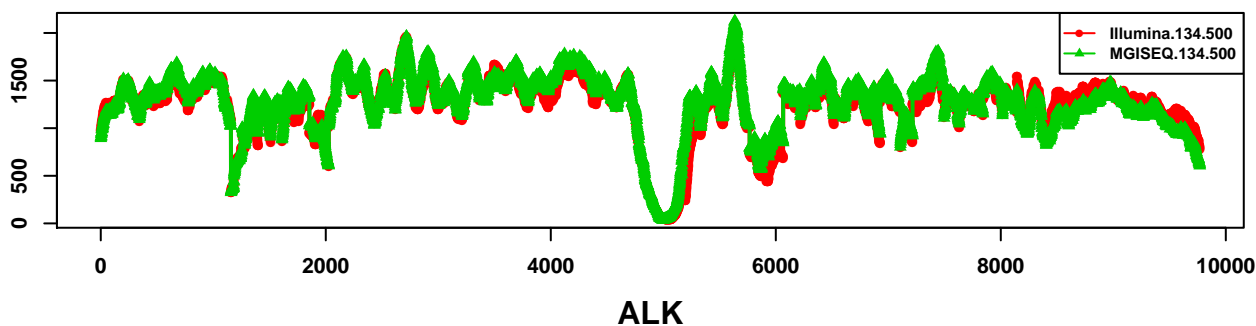

Supplement: Supplementary file 3 [file Presentation2.zip › ALK/19ZN13489P.pdf]

Sequencing Depth

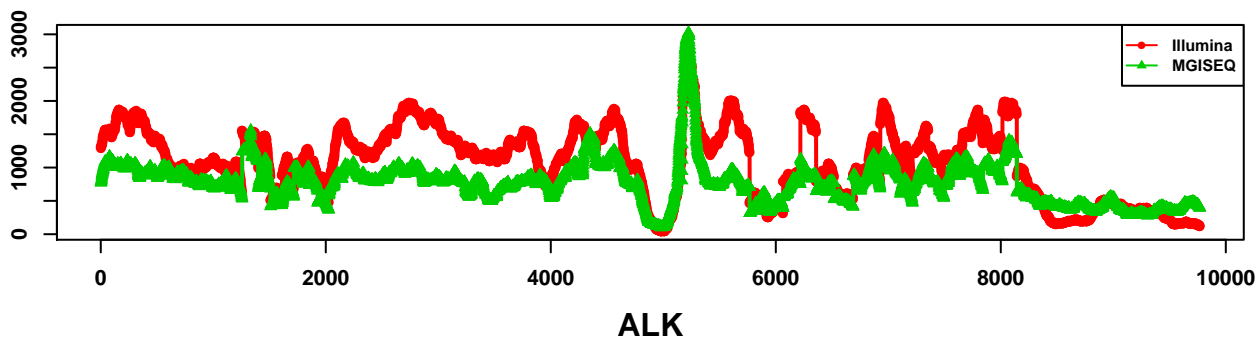

Sequencing Depth

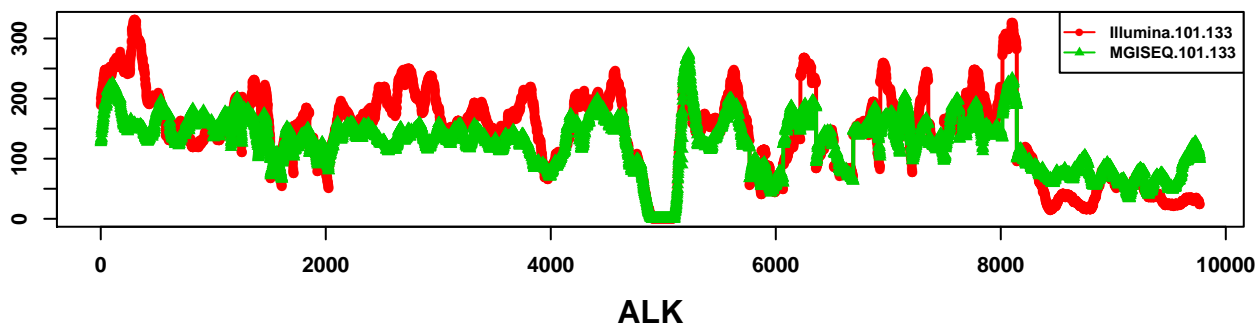

Sequencing Depth

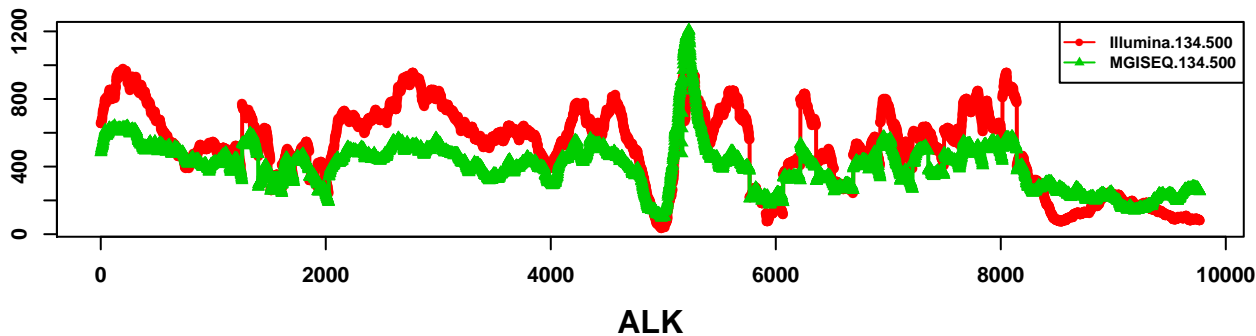

Supplement: Supplementary file 3 [file Presentation2.zip › ALK/19ZN12575F.pdf]

Sequencing Depth

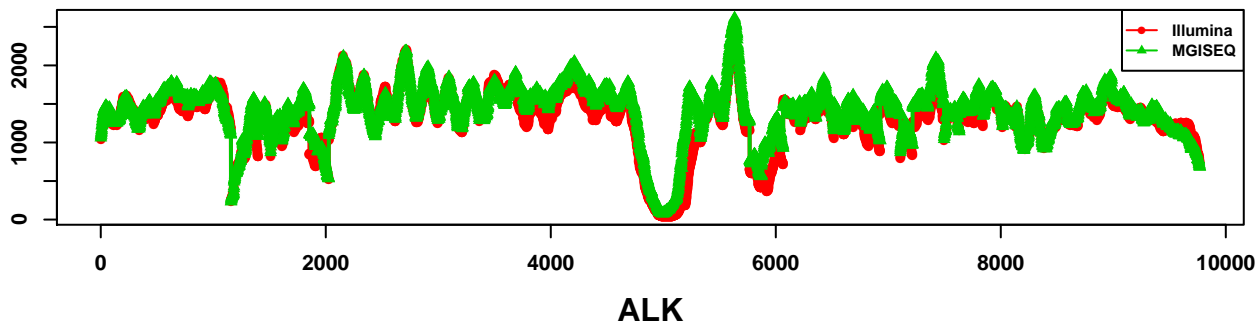

Sequencing Depth

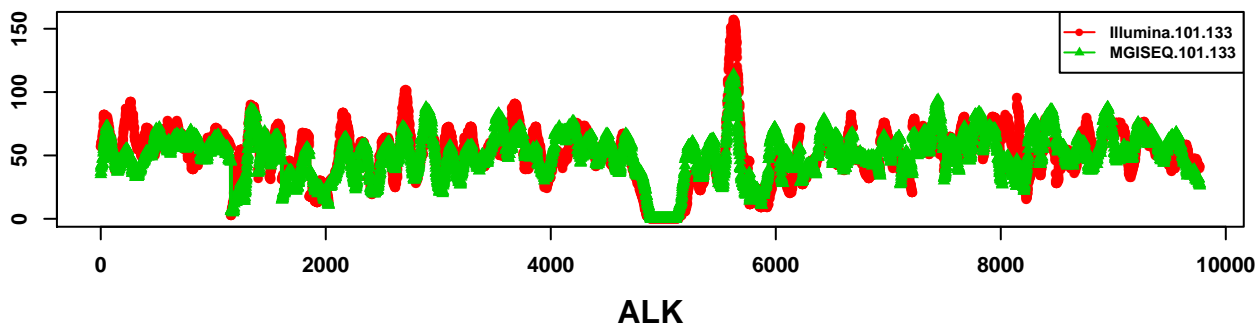

Sequencing Depth

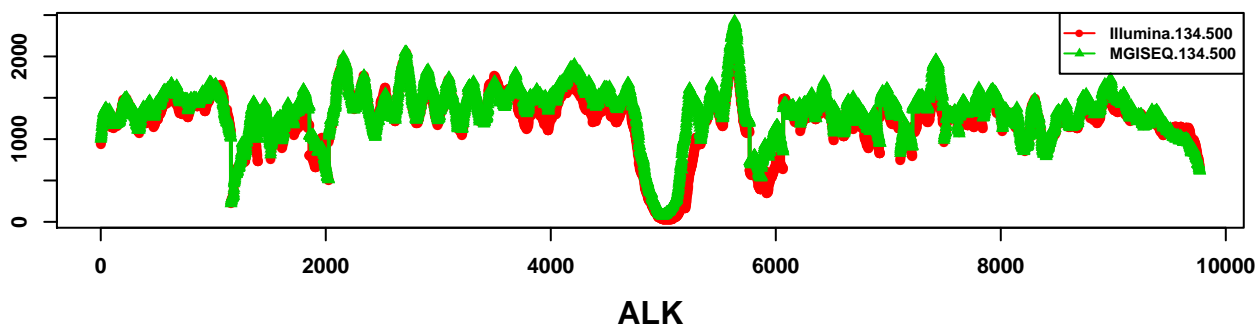

Supplement: Supplementary file 3 [file Presentation2.zip › ALK/19JS48265P.pdf]
