# Supplementary figures and images for: Evaluation of the MGISEQ-2000 Sequencing Platform for Illumina Target Capture Sequencing Libraries (part 2 of 6)
Source: Front Genet. 2021 Oct 27;12:730519. doi: 10.3389/fgene.2021.730519 (PMC8578046; doi:10.3389/fgene.2021.730519)

Sequencing Depth

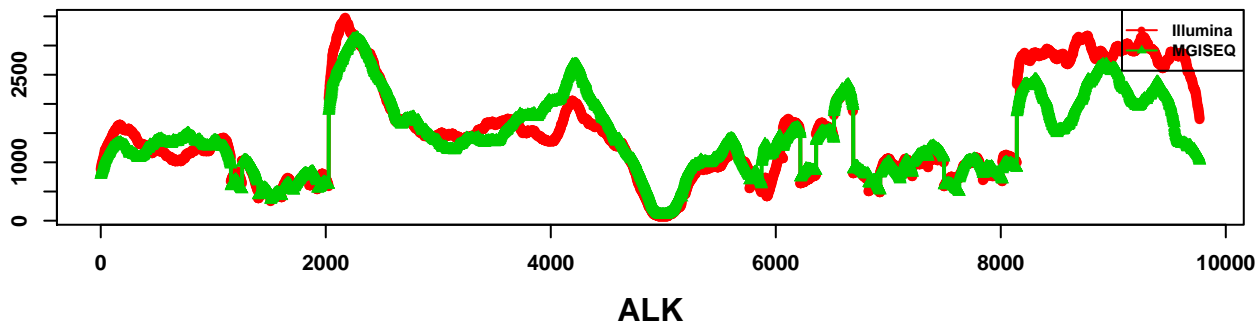

Sequencing Depth

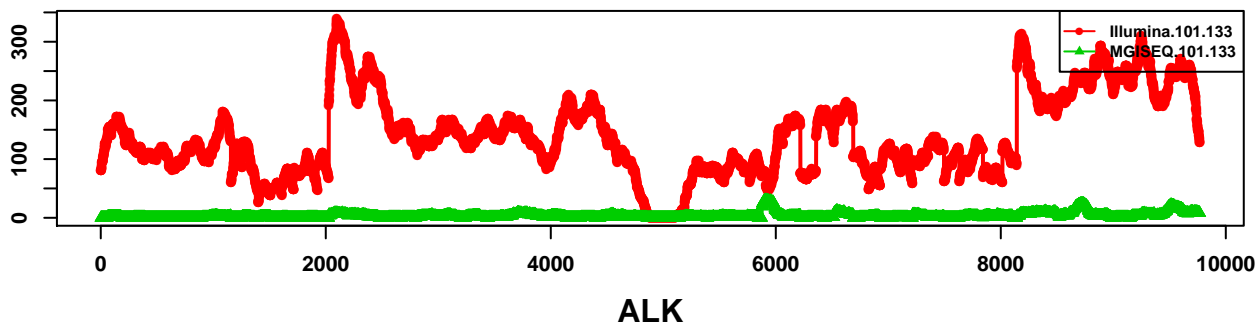

Sequencing Depth

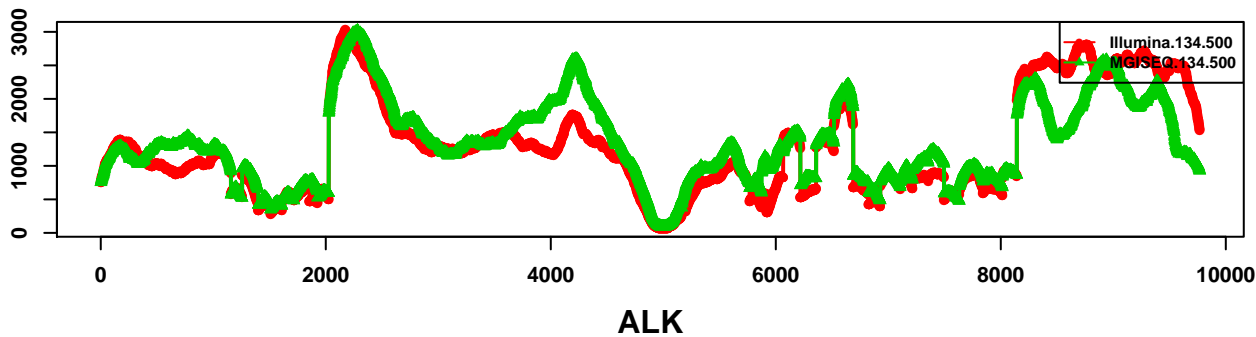

Supplement: Supplementary file 3 [file Presentation2.zip › ALK/19N01658F.pdf]

Sequencing Depth

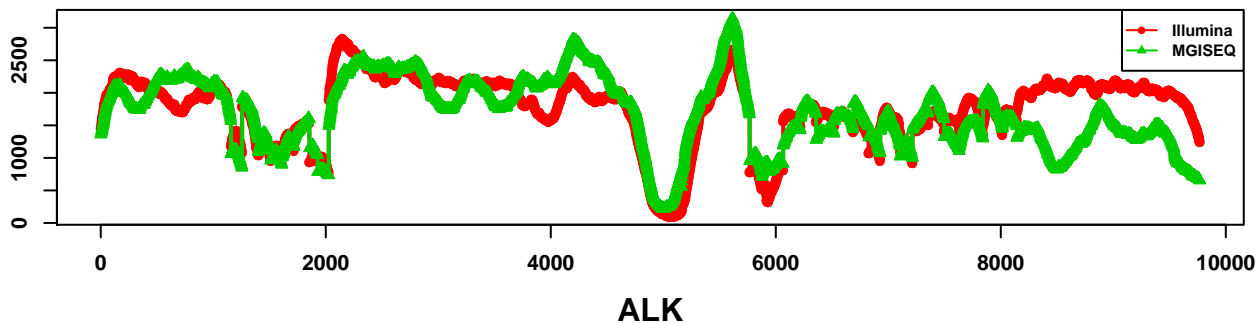

Sequencing Depth

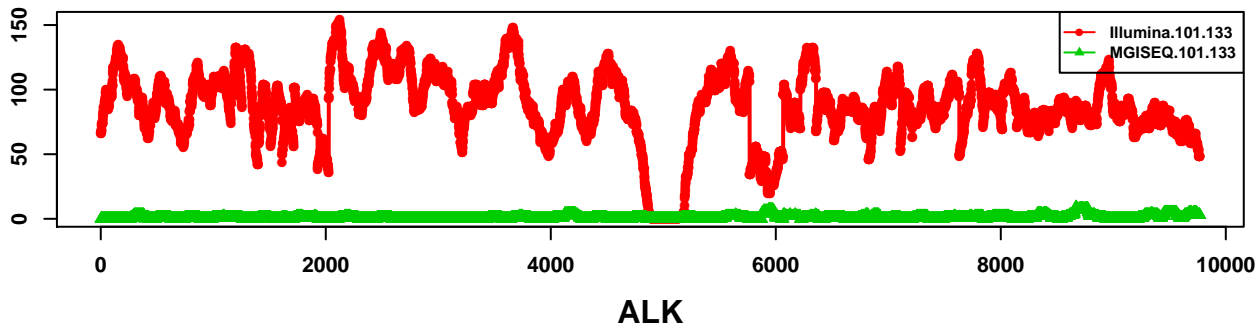

Sequencing Depth

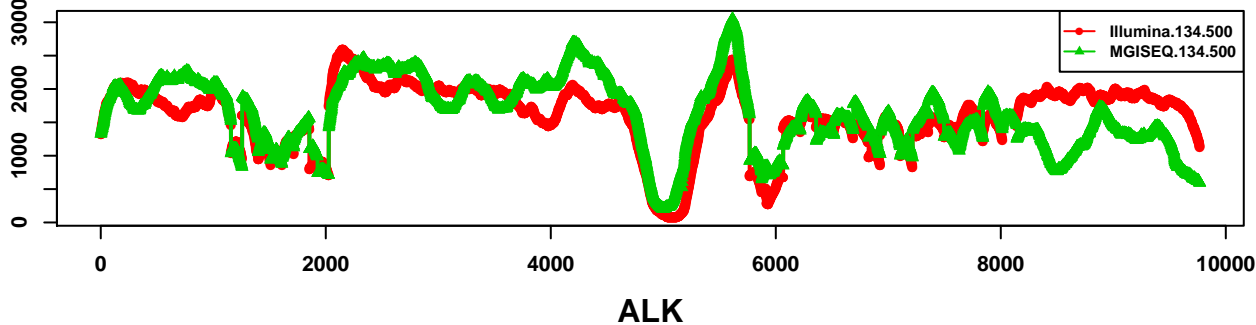

Supplement: Supplementary file 3 [file Presentation2.zip › ALK/ZK0603-G.pdf]

Sequencing Depth

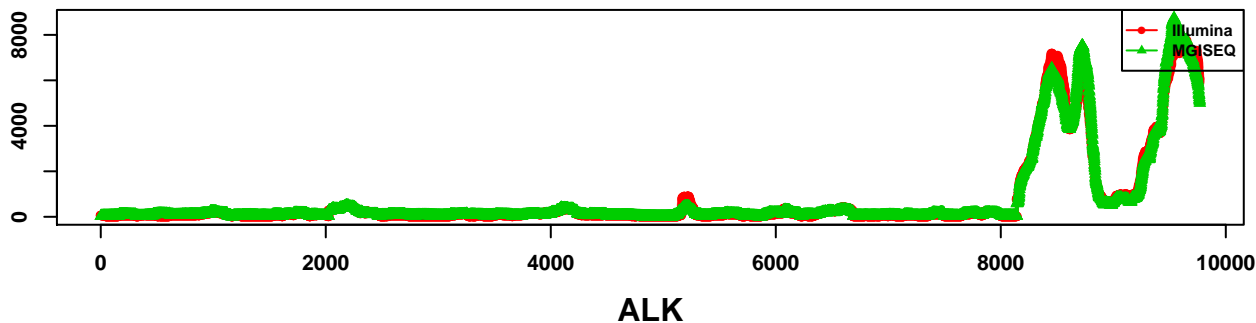

Sequencing Depth

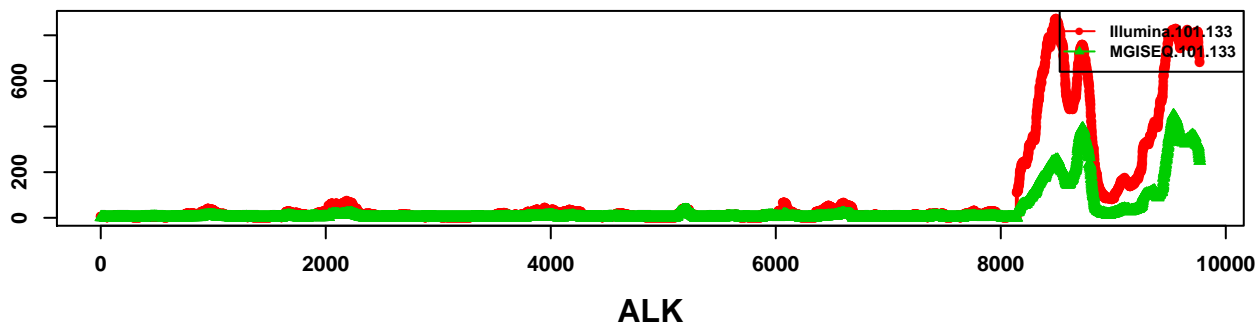

Sequencing Depth

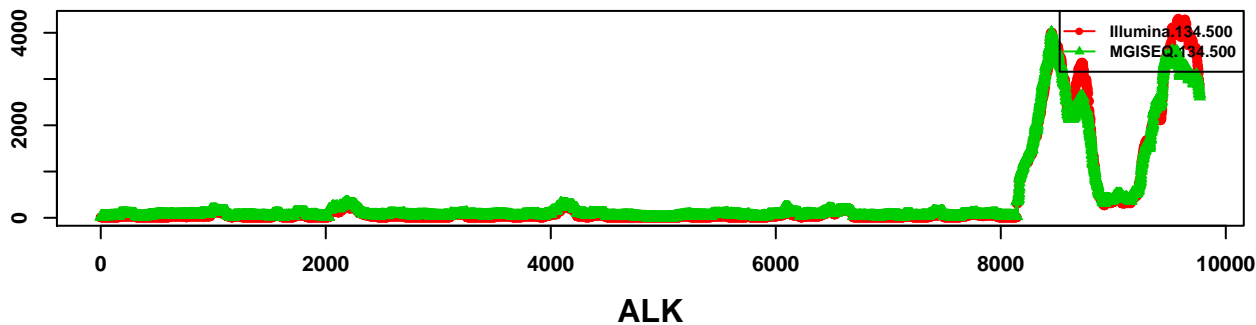

Supplement: Supplementary file 3 [file Presentation2.zip › ALK/19ZN13099T.pdf]

Sequencing Depth

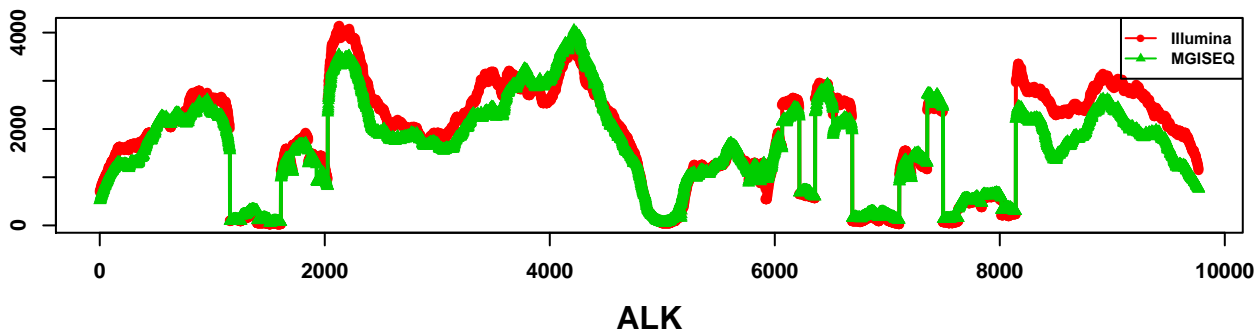

Sequencing Depth

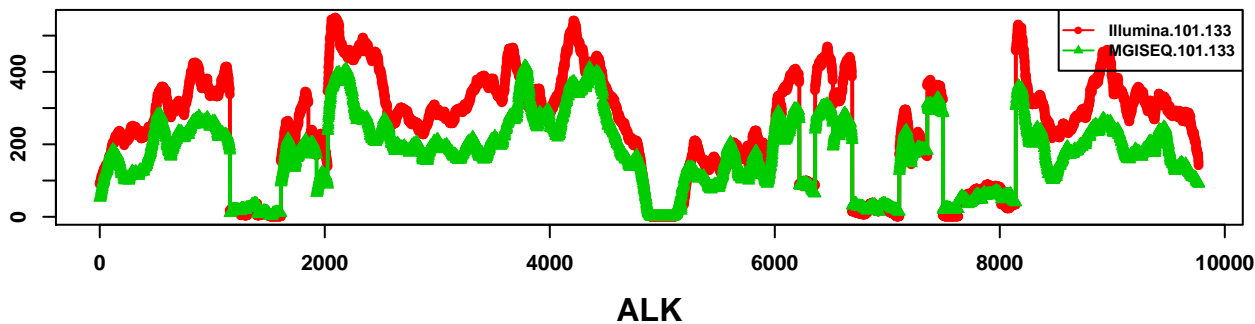

Sequencing Depth

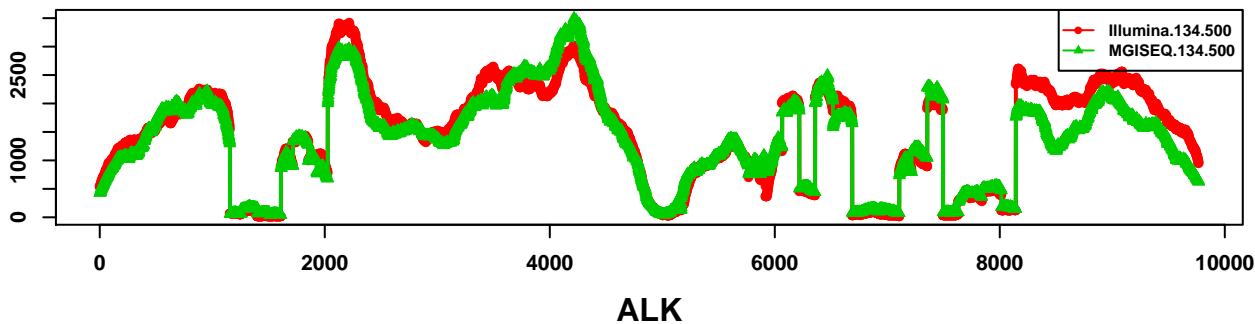

Supplement: Supplementary file 3 [file Presentation2.zip › ALK/19N02331T.pdf]

Sequencing Depth

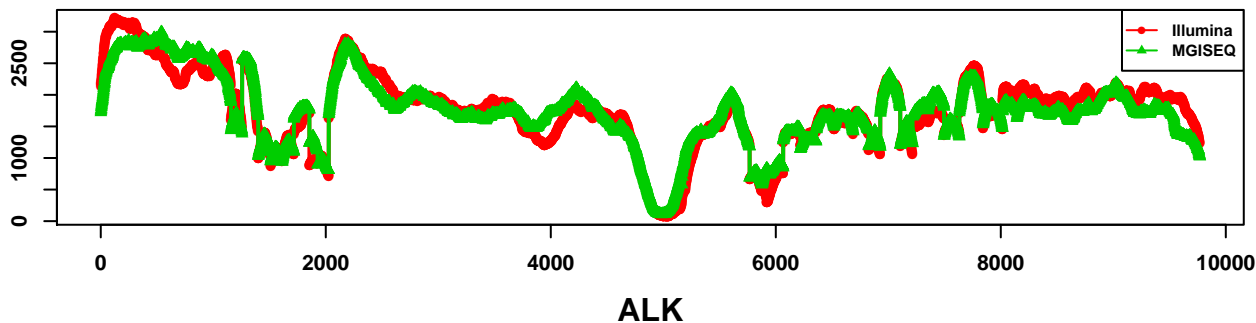

Sequencing Depth

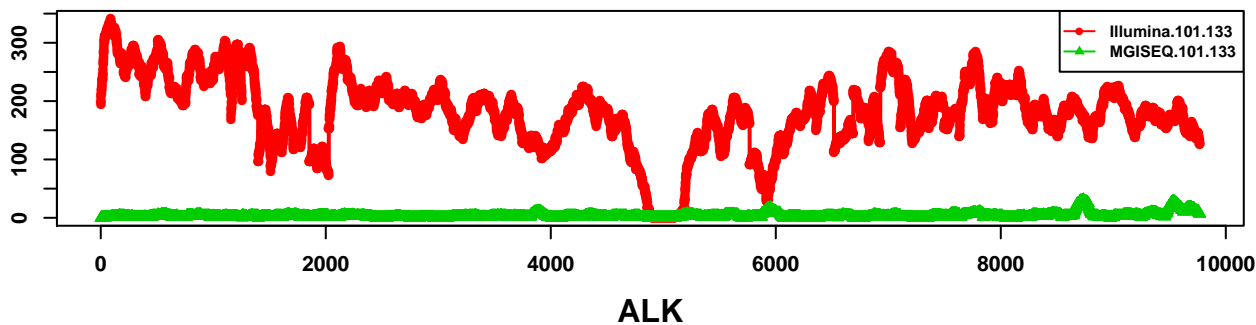

Sequencing Depth

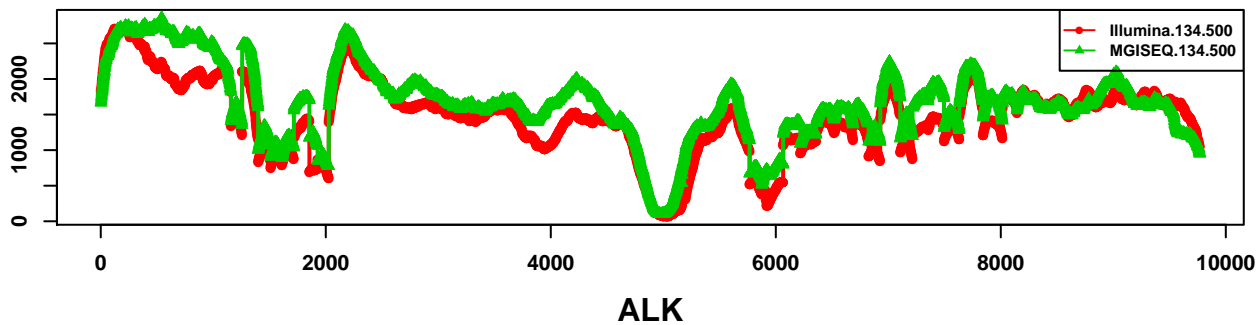

Supplement: Supplementary file 3 [file Presentation2.zip › ALK/19HE22108F.pdf]

Sequencing Depth

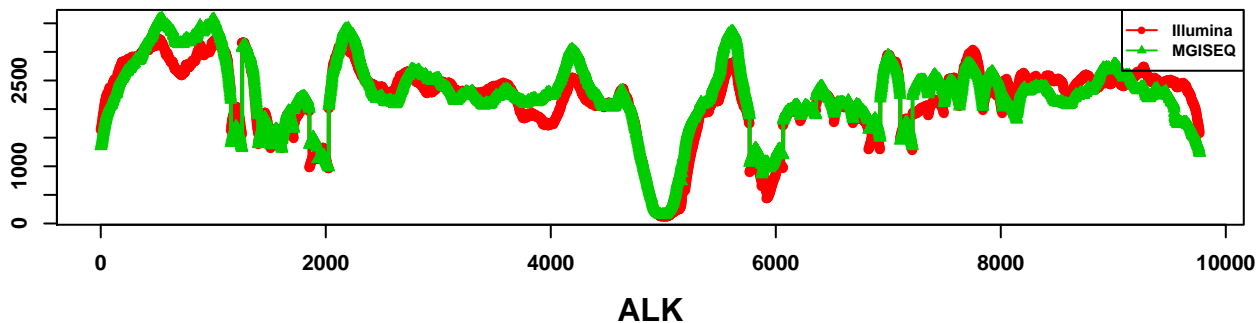

Sequencing Depth

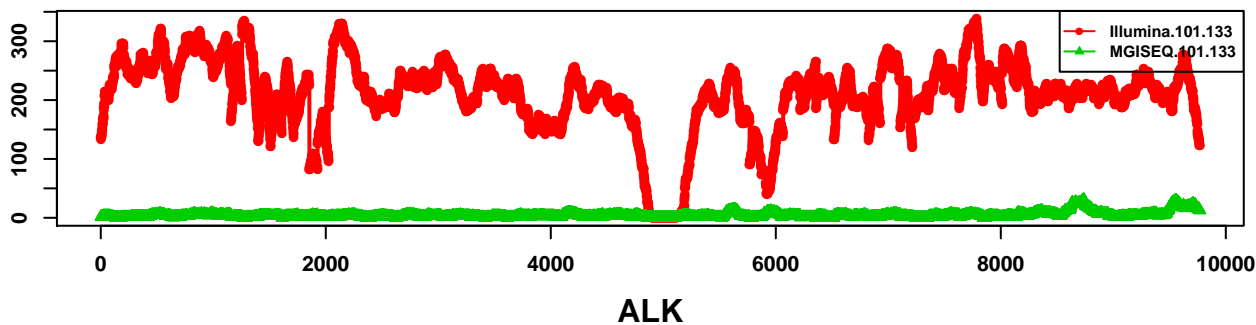

Sequencing Depth

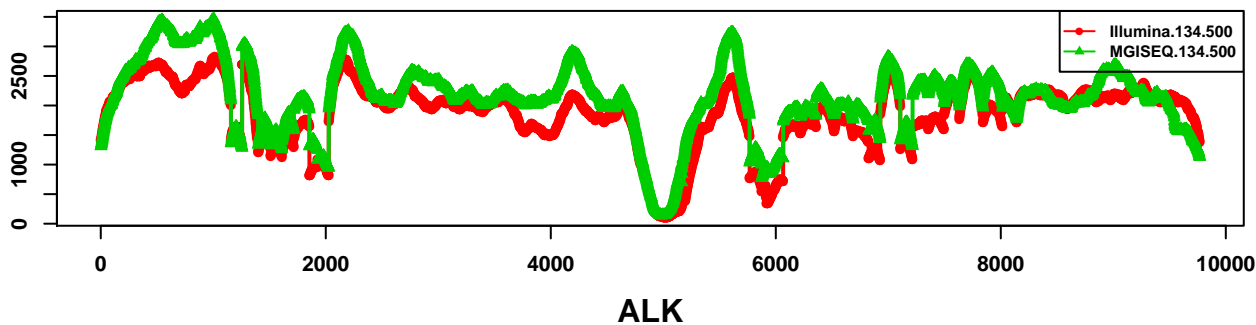

Supplement: Supplementary file 3 [file Presentation2.zip › ALK/19ZN11292F.pdf]

Sequencing Depth

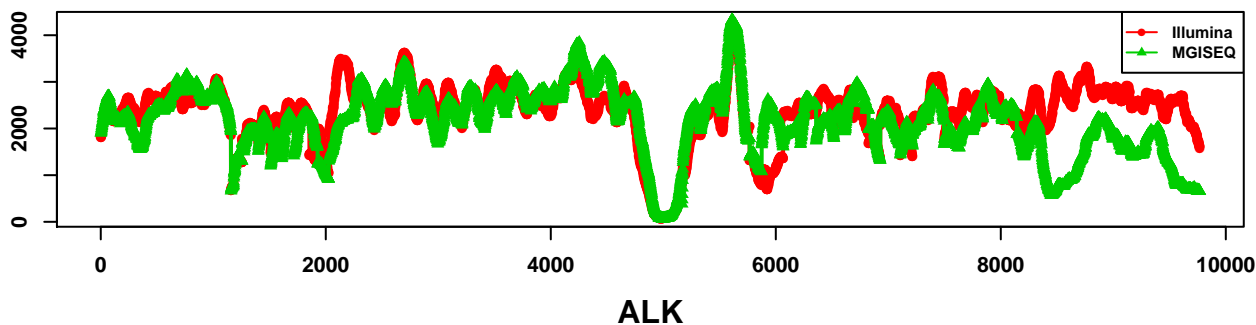

Sequencing Depth

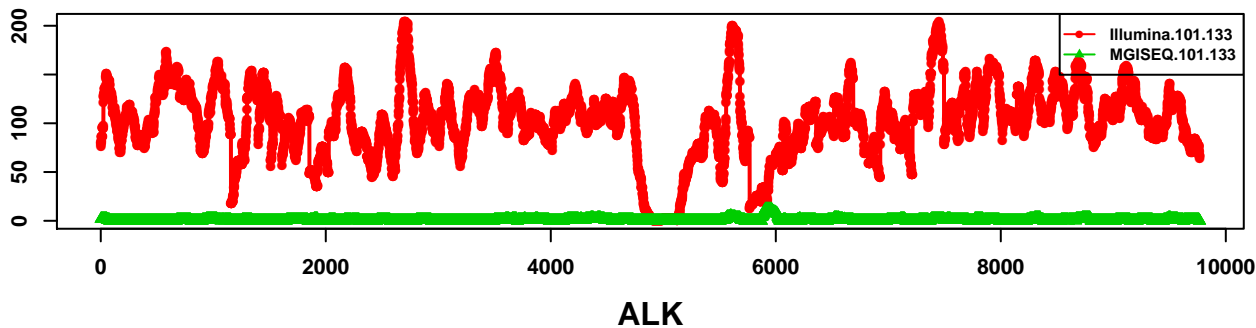

Sequencing Depth

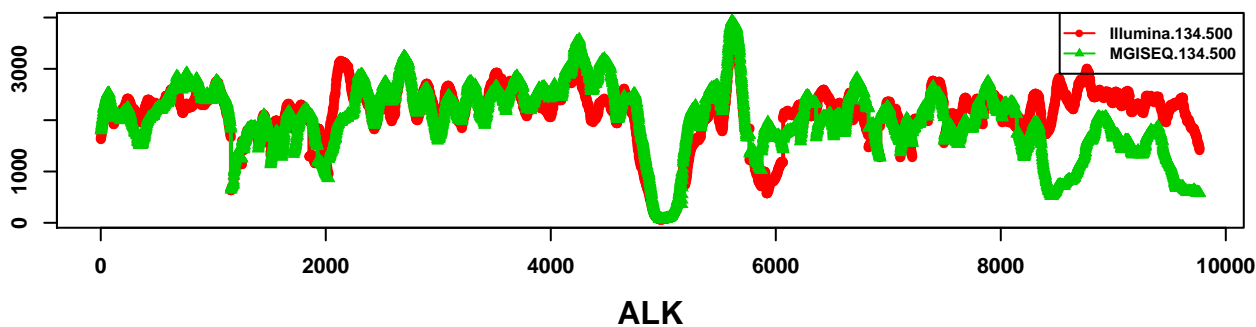

Supplement: Supplementary file 3 [file Presentation2.zip › ALK/19HS86165P.pdf]

Sequencing Depth

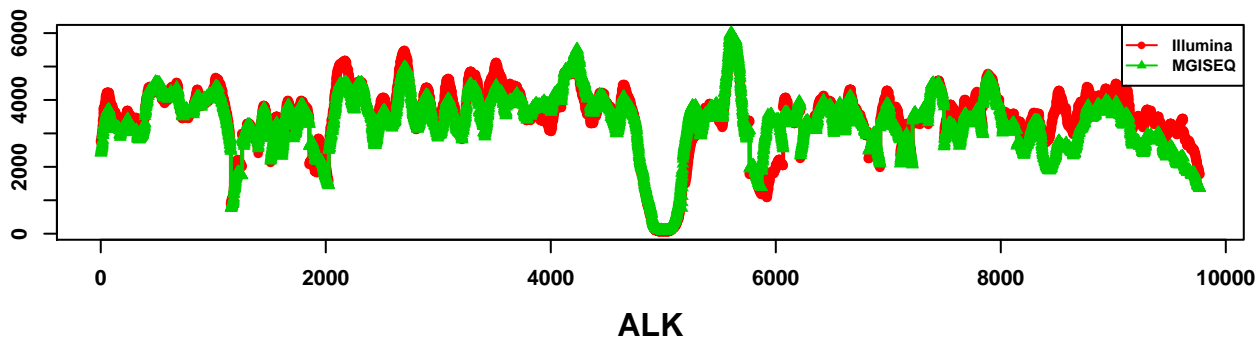

Sequencing Depth

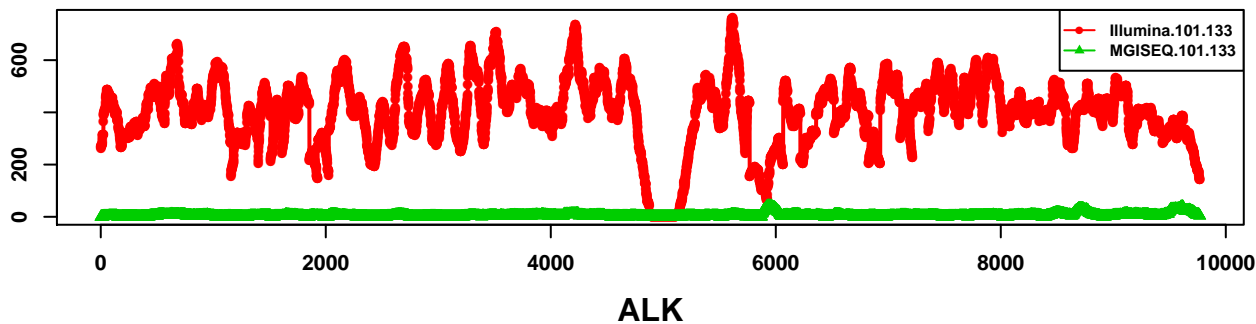

Sequencing Depth

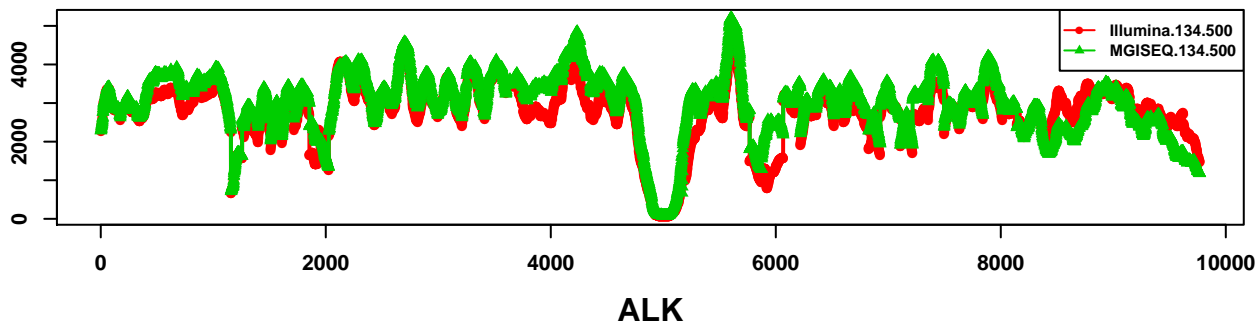

Supplement: Supplementary file 3 [file Presentation2.zip › ALK/FZ19-03014P.pdf]

Sequencing Depth

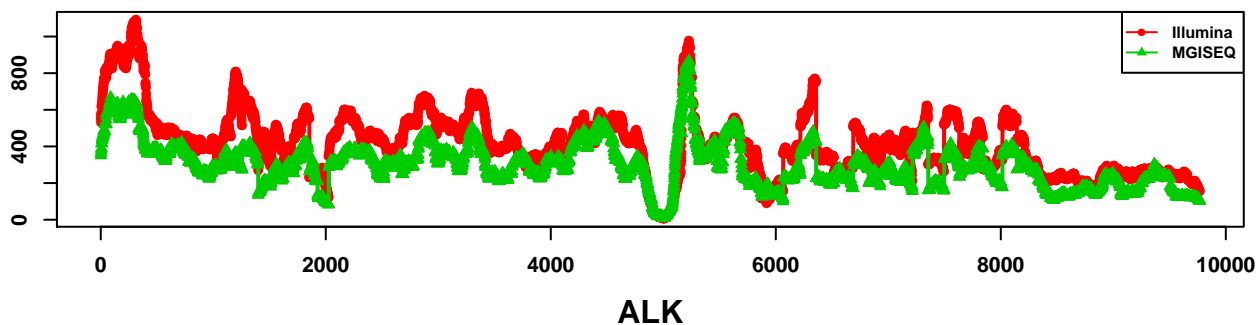

Sequencing Depth

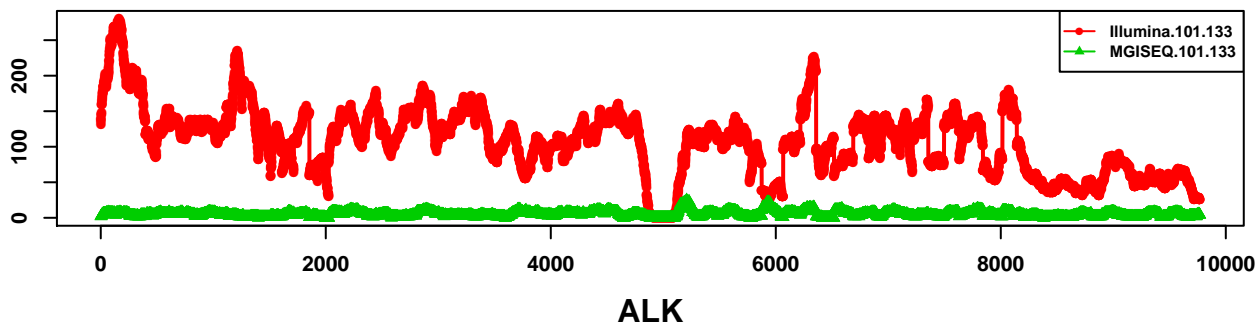

Sequencing Depth

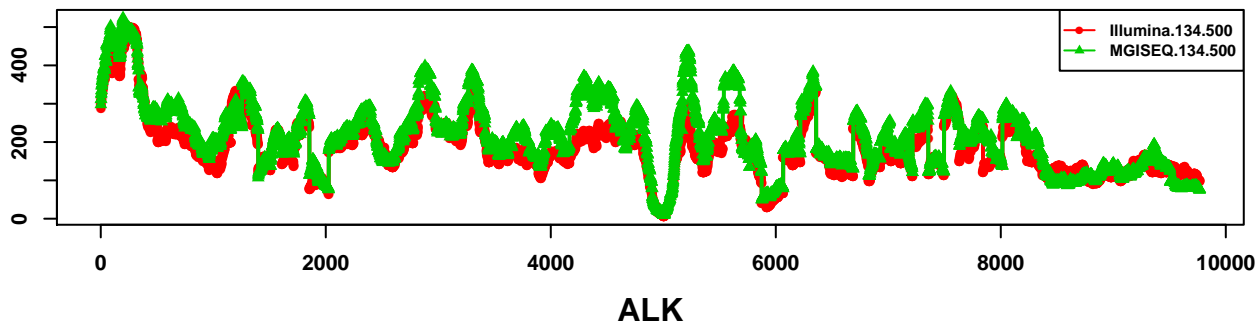

Supplement: Supplementary file 3 [file Presentation2.zip › ALK/19HE21737-IIF.pdf]

Sequencing Depth

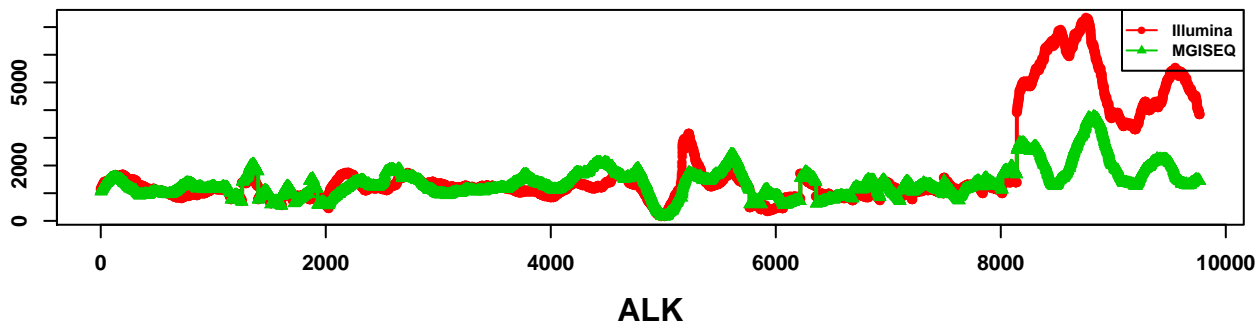

Sequencing Depth

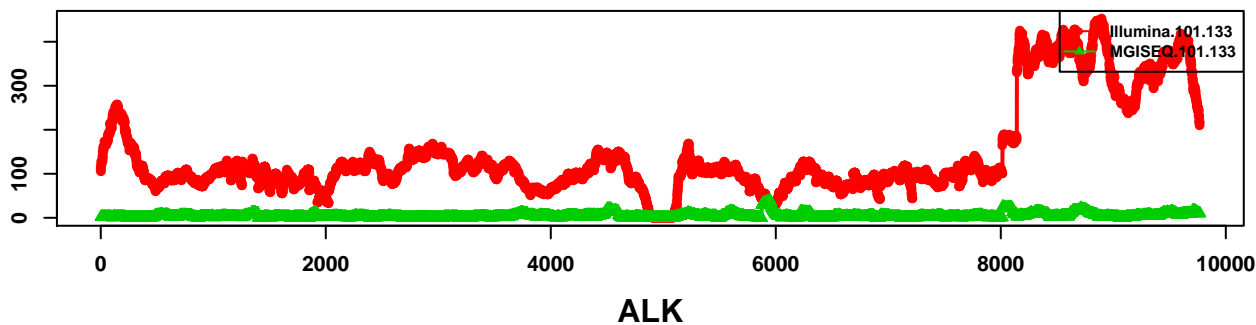

Sequencing Depth

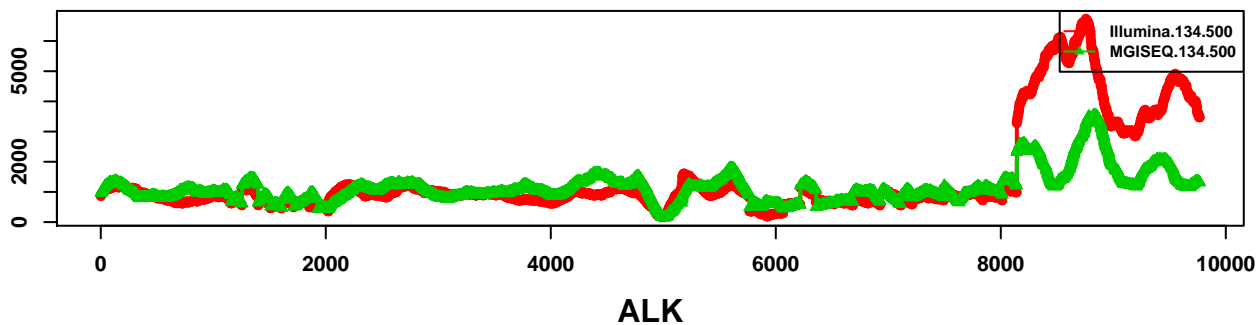

Supplement: Supplementary file 3 [file Presentation2.zip › ALK/19ZN13094F.pdf]

Sequencing Depth

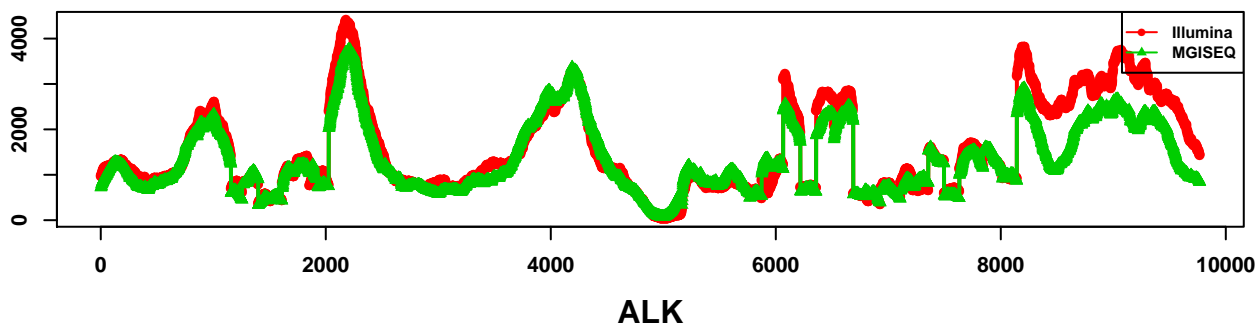

Sequencing Depth

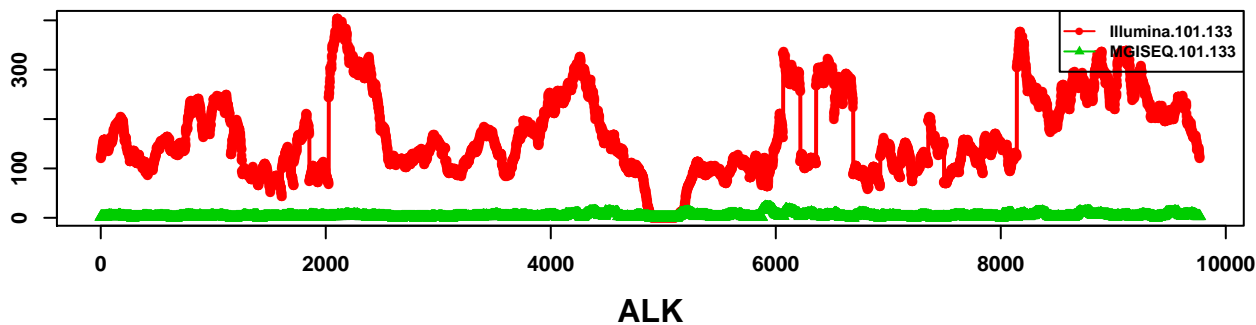

Sequencing Depth

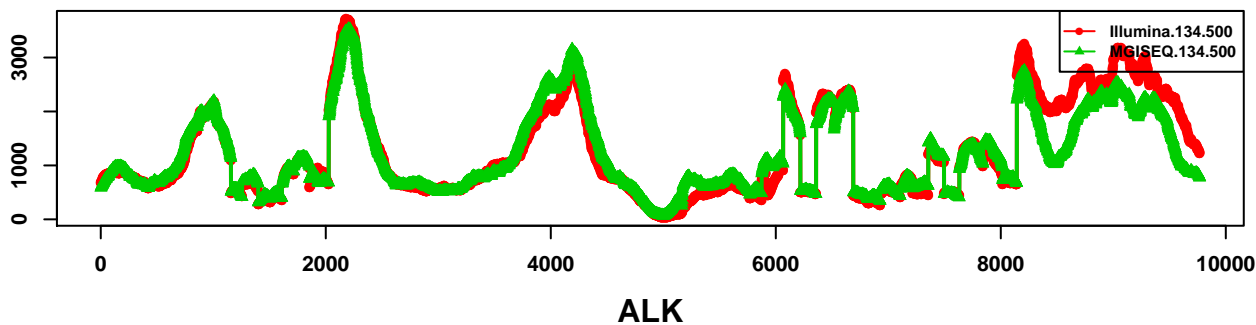

Supplement: Supplementary file 3 [file Presentation2.zip › ALK/19CY96044F.pdf]

Sequencing Depth

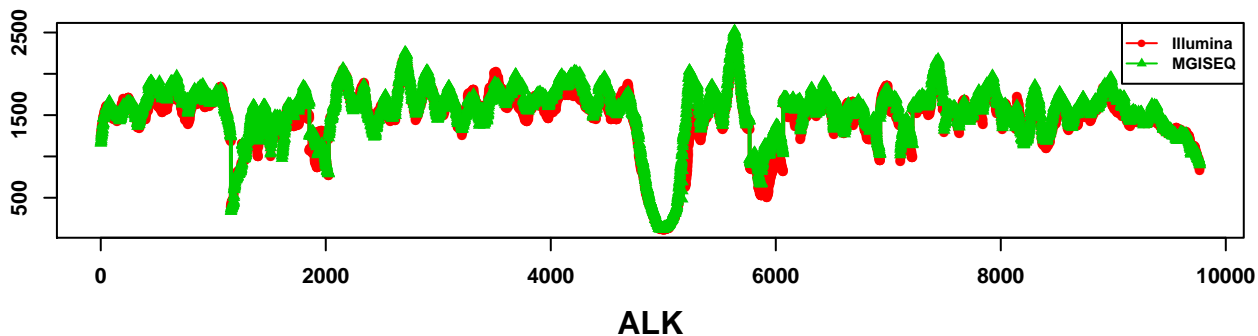

Sequencing Depth

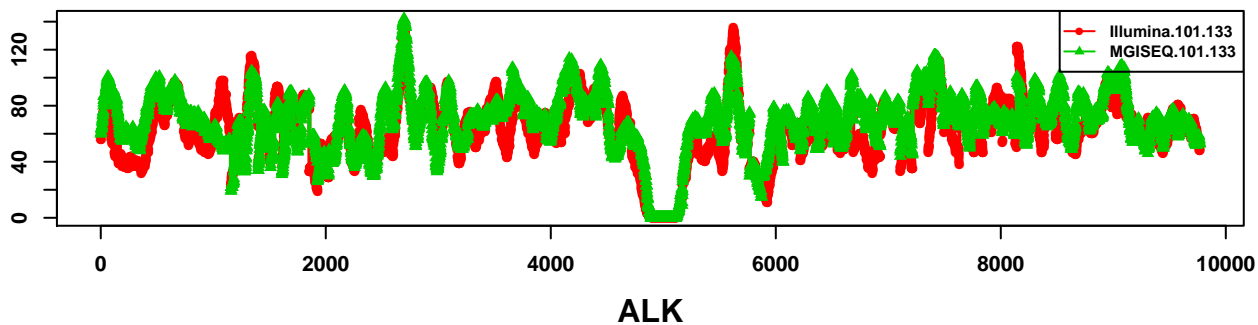

Sequencing Depth

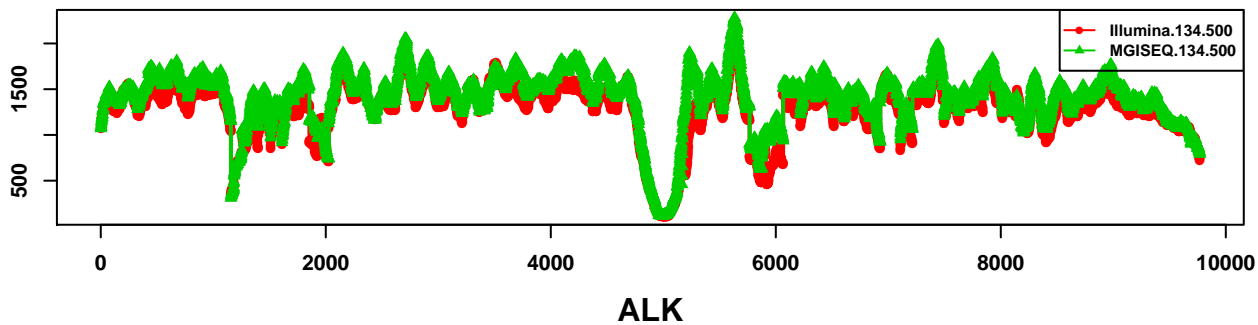

Supplement: Supplementary file 3 [file Presentation2.zip › ALK/19YT53879P.pdf]

Sequencing Depth

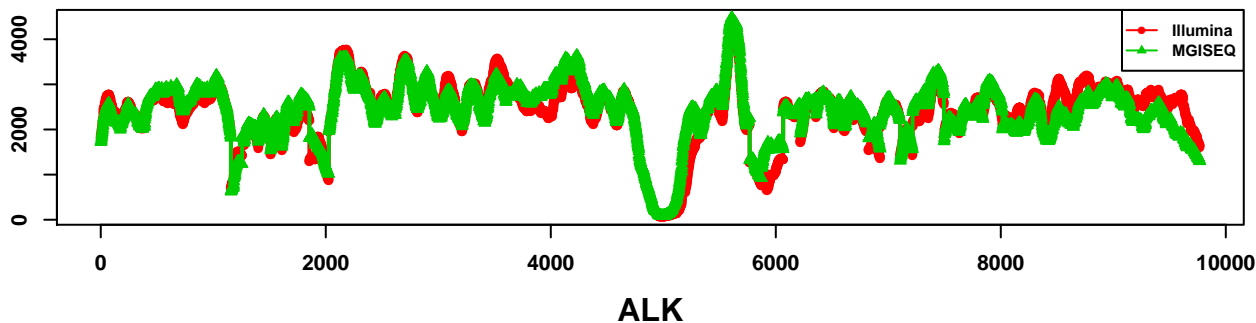

Sequencing Depth

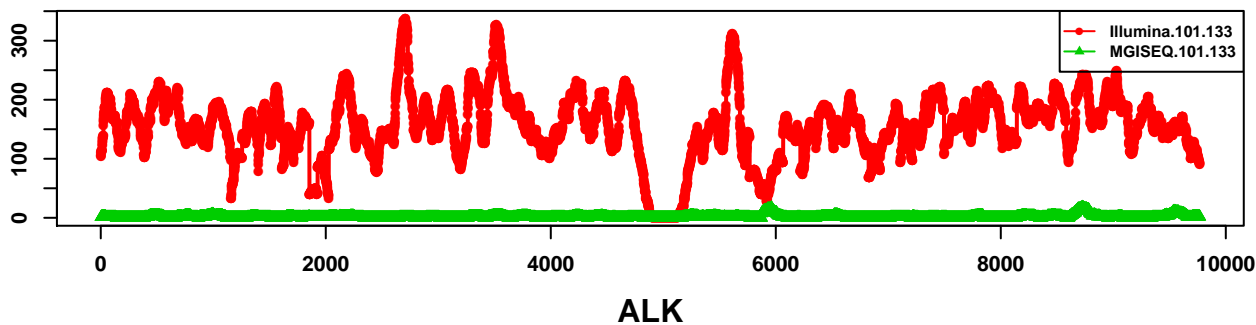

Sequencing Depth

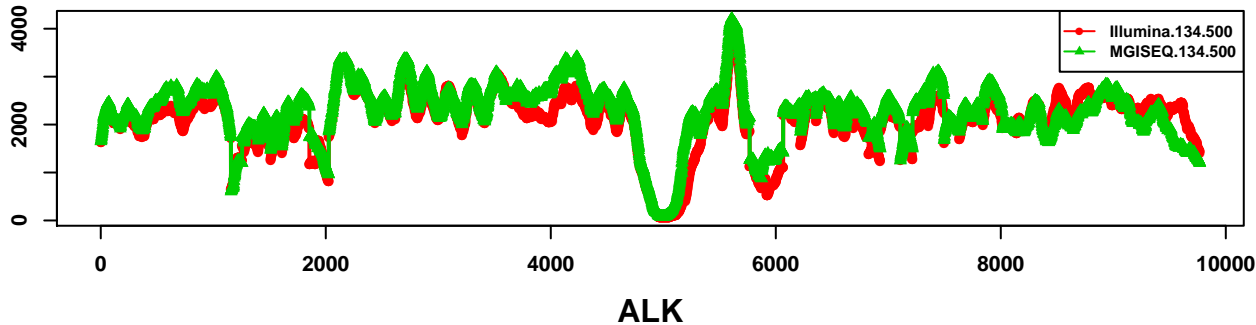

Supplement: Supplementary file 3 [file Presentation2.zip › ALK/19ZN13104P.pdf]

Sequencing Depth

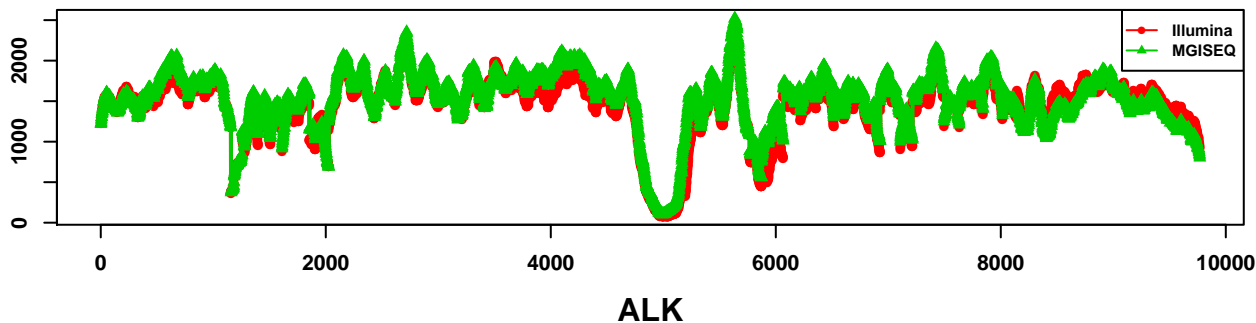

Sequencing Depth

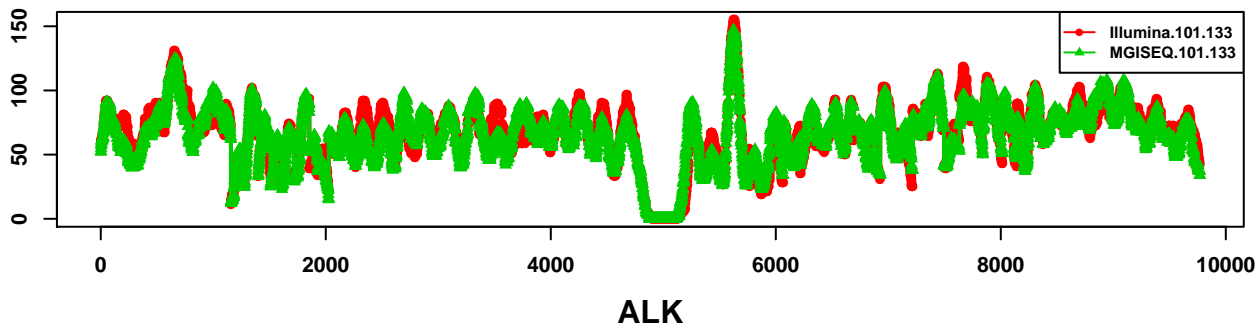

Sequencing Depth

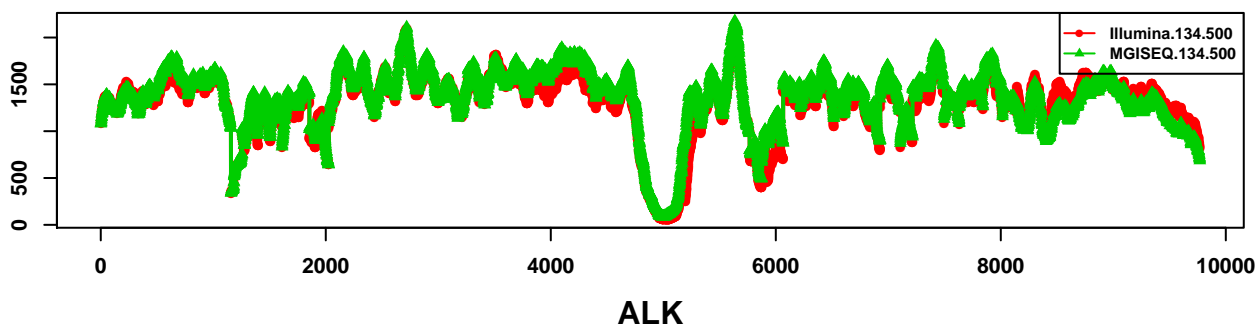

Supplement: Supplementary file 3 [file Presentation2.zip › ALK/19ZQ13135P.pdf]

Sequencing Depth

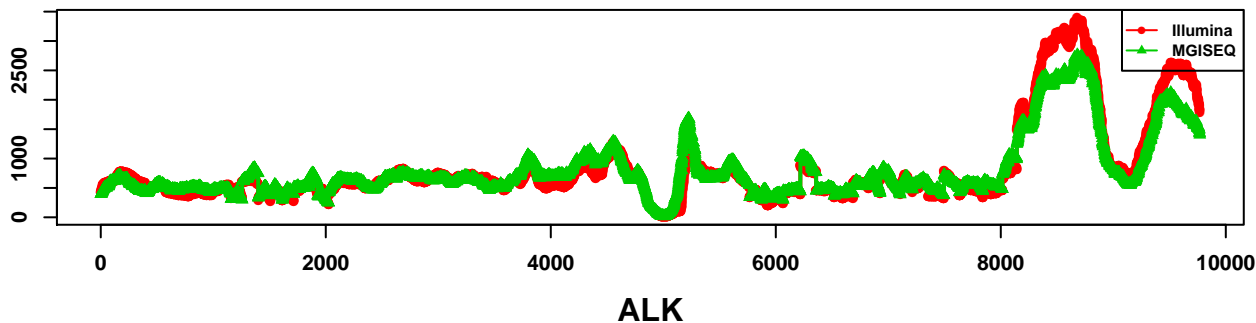

Sequencing Depth

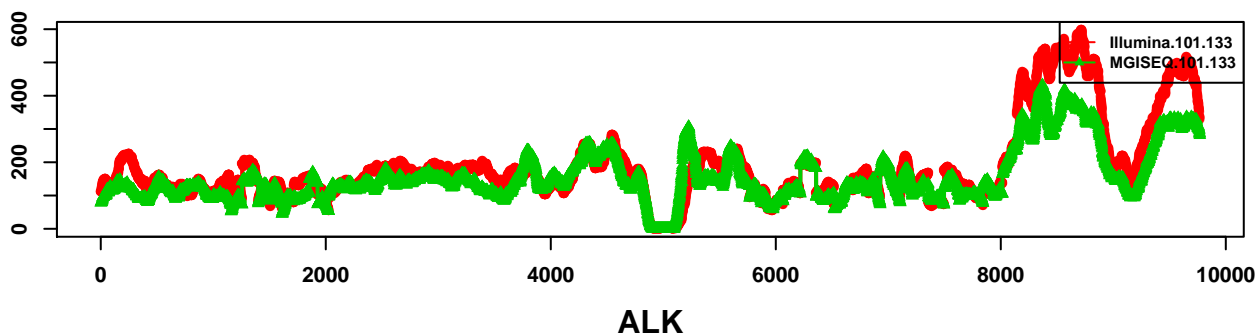

Sequencing Depth

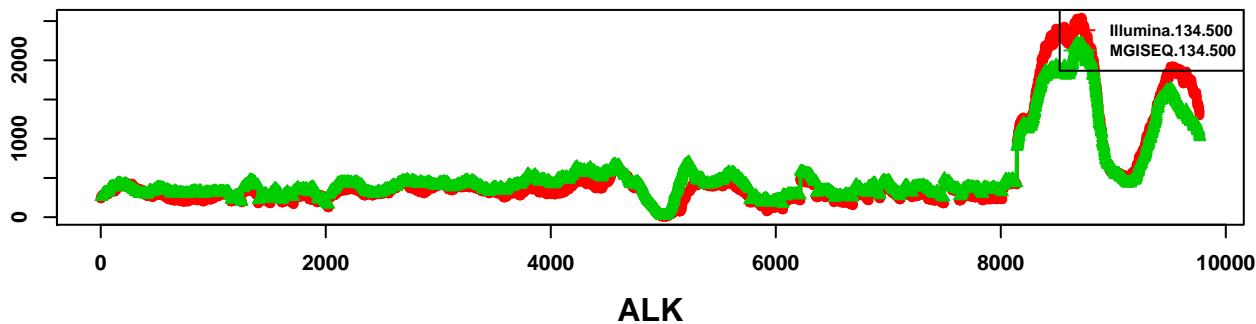

Supplement: Supplementary file 3 [file Presentation2.zip › ALK/19CF15689F.pdf]

Sequencing Depth

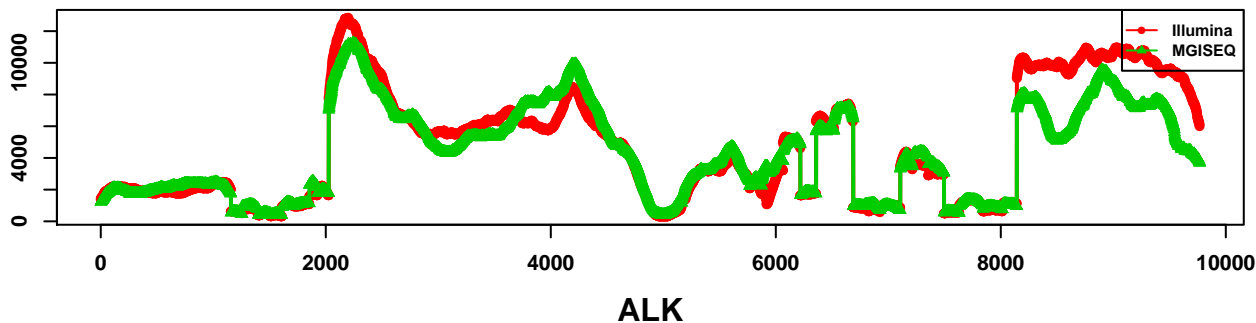

Sequencing Depth

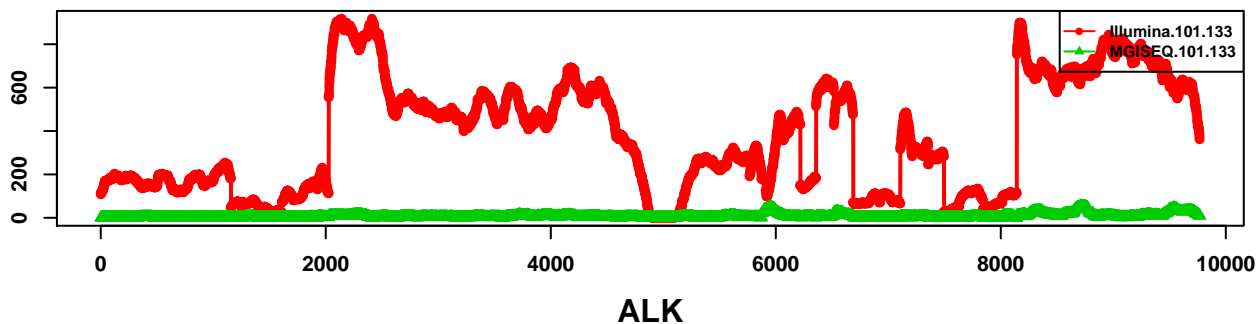

Sequencing Depth

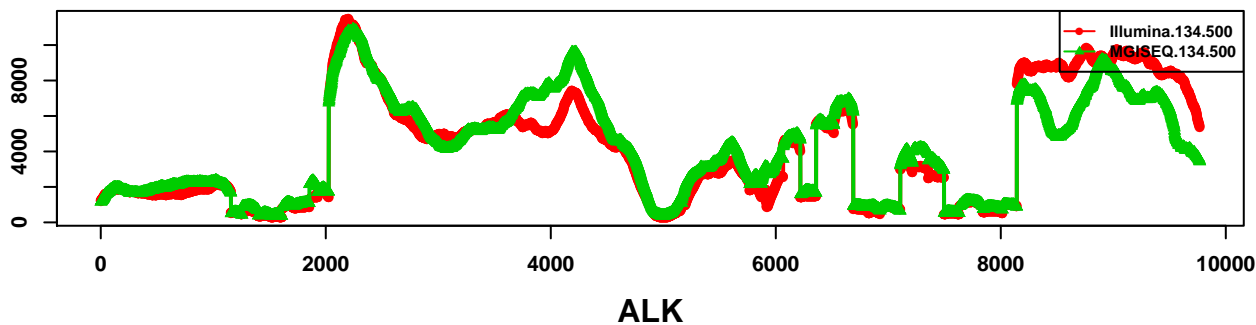

Supplement: Supplementary file 3 [file Presentation2.zip › ALK/19HE22145F.pdf]

Sequencing Depth

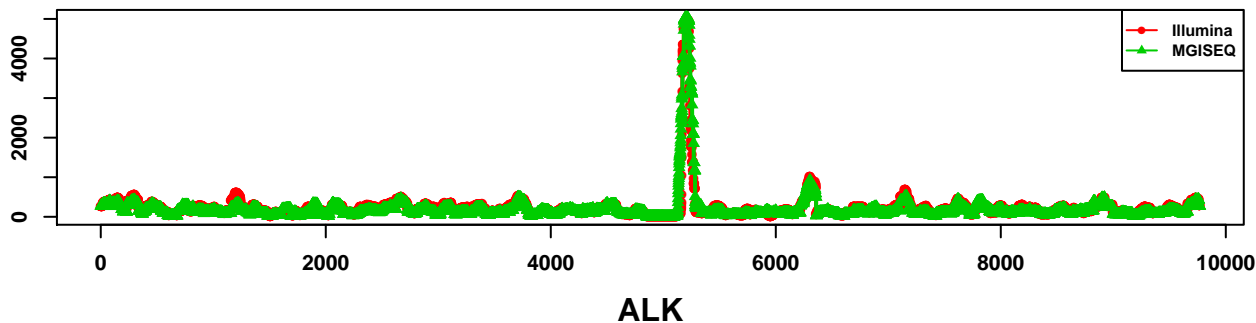

Sequencing Depth

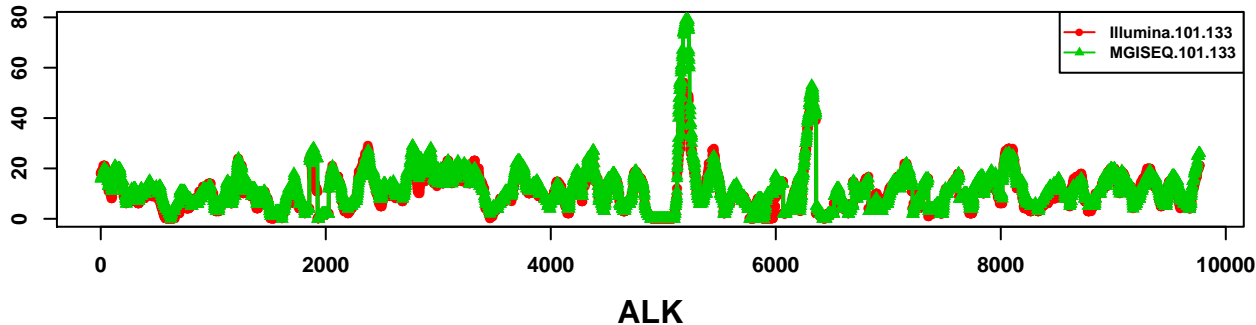

Sequencing Depth

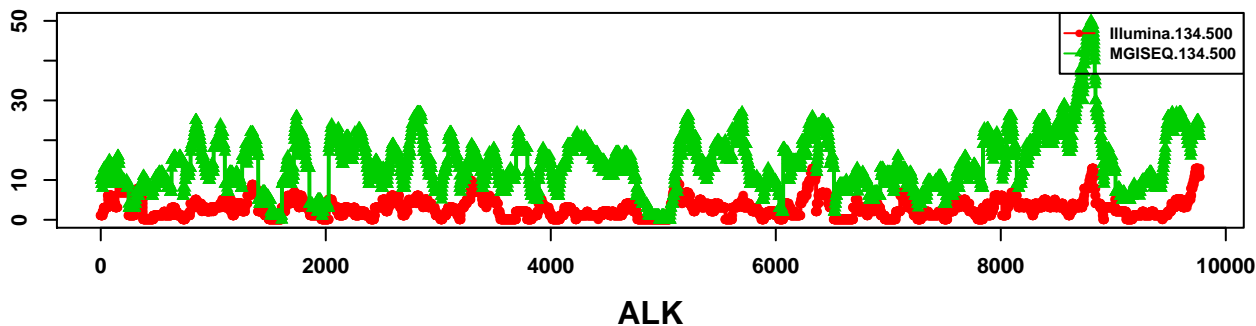

Supplement: Supplementary file 3 [file Presentation2.zip › ALK/19N01984F.pdf]

Sequencing Depth

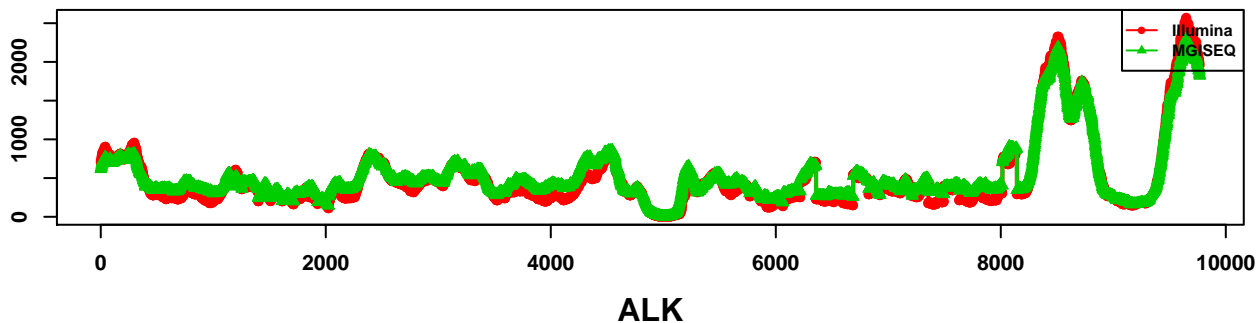

Sequencing Depth

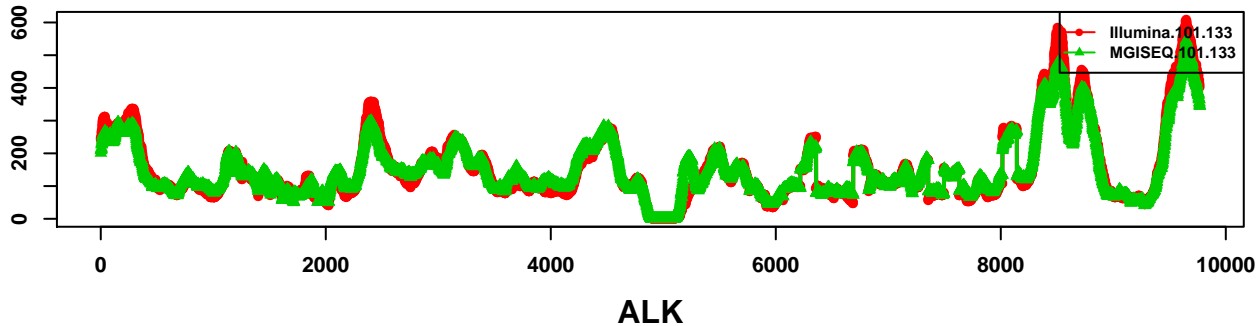

Sequencing Depth

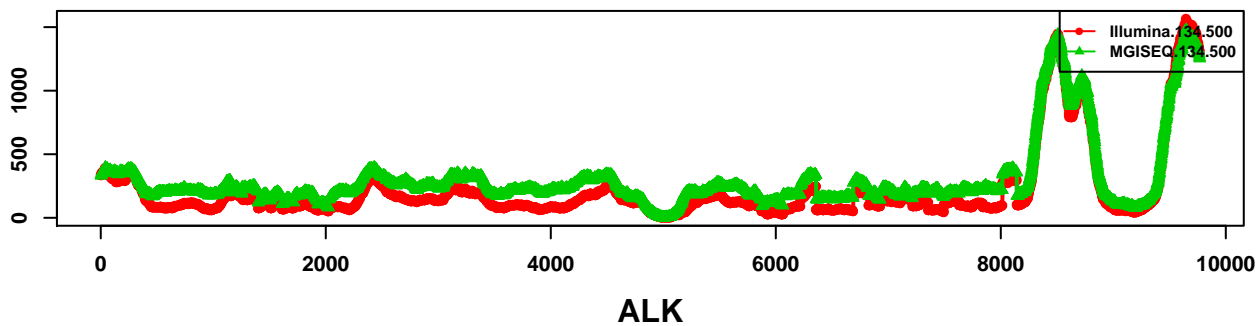

Supplement: Supplementary file 3 [file Presentation2.zip › ALK/19ZN13493T.pdf]

Sequencing Depth

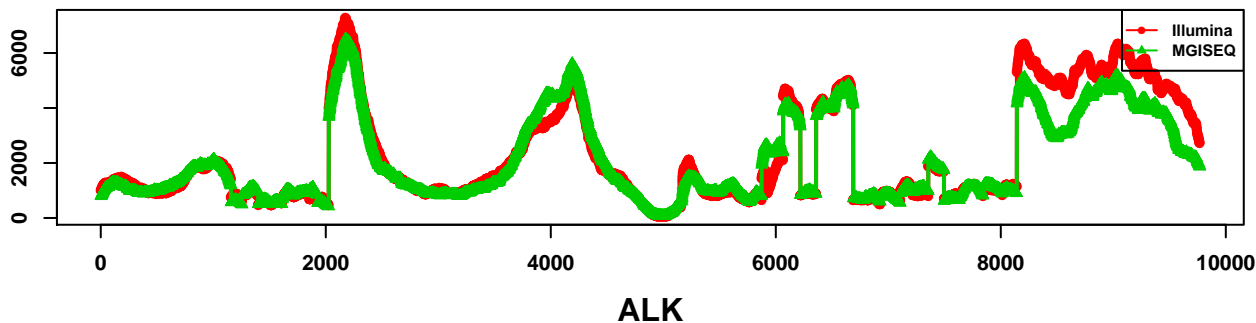

Sequencing Depth

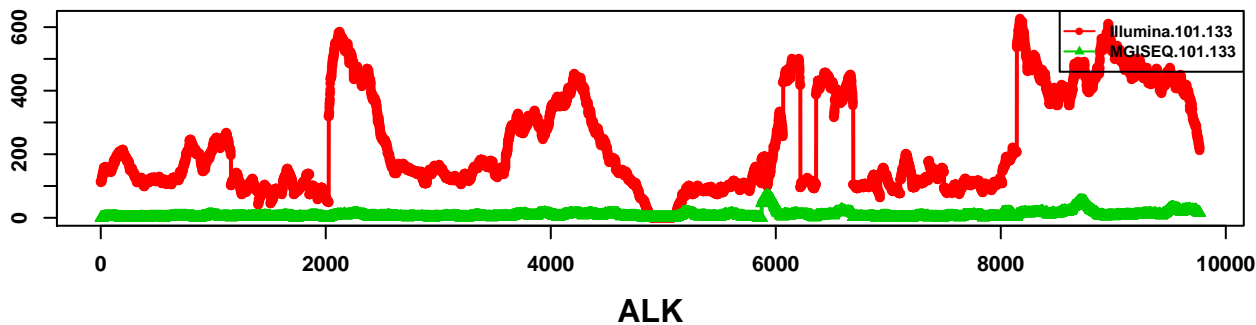

Sequencing Depth

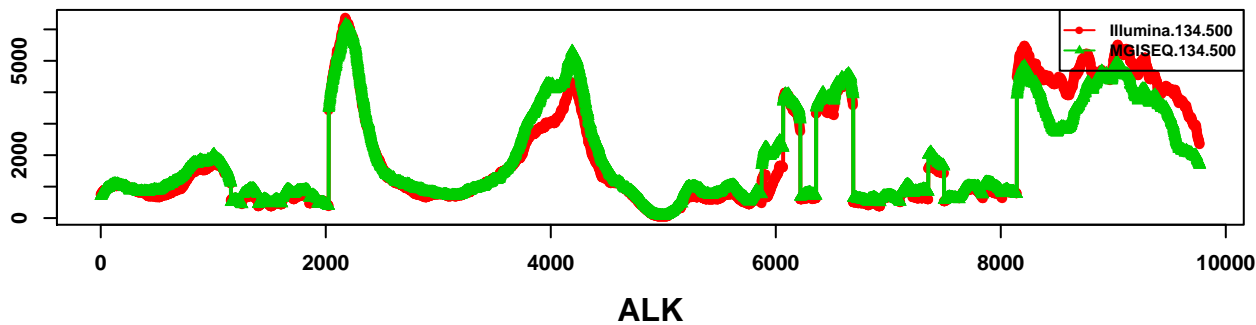

Supplement: Supplementary file 3 [file Presentation2.zip › ALK/19HS86164F.pdf]

Sequencing Depth

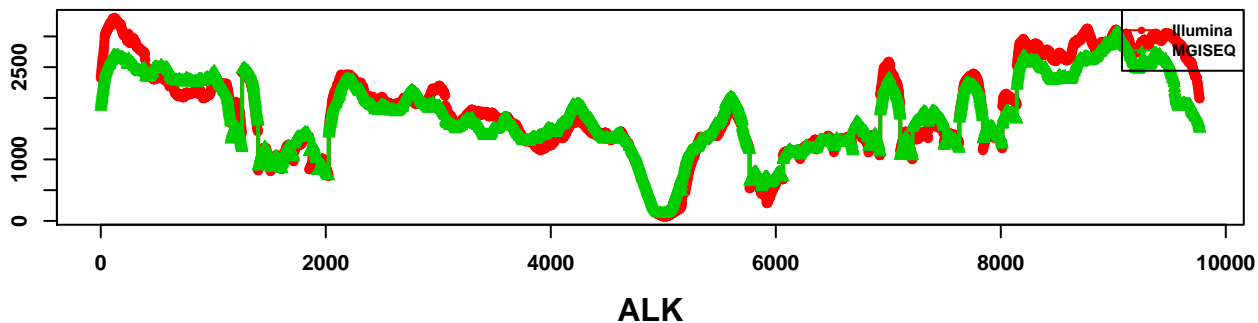

Sequencing Depth

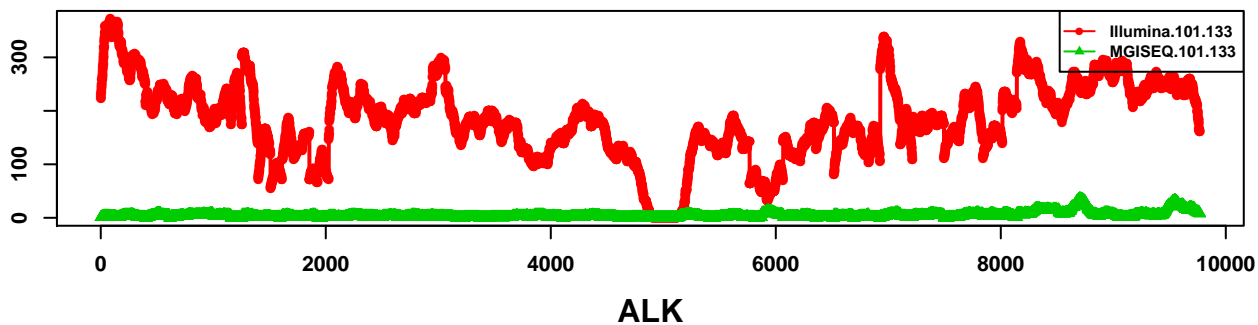

Sequencing Depth

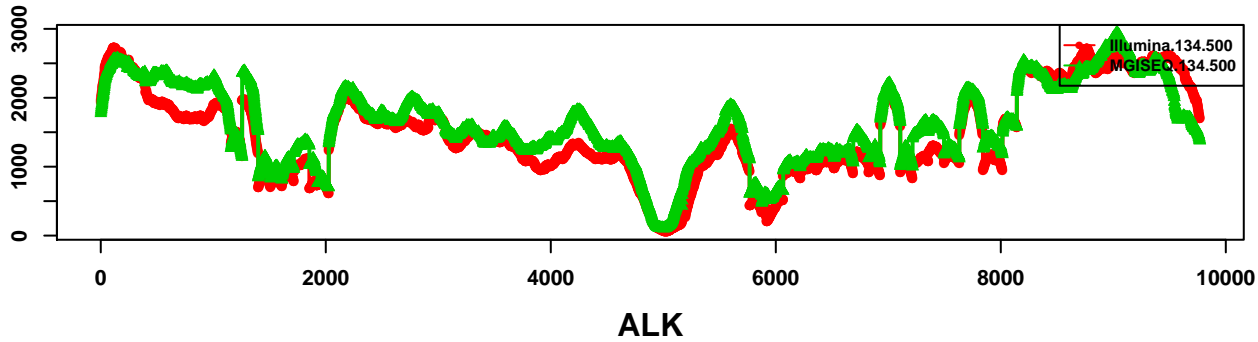

Supplement: Supplementary file 3 [file Presentation2.zip › ALK/19HE22001F.pdf]

Sequencing Depth

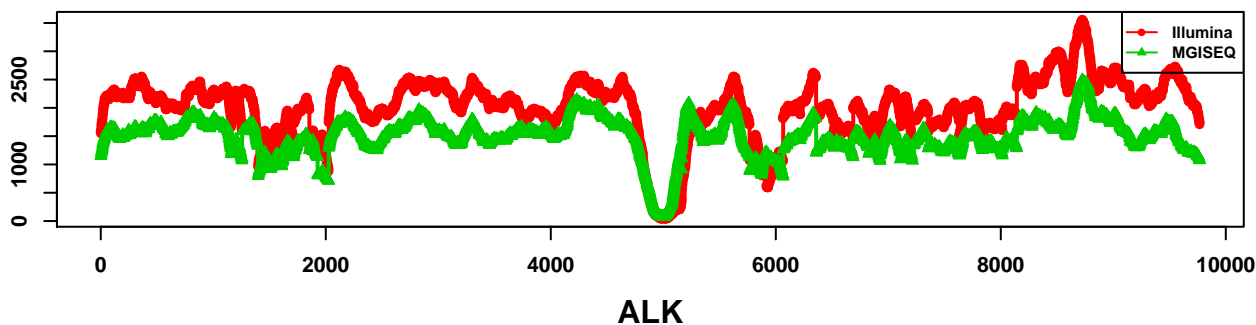

Sequencing Depth

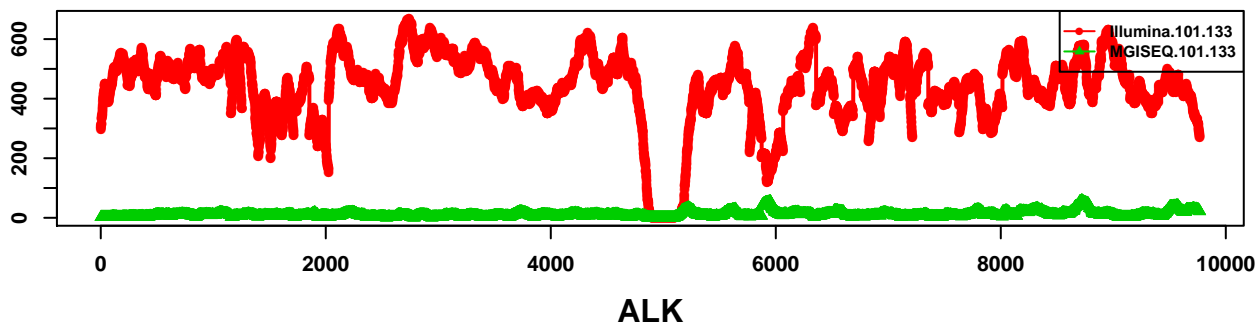

Sequencing Depth

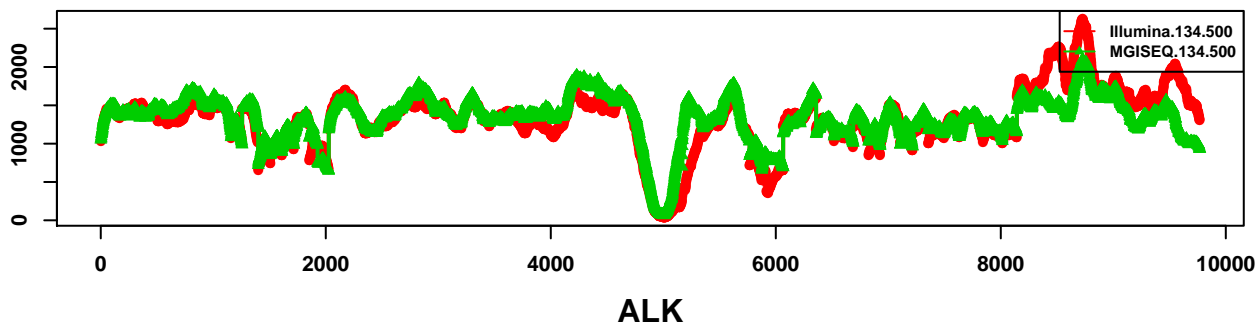

Supplement: Supplementary file 3 [file Presentation2.zip › ALK/19ZN12313F.pdf]

Sequencing Depth

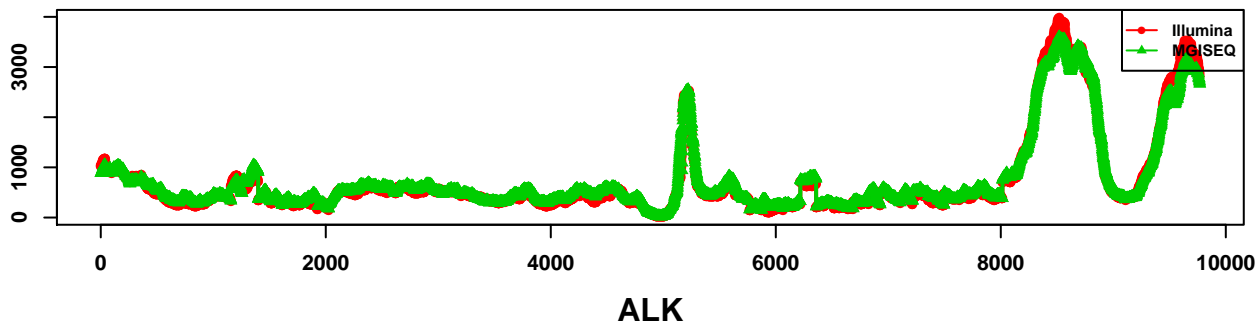

Sequencing Depth

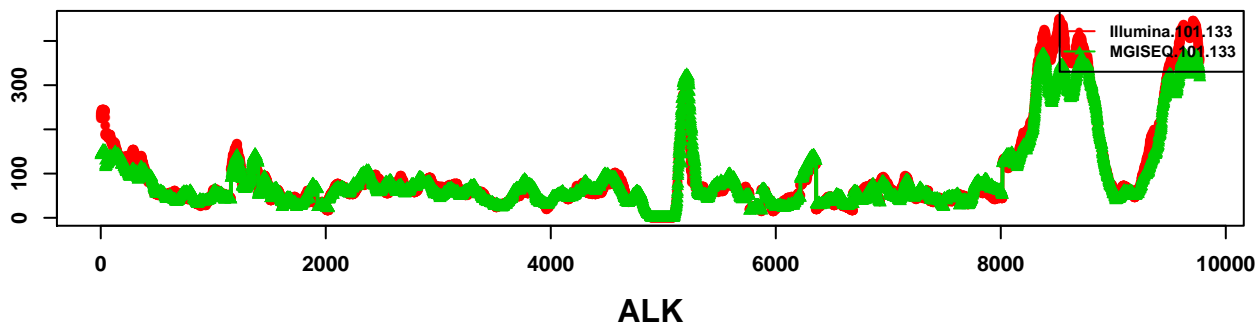

Sequencing Depth

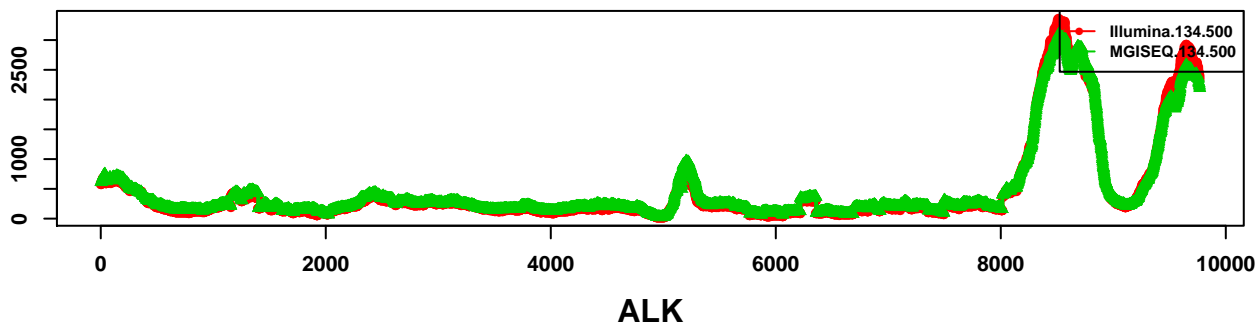

Supplement: Supplementary file 3 [file Presentation2.zip › ALK/19N01985F.pdf]

Sequencing Depth

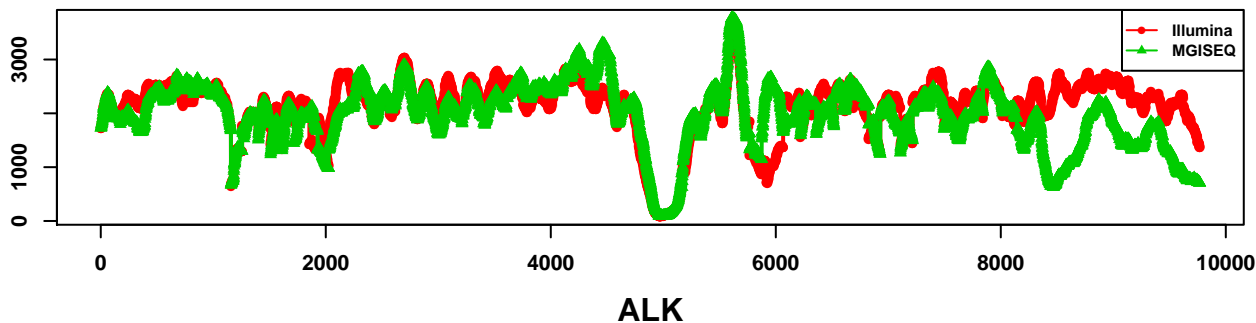

Sequencing Depth

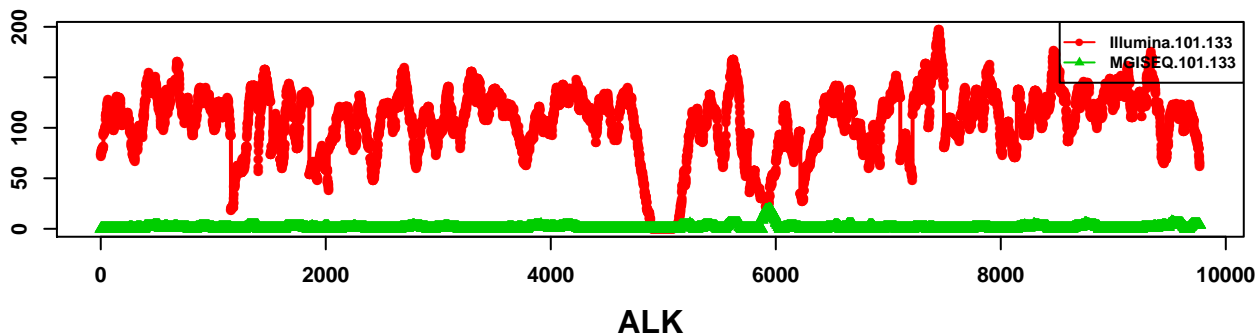

Sequencing Depth

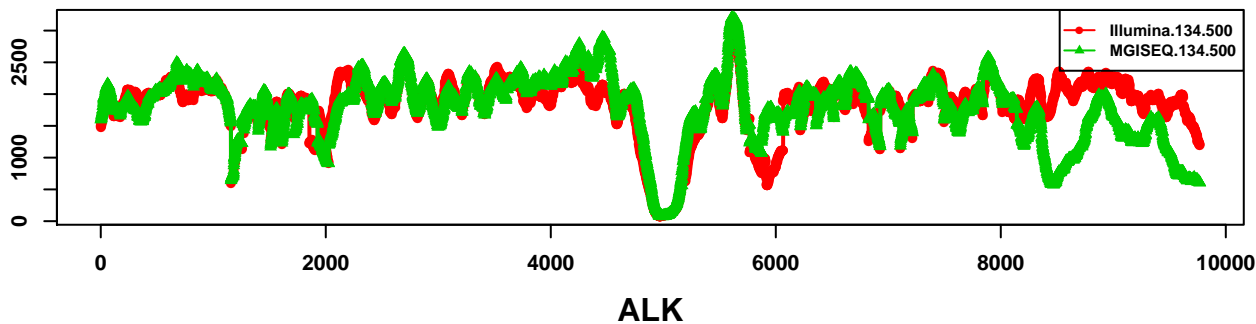

Supplement: Supplementary file 3 [file Presentation2.zip › ALK/19ZN12368P.pdf]

Sequencing Depth

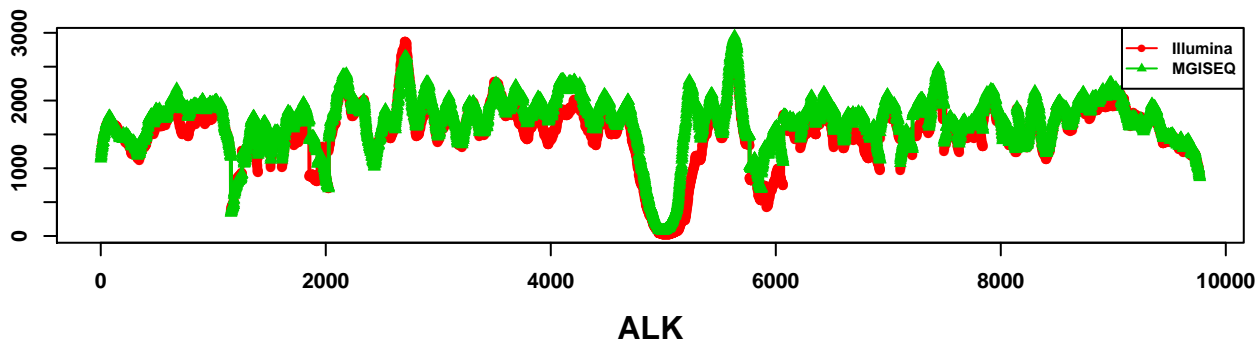

Sequencing Depth

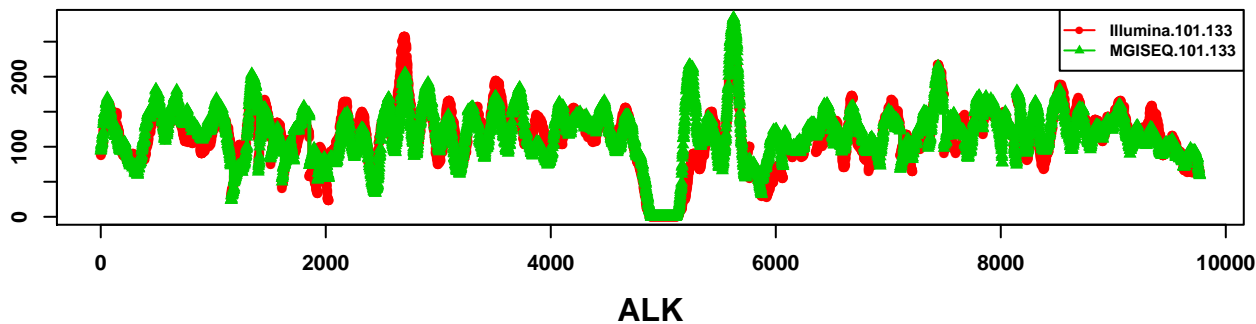

Sequencing Depth

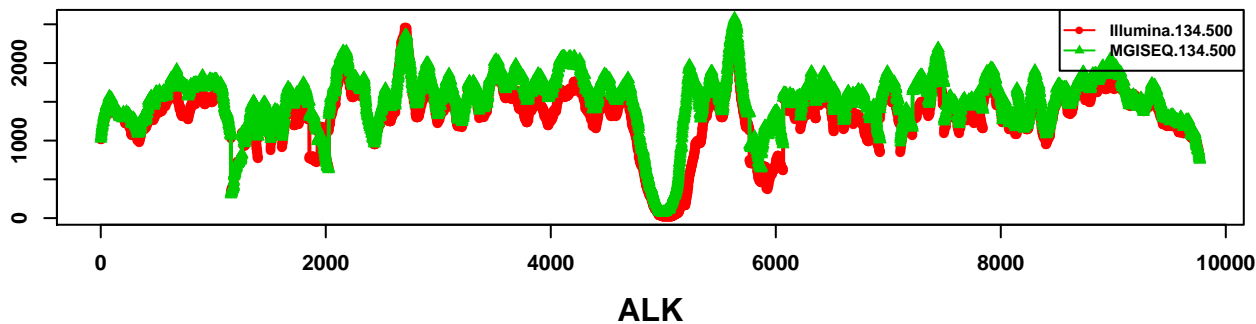

Supplement: Supplementary file 3 [file Presentation2.zip › ALK/19ZN13980P.pdf]

Sequencing Depth

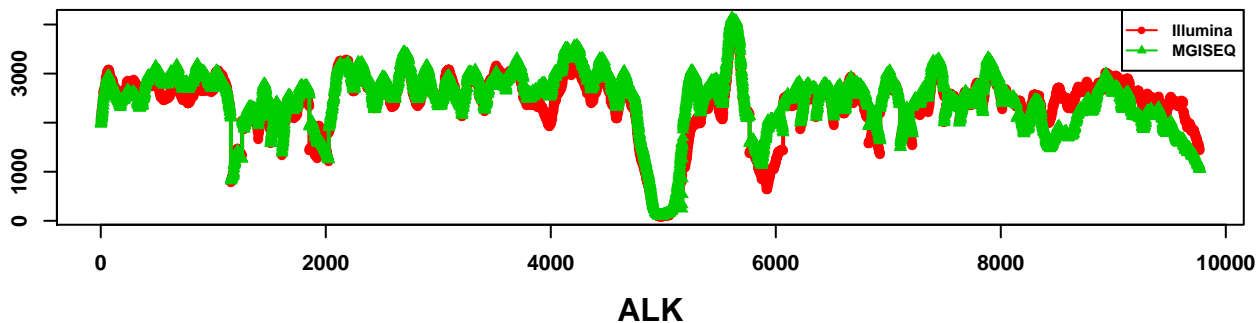

Sequencing Depth

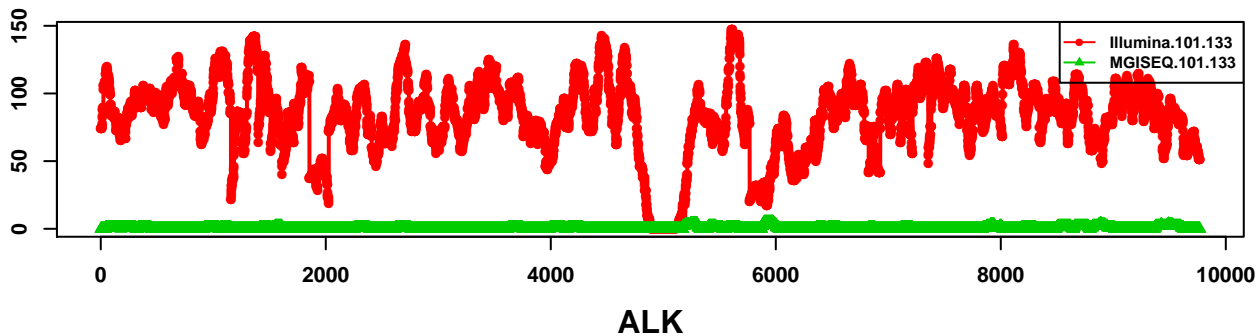

Sequencing Depth

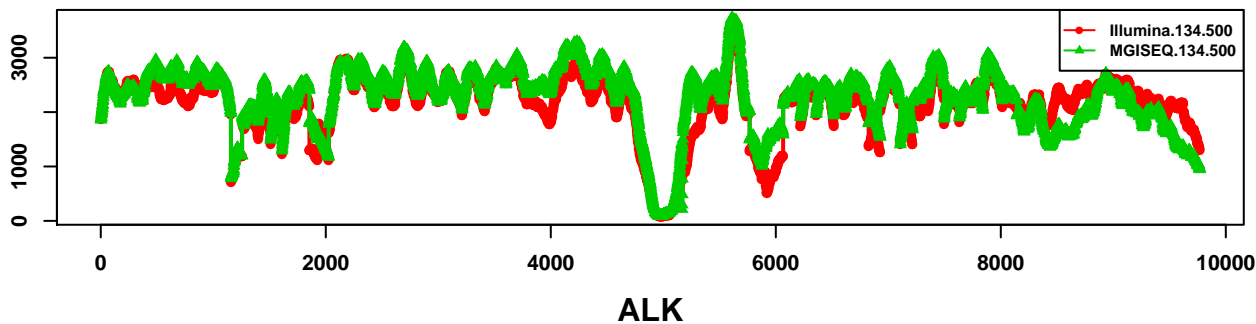

Supplement: Supplementary file 3 [file Presentation2.zip › ALK/19N01383P.pdf]

Sequencing Depth

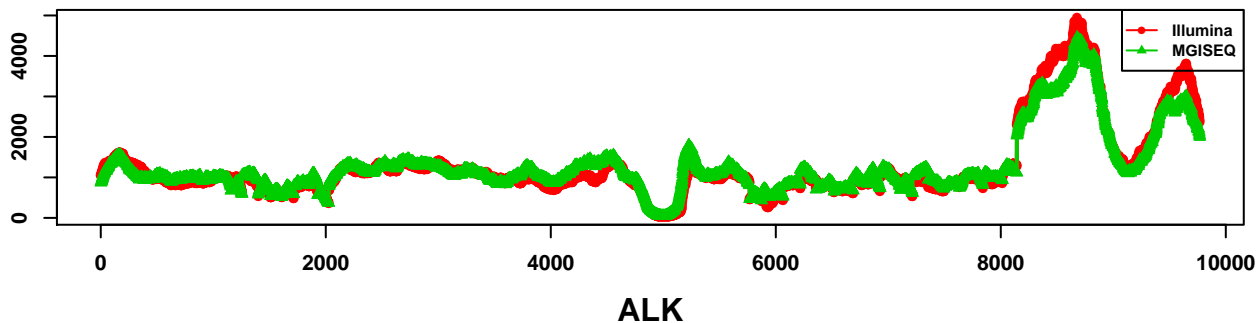

Sequencing Depth

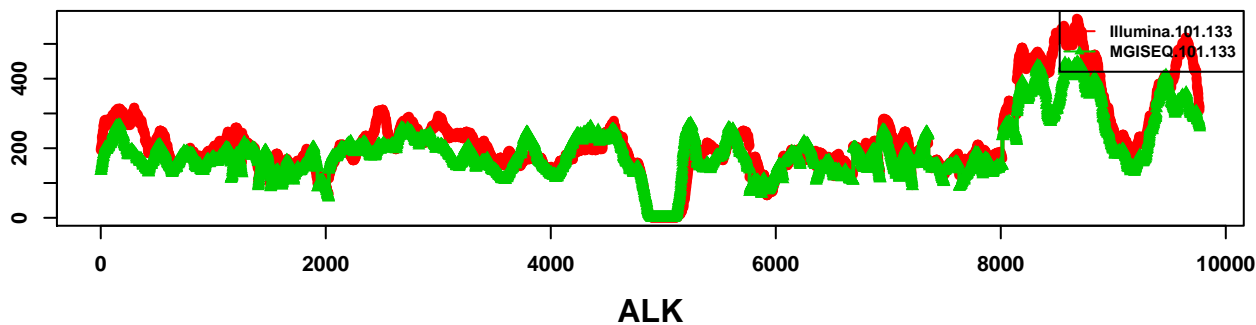

Sequencing Depth

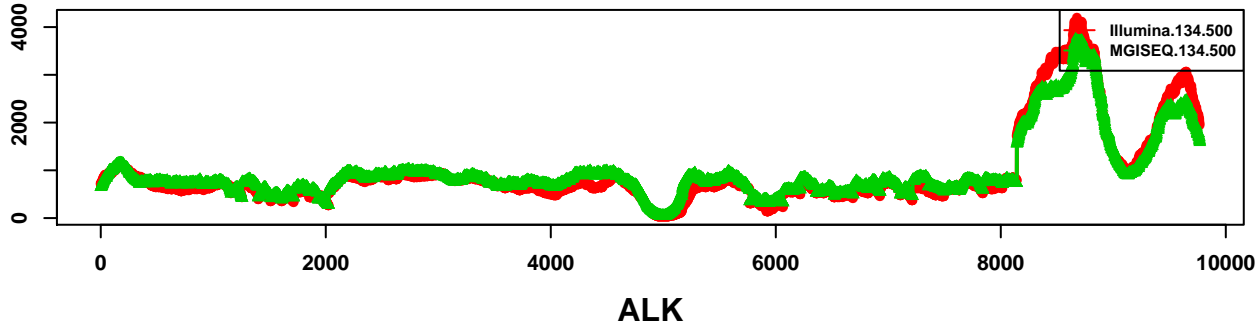

Supplement: Supplementary file 3 [file Presentation2.zip › ALK/19ZN12597F.pdf]

Sequencing Depth

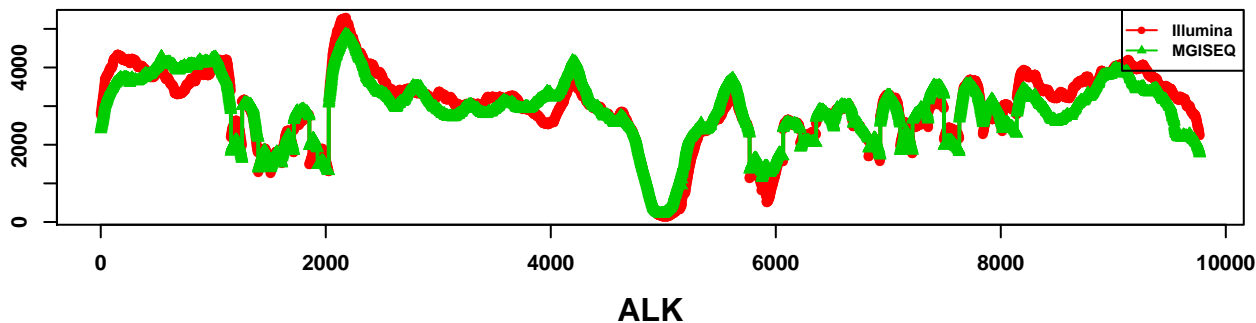

Sequencing Depth

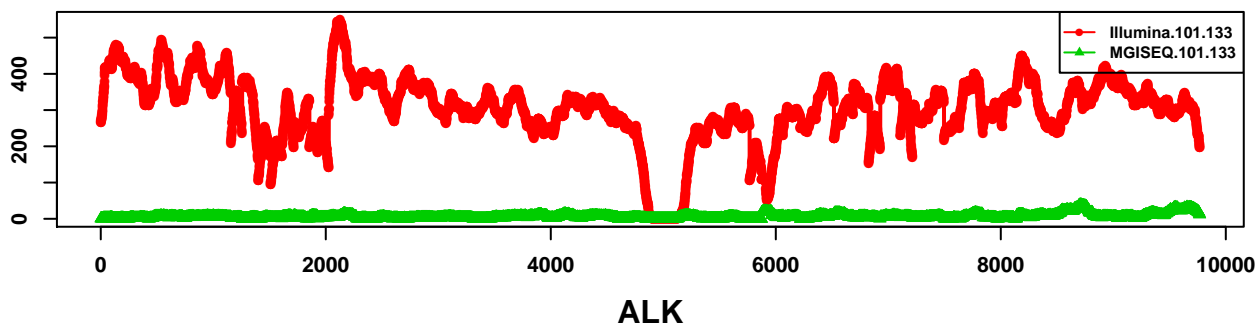

Sequencing Depth

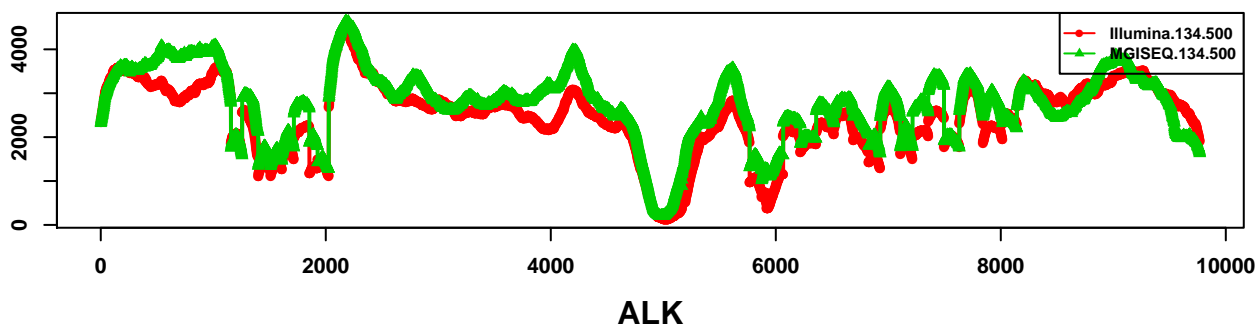

Supplement: Supplementary file 3 [file Presentation2.zip › ALK/19HE22187F.pdf]

Sequencing Depth

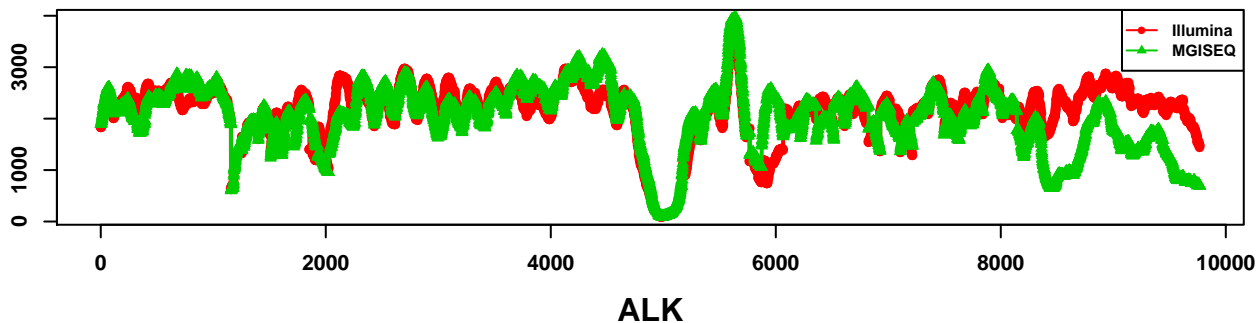

Sequencing Depth

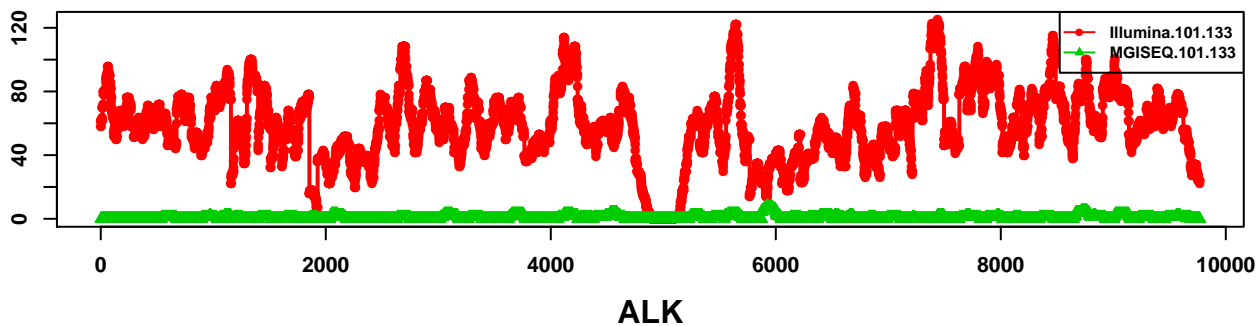

Sequencing Depth

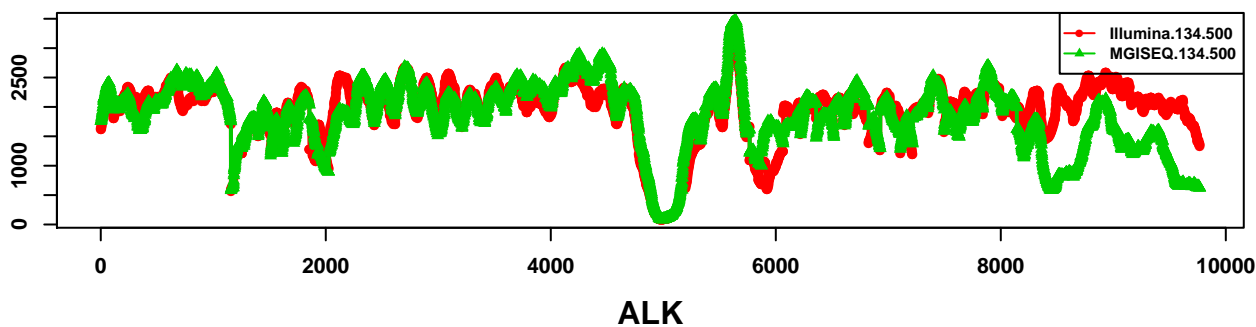

Supplement: Supplementary file 3 [file Presentation2.zip › ALK/19JS48182P.pdf]

Sequencing Depth

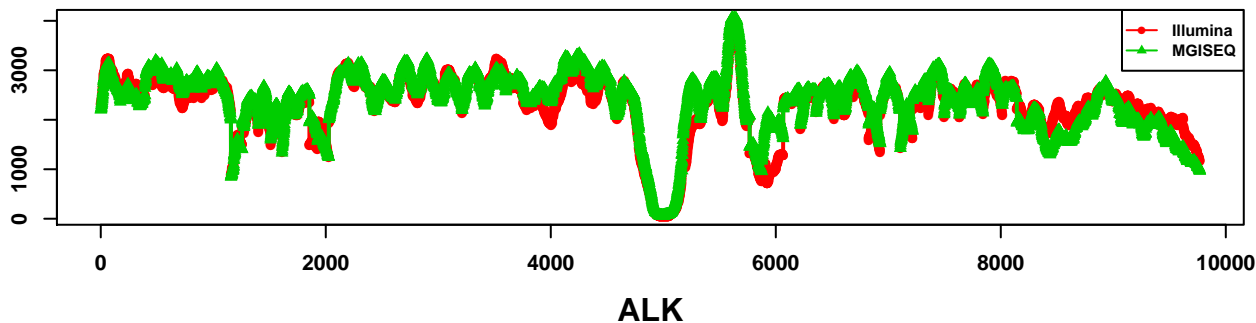

Sequencing Depth

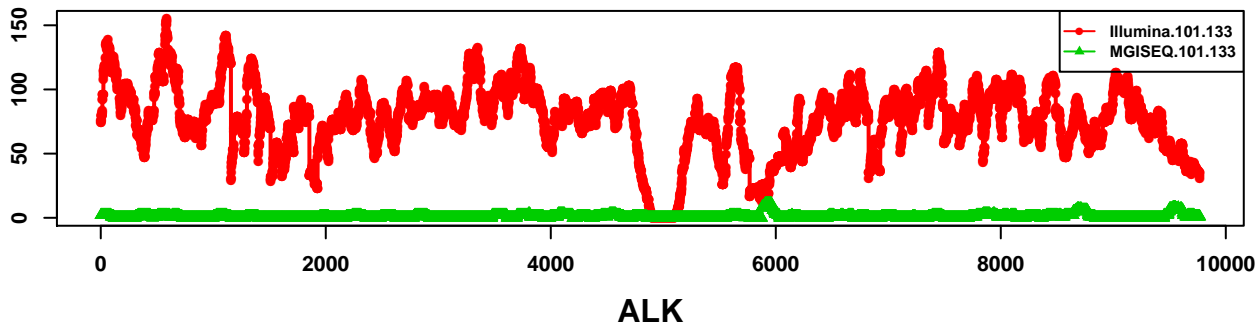

Sequencing Depth

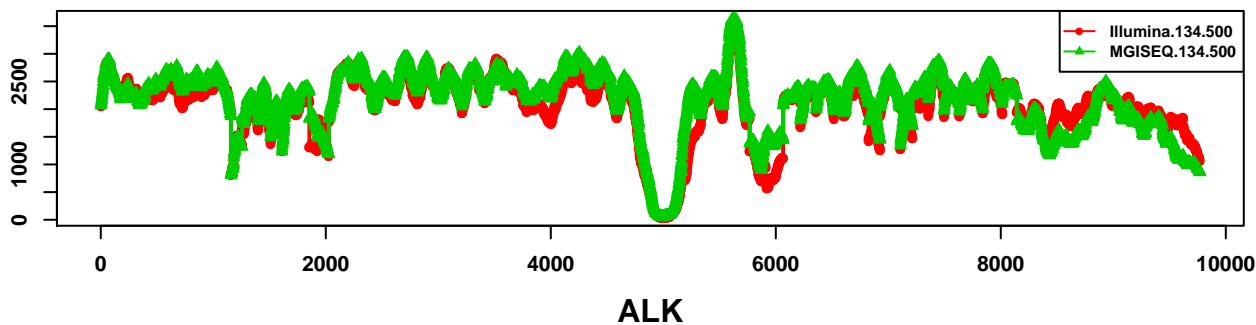

Supplement: Supplementary file 3 [file Presentation2.zip › ALK/19N01655P.pdf]

Sequencing Depth

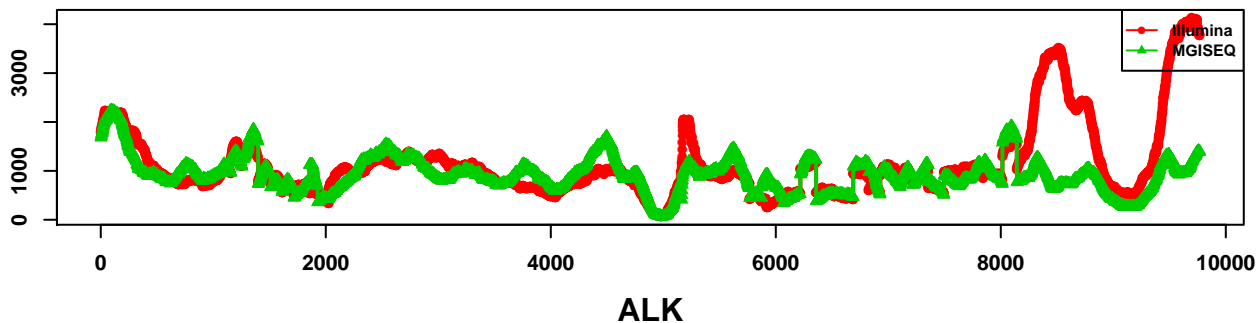

Sequencing Depth

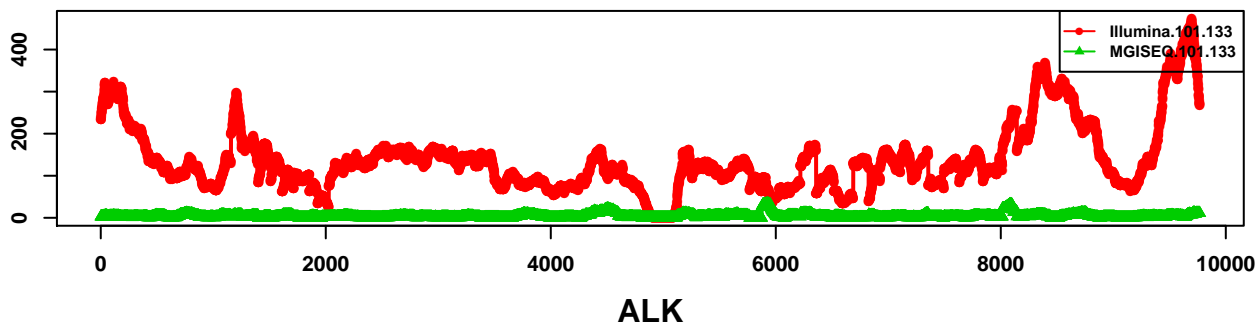

Sequencing Depth

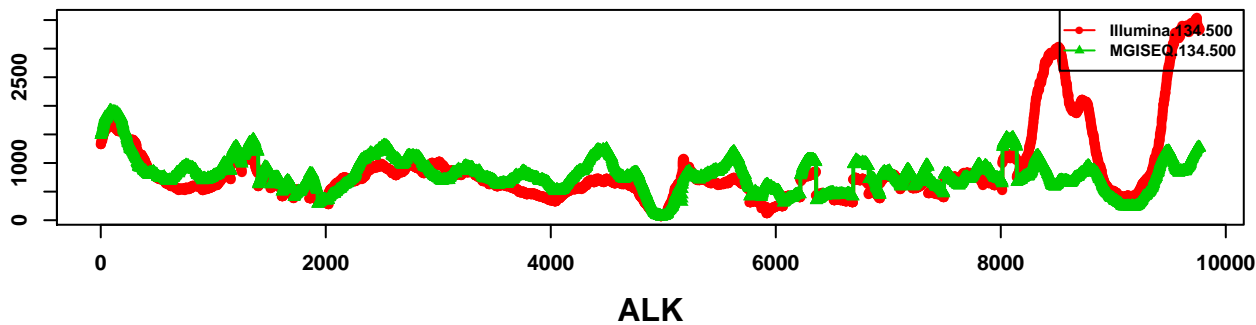

Supplement: Supplementary file 3 [file Presentation2.zip › ALK/19ZN13095F.pdf]

Sequencing Depth

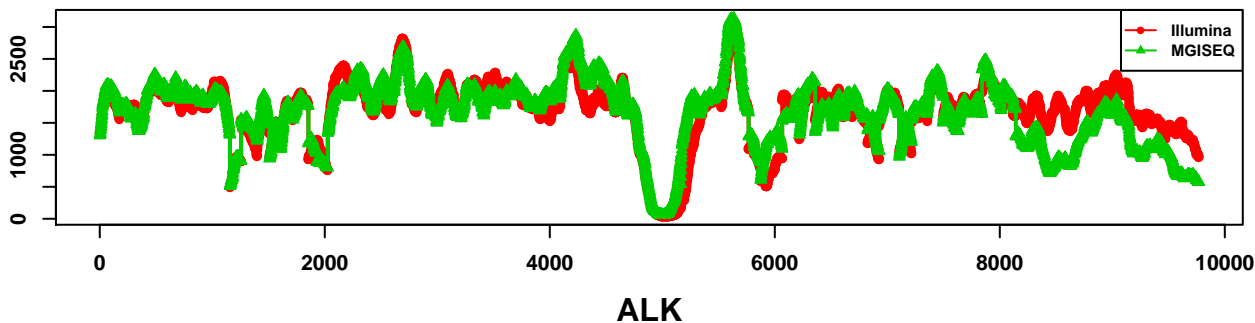

Sequencing Depth

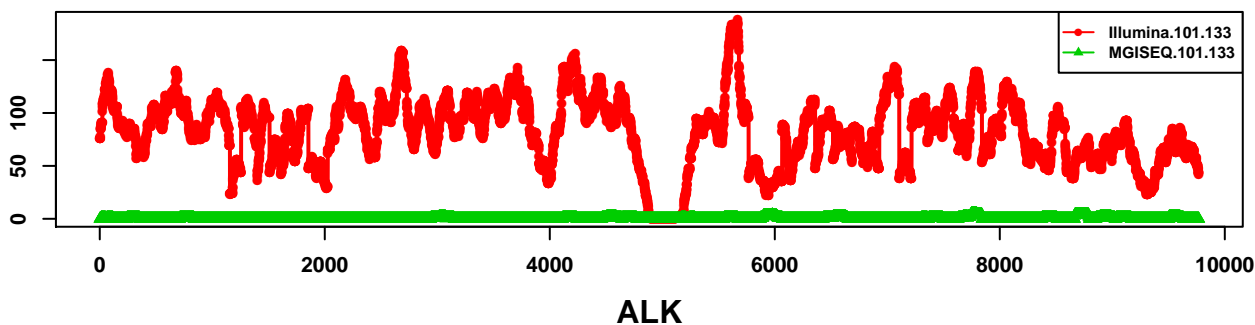

Sequencing Depth

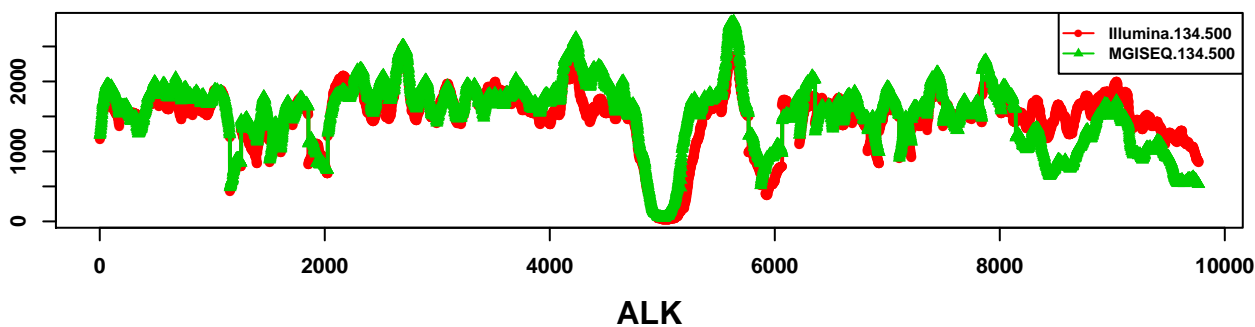

Supplement: Supplementary file 3 [file Presentation2.zip › ALK/ZK0604-CF.pdf]

Sequencing Depth

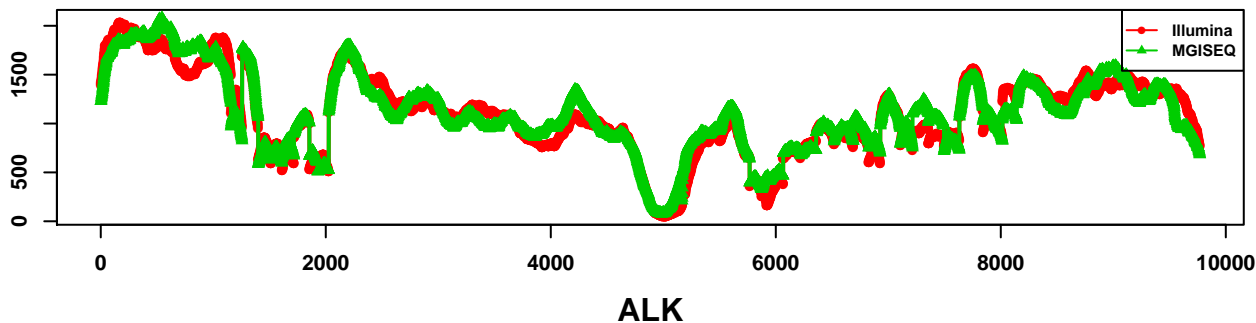

Sequencing Depth

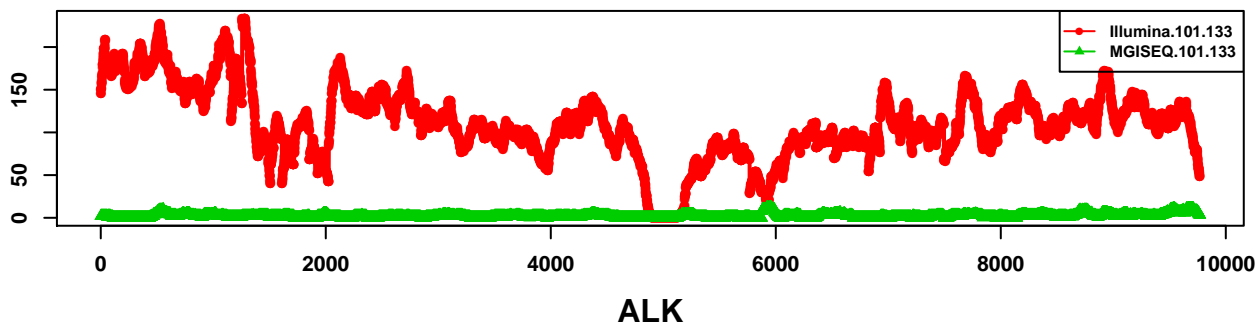

Sequencing Depth

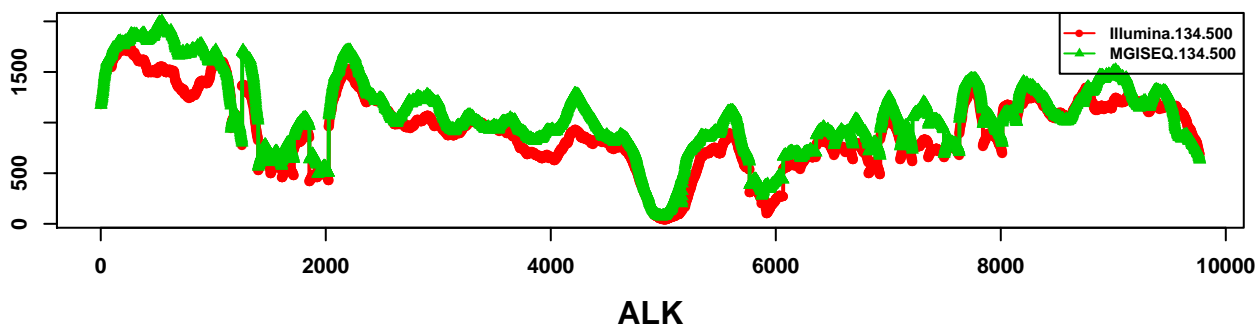

Supplement: Supplementary file 4 [file Presentation3.zip › ALK/19HE22109F.pdf]

Sequencing Depth

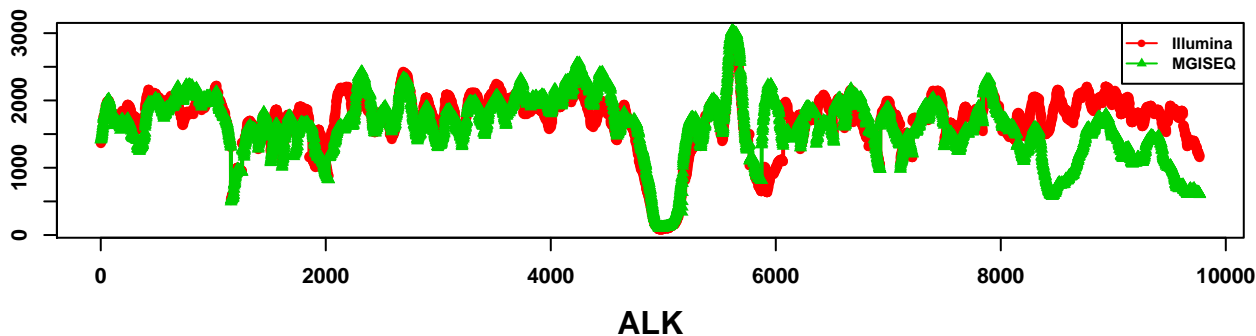

Sequencing Depth

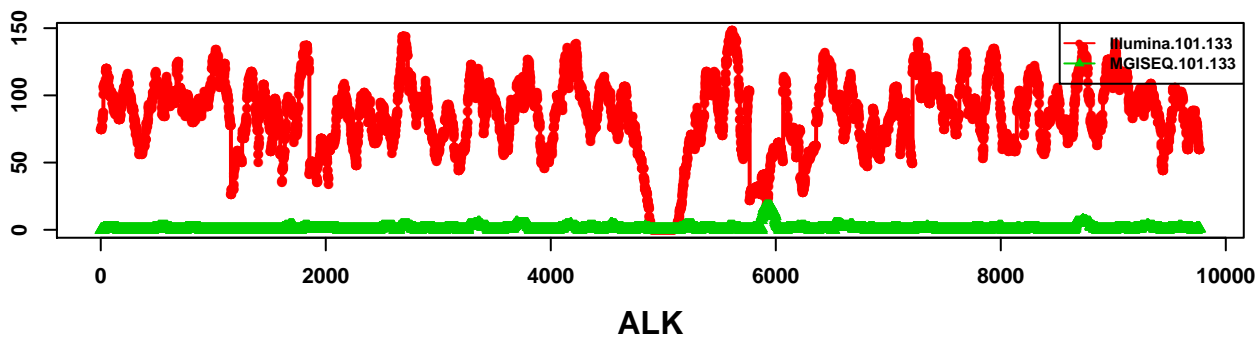

Sequencing Depth

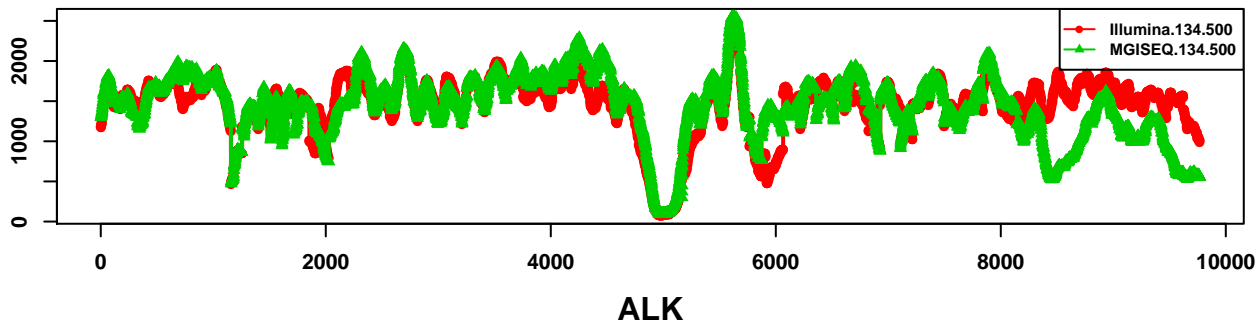

Supplement: Supplementary file 4 [file Presentation3.zip › ALK/19JM45590P.pdf]

Sequencing Depth

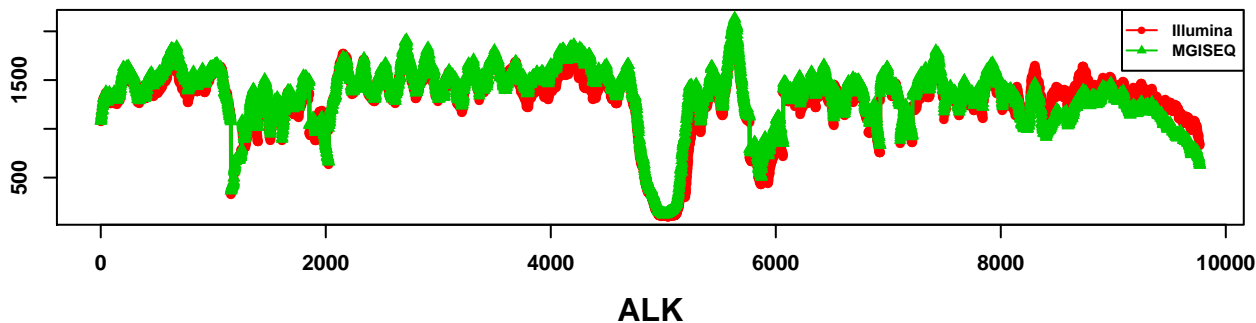

Sequencing Depth

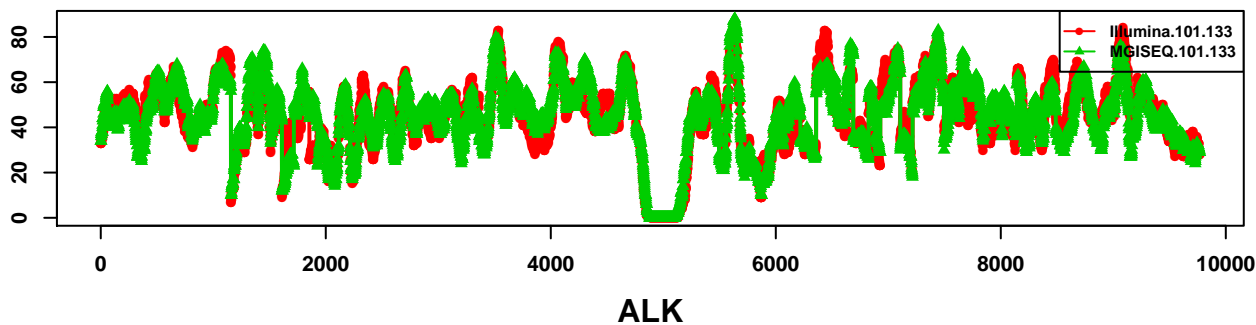

Sequencing Depth

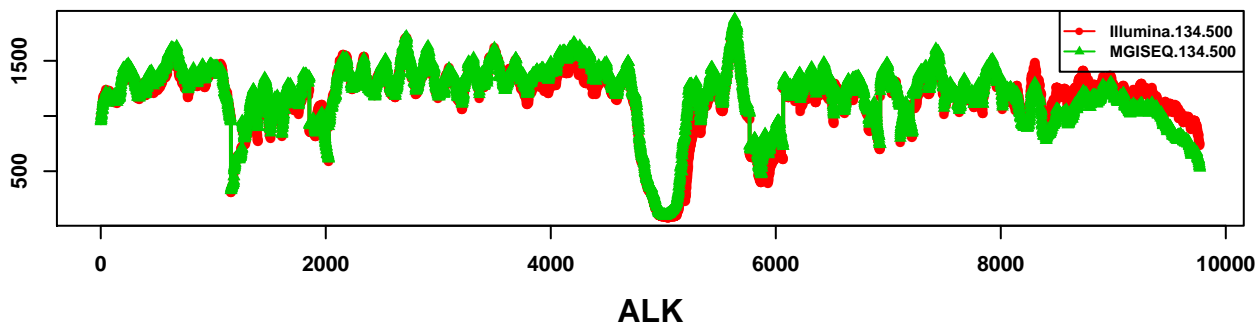

Supplement: Supplementary file 4 [file Presentation3.zip › ALK/19JS48248P.pdf]

Sequencing Depth

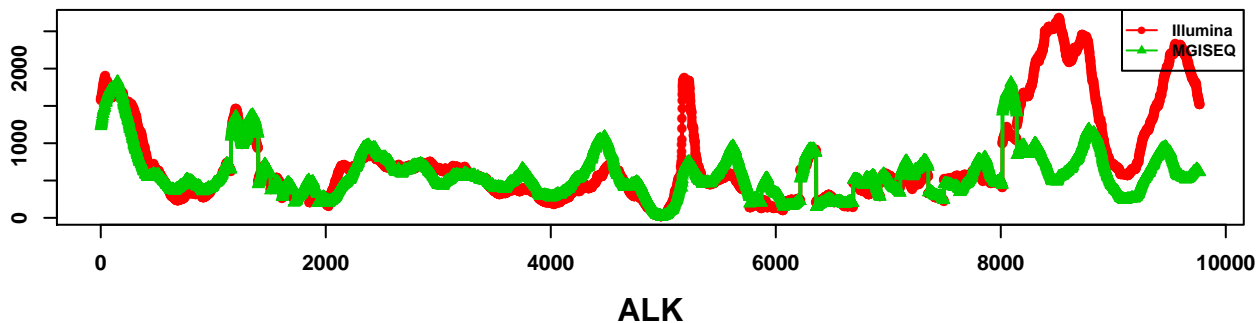

Sequencing Depth

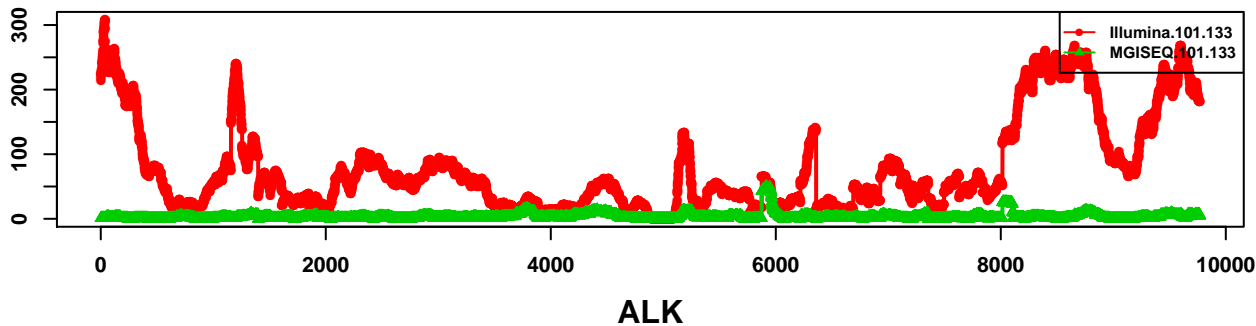

Sequencing Depth

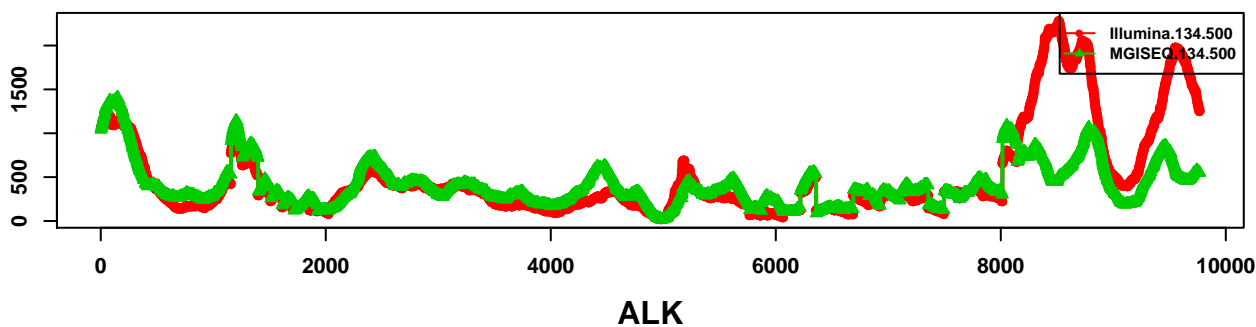

Supplement: Supplementary file 4 [file Presentation3.zip › ALK/19ZN12364F.pdf]

Sequencing Depth

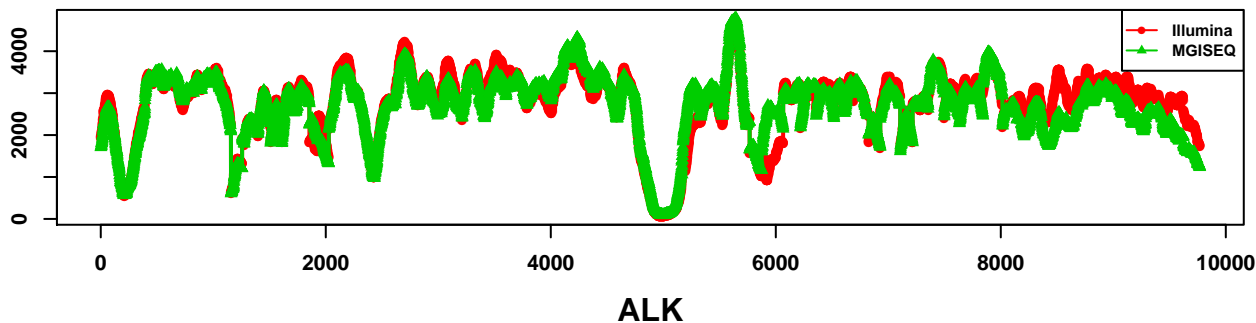

Sequencing Depth

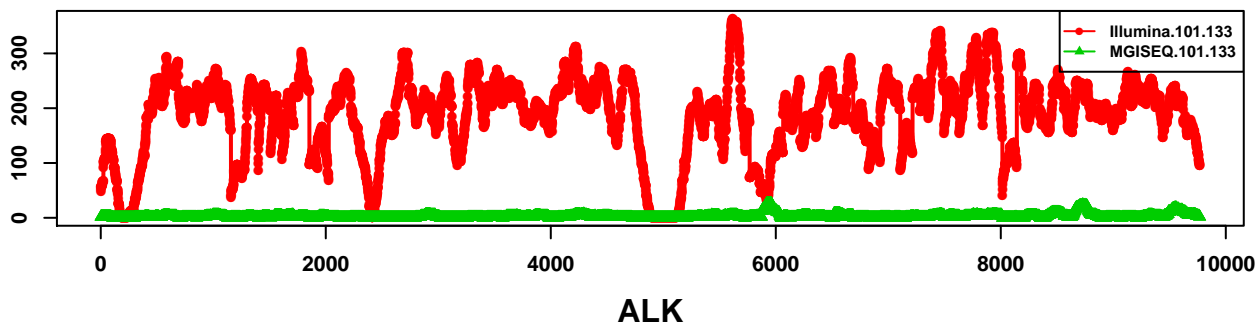

Sequencing Depth

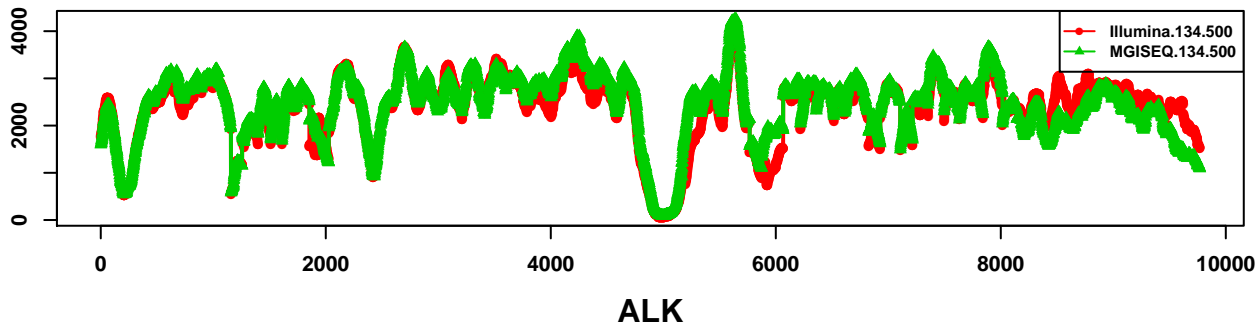

Supplement: Supplementary file 4 [file Presentation3.zip › ALK/19N01659P.pdf]

Sequencing Depth

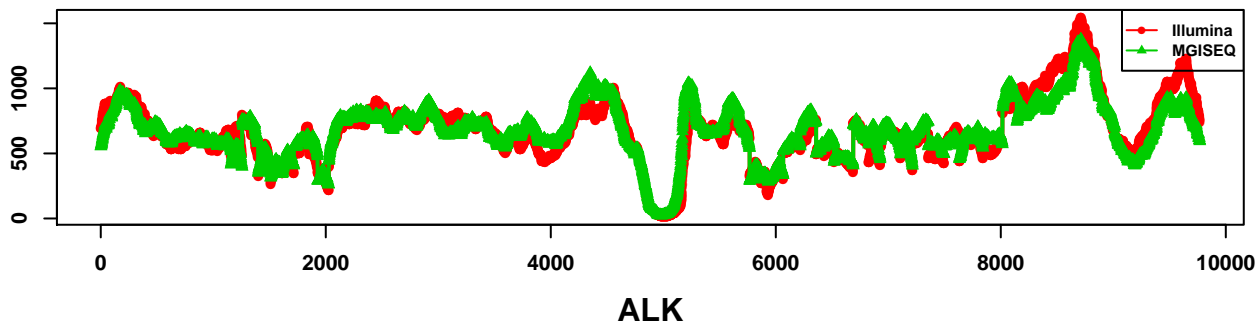

Sequencing Depth

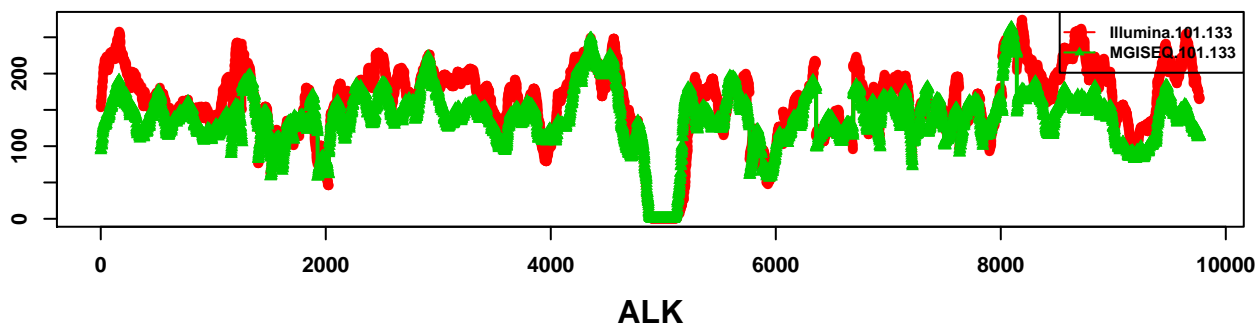

Sequencing Depth

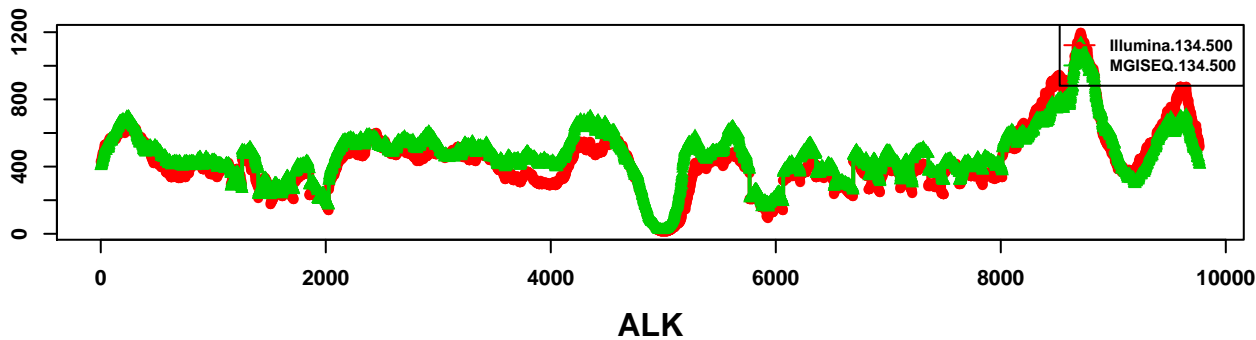

Supplement: Supplementary file 4 [file Presentation3.zip › ALK/19CF15714F.pdf]

Sequencing Depth

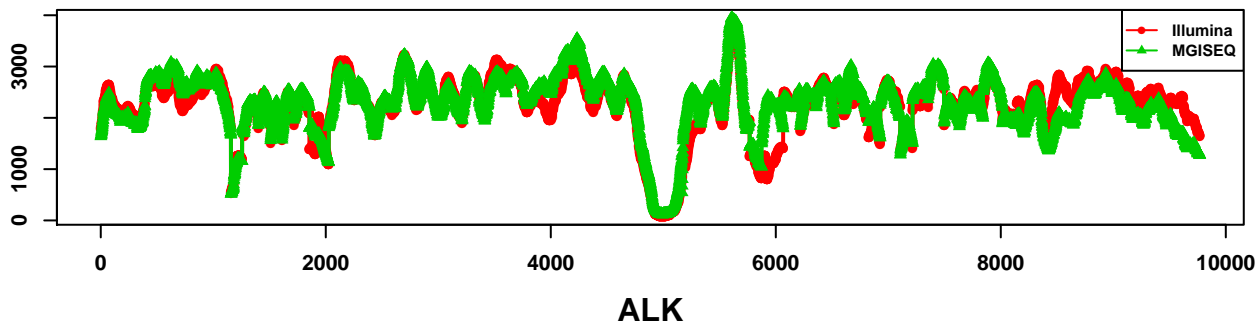

Sequencing Depth

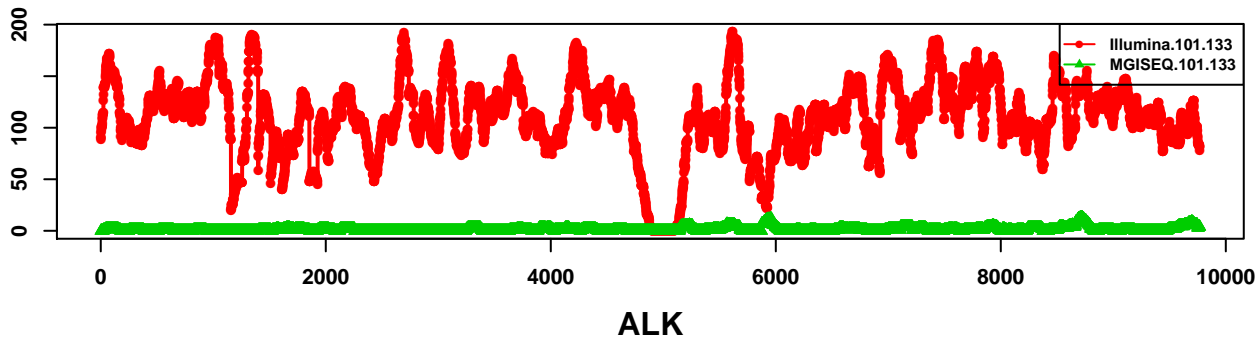

Sequencing Depth

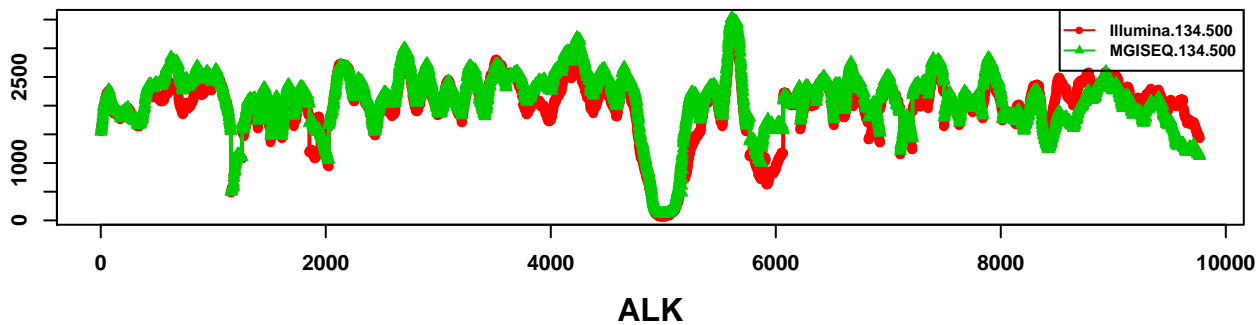

Supplement: Supplementary file 4 [file Presentation3.zip › ALK/19LN70345P.pdf]

Sequencing Depth

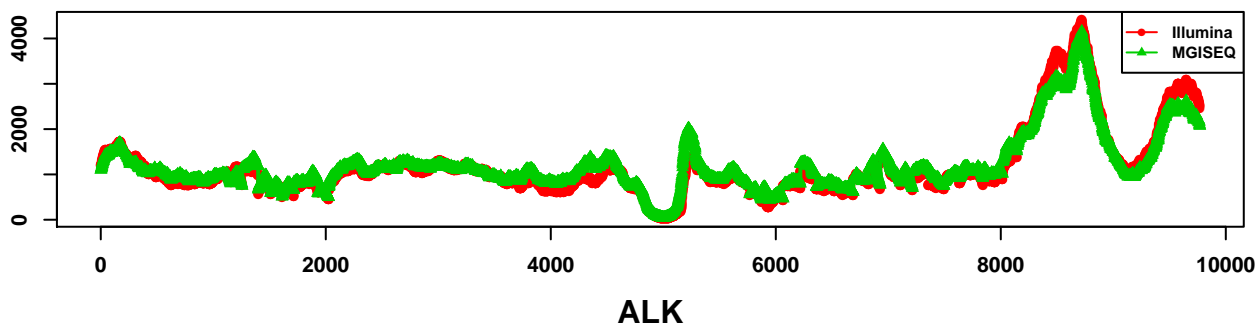

Sequencing Depth

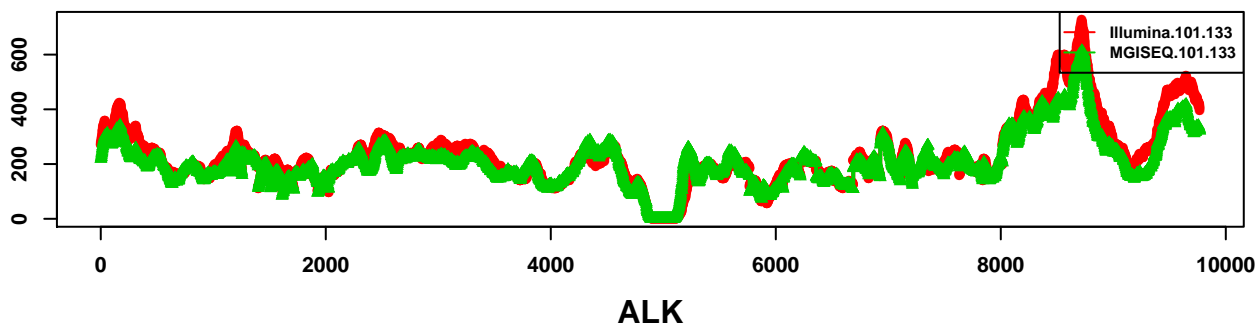

Sequencing Depth

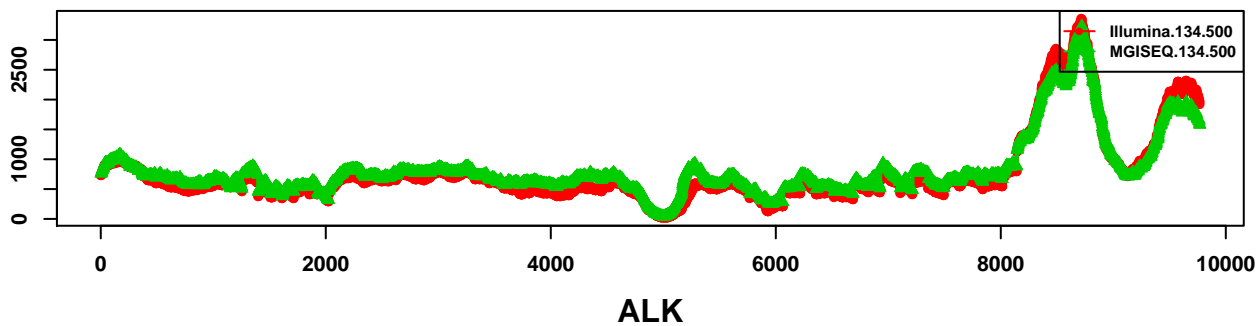

Supplement: Supplementary file 4 [file Presentation3.zip › ALK/19ZN12775F.pdf]

Sequencing Depth

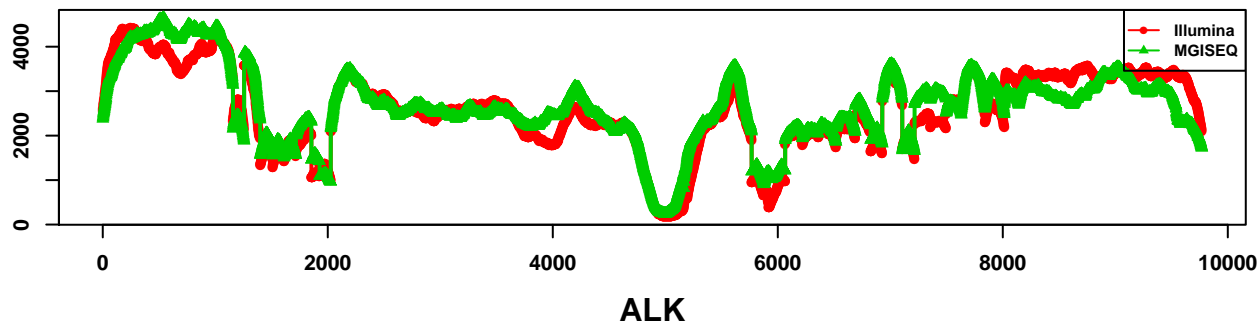

Sequencing Depth

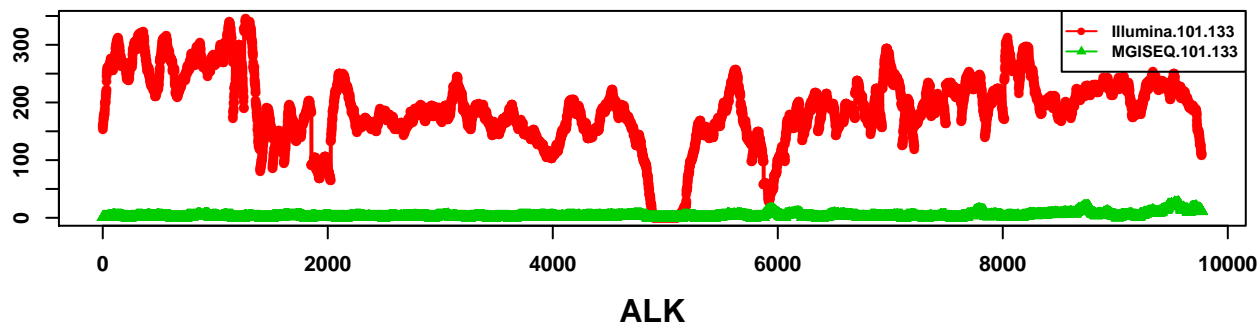

Sequencing Depth

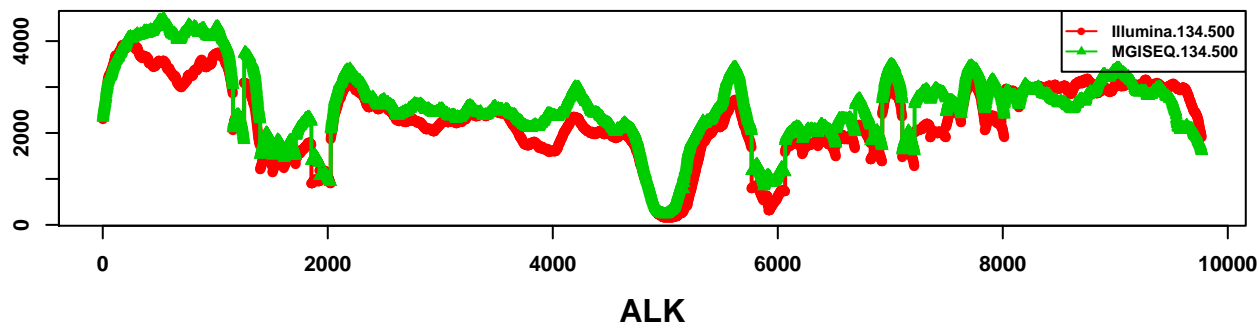

Supplement: Supplementary file 4 [file Presentation3.zip › ALK/19HE22223F.pdf]

Sequencing Depth

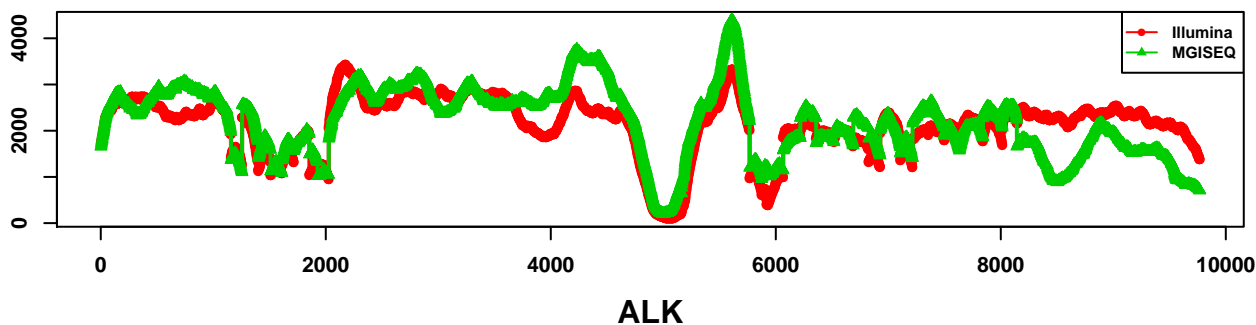

Sequencing Depth

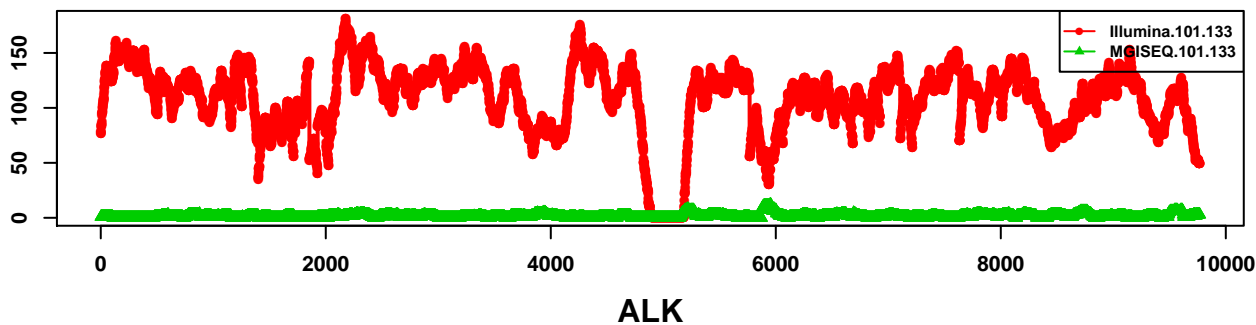

Sequencing Depth

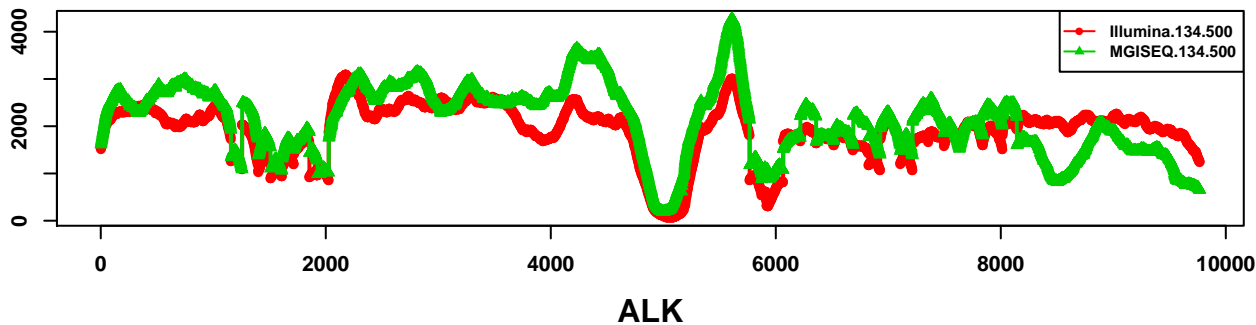

Supplement: Supplementary file 4 [file Presentation3.zip › ALK/19N01661T.pdf]

Sequencing Depth

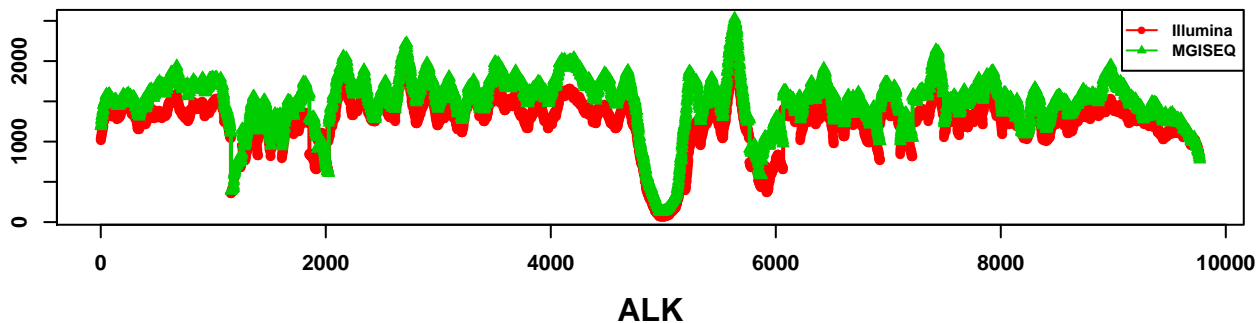

Sequencing Depth

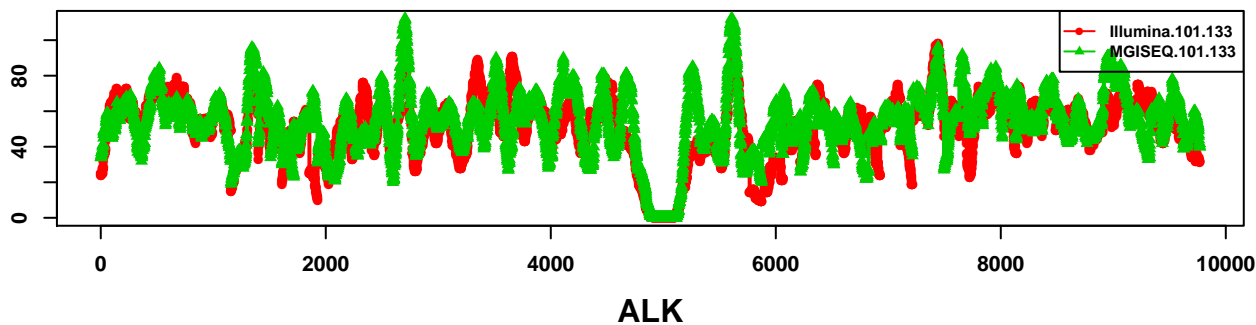

Sequencing Depth

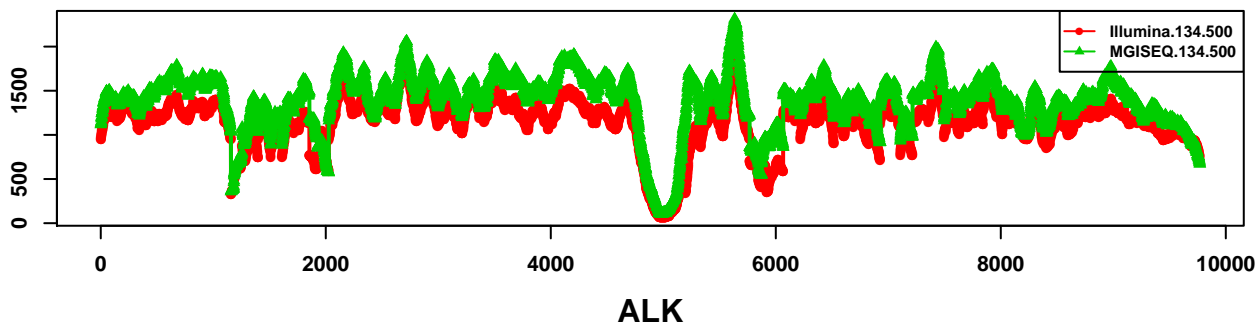

Supplement: Supplementary file 4 [file Presentation3.zip › ALK/19ZN13999P.pdf]

Sequencing Depth

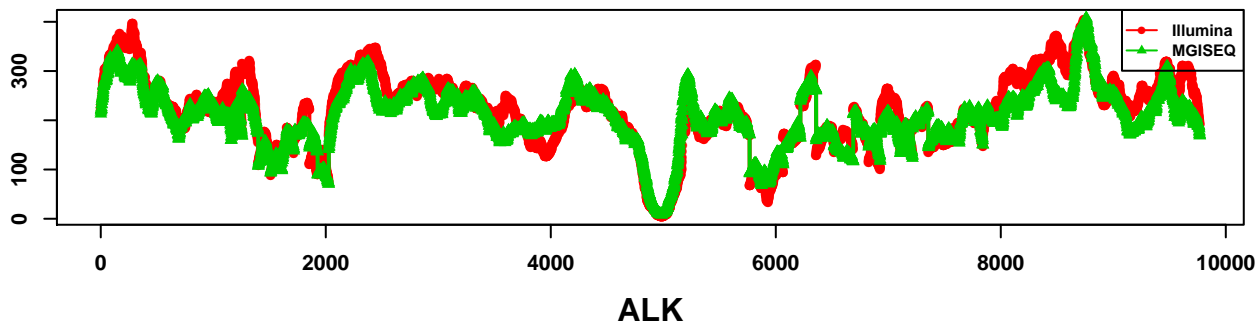

Sequencing Depth

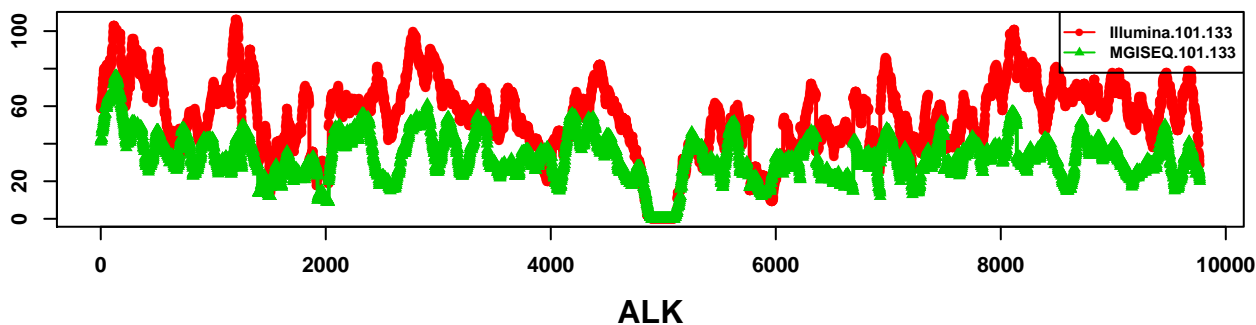

Sequencing Depth

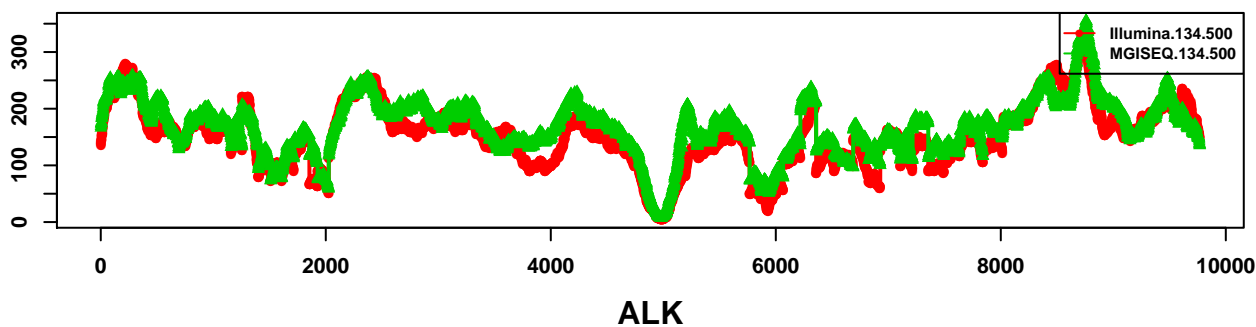

Supplement: Supplementary file 4 [file Presentation3.zip › ALK/19N01973F.pdf]

Sequencing Depth

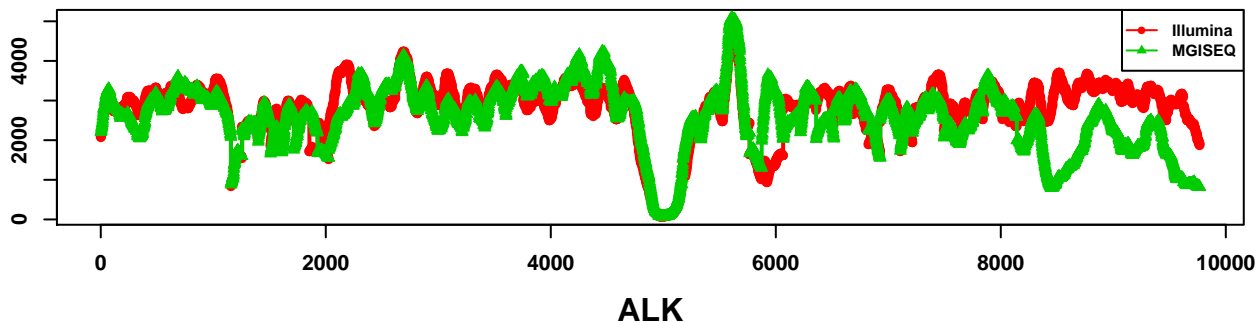

Sequencing Depth

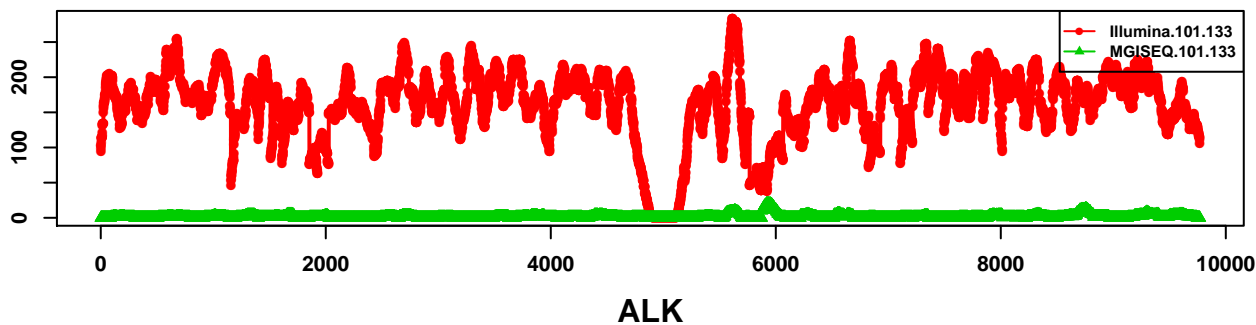

Sequencing Depth

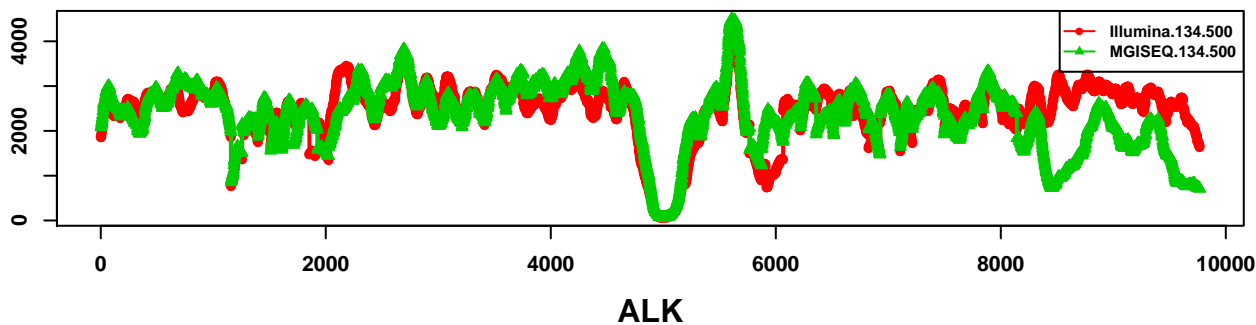

Supplement: Supplementary file 4 [file Presentation3.zip › ALK/19HS86151P.pdf]

Sequencing Depth

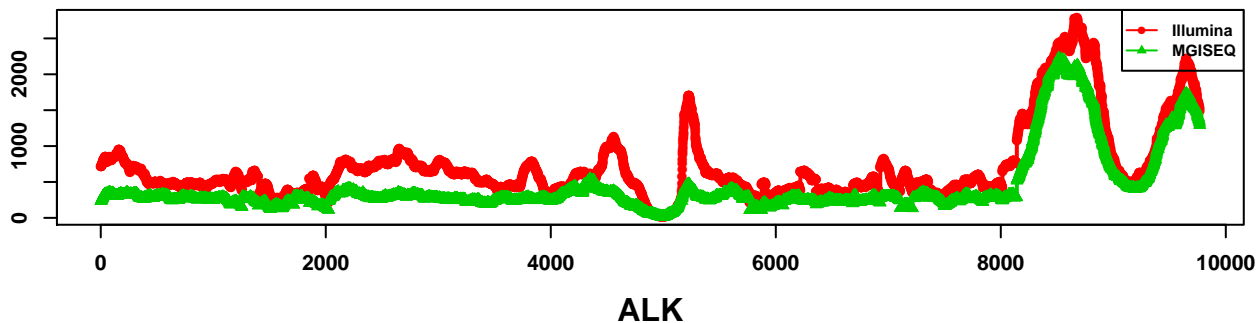

Sequencing Depth

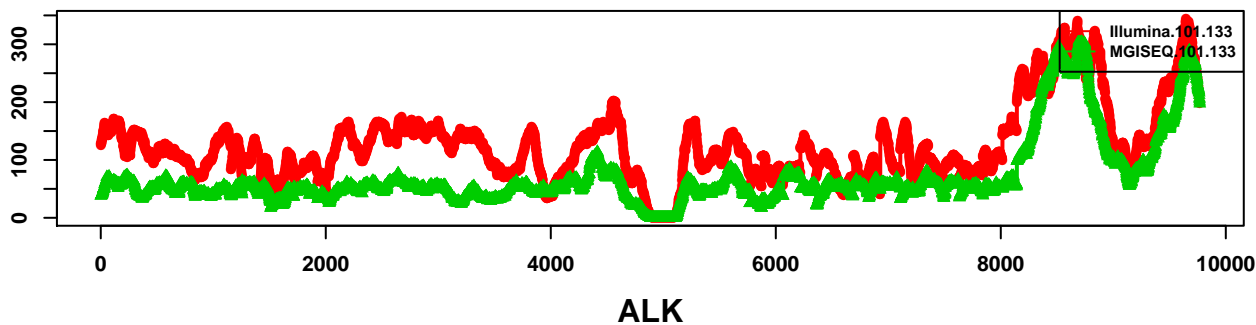

Sequencing Depth

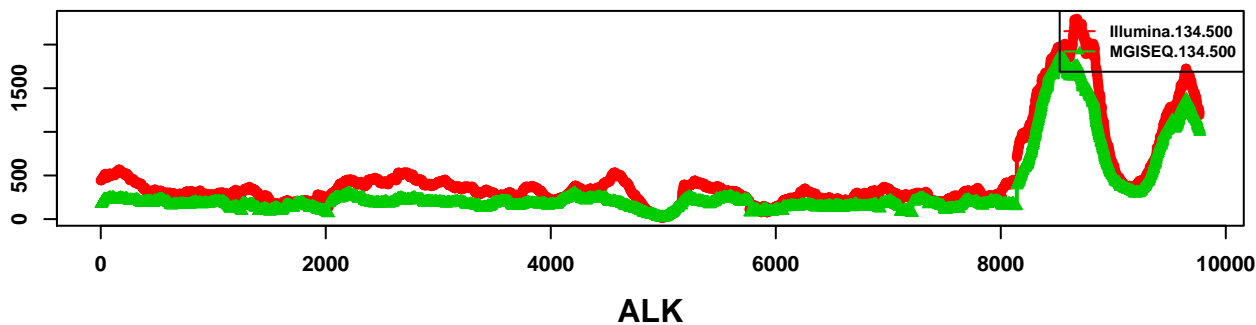

Supplement: Supplementary file 4 [file Presentation3.zip › ALK/19ZN12577F.pdf]

Sequencing Depth

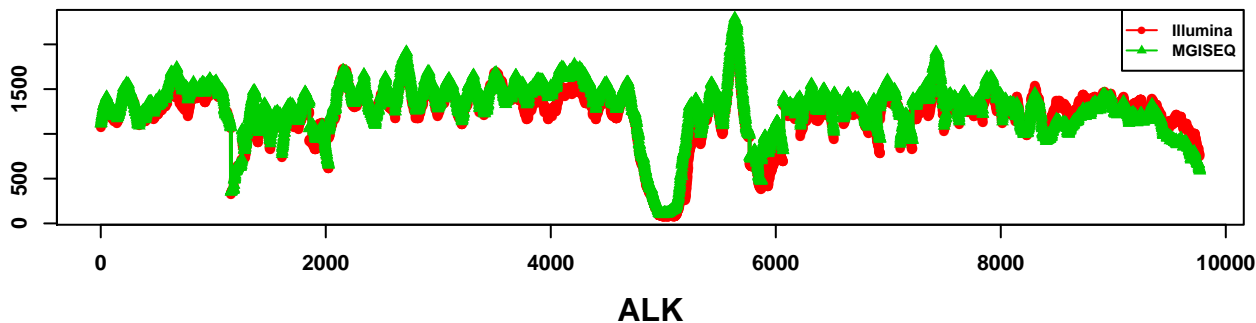

Sequencing Depth

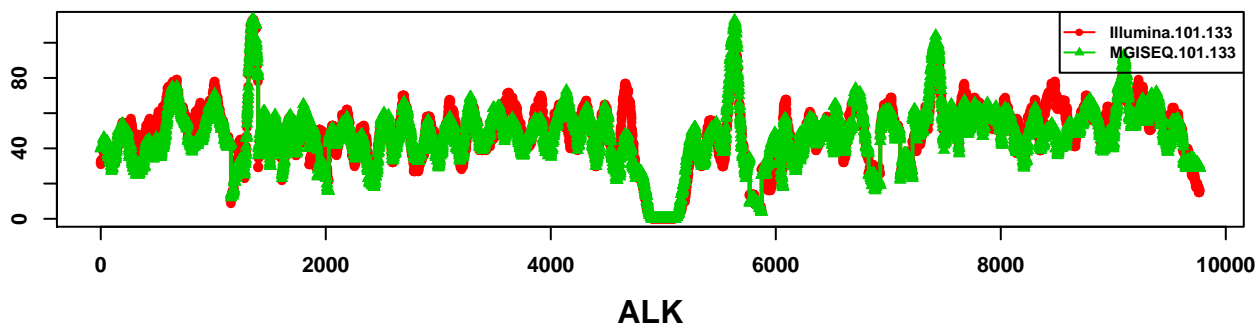

Sequencing Depth

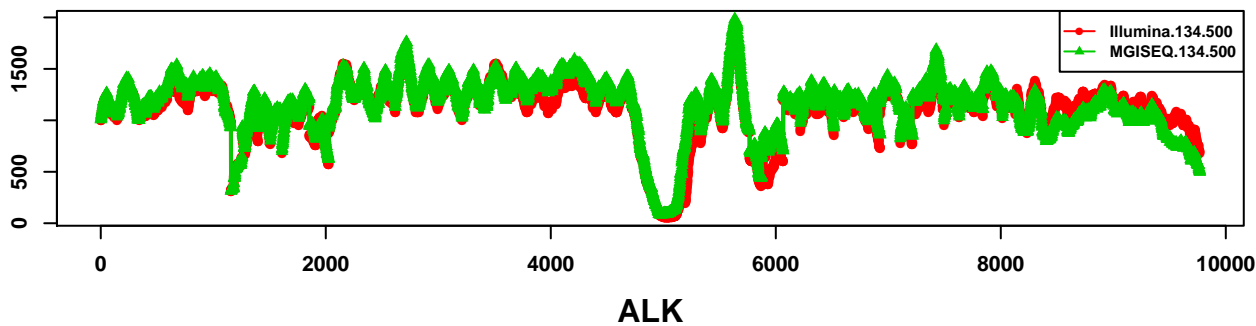

Supplement: Supplementary file 4 [file Presentation3.zip › ALK/19ZQ13269P.pdf]

Sequencing Depth

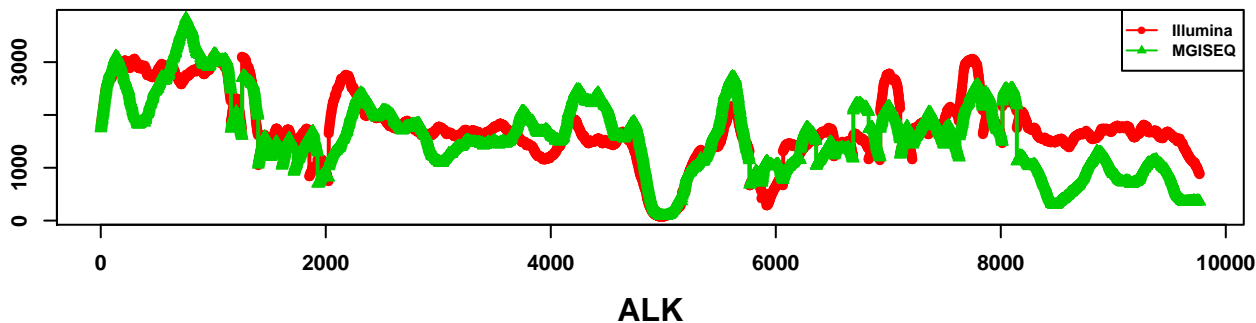

Sequencing Depth

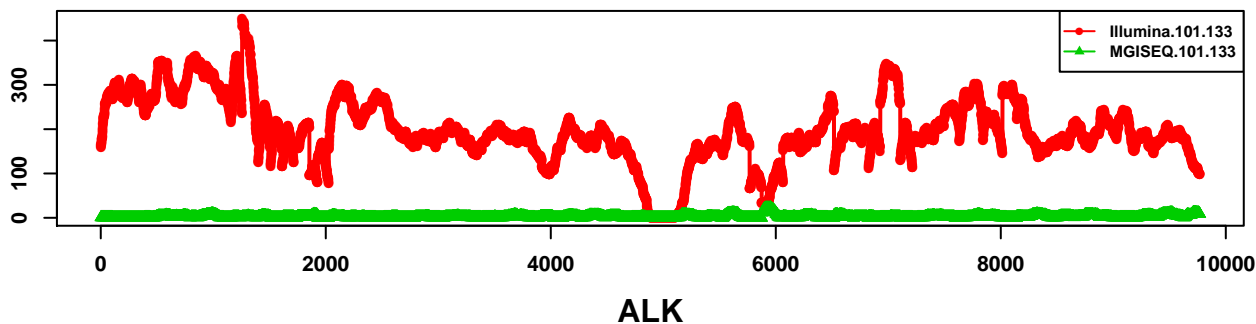

Sequencing Depth

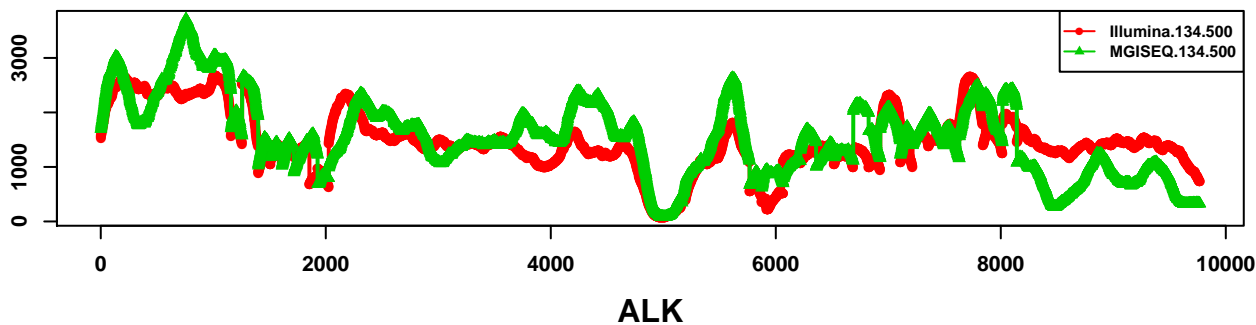

Supplement: Supplementary file 4 [file Presentation3.zip › ALK/19FC40256F.pdf]

Sequencing Depth

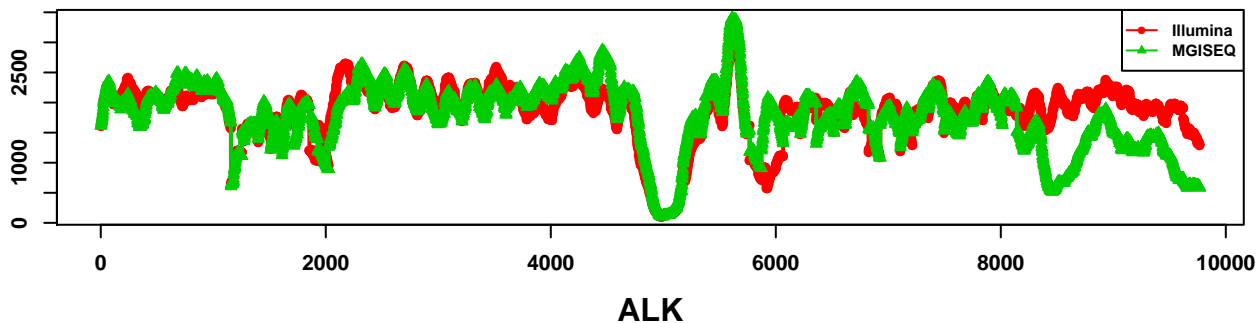

Sequencing Depth

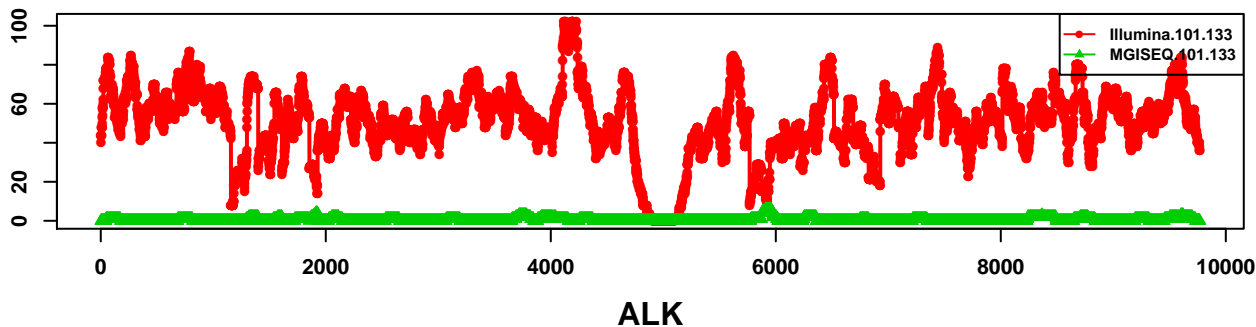

Sequencing Depth

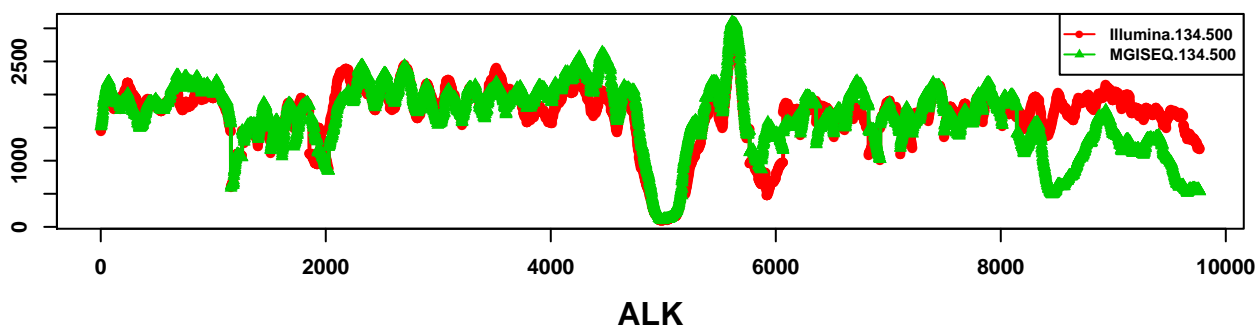

Supplement: Supplementary file 4 [file Presentation3.zip › ALK/19HS86080P.pdf]

Sequencing Depth

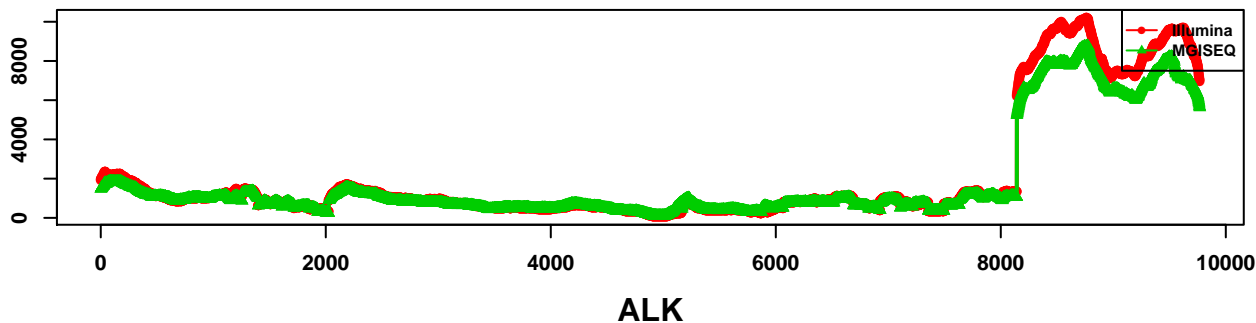

Sequencing Depth

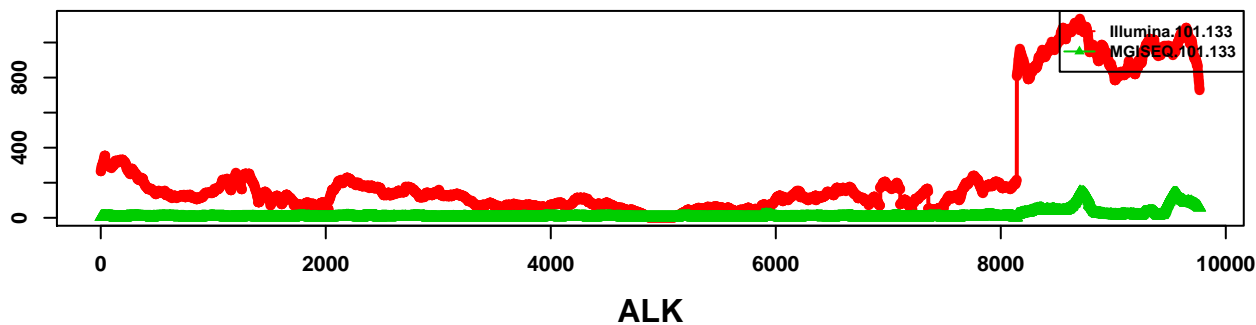

Sequencing Depth

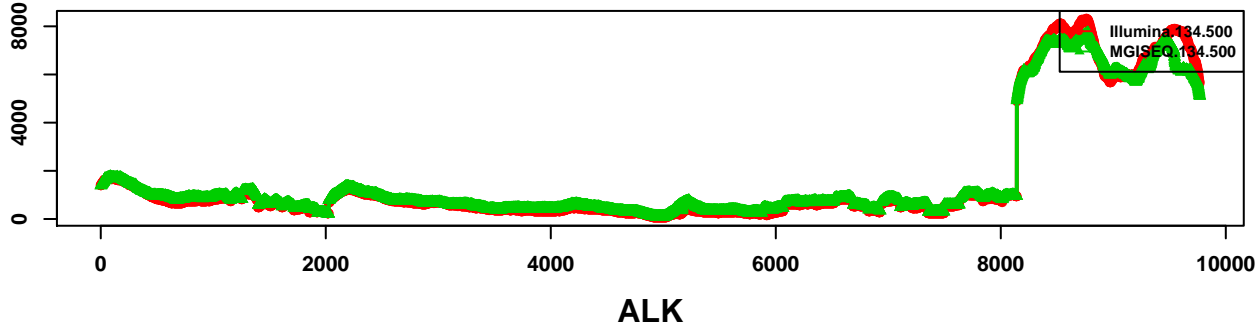

Supplement: Supplementary file 4 [file Presentation3.zip › ALK/19N01949F.pdf]

Sequencing Depth

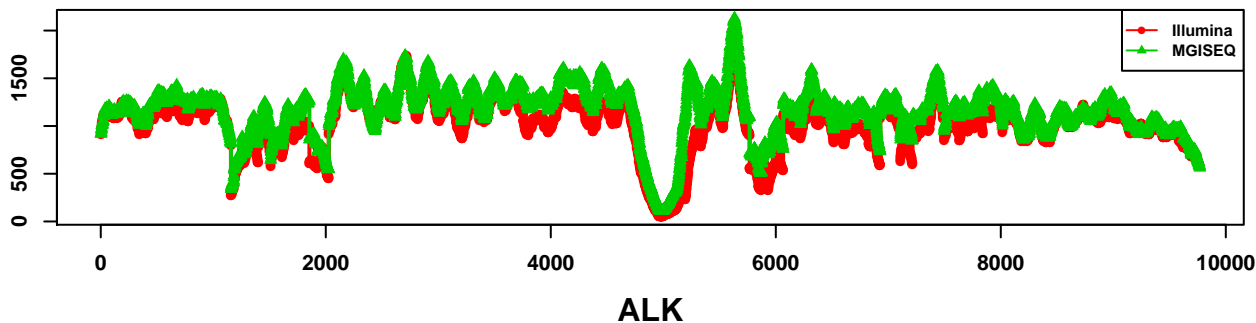

Sequencing Depth

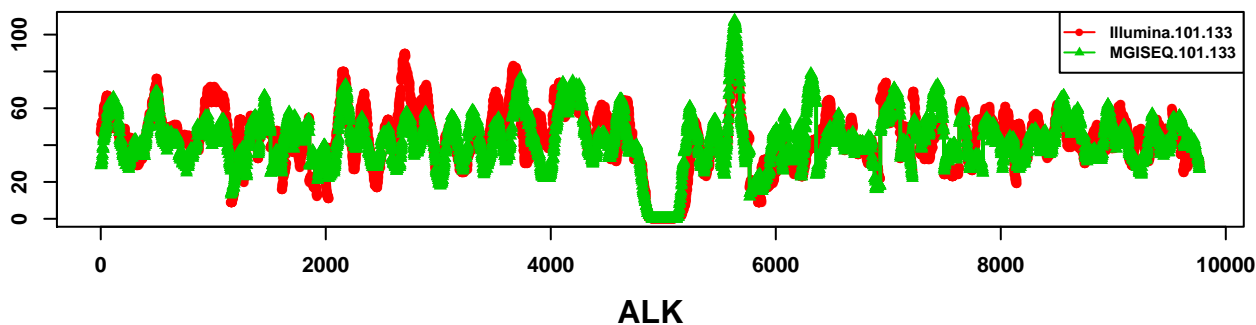

Sequencing Depth

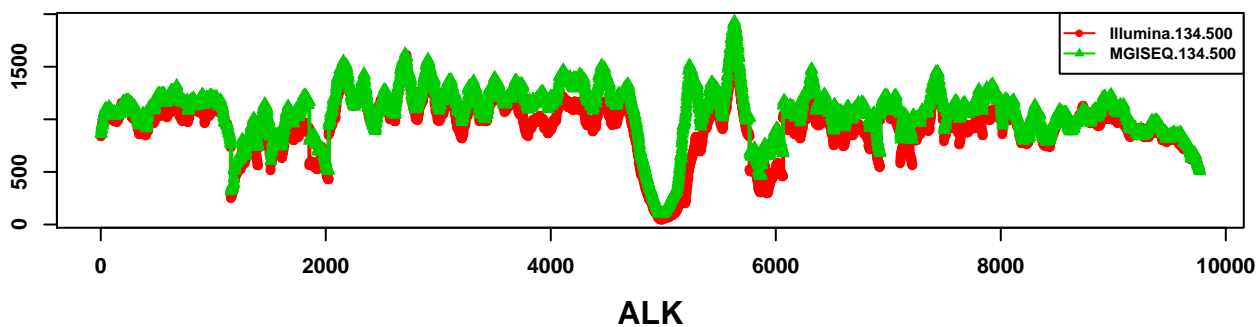

Supplement: Supplementary file 4 [file Presentation3.zip › ALK/19ZN12259P.pdf]

Sequencing Depth

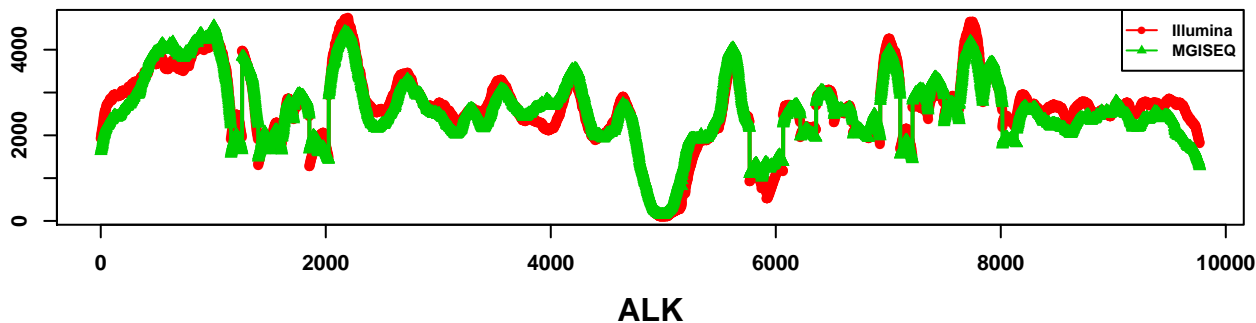

Sequencing Depth

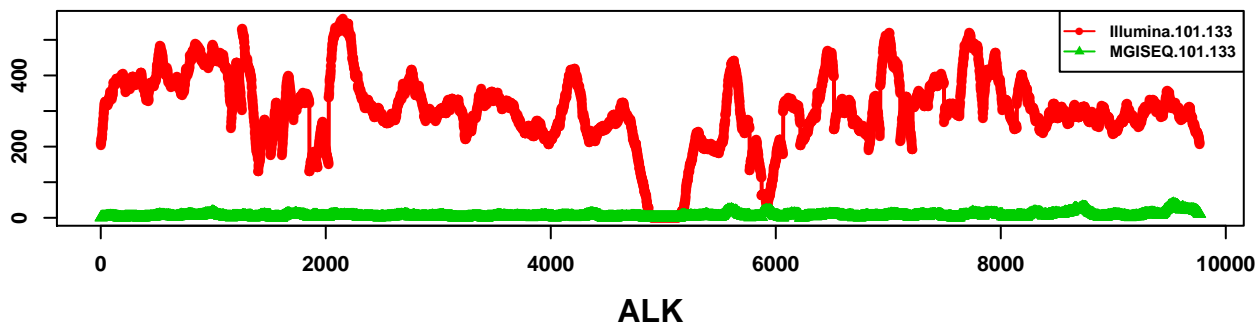

Sequencing Depth

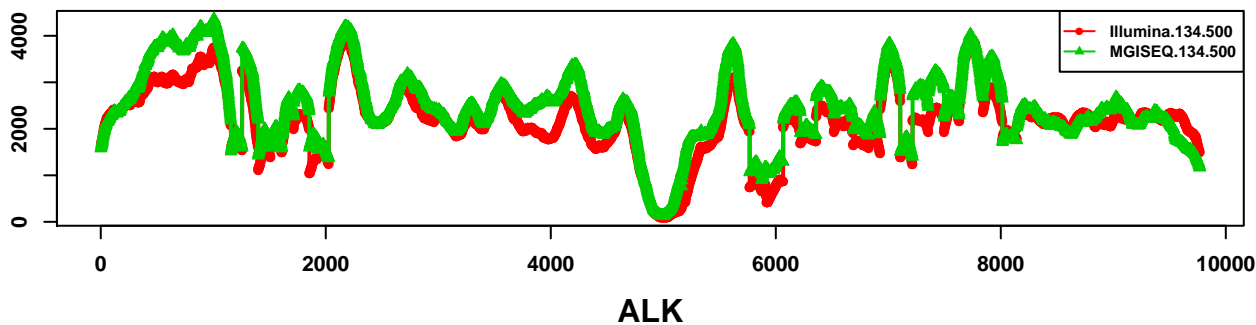

Supplement: Supplementary file 4 [file Presentation3.zip › ALK/19ZN12367F.pdf]

Sequencing Depth

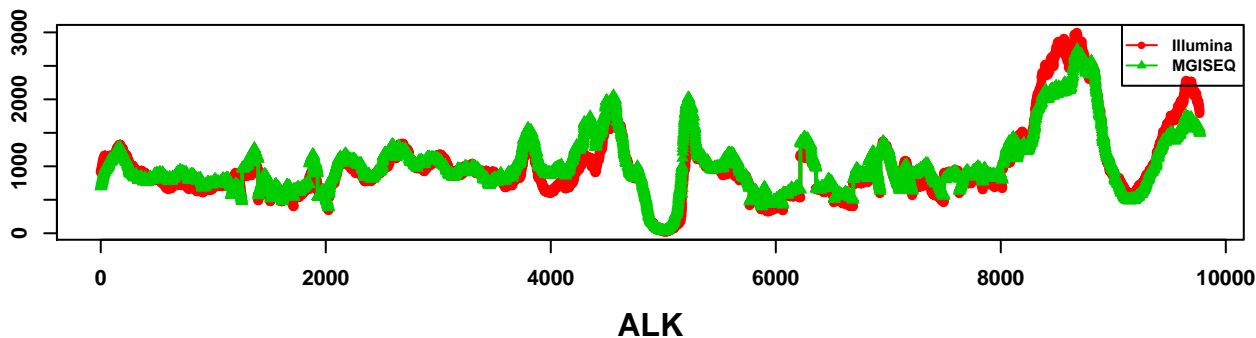

Sequencing Depth

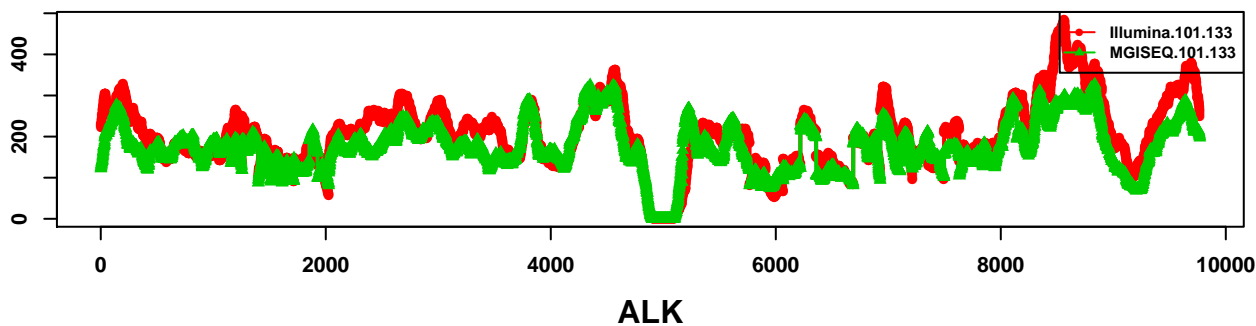

Sequencing Depth

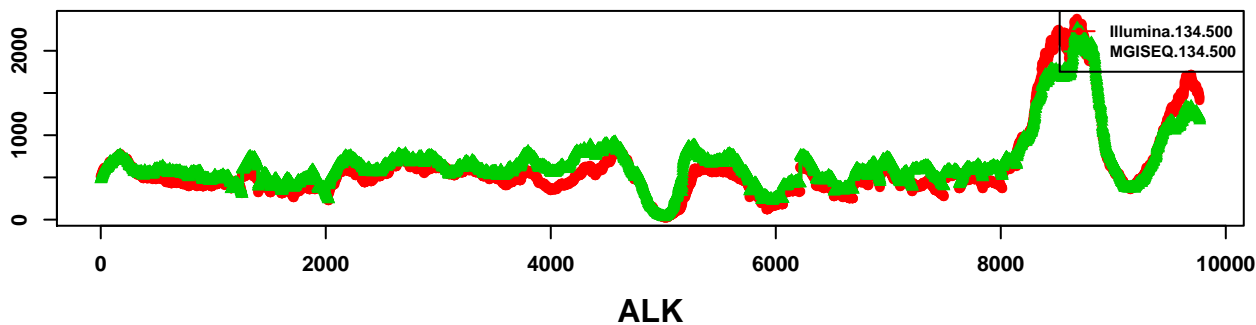

Supplement: Supplementary file 4 [file Presentation3.zip › ALK/19N02332F.pdf]

Sequencing Depth

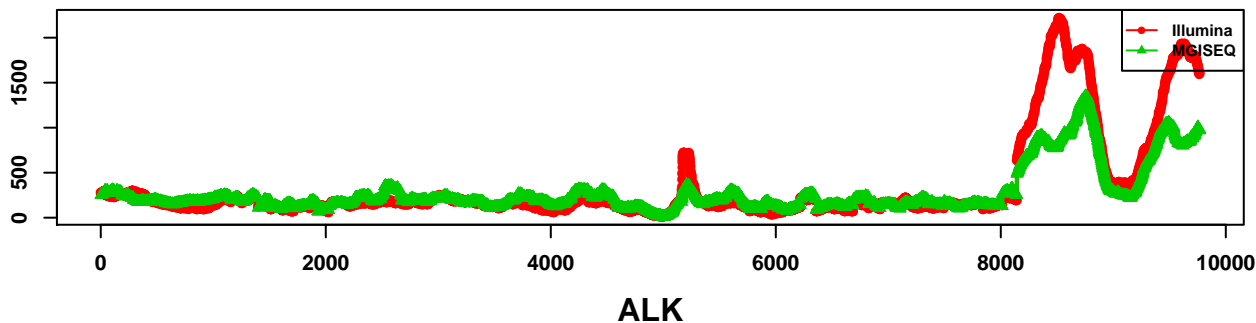

Sequencing Depth

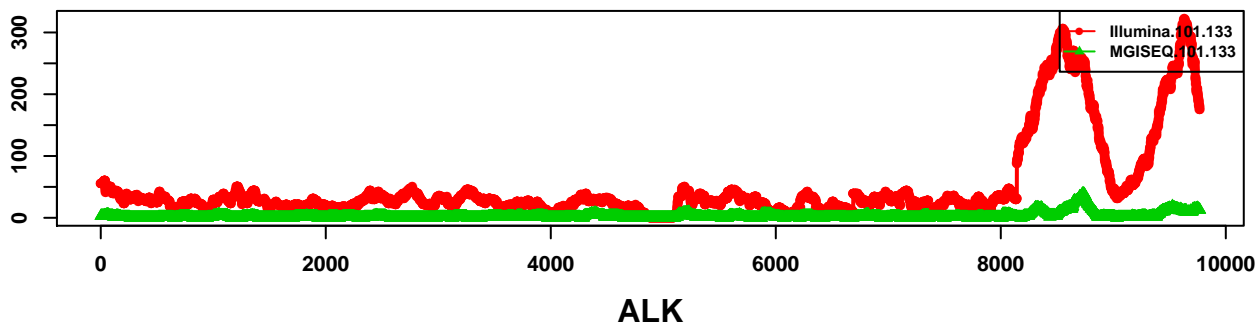

Sequencing Depth

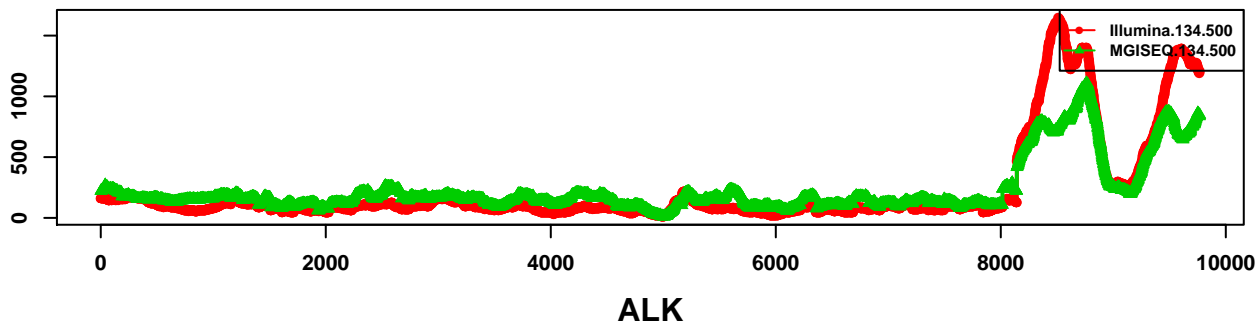

Supplement: Supplementary file 4 [file Presentation3.zip › ALK/19ZN12371F.pdf]

Sequencing Depth

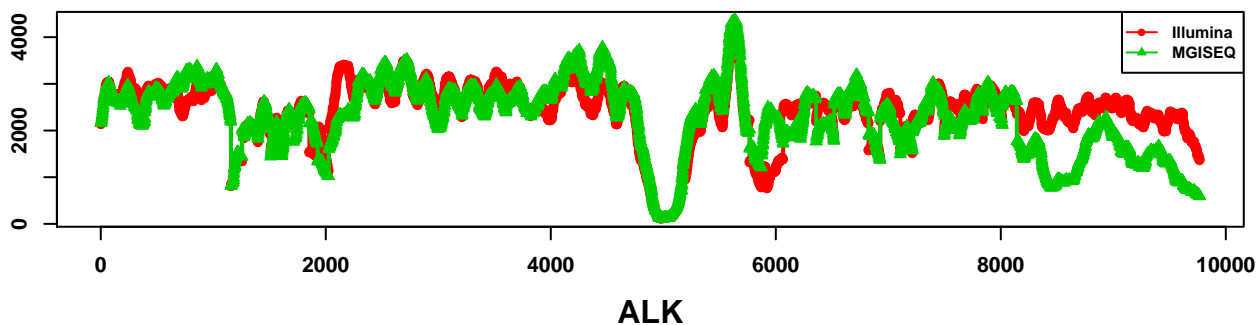

Sequencing Depth

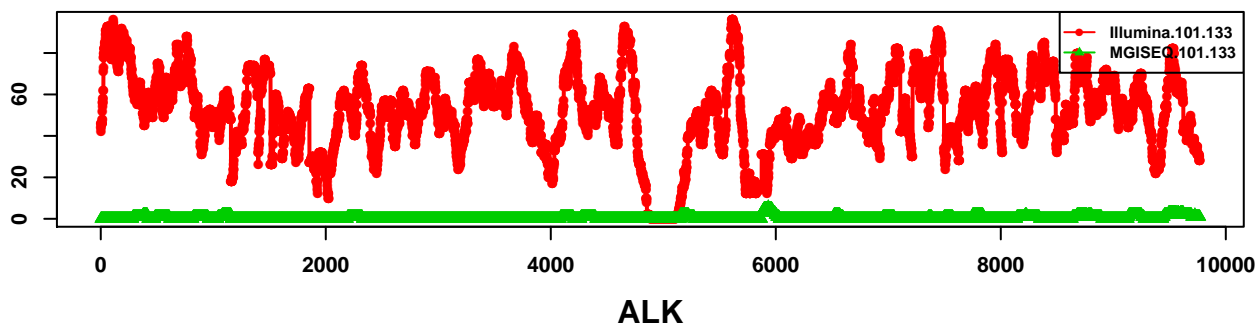

Sequencing Depth

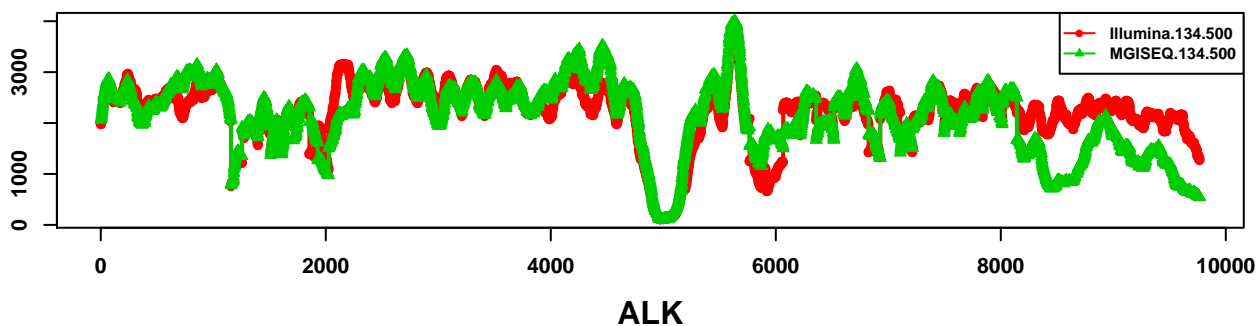

Supplement: Supplementary file 4 [file Presentation3.zip › ALK/19HS86079P.pdf]

Sequencing Depth

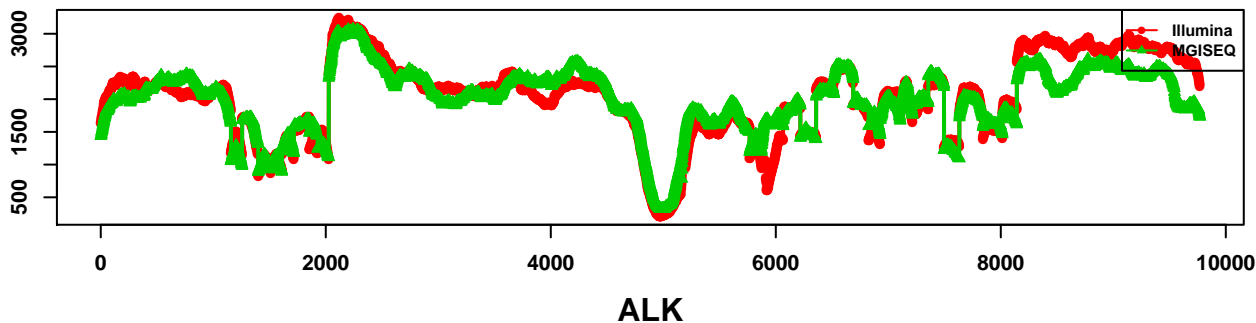

Sequencing Depth

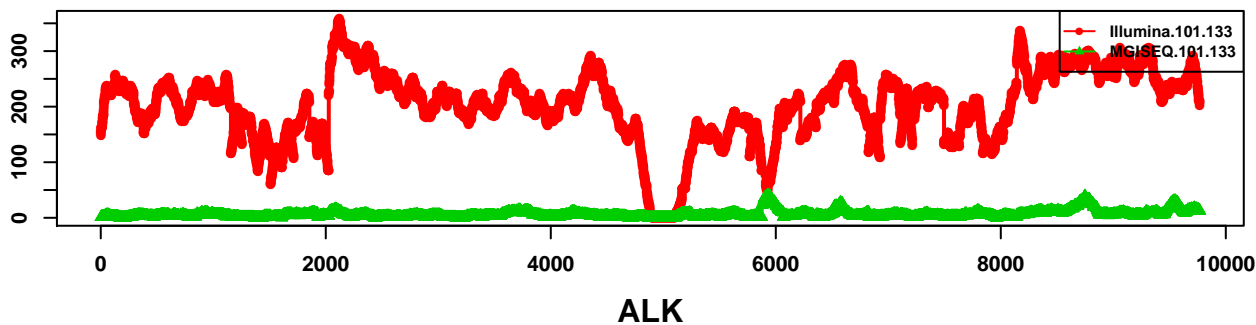

Sequencing Depth

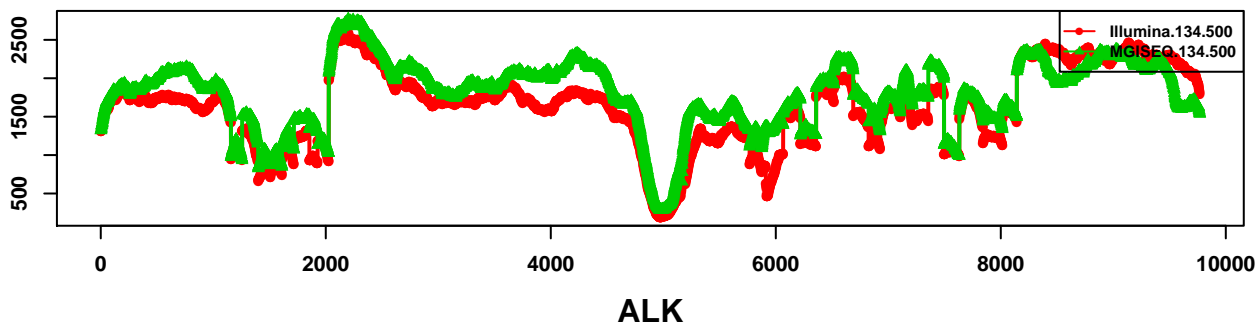

Supplement: Supplementary file 4 [file Presentation3.zip › ALK/19N01660F.pdf]

Sequencing Depth

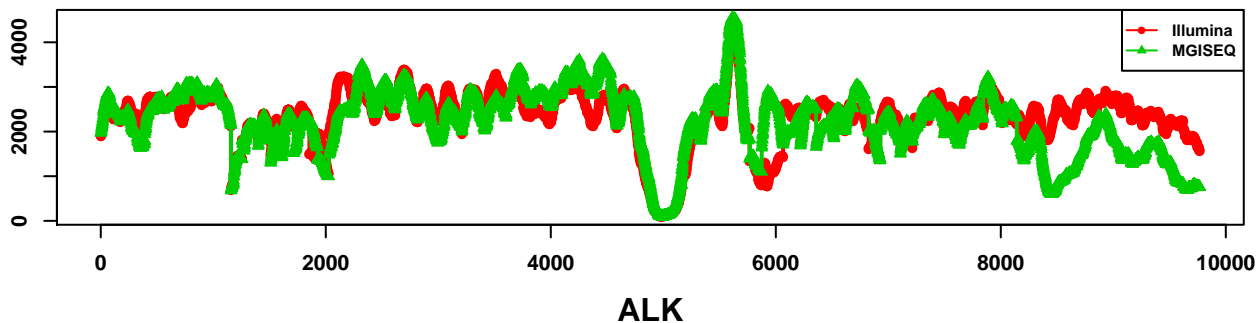

Sequencing Depth

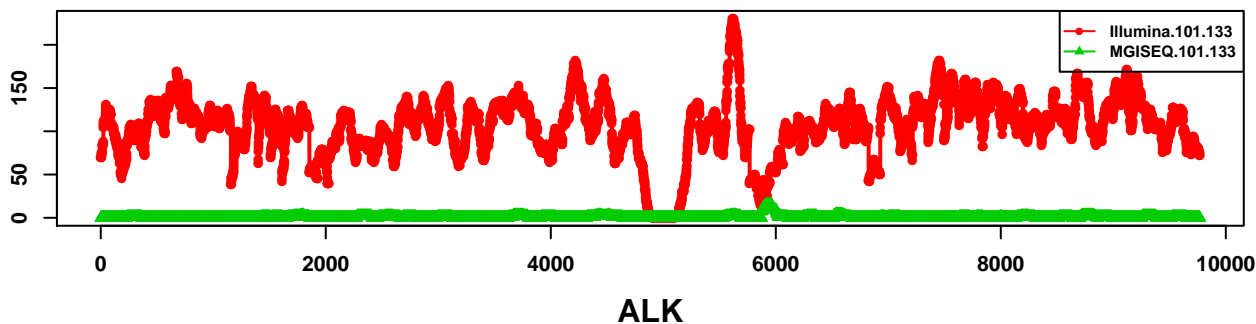

Sequencing Depth

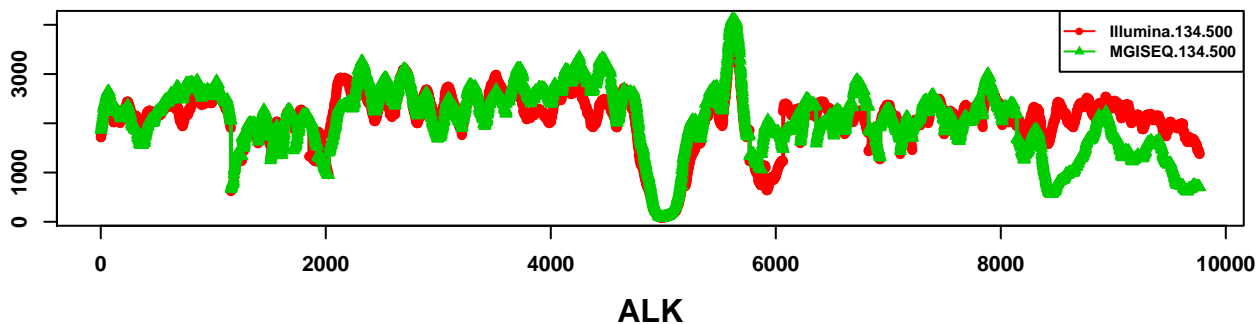

Supplement: Supplementary file 4 [file Presentation3.zip › ALK/19GY94045P.pdf]

Sequencing Depth

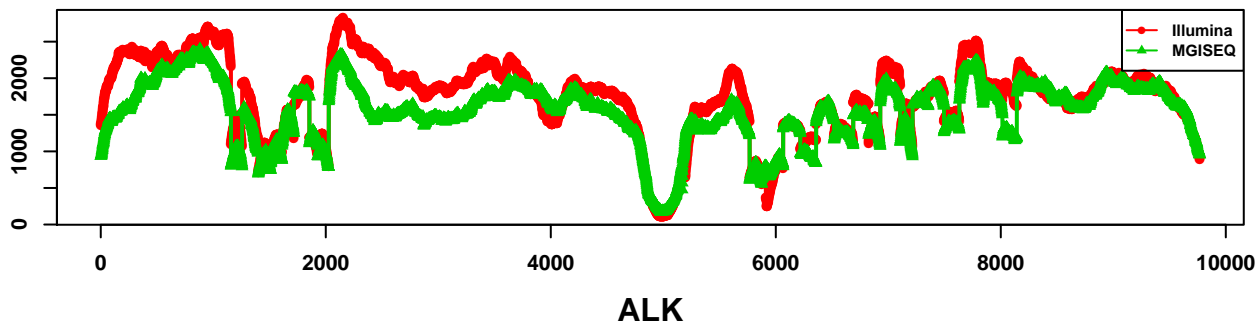

Sequencing Depth

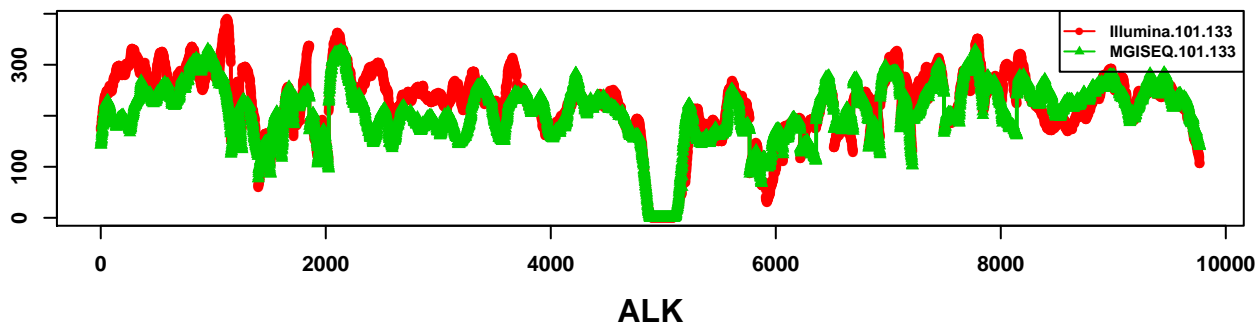

Sequencing Depth

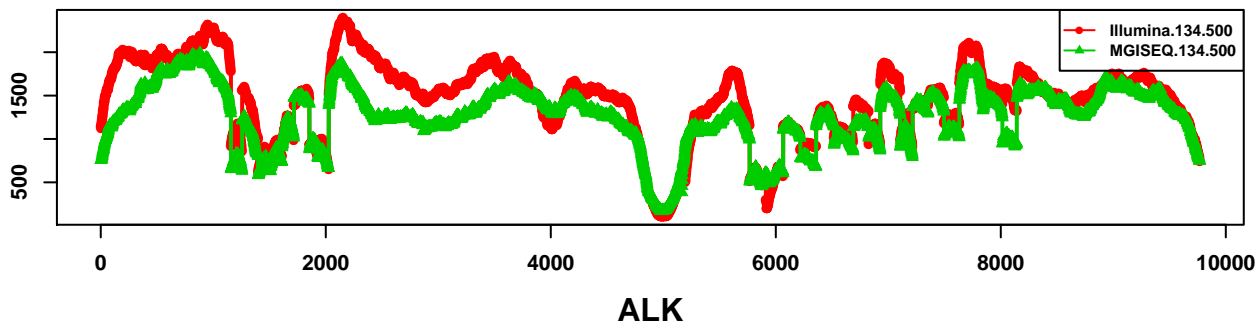

Supplement: Supplementary file 4 [file Presentation3.zip › ALK/19HE22805F.pdf]

Sequencing Depth

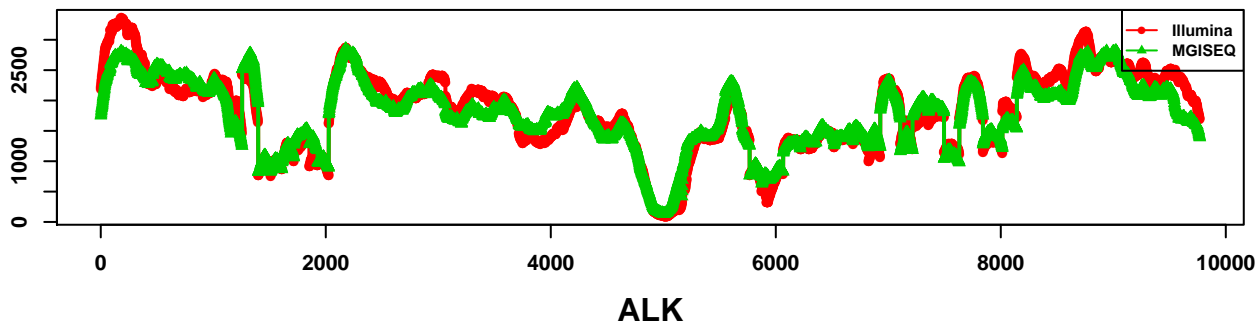

Sequencing Depth

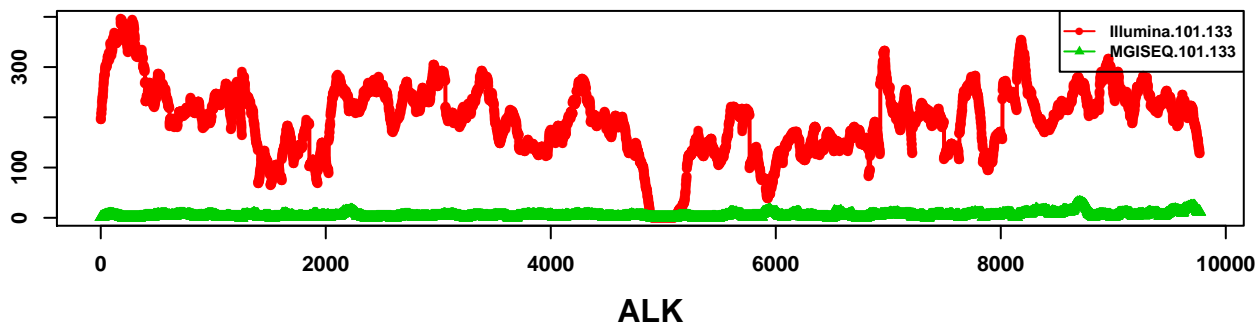

Sequencing Depth

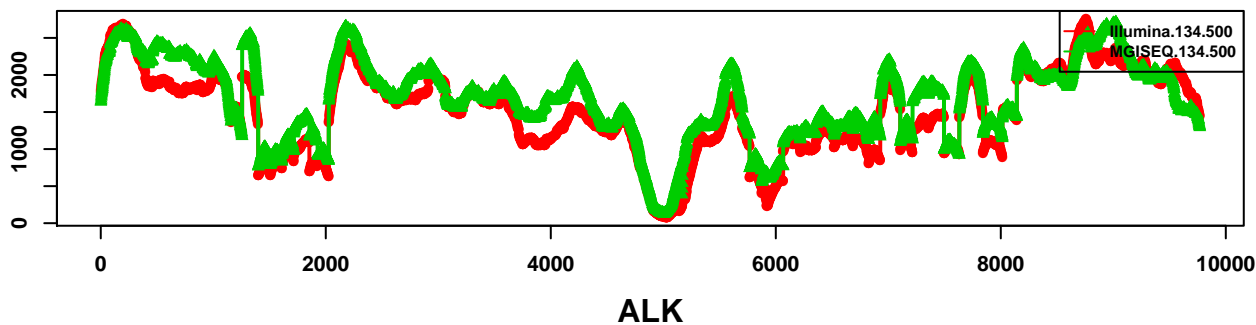

Supplement: Supplementary file 4 [file Presentation3.zip › ALK/19HE21950F.pdf]

Sequencing Depth

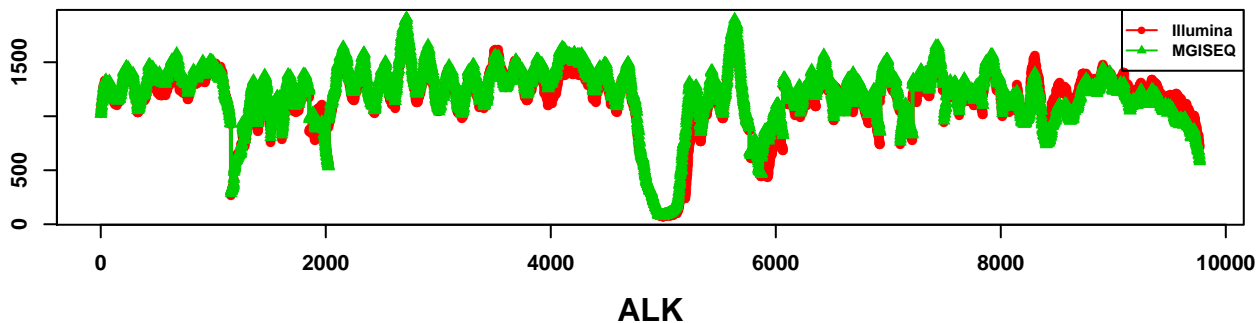

Sequencing Depth

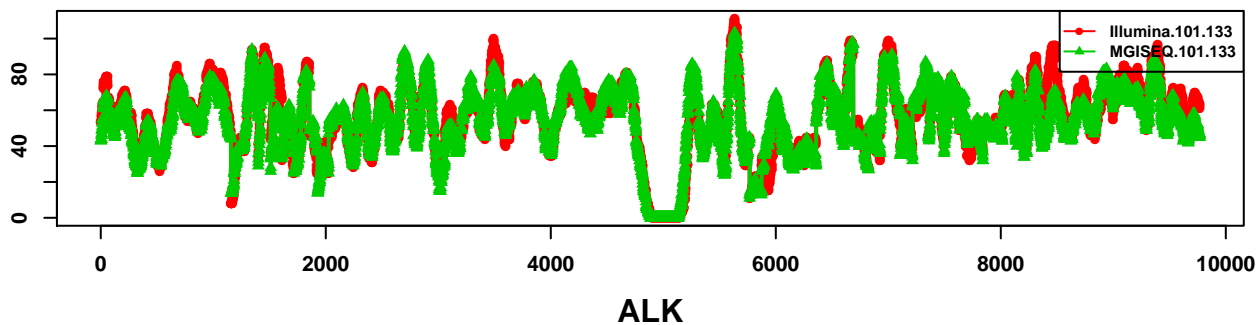

Sequencing Depth

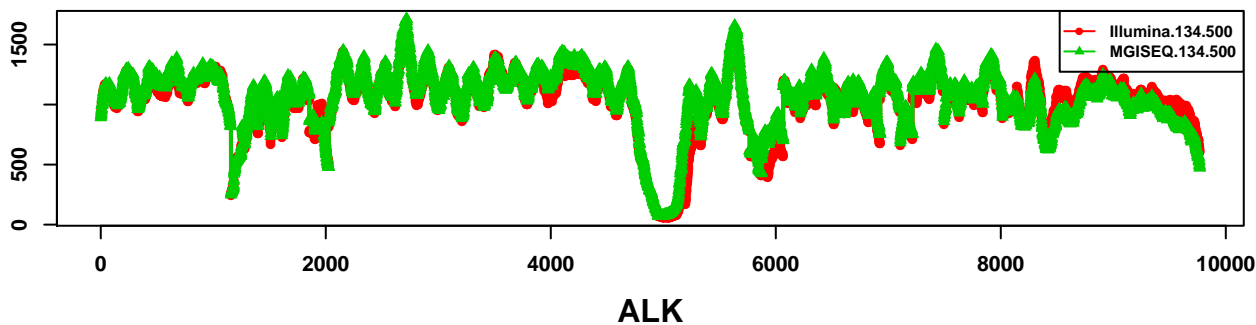

Supplement: Supplementary file 4 [file Presentation3.zip › ALK/19ZN13491P.pdf]

Sequencing Depth

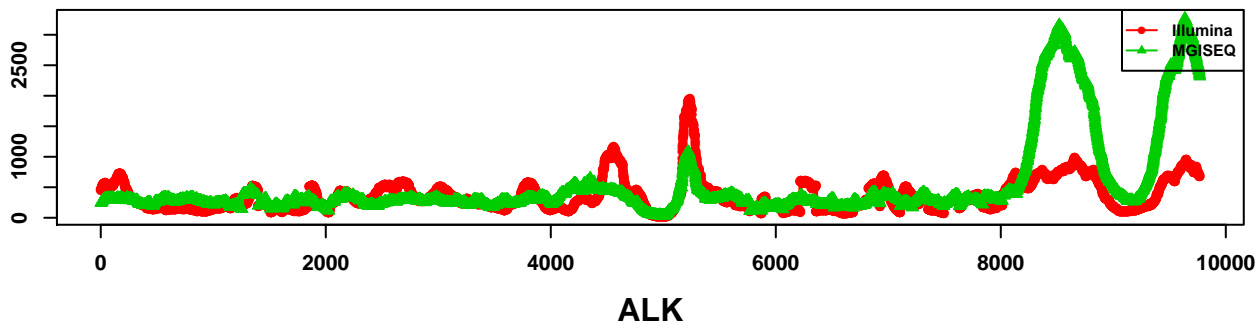

Sequencing Depth

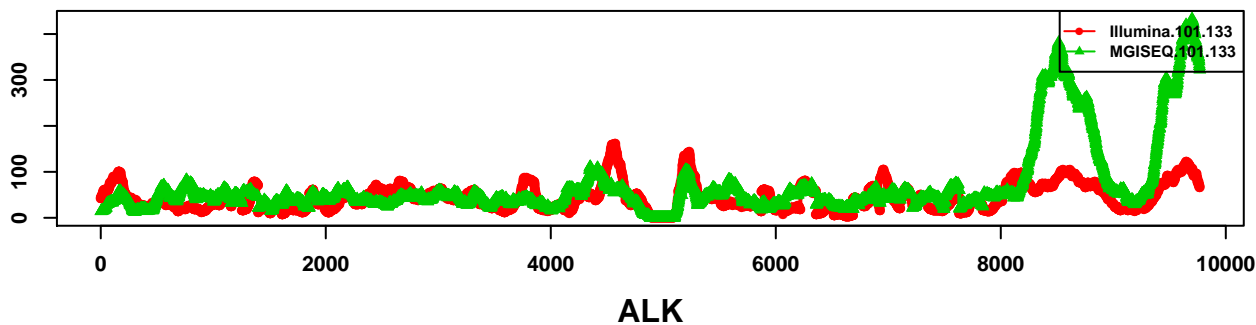

Sequencing Depth

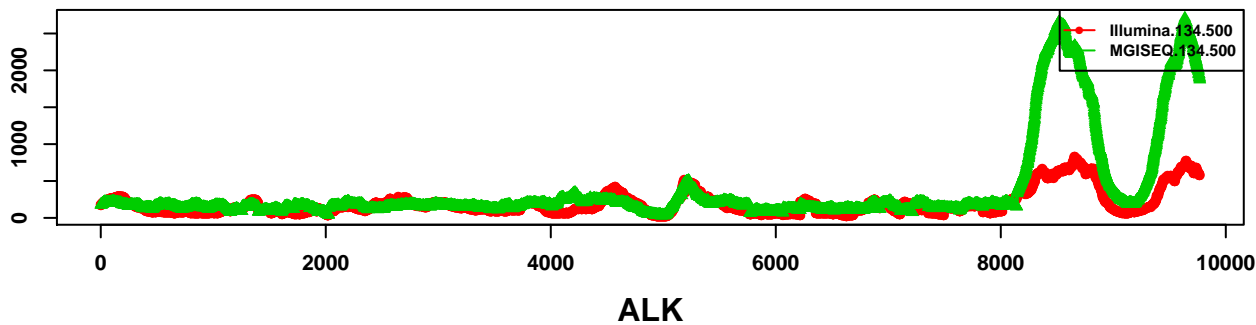

Supplement: Supplementary file 4 [file Presentation3.zip › ALK/19FC40427F.pdf]

Sequencing Depth

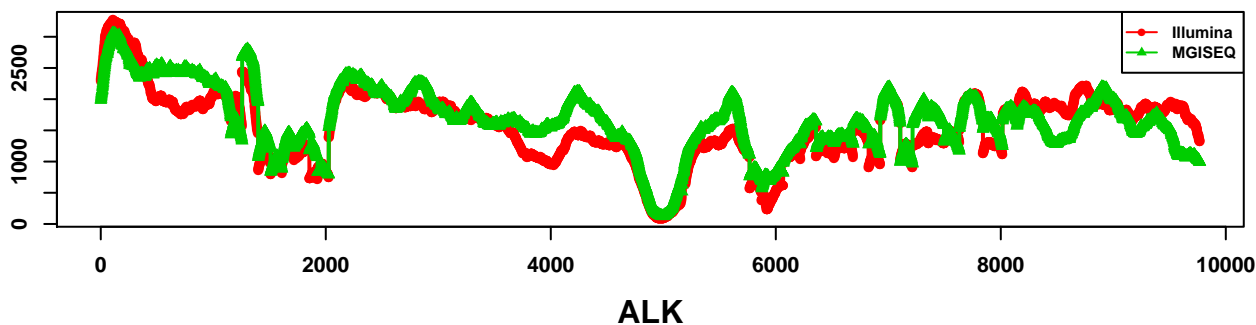

Sequencing Depth

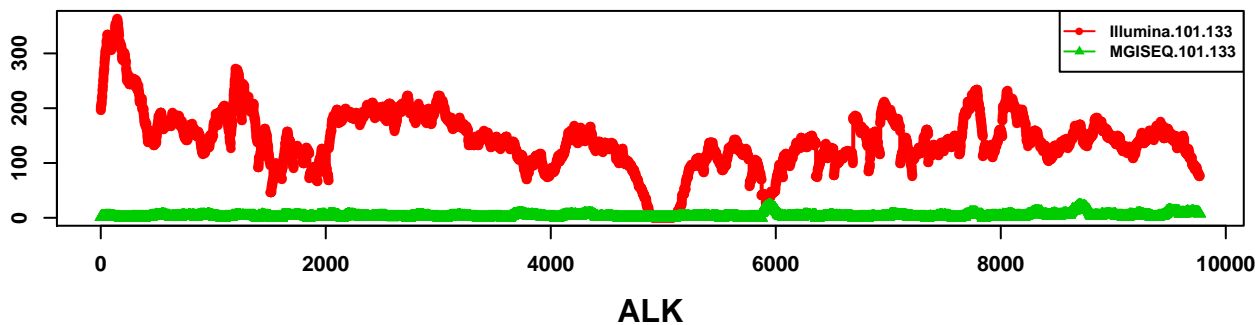

Sequencing Depth

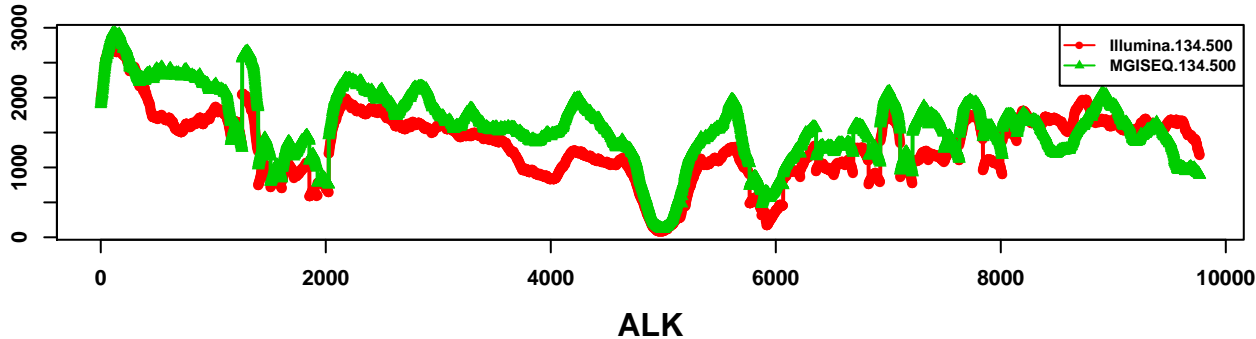

Supplement: Supplementary file 4 [file Presentation3.zip › ALK/19HE21842F.pdf]

Sequencing Depth

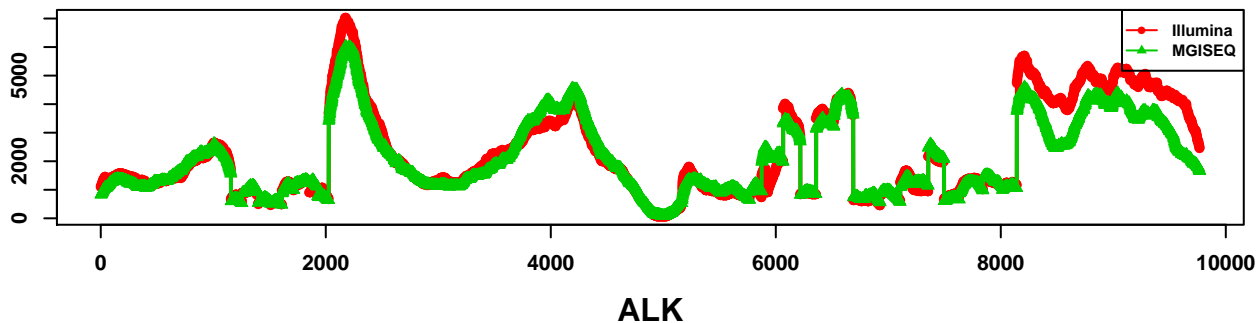

Sequencing Depth

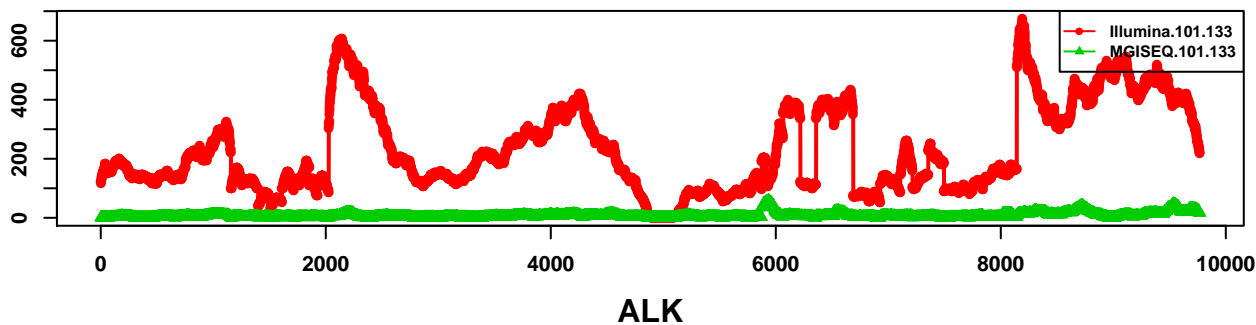

Sequencing Depth

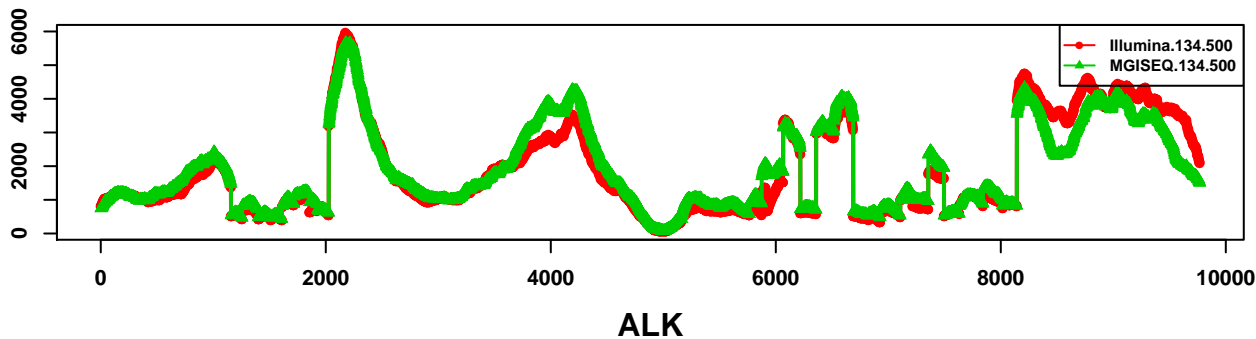

Supplement: Supplementary file 4 [file Presentation3.zip › ALK/19N01656F.pdf]

Sequencing Depth

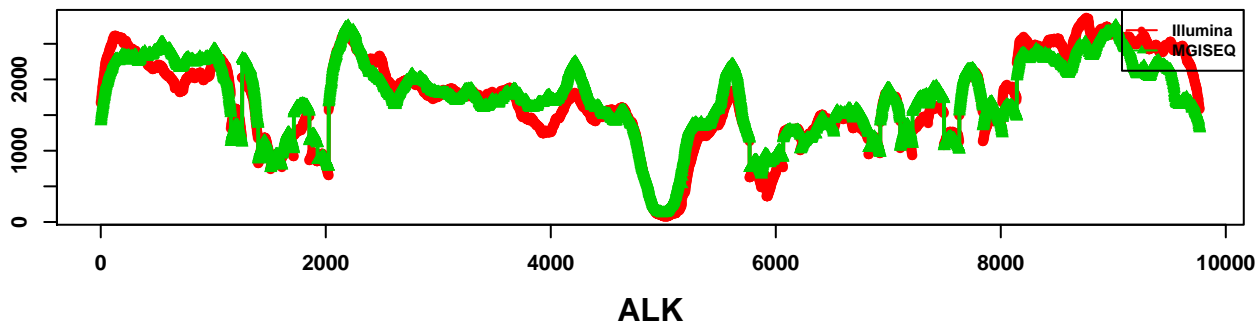

Sequencing Depth

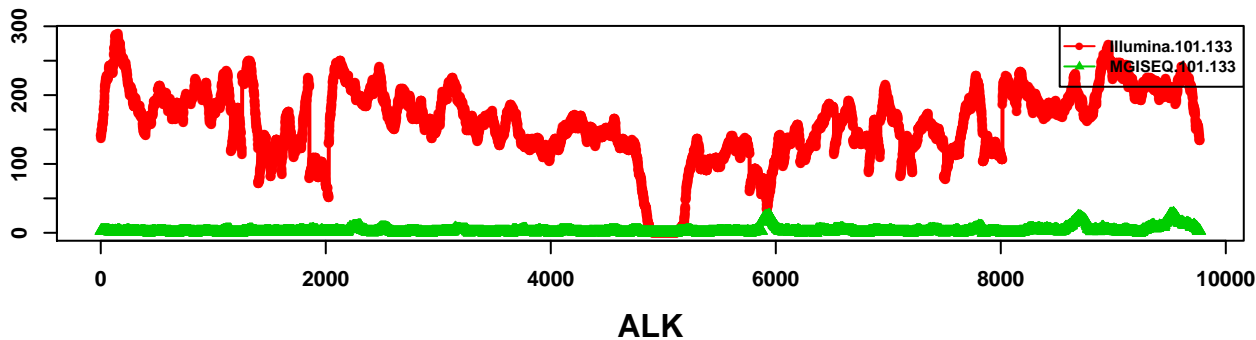

Sequencing Depth

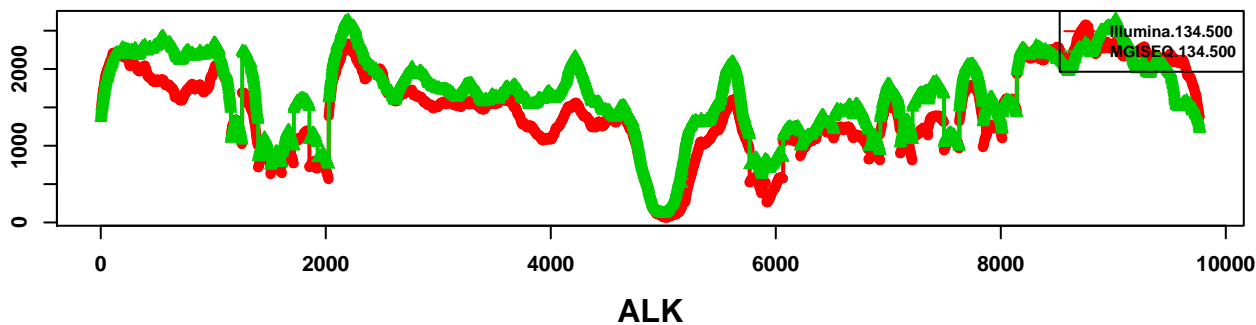

Supplement: Supplementary file 4 [file Presentation3.zip › ALK/19HE22110F.pdf]

Sequencing Depth

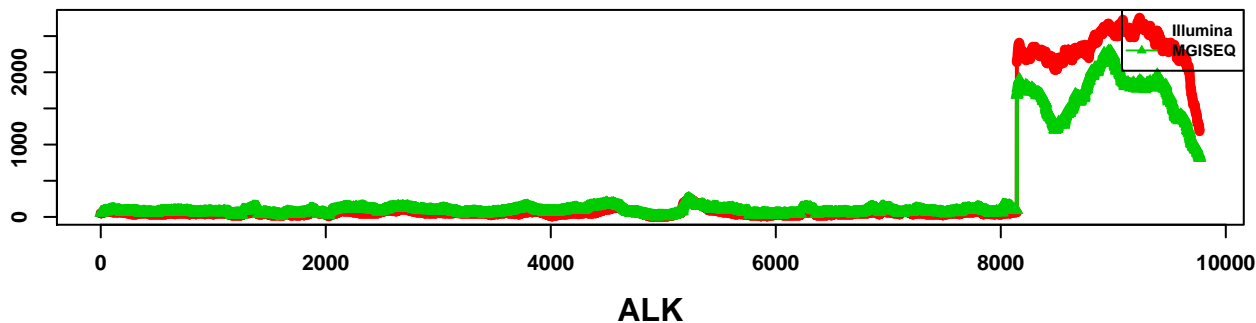

Sequencing Depth

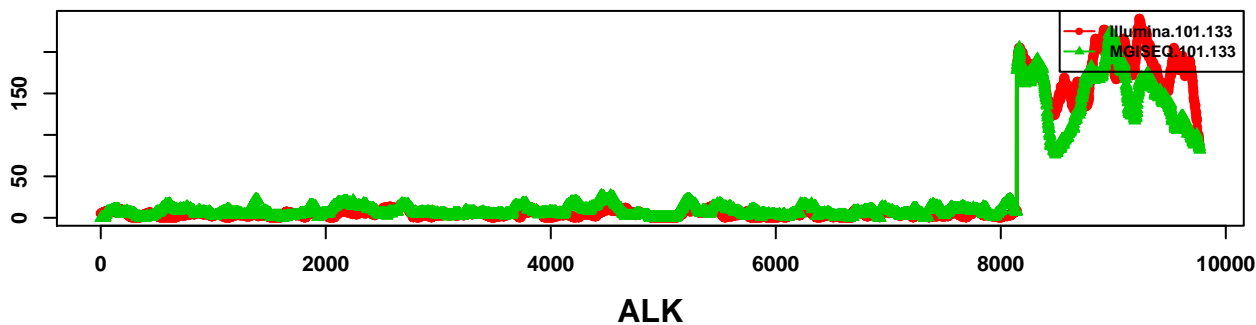

Sequencing Depth

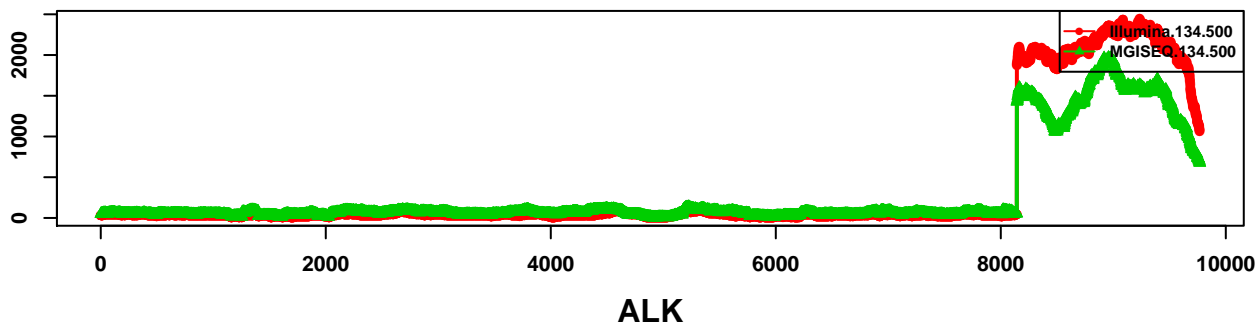

Supplement: Supplementary file 4 [file Presentation3.zip › ALK/19ZN13486T.pdf]

Sequencing Depth

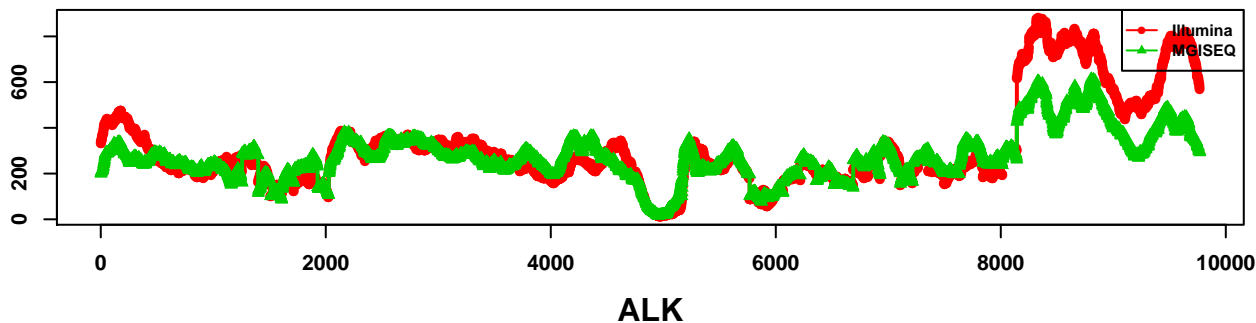

Sequencing Depth

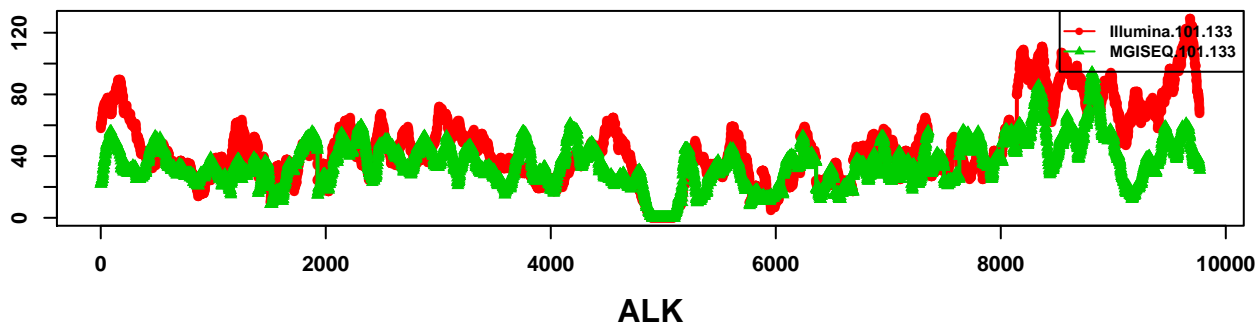

Sequencing Depth

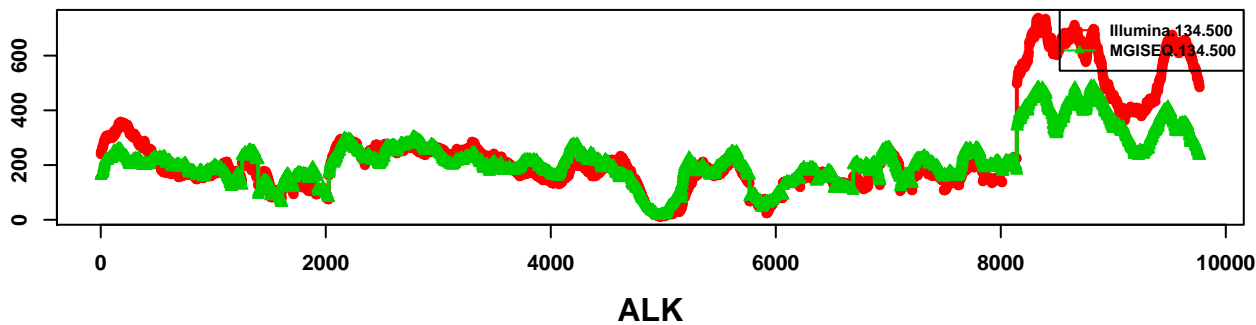

Supplement: Supplementary file 4 [file Presentation3.zip › ALK/19JS48247F.pdf]

Sequencing Depth

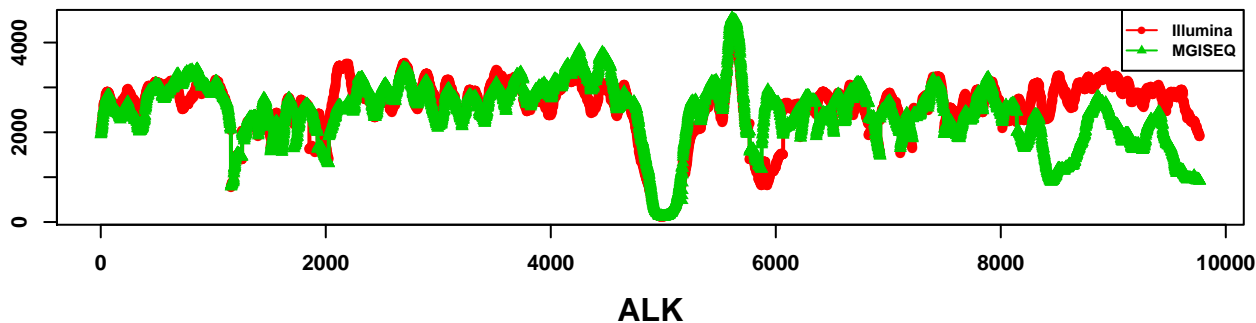

Sequencing Depth

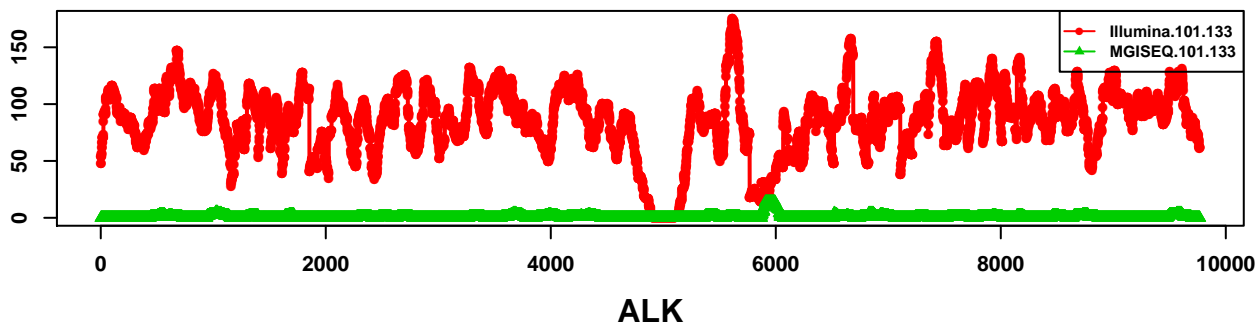

Sequencing Depth

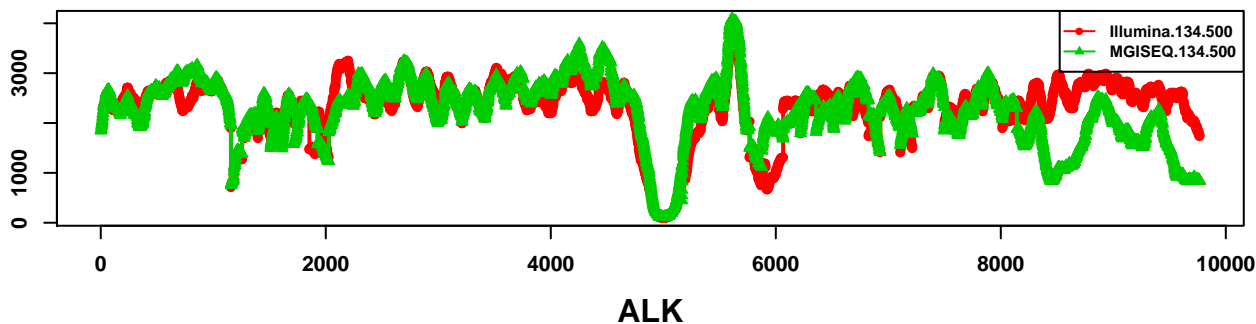

Supplement: Supplementary file 4 [file Presentation3.zip › ALK/19ZN12296P.pdf]

Sequencing Depth

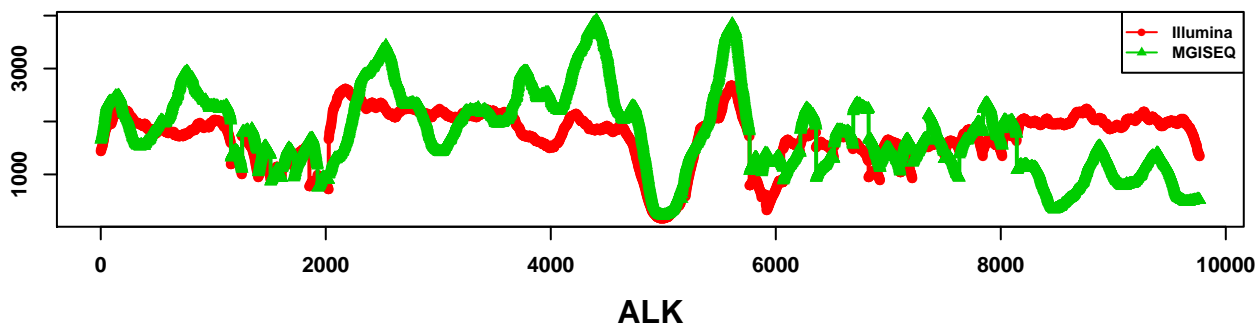

Sequencing Depth

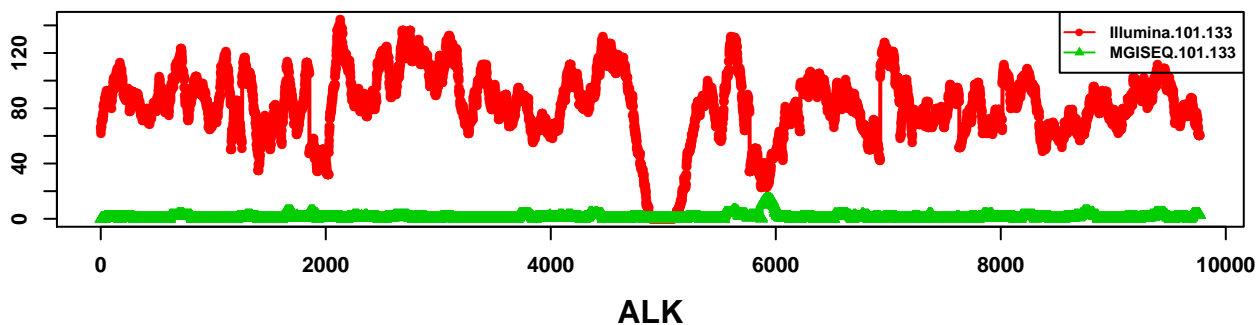

Sequencing Depth

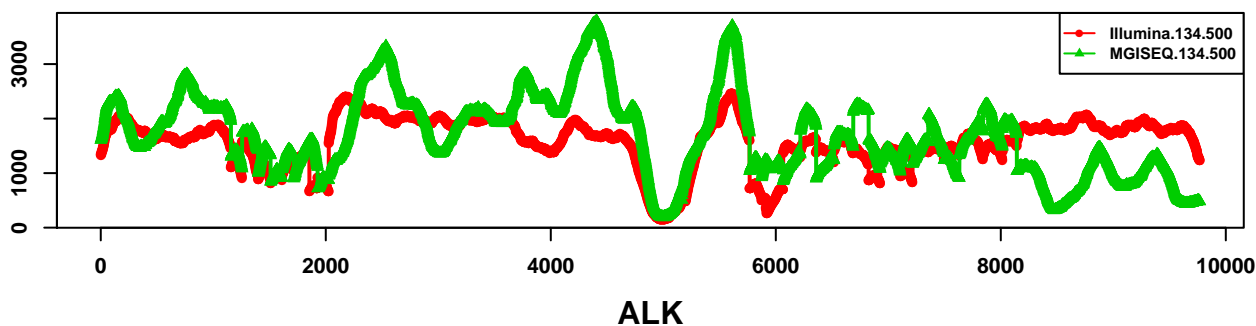

Supplement: Supplementary file 4 [file Presentation3.zip › ALK/19CF15537B.pdf]

Sequencing Depth

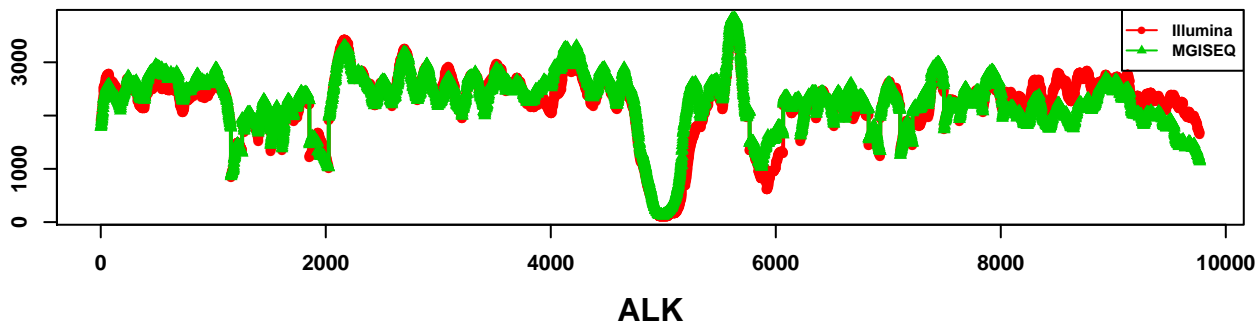

Sequencing Depth

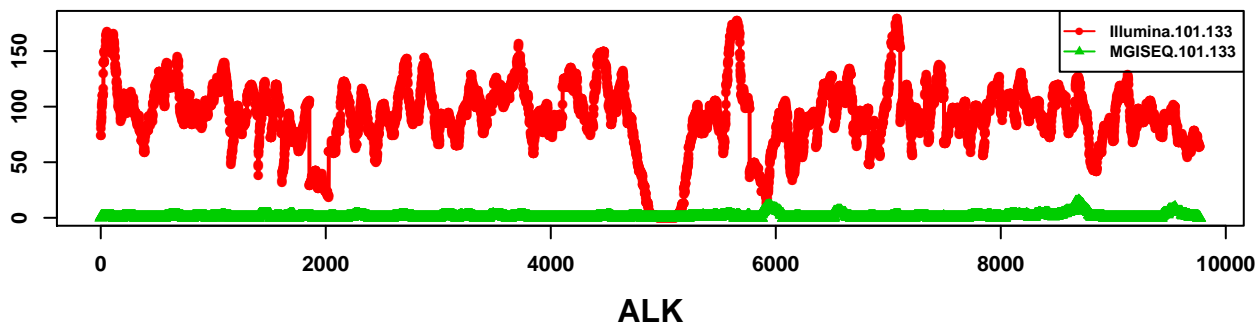

Sequencing Depth

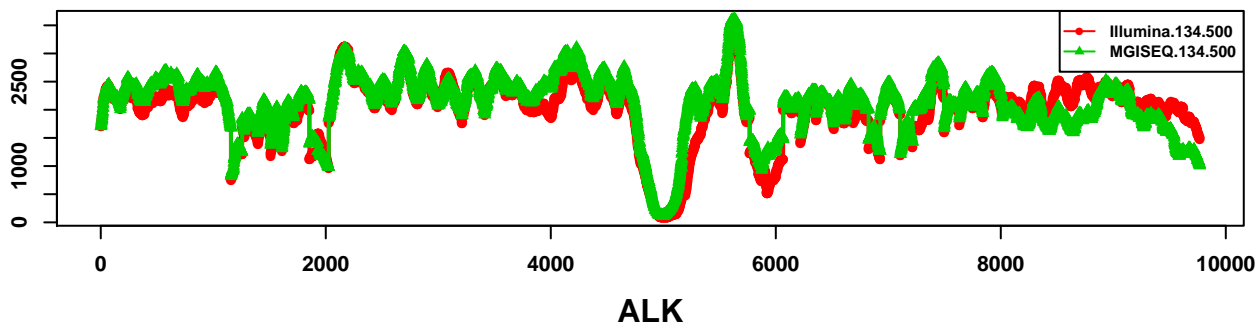

Supplement: Supplementary file 4 [file Presentation3.zip › ALK/19ZN13106P.pdf]

Sequencing Depth

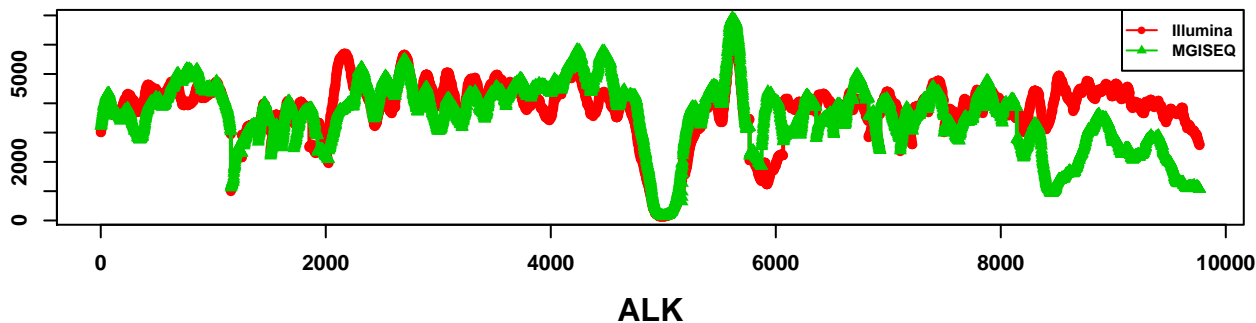

Sequencing Depth

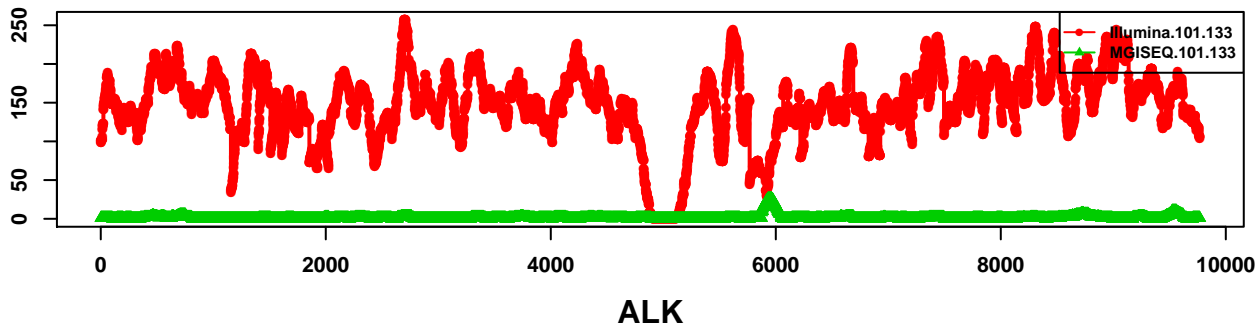

Sequencing Depth

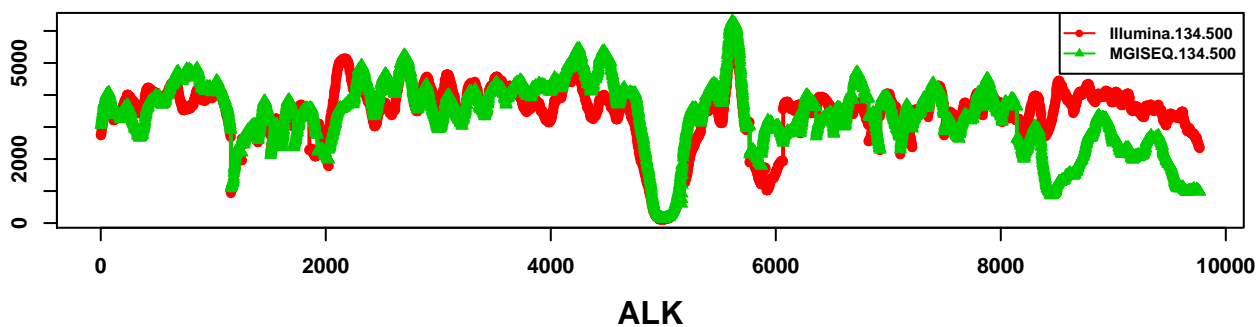

Supplement: Supplementary file 4 [file Presentation3.zip › ALK/19JS48181P.pdf]

Sequencing Depth

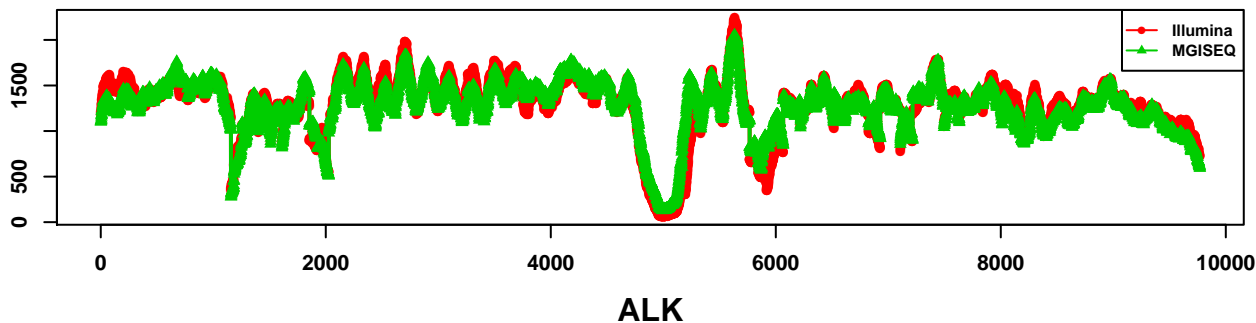

Sequencing Depth

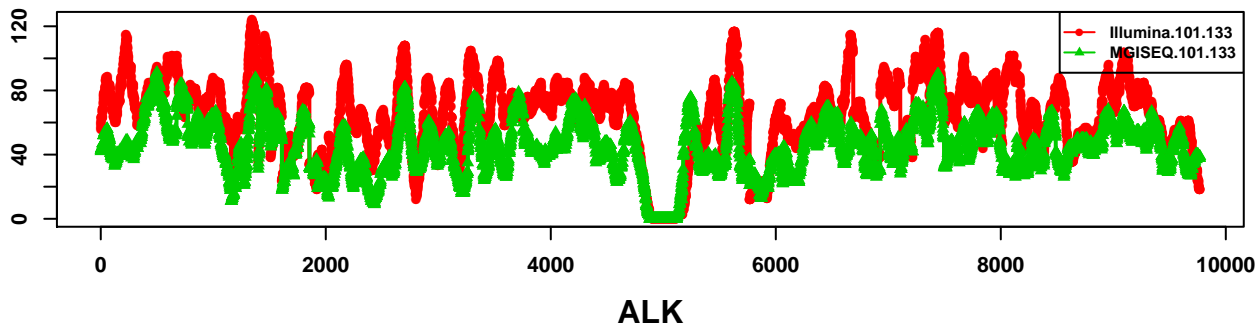

Sequencing Depth

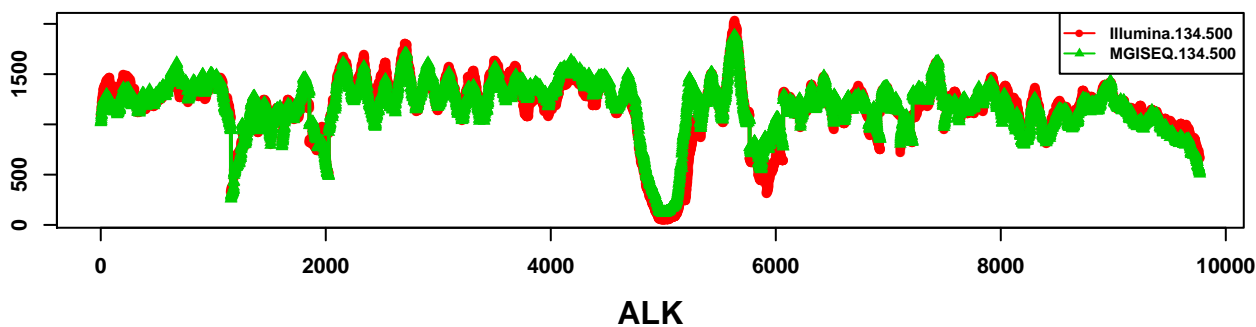

Supplement: Supplementary file 4 [file Presentation3.zip › ALK/19N02353P.pdf]

Sequencing Depth

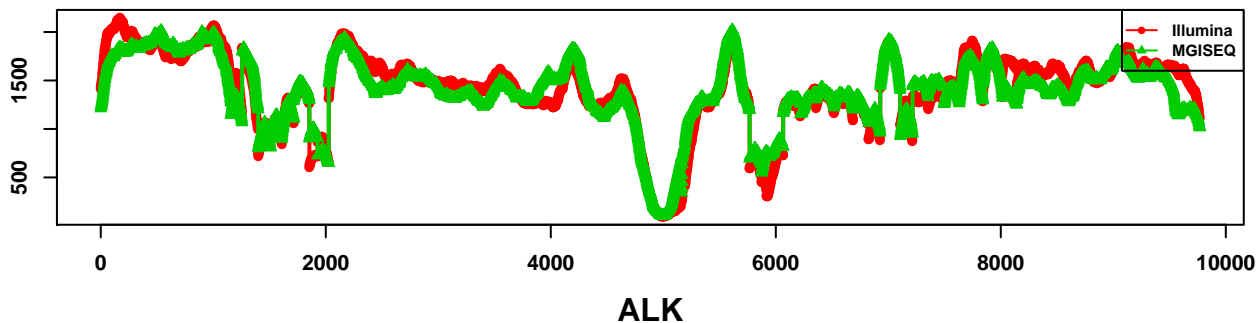

Sequencing Depth

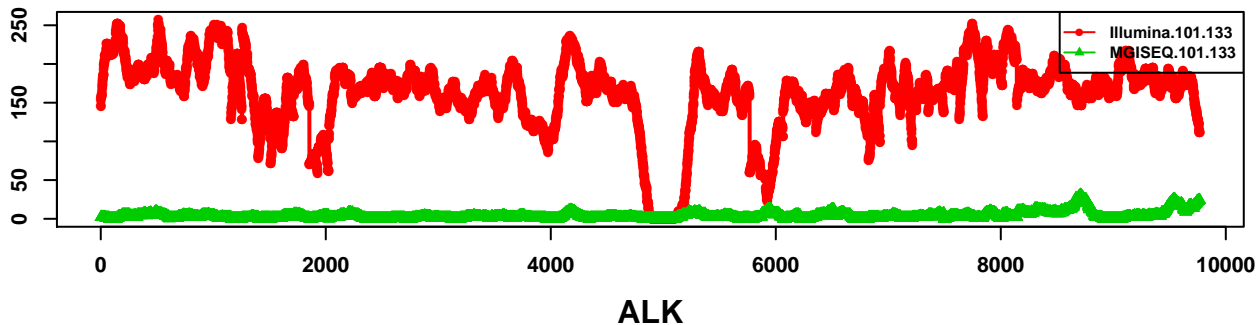

Sequencing Depth

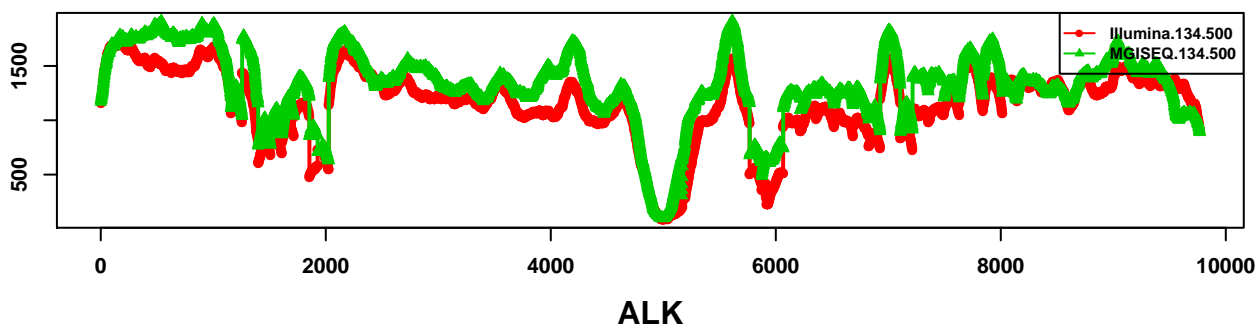

Supplement: Supplementary file 4 [file Presentation3.zip › ALK/19FC40260F.pdf]

Sequencing Depth

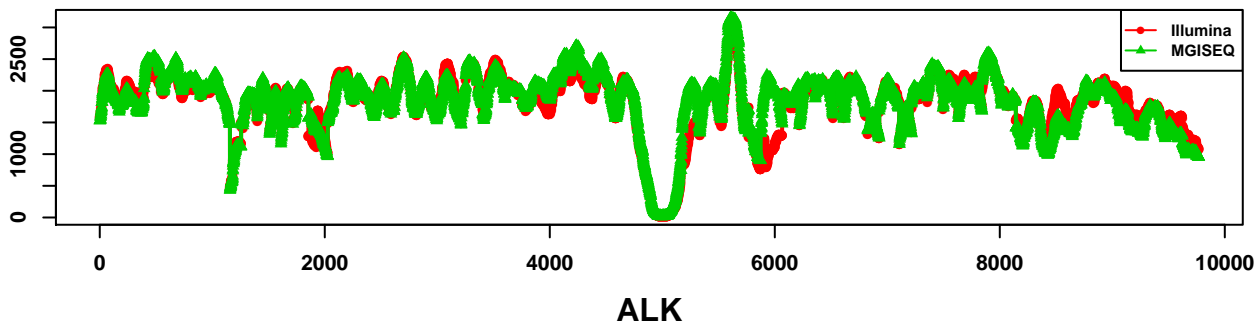

Sequencing Depth

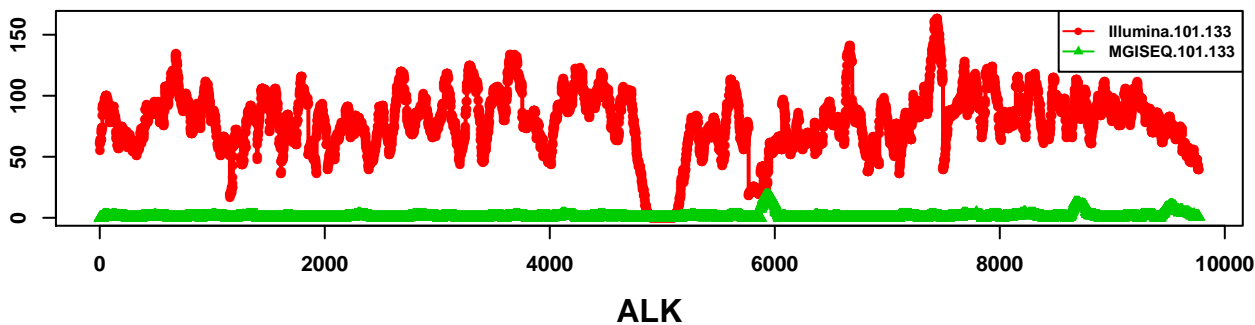

Sequencing Depth

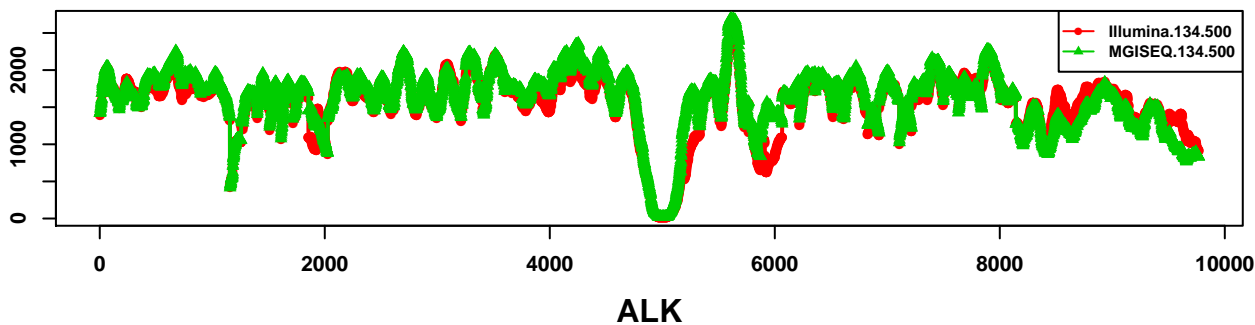

Supplement: Supplementary file 4 [file Presentation3.zip › ALK/19N01456-IIIP.pdf]

Sequencing Depth

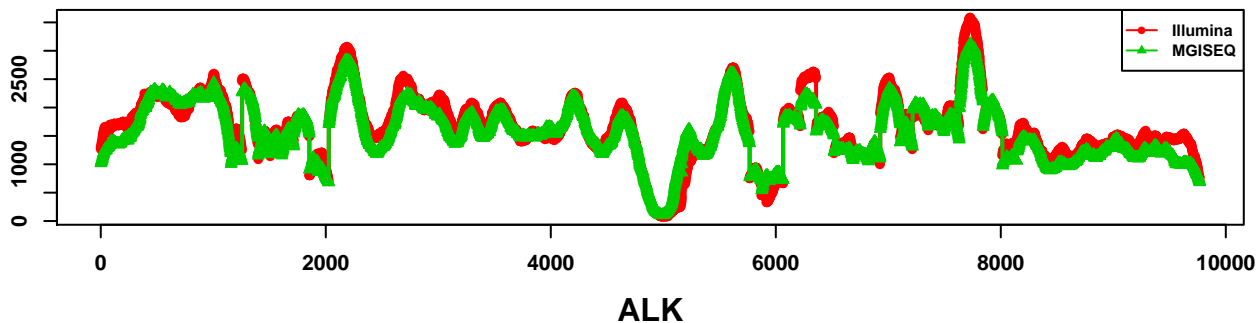

Sequencing Depth

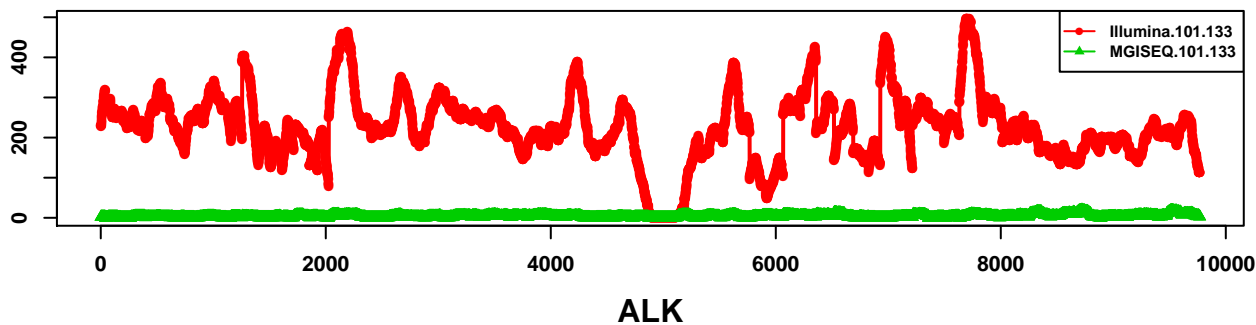

Sequencing Depth

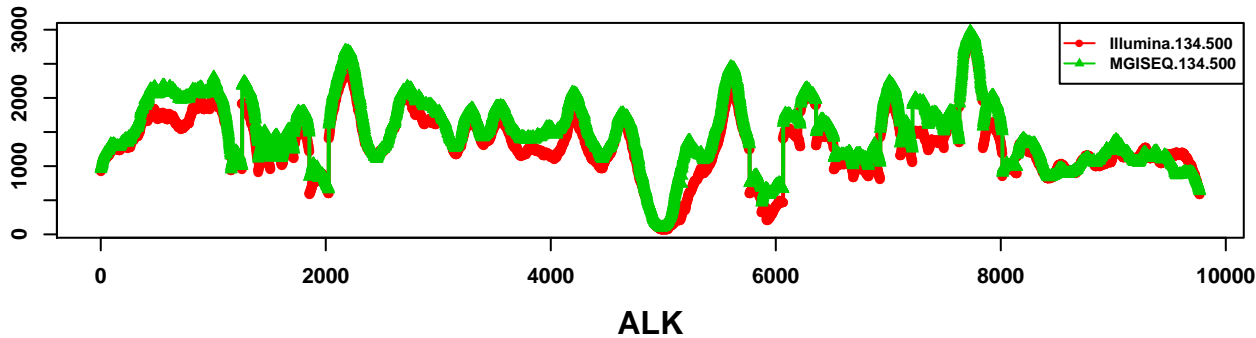

Supplement: Supplementary file 4 [file Presentation3.zip › ALK/19FC40261F.pdf]

Sequencing Depth

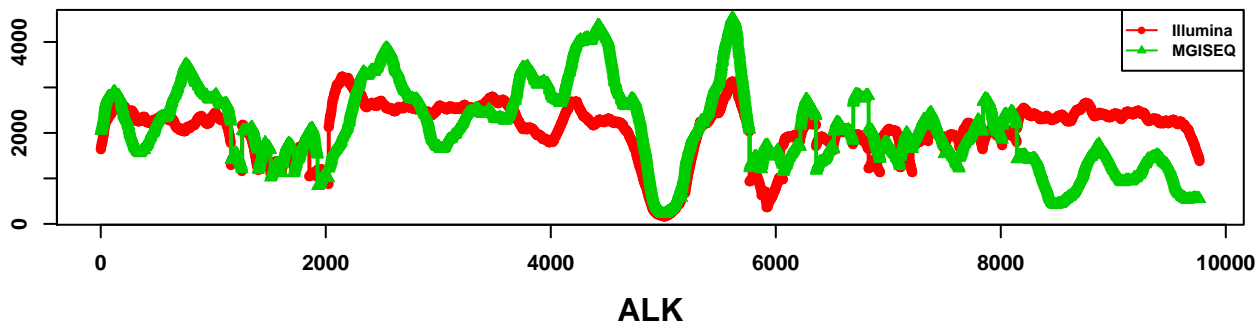

Sequencing Depth

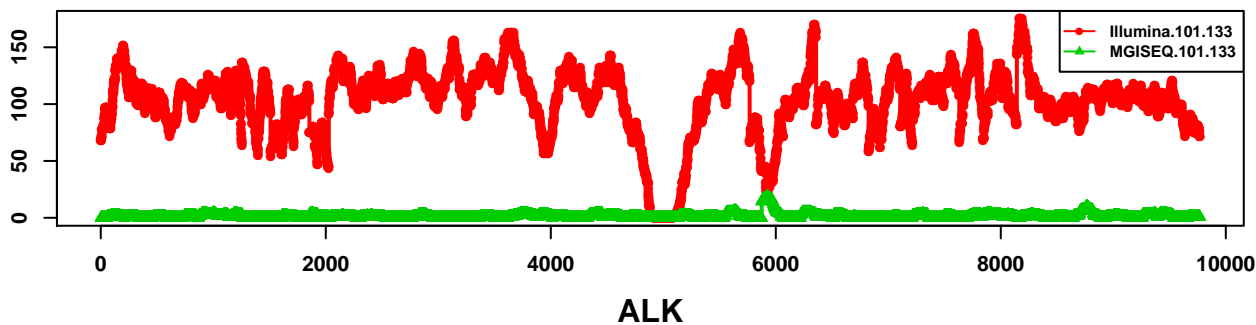

Sequencing Depth

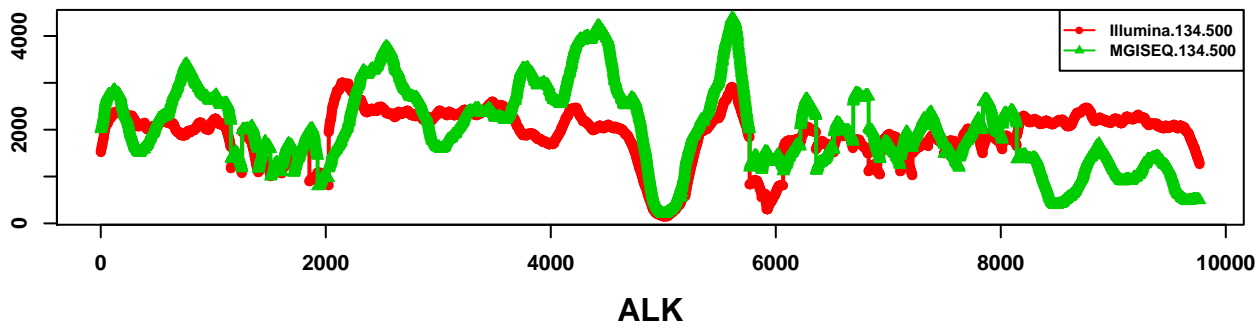

Supplement: Supplementary file 4 [file Presentation3.zip › ALK/19ZN13096B.pdf]

Sequencing Depth

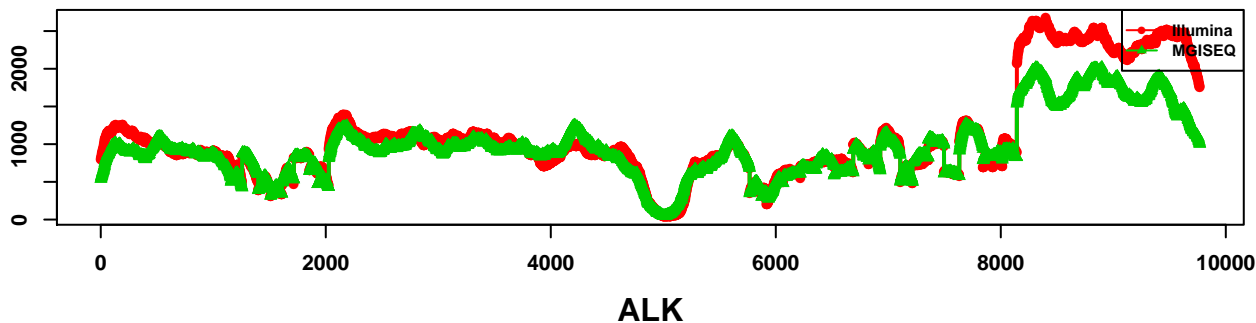

Sequencing Depth

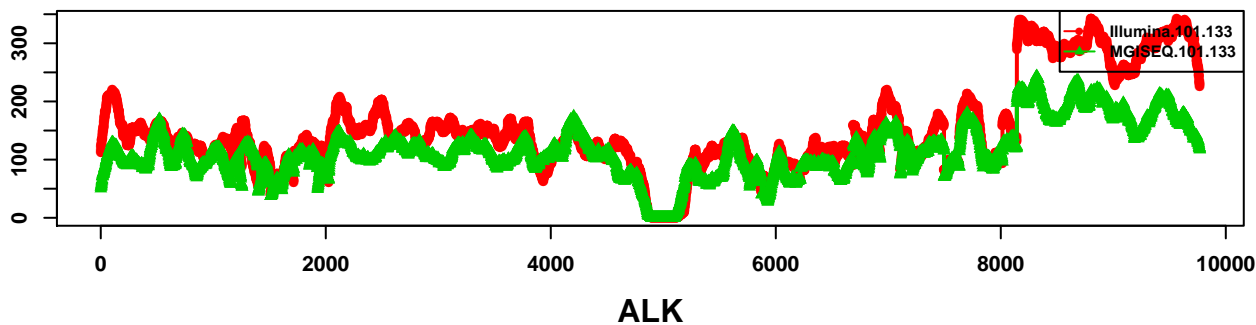

Sequencing Depth

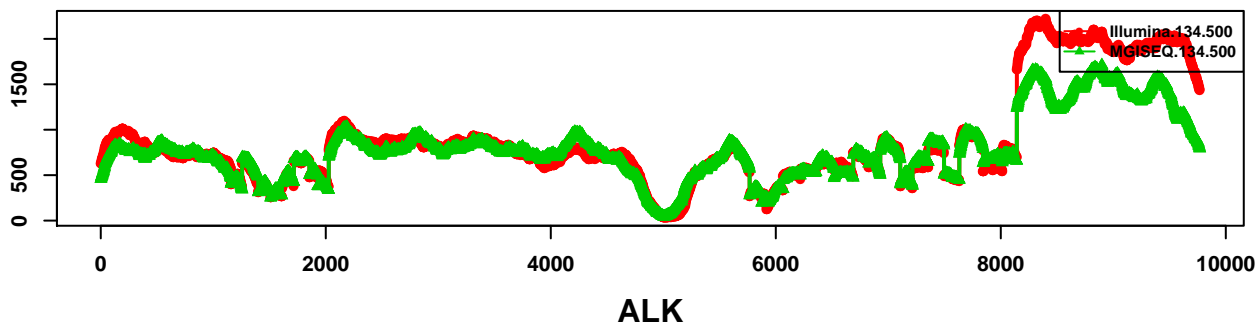

Supplement: Supplementary file 4 [file Presentation3.zip › ALK/19N02329F.pdf]

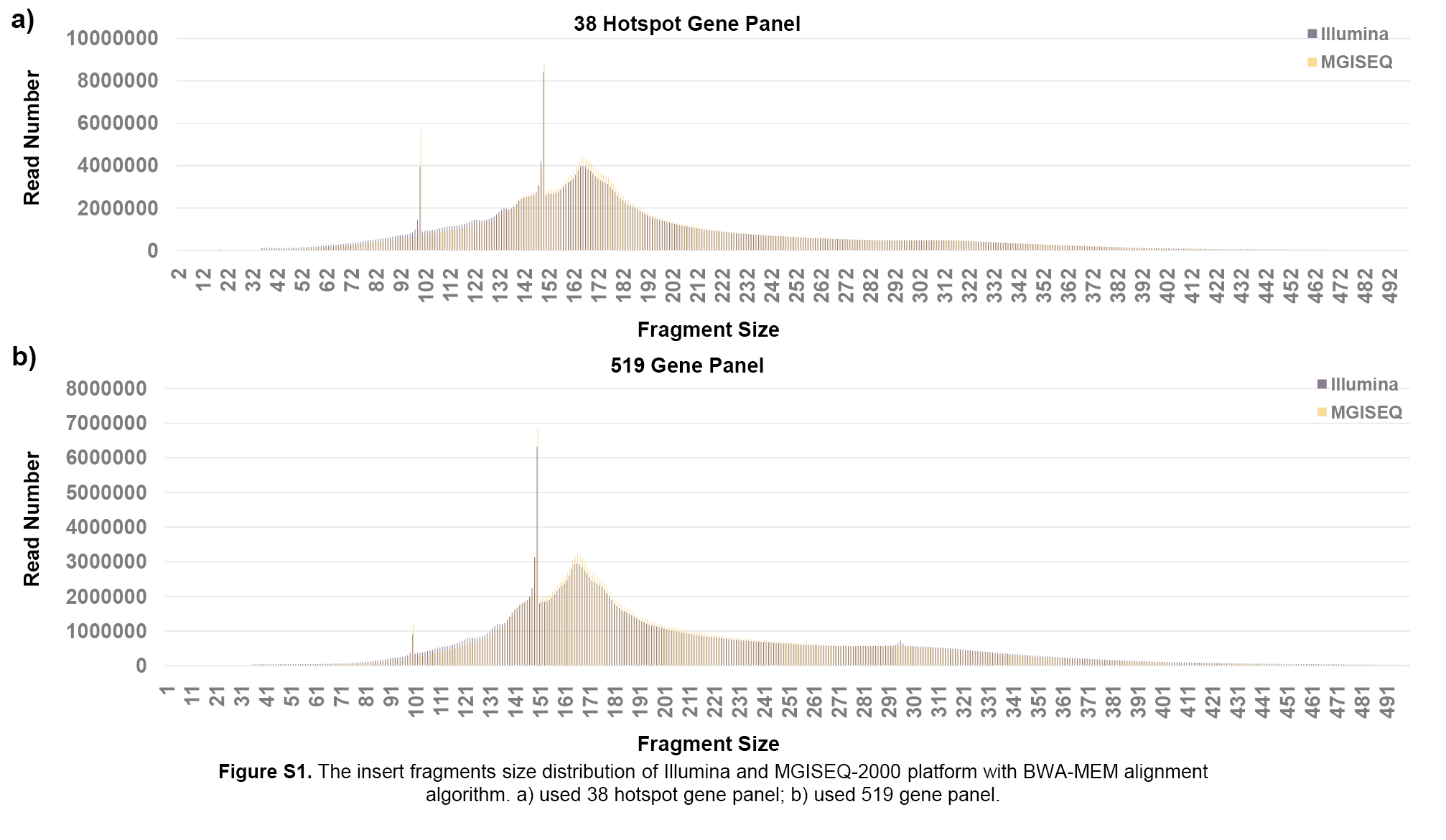

Supplement: Supplementary file 5 [file Image1.TIF]

Sequencing Depth

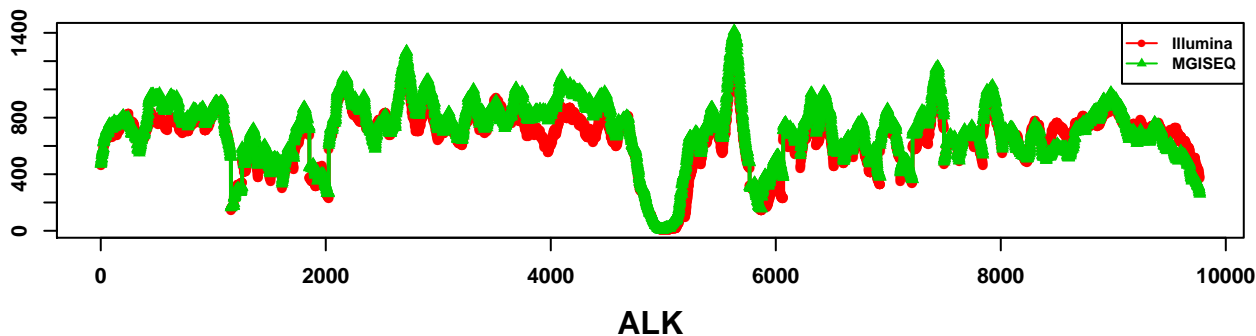

Sequencing Depth

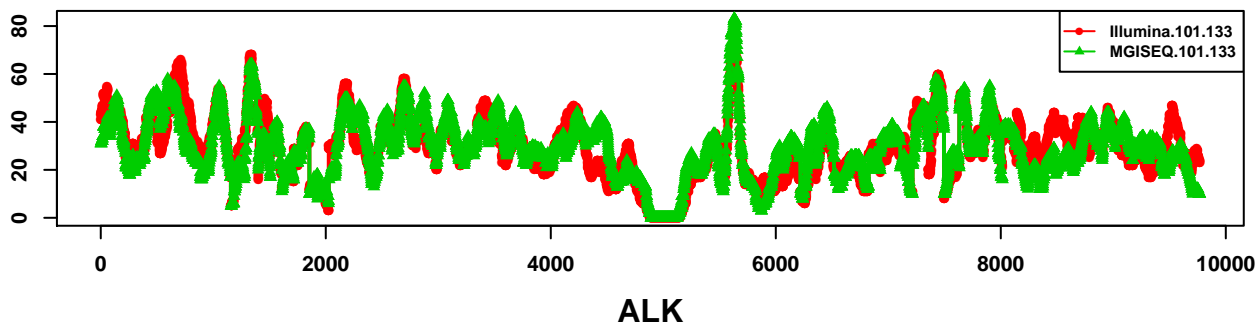

Sequencing Depth

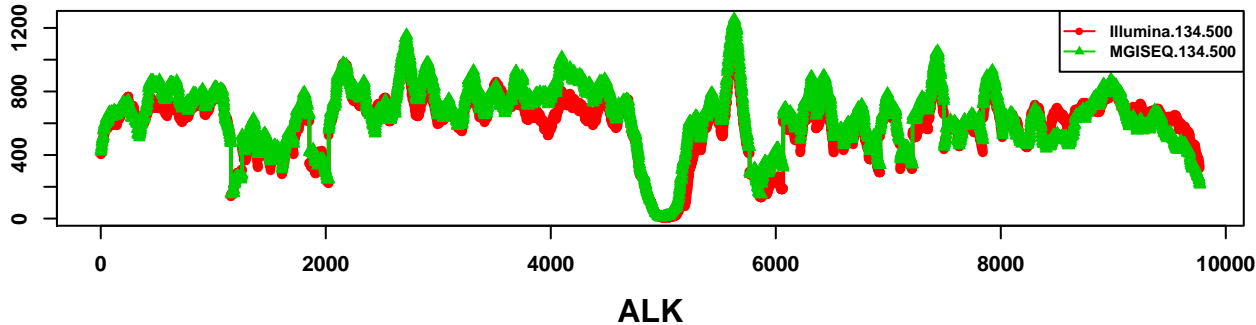

Supplement: Supplementary file 6 [file Presentation5.zip › ALK/19ZN12531P.pdf]

Sequencing Depth

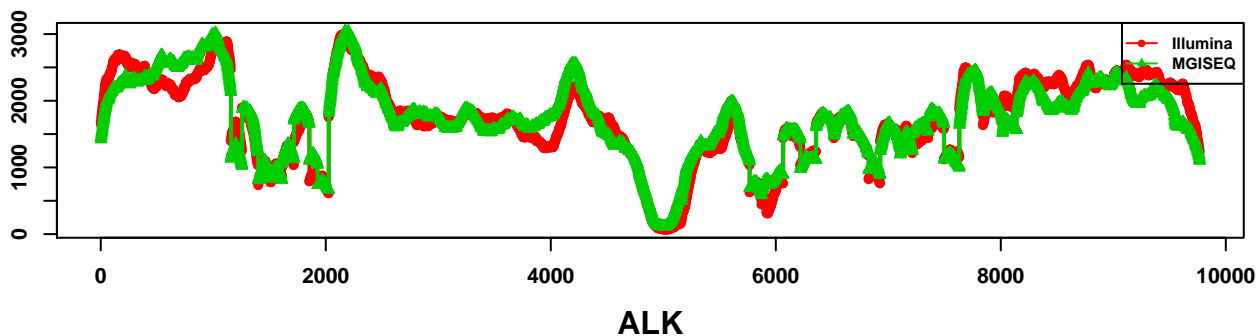

Sequencing Depth

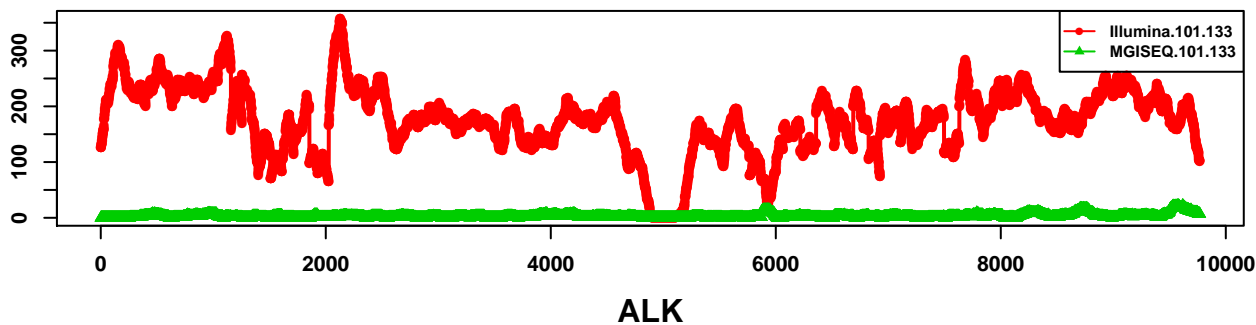

Sequencing Depth

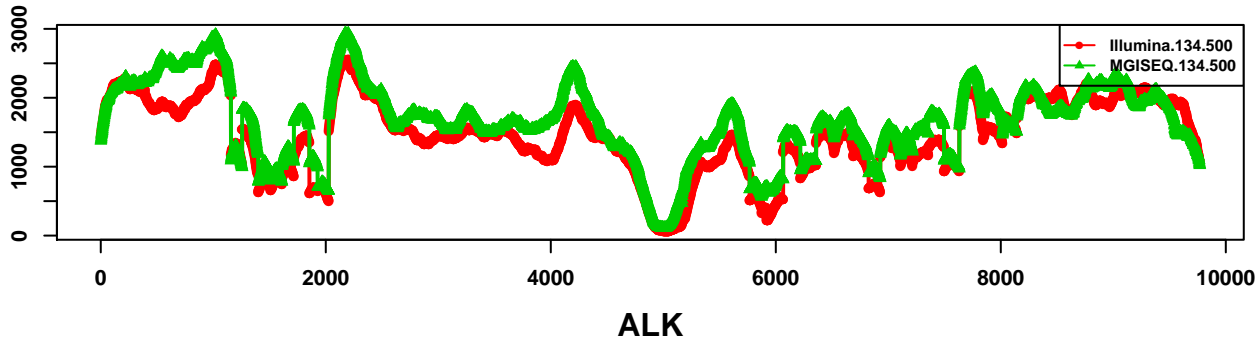

Supplement: Supplementary file 6 [file Presentation5.zip › ALK/19HE21977F.pdf]

Sequencing Depth

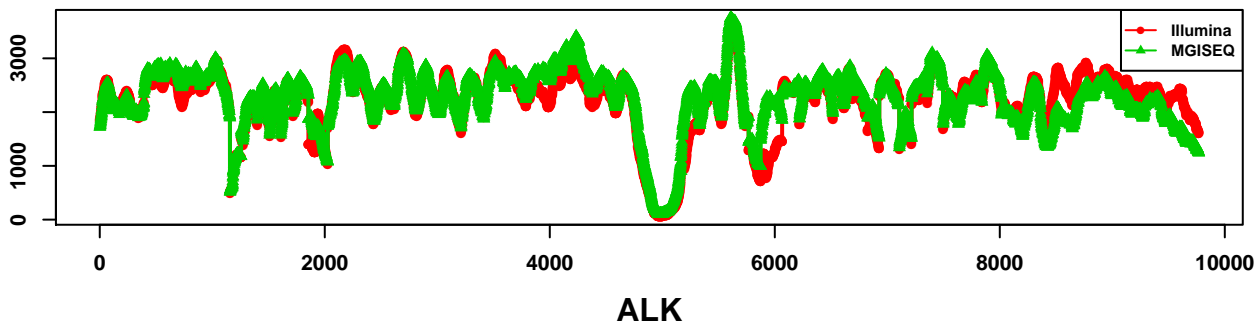

Sequencing Depth

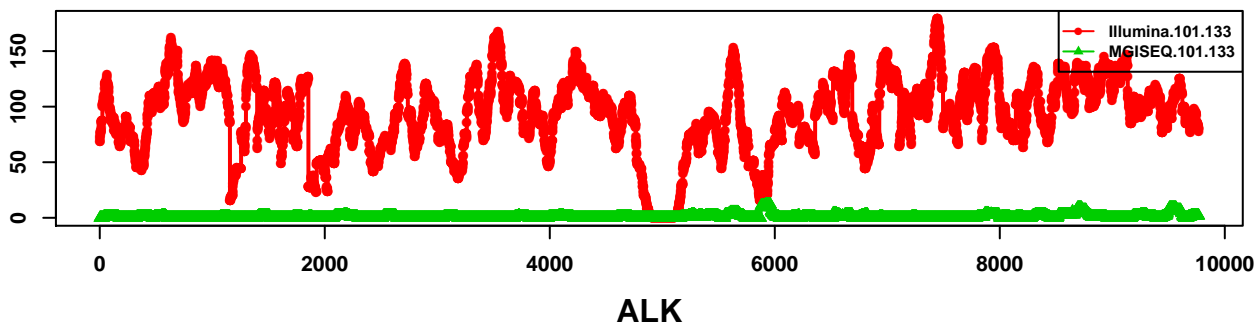

Sequencing Depth

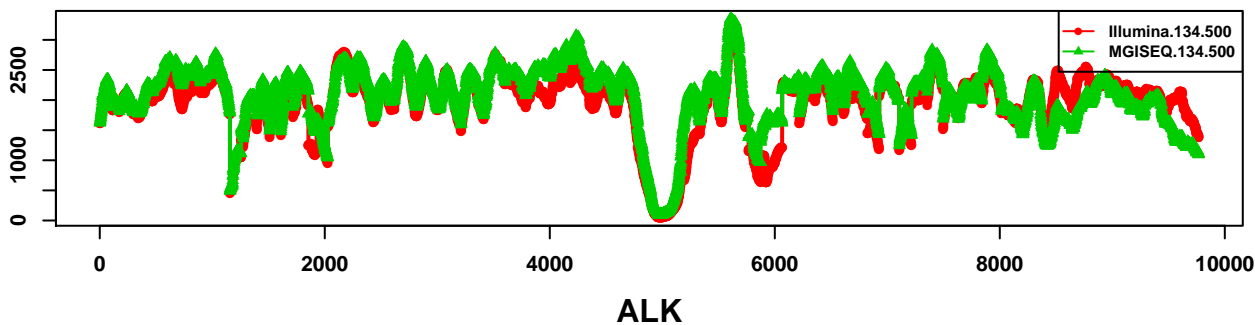

Supplement: Supplementary file 6 [file Presentation5.zip › ALK/19ZN10625P.pdf]

Sequencing Depth

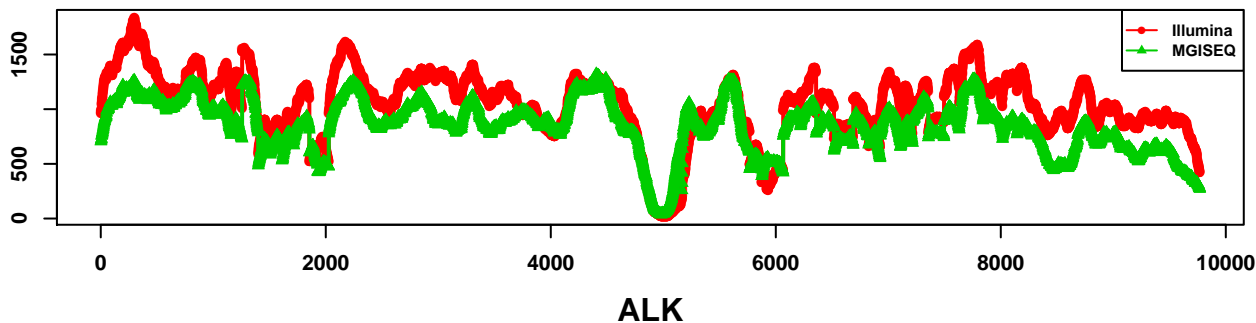

Sequencing Depth

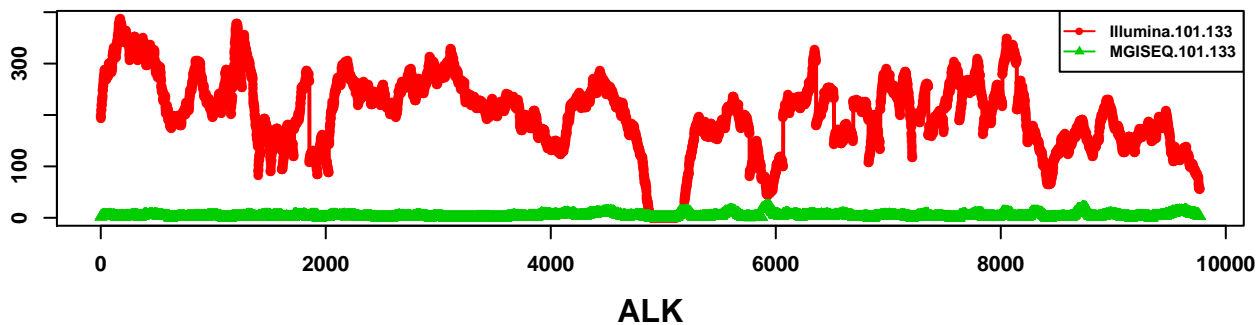

Sequencing Depth

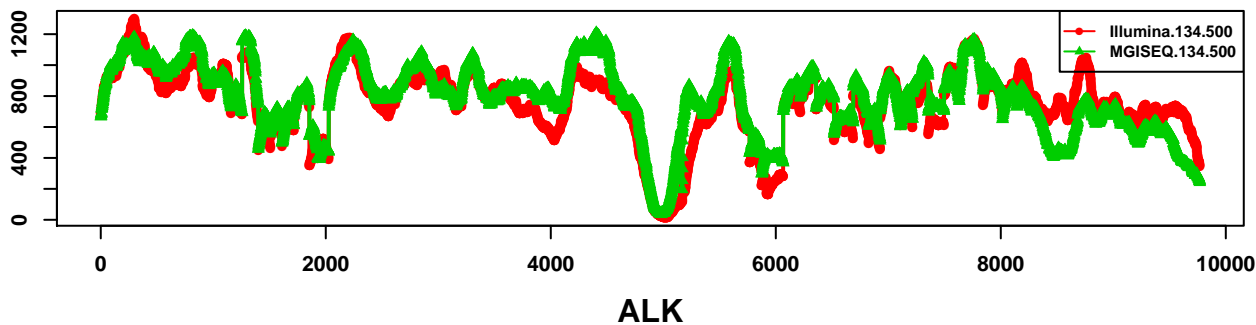

Supplement: Supplementary file 6 [file Presentation5.zip › ALK/19N01626F.pdf]

Sequencing Depth

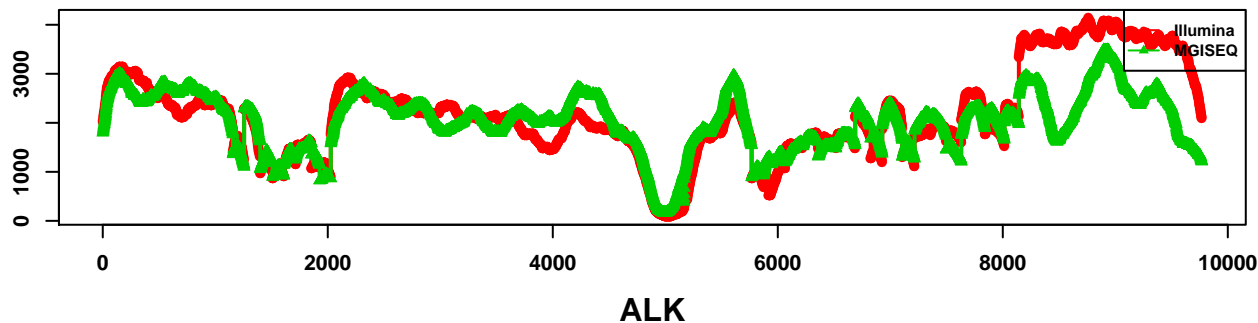

Sequencing Depth

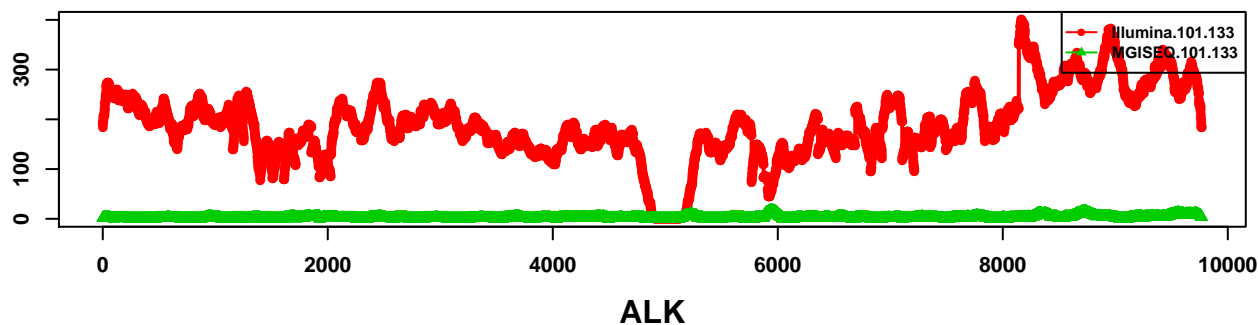

Sequencing Depth

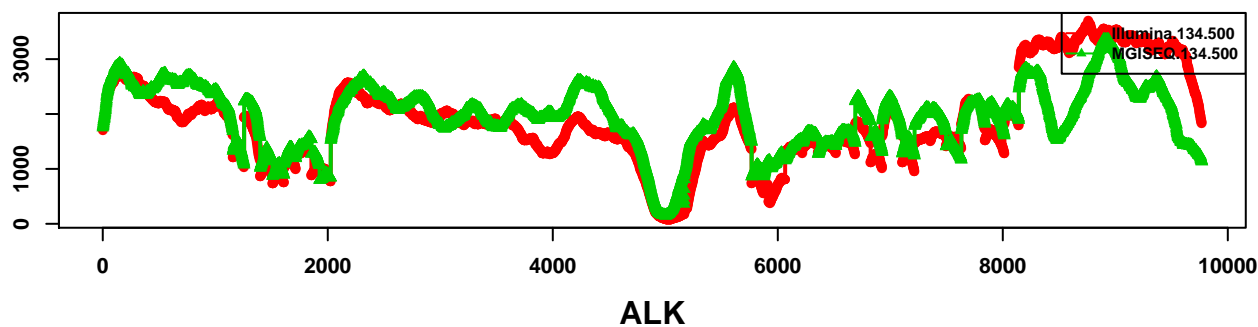

Supplement: Supplementary file 6 [file Presentation5.zip › ALK/19N01567QC.pdf]

Sequencing Depth

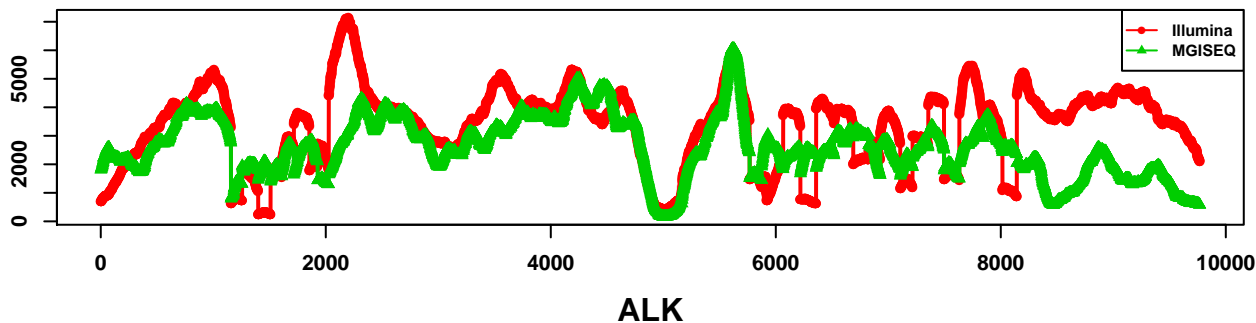

Sequencing Depth

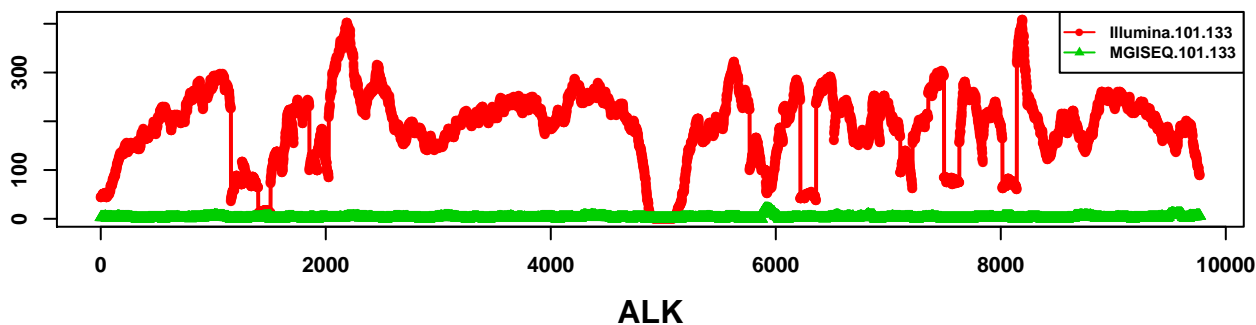

Sequencing Depth

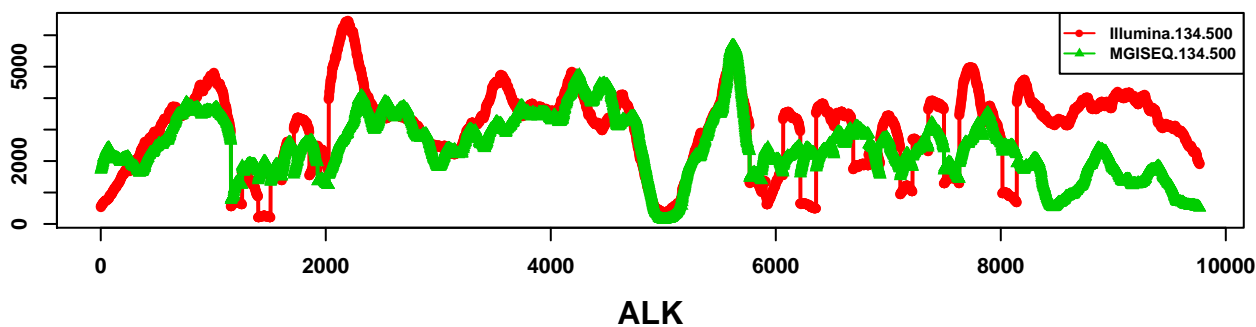

Supplement: Supplementary file 6 [file Presentation5.zip › ALK/19N01670T.pdf]

Sequencing Depth

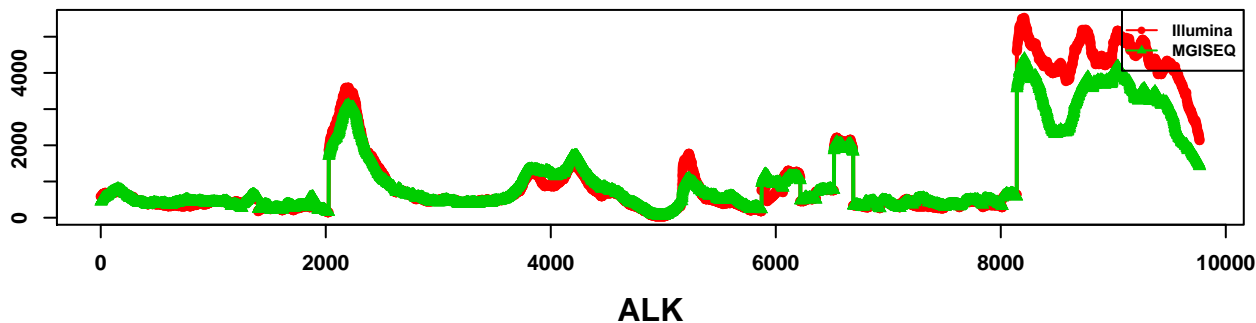

Sequencing Depth

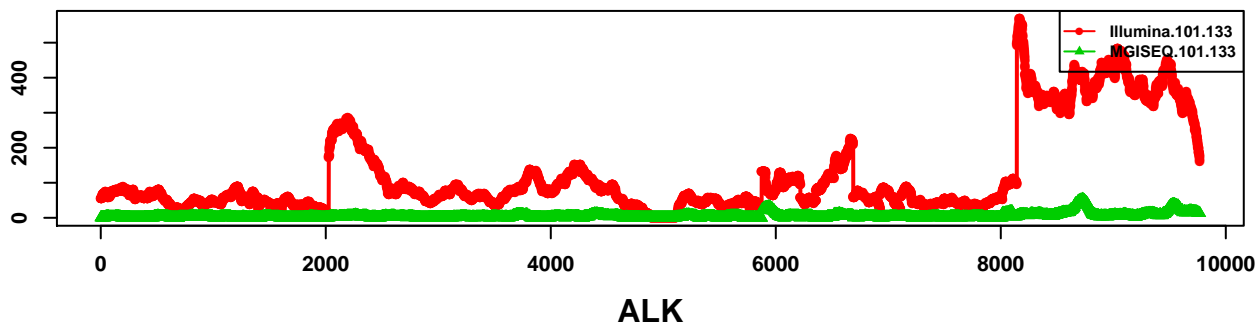

Sequencing Depth

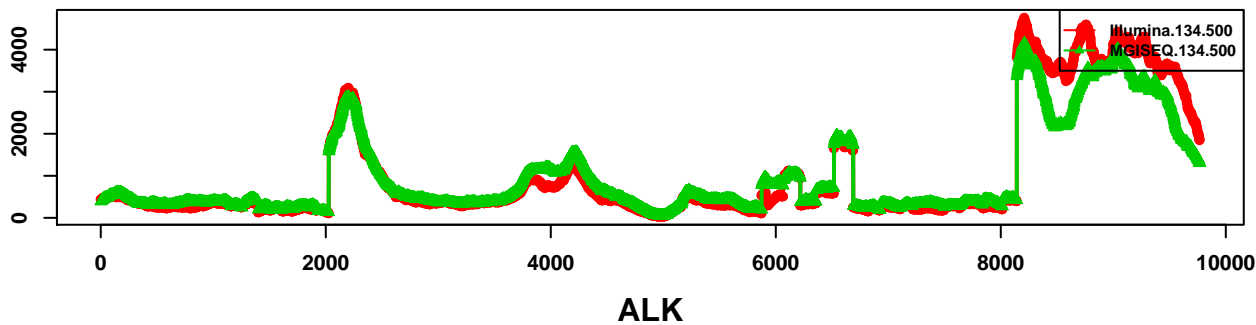

Supplement: Supplementary file 6 [file Presentation5.zip › ALK/19FC40251F.pdf]

Sequencing Depth

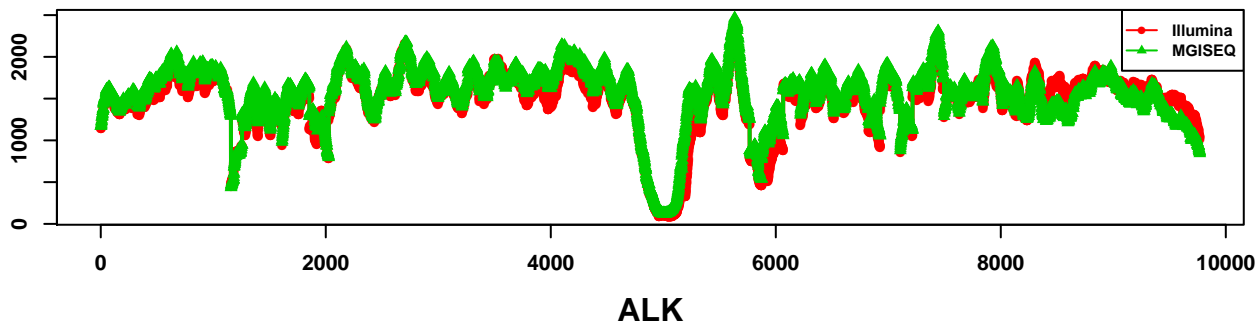

Sequencing Depth

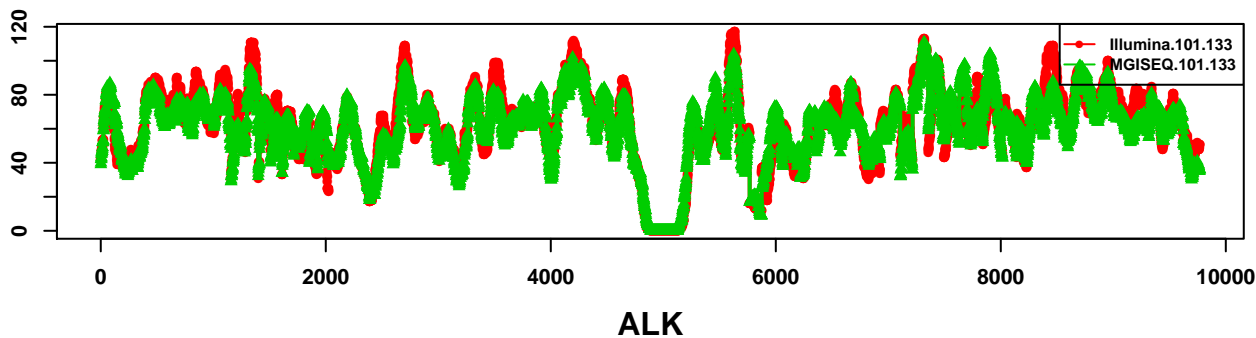

Sequencing Depth

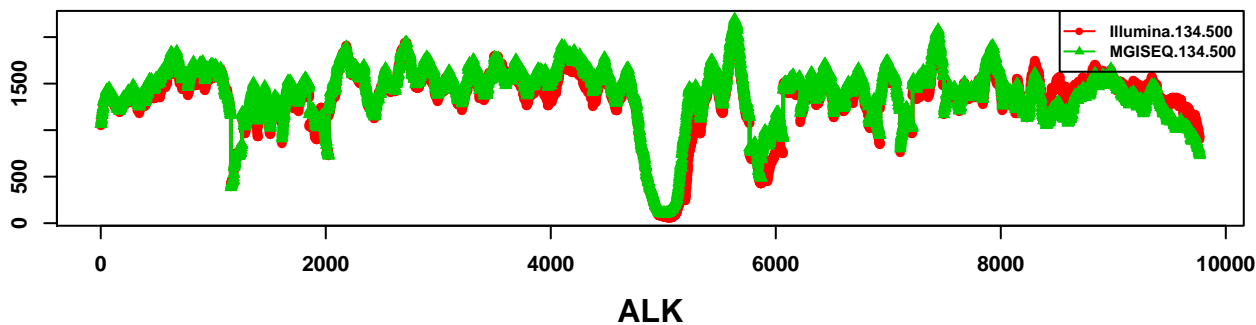

Supplement: Supplementary file 6 [file Presentation5.zip › ALK/19BZ58024P.pdf]

Sequencing Depth

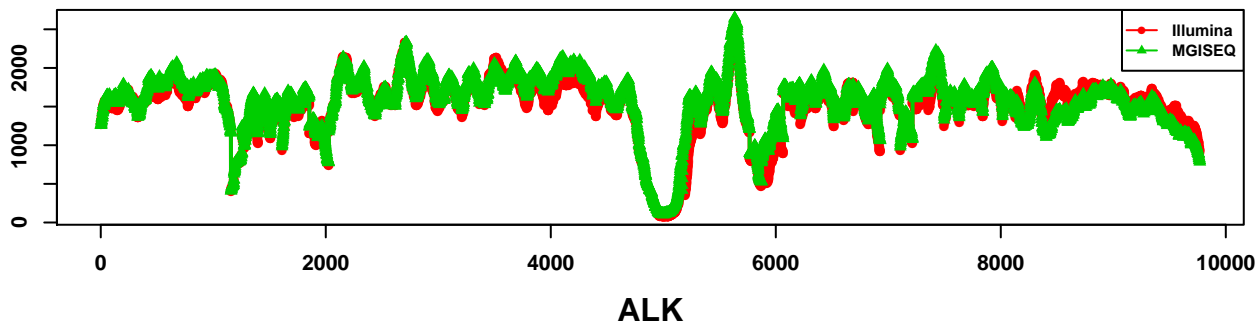

Sequencing Depth

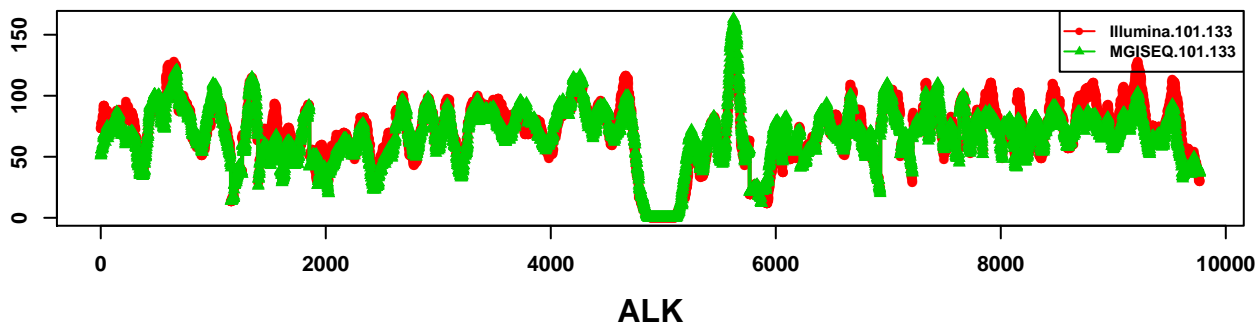

Sequencing Depth

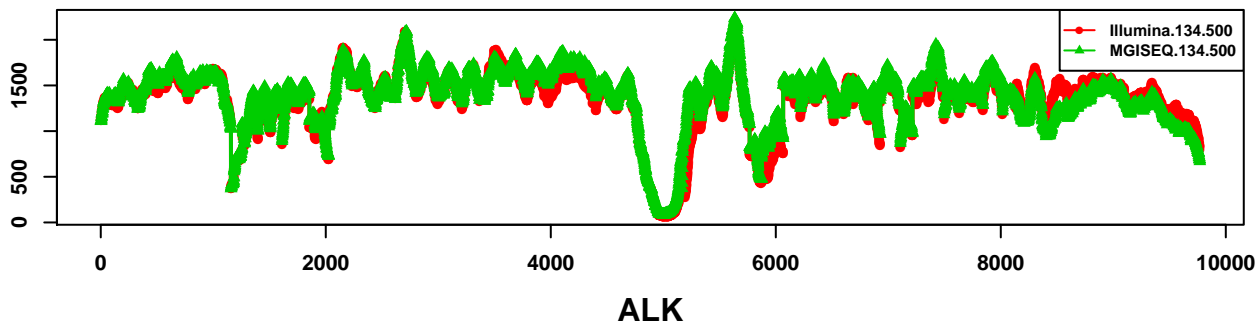

Supplement: Supplementary file 6 [file Presentation5.zip › ALK/19CF15710P.pdf]

Sequencing Depth

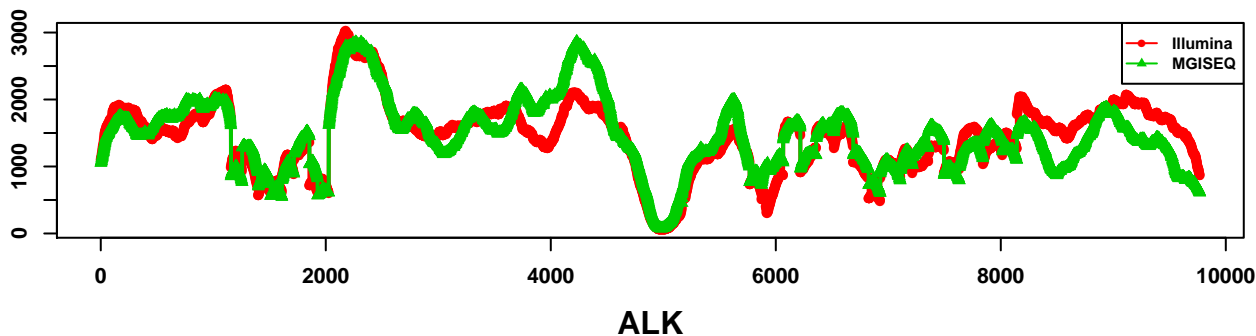

Sequencing Depth

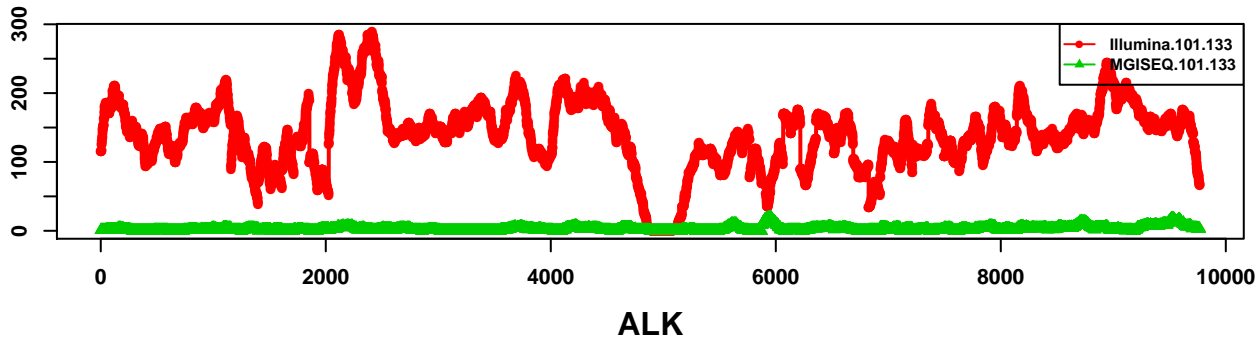

Sequencing Depth

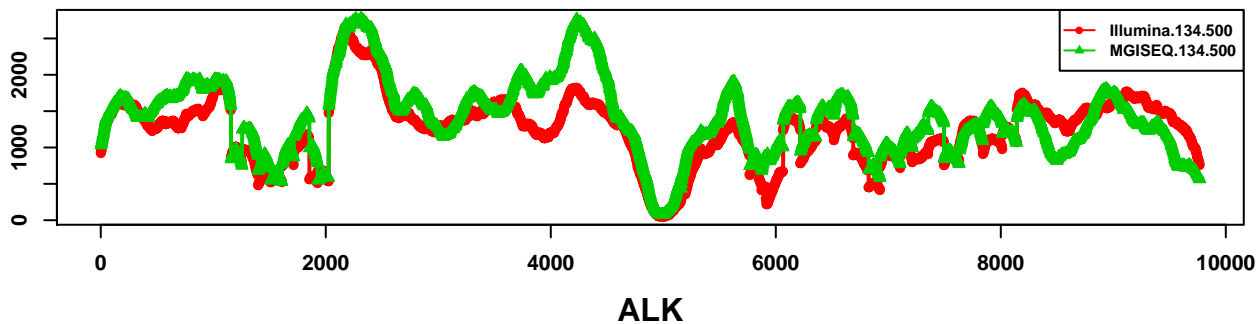

Supplement: Supplementary file 6 [file Presentation5.zip › ALK/19LN70341F.pdf]

Sequencing Depth

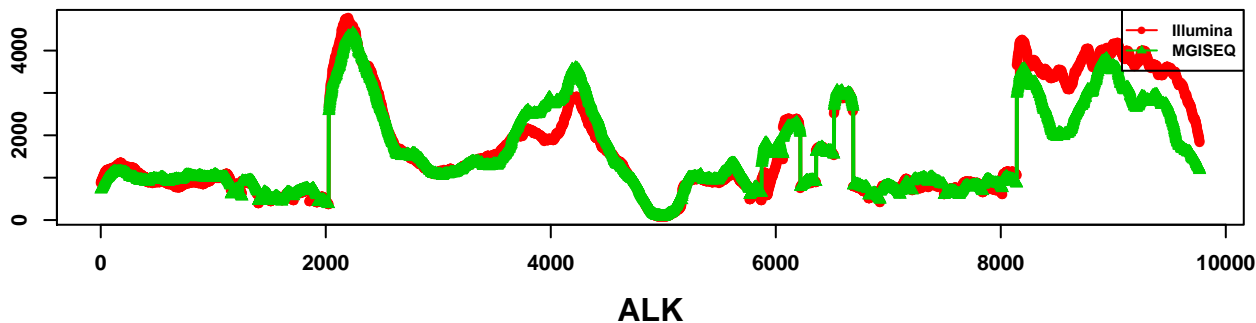

Sequencing Depth

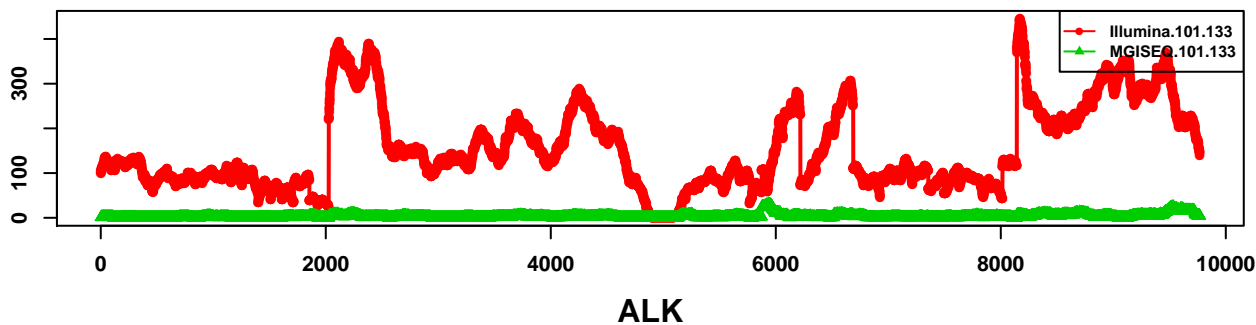

Sequencing Depth

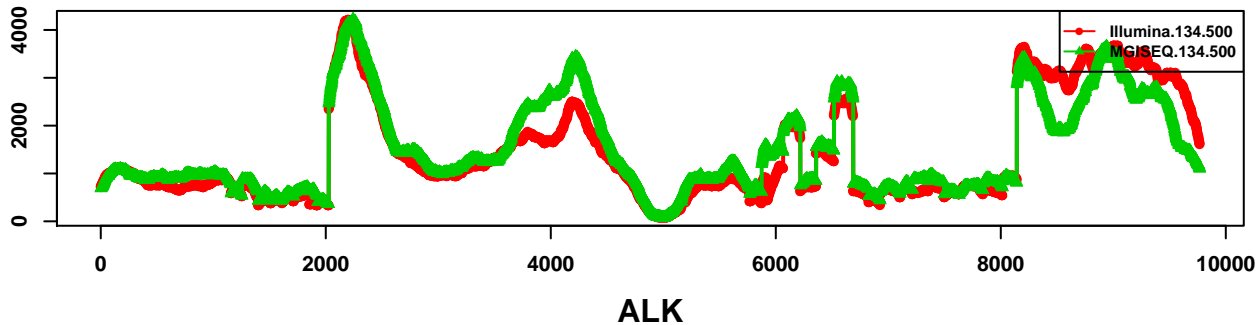

Supplement: Supplementary file 6 [file Presentation5.zip › ALK/19FC40247F.pdf]

Sequencing Depth

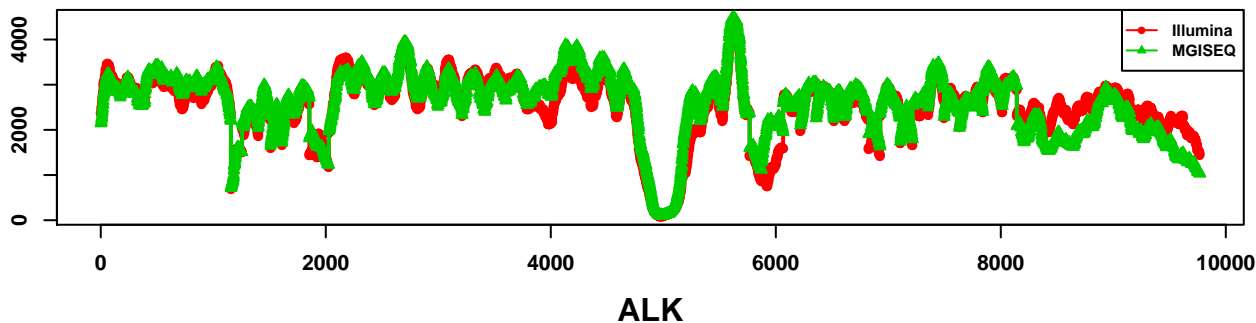

Sequencing Depth

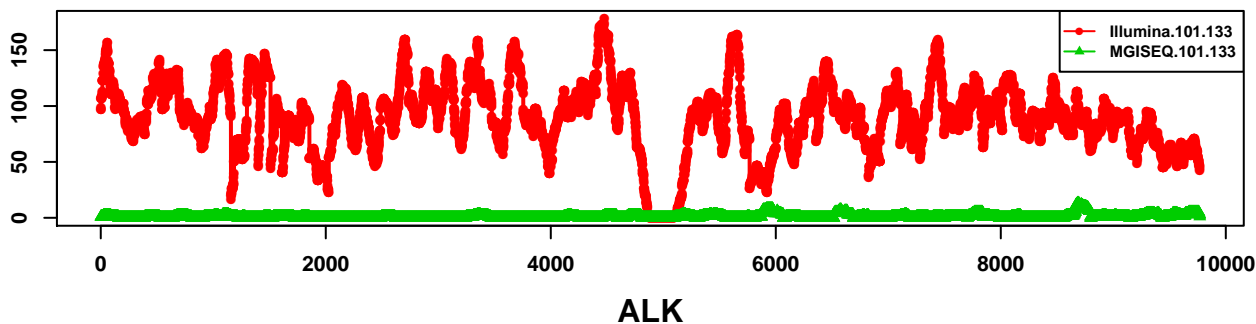

Sequencing Depth

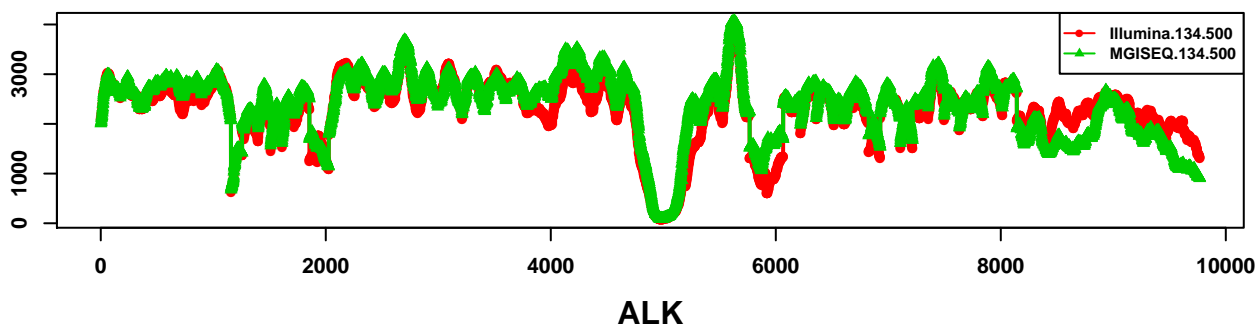

Supplement: Supplementary file 6 [file Presentation5.zip › ALK/19ZN12356P.pdf]

Sequencing Depth

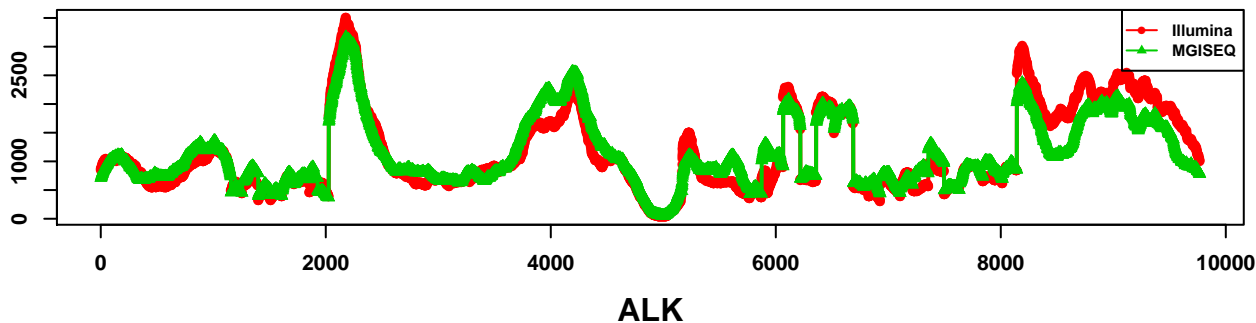

Sequencing Depth

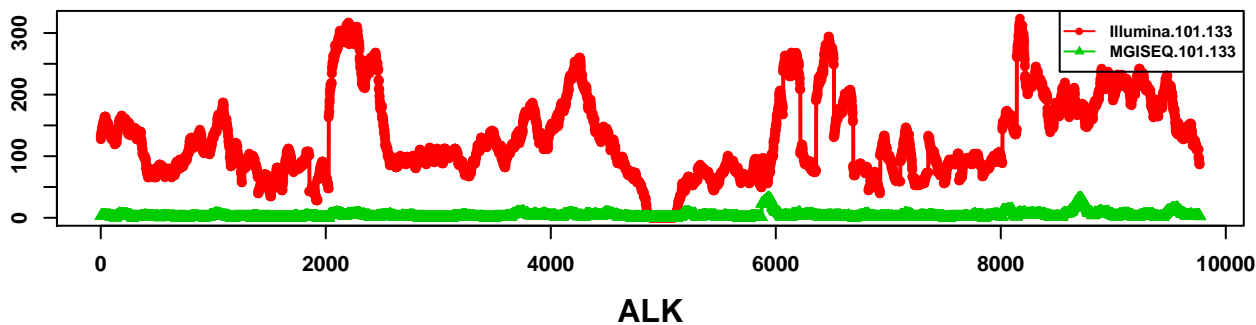

Sequencing Depth

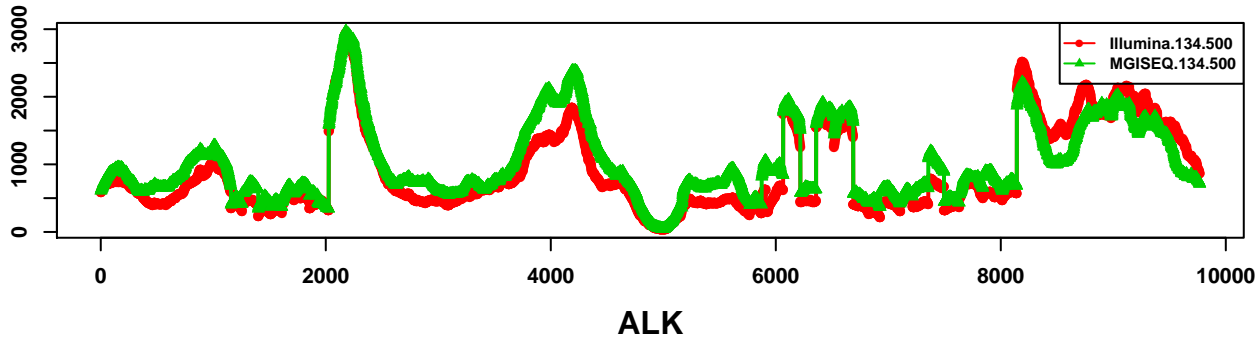

Supplement: Supplementary file 6 [file Presentation5.zip › ALK/19JM45577F.pdf]

Sequencing Depth

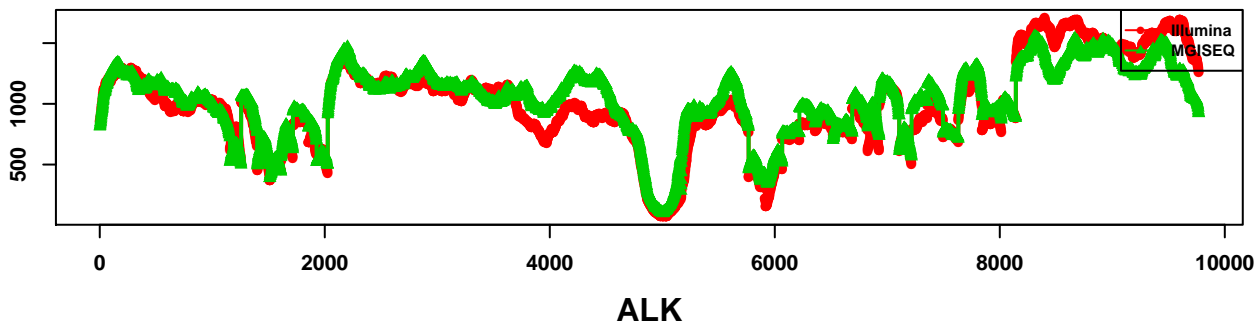

Sequencing Depth

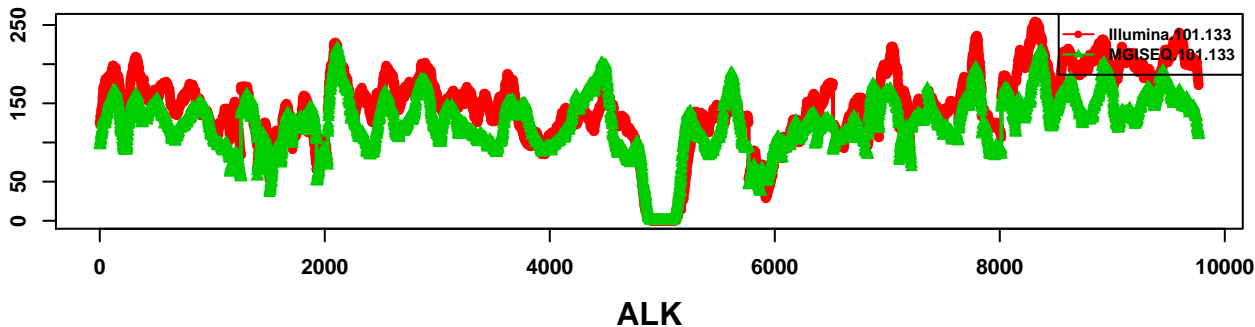

Sequencing Depth

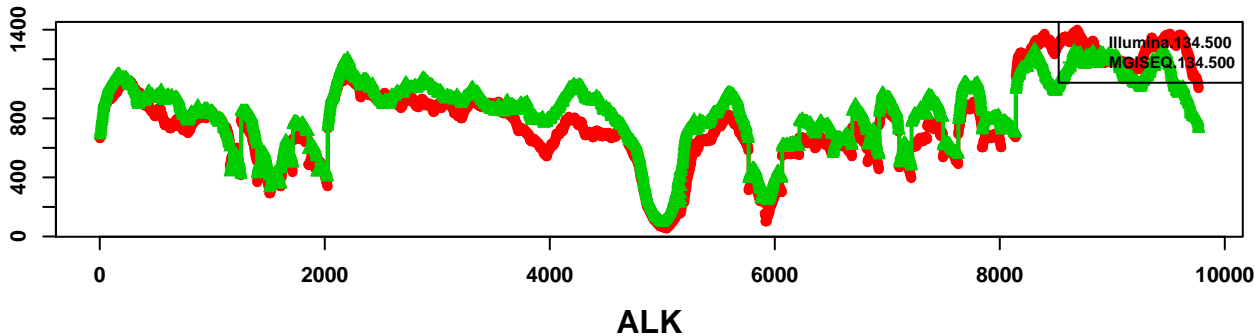

Supplement: Supplementary file 6 [file Presentation5.zip › ALK/19ZN12546F.pdf]

Sequencing Depth

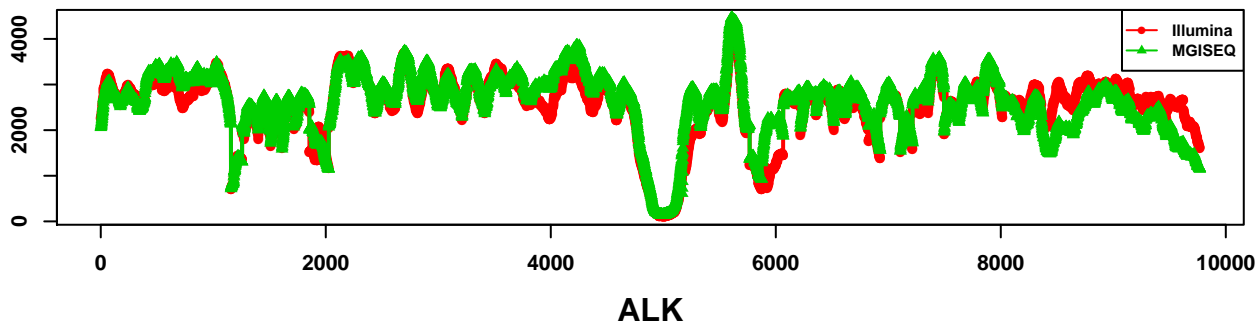

Sequencing Depth

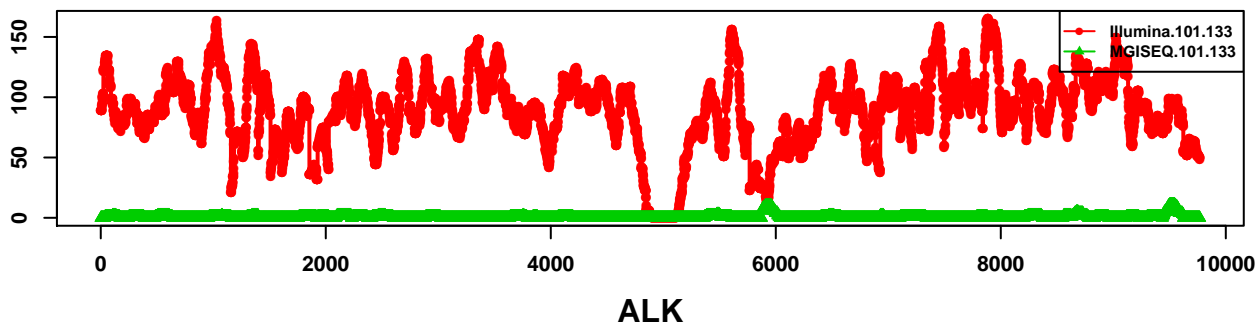

Sequencing Depth

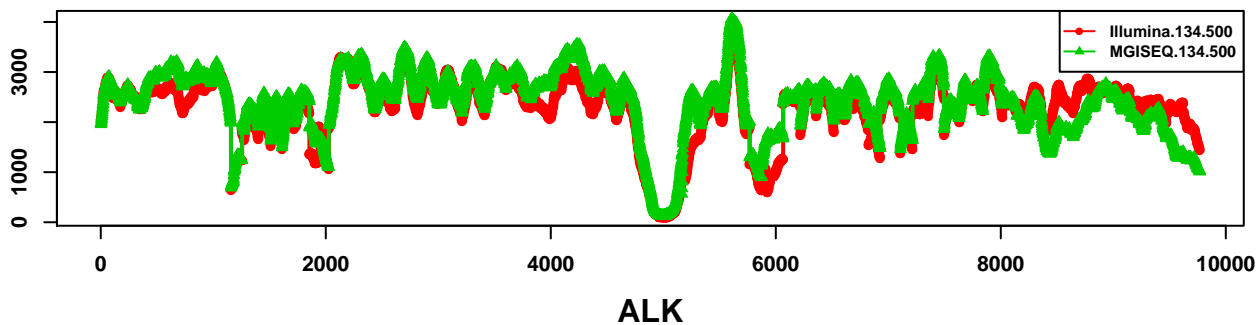

Supplement: Supplementary file 6 [file Presentation5.zip › ALK/M1900799-I-IIP.pdf]

Sequencing Depth

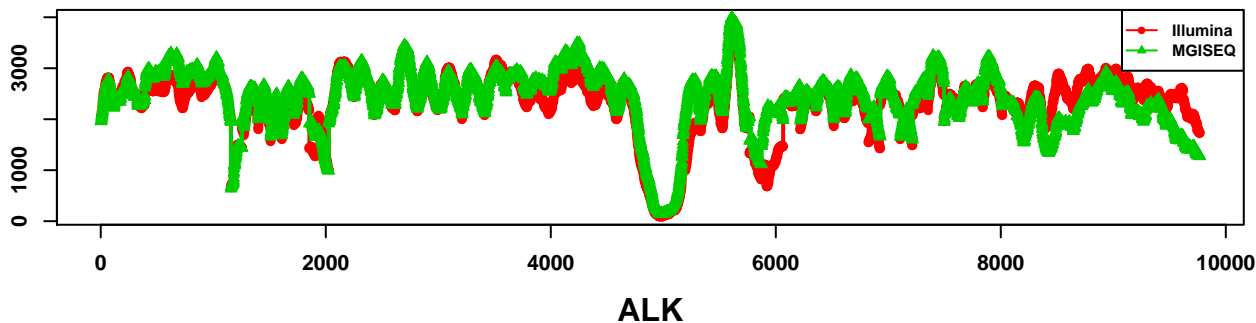

Sequencing Depth

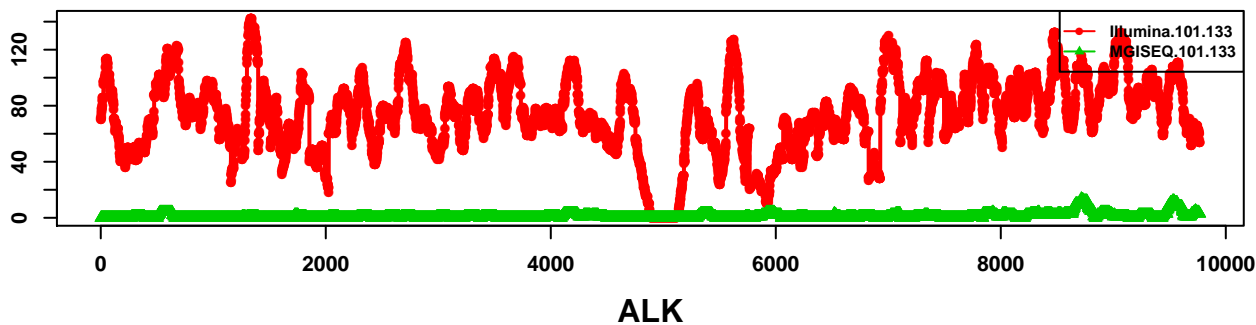

Sequencing Depth

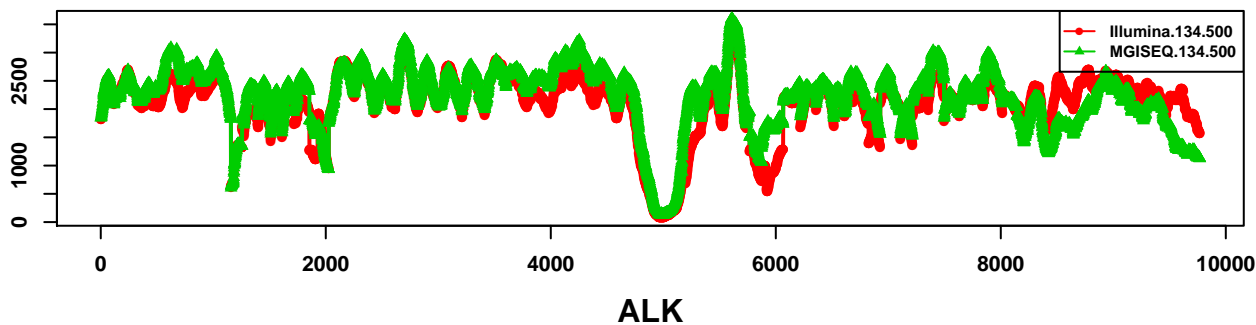

Supplement: Supplementary file 6 [file Presentation5.zip › ALK/19HE22140P.pdf]

Sequencing Depth

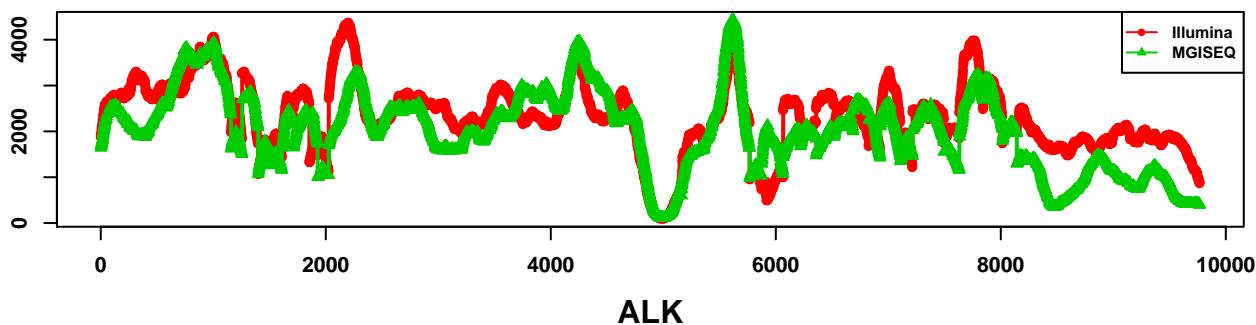

Sequencing Depth

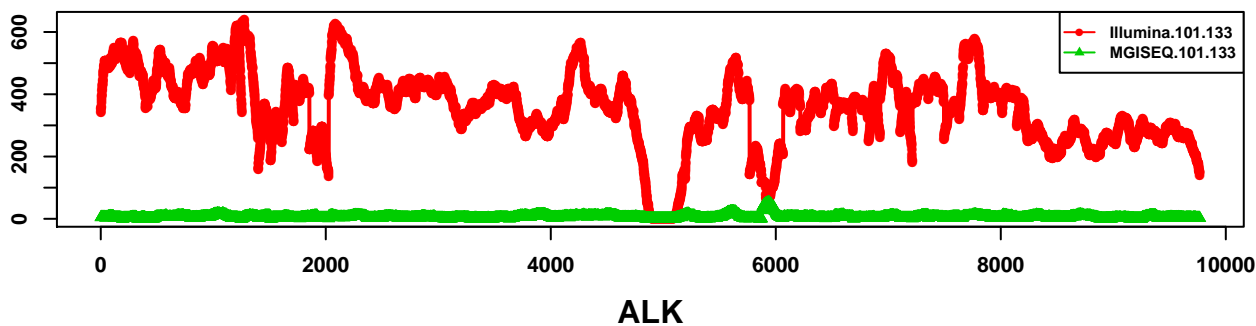

Sequencing Depth

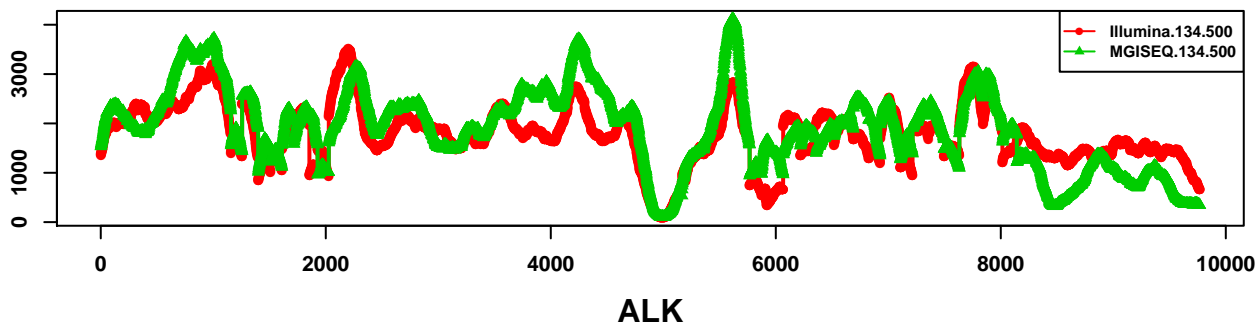

Supplement: Supplementary file 6 [file Presentation5.zip › ALK/19CF15531F.pdf]

Sequencing Depth

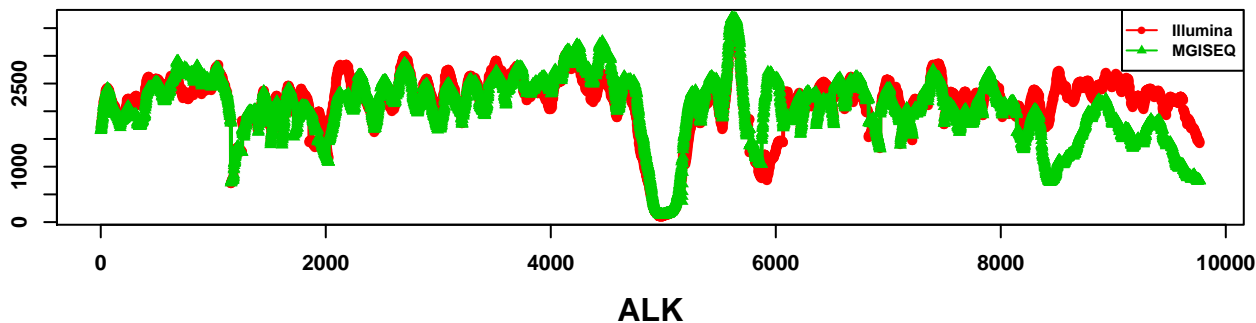

Sequencing Depth

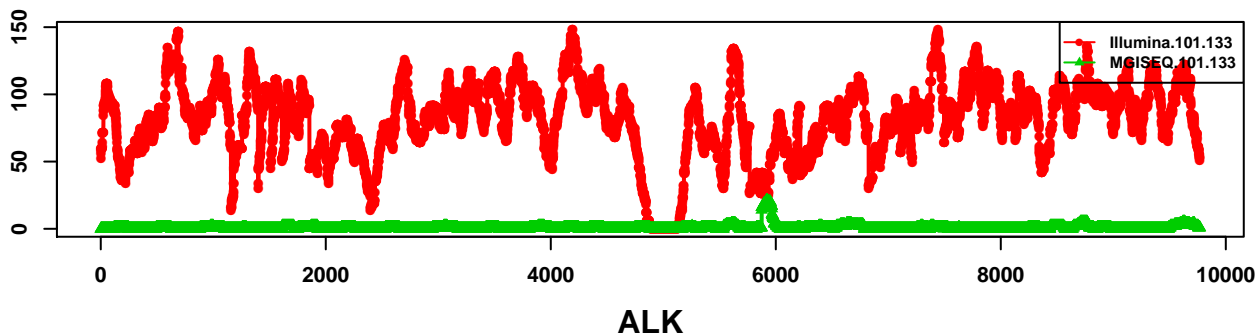

Sequencing Depth

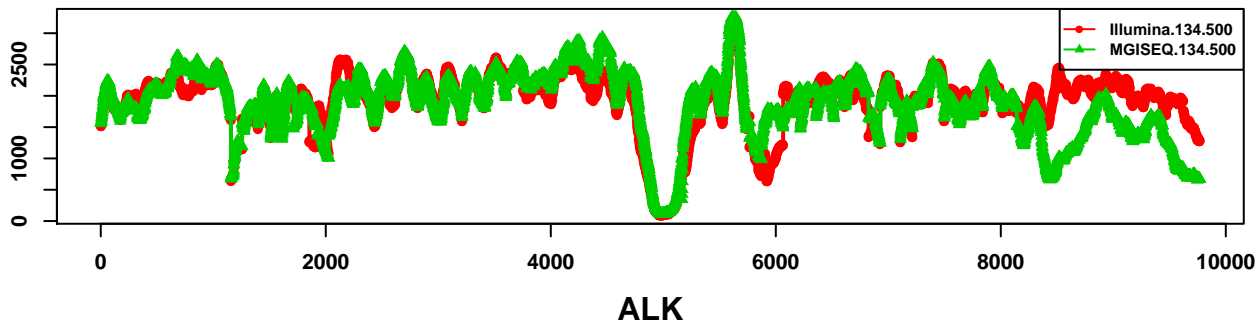

Supplement: Supplementary file 6 [file Presentation5.zip › ALK/M1900858-IP.pdf]

Sequencing Depth

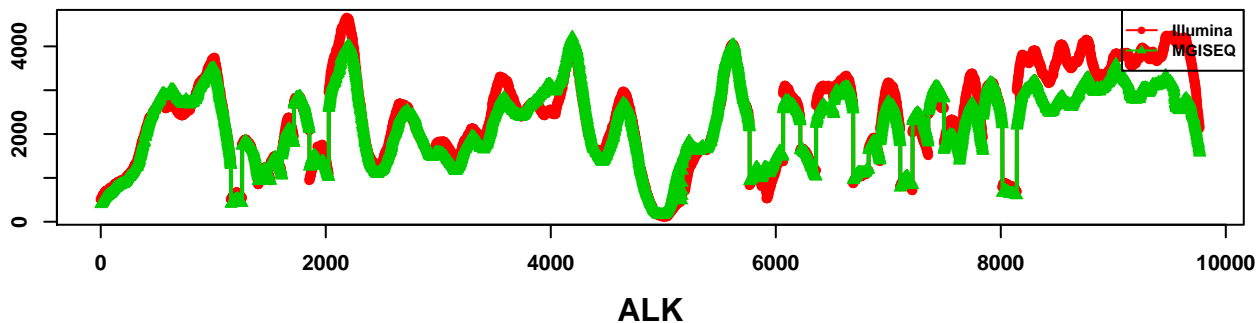

Sequencing Depth

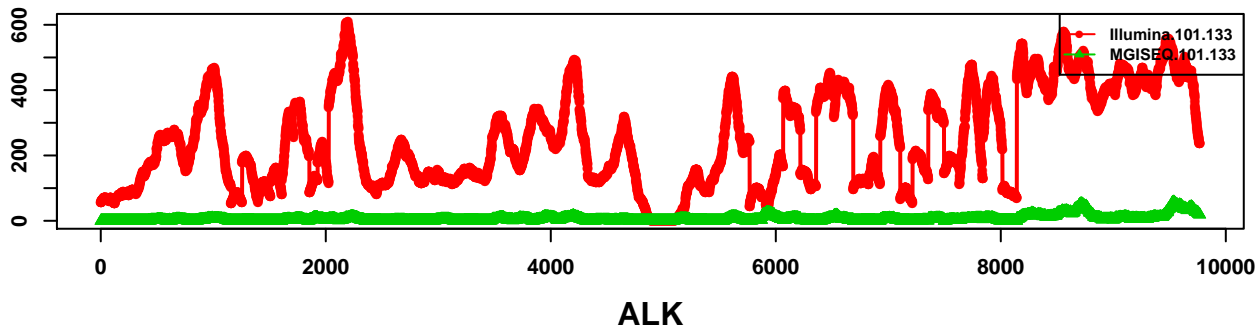

Sequencing Depth

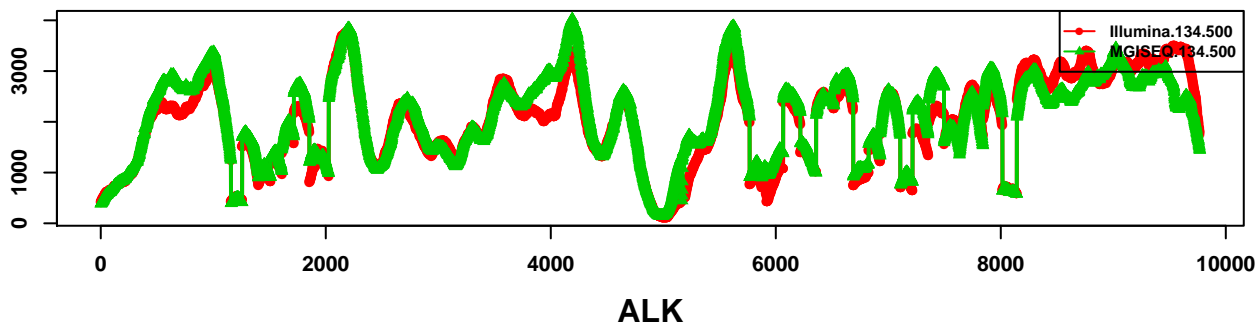

Supplement: Supplementary file 6 [file Presentation5.zip › ALK/19N01368F.pdf]

Sequencing Depth

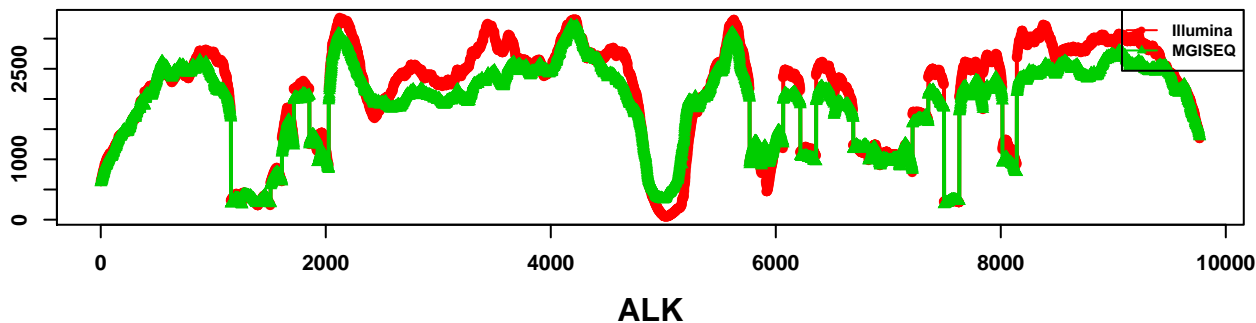

Sequencing Depth

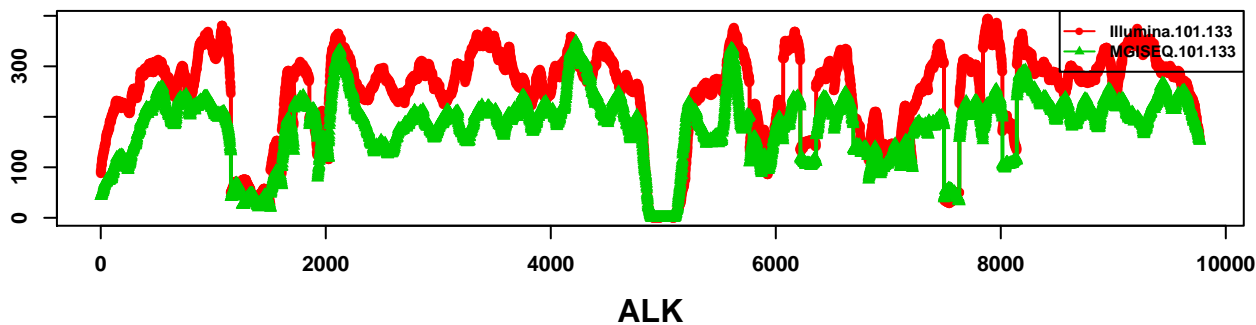

Sequencing Depth

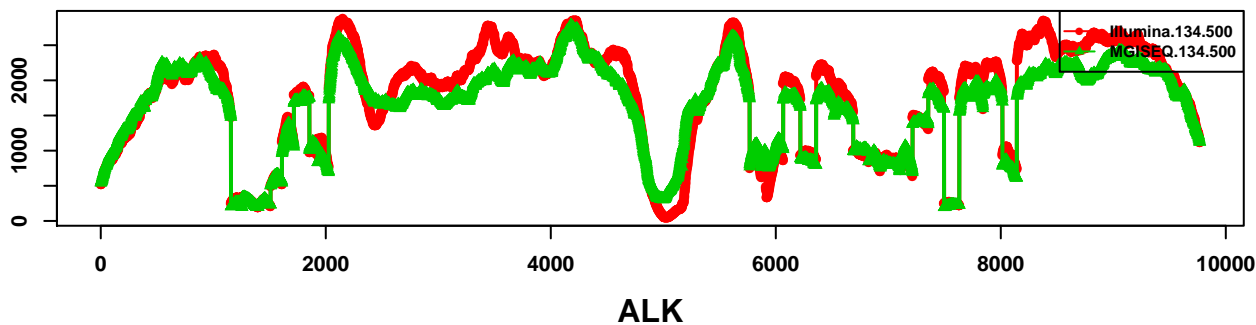

Supplement: Supplementary file 6 [file Presentation5.zip › ALK/19ZN12592T.pdf]

Sequencing Depth

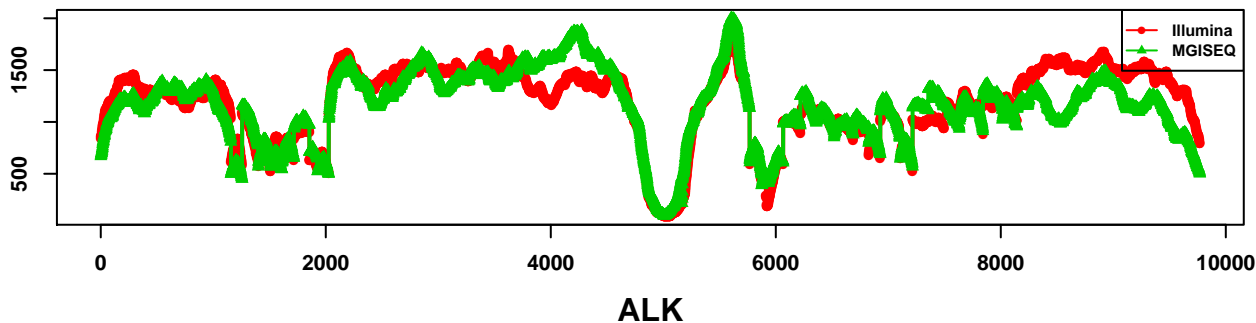

Sequencing Depth

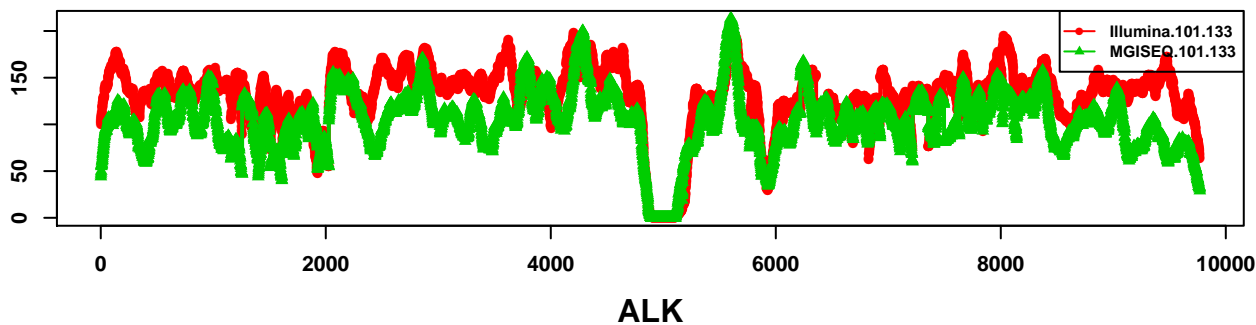

Sequencing Depth

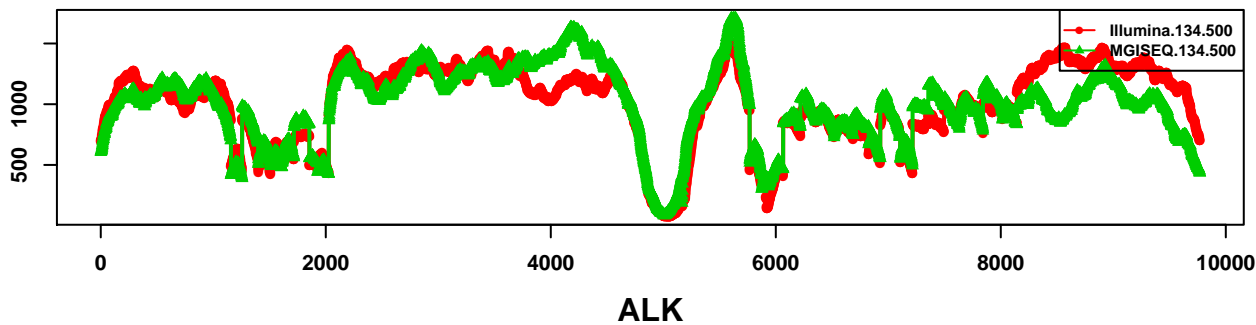

Supplement: Supplementary file 6 [file Presentation5.zip › ALK/ZK190805-G.pdf]

Sequencing Depth

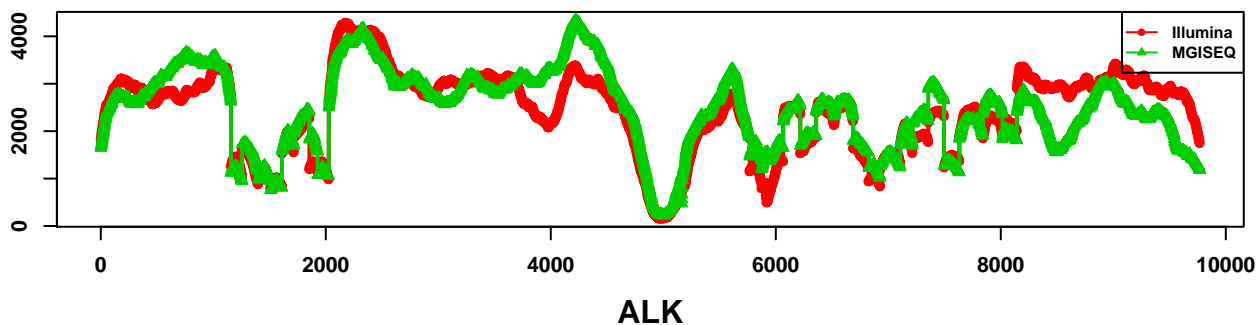

Sequencing Depth

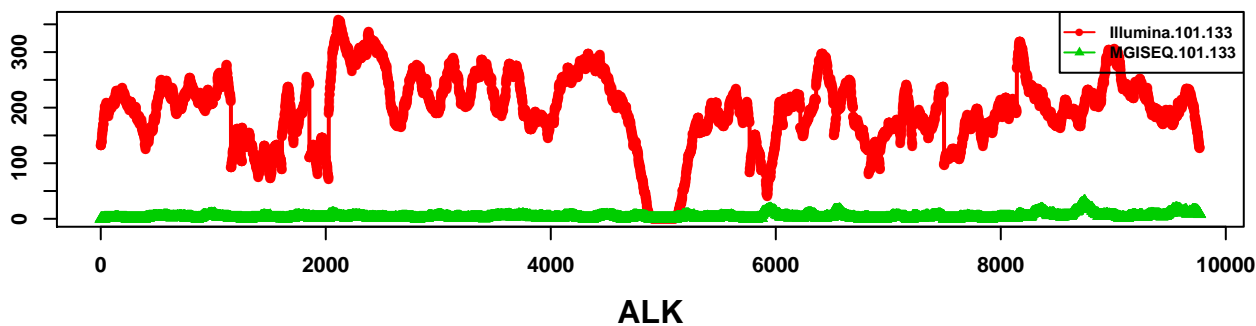

Sequencing Depth

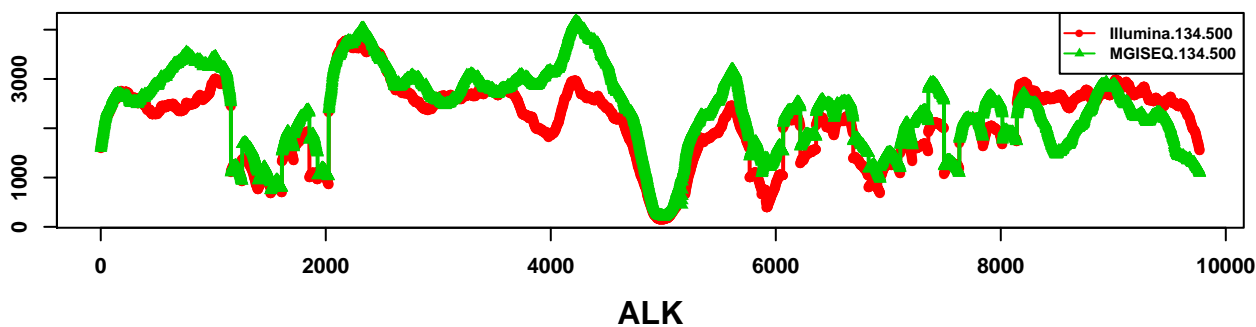

Supplement: Supplementary file 6 [file Presentation5.zip › ALK/19N01651F.pdf]

Sequencing Depth

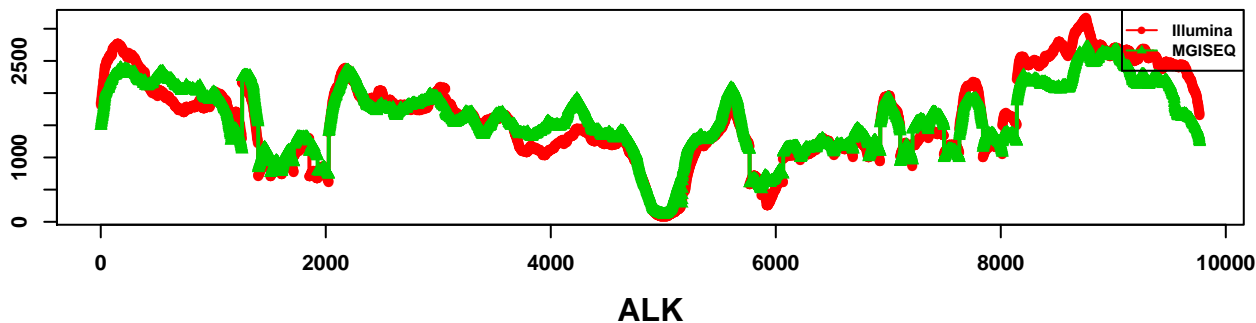

Sequencing Depth

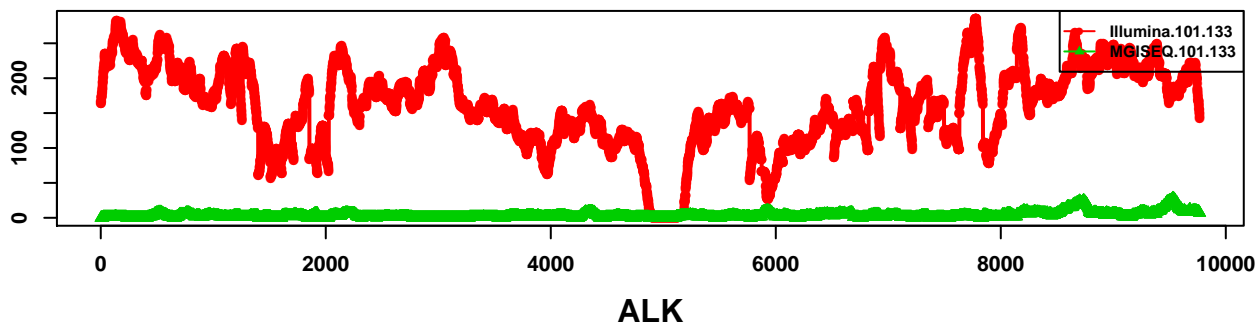

Sequencing Depth

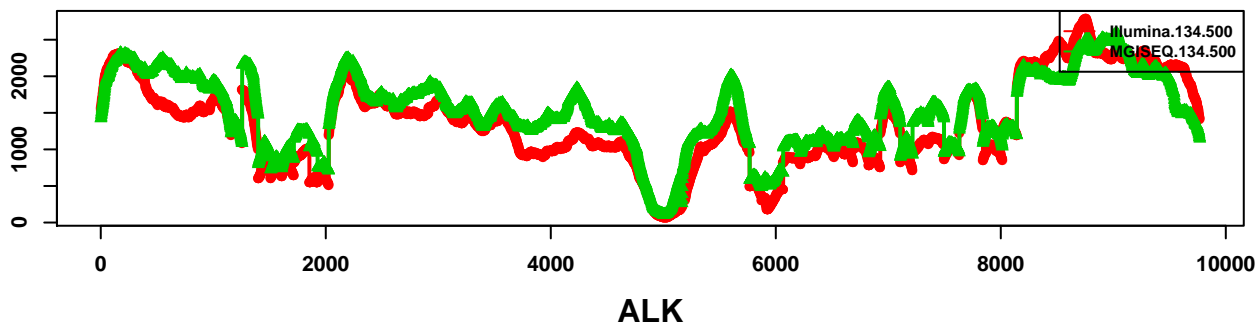

Supplement: Supplementary file 6 [file Presentation5.zip › ALK/19HE22005F.pdf]

Sequencing Depth

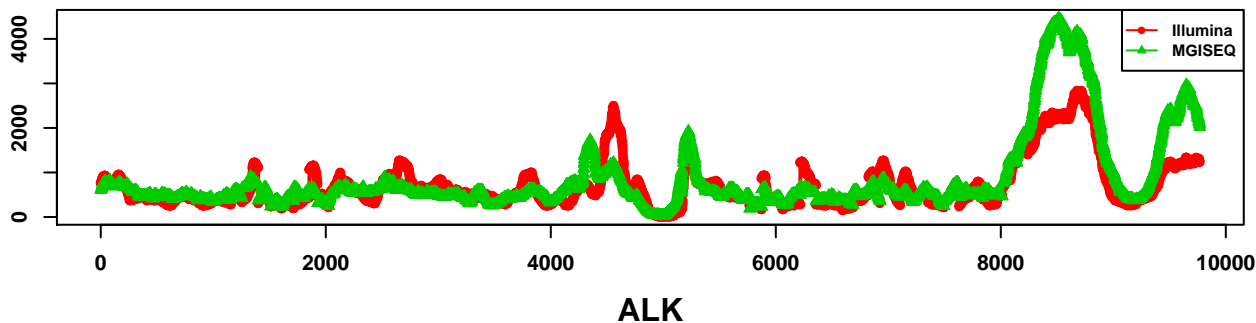

Sequencing Depth

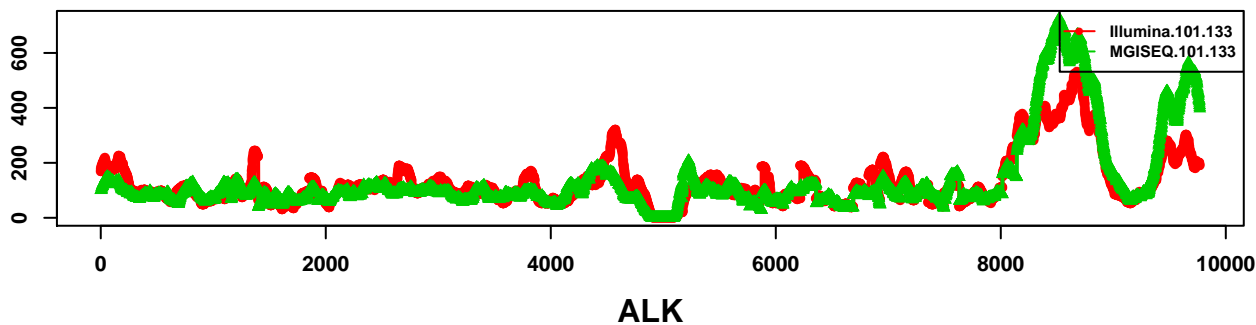

Sequencing Depth

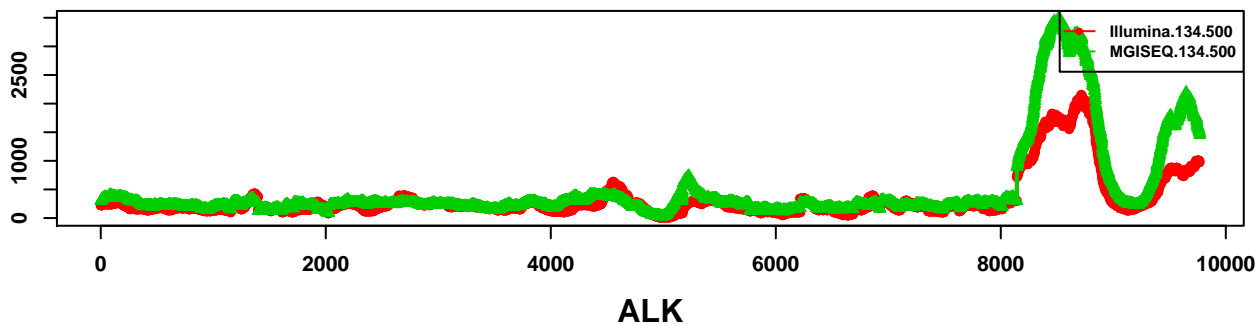

Supplement: Supplementary file 6 [file Presentation5.zip › ALK/19FC40420F.pdf]
