# Supplementary figures and images for: Evaluation of the MGISEQ-2000 Sequencing Platform for Illumina Target Capture Sequencing Libraries (part 5 of 6)
Source: Front Genet. 2021 Oct 27;12:730519. doi: 10.3389/fgene.2021.730519 (PMC8578046; doi:10.3389/fgene.2021.730519)

Sequencing Depth

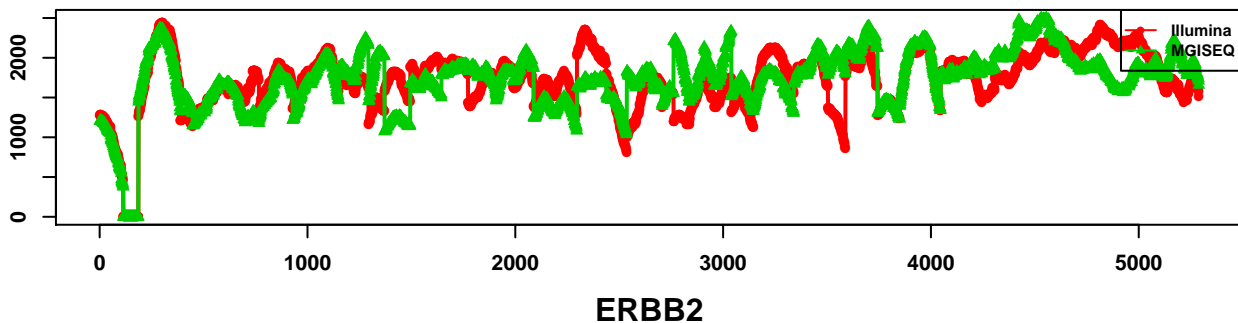

Sequencing Depth

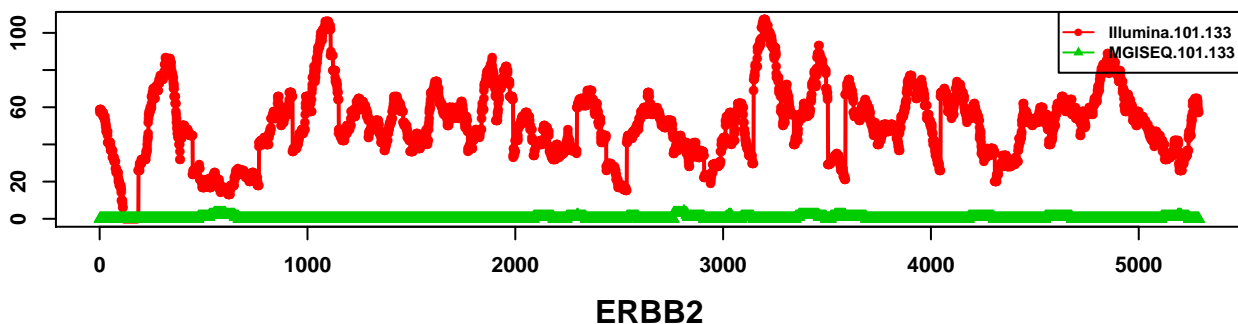

Sequencing Depth

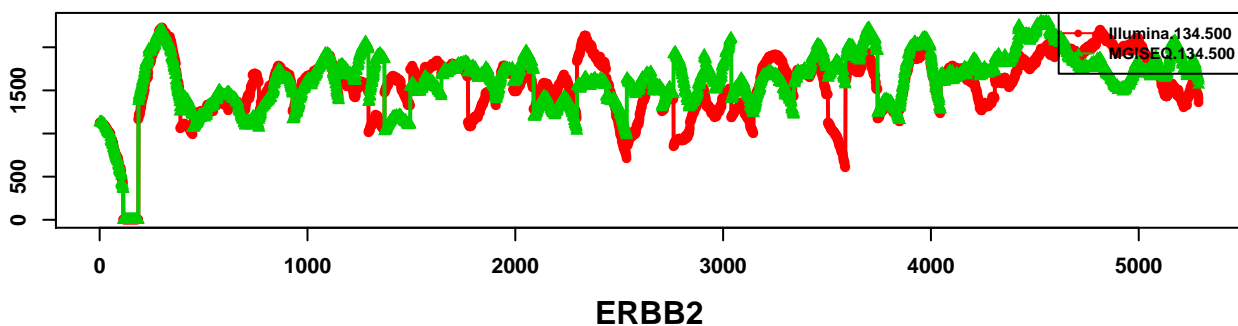

Supplement: Supplementary file 7 [file Presentation7.zip › ERBB2/19HS86080P.pdf]

Sequencing Depth

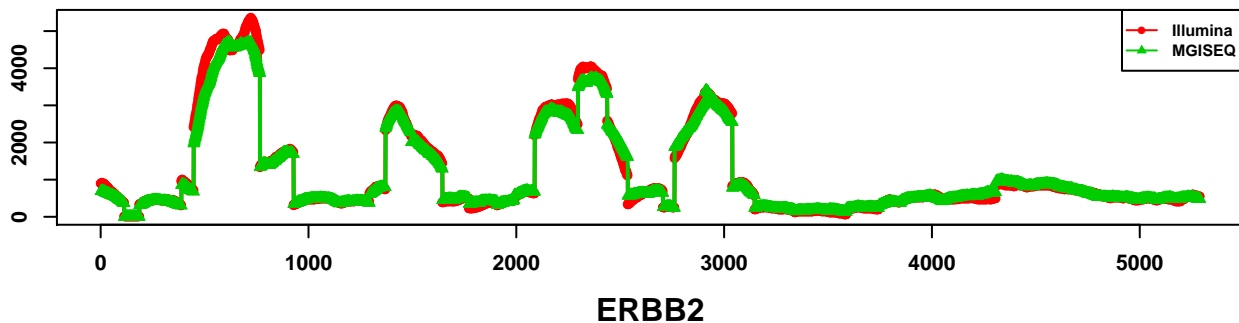

Sequencing Depth

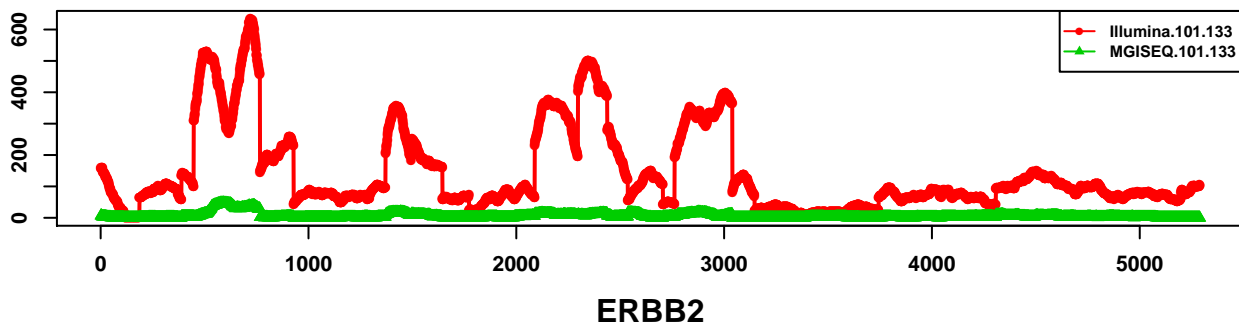

Sequencing Depth

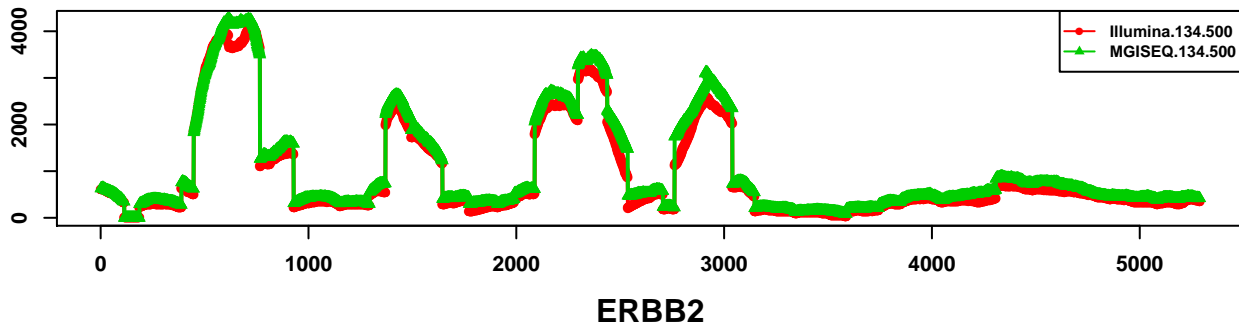

Supplement: Supplementary file 7 [file Presentation7.zip › ERBB2/19N01949F.pdf]

Sequencing Depth

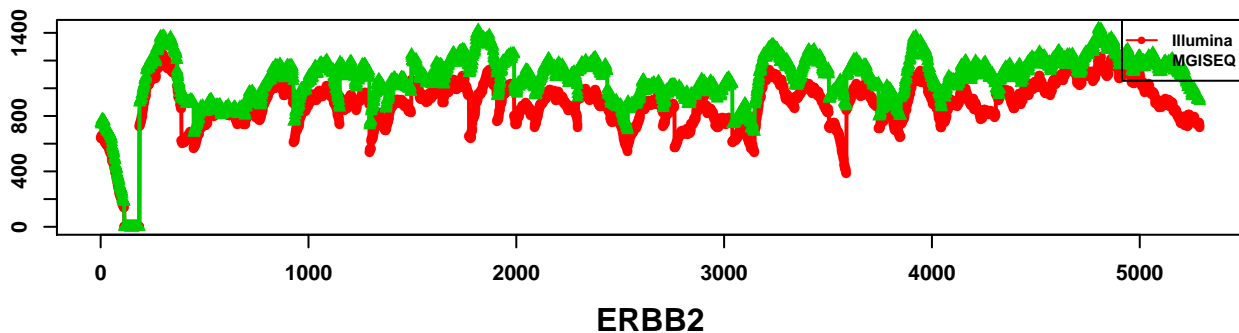

Sequencing Depth

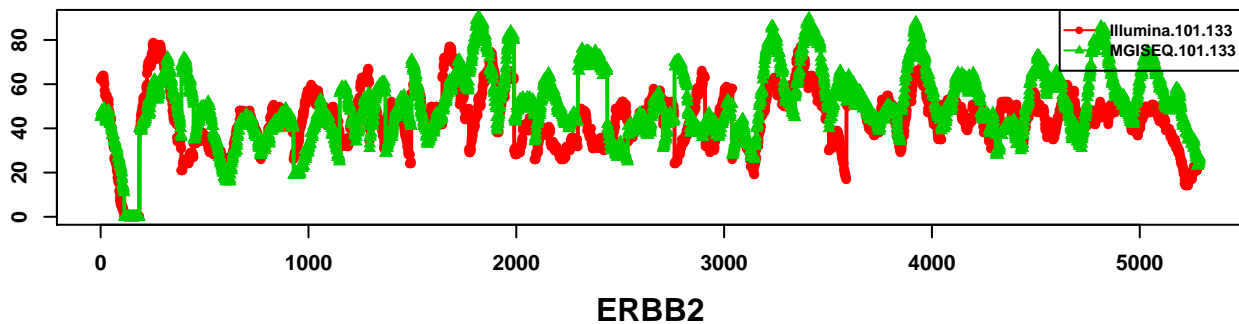

Sequencing Depth

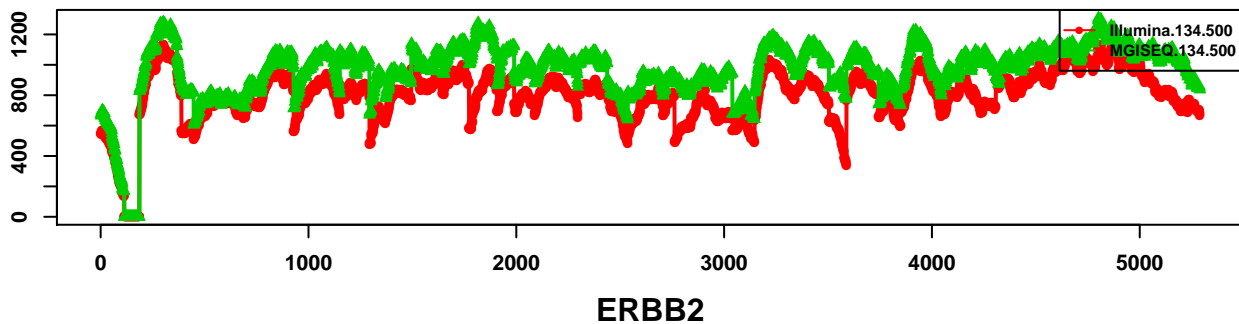

Supplement: Supplementary file 7 [file Presentation7.zip › ERBB2/19ZN12259P.pdf]

Sequencing Depth

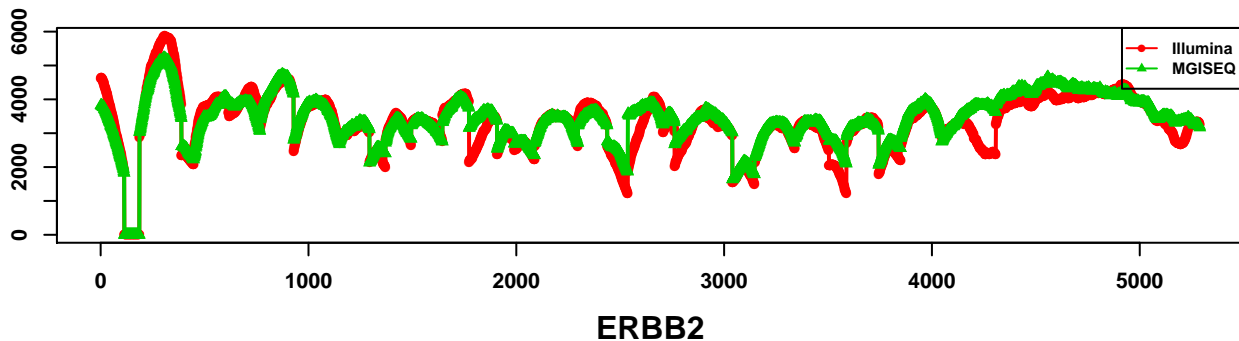

Sequencing Depth

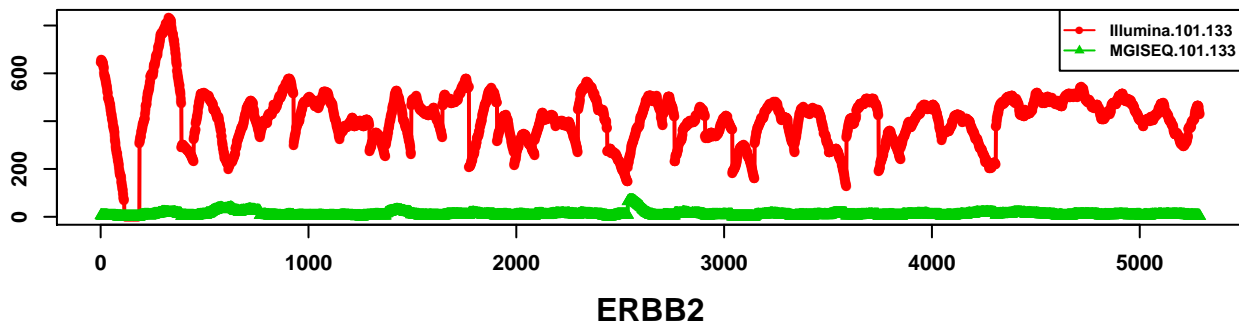

Sequencing Depth

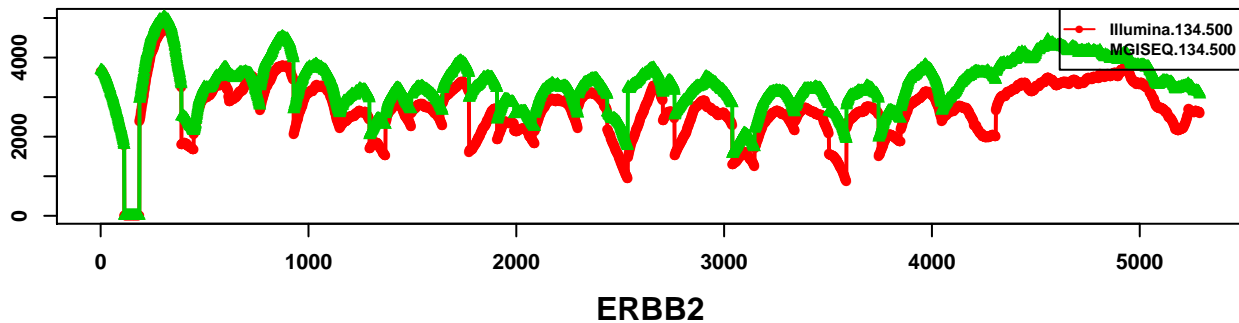

Supplement: Supplementary file 7 [file Presentation7.zip › ERBB2/19ZN12367F.pdf]

Sequencing Depth

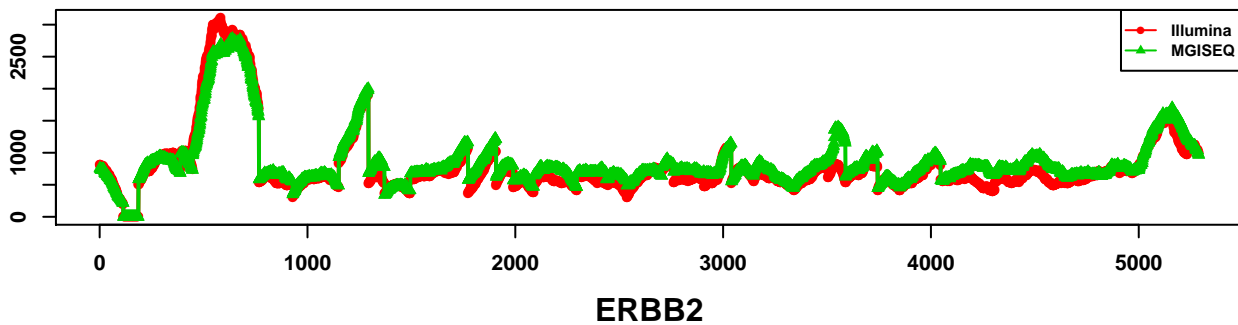

Sequencing Depth

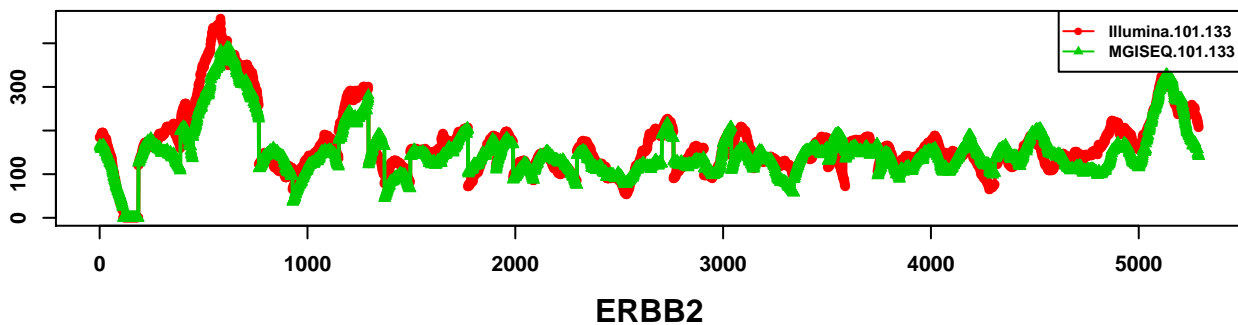

Sequencing Depth

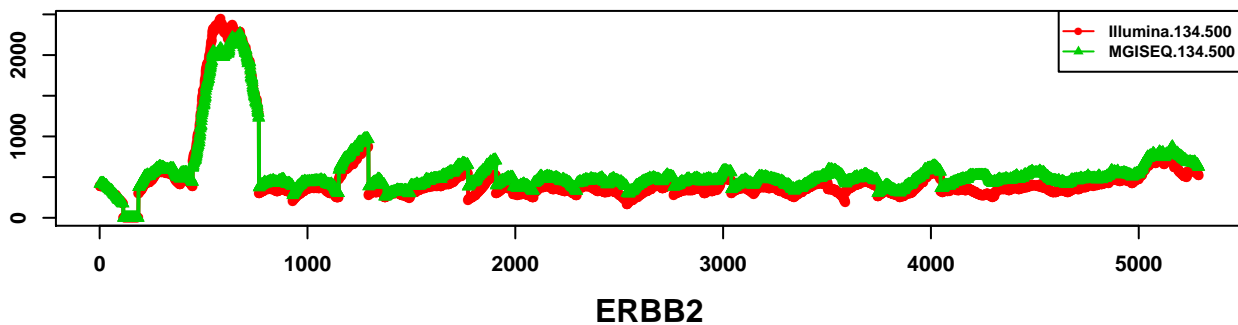

Supplement: Supplementary file 7 [file Presentation7.zip › ERBB2/19N02332F.pdf]

Sequencing Depth

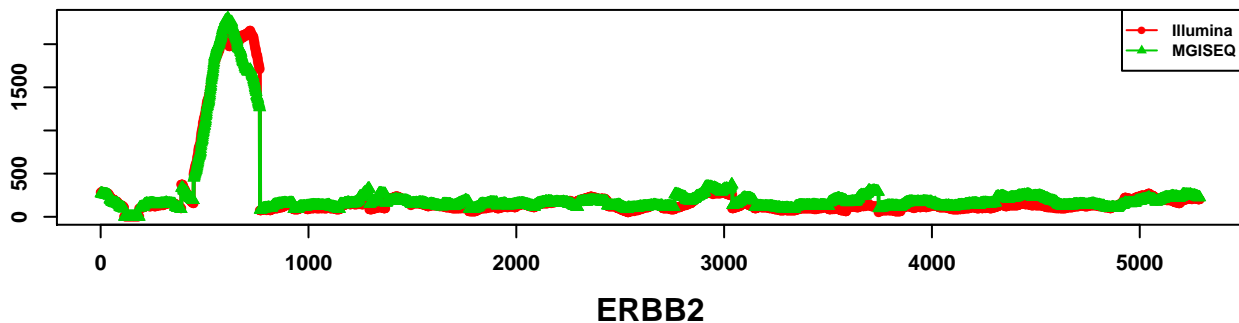

Sequencing Depth

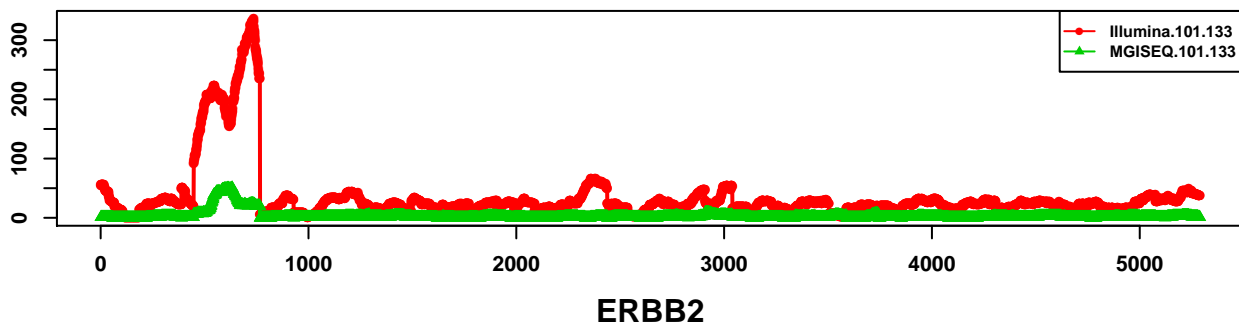

Sequencing Depth

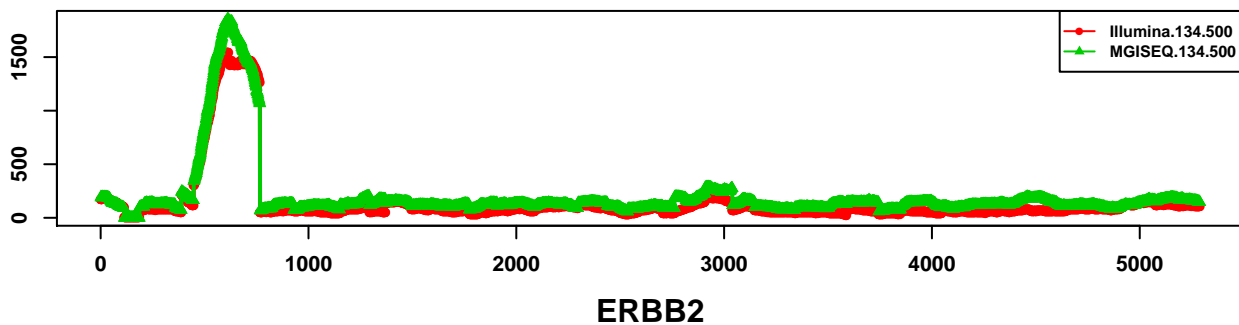

Supplement: Supplementary file 7 [file Presentation7.zip › ERBB2/19ZN12371F.pdf]

Sequencing Depth

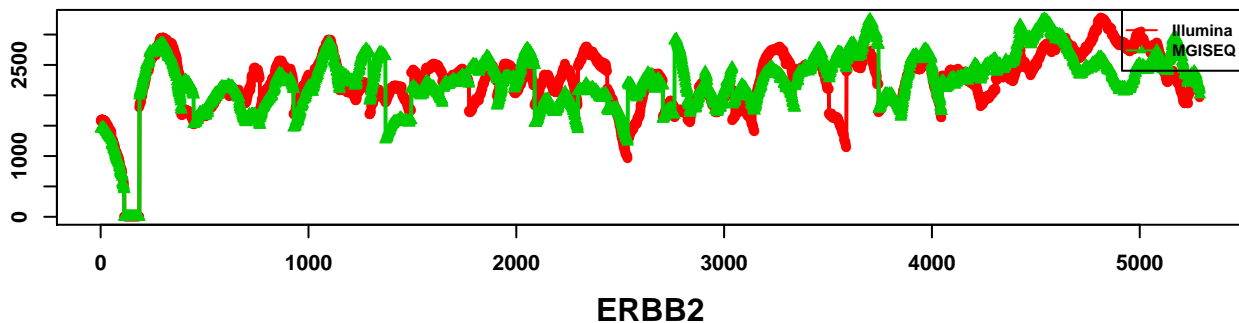

Sequencing Depth

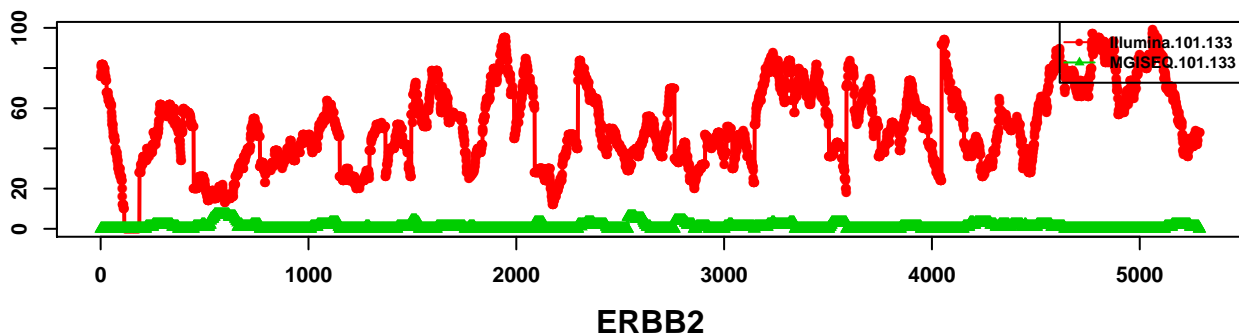

Sequencing Depth

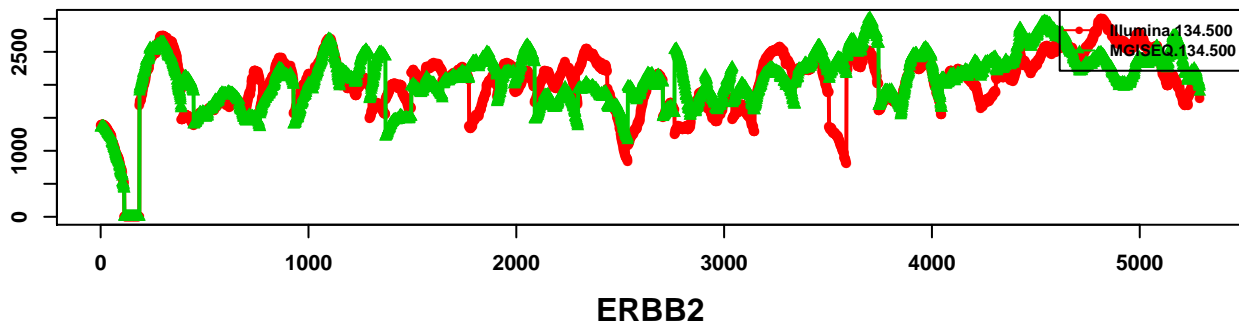

Supplement: Supplementary file 7 [file Presentation7.zip › ERBB2/19HS86079P.pdf]

Sequencing Depth

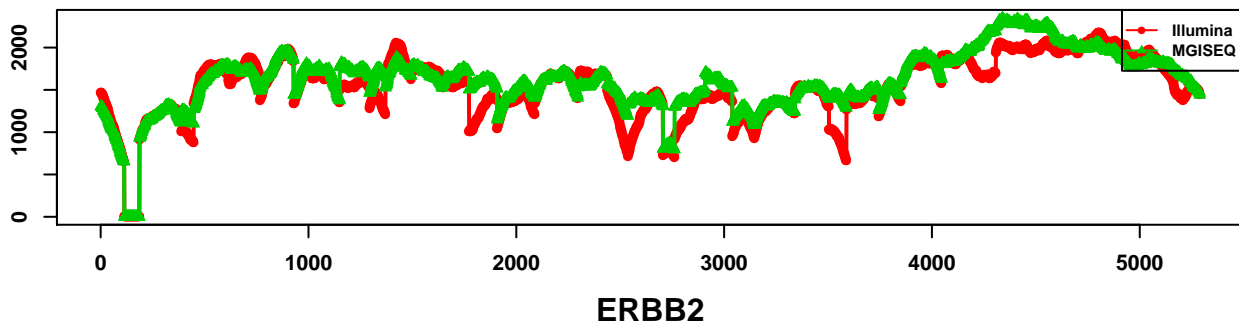

Sequencing Depth

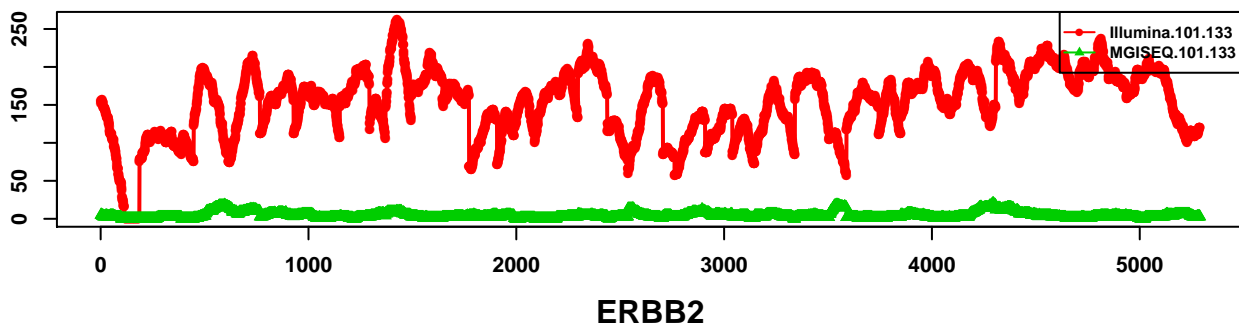

Sequencing Depth

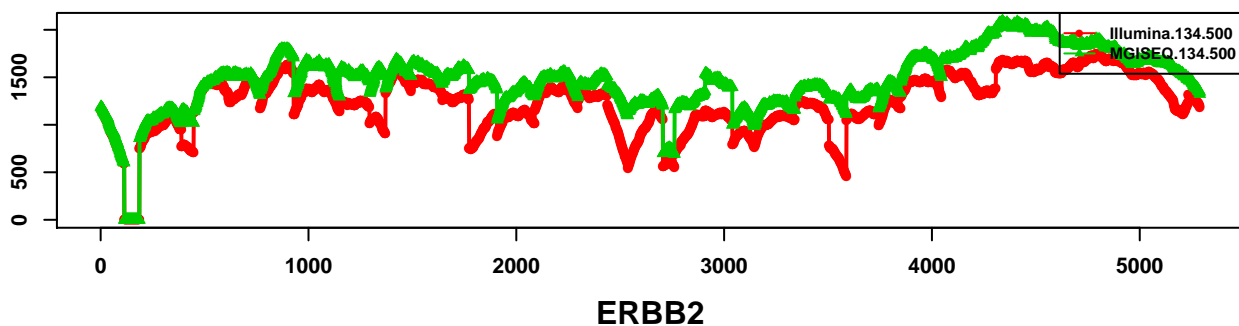

Supplement: Supplementary file 7 [file Presentation7.zip › ERBB2/19N01660F.pdf]

Sequencing Depth

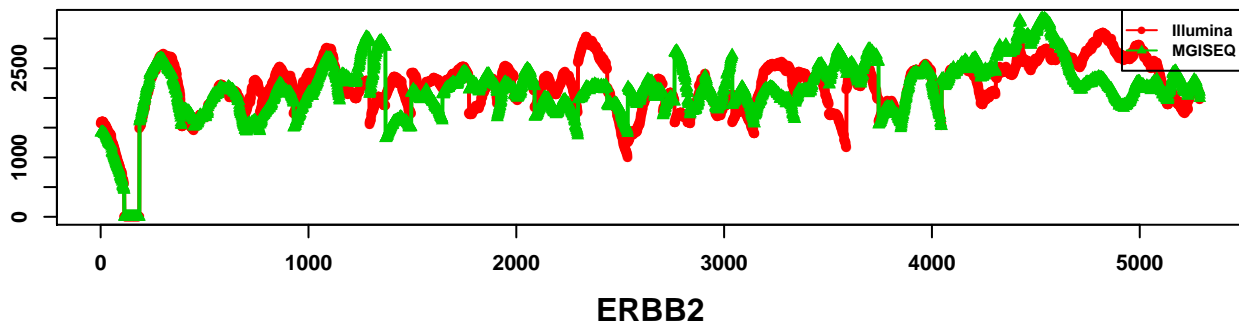

Sequencing Depth

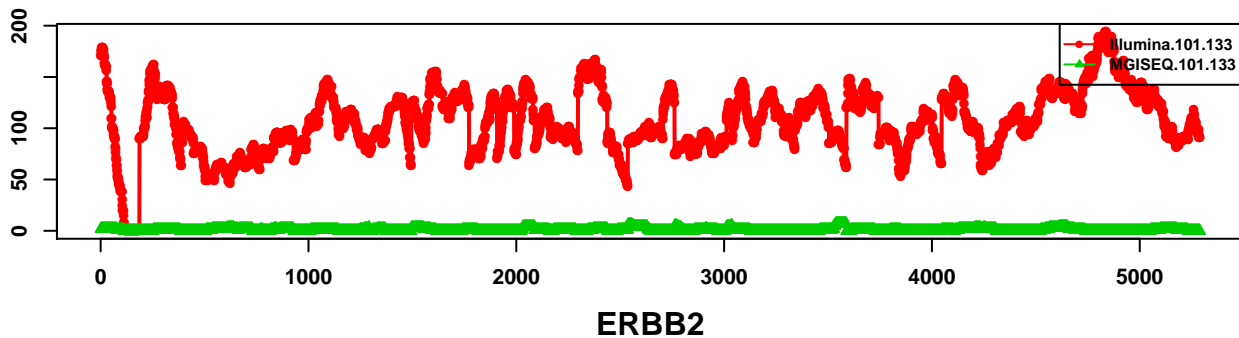

Sequencing Depth

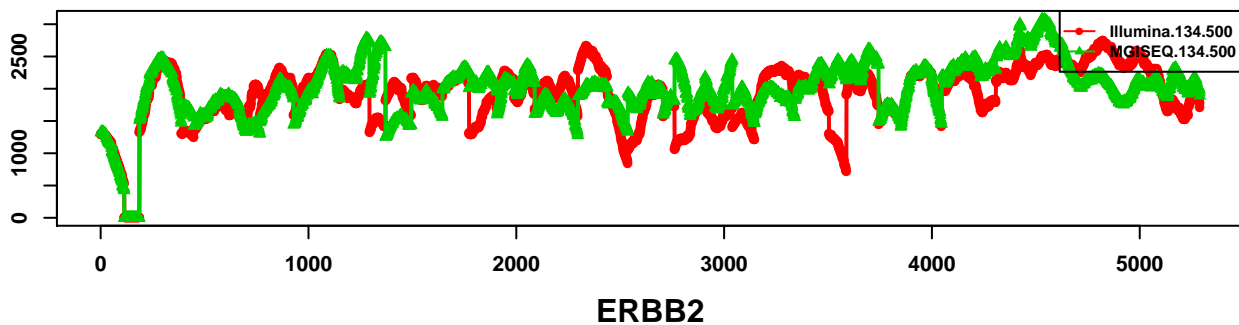

Supplement: Supplementary file 7 [file Presentation7.zip › ERBB2/19GY94045P.pdf]

Sequencing Depth

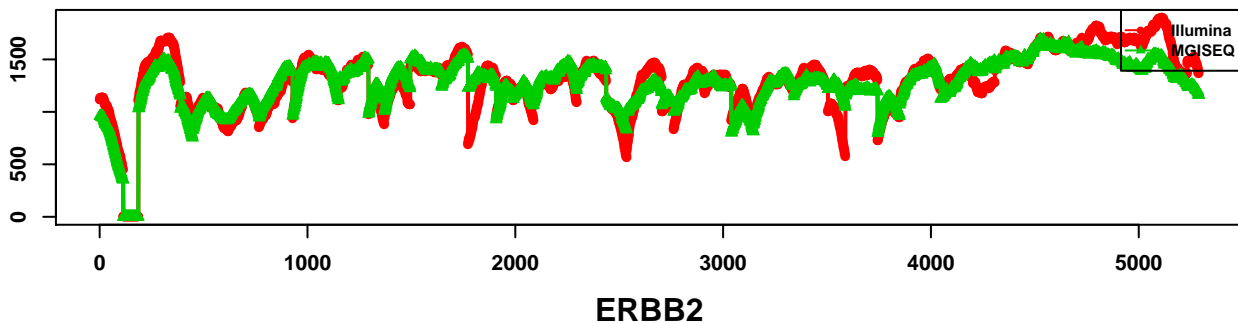

Sequencing Depth

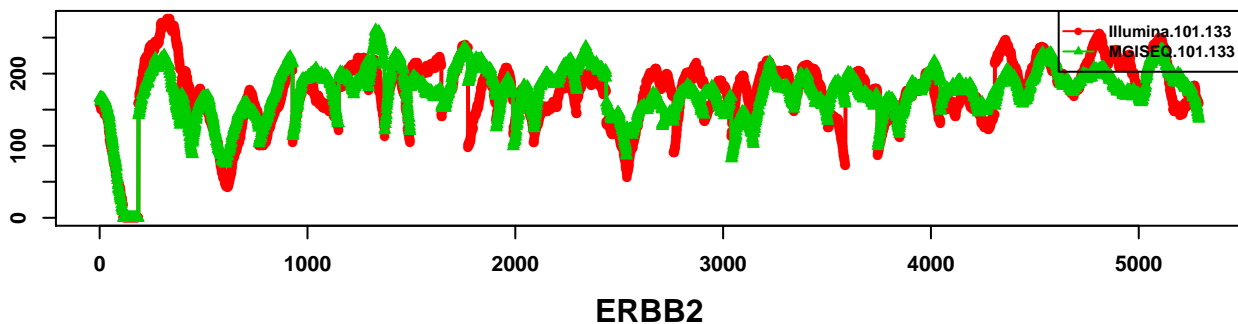

Sequencing Depth

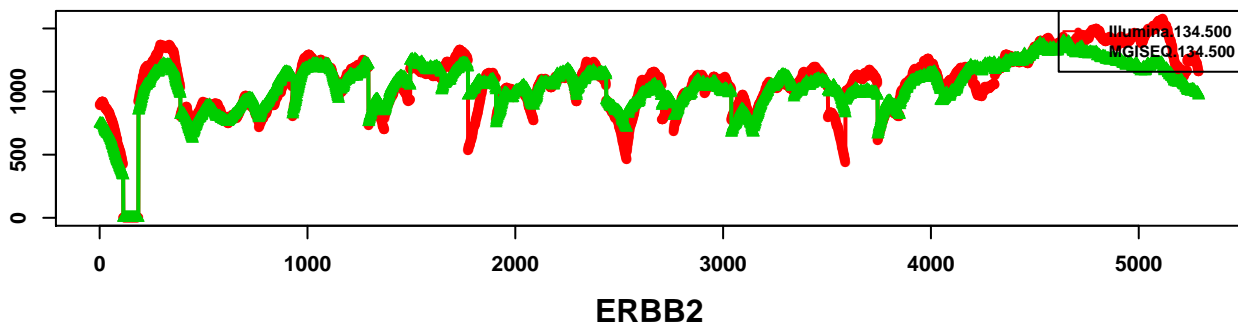

Supplement: Supplementary file 7 [file Presentation7.zip › ERBB2/19HE22805F.pdf]

Sequencing Depth

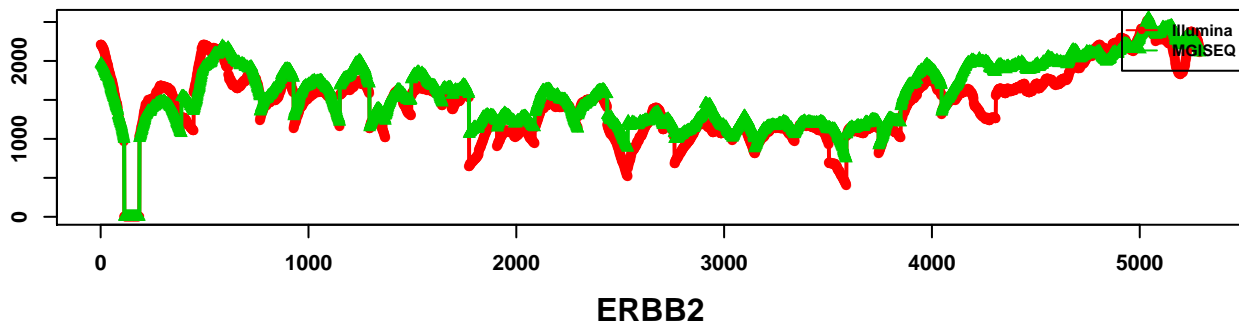

Sequencing Depth

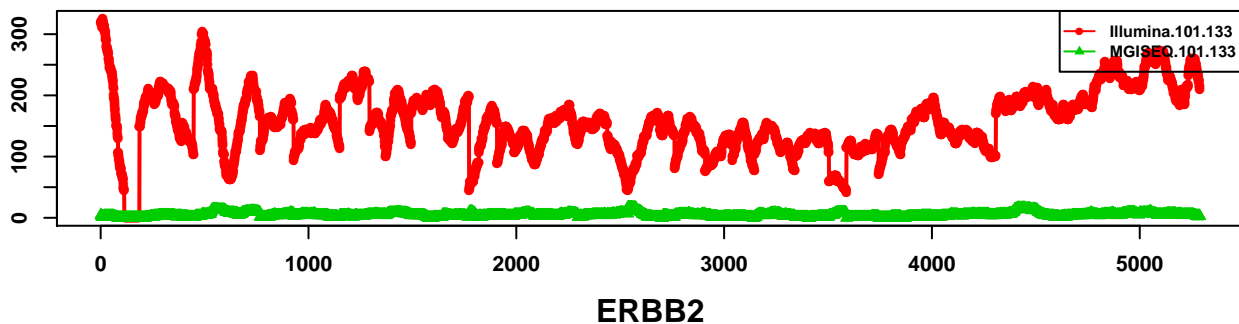

Sequencing Depth

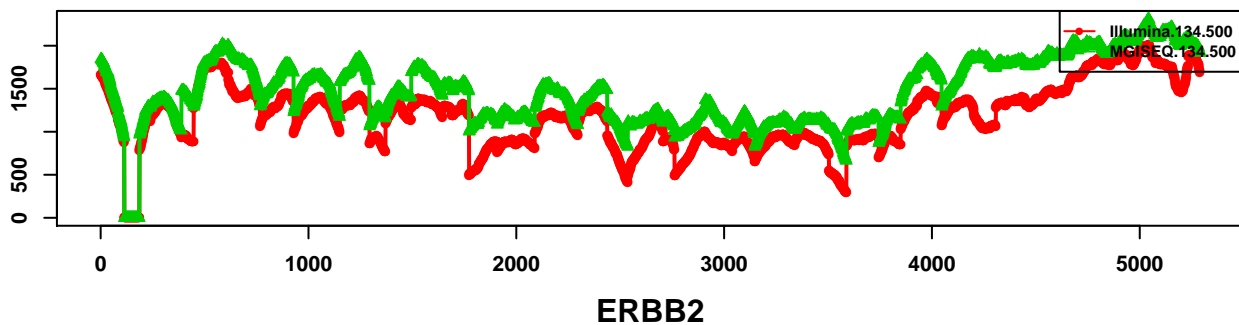

Supplement: Supplementary file 7 [file Presentation7.zip › ERBB2/19HE21950F.pdf]

Sequencing Depth

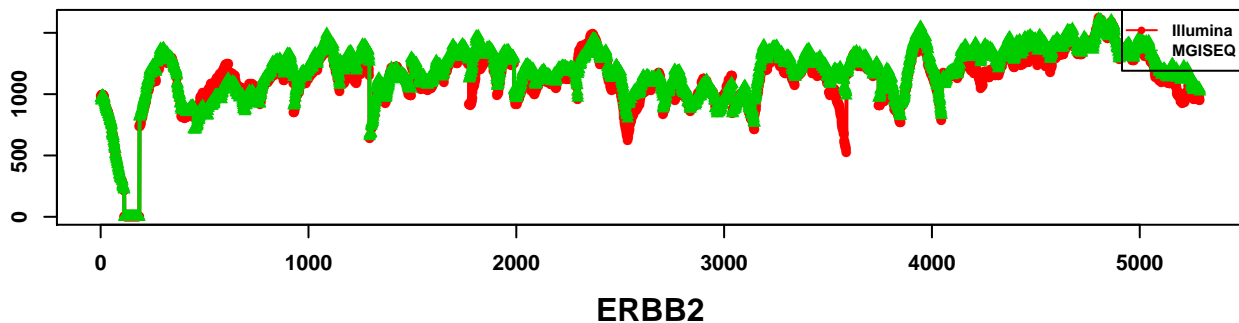

Sequencing Depth

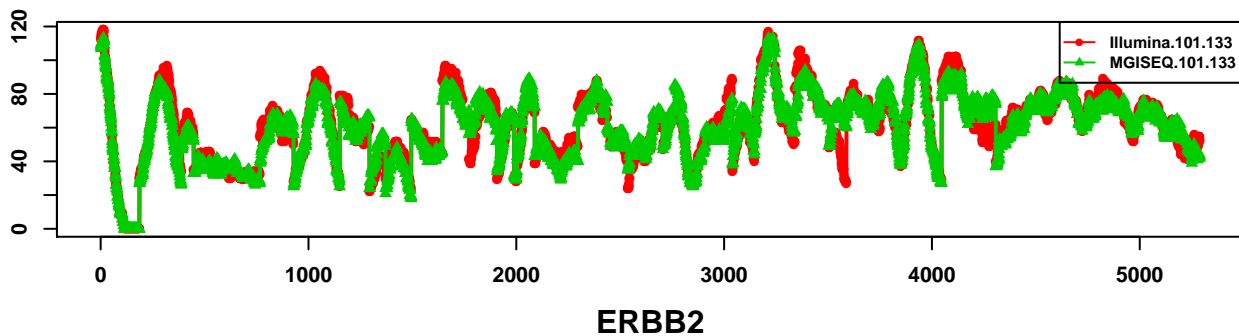

Sequencing Depth

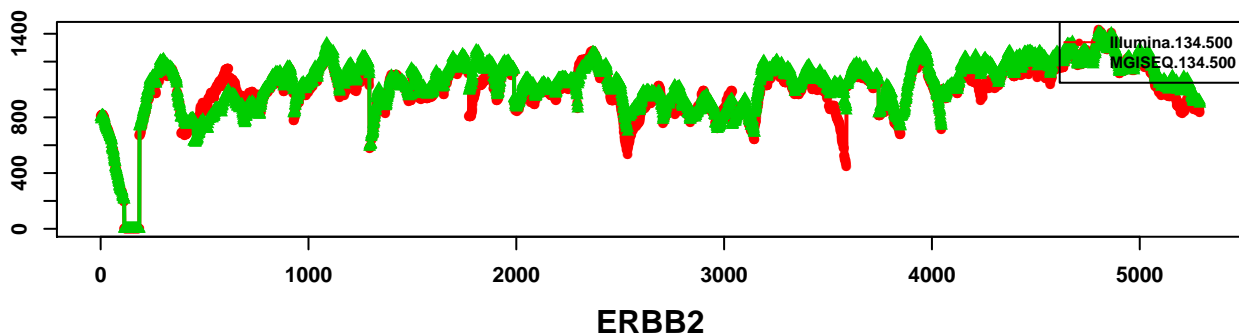

Supplement: Supplementary file 7 [file Presentation7.zip › ERBB2/19ZN13491P.pdf]

Sequencing Depth

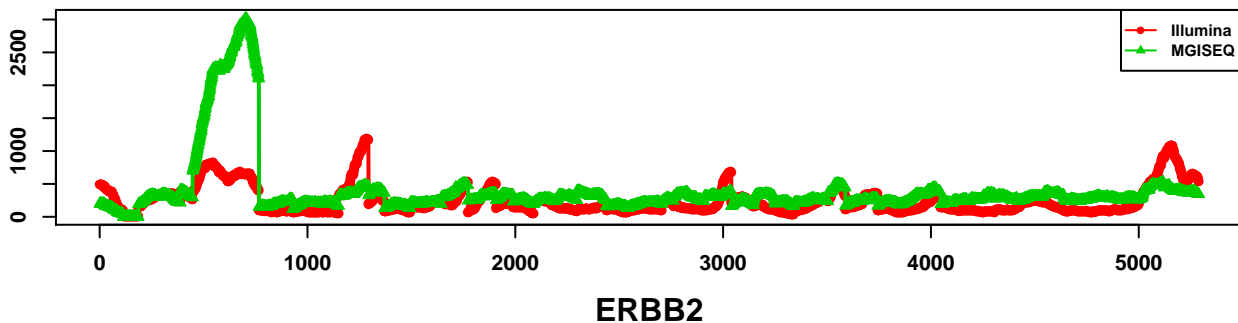

Sequencing Depth

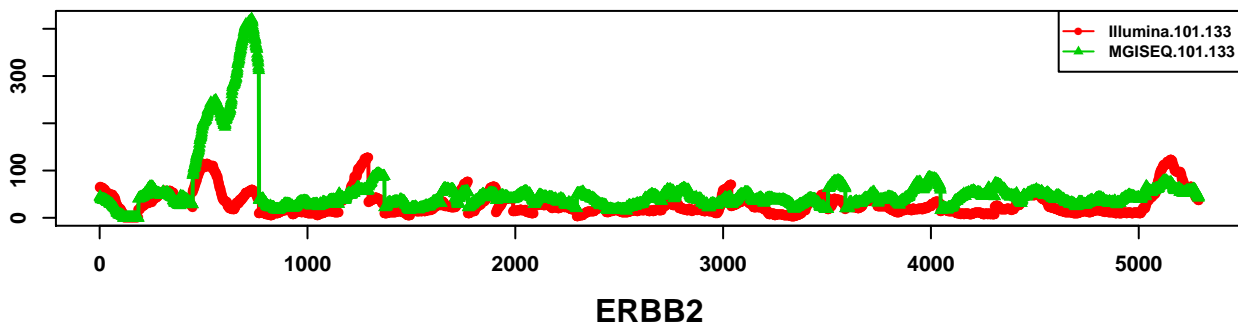

Sequencing Depth

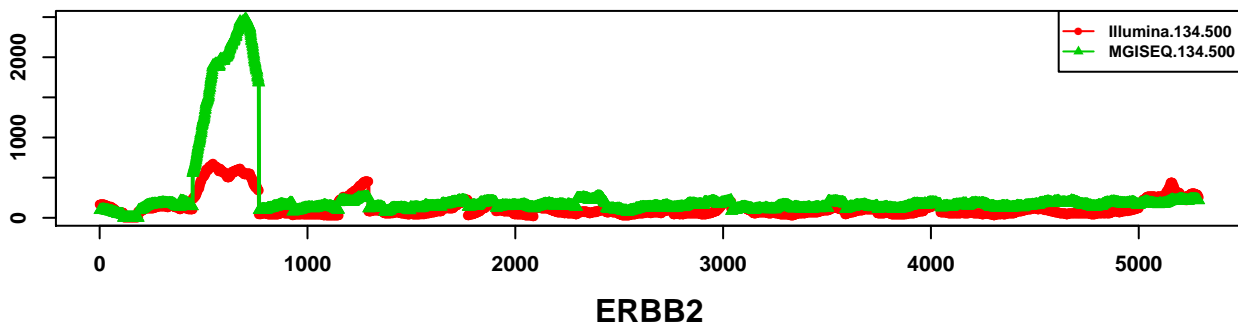

Supplement: Supplementary file 7 [file Presentation7.zip › ERBB2/19FC40427F.pdf]

Sequencing Depth

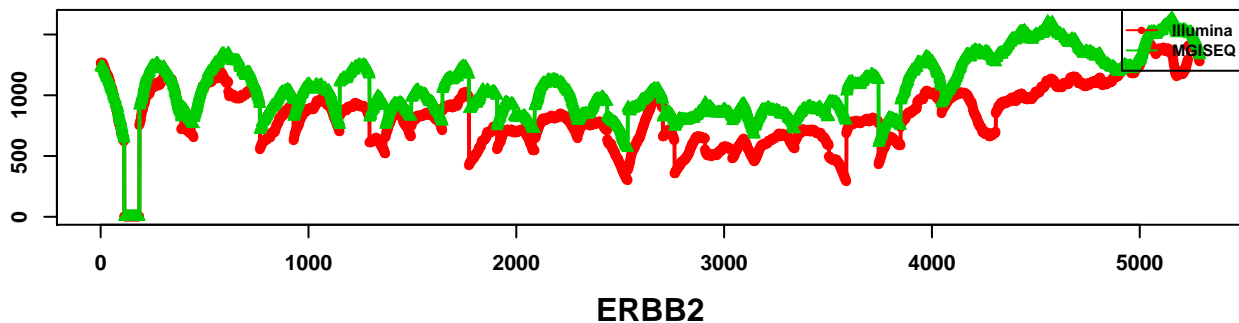

Sequencing Depth

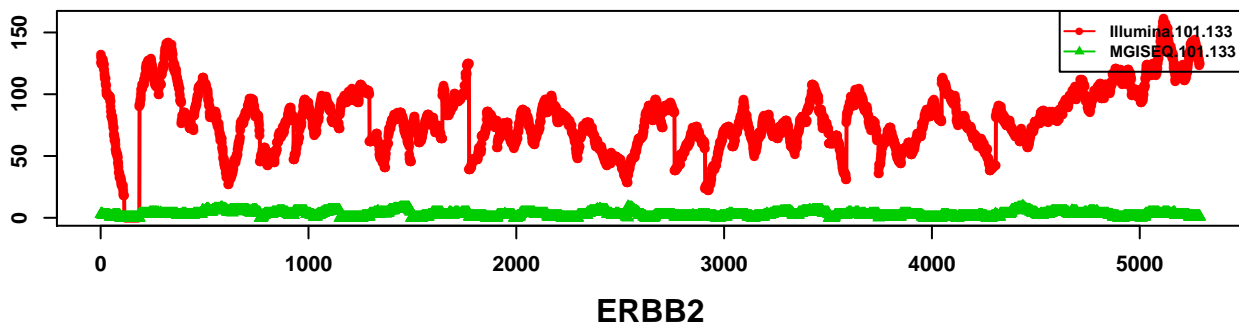

Sequencing Depth

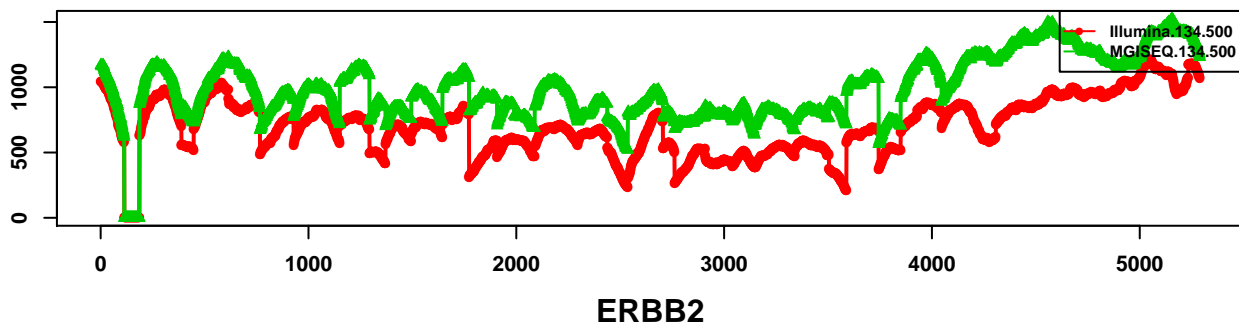

Supplement: Supplementary file 7 [file Presentation7.zip › ERBB2/19HE21842F.pdf]

Sequencing Depth

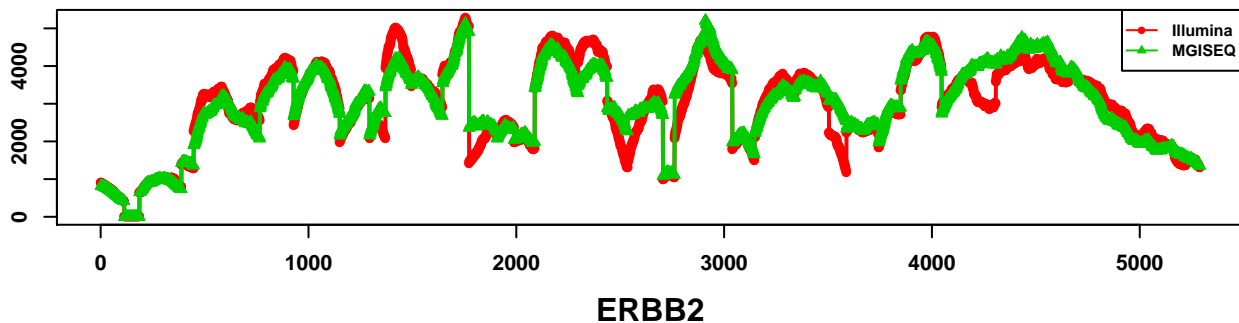

Sequencing Depth

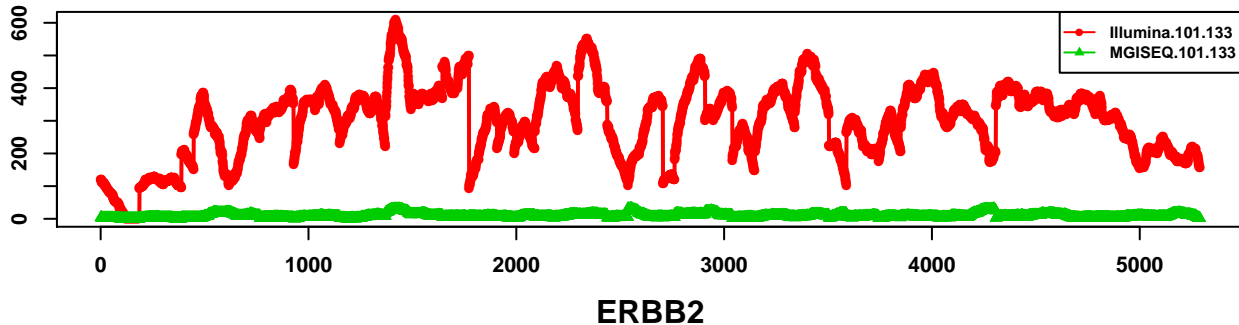

Sequencing Depth

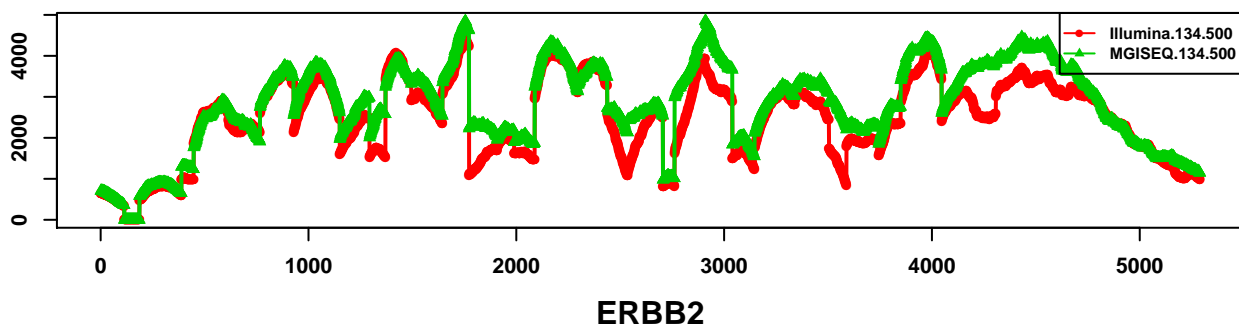

Supplement: Supplementary file 7 [file Presentation7.zip › ERBB2/19N01656F.pdf]

Sequencing Depth

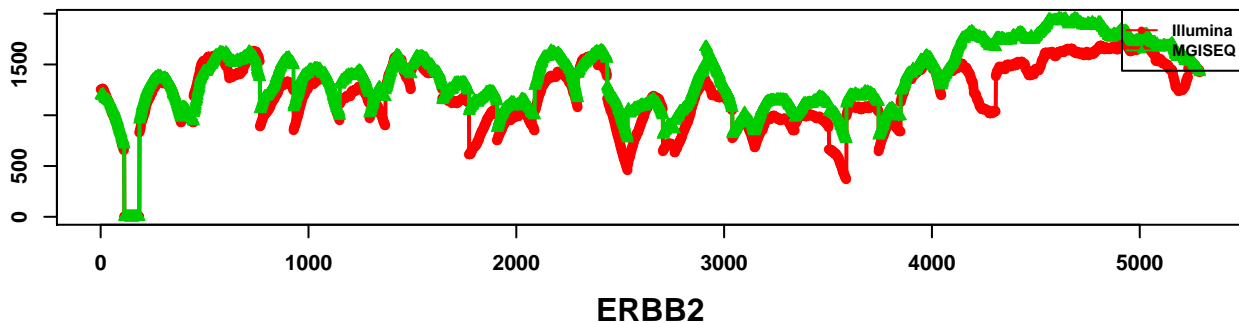

Sequencing Depth

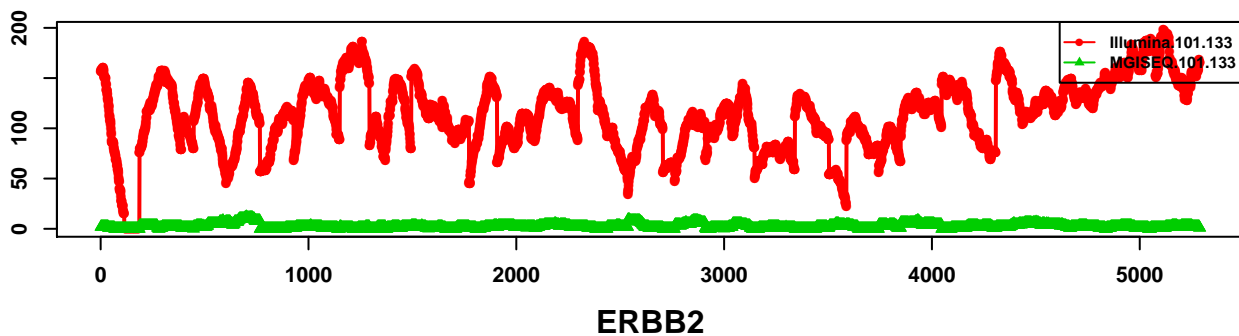

Sequencing Depth

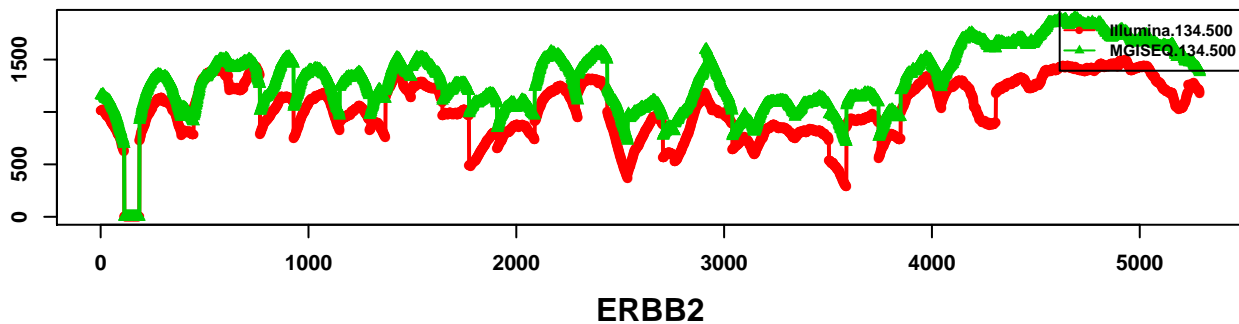

Supplement: Supplementary file 7 [file Presentation7.zip › ERBB2/19HE22110F.pdf]

Sequencing Depth

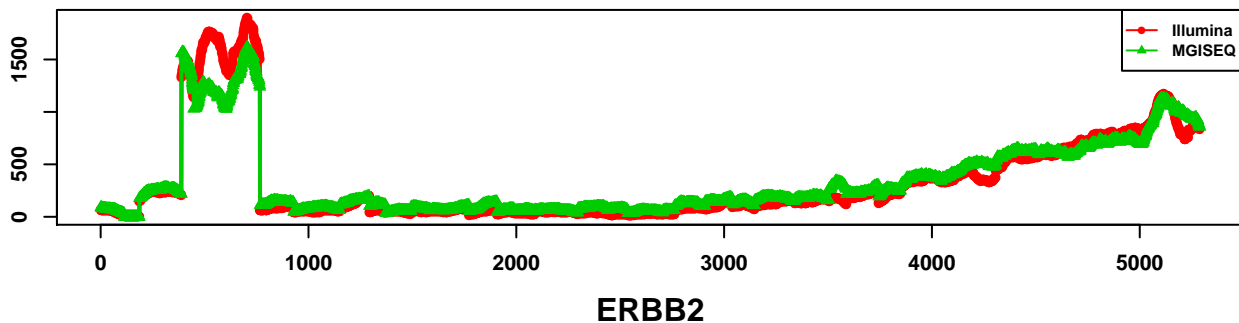

Sequencing Depth

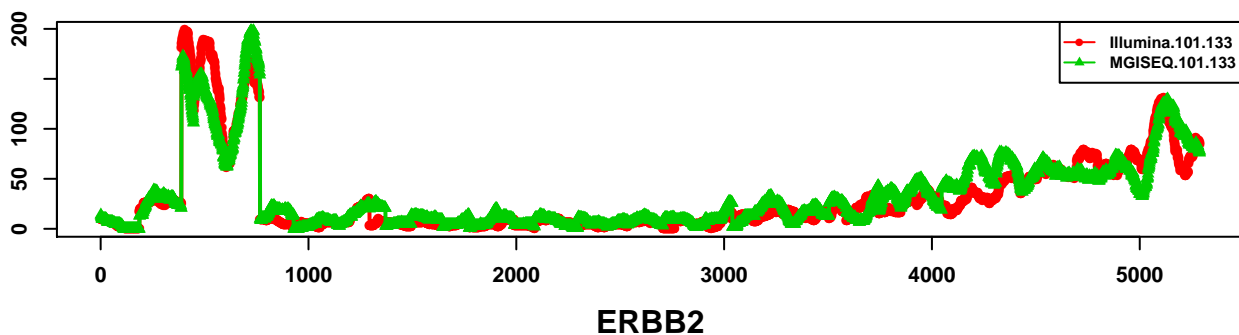

Sequencing Depth

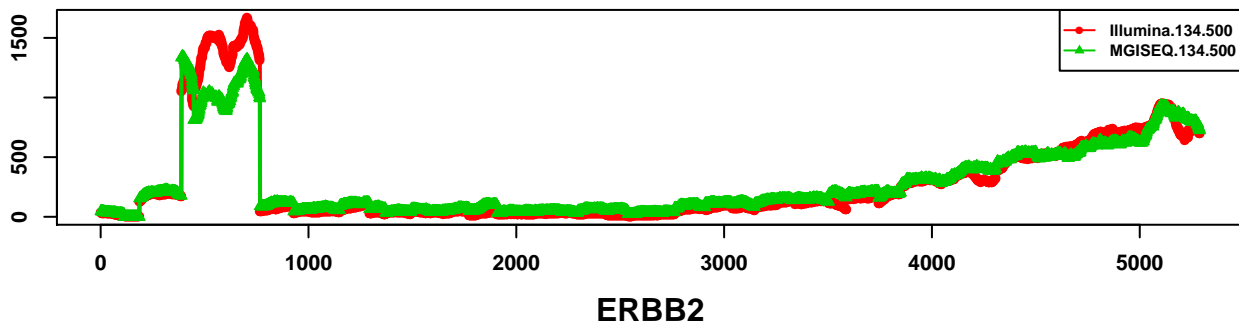

Supplement: Supplementary file 7 [file Presentation7.zip › ERBB2/19ZN13486T.pdf]

Sequencing Depth

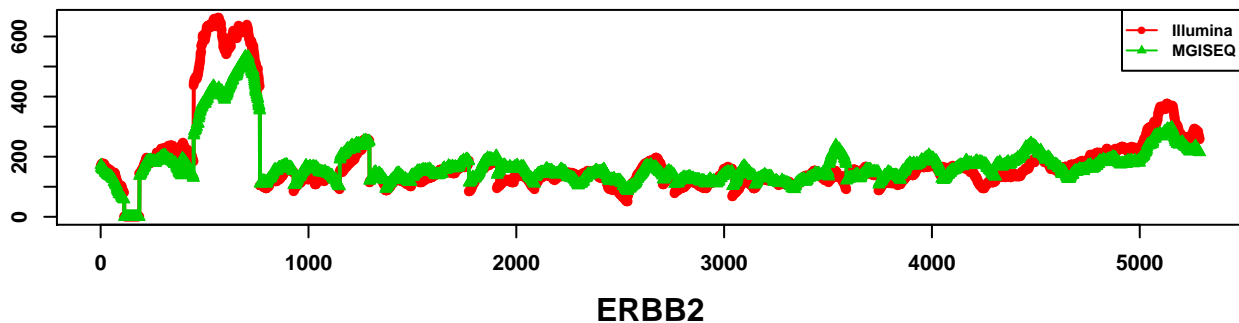

Sequencing Depth

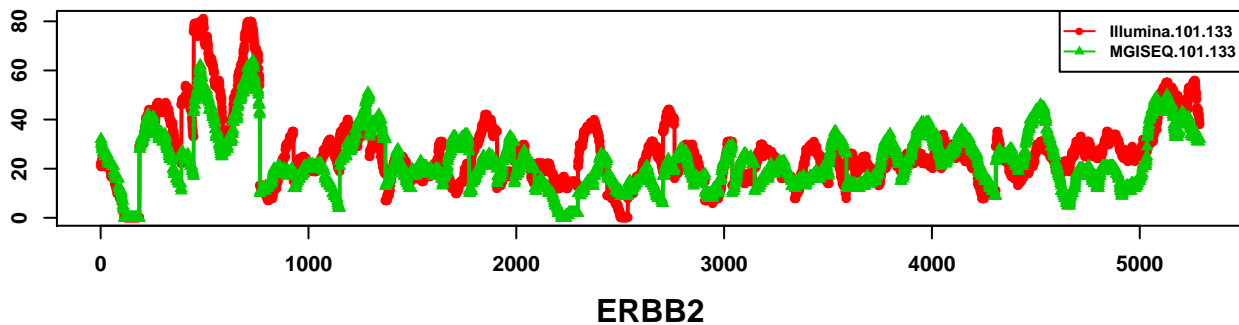

Sequencing Depth

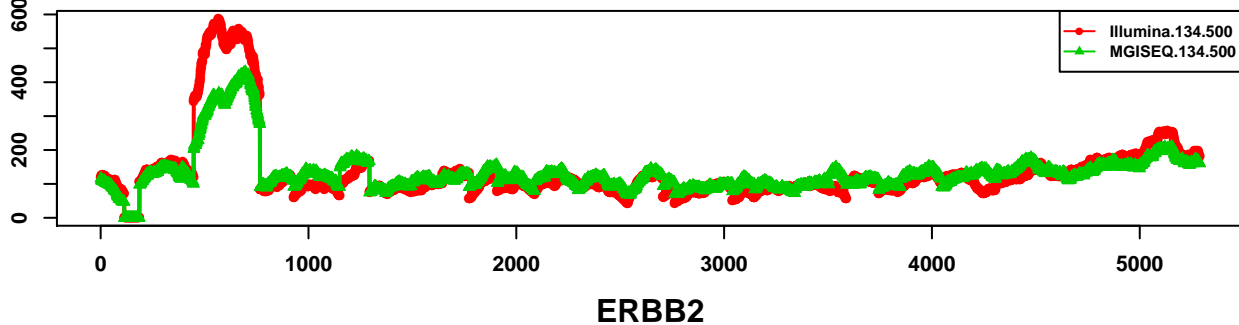

Supplement: Supplementary file 7 [file Presentation7.zip › ERBB2/19JS48247F.pdf]

Sequencing Depth

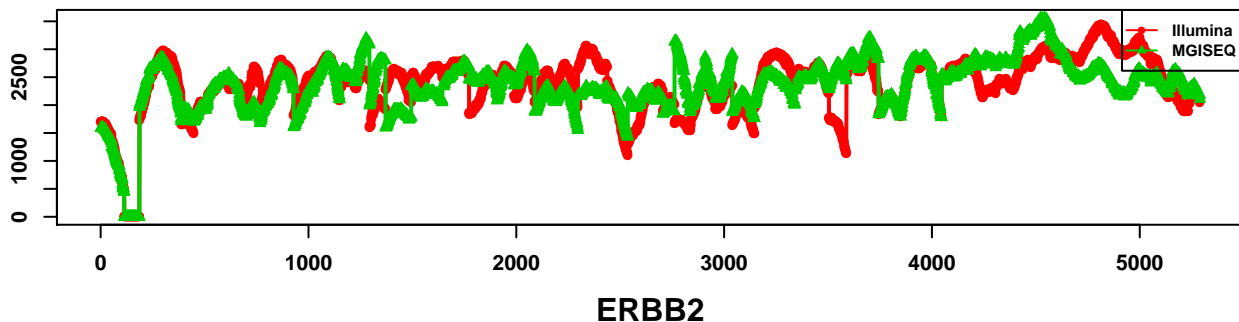

Sequencing Depth

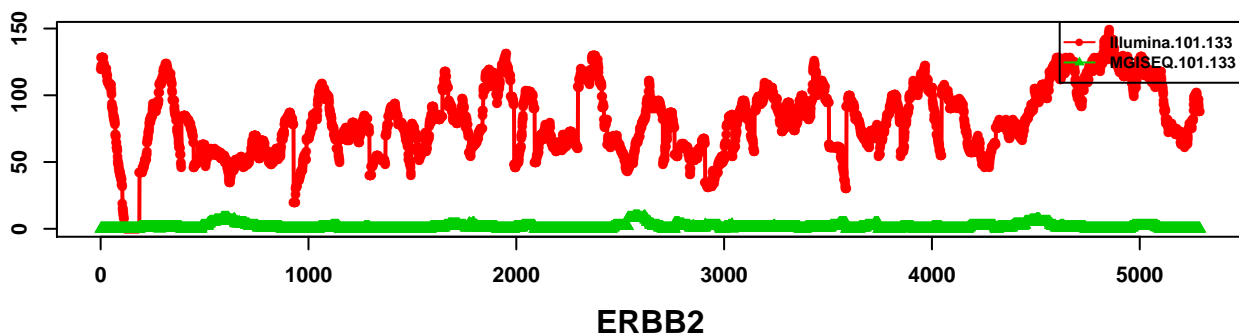

Sequencing Depth

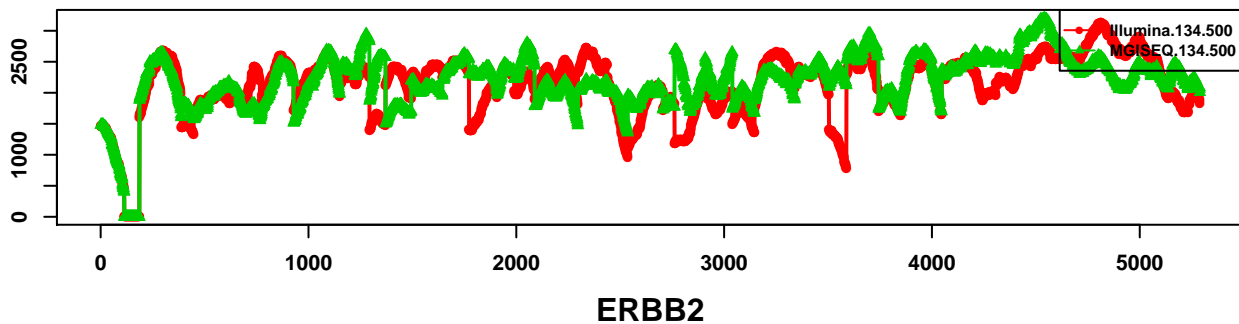

Supplement: Supplementary file 7 [file Presentation7.zip › ERBB2/19ZN12296P.pdf]

Sequencing Depth

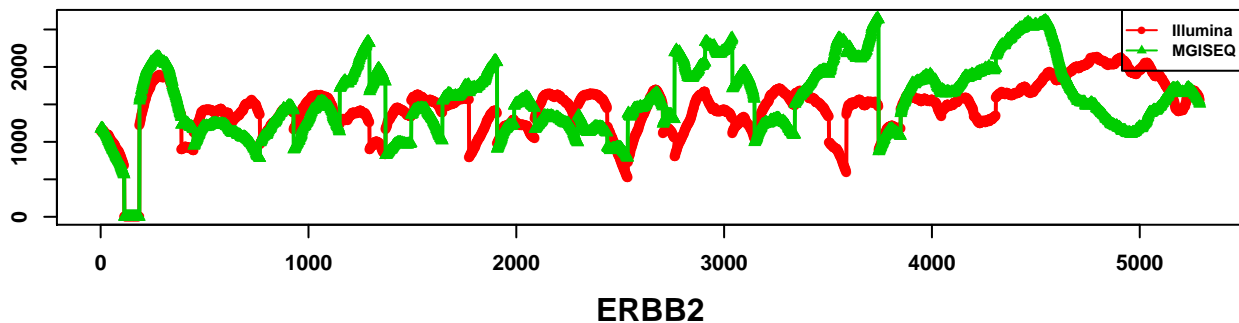

Sequencing Depth

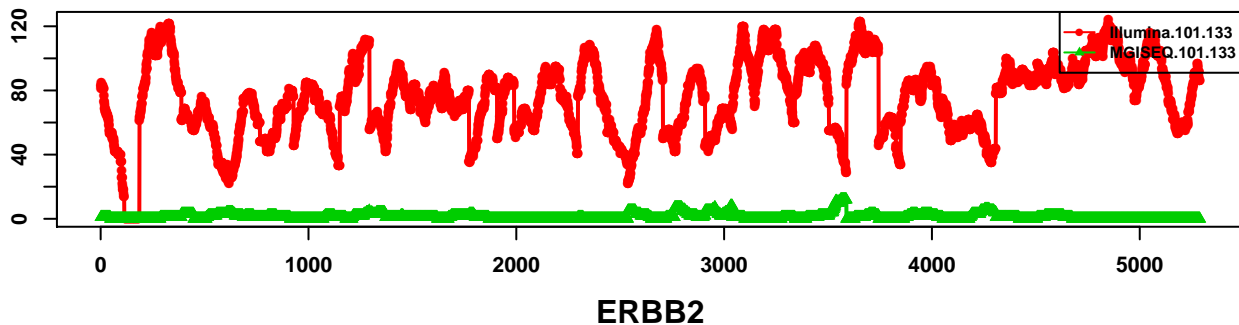

Sequencing Depth

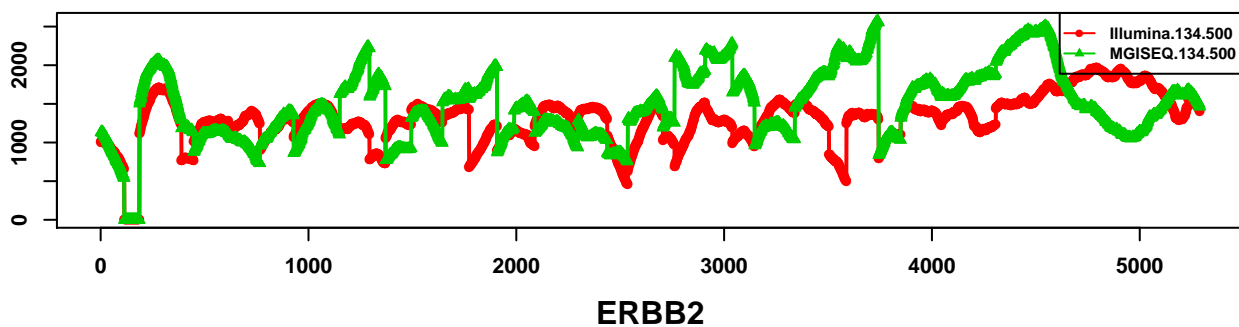

Supplement: Supplementary file 7 [file Presentation7.zip › ERBB2/19CF15537B.pdf]

Sequencing Depth

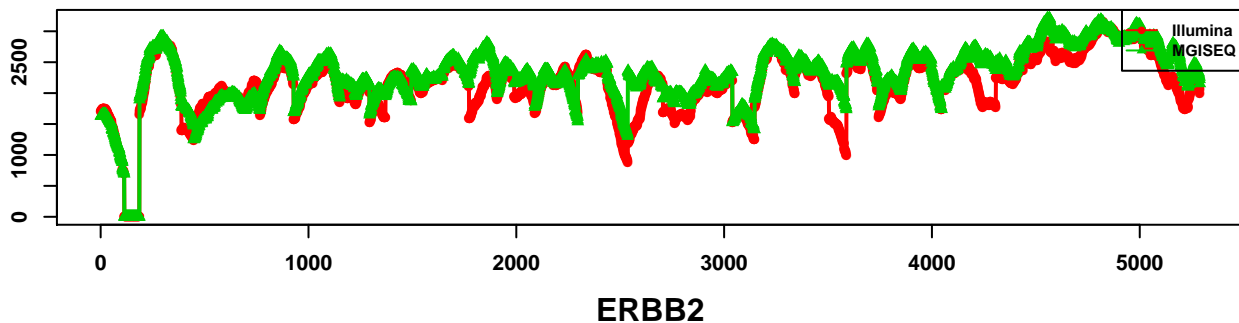

Sequencing Depth

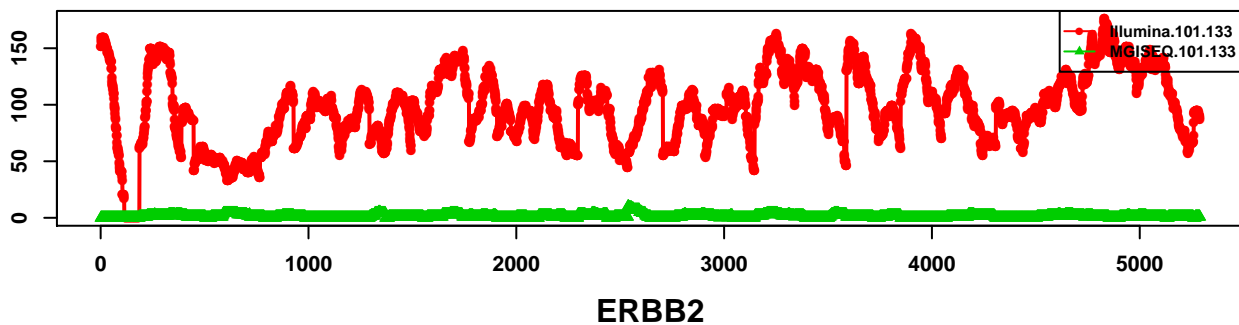

Sequencing Depth

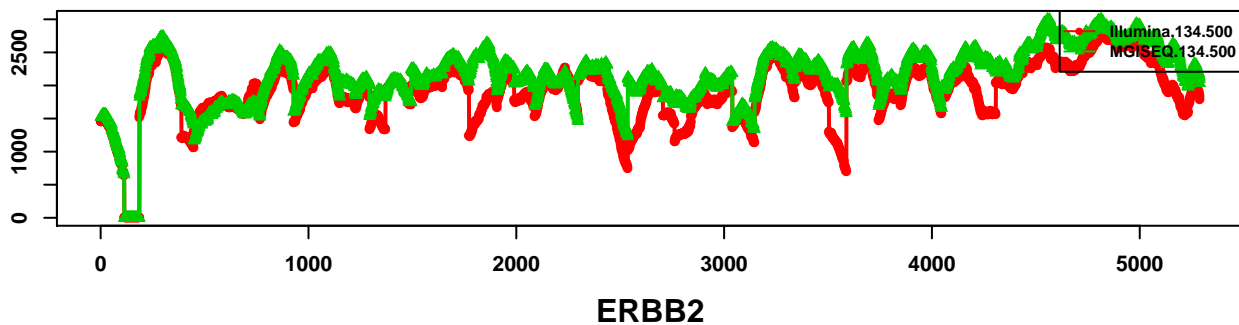

Supplement: Supplementary file 7 [file Presentation7.zip › ERBB2/19ZN13106P.pdf]

Sequencing Depth

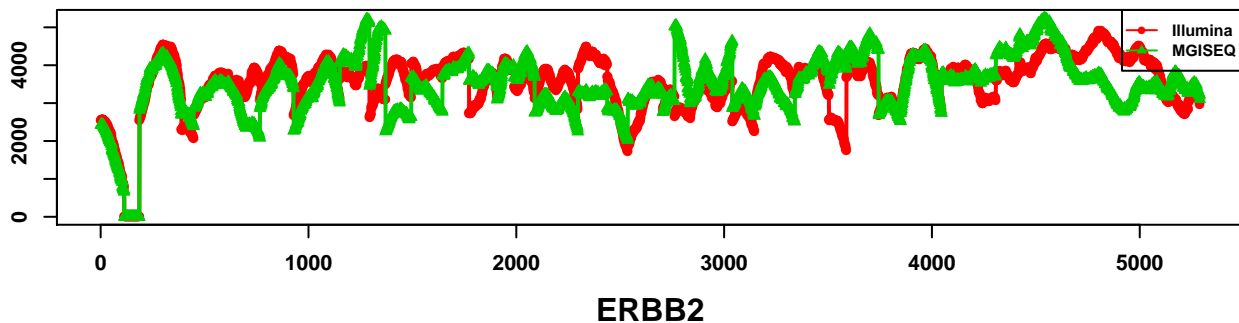

Sequencing Depth

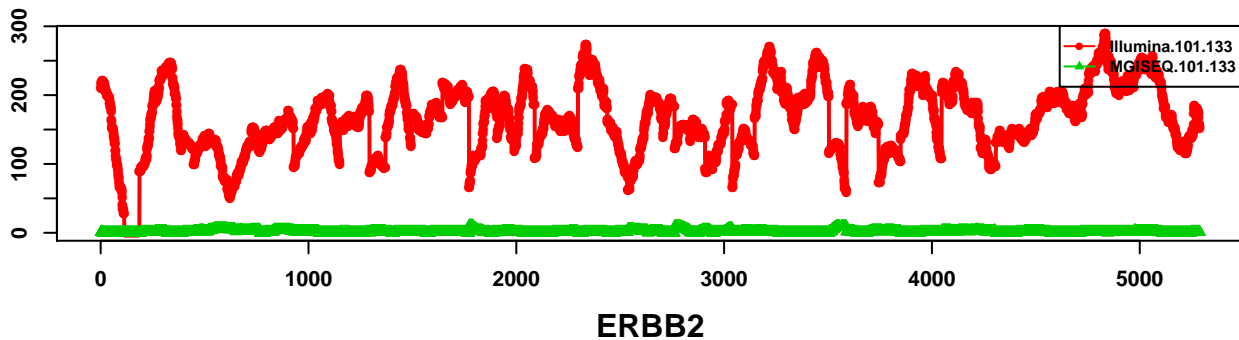

Sequencing Depth

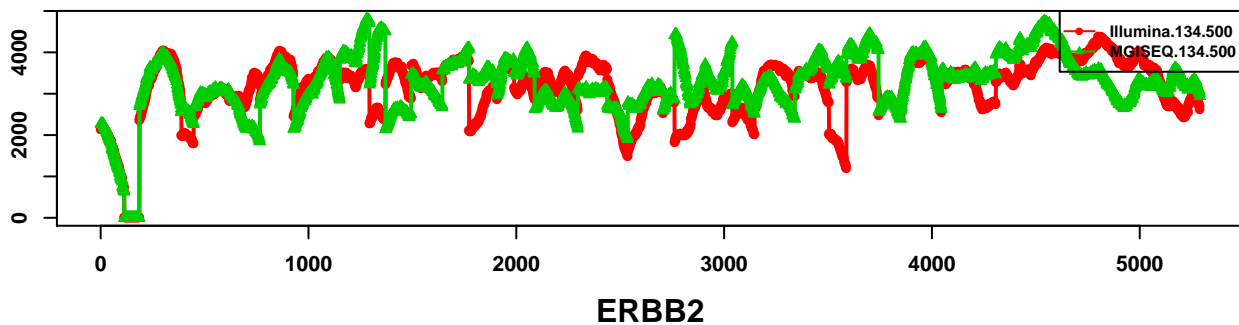

Supplement: Supplementary file 7 [file Presentation7.zip › ERBB2/19JS48181P.pdf]

Sequencing Depth

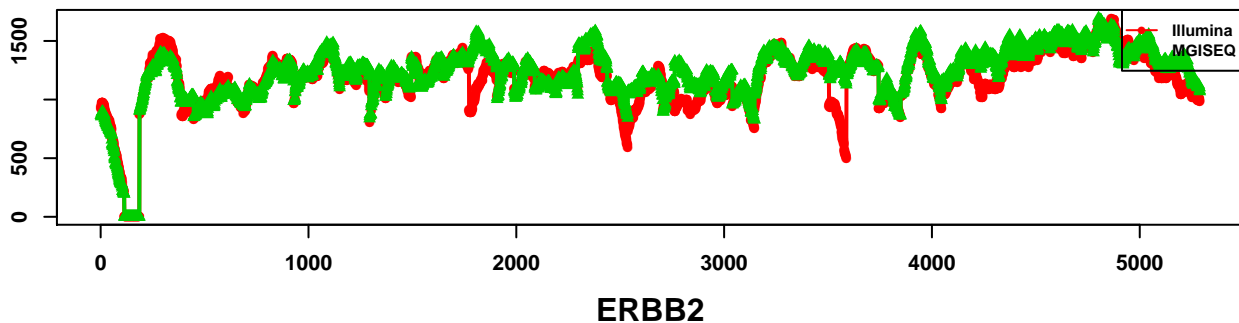

Sequencing Depth

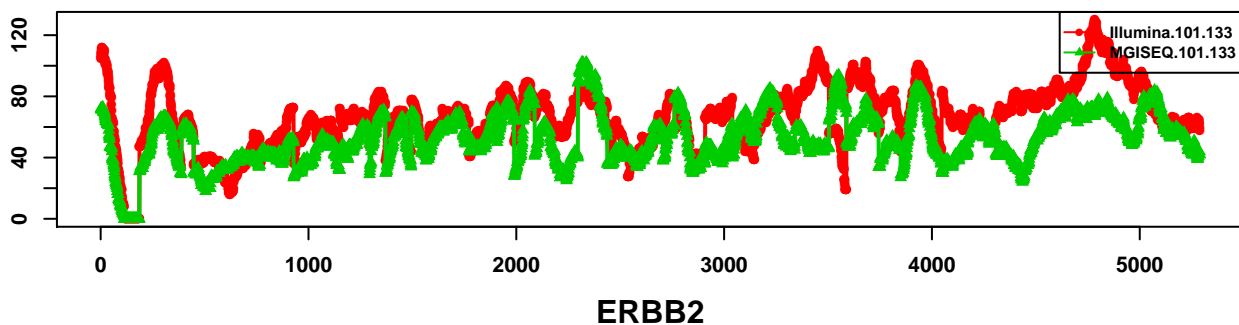

Sequencing Depth

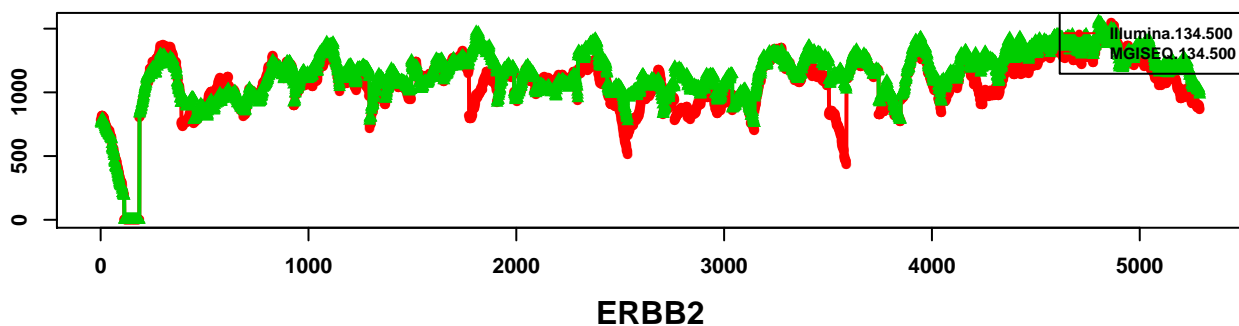

Supplement: Supplementary file 7 [file Presentation7.zip › ERBB2/19N02353P.pdf]

Sequencing Depth

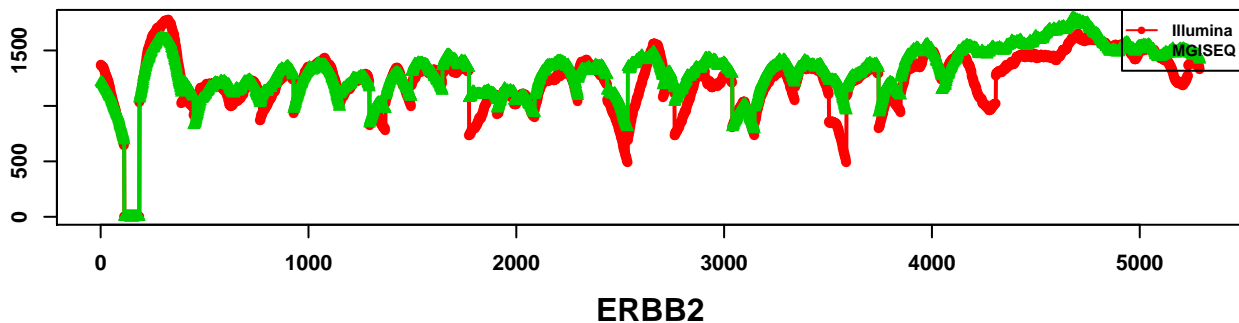

Sequencing Depth

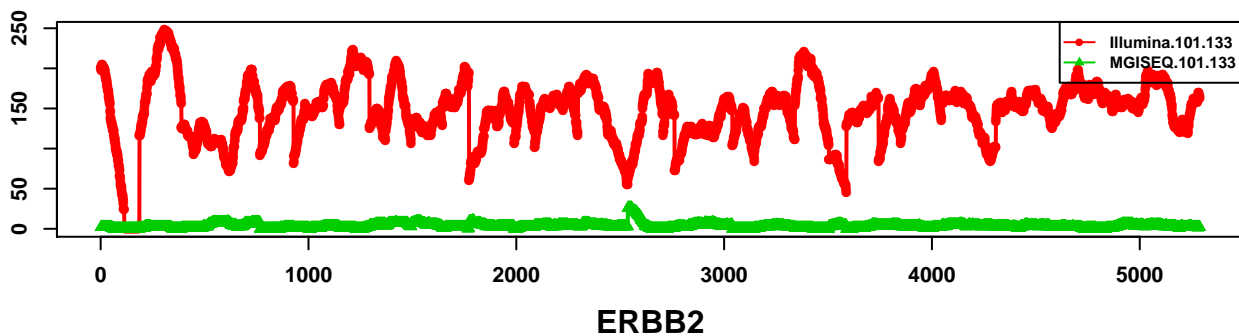

Sequencing Depth

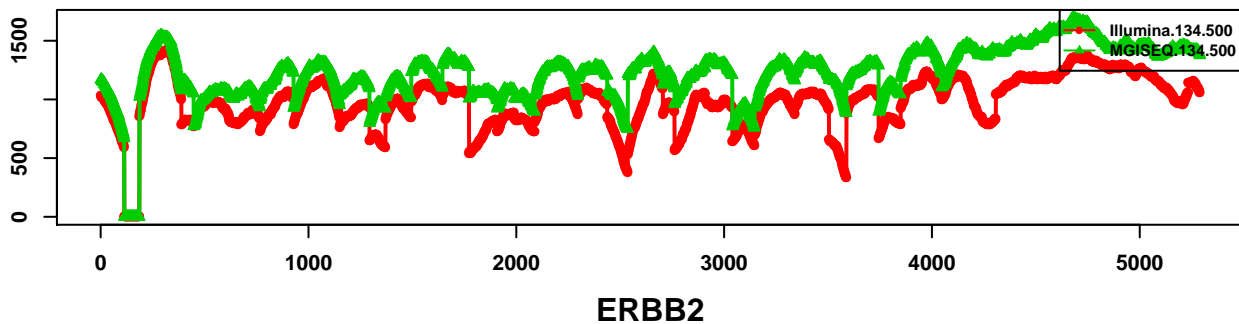

Supplement: Supplementary file 7 [file Presentation7.zip › ERBB2/19FC40260F.pdf]

Sequencing Depth

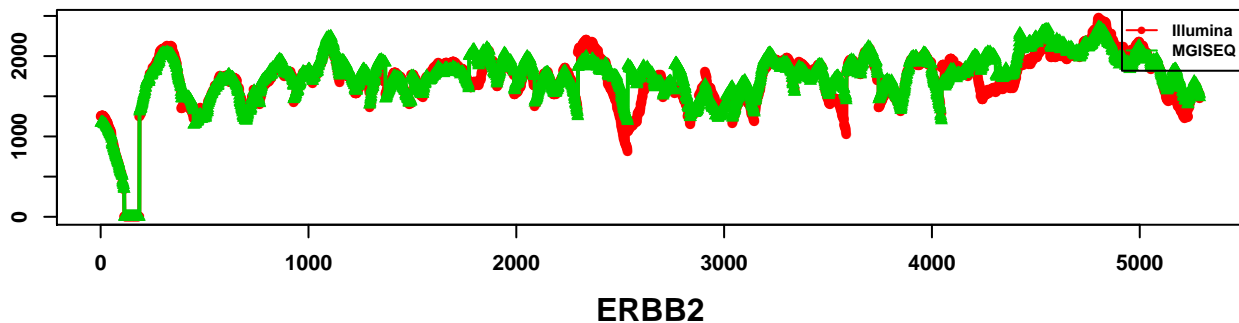

Sequencing Depth

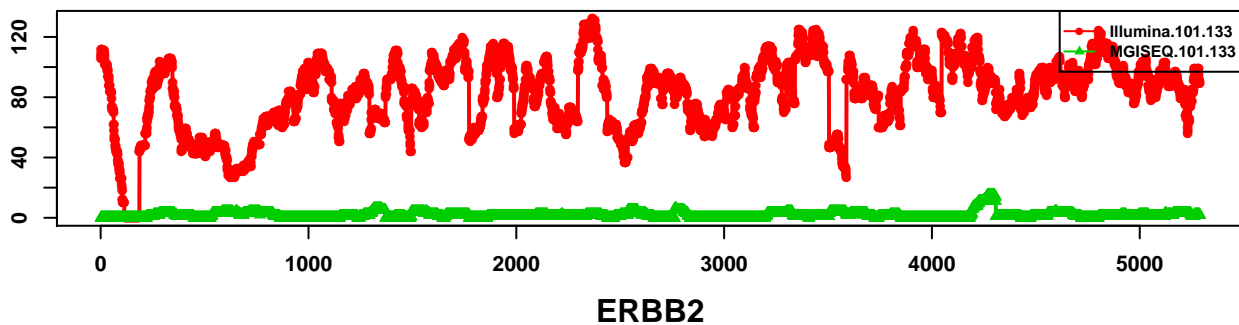

Sequencing Depth

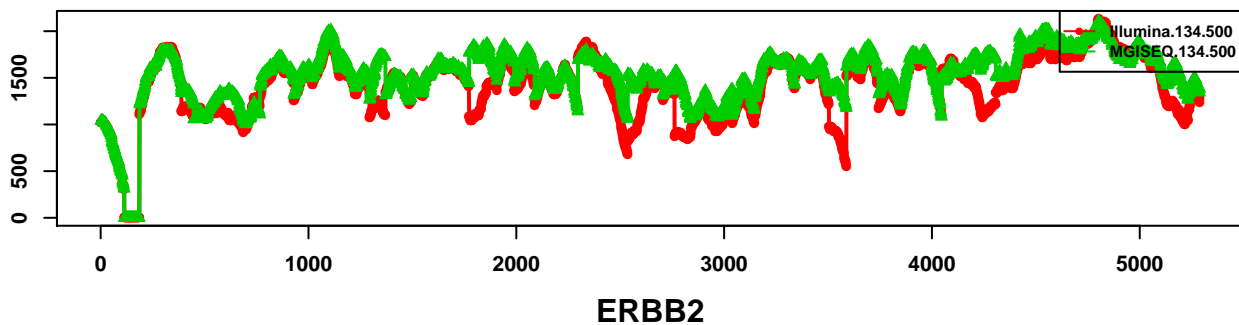

Supplement: Supplementary file 7 [file Presentation7.zip › ERBB2/19N01456-IIIP.pdf]

Sequencing Depth

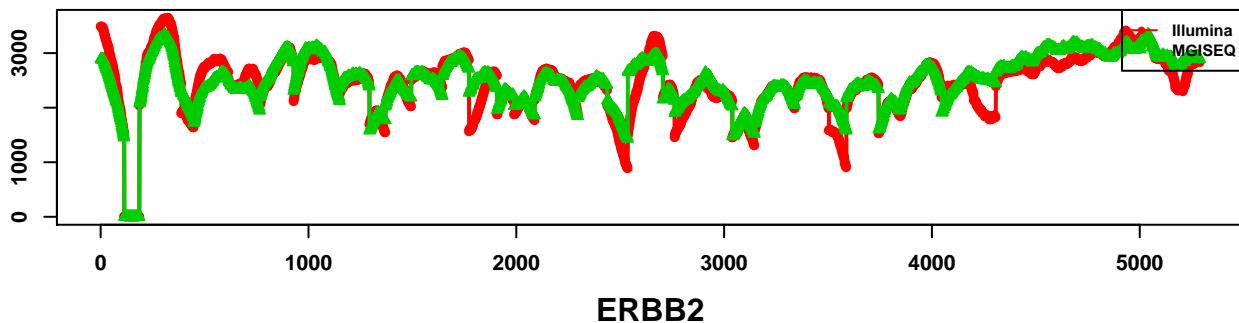

Sequencing Depth

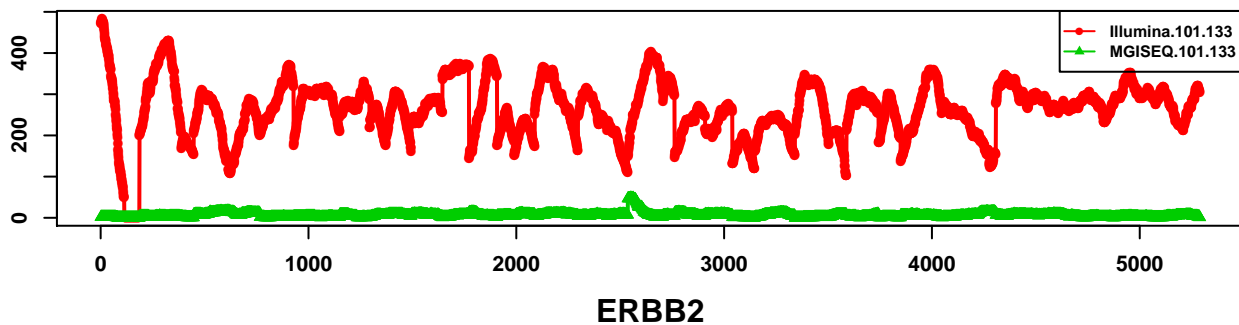

Sequencing Depth

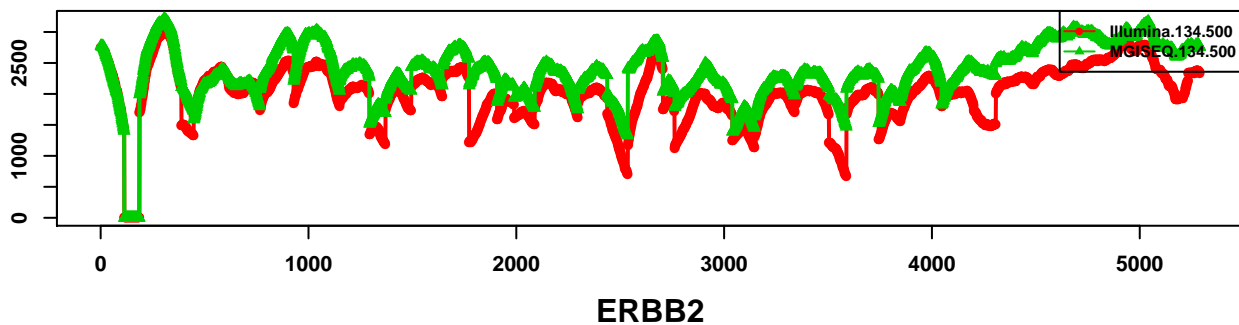

Supplement: Supplementary file 7 [file Presentation7.zip › ERBB2/19FC40261F.pdf]

Sequencing Depth

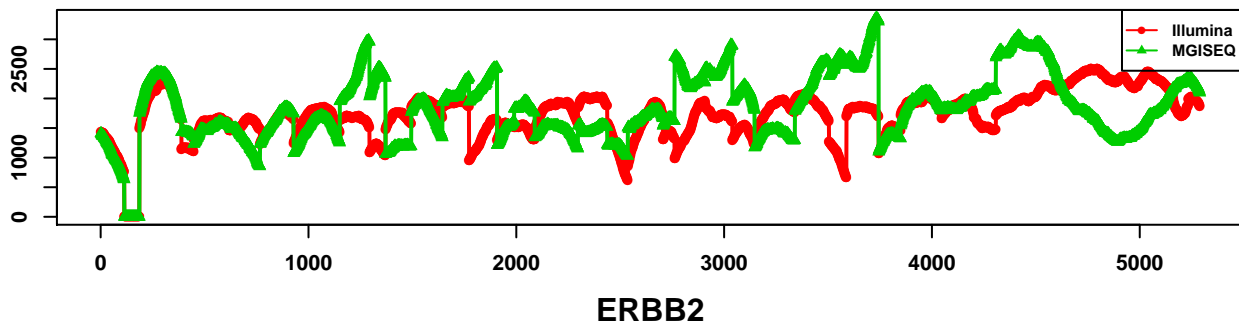

Sequencing Depth

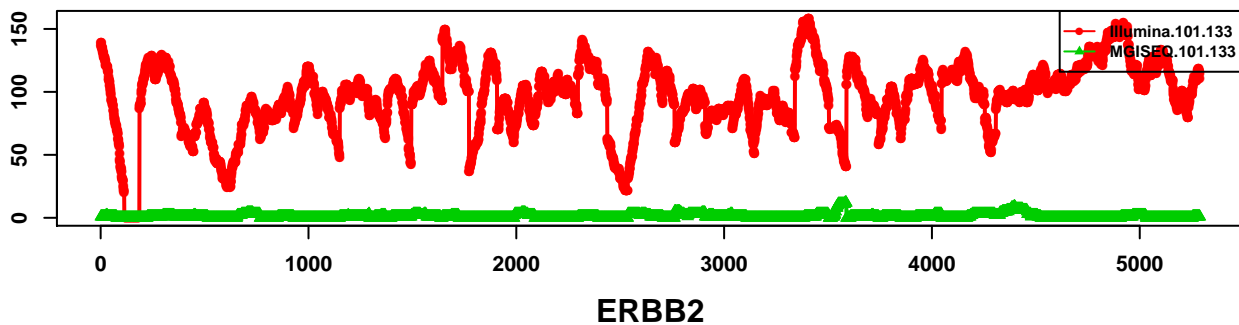

Sequencing Depth

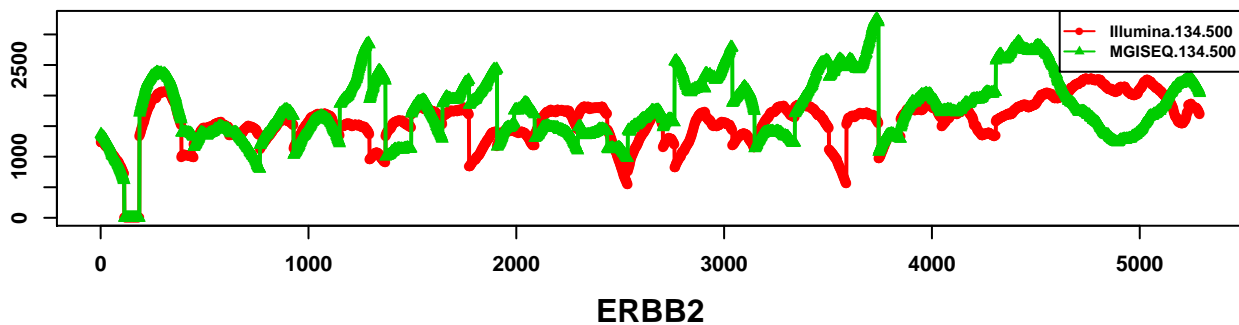

Supplement: Supplementary file 7 [file Presentation7.zip › ERBB2/19ZN13096B.pdf]

Sequencing Depth

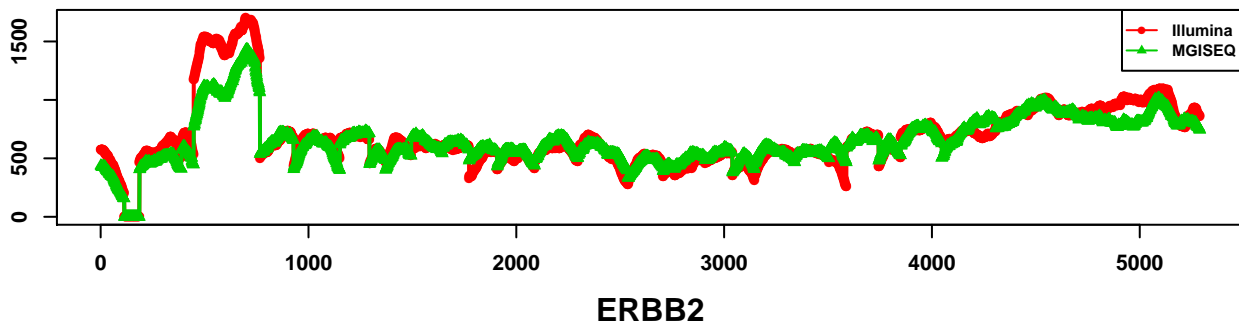

Sequencing Depth

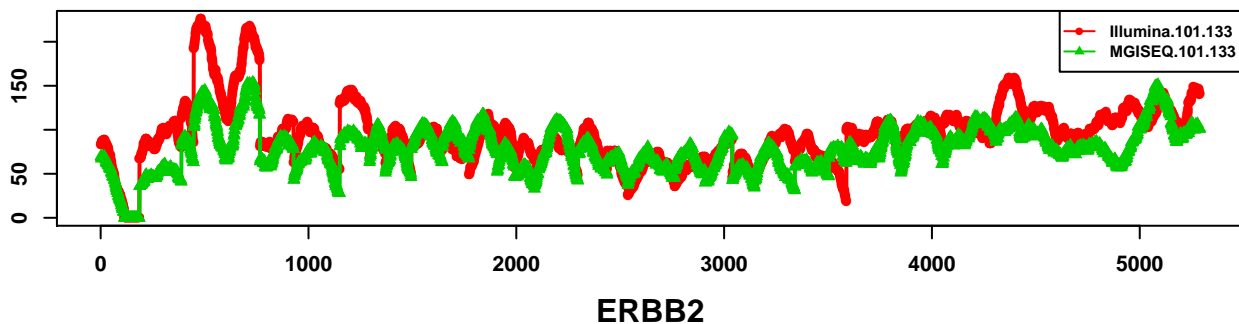

Sequencing Depth

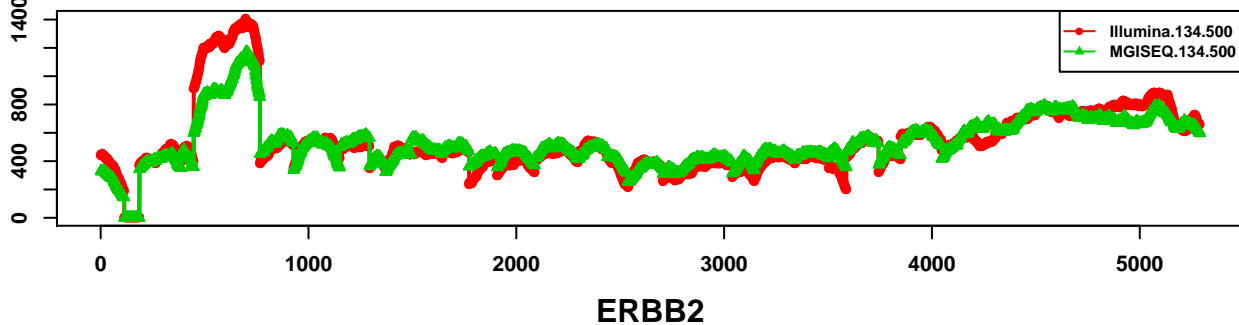

Supplement: Supplementary file 7 [file Presentation7.zip › ERBB2/19N02329F.pdf]

Sequencing Depth

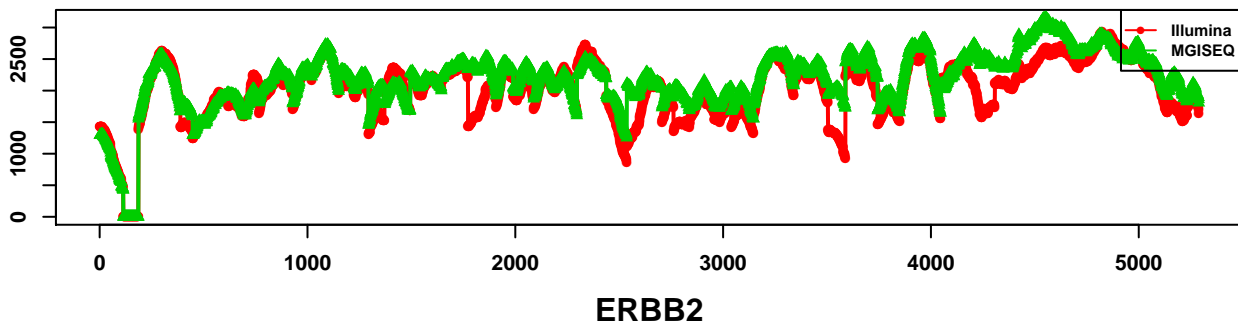

Sequencing Depth

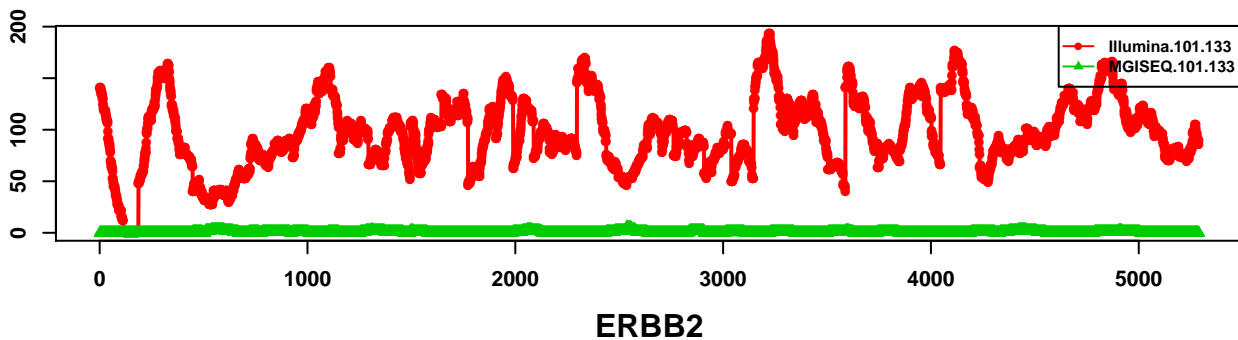

Sequencing Depth

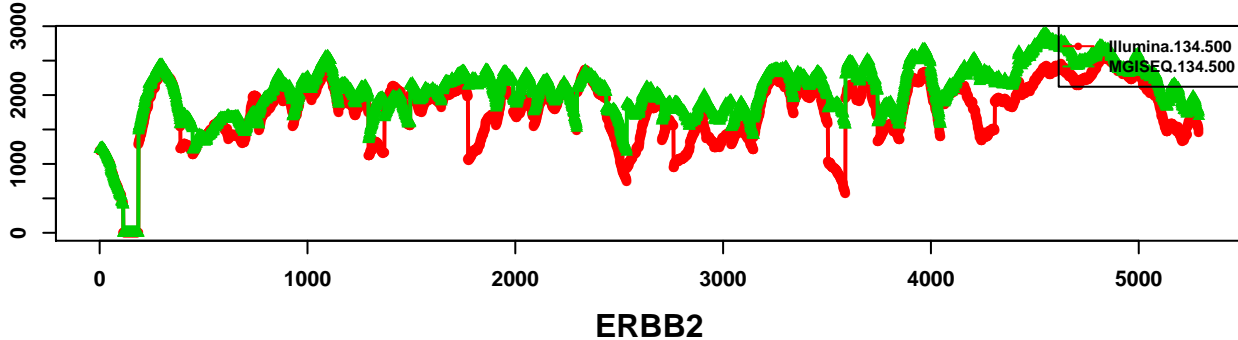

Supplement: Supplementary file 7 [file Presentation7.zip › ERBB2/19JS48180P.pdf]

Sequencing Depth

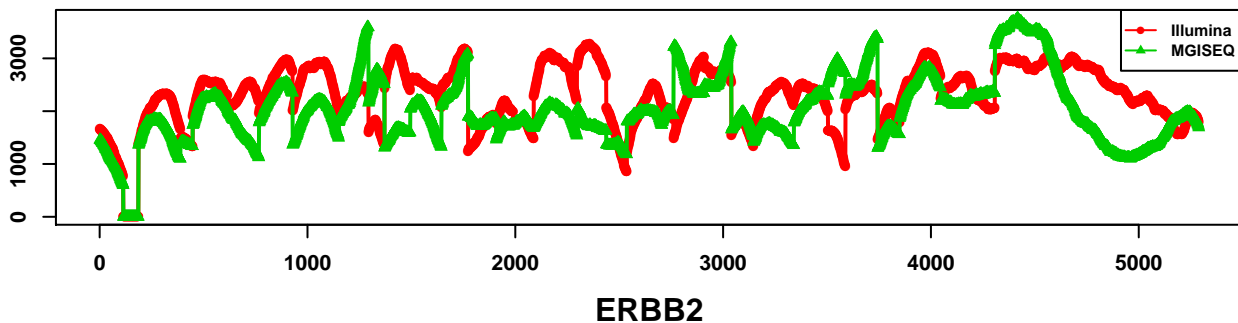

Sequencing Depth

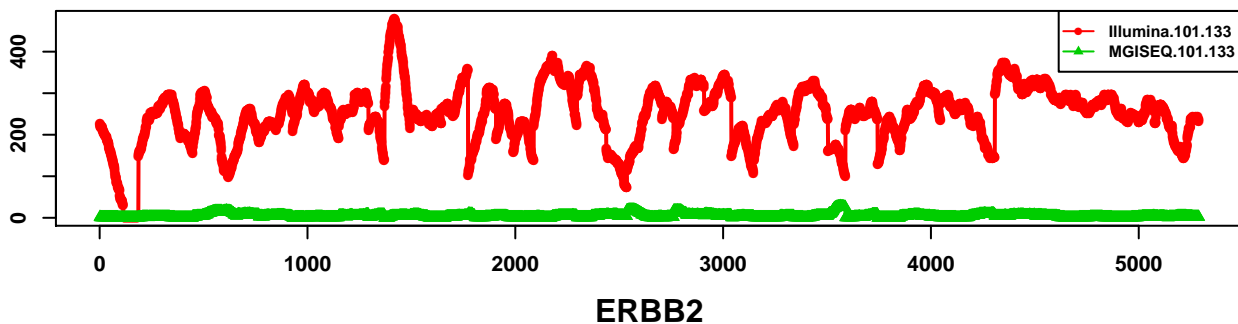

Sequencing Depth

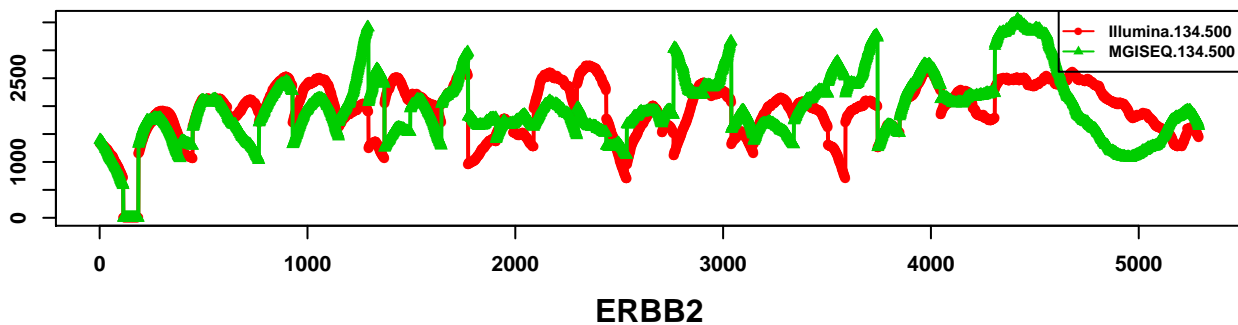

Supplement: Supplementary file 7 [file Presentation7.zip › ERBB2/19HE22185F.pdf]

Sequencing Depth

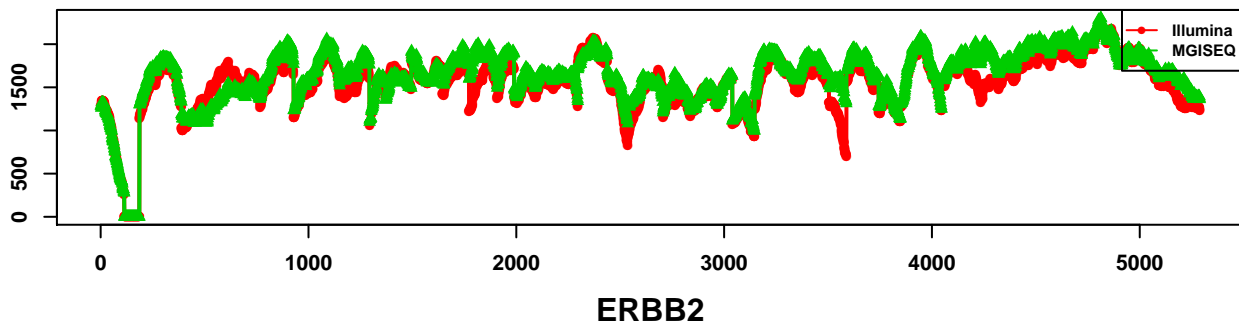

Sequencing Depth

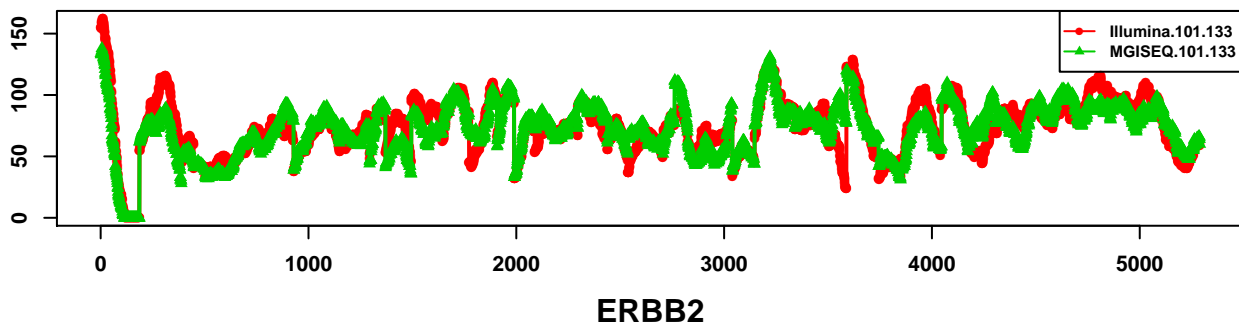

Sequencing Depth

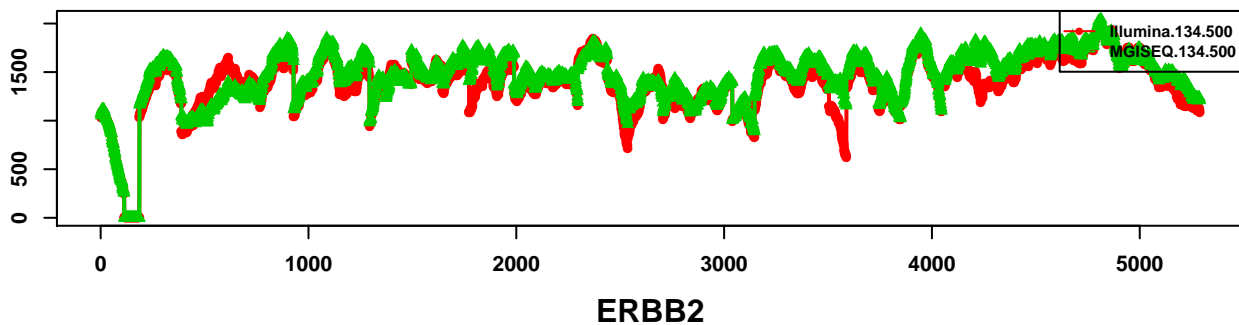

Supplement: Supplementary file 7 [file Presentation7.zip › ERBB2/19JS48246P.pdf]

Sequencing Depth

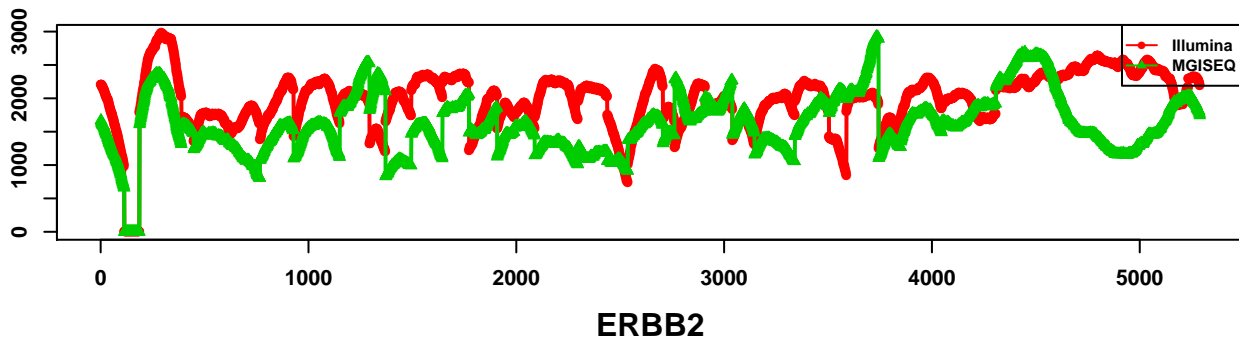

Sequencing Depth

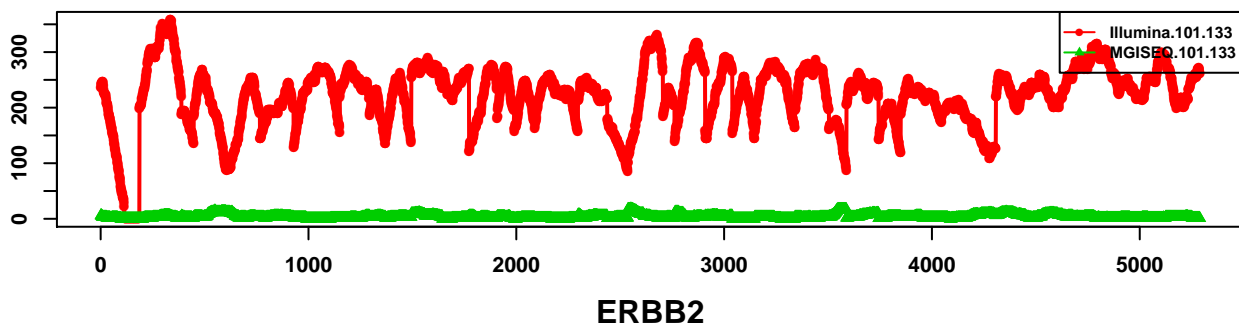

Sequencing Depth

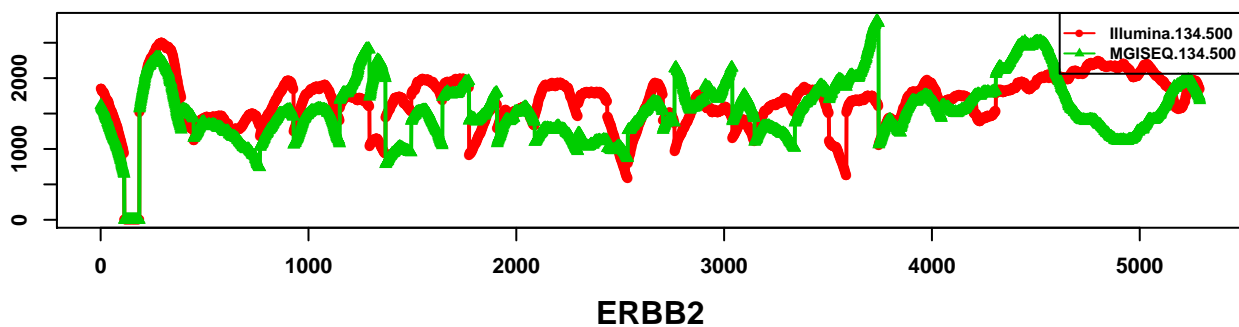

Supplement: Supplementary file 7 [file Presentation7.zip › ERBB2/19ZN12297F.pdf]

Sequencing Depth

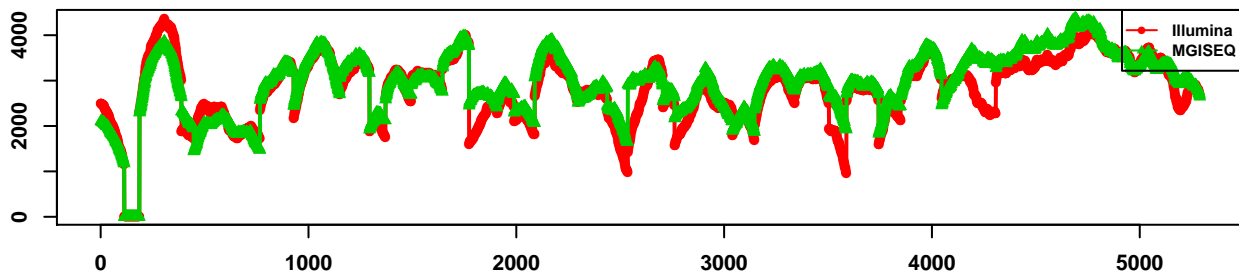

ERBB2

Sequencing Depth

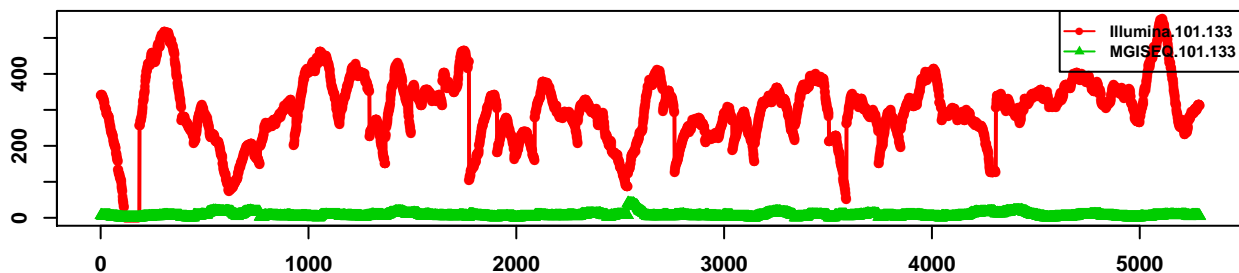

ERBB2

Sequencing Depth

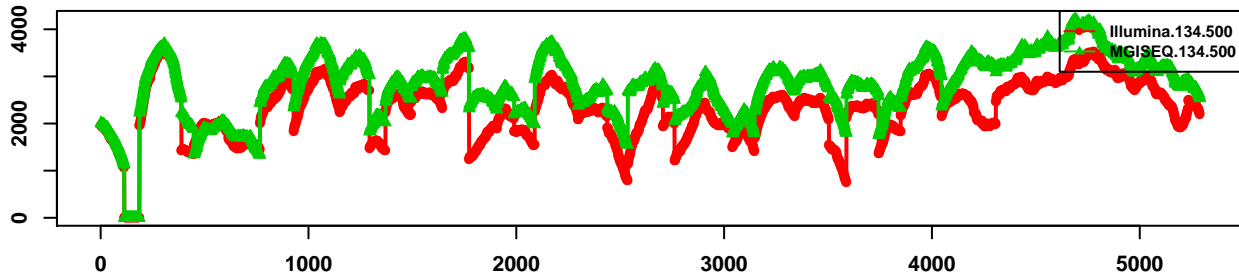

ERBB2

Supplement: Supplementary file 7 [file Presentation7.zip › ERBB2/19FC40253-IIF.pdf]

Sequencing Depth

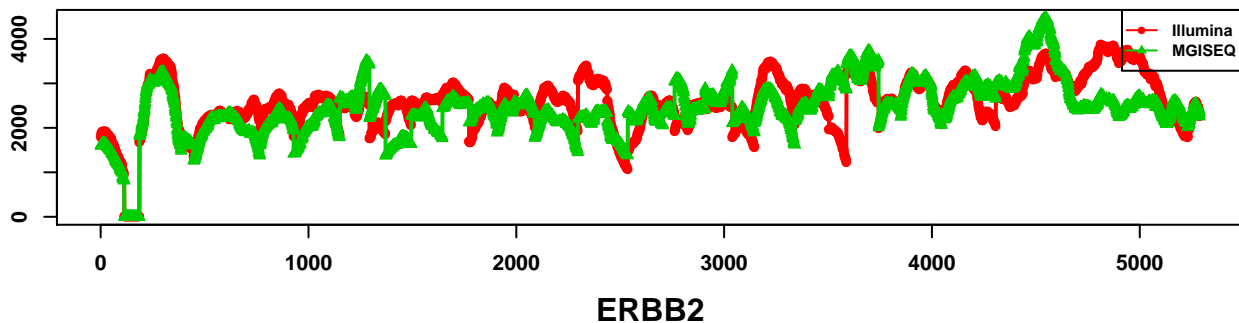

Sequencing Depth

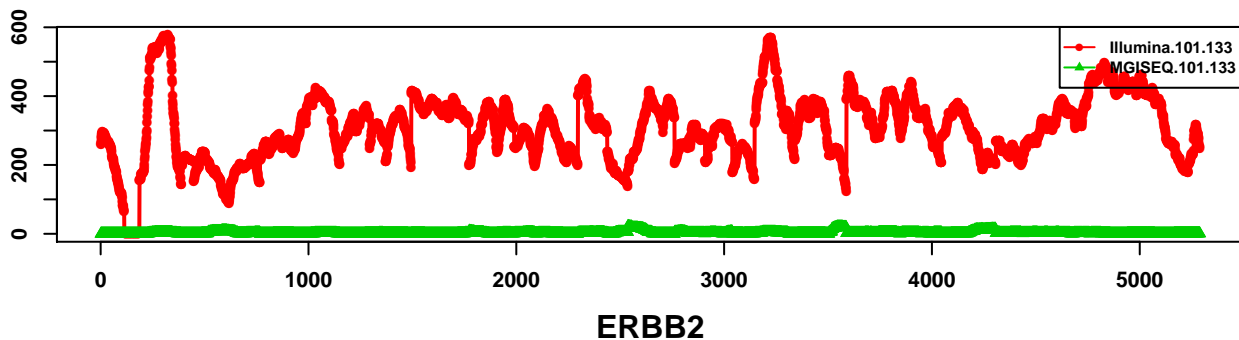

Sequencing Depth

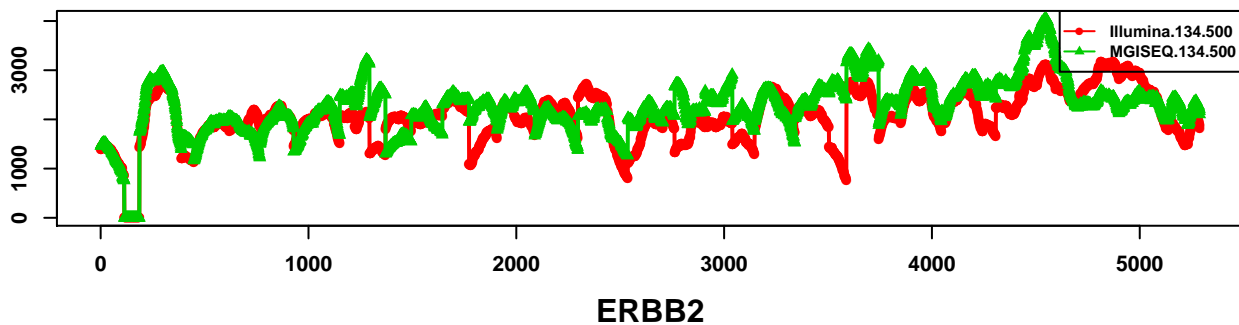

Supplement: Supplementary file 7 [file Presentation7.zip › ERBB2/19ZN13097P.pdf]

Sequencing Depth

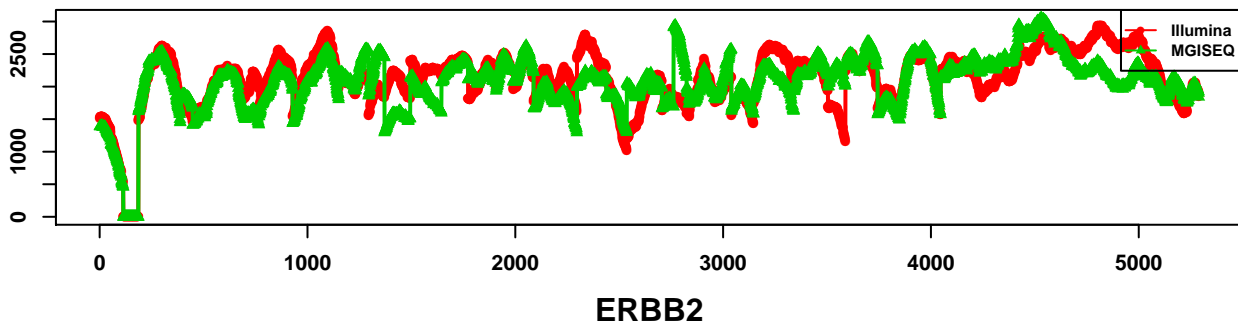

Sequencing Depth

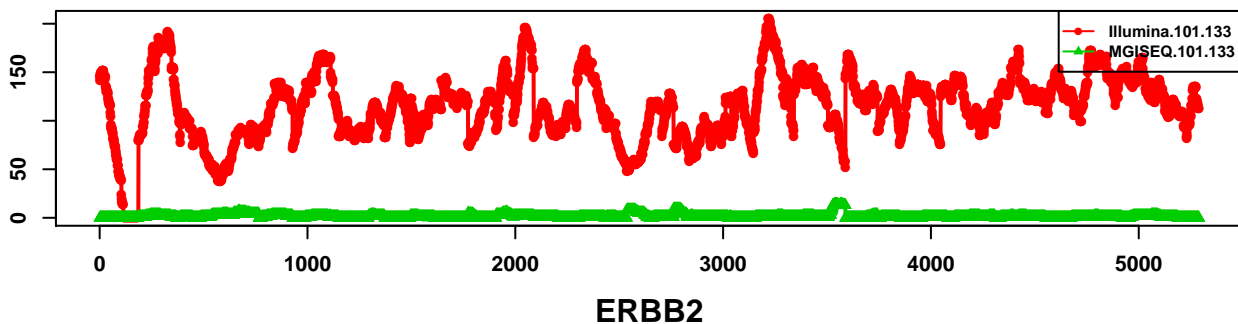

Sequencing Depth

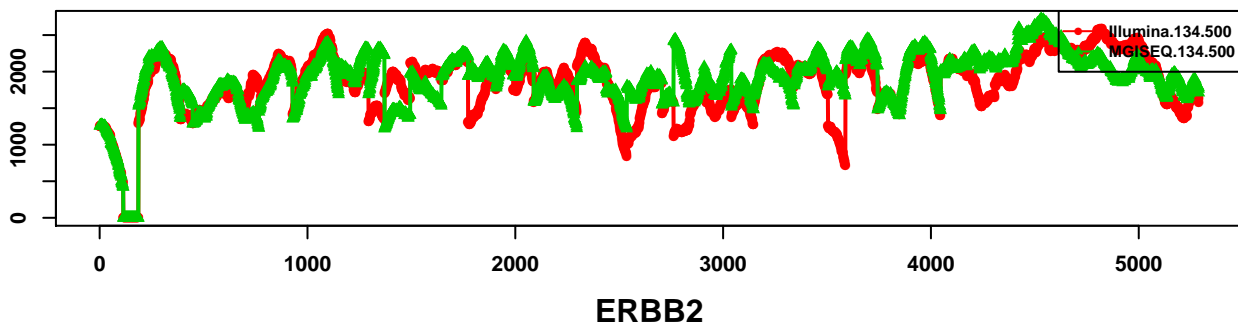

Supplement: Supplementary file 7 [file Presentation7.zip › ERBB2/19GY94044P.pdf]

Sequencing Depth

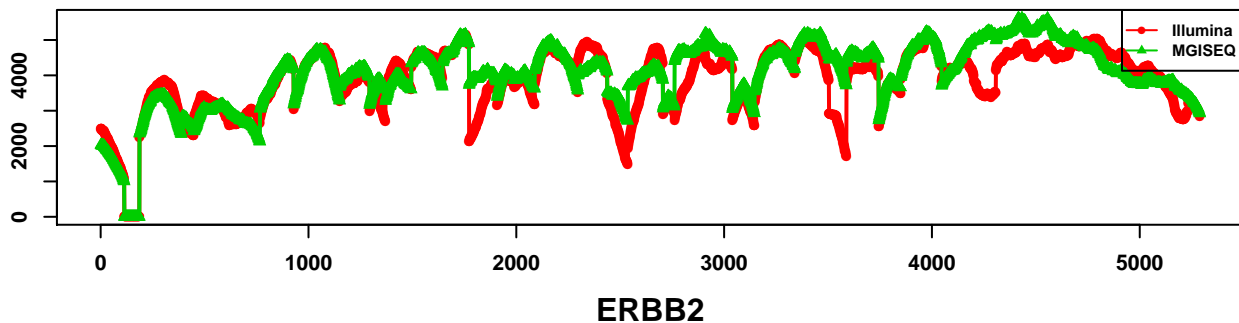

Sequencing Depth

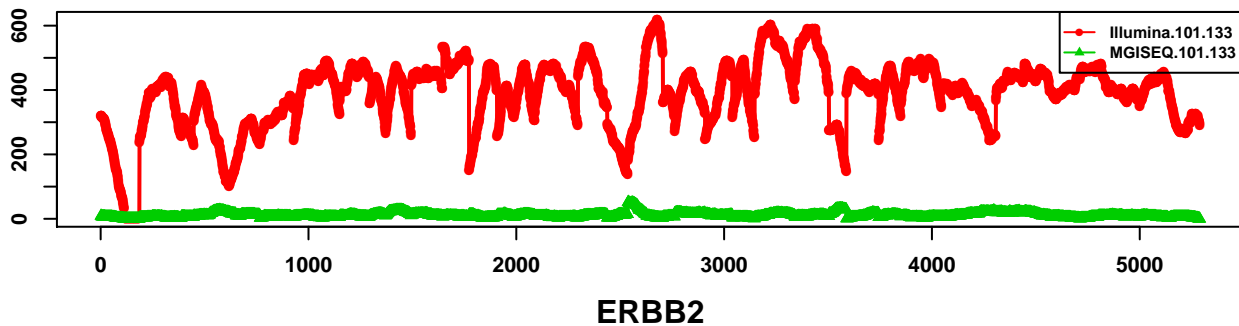

Sequencing Depth

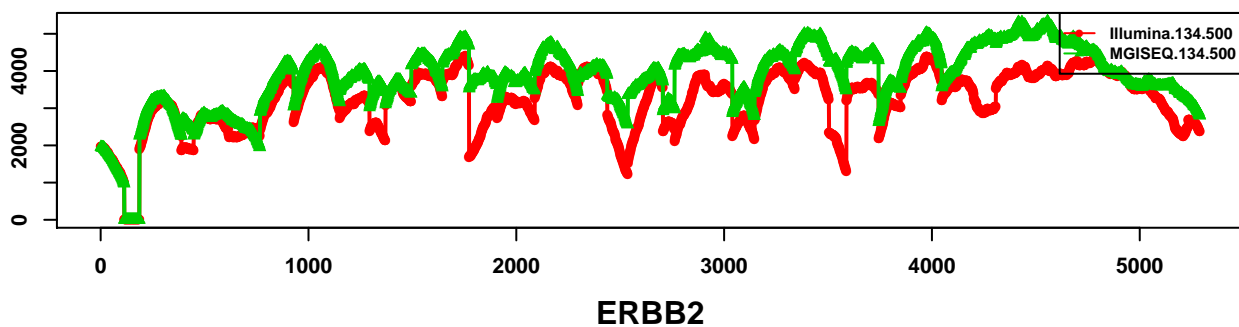

Supplement: Supplementary file 7 [file Presentation7.zip › ERBB2/19JS48179F.pdf]

Sequencing Depth

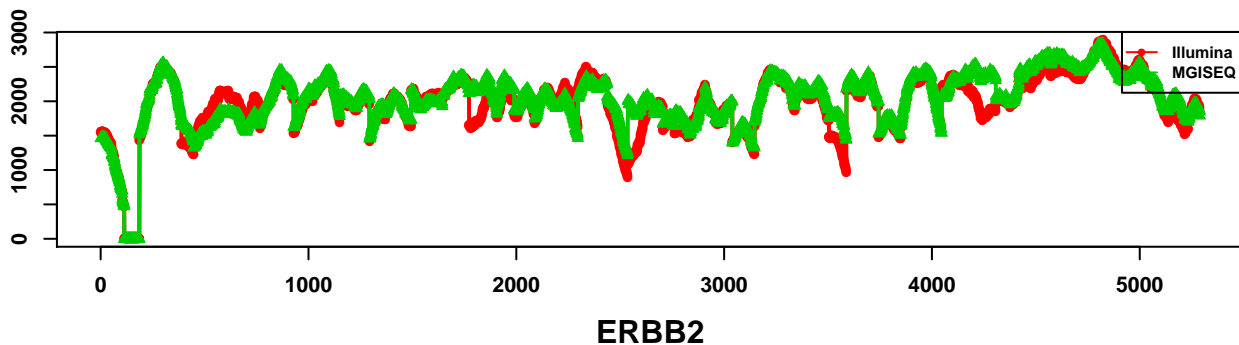

Sequencing Depth

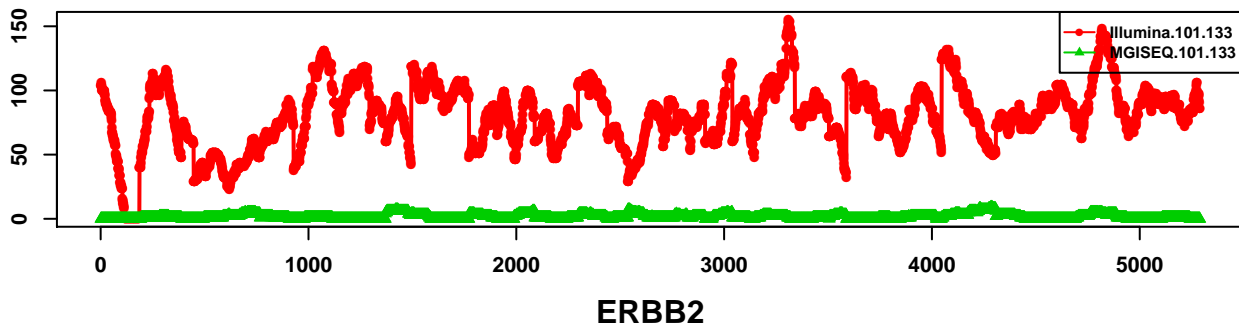

Sequencing Depth

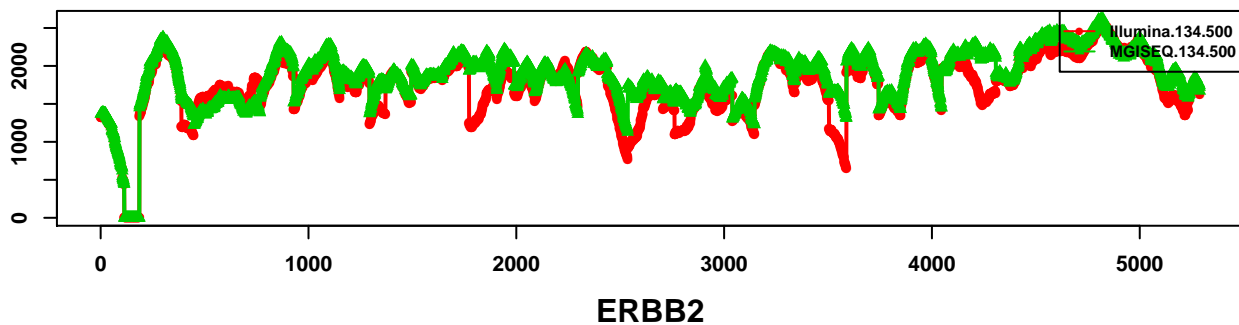

Supplement: Supplementary file 7 [file Presentation7.zip › ERBB2/19N01682P.pdf]

Sequencing Depth

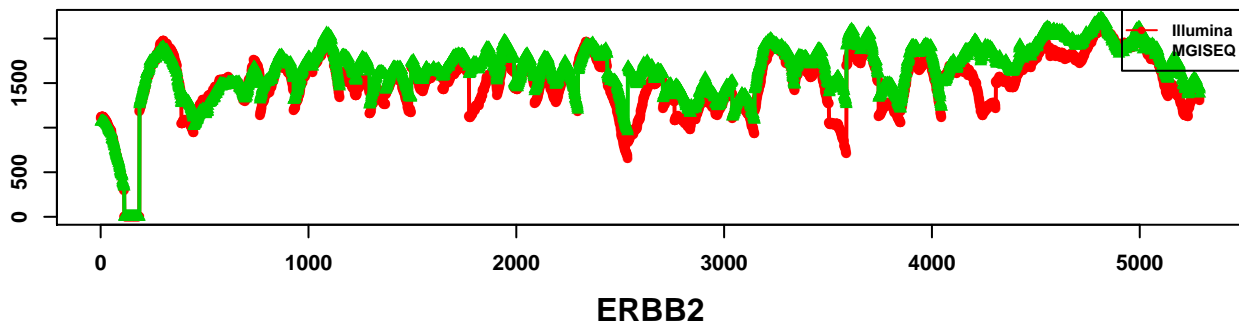

Sequencing Depth

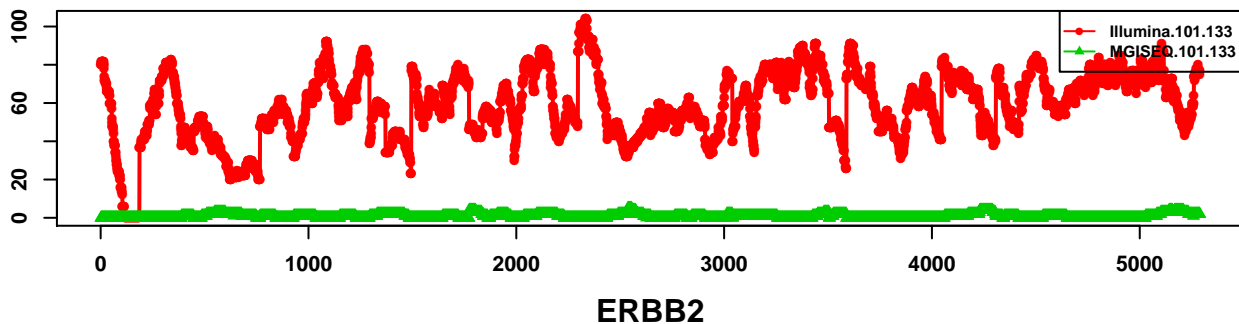

Sequencing Depth

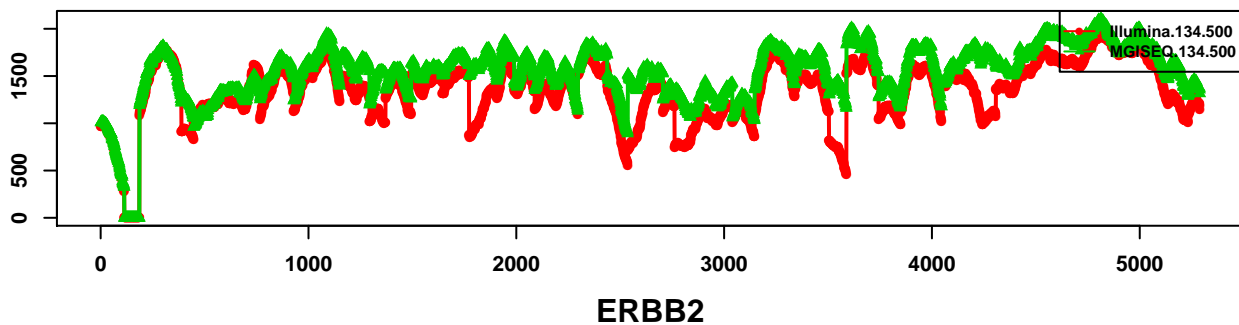

Supplement: Supplementary file 7 [file Presentation7.zip › ERBB2/19N01378P.pdf]

Sequencing Depth

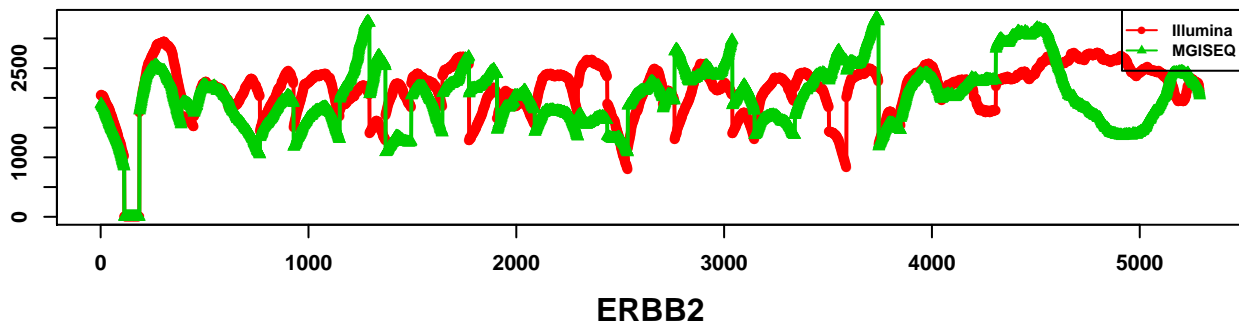

Sequencing Depth

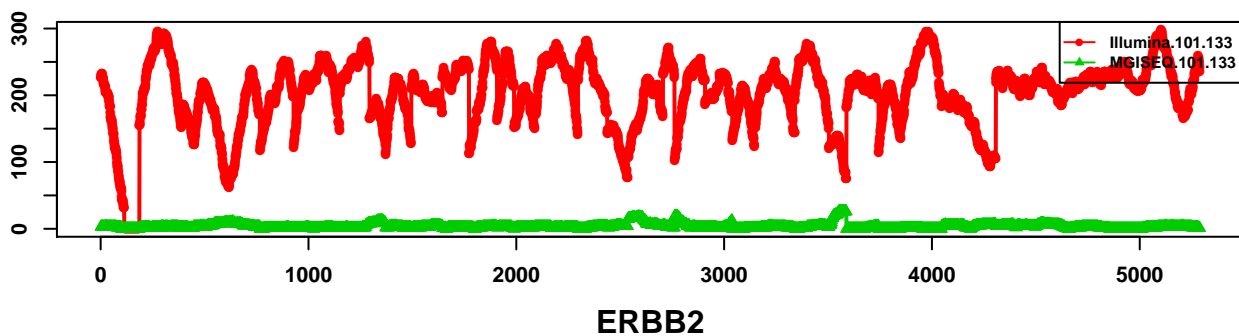

Sequencing Depth

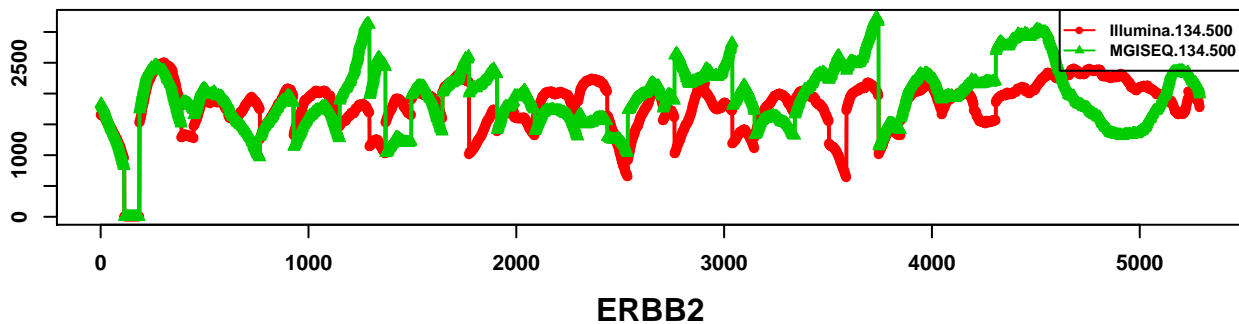

Supplement: Supplementary file 7 [file Presentation7.zip › ERBB2/19HE22170F.pdf]

Sequencing Depth

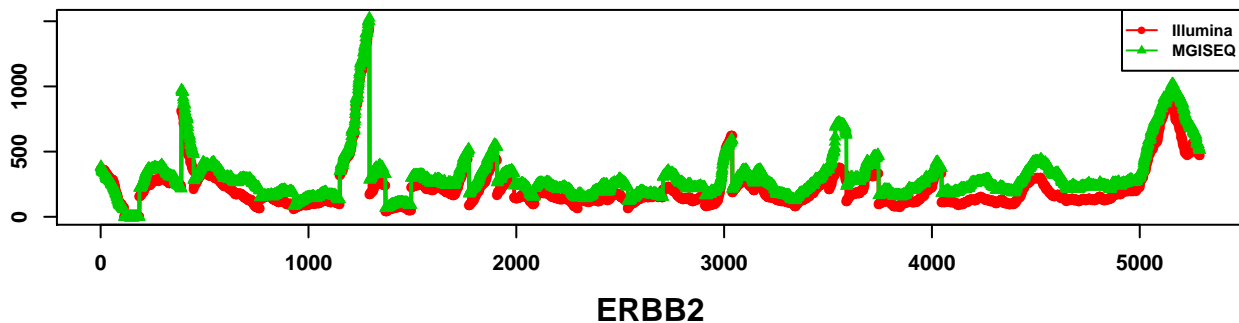

Sequencing Depth

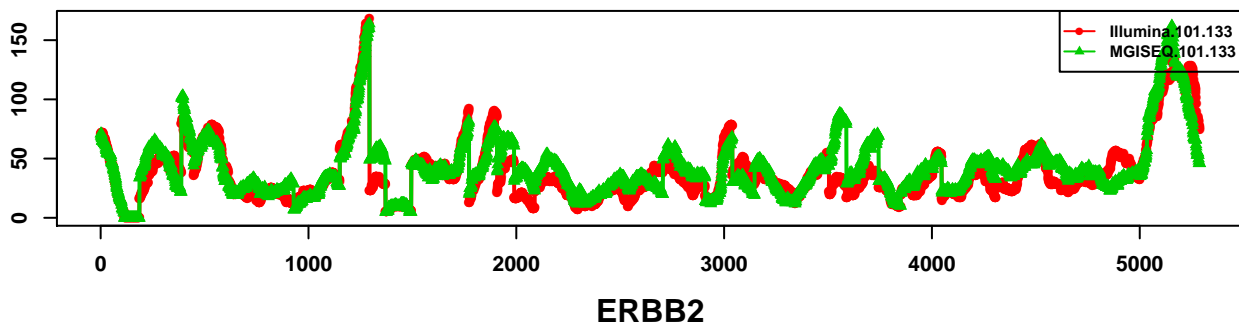

Sequencing Depth

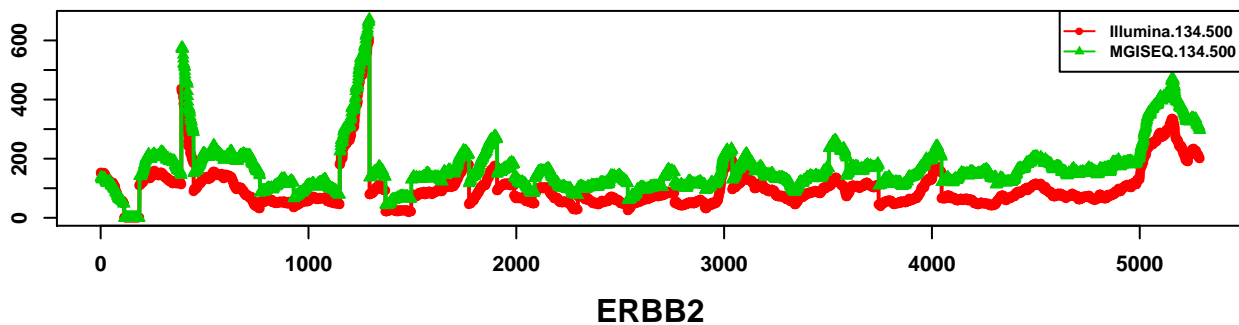

Supplement: Supplementary file 7 [file Presentation7.zip › ERBB2/19FC40390F.pdf]

Sequencing Depth

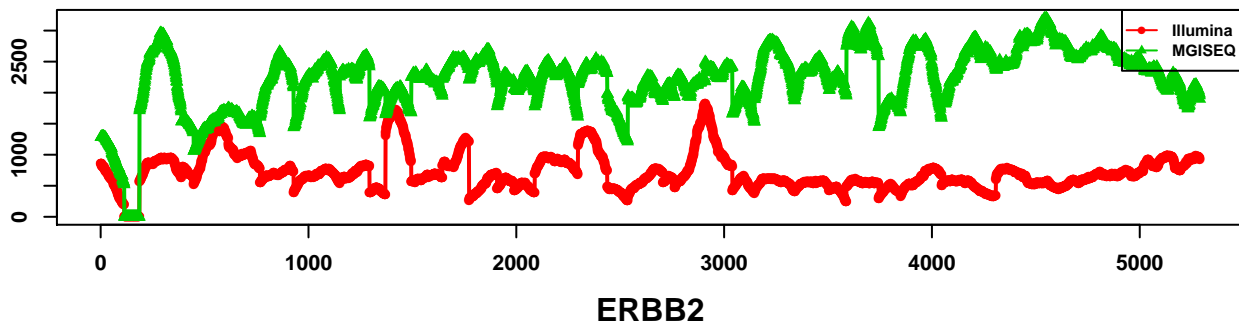

Sequencing Depth

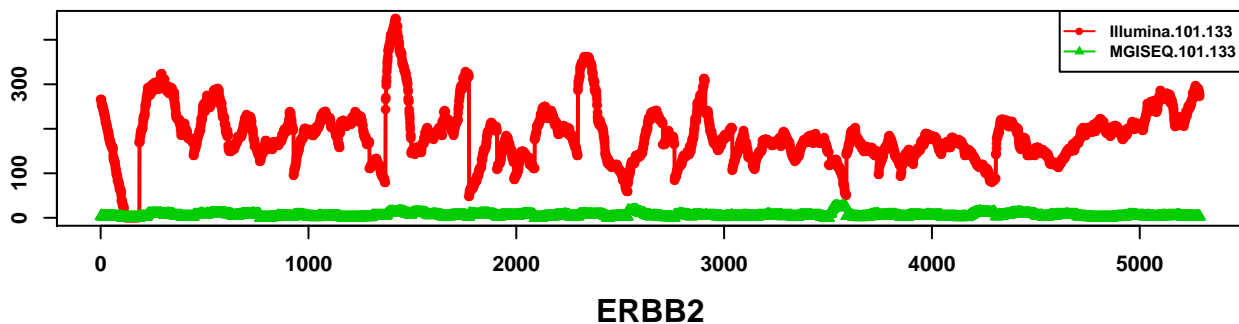

Sequencing Depth

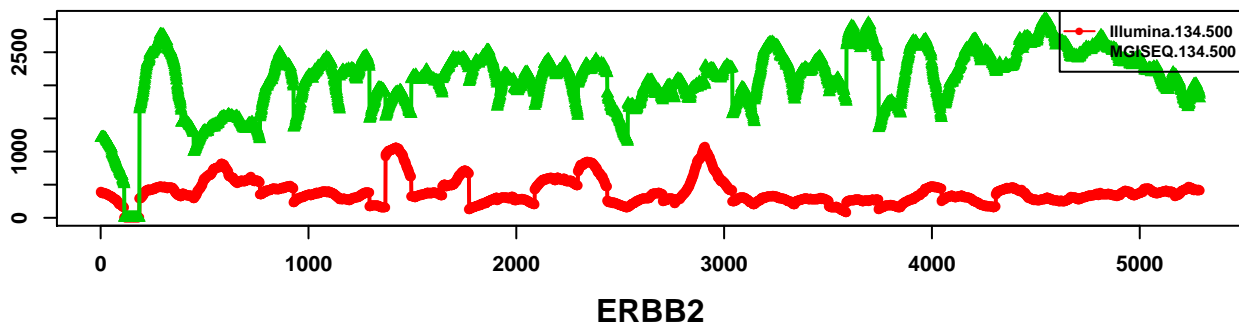

Supplement: Supplementary file 7 [file Presentation7.zip › ERBB2/19HE22131F.pdf]

Sequencing Depth

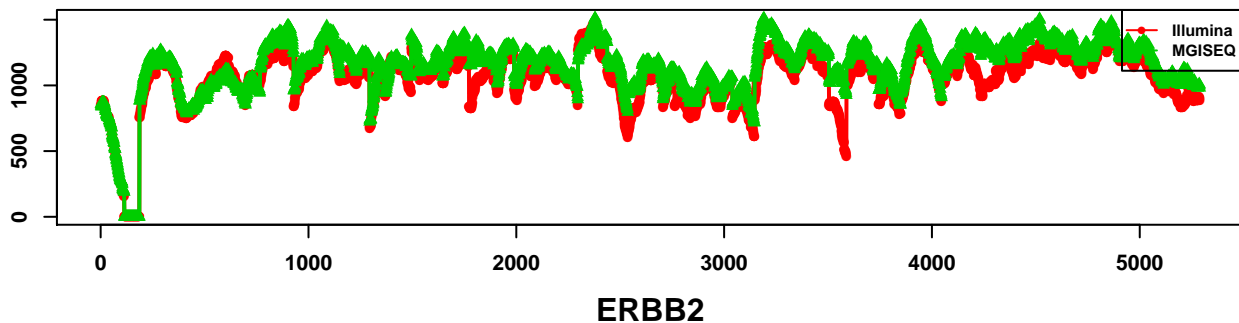

Sequencing Depth

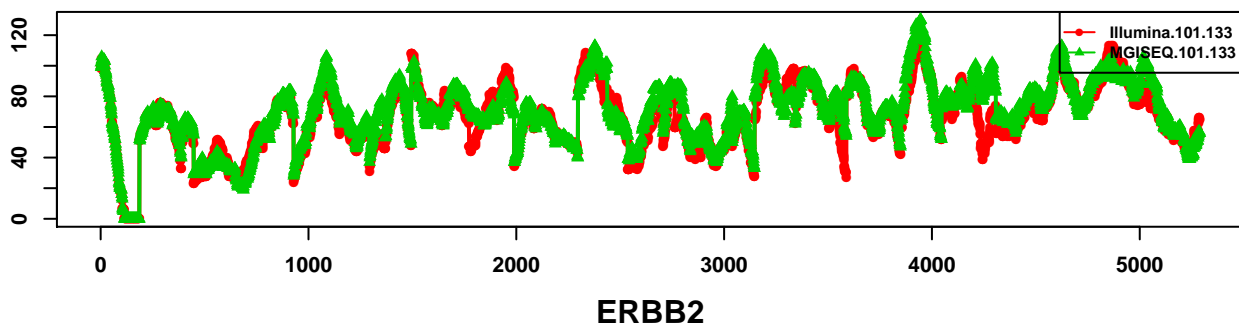

Sequencing Depth

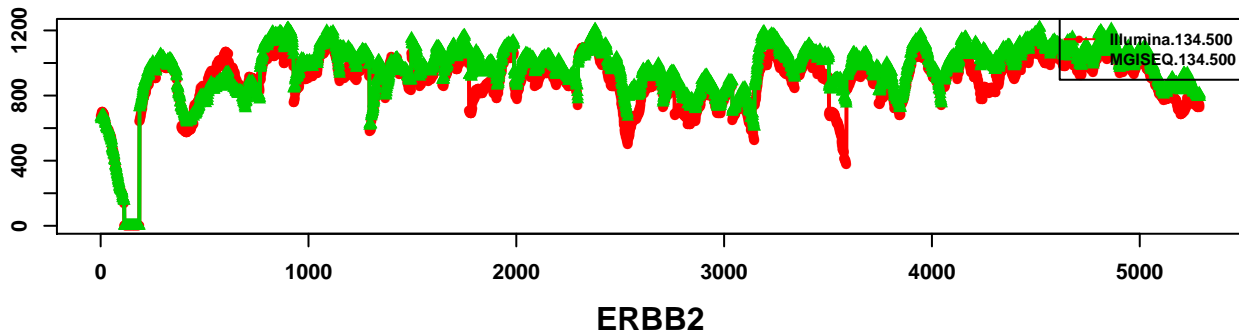

Supplement: Supplementary file 7 [file Presentation7.zip › ERBB2/M1901223P.pdf]

Sequencing Depth

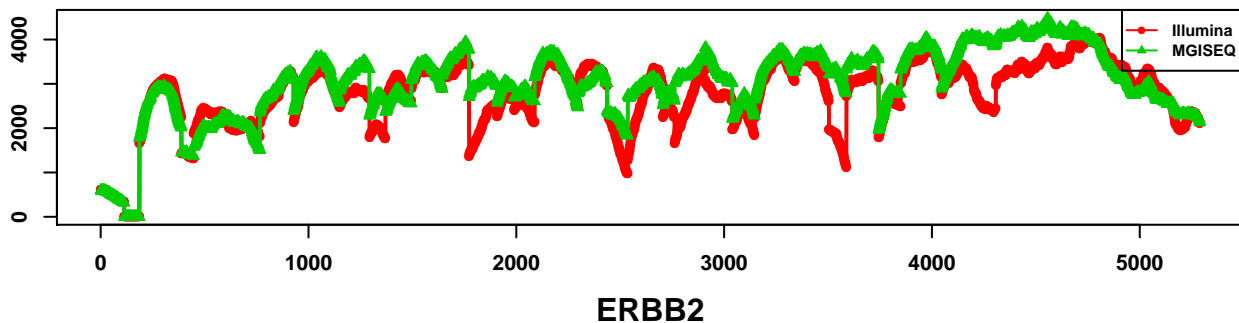

Sequencing Depth

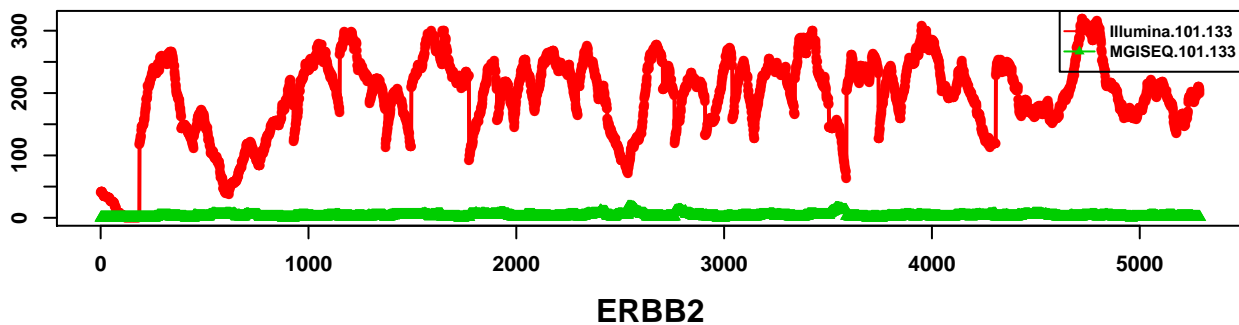

Sequencing Depth

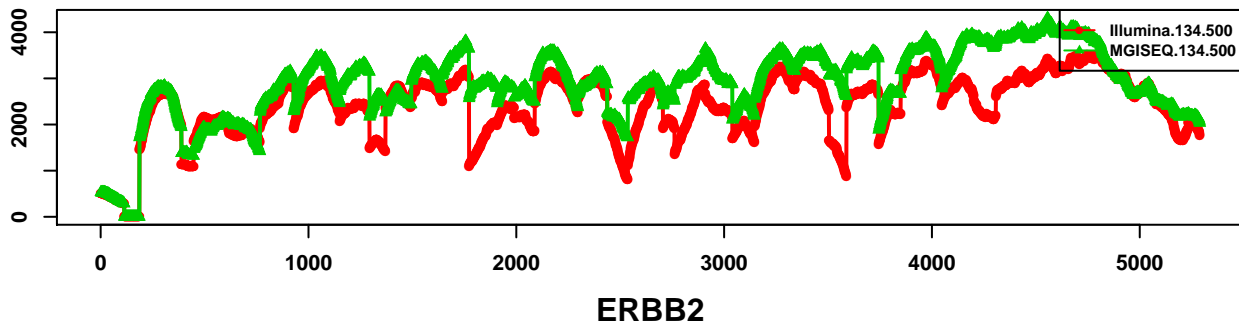

Supplement: Supplementary file 7 [file Presentation7.zip › ERBB2/19N01654H.pdf]

Sequencing Depth

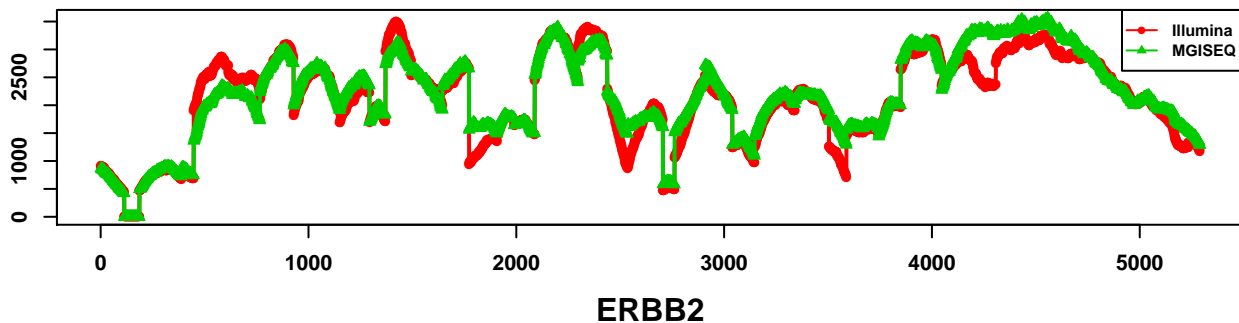

Sequencing Depth

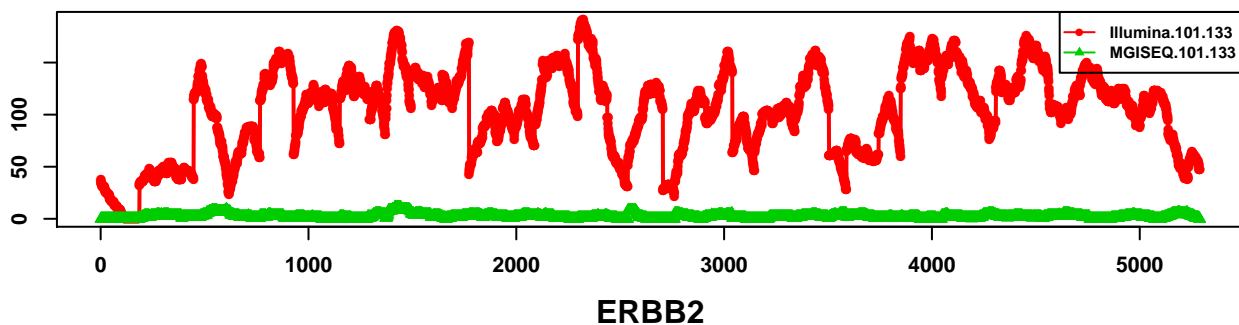

Sequencing Depth

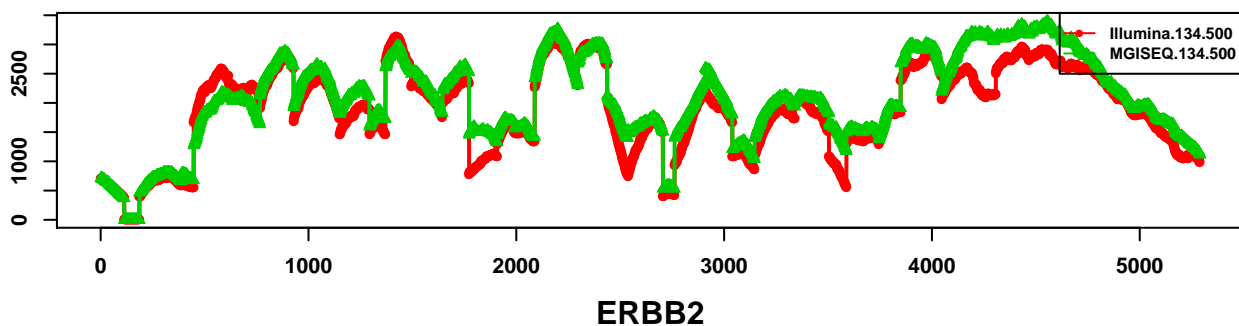

Supplement: Supplementary file 7 [file Presentation7.zip › ERBB2/19N01676T.pdf]

Sequencing Depth

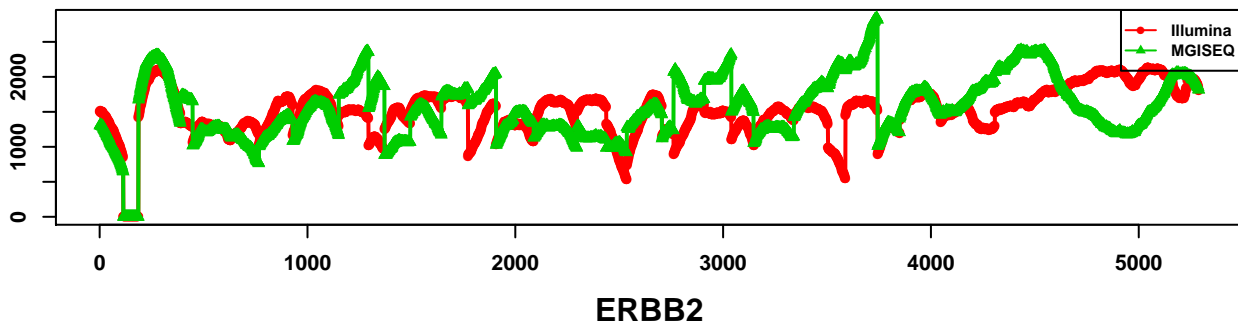

Sequencing Depth

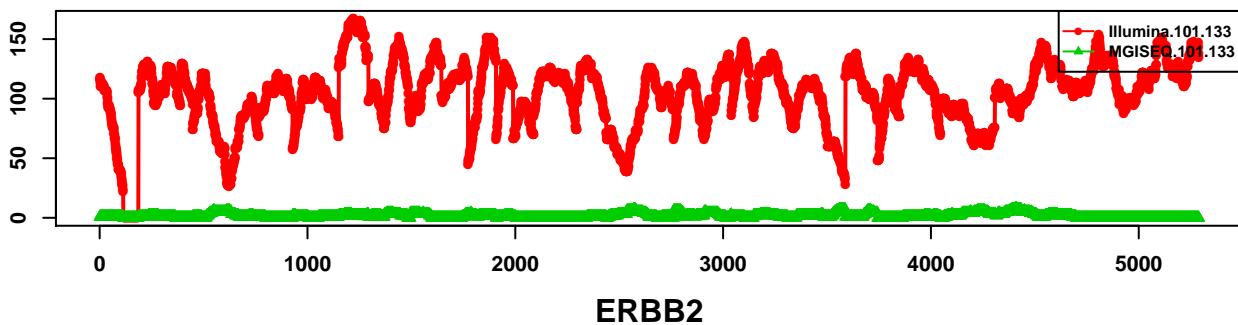

Sequencing Depth

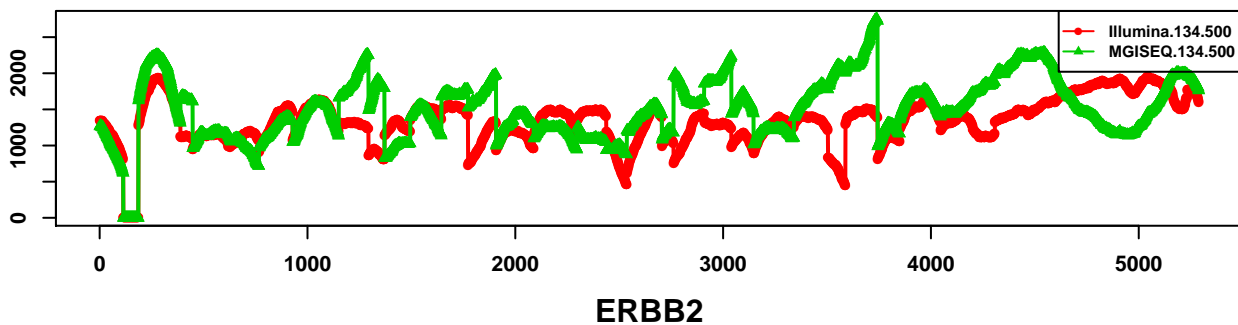

Supplement: Supplementary file 7 [file Presentation7.zip › ERBB2/19FC40257F.pdf]

Sequencing Depth

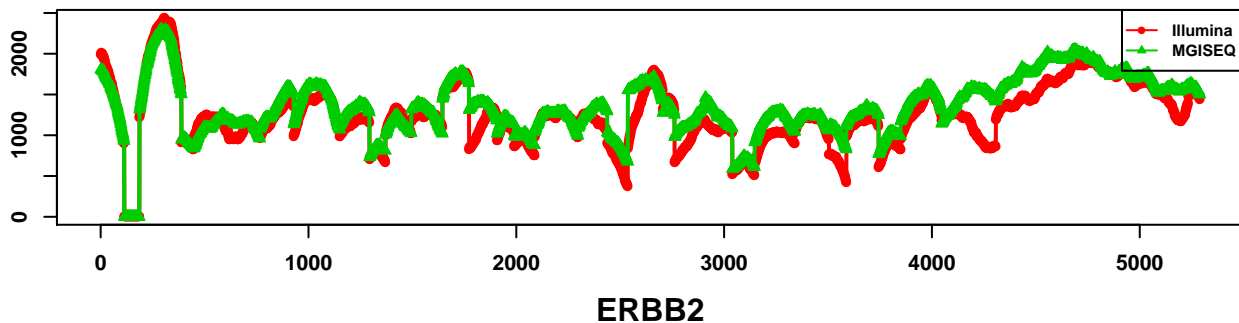

Sequencing Depth

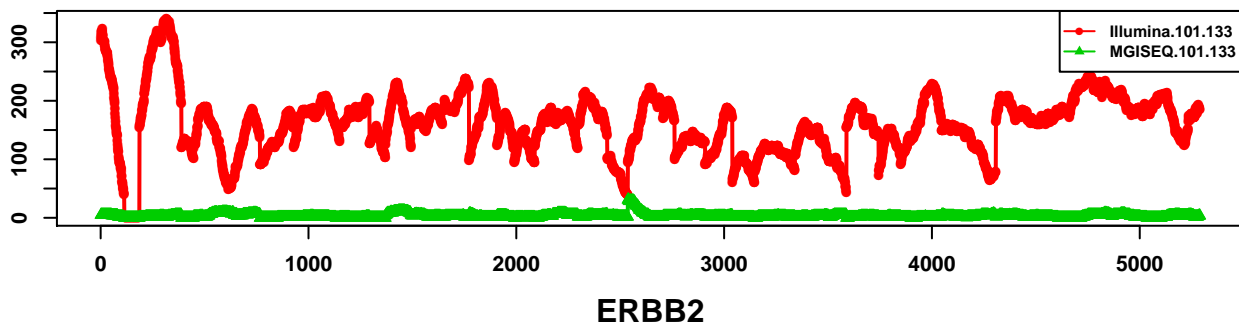

Sequencing Depth

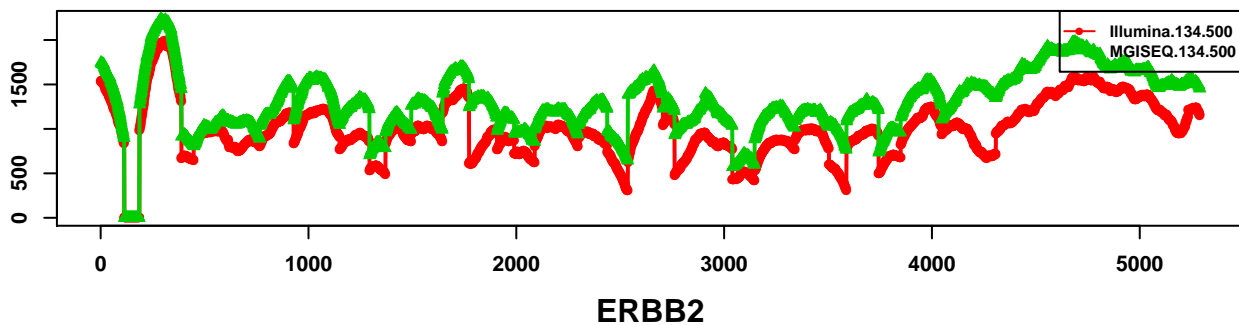

Supplement: Supplementary file 7 [file Presentation7.zip › ERBB2/19CF15540F.pdf]

Sequencing Depth

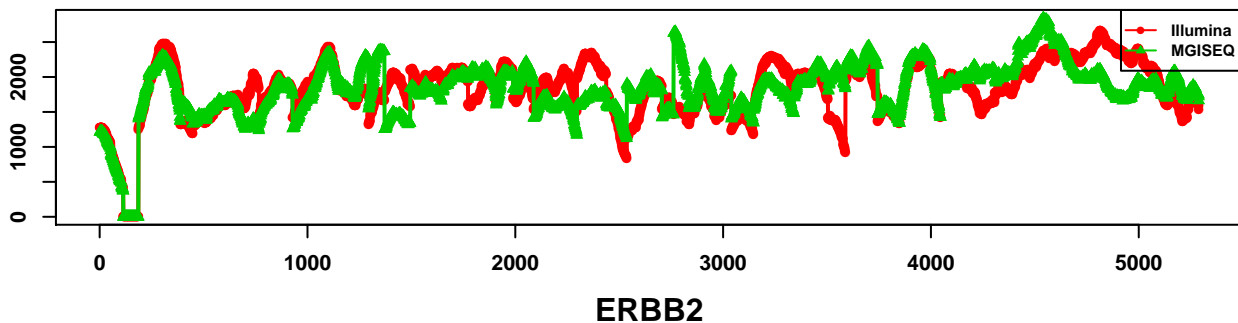

Sequencing Depth

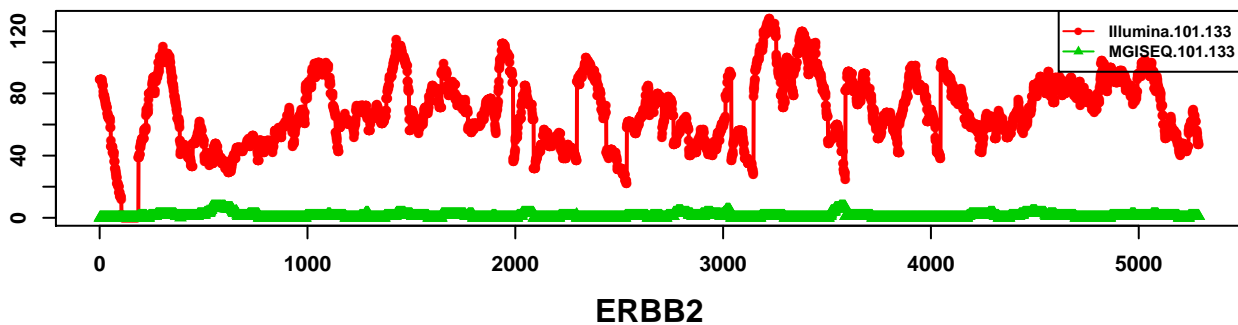

Sequencing Depth

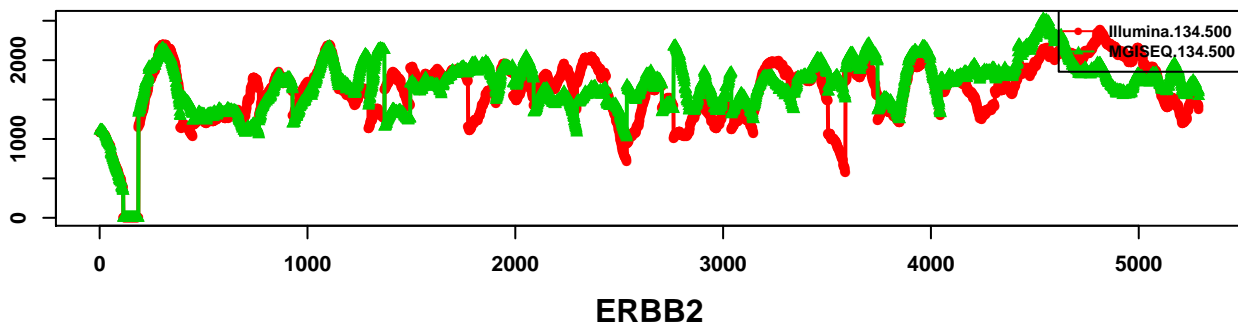

Supplement: Supplementary file 7 [file Presentation7.zip › ERBB2/19ZN12366P.pdf]

Sequencing Depth

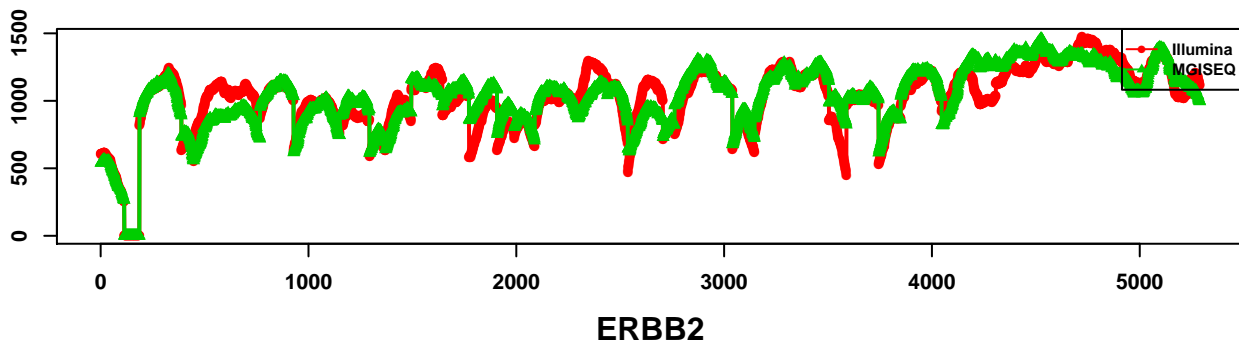

Sequencing Depth

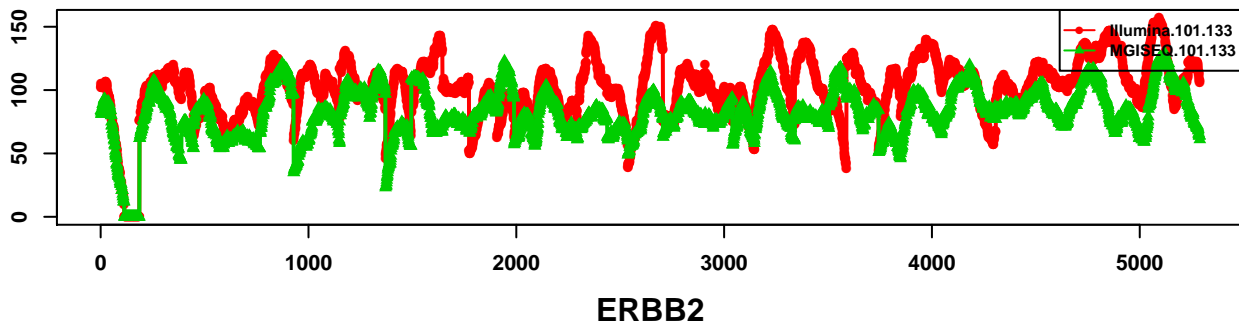

Sequencing Depth

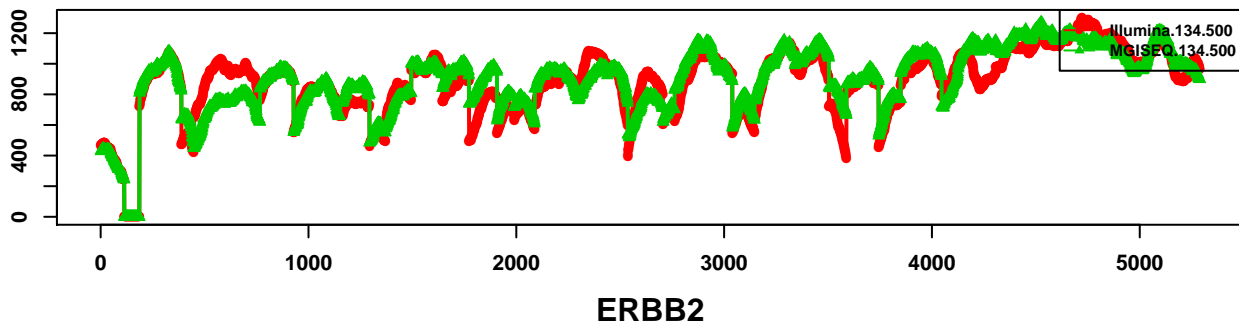

Supplement: Supplementary file 7 [file Presentation7.zip › ERBB2/19GY94073B.pdf]

Sequencing Depth

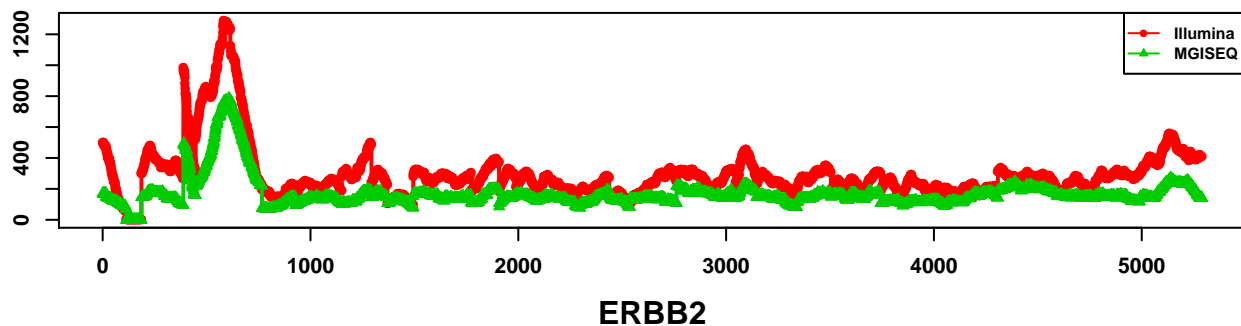

Sequencing Depth

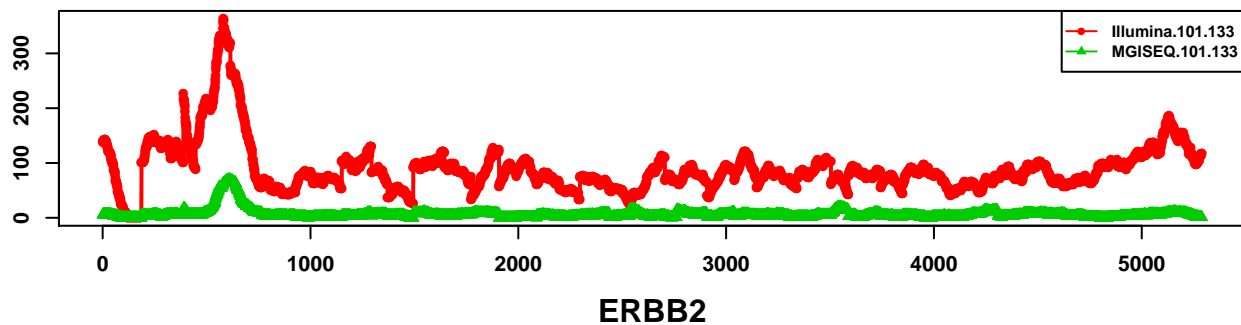

Sequencing Depth

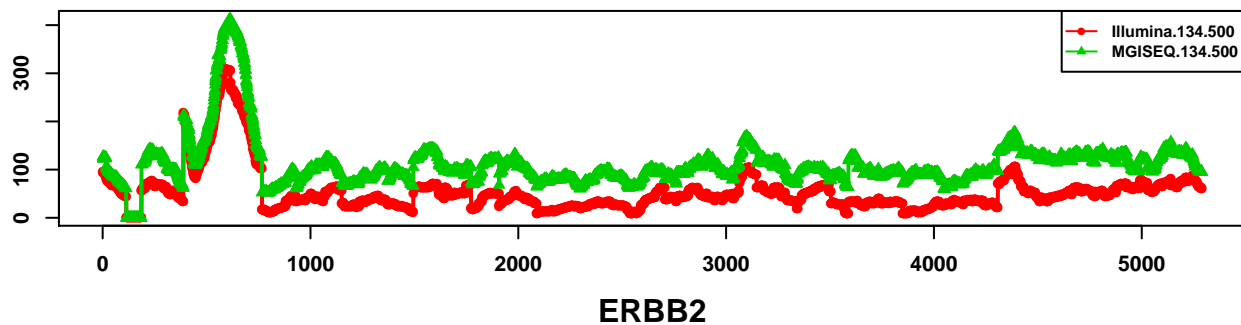

Supplement: Supplementary file 7 [file Presentation7.zip › ERBB2/19ZN12357F.pdf]

Sequencing Depth

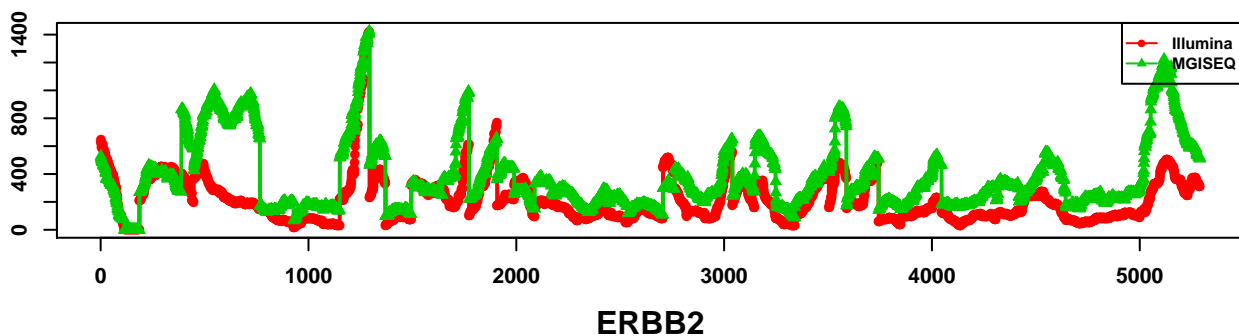

Sequencing Depth

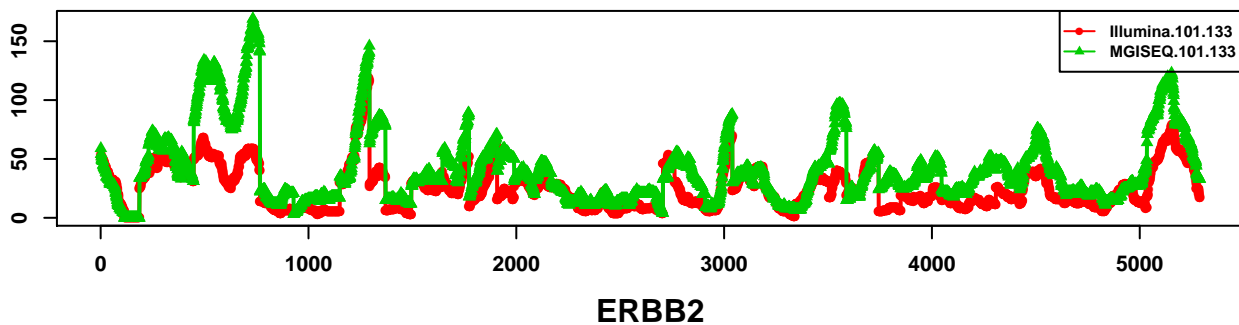

Sequencing Depth

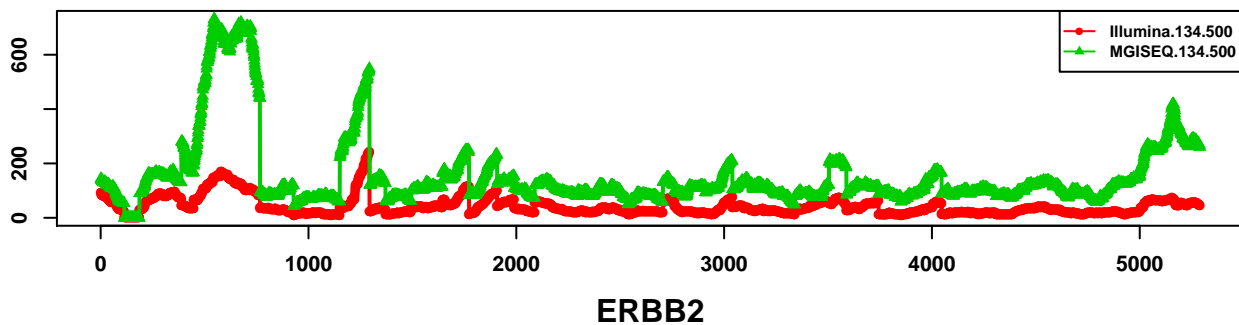

Supplement: Supplementary file 7 [file Presentation7.zip › ERBB2/19N02343F.pdf]

Sequencing Depth

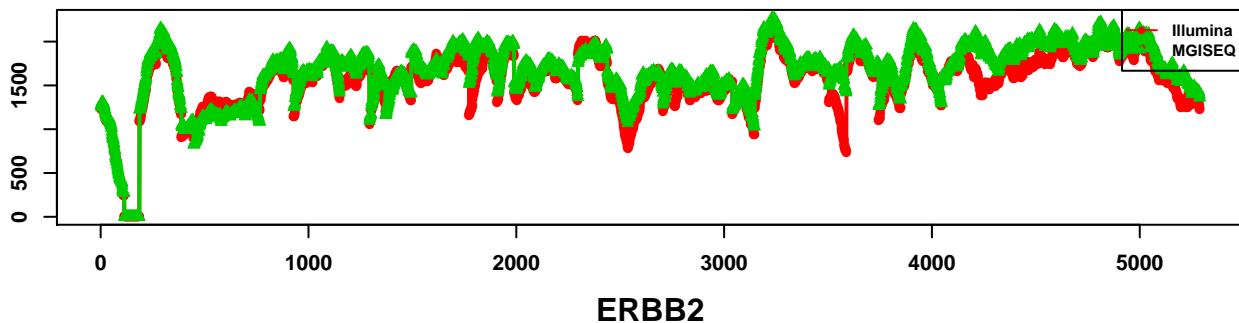

Sequencing Depth

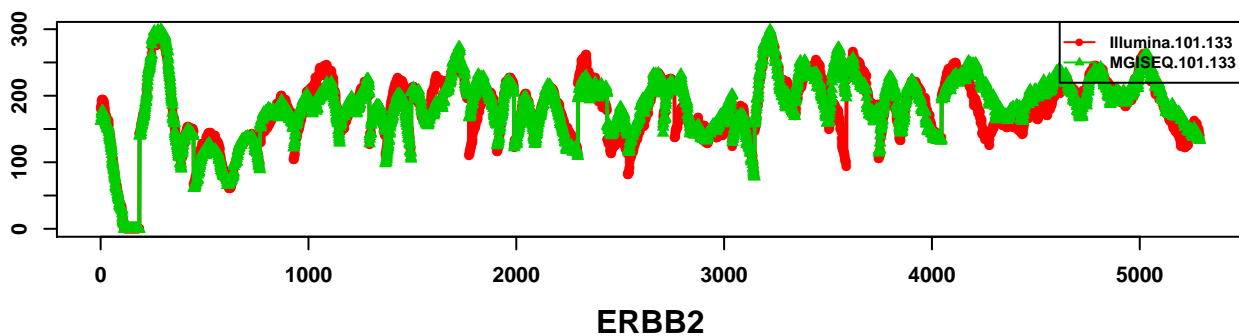

Sequencing Depth

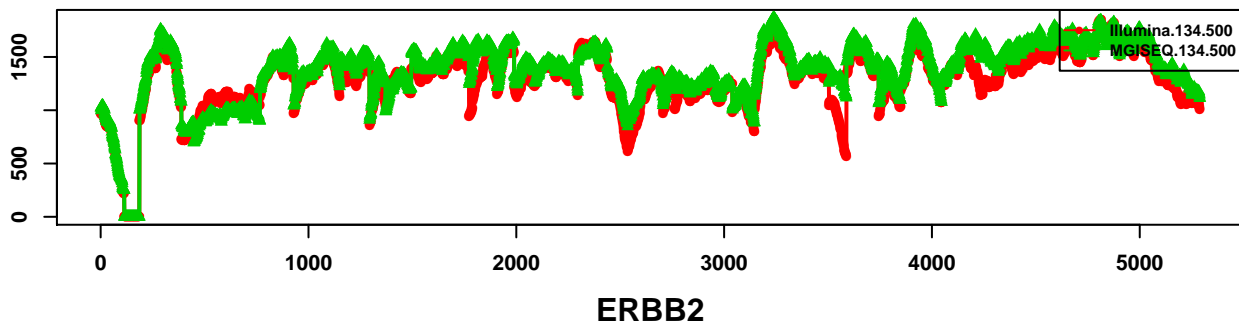

Supplement: Supplementary file 8 [file Presentation8.zip › ERBB2/19ZN12547P.pdf]

Sequencing Depth

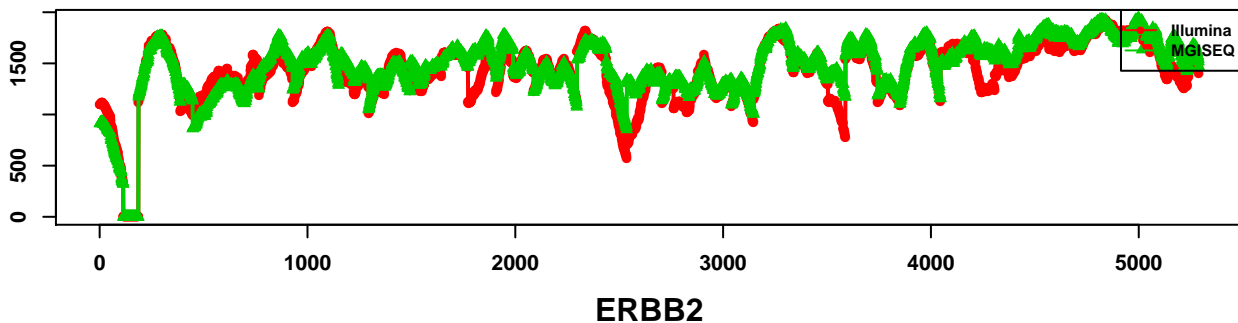

Sequencing Depth

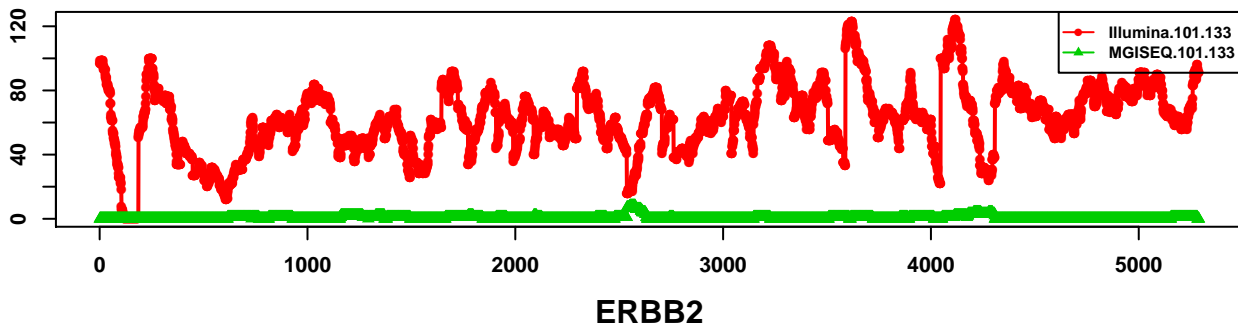

Sequencing Depth

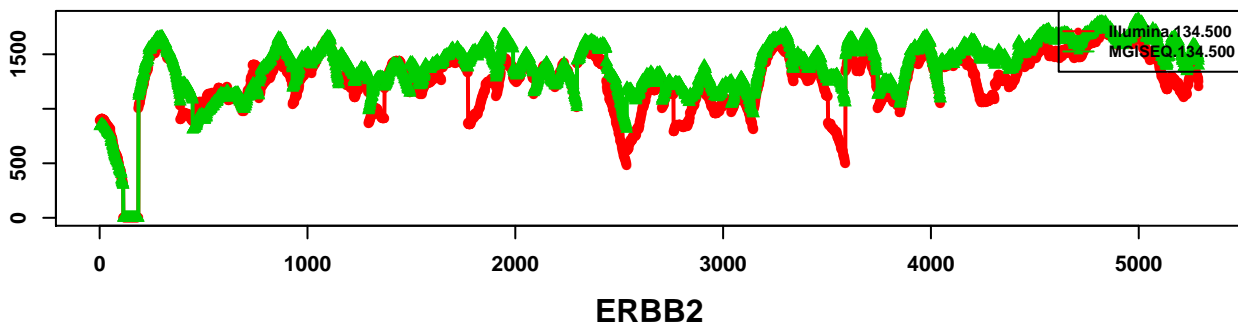

Supplement: Supplementary file 8 [file Presentation8.zip › ERBB2/19ZN13100P.pdf]

Sequencing Depth

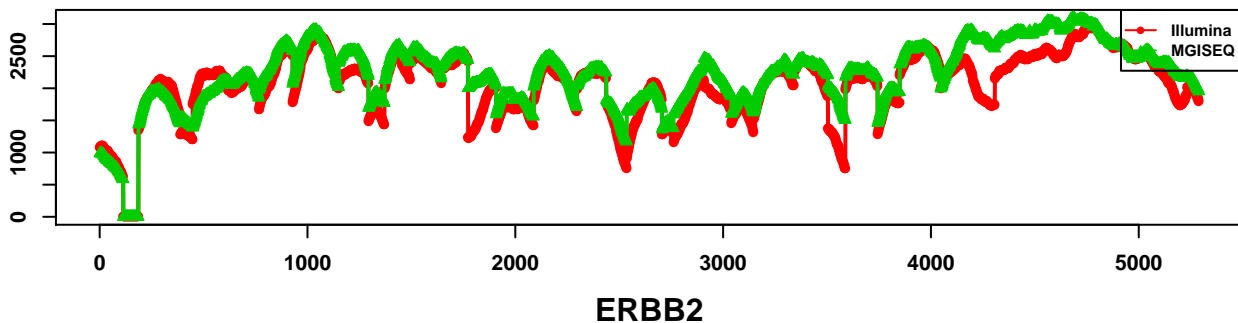

Sequencing Depth

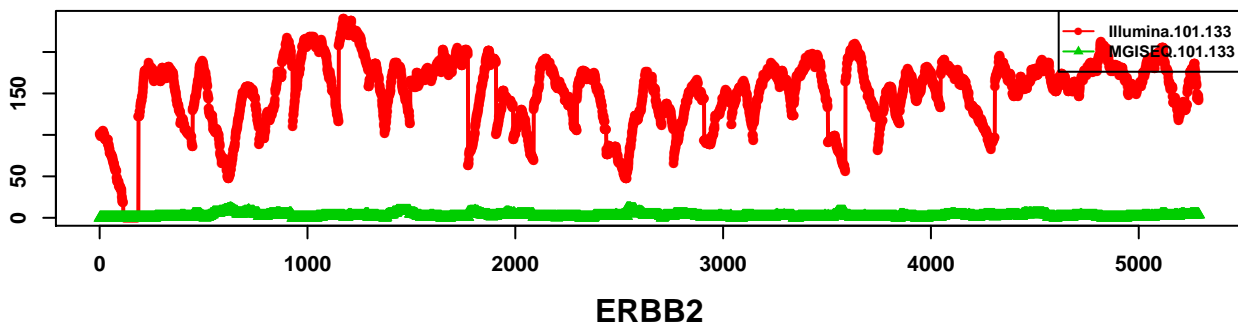

Sequencing Depth

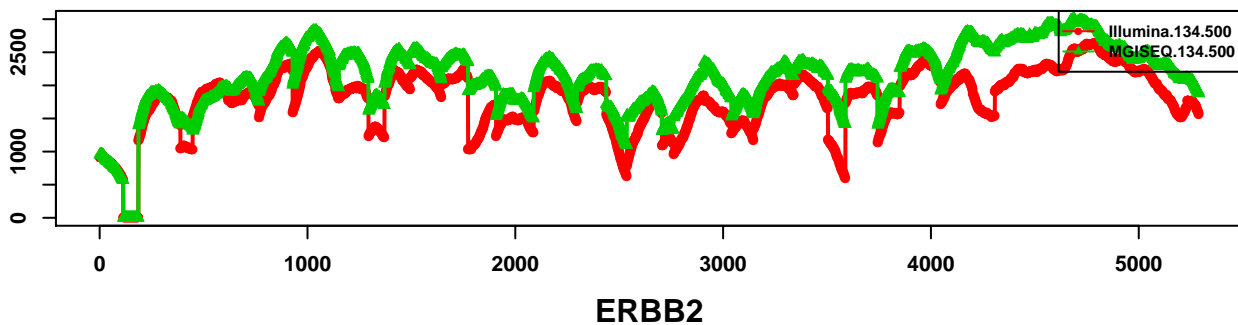

Supplement: Supplementary file 8 [file Presentation8.zip › ERBB2/19ZN13101T.pdf]

Sequencing Depth

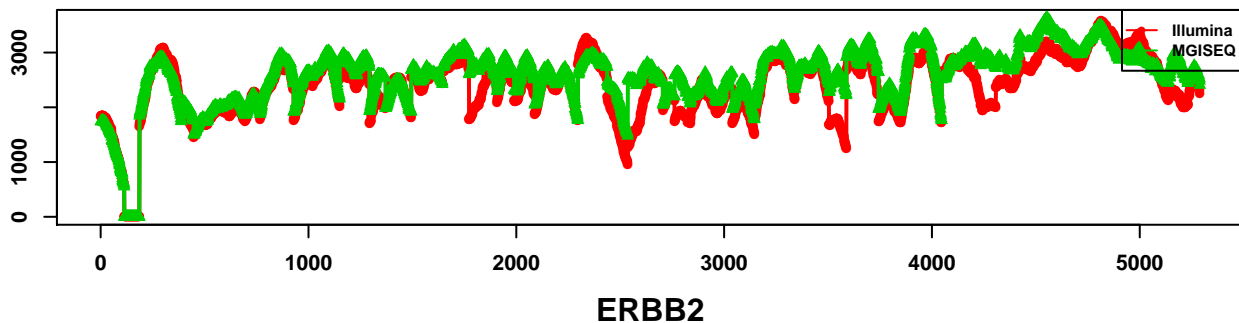

Sequencing Depth

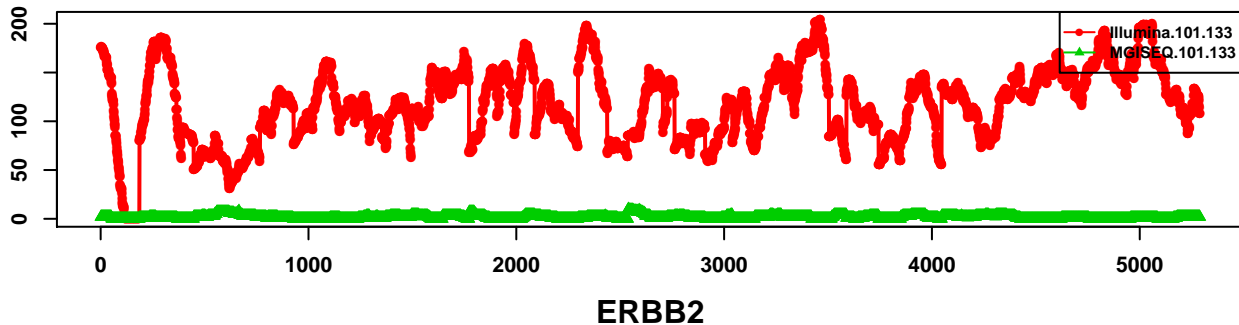

Sequencing Depth

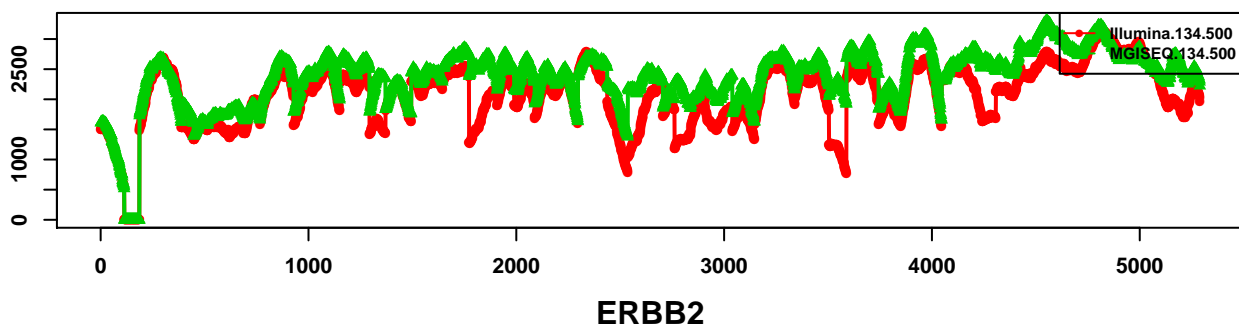

Supplement: Supplementary file 8 [file Presentation8.zip › ERBB2/19N01650P.pdf]

Sequencing Depth

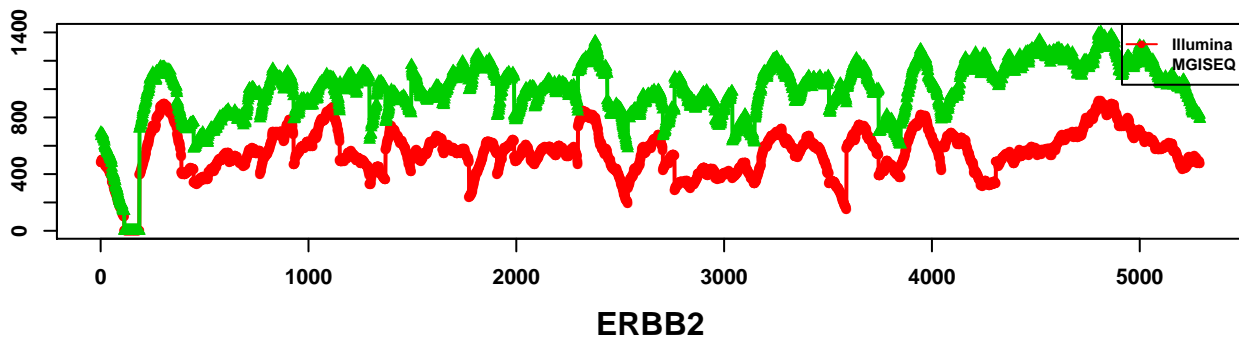

Sequencing Depth

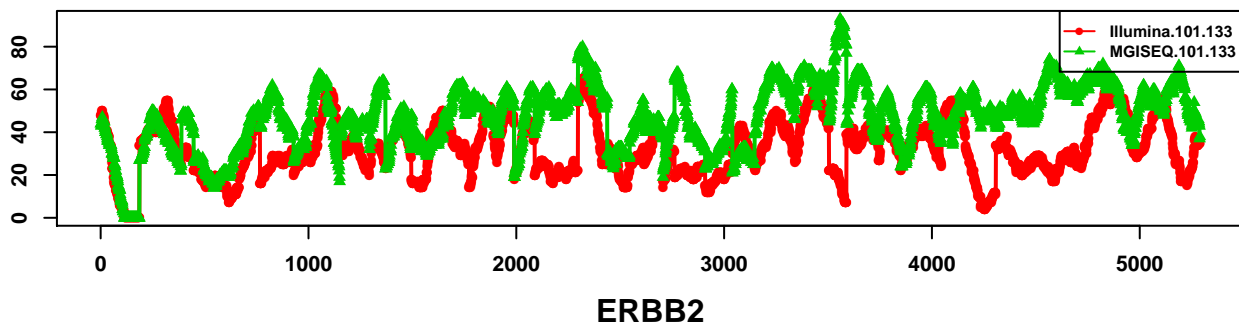

Sequencing Depth

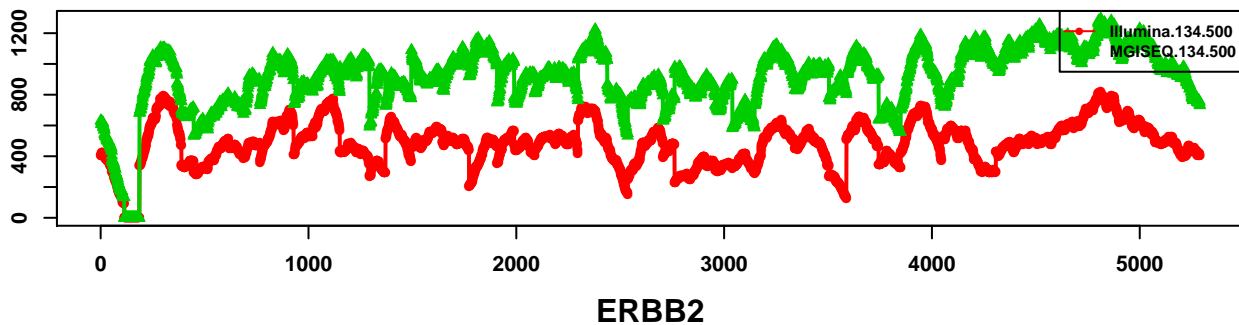

Supplement: Supplementary file 8 [file Presentation8.zip › ERBB2/19ZN12584P.pdf]

Sequencing Depth

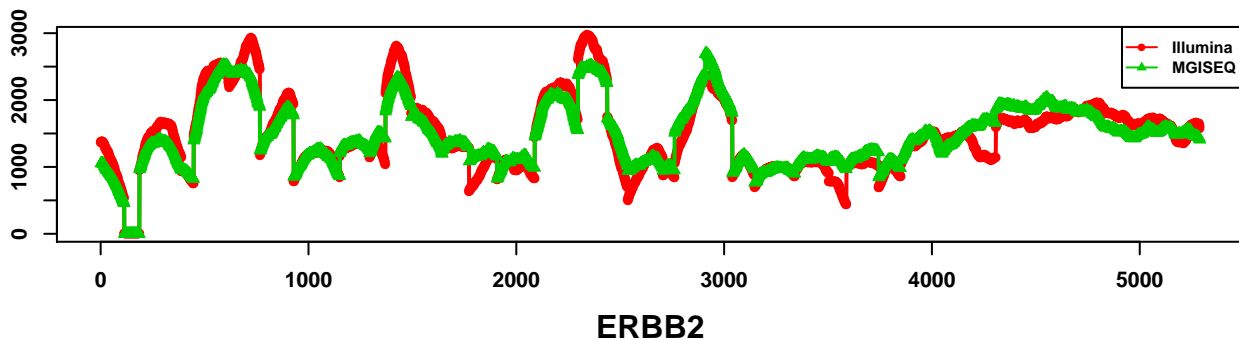

Sequencing Depth

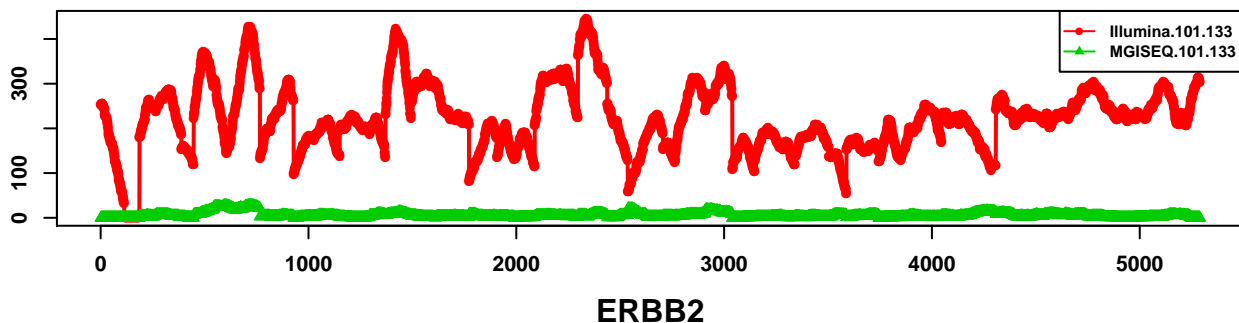

Sequencing Depth

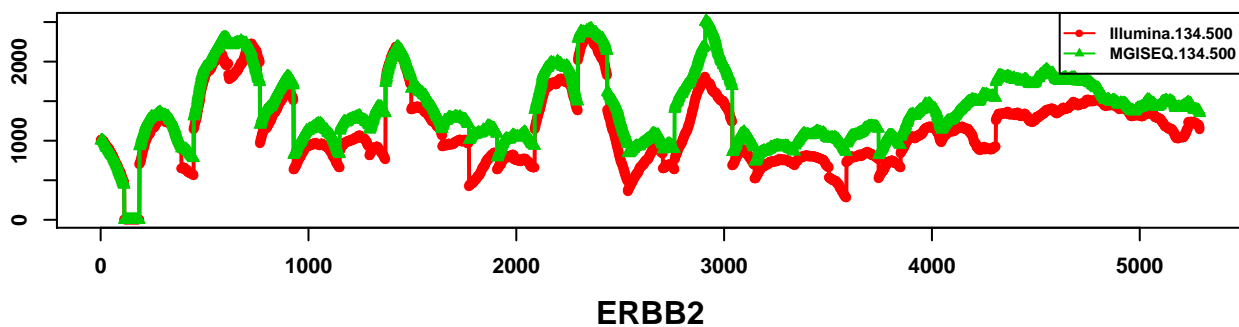

Supplement: Supplementary file 8 [file Presentation8.zip › ERBB2/19ZN11296F.pdf]

Sequencing Depth

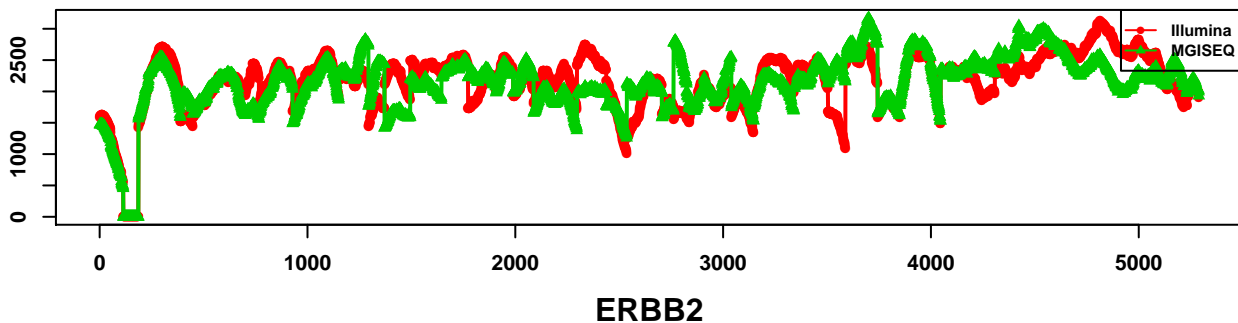

Sequencing Depth

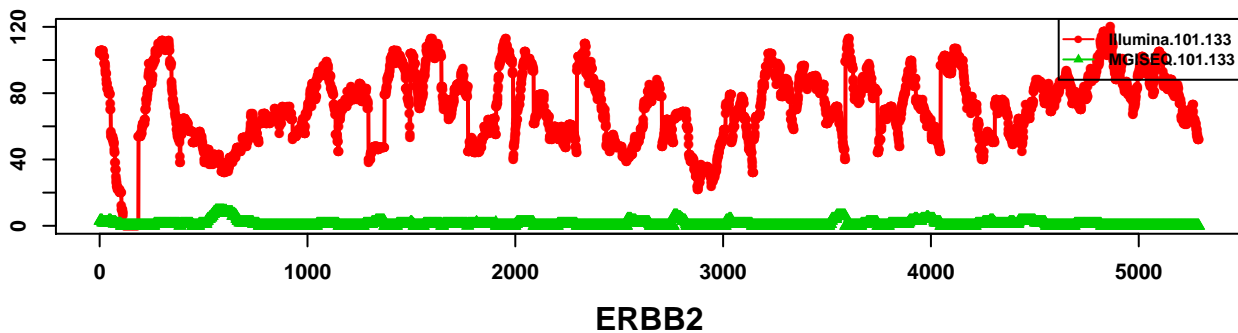

Sequencing Depth

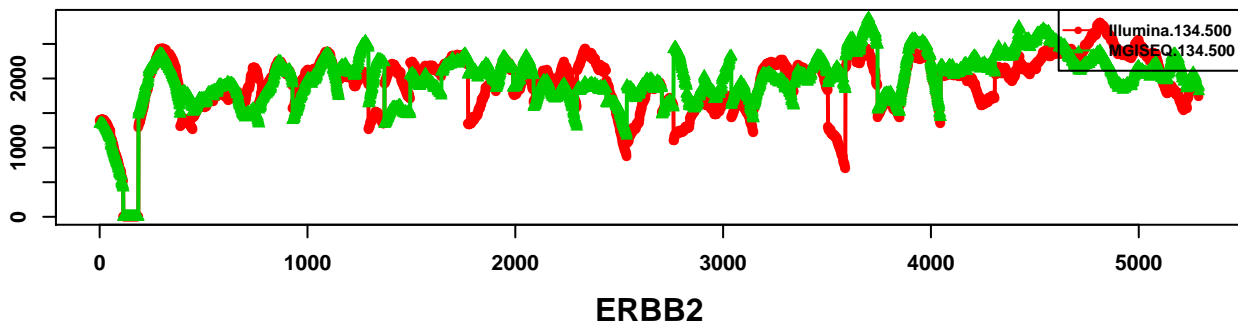

Supplement: Supplementary file 8 [file Presentation8.zip › ERBB2/19N01666P.pdf]

Sequencing Depth

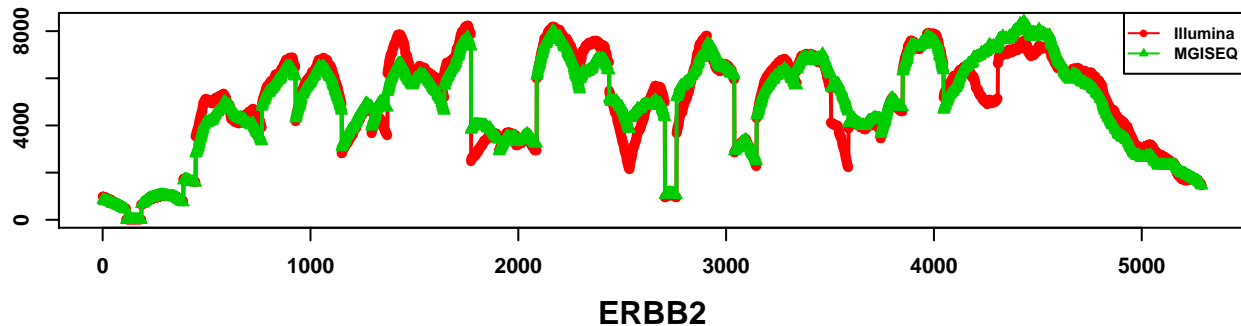

Sequencing Depth

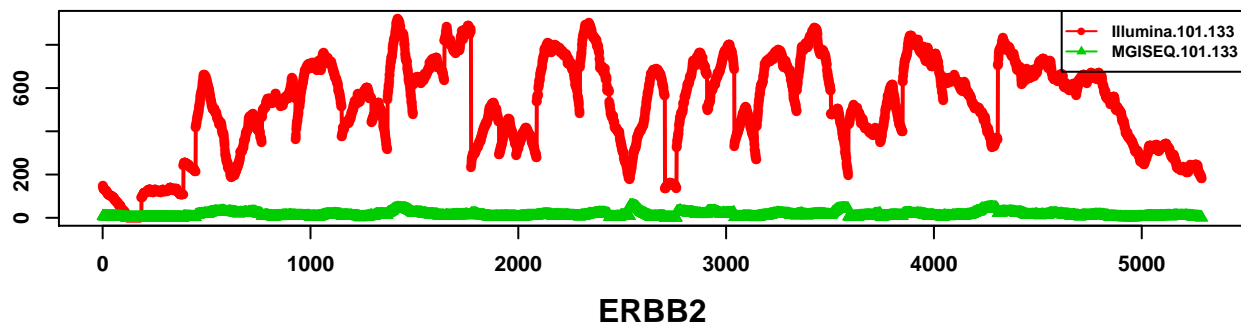

Sequencing Depth

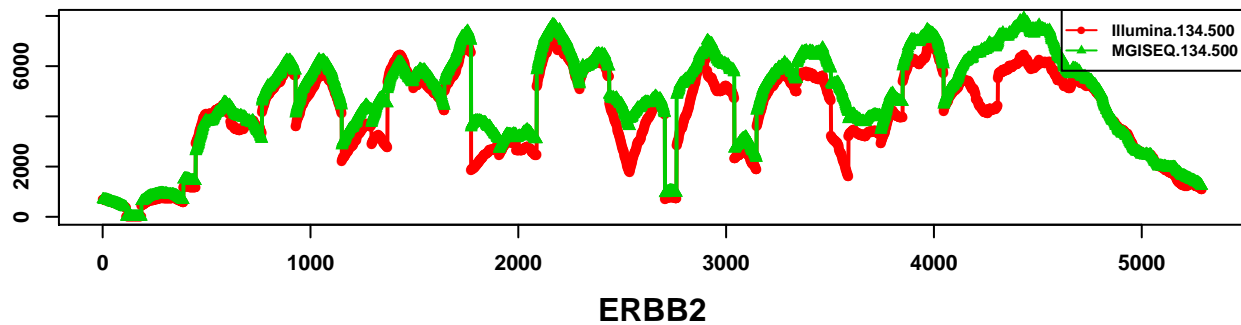

Supplement: Supplementary file 8 [file Presentation8.zip › ERBB2/19FC40246F.pdf]

Sequencing Depth

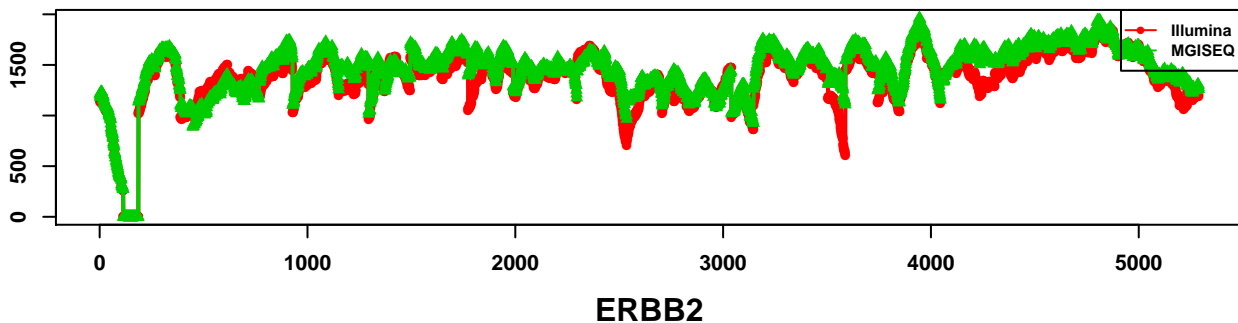

Sequencing Depth

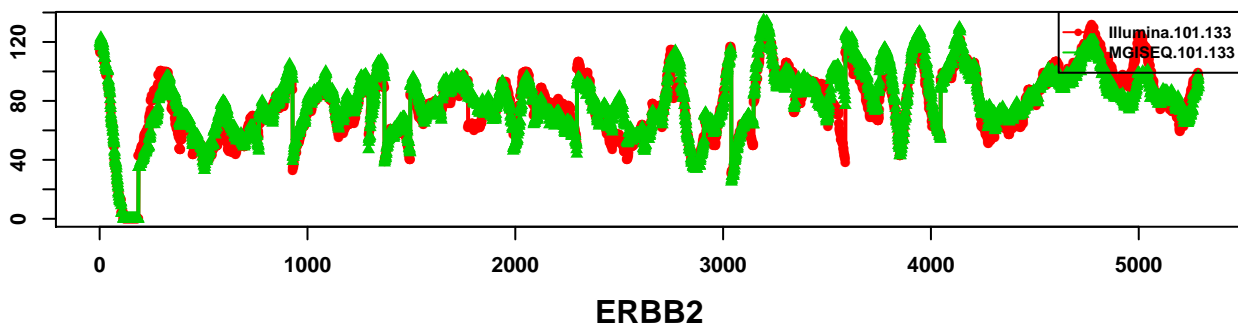

Sequencing Depth

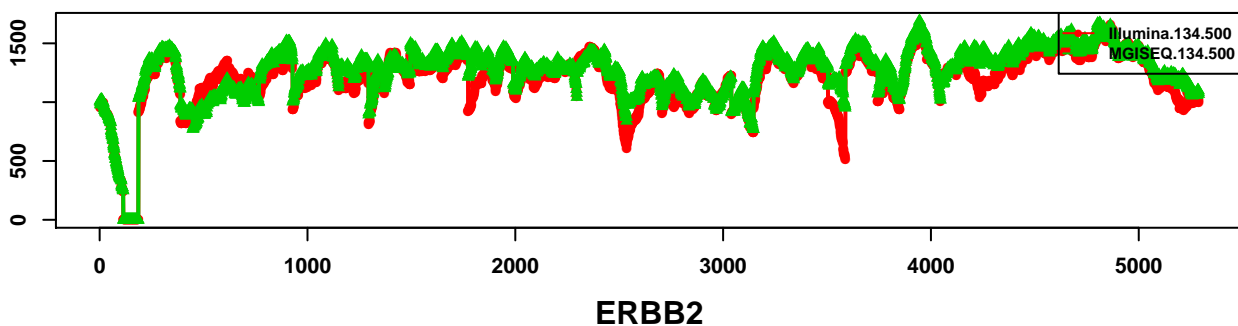

Supplement: Supplementary file 8 [file Presentation8.zip › ERBB2/19CF15711P.pdf]

Sequencing Depth

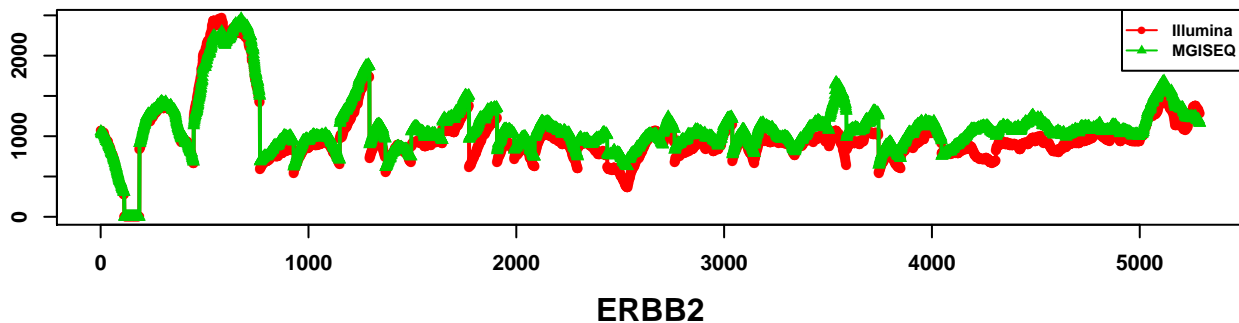

Sequencing Depth

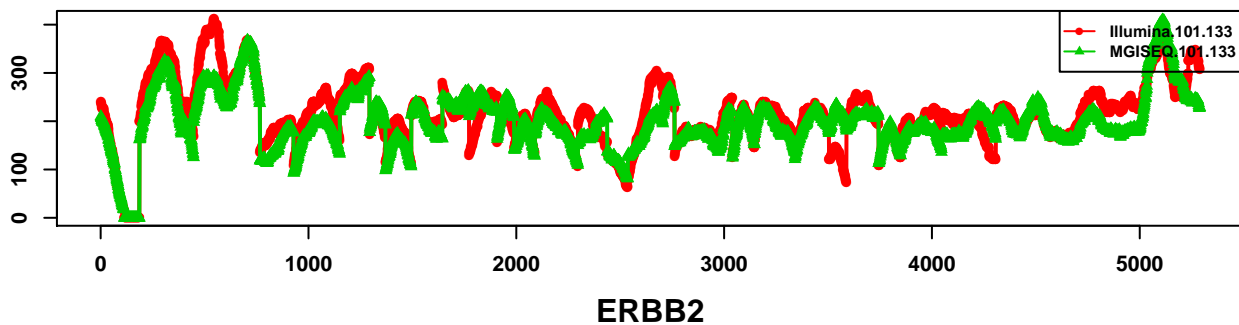

Sequencing Depth

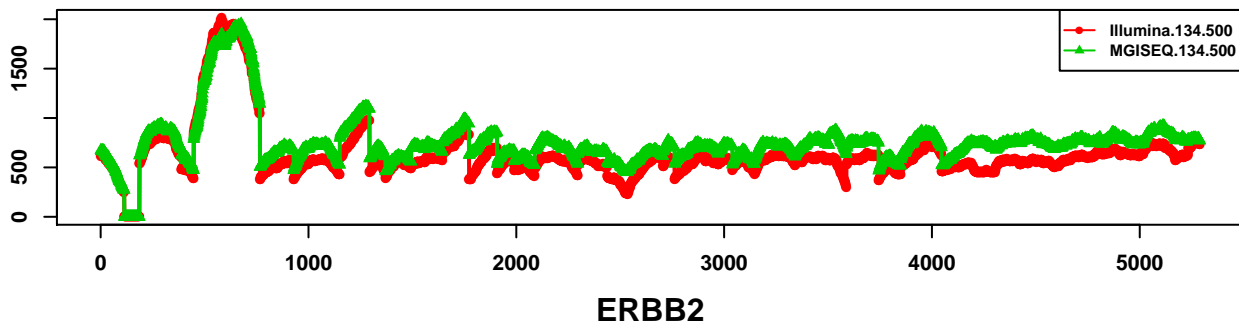

Supplement: Supplementary file 8 [file Presentation8.zip › ERBB2/19Q06297F.pdf]

Sequencing Depth

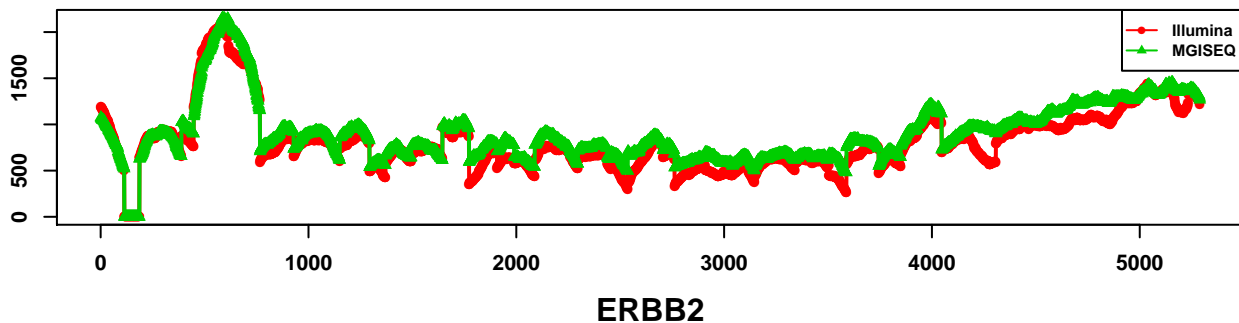

Sequencing Depth

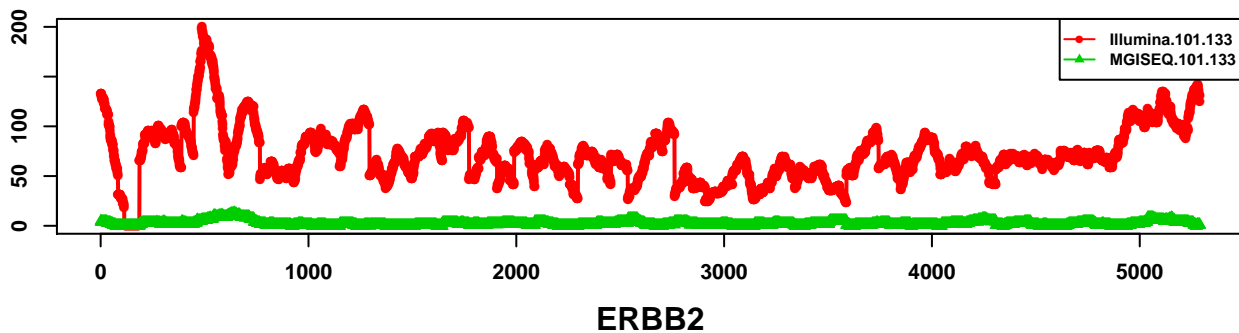

Sequencing Depth

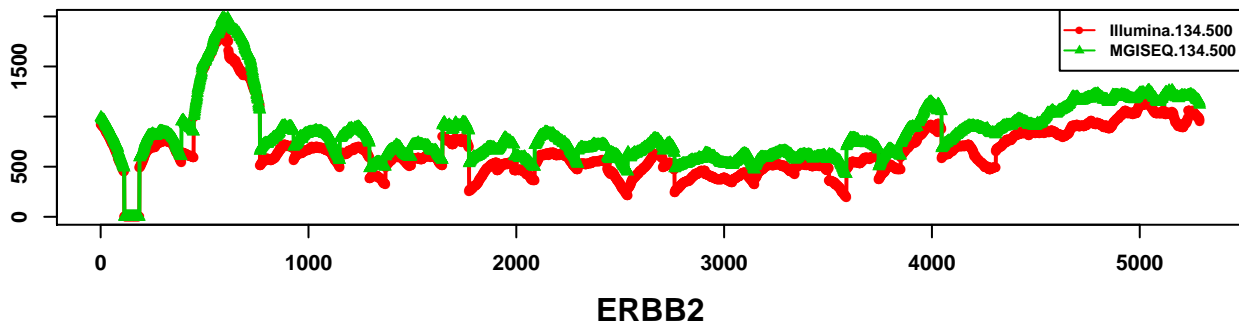

Supplement: Supplementary file 8 [file Presentation8.zip › ERBB2/19HE22008F.pdf]

Sequencing Depth

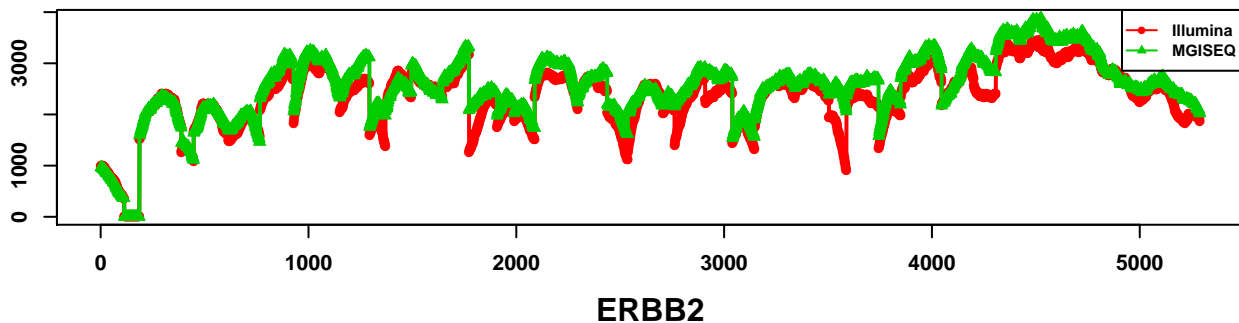

Sequencing Depth

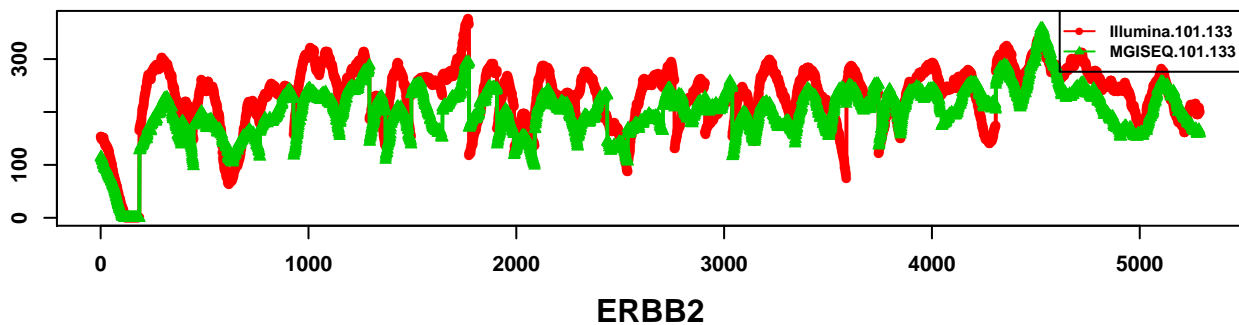

Sequencing Depth

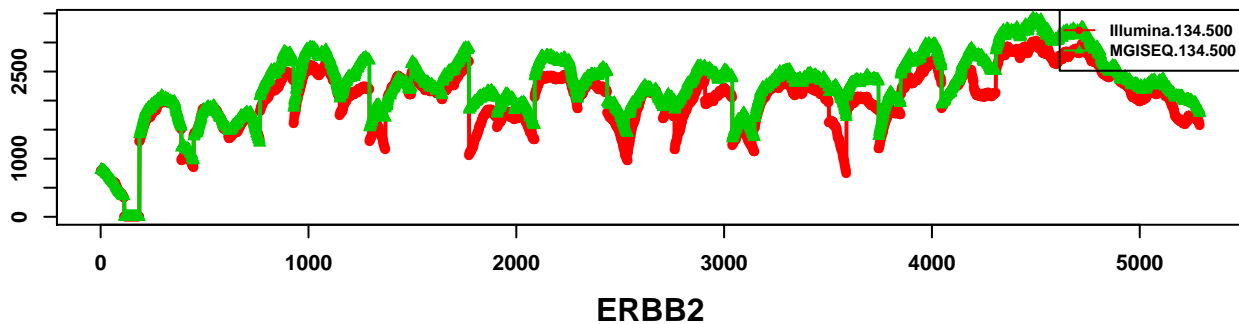

Supplement: Supplementary file 8 [file Presentation8.zip › ERBB2/19Q06147F.pdf]

Sequencing Depth

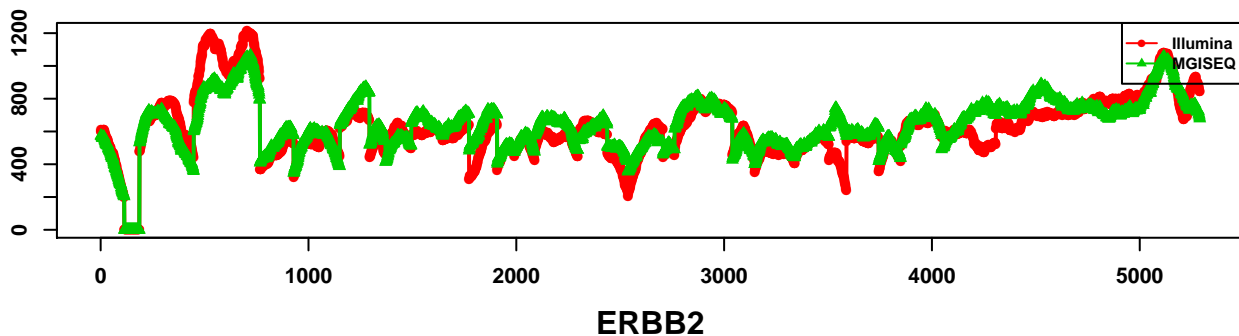

Sequencing Depth

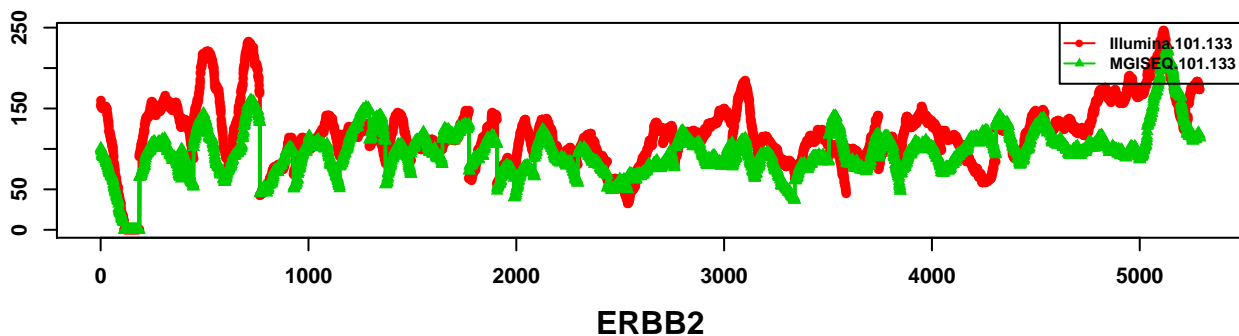

Sequencing Depth

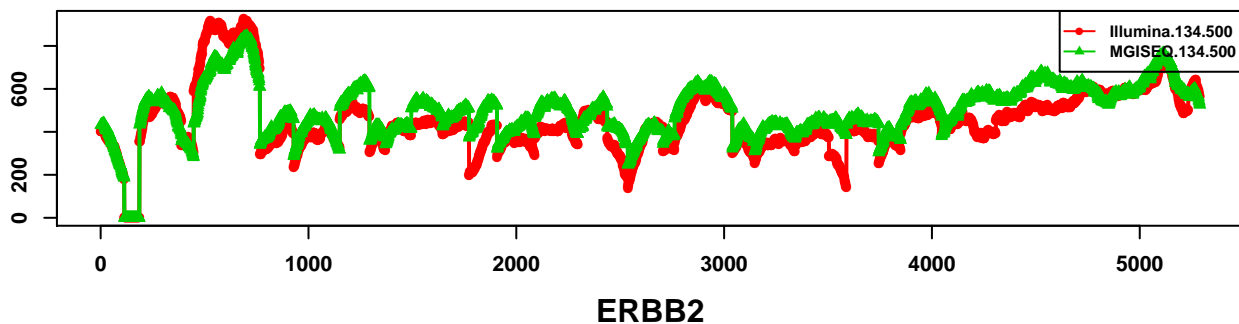

Supplement: Supplementary file 8 [file Presentation8.zip › ERBB2/19ZN13675F.pdf]

Sequencing Depth

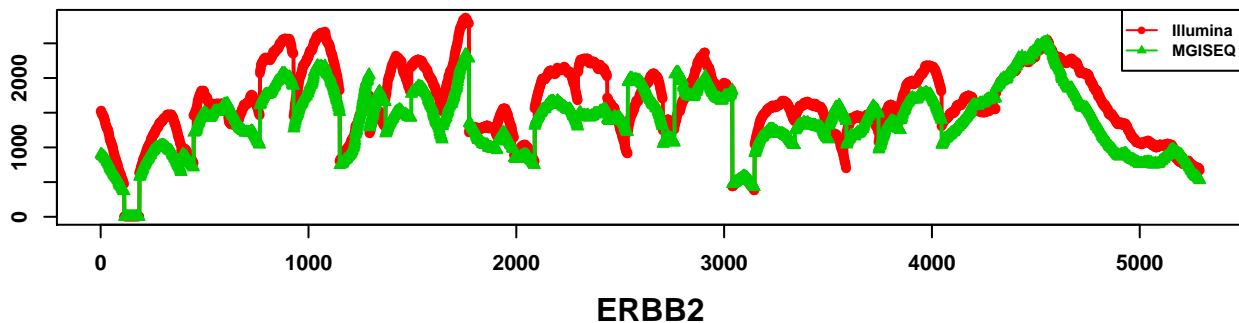

Sequencing Depth

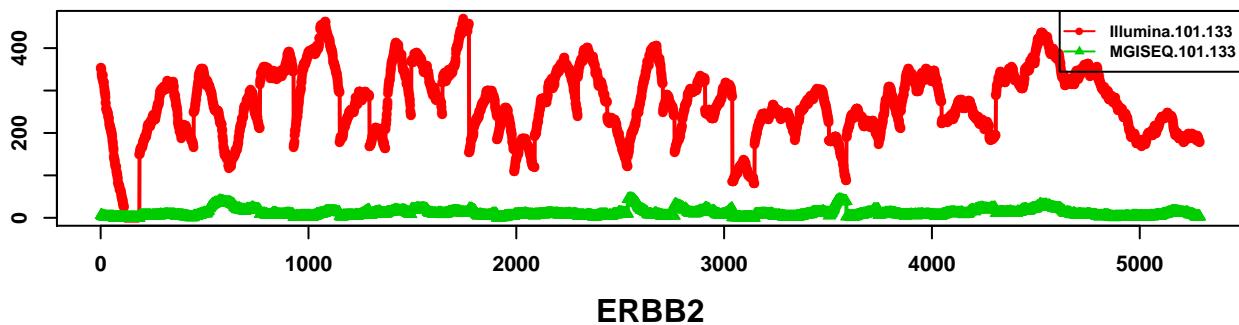

Sequencing Depth

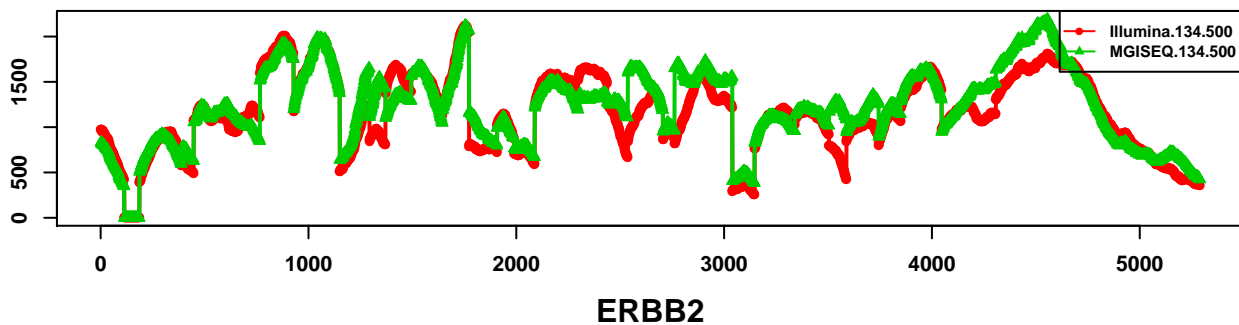

Supplement: Supplementary file 8 [file Presentation8.zip › ERBB2/19N01671T.pdf]

Sequencing Depth

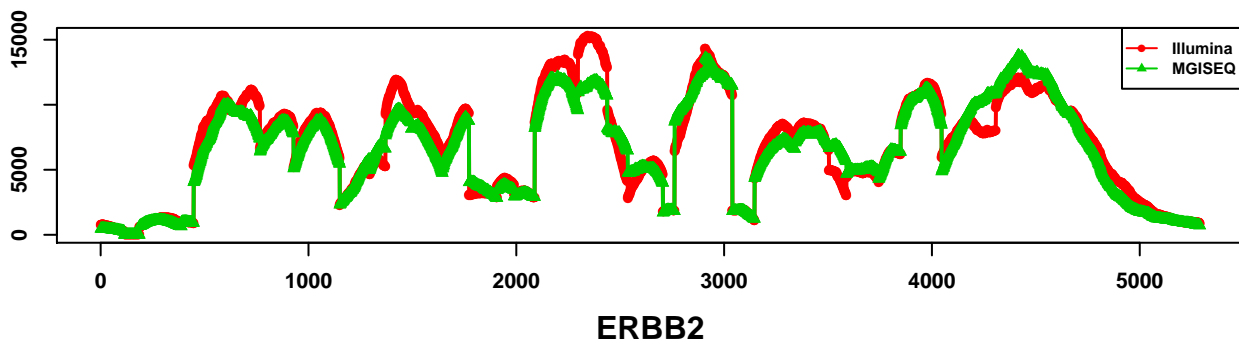

Sequencing Depth

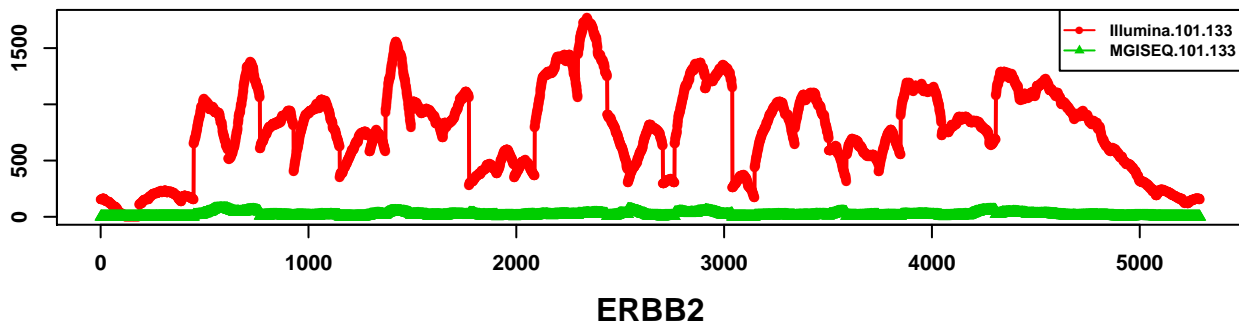

Sequencing Depth

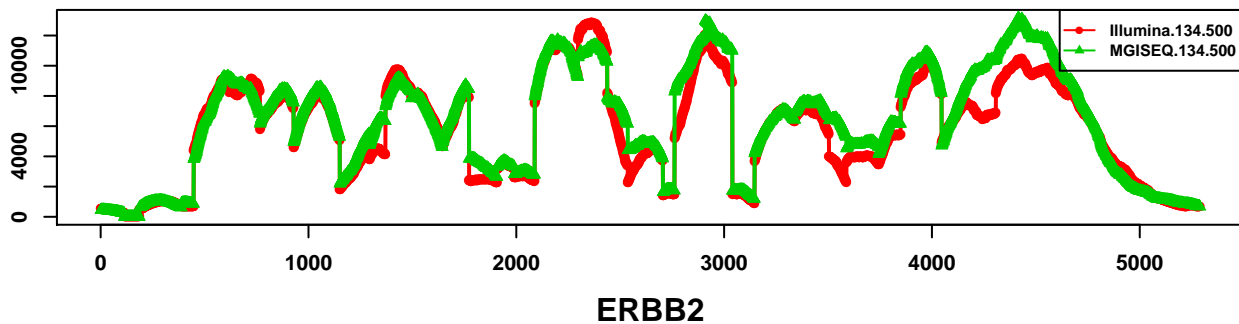

Supplement: Supplementary file 8 [file Presentation8.zip › ERBB2/19N01467QC.pdf]

Sequencing Depth

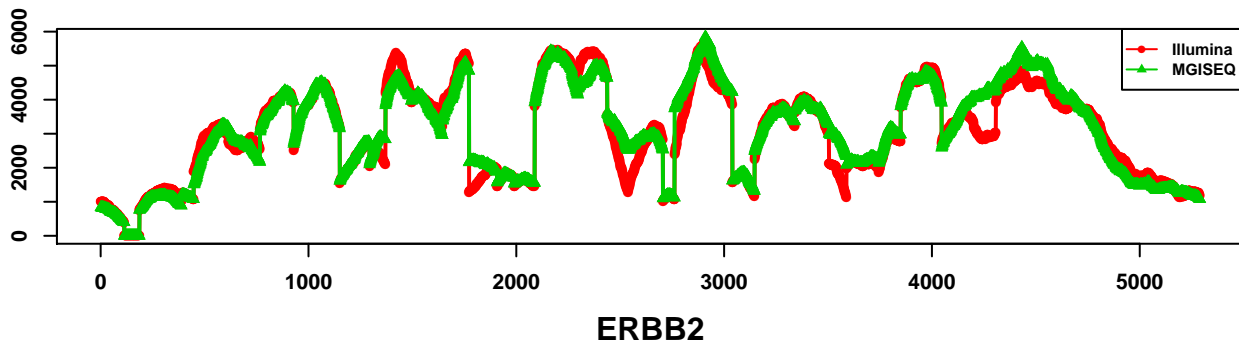

Sequencing Depth

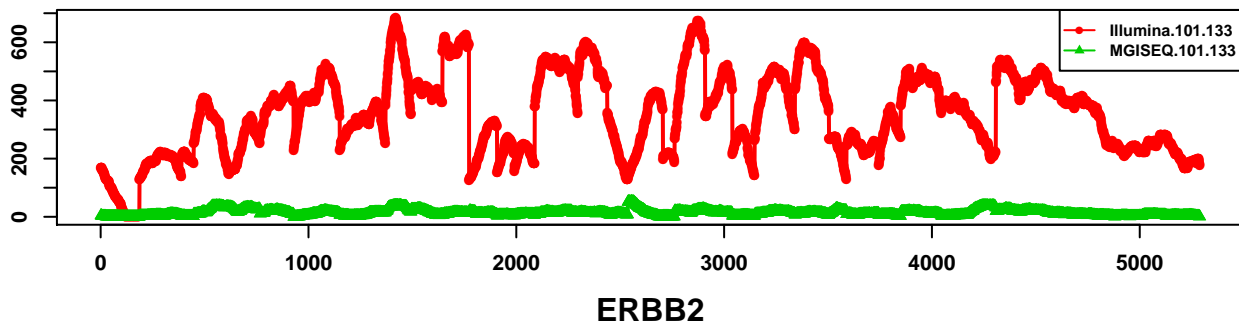

Sequencing Depth

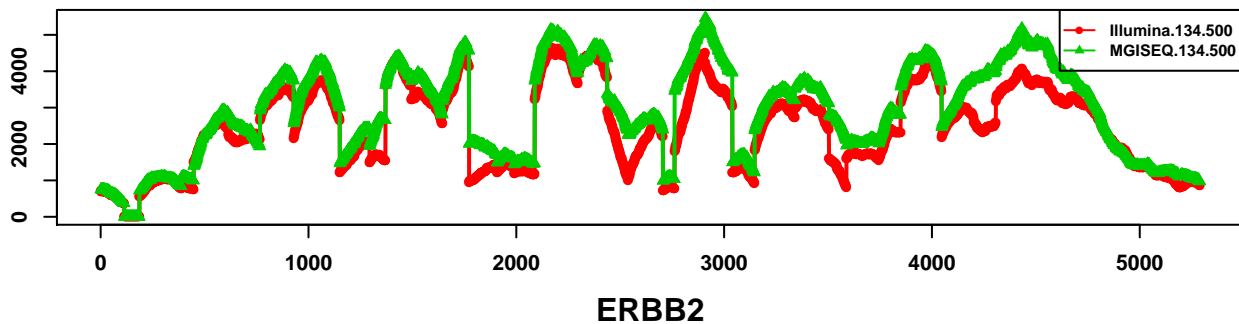

Supplement: Supplementary file 8 [file Presentation8.zip › ERBB2/19FC40250F.pdf]

Sequencing Depth

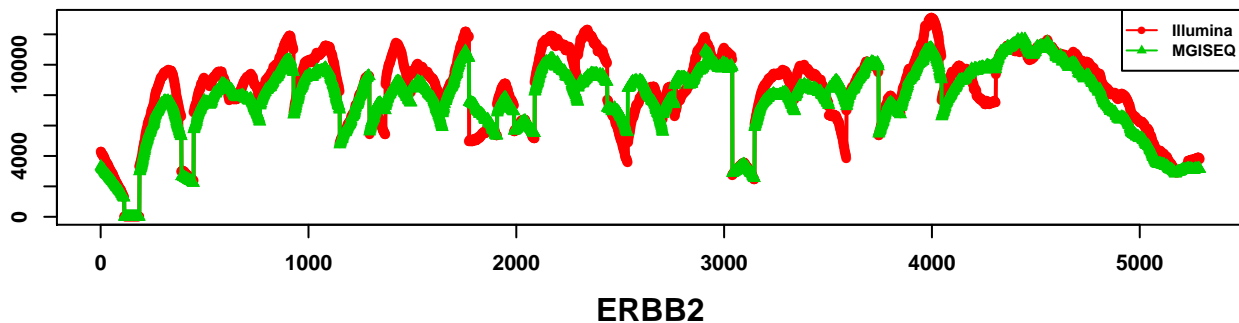

Sequencing Depth

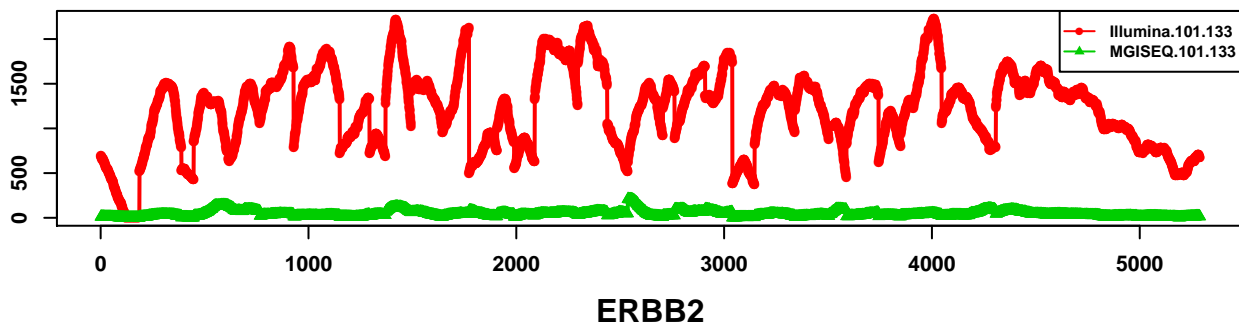

Sequencing Depth

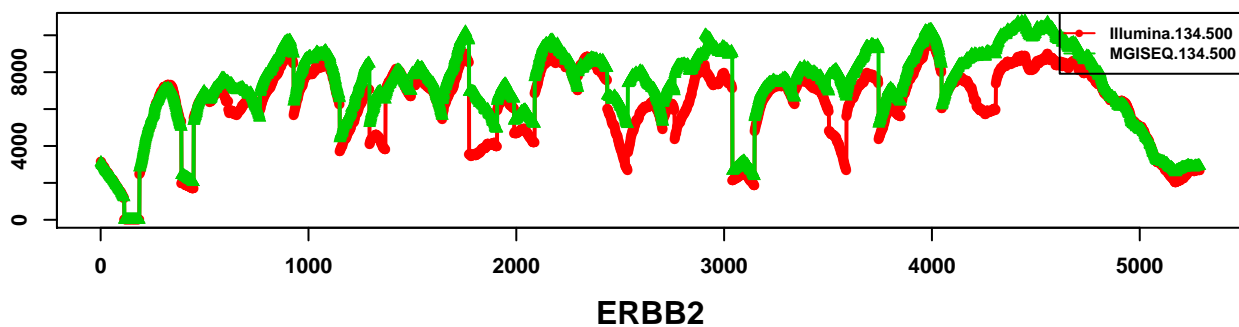

Supplement: Supplementary file 8 [file Presentation8.zip › ERBB2/19HE22120F.pdf]

Sequencing Depth

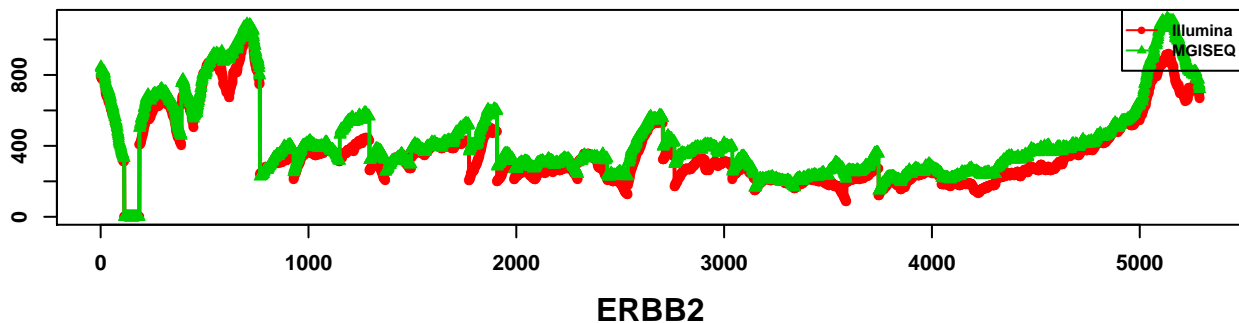

Sequencing Depth

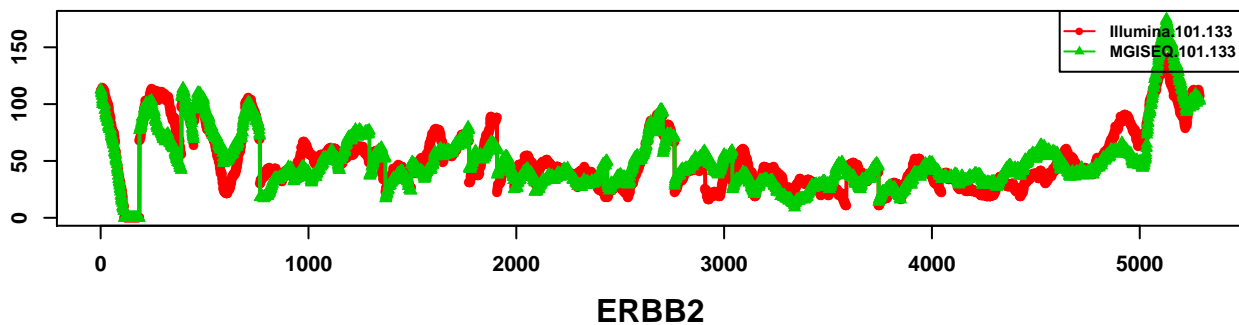

Sequencing Depth

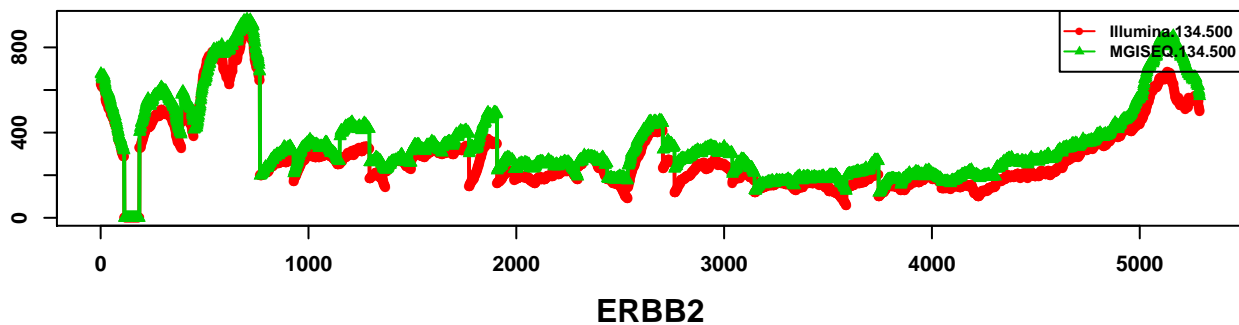

Supplement: Supplementary file 8 [file Presentation8.zip › ERBB2/19N01373F.pdf]

Sequencing Depth

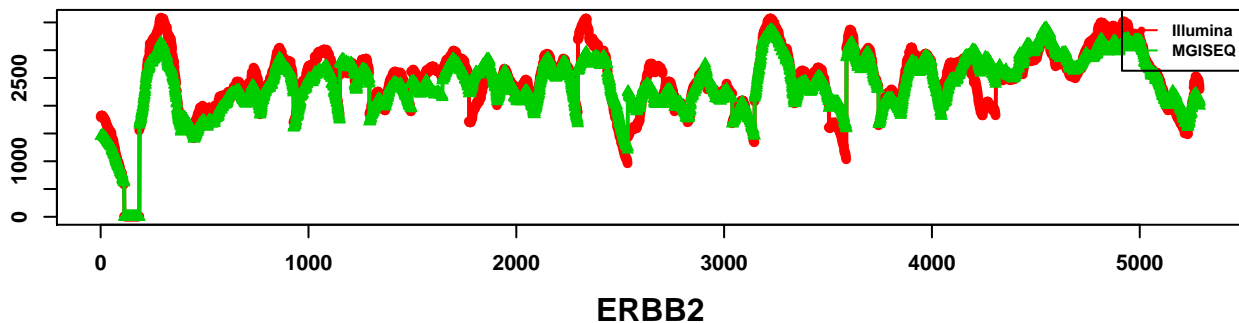

Sequencing Depth

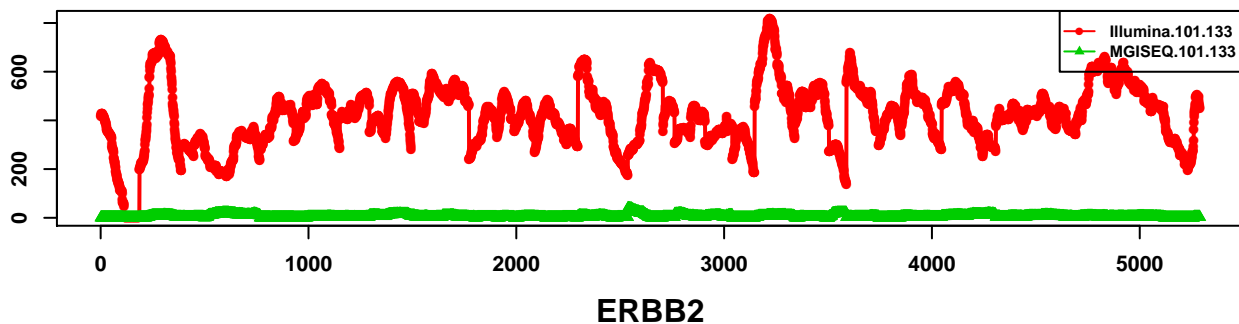

Sequencing Depth

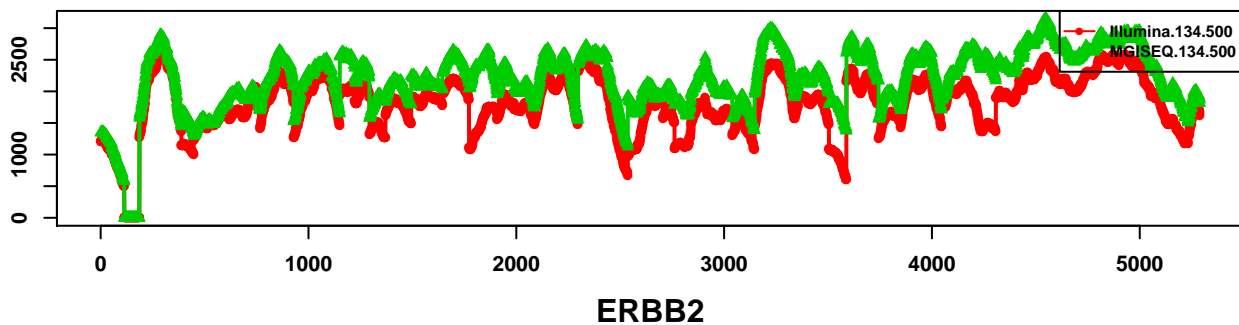

Supplement: Supplementary file 8 [file Presentation8.zip › ERBB2/19HE22225P.pdf]

Sequencing Depth

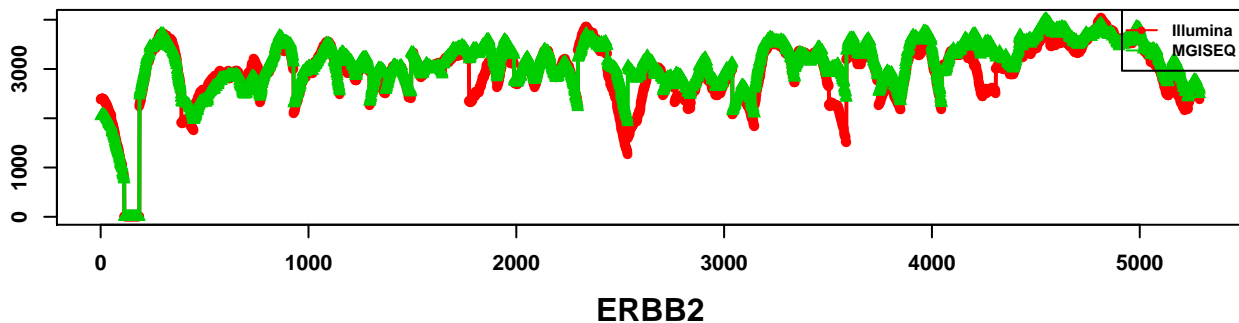

Sequencing Depth

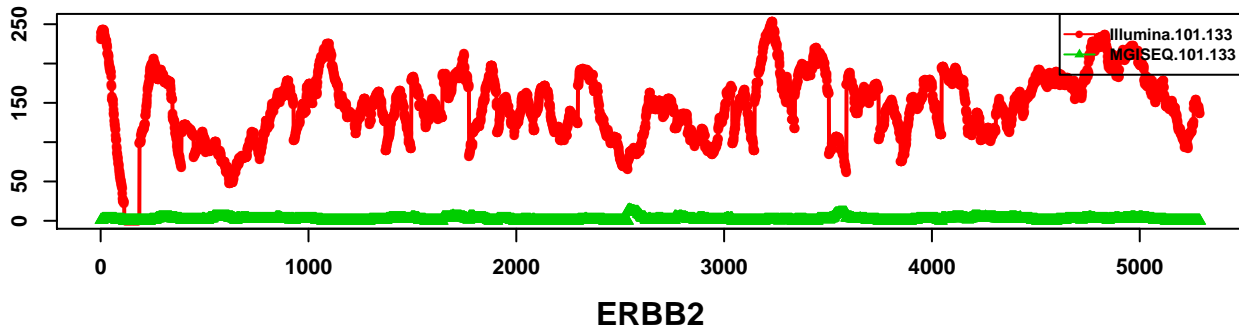

Sequencing Depth

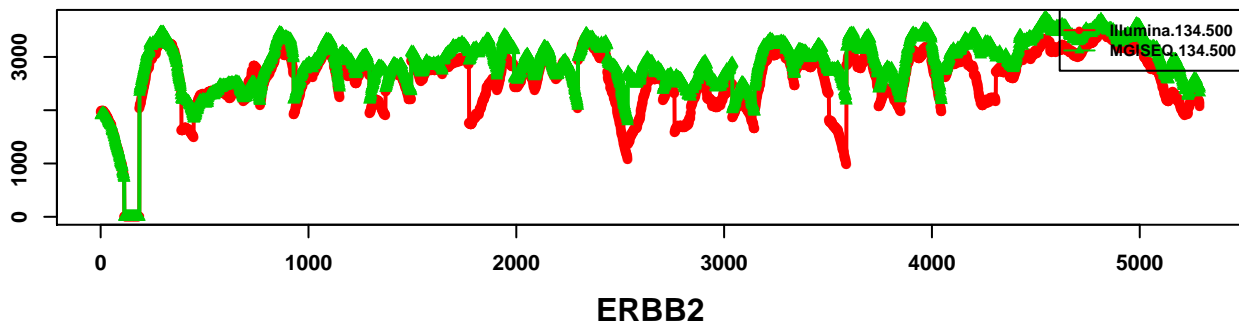

Supplement: Supplementary file 8 [file Presentation8.zip › ERBB2/19YT52688P.pdf]

Sequencing Depth

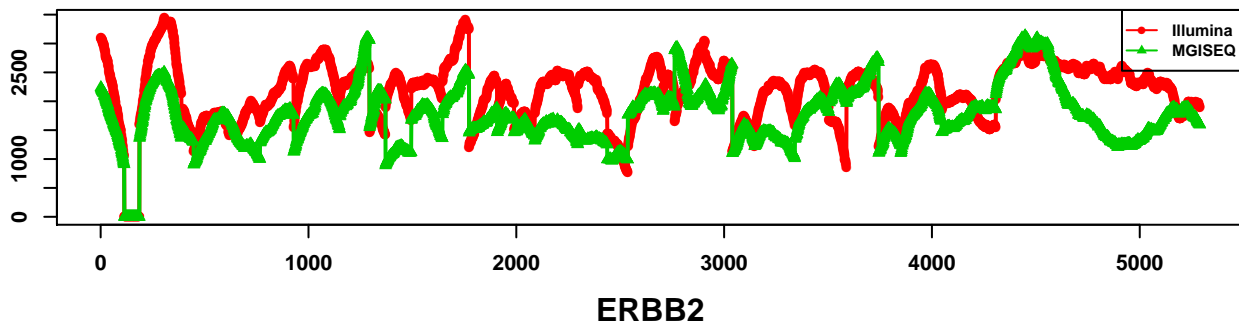

Sequencing Depth

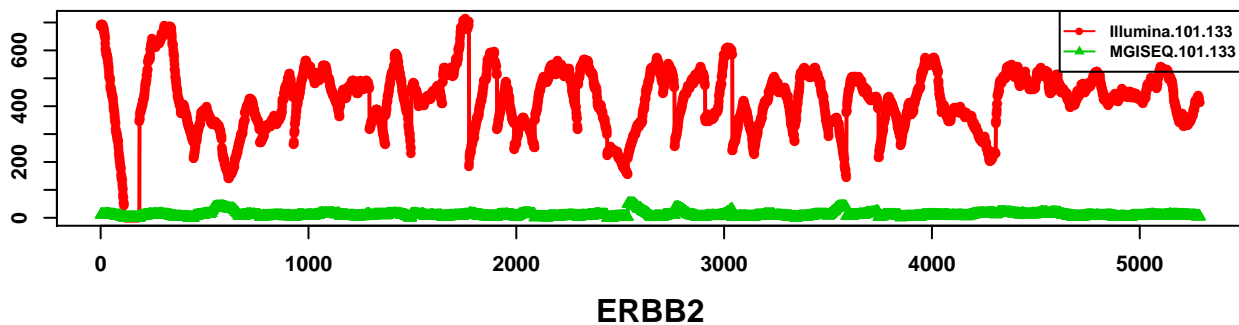

Sequencing Depth

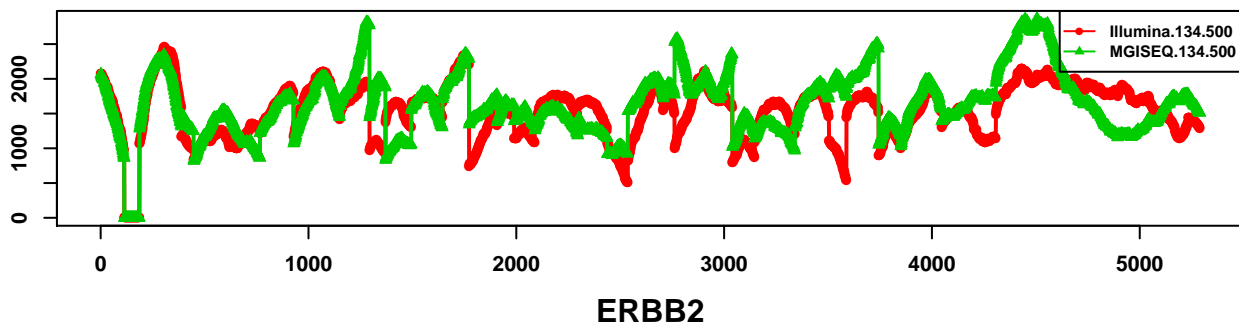

Supplement: Supplementary file 8 [file Presentation8.zip › ERBB2/19N01667F.pdf]

Sequencing Depth

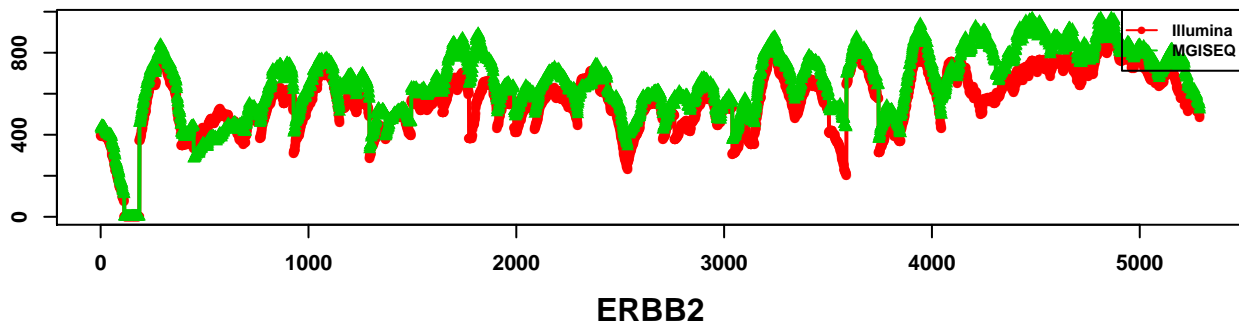

Sequencing Depth

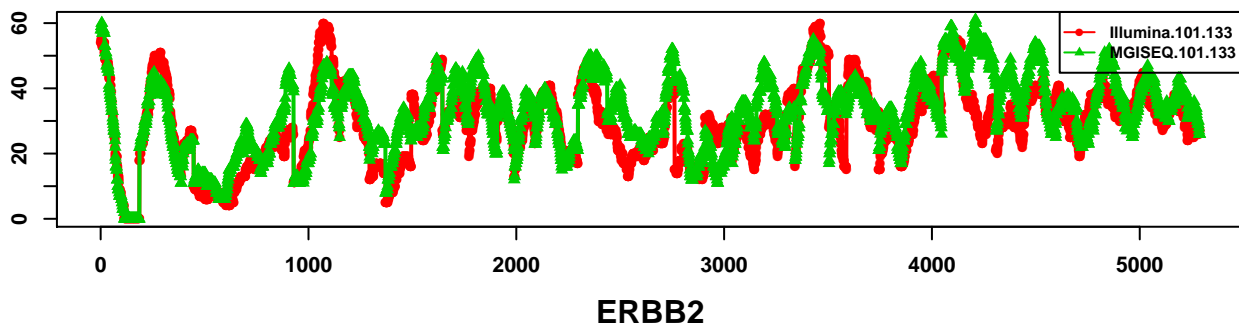

Sequencing Depth

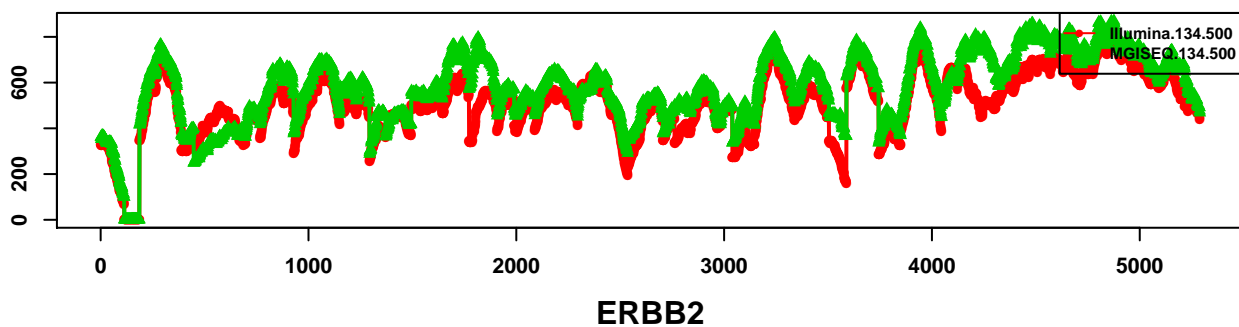

Supplement: Supplementary file 8 [file Presentation8.zip › ERBB2/19ZN12531P.pdf]

Sequencing Depth

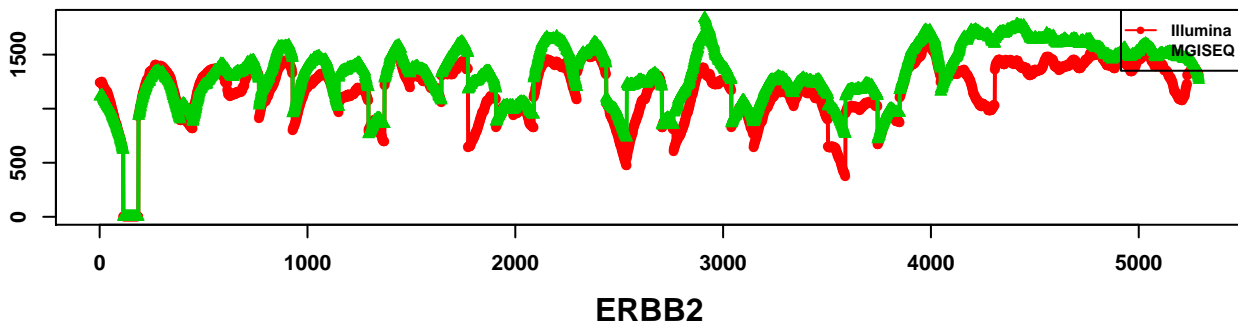

Sequencing Depth

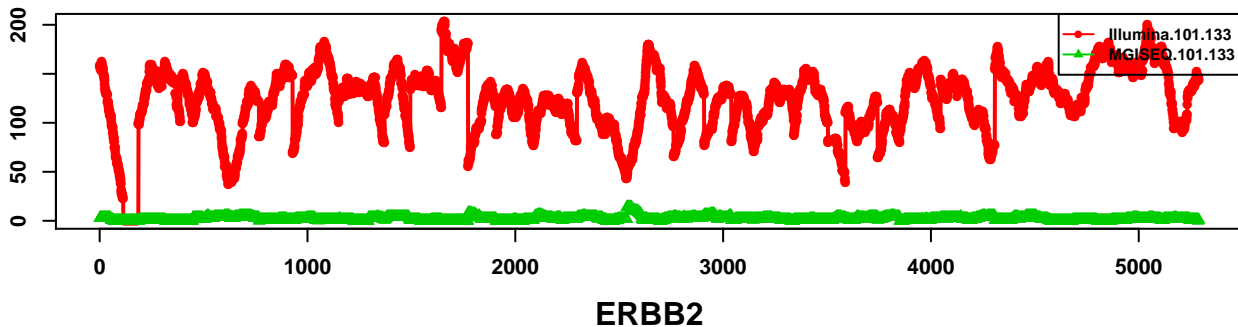

Sequencing Depth

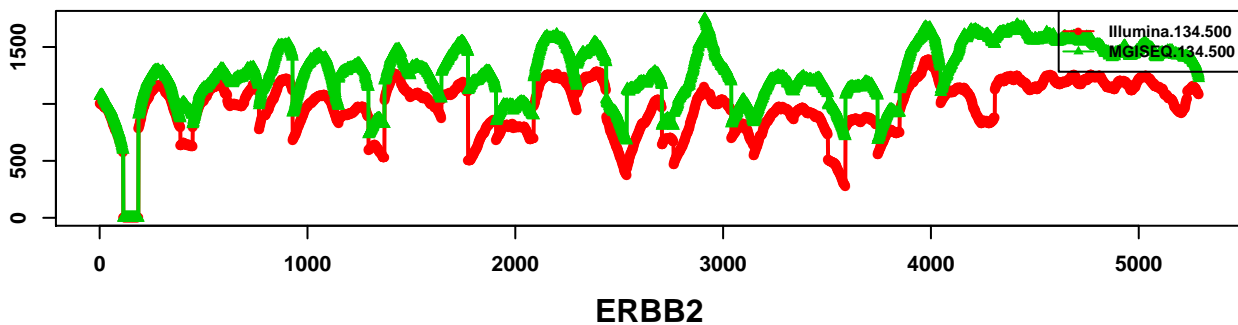

Supplement: Supplementary file 8 [file Presentation8.zip › ERBB2/19HE21977F.pdf]

Sequencing Depth

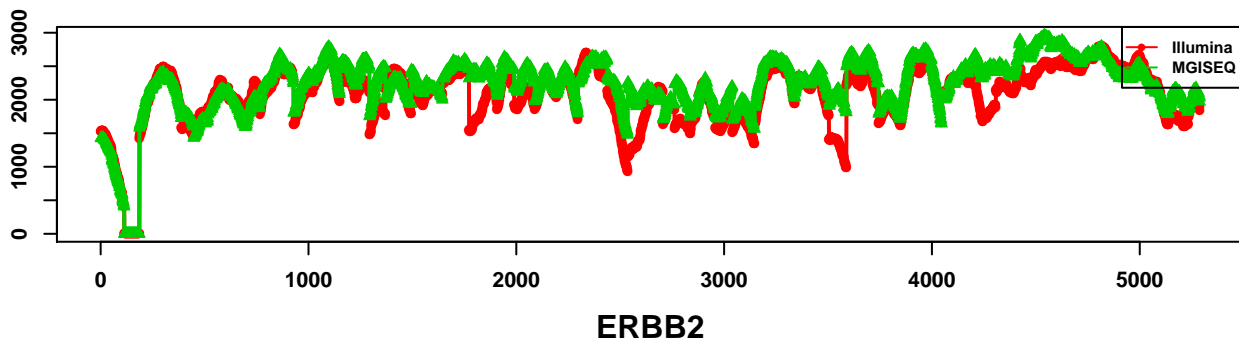

Sequencing Depth

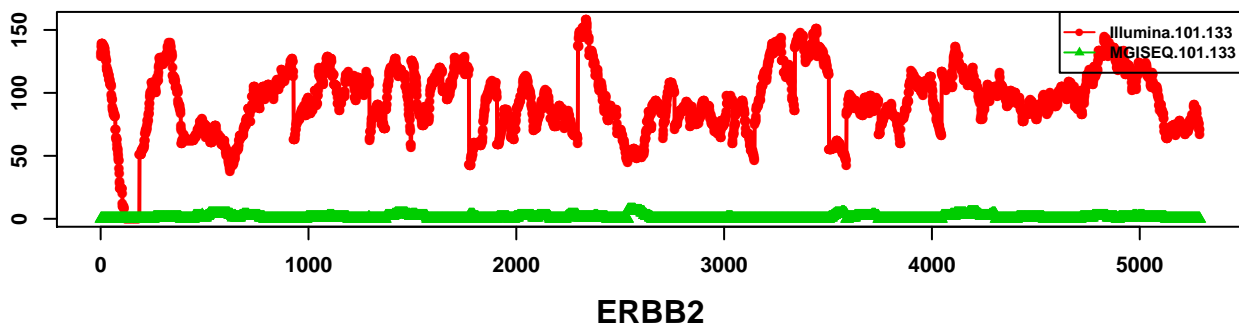

Sequencing Depth

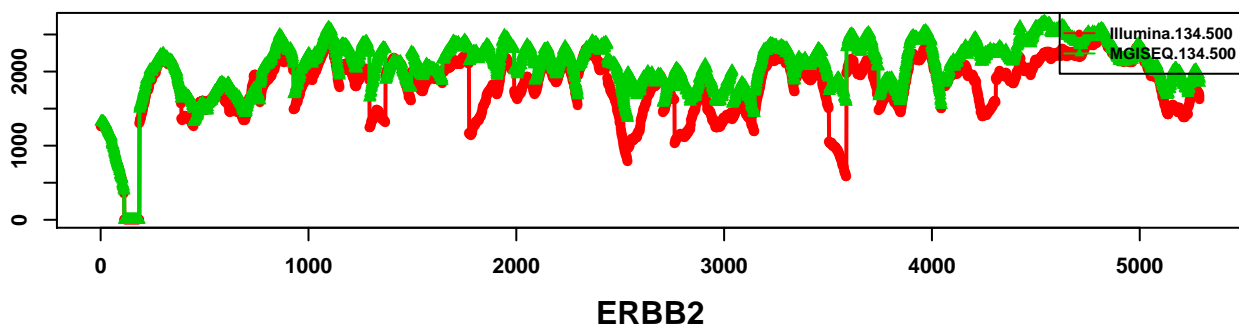

Supplement: Supplementary file 8 [file Presentation8.zip › ERBB2/19ZN10625P.pdf]

Sequencing Depth

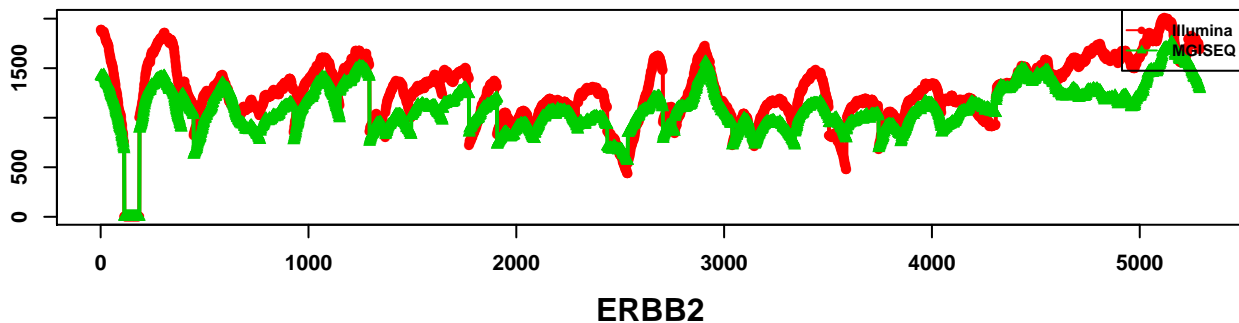

Sequencing Depth

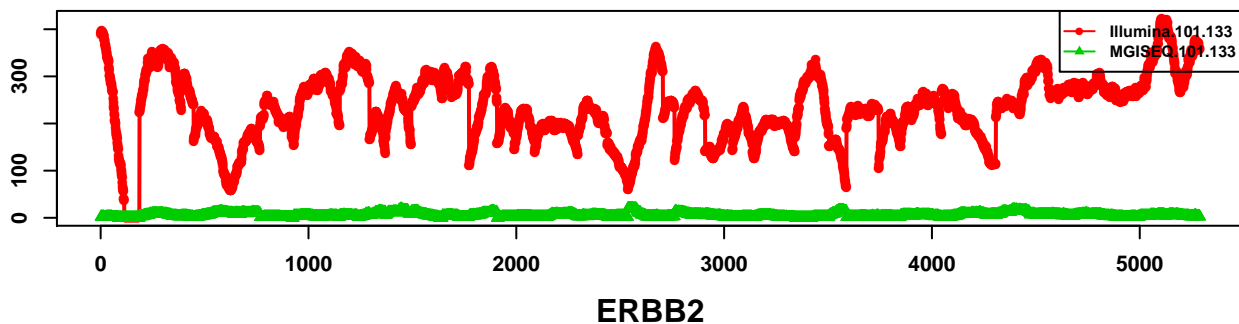

Sequencing Depth

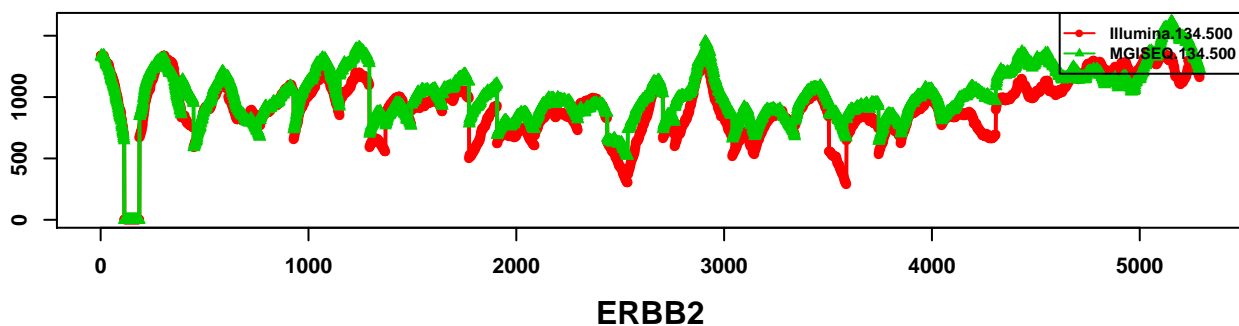

Supplement: Supplementary file 8 [file Presentation8.zip › ERBB2/19N01626F.pdf]

Sequencing Depth

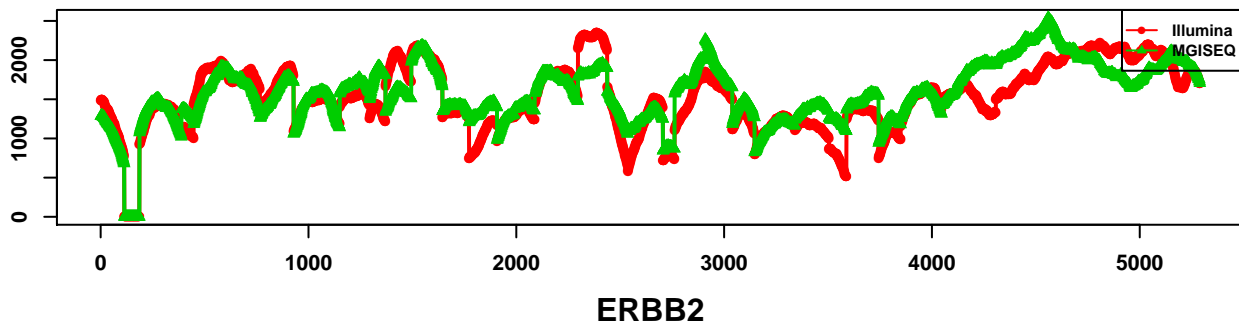

Sequencing Depth

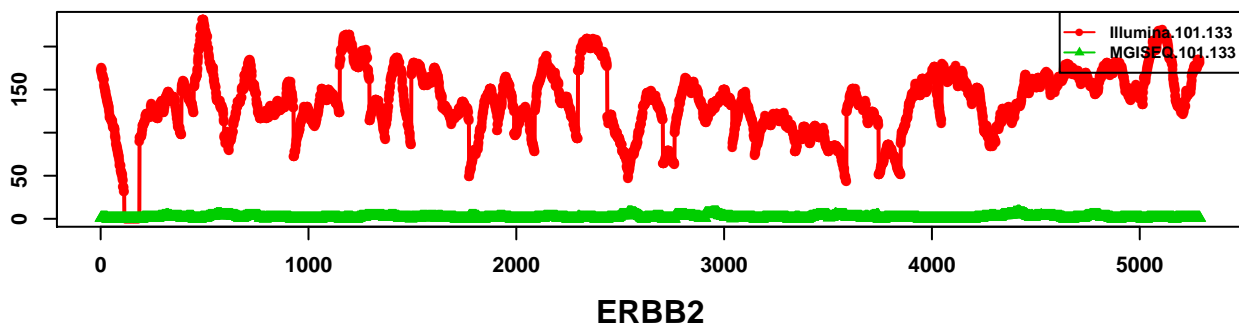

Sequencing Depth

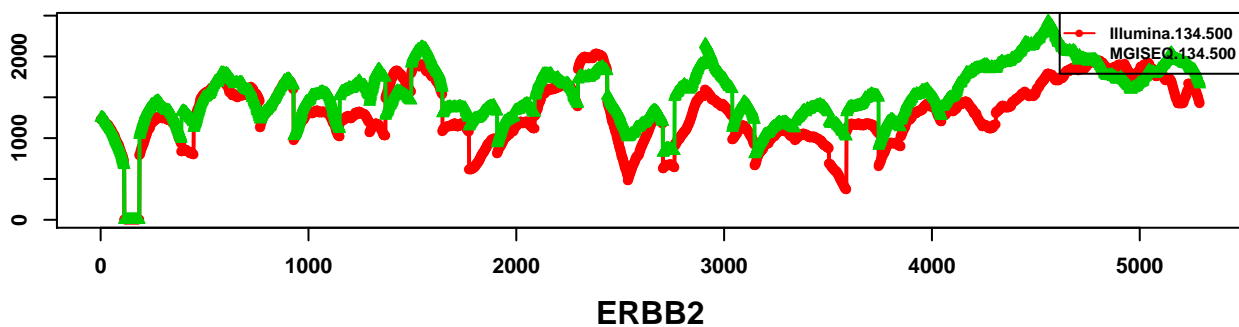

Supplement: Supplementary file 8 [file Presentation8.zip › ERBB2/19N01567QC.pdf]

Sequencing Depth

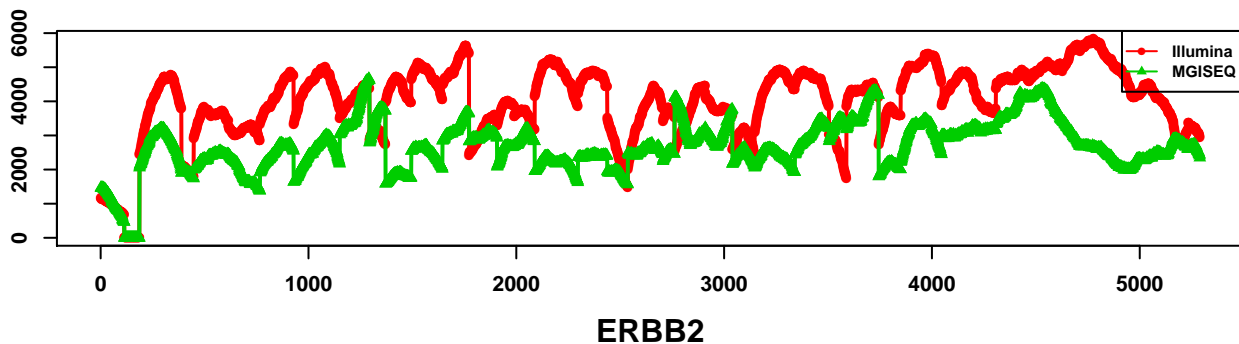

Sequencing Depth

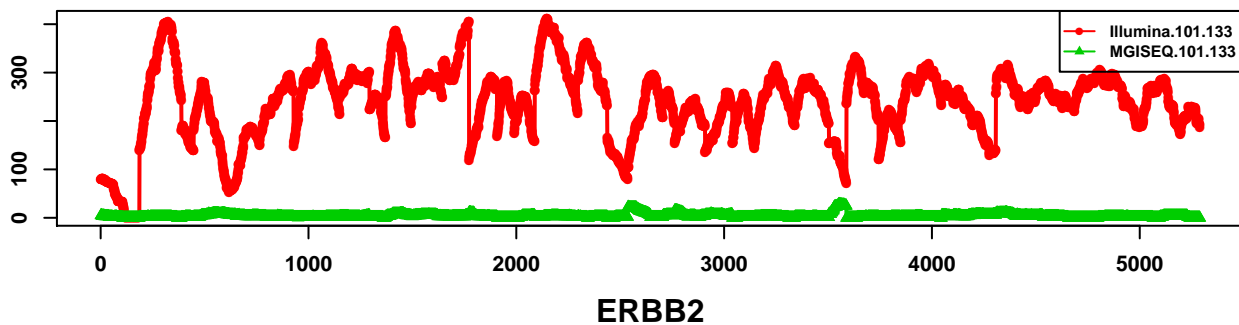

Sequencing Depth

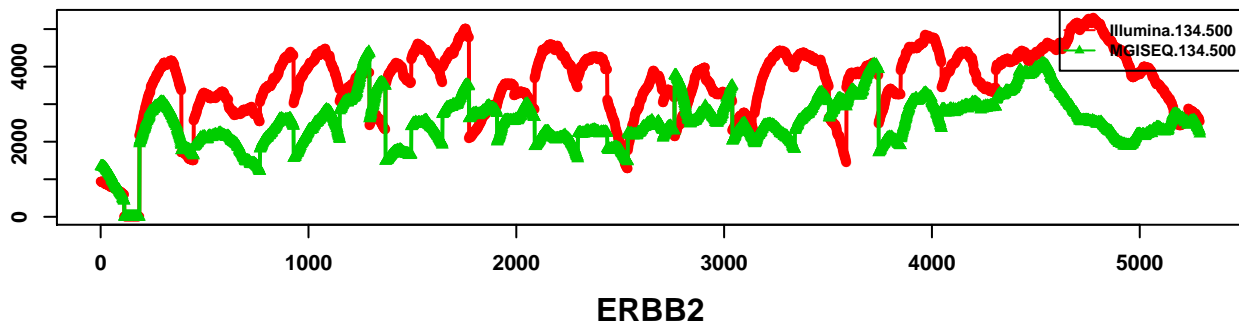

Supplement: Supplementary file 8 [file Presentation8.zip › ERBB2/19N01670T.pdf]

Sequencing Depth

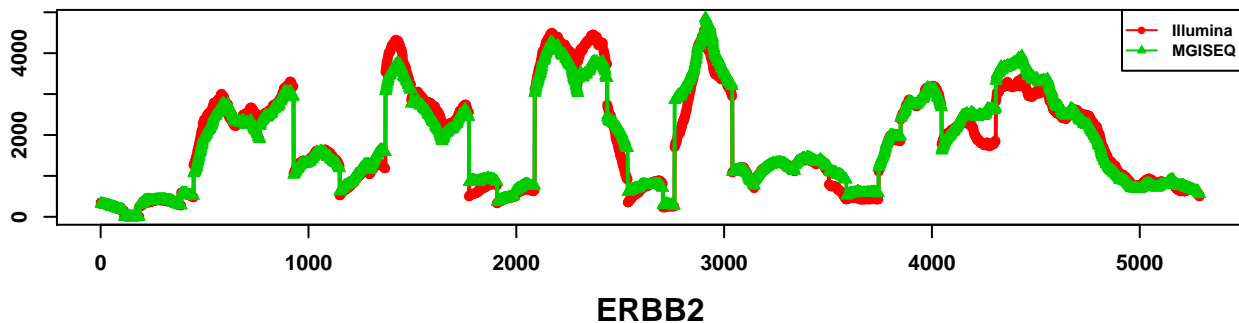

Sequencing Depth

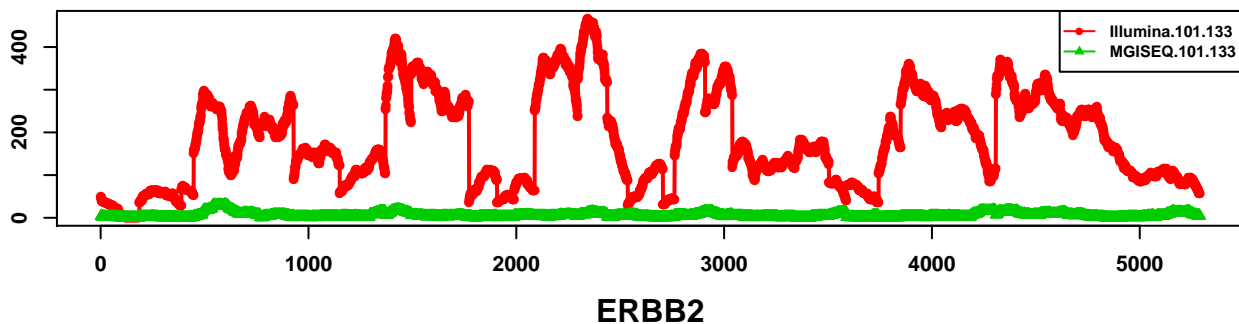

Sequencing Depth

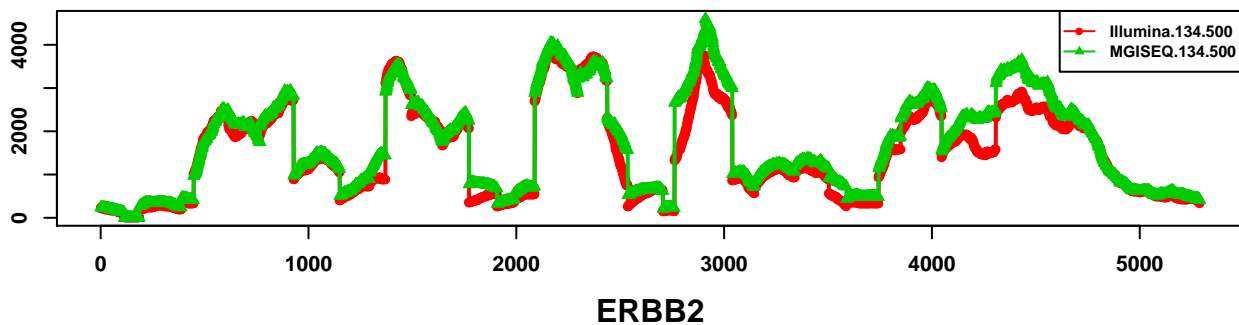

Supplement: Supplementary file 8 [file Presentation8.zip › ERBB2/19FC40251F.pdf]

Sequencing Depth

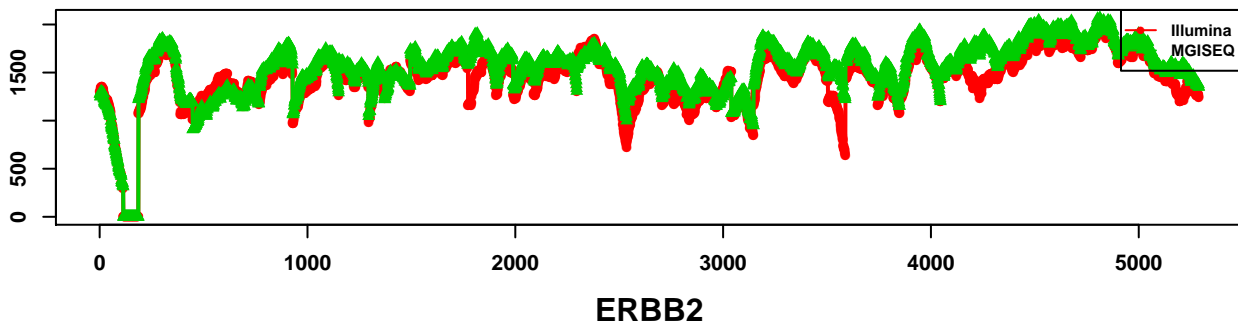

Sequencing Depth

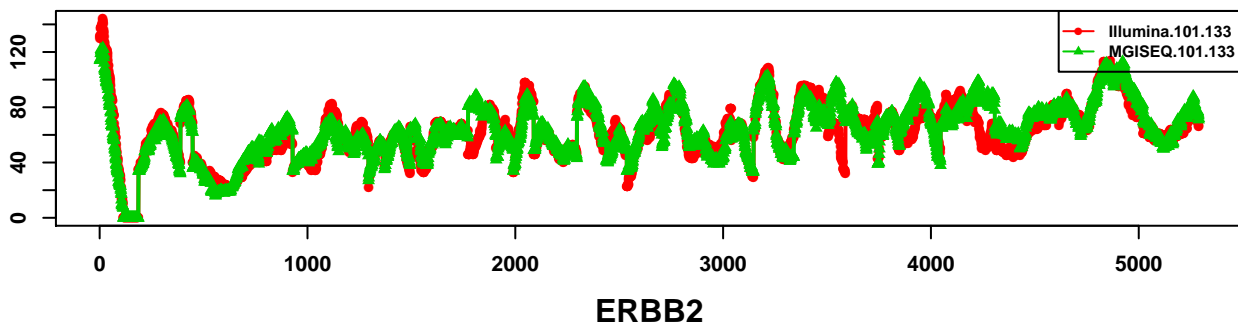

Sequencing Depth

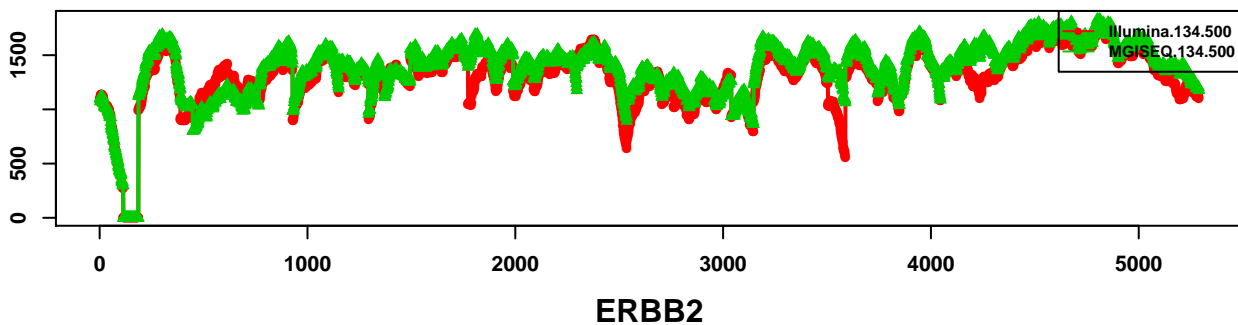

Supplement: Supplementary file 8 [file Presentation8.zip › ERBB2/19BZ58024P.pdf]

Sequencing Depth

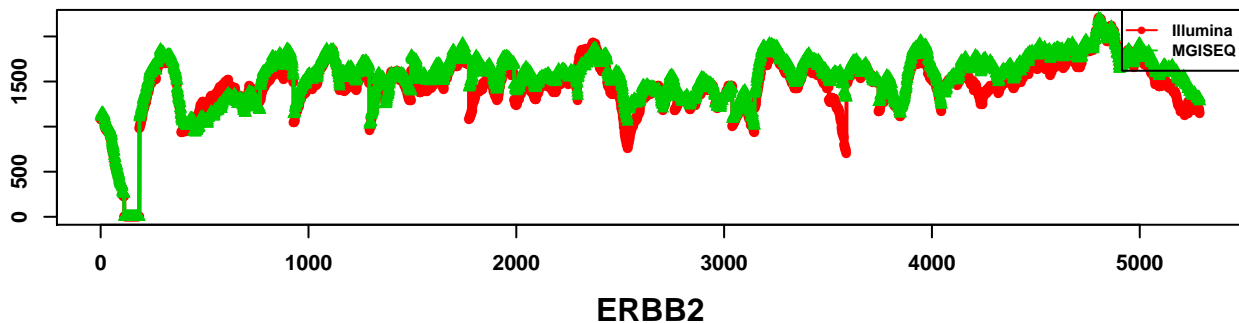

Sequencing Depth

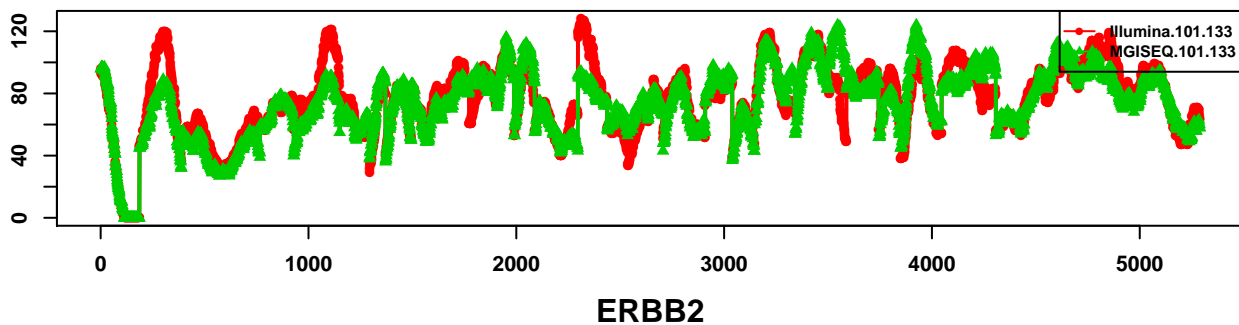

Sequencing Depth

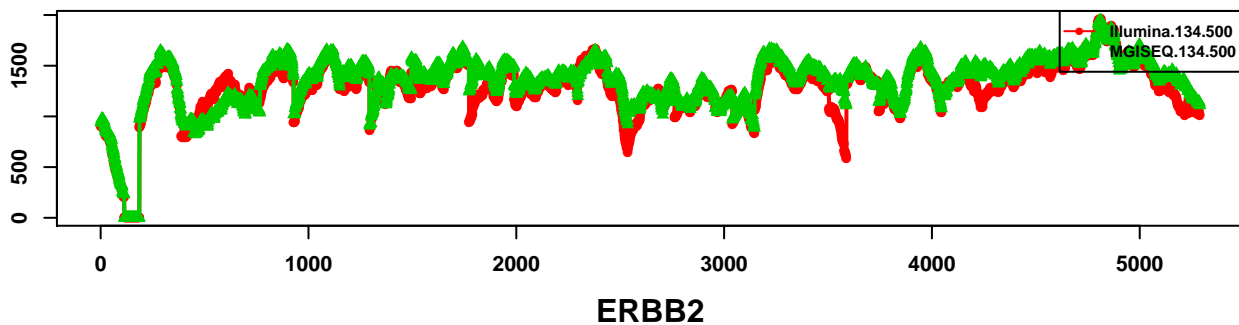

Supplement: Supplementary file 8 [file Presentation8.zip › ERBB2/19CF15710P.pdf]

Sequencing Depth

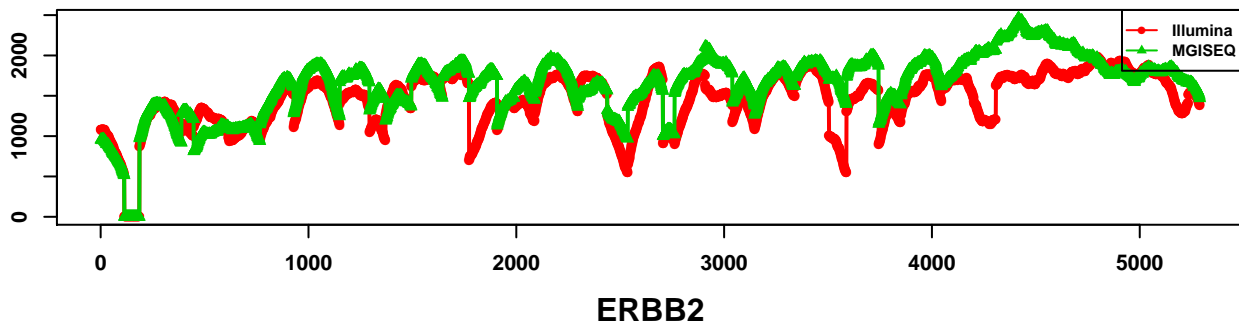

Sequencing Depth

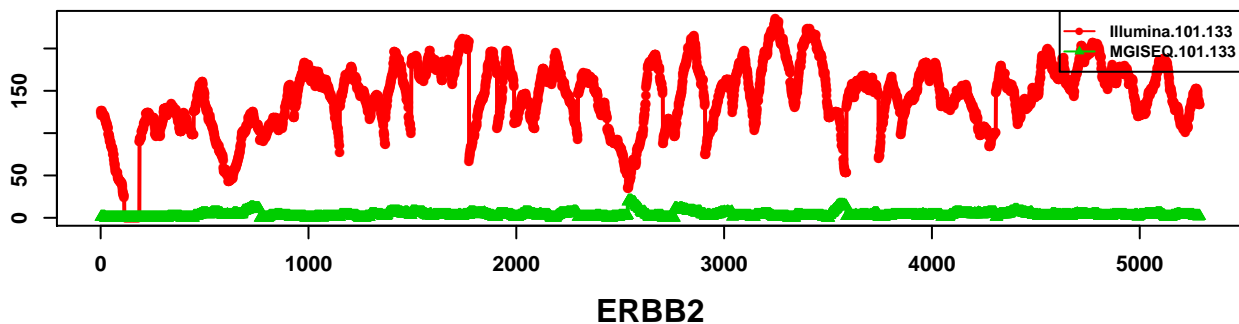

Sequencing Depth

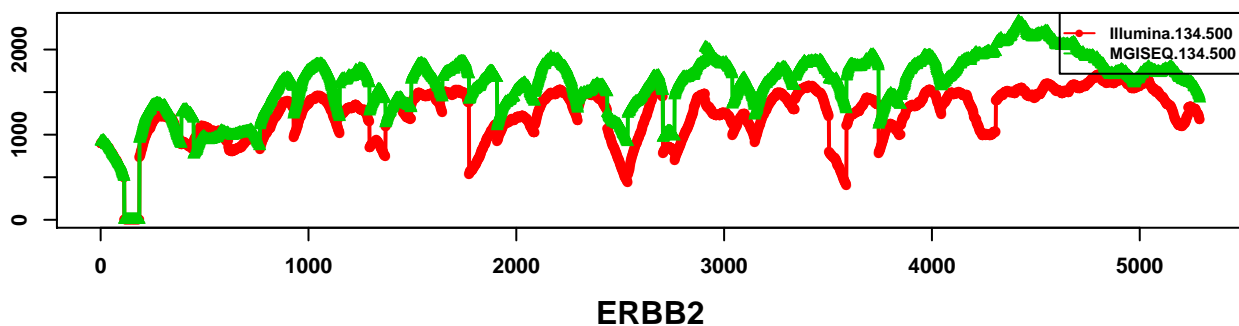

Supplement: Supplementary file 8 [file Presentation8.zip › ERBB2/19LN70341F.pdf]

Sequencing Depth

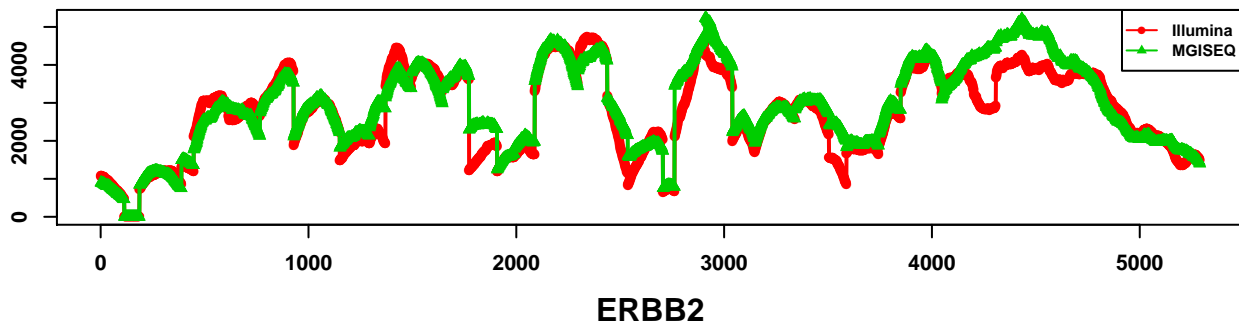

Sequencing Depth

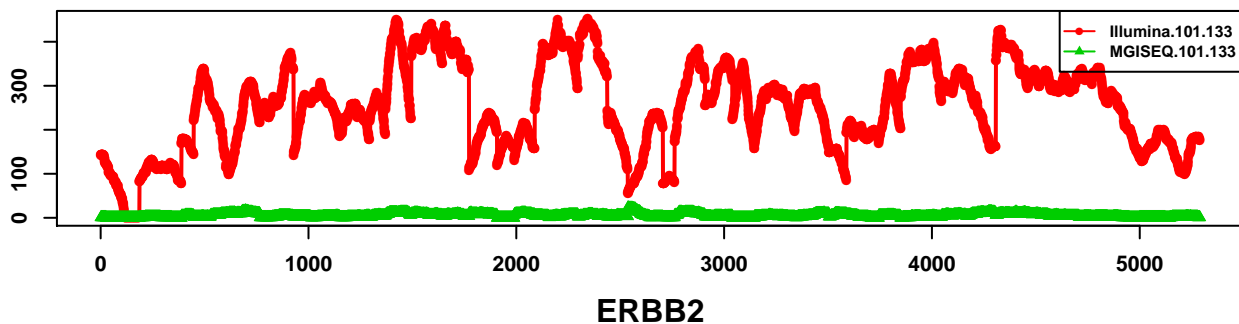

Sequencing Depth

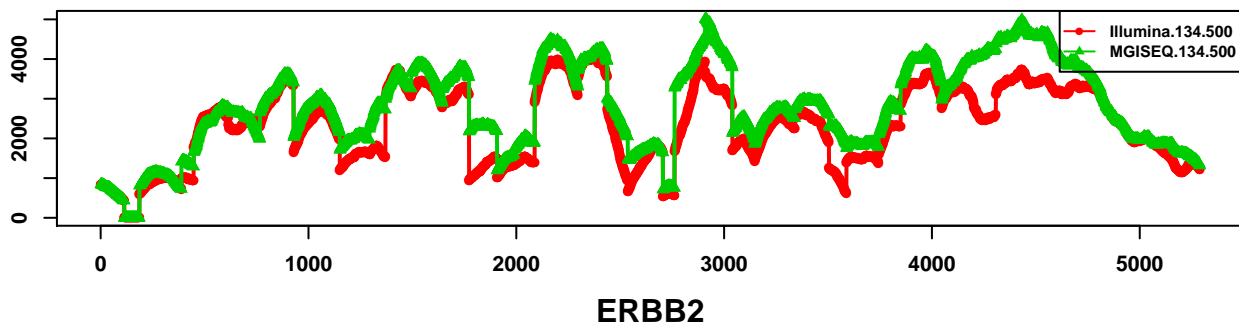

Supplement: Supplementary file 8 [file Presentation8.zip › ERBB2/19FC40247F.pdf]

Sequencing Depth

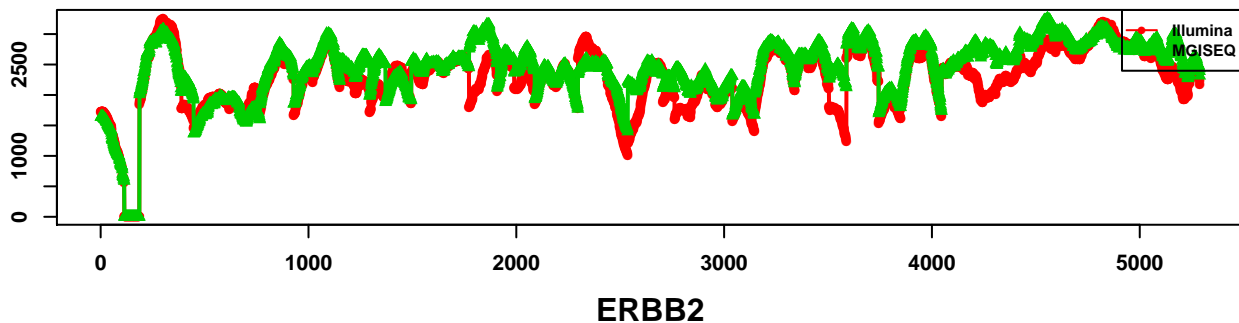

Sequencing Depth

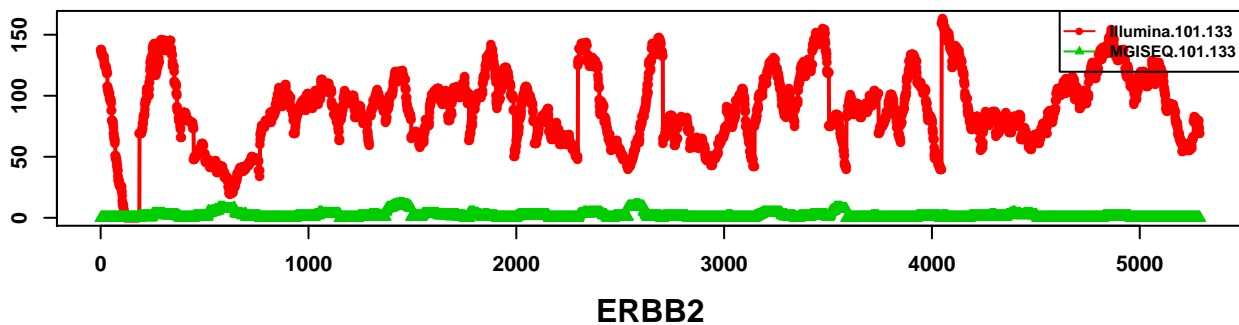

Sequencing Depth

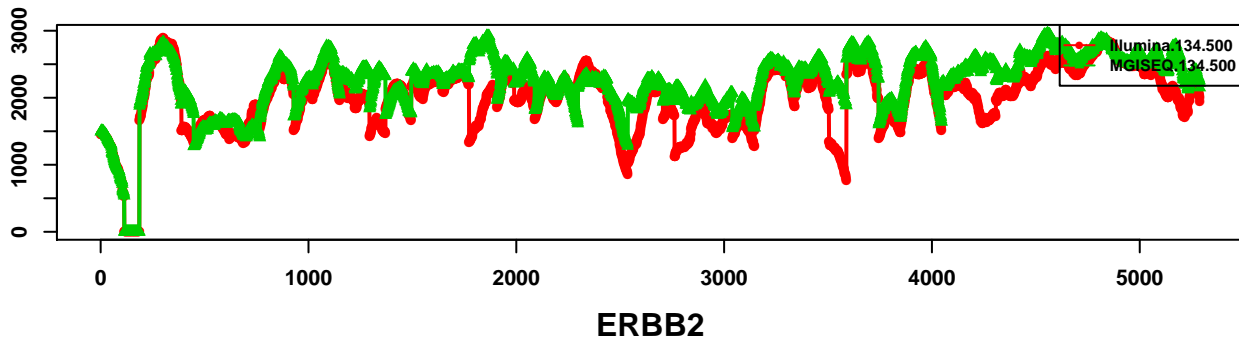

Supplement: Supplementary file 8 [file Presentation8.zip › ERBB2/19ZN12356P.pdf]

Sequencing Depth

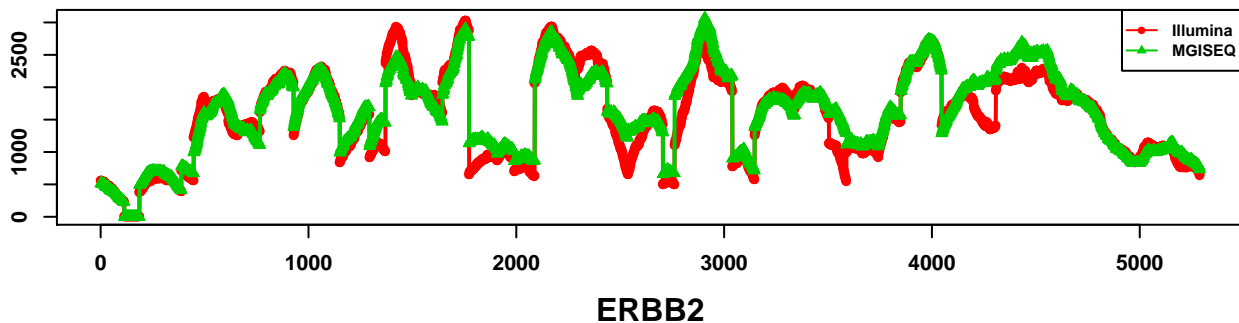

Sequencing Depth

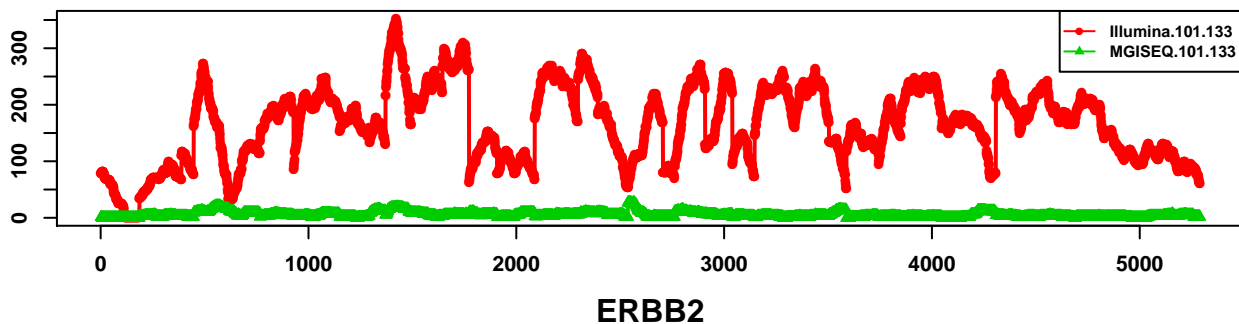

Sequencing Depth

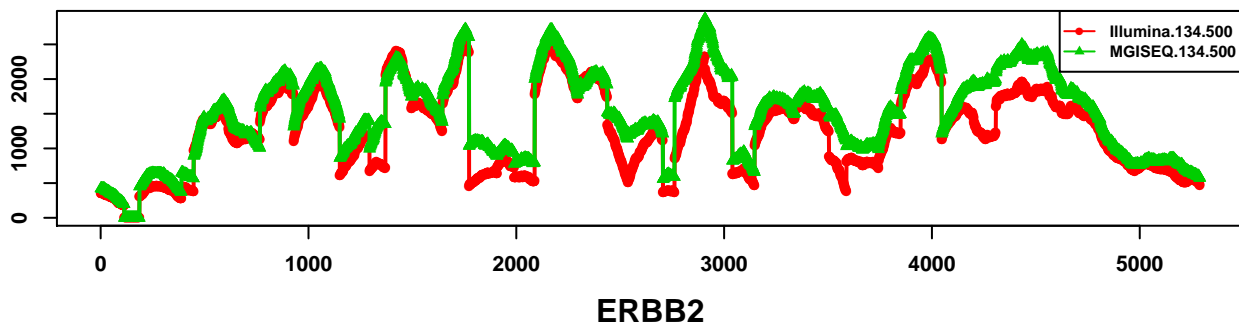

Supplement: Supplementary file 8 [file Presentation8.zip › ERBB2/19JM45577F.pdf]

Sequencing Depth

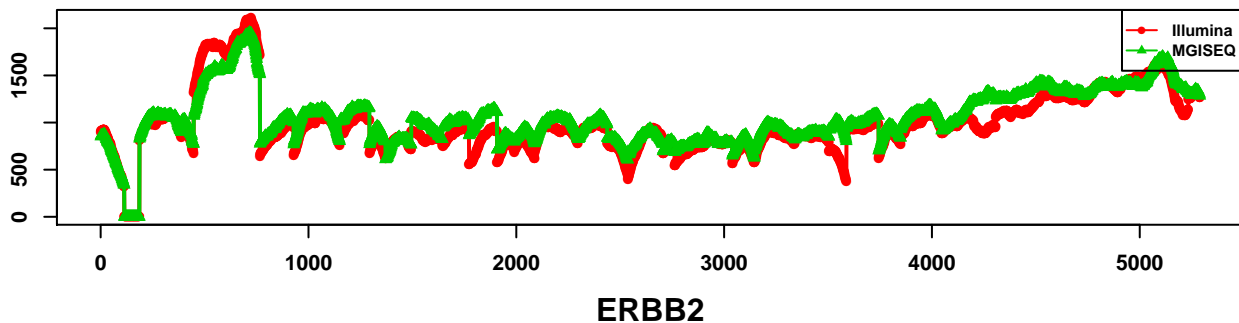

Sequencing Depth

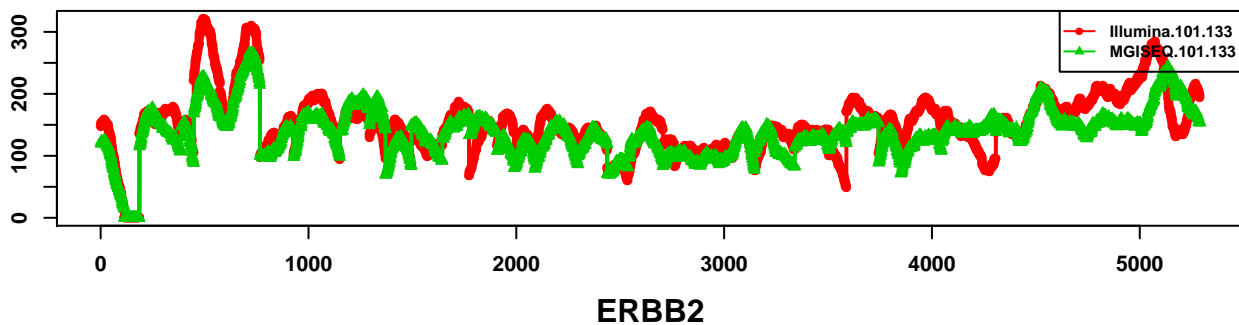

Sequencing Depth

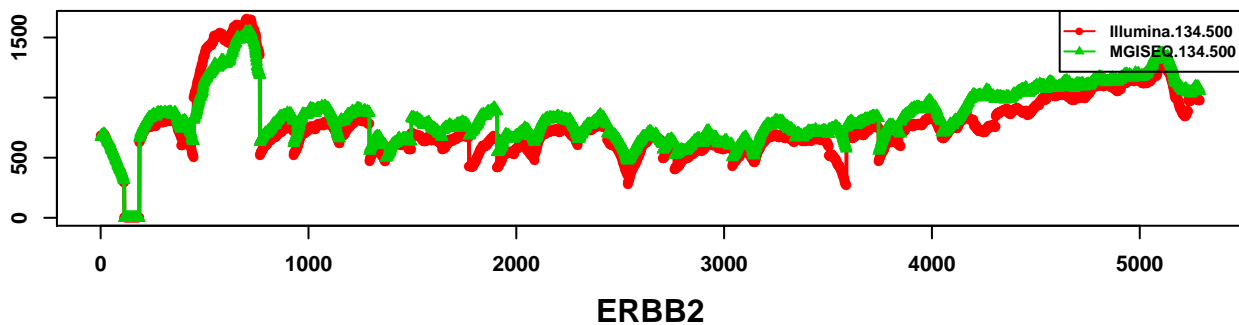

Supplement: Supplementary file 8 [file Presentation8.zip › ERBB2/19ZN12546F.pdf]

Sequencing Depth

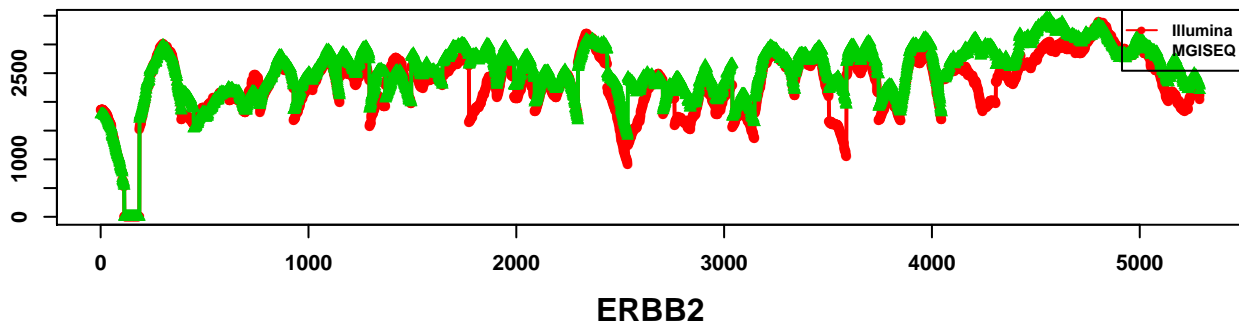

Sequencing Depth

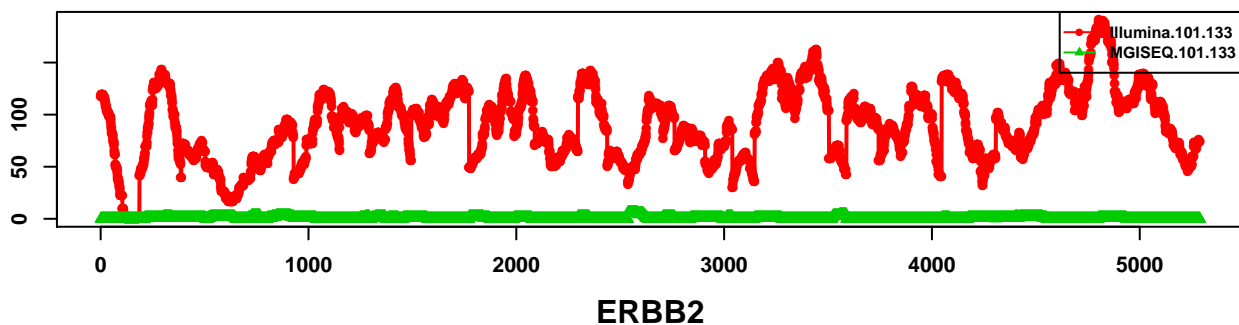

Sequencing Depth

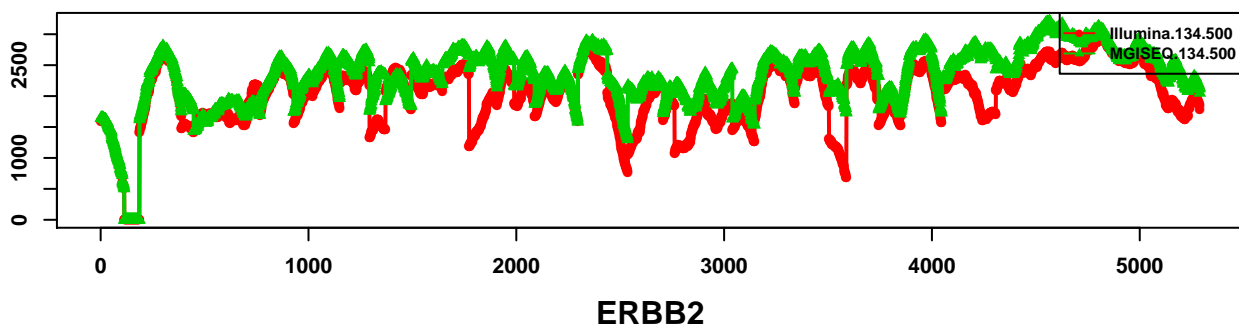

Supplement: Supplementary file 8 [file Presentation8.zip › ERBB2/M1900799-I-IIP.pdf]

Sequencing Depth

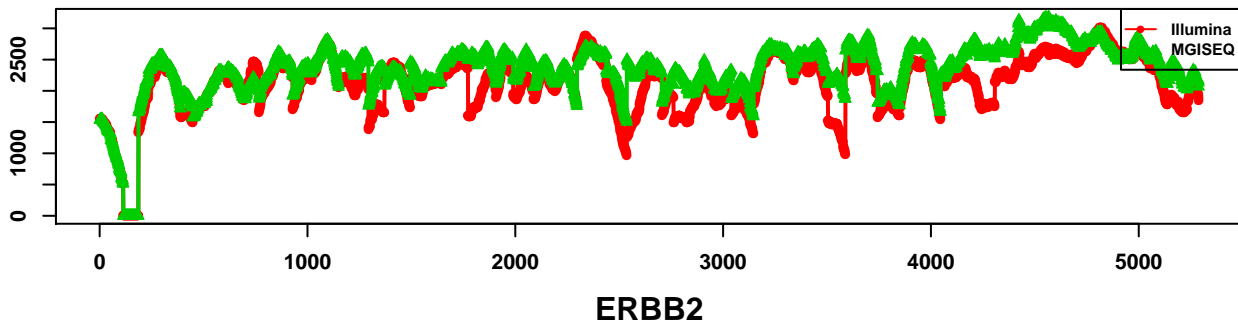

Sequencing Depth

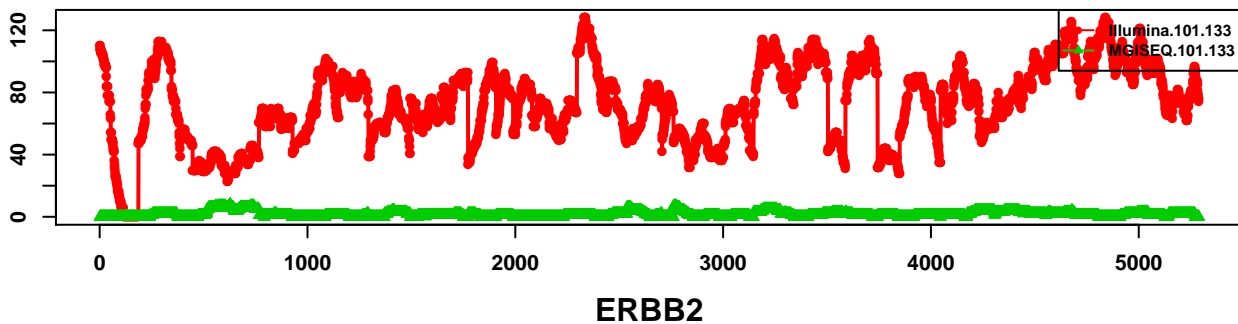

Sequencing Depth

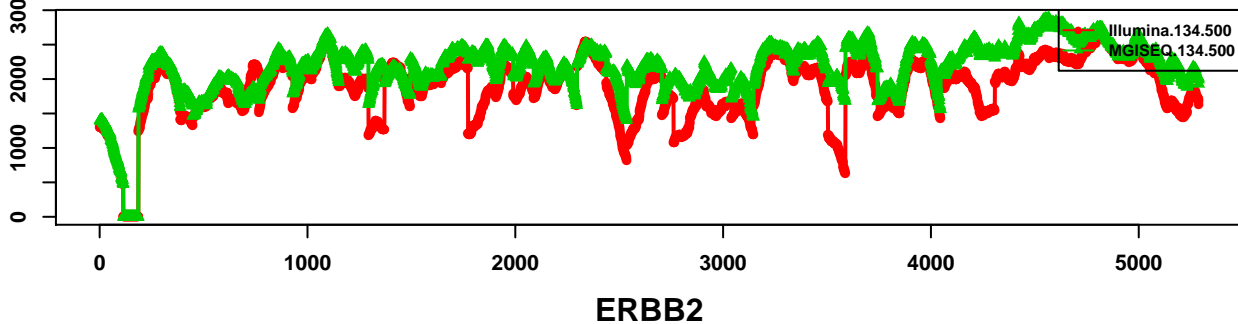

Supplement: Supplementary file 8 [file Presentation8.zip › ERBB2/19HE22140P.pdf]

Sequencing Depth

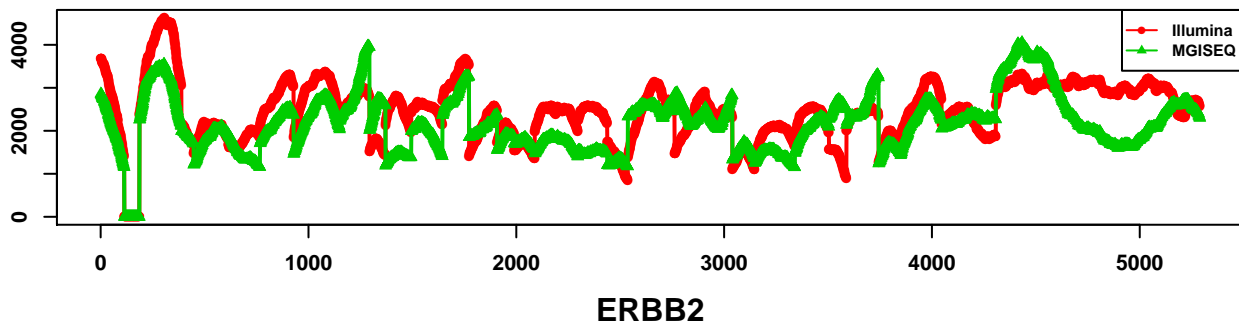

Sequencing Depth

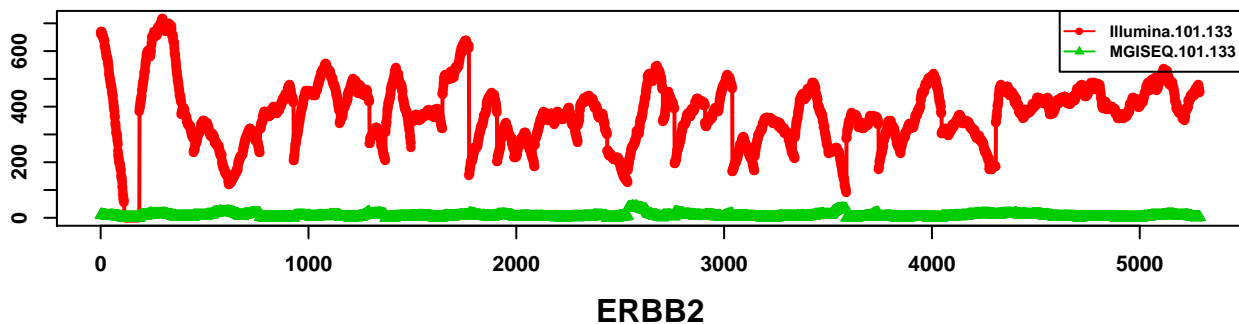

Sequencing Depth

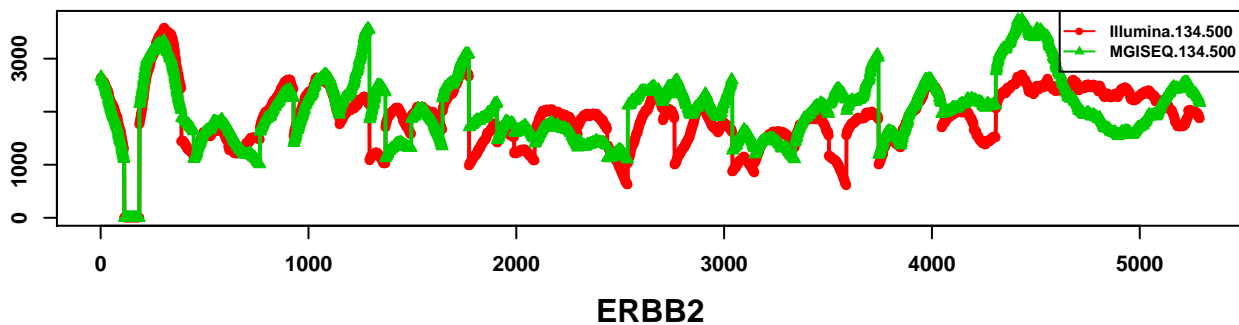

Supplement: Supplementary file 8 [file Presentation8.zip › ERBB2/19CF15531F.pdf]

Sequencing Depth

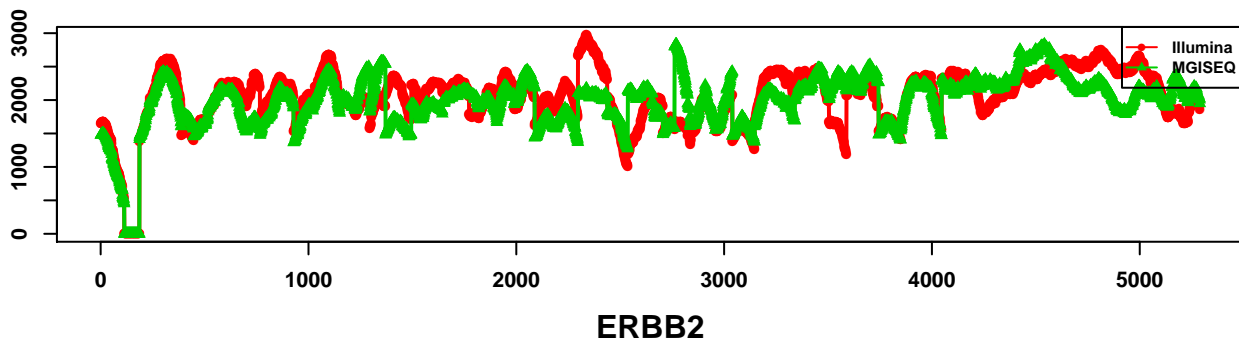

Sequencing Depth

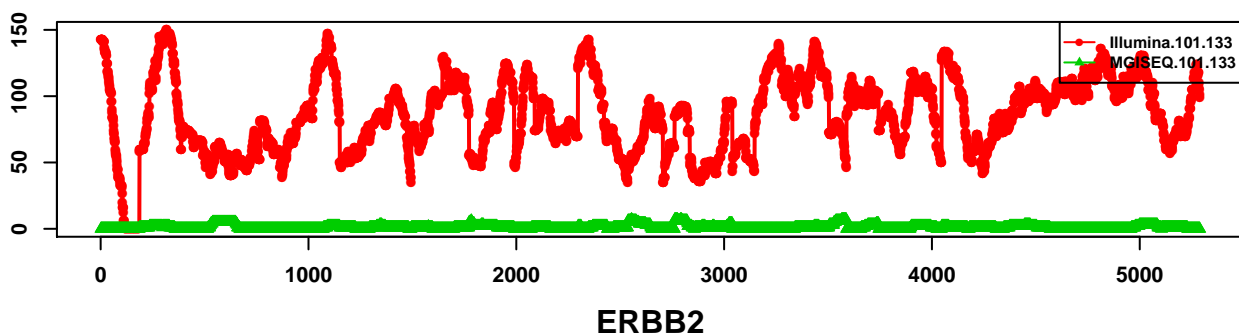

Sequencing Depth

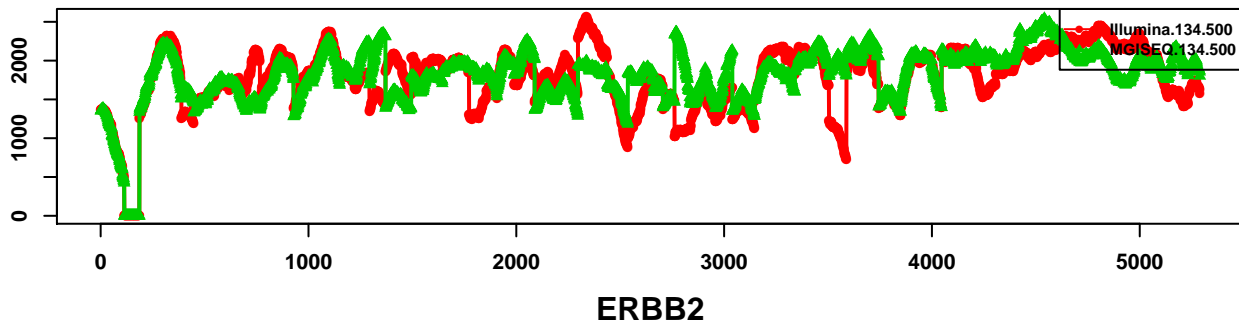

Supplement: Supplementary file 8 [file Presentation8.zip › ERBB2/M1900858-IP.pdf]

Sequencing Depth

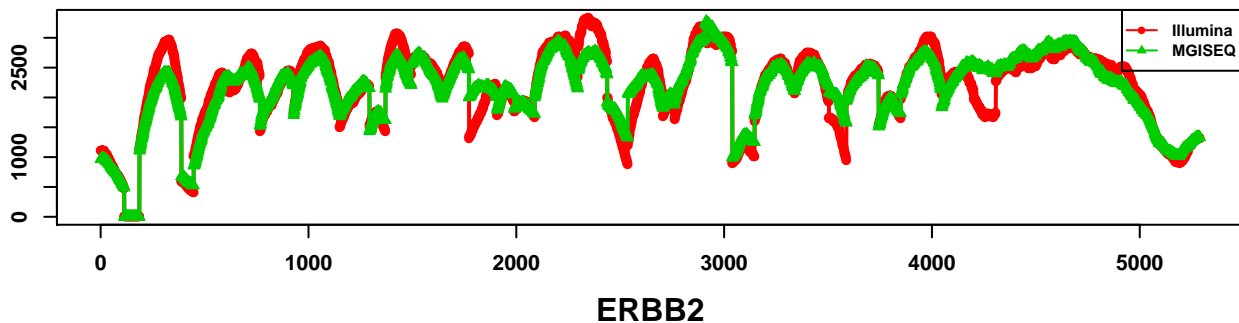

Sequencing Depth

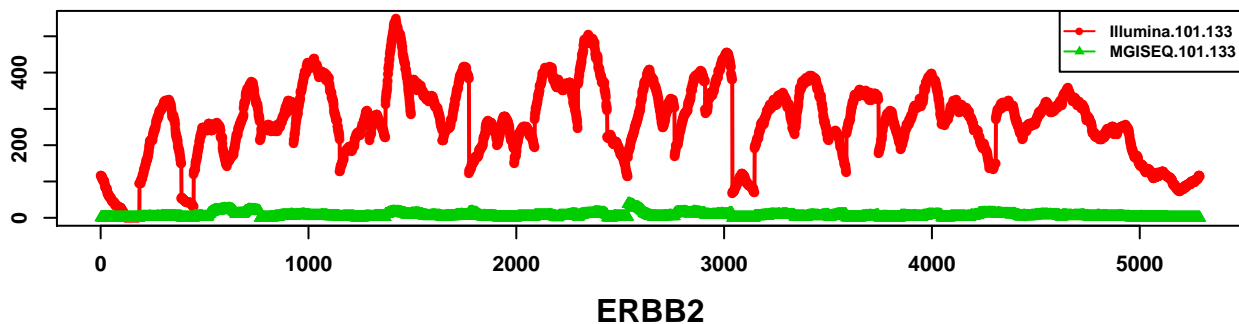

Sequencing Depth

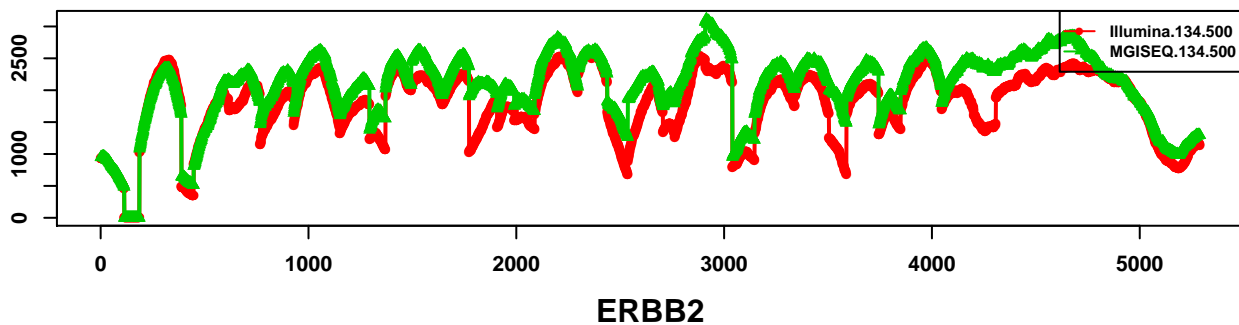

Supplement: Supplementary file 8 [file Presentation8.zip › ERBB2/19N01368F.pdf]

Sequencing Depth

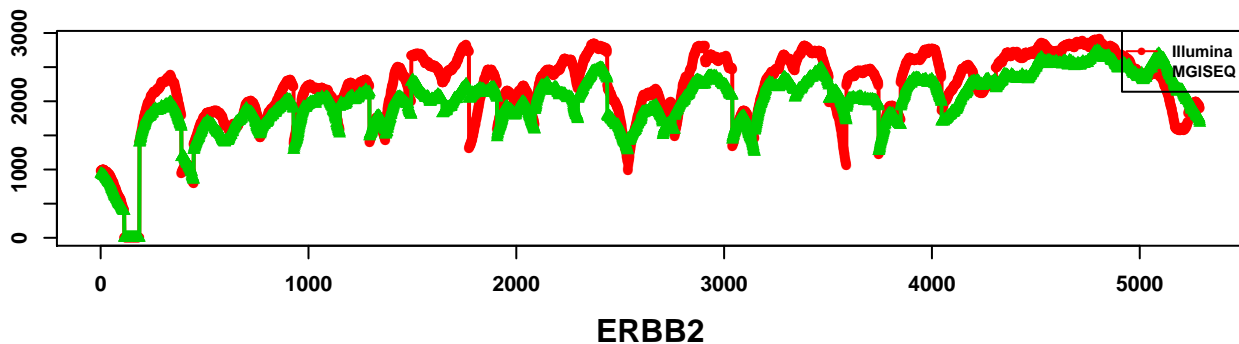

Sequencing Depth

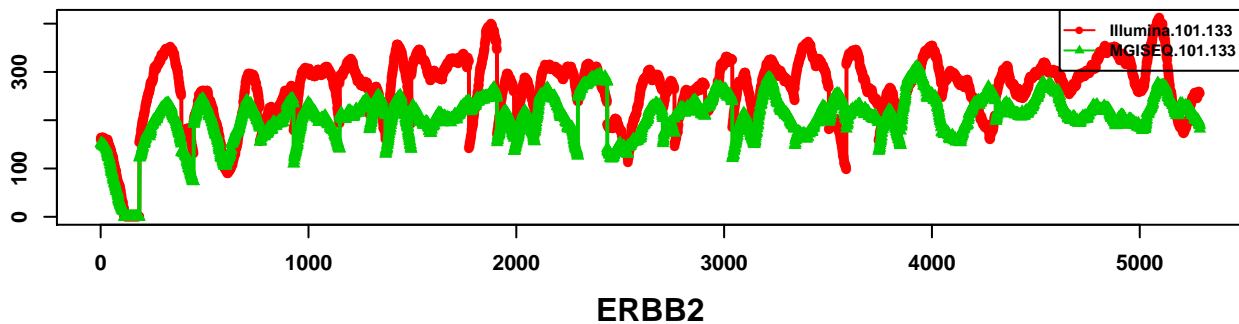

Sequencing Depth

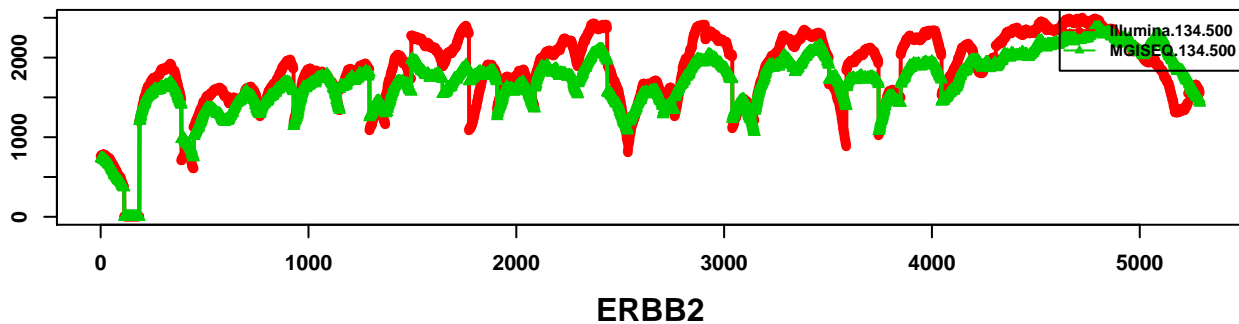

Supplement: Supplementary file 8 [file Presentation8.zip › ERBB2/19ZN12592T.pdf]

Sequencing Depth

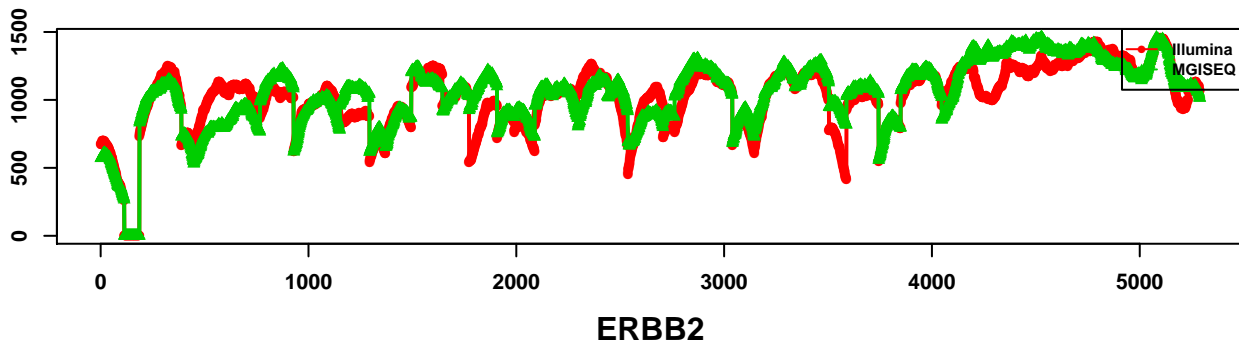

Sequencing Depth

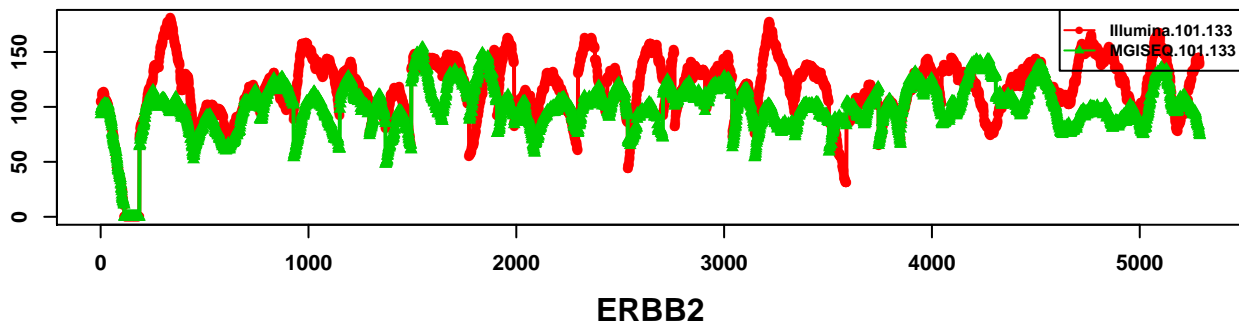

Sequencing Depth

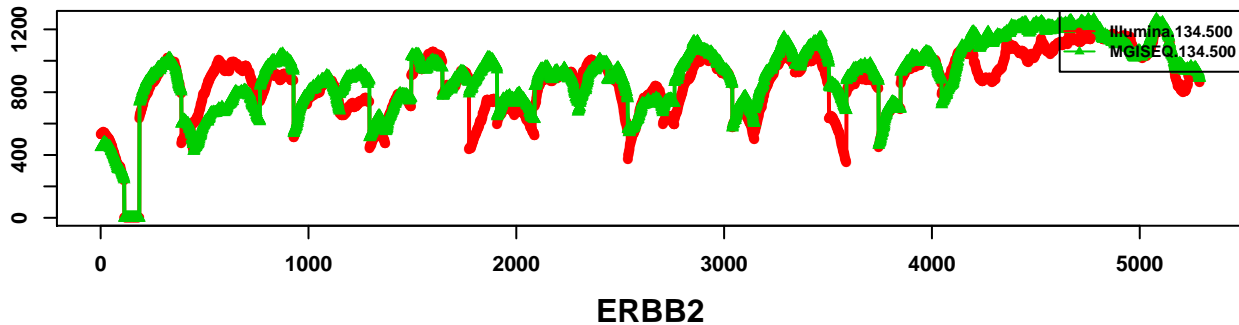

Supplement: Supplementary file 8 [file Presentation8.zip › ERBB2/ZK190805-G.pdf]

Sequencing Depth

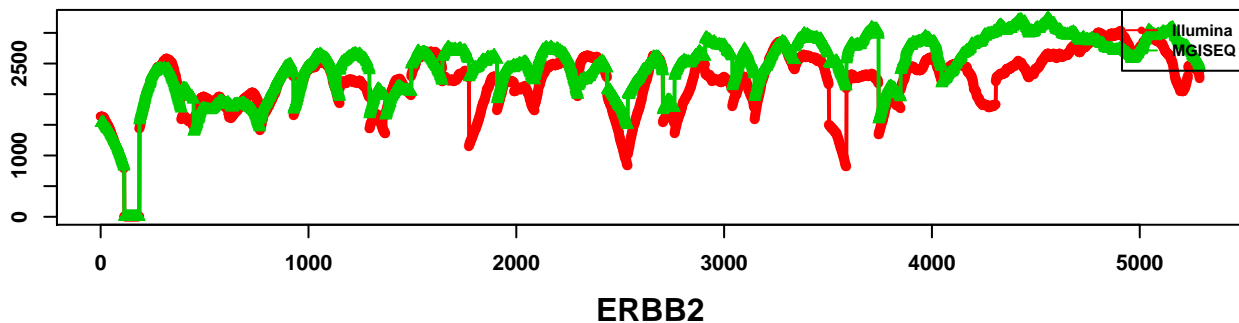

Sequencing Depth

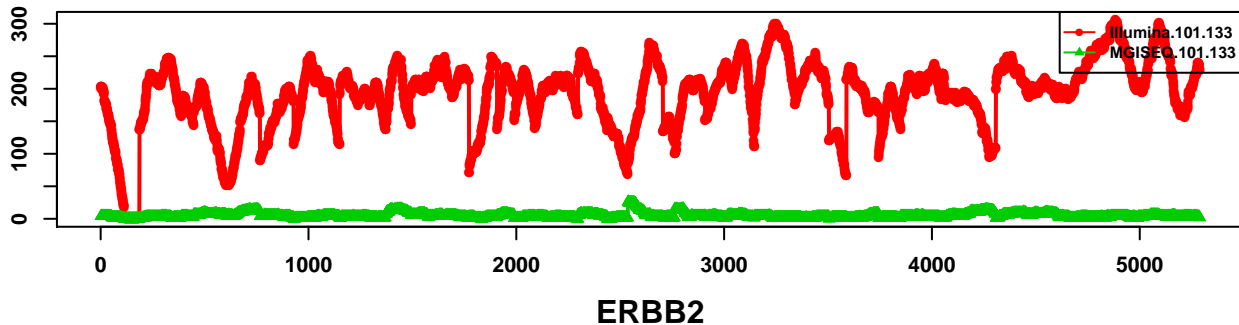

Sequencing Depth

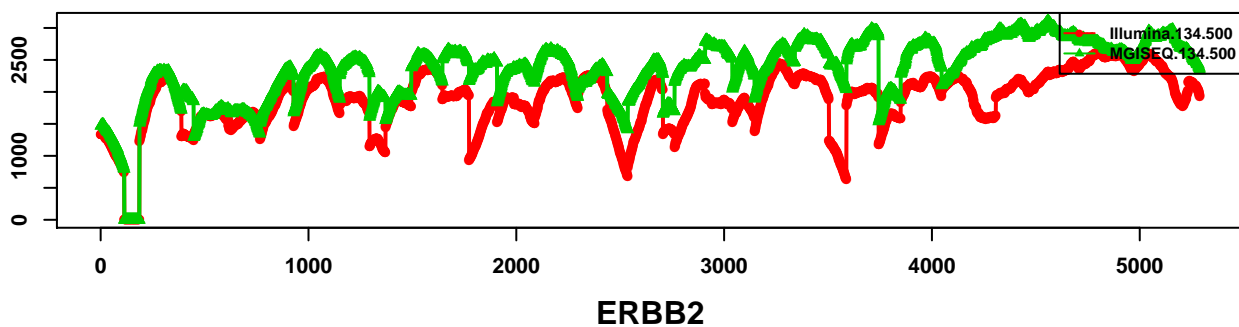

Supplement: Supplementary file 8 [file Presentation8.zip › ERBB2/19N01651F.pdf]

Sequencing Depth

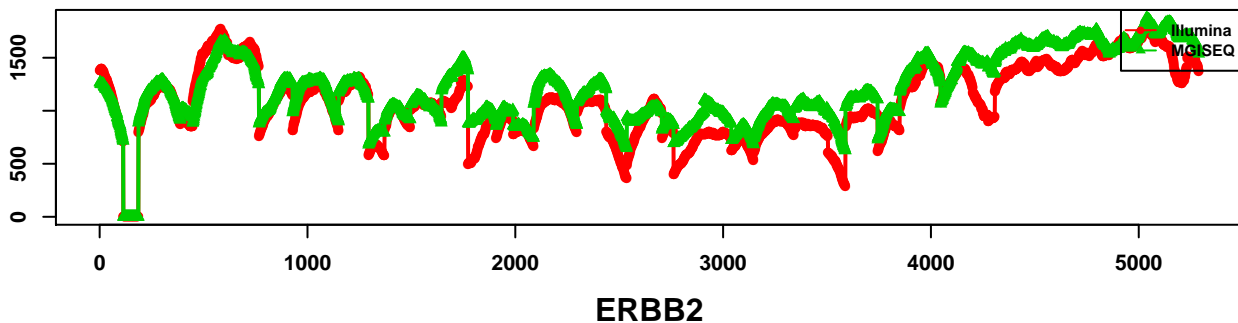

Sequencing Depth

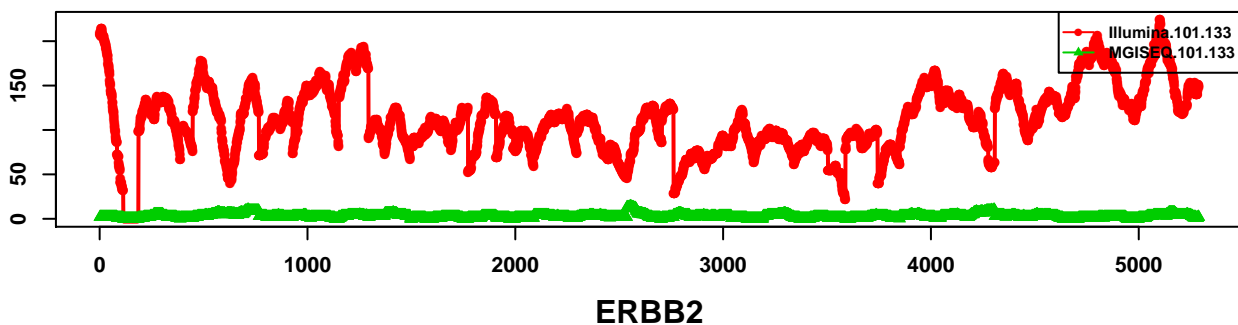

Sequencing Depth

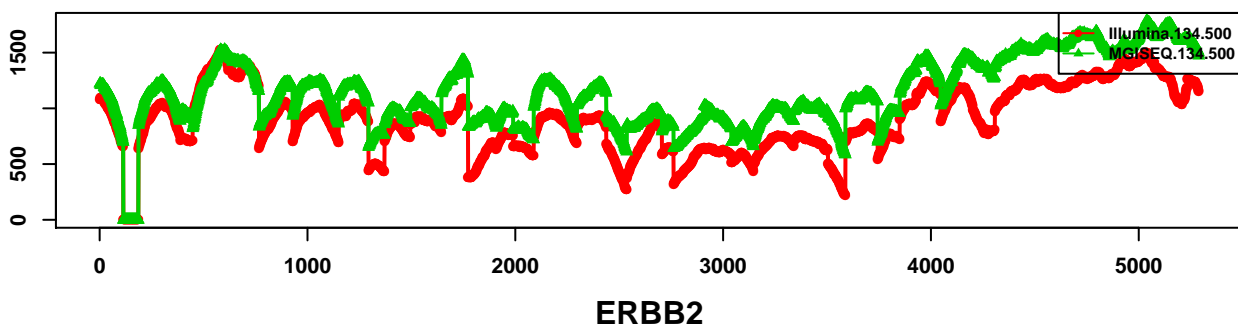

Supplement: Supplementary file 8 [file Presentation8.zip › ERBB2/19HE22005F.pdf]

Sequencing Depth

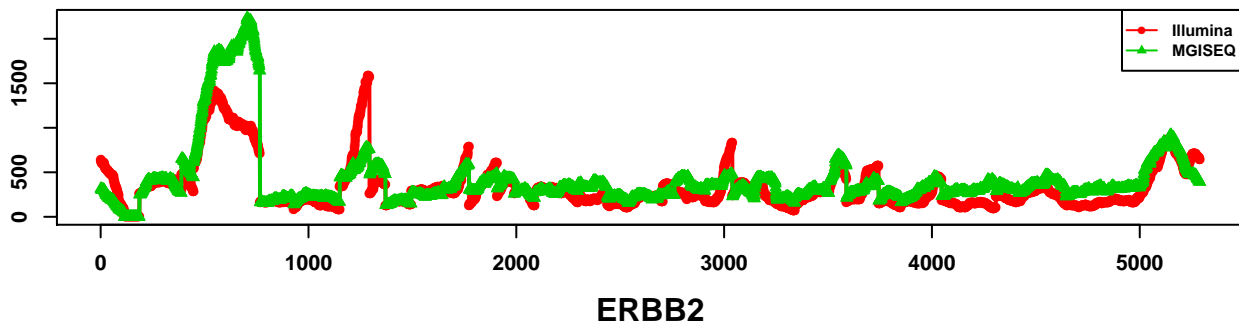

Sequencing Depth

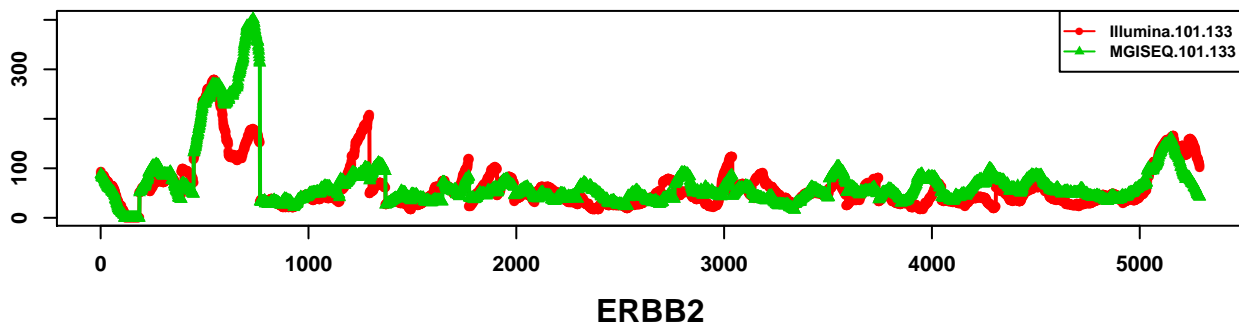

Sequencing Depth

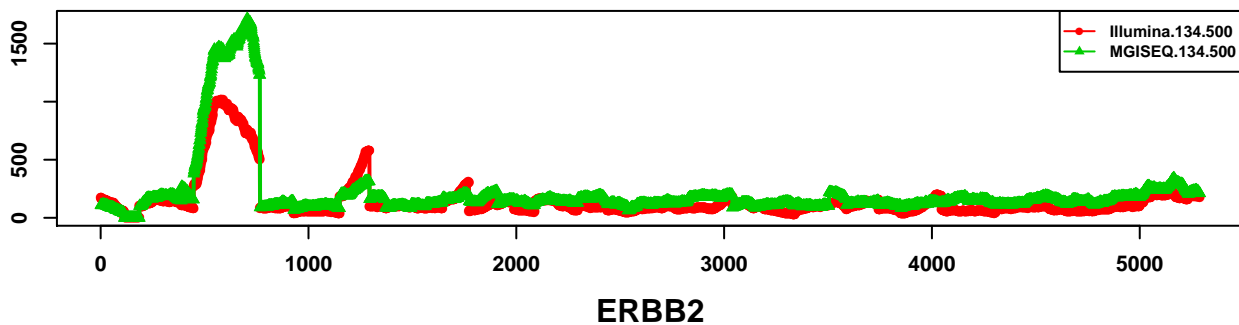

Supplement: Supplementary file 8 [file Presentation8.zip › ERBB2/19FC40420F.pdf]

Sequencing Depth

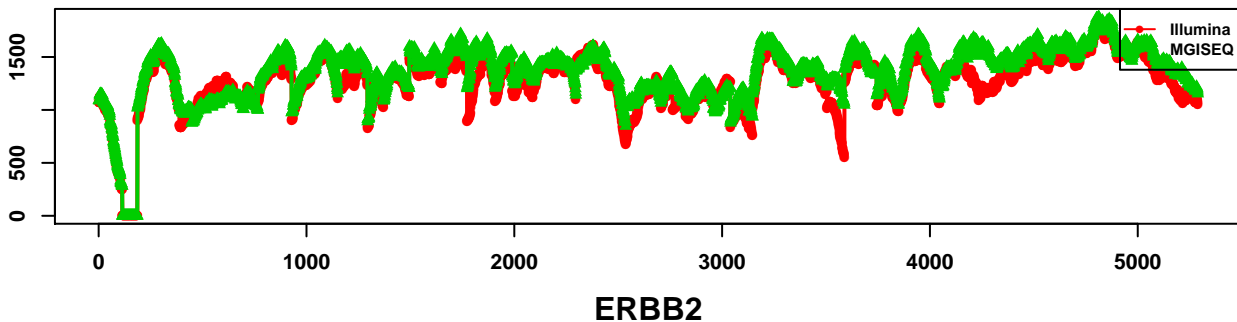

Sequencing Depth

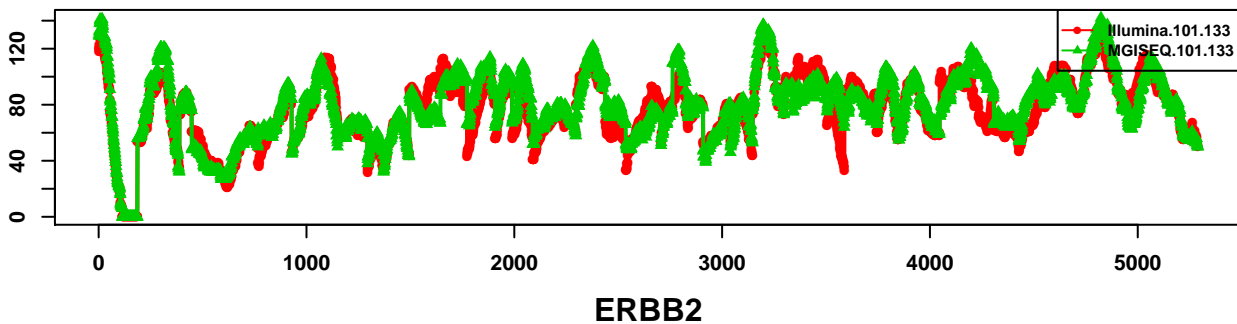

Sequencing Depth

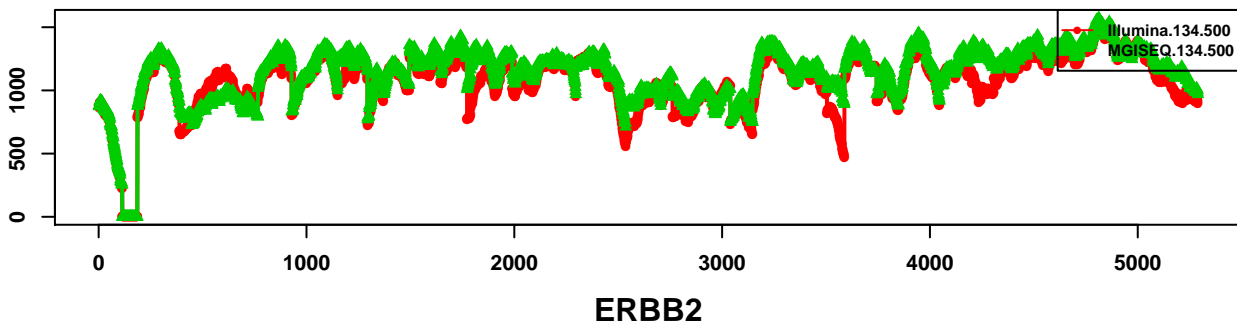

Supplement: Supplementary file 8 [file Presentation8.zip › ERBB2/M1901244P.pdf]

Sequencing Depth

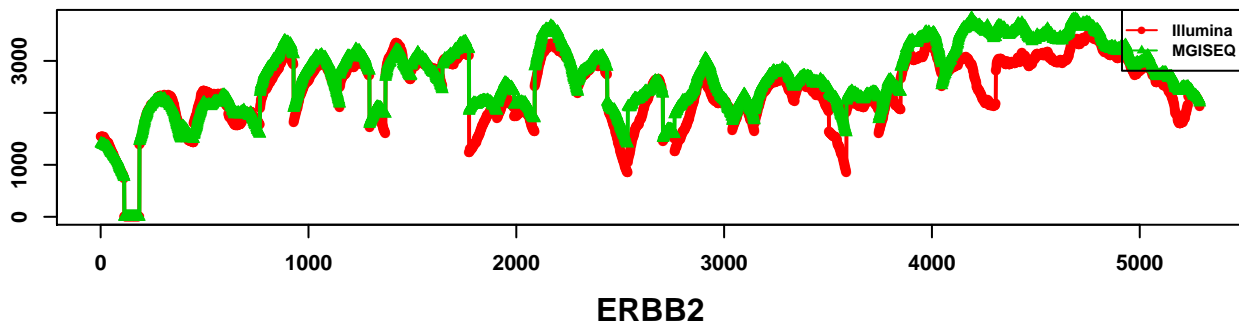

Sequencing Depth

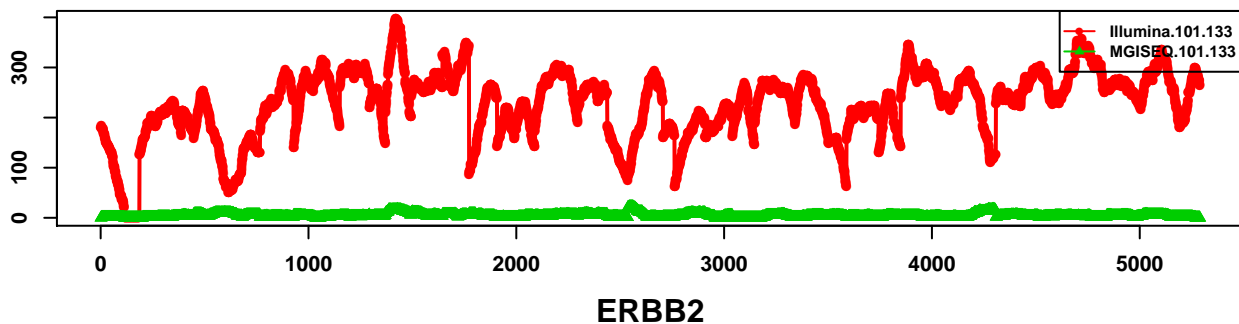

Sequencing Depth

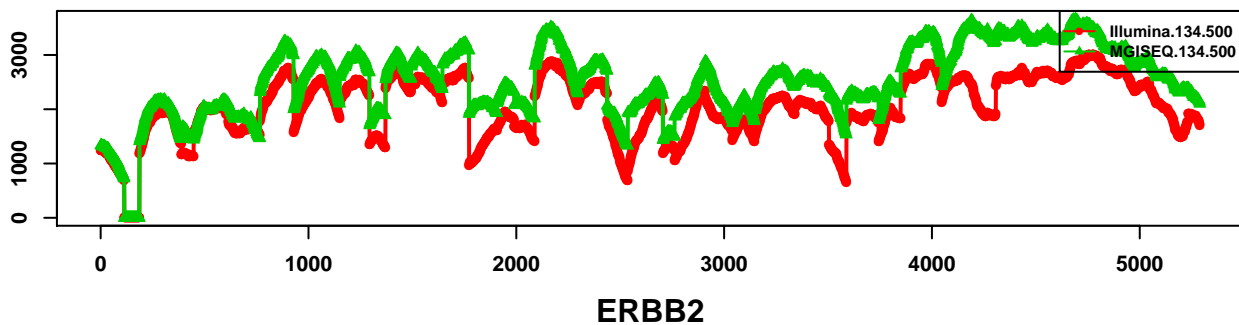

Supplement: Supplementary file 8 [file Presentation8.zip › ERBB2/19HS86160F.pdf]

Sequencing Depth

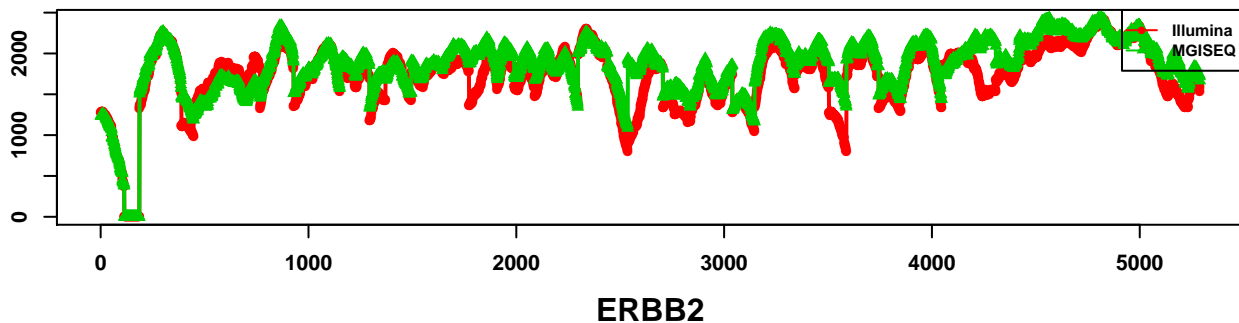

Sequencing Depth

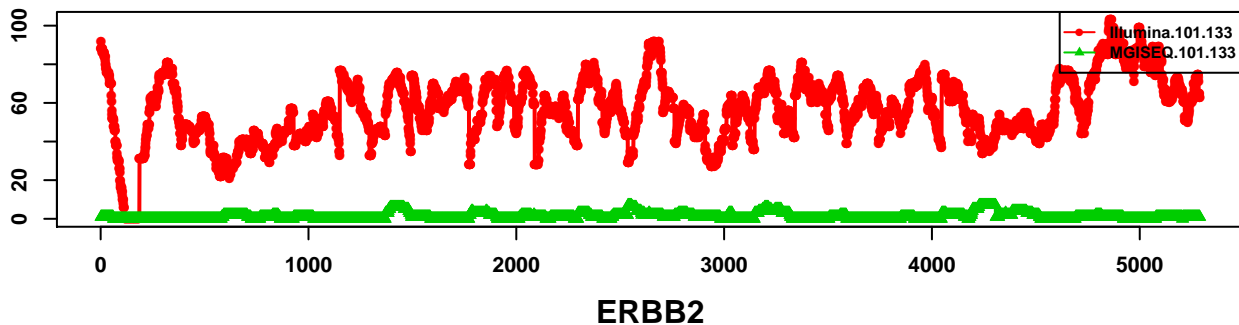

Sequencing Depth

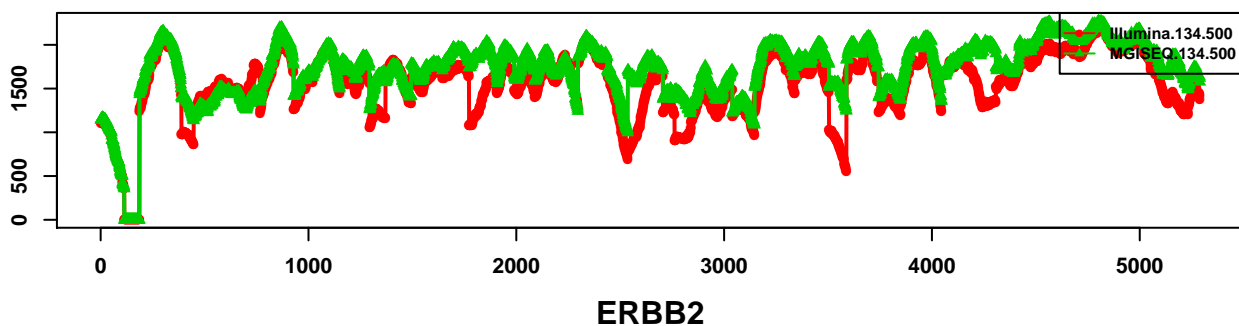

Supplement: Supplementary file 8 [file Presentation8.zip › ERBB2/19JM45609P.pdf]

Sequencing Depth

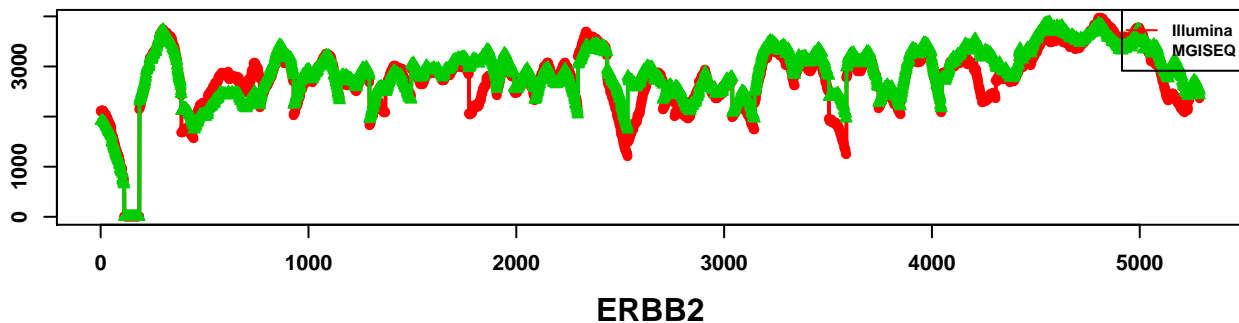

Sequencing Depth

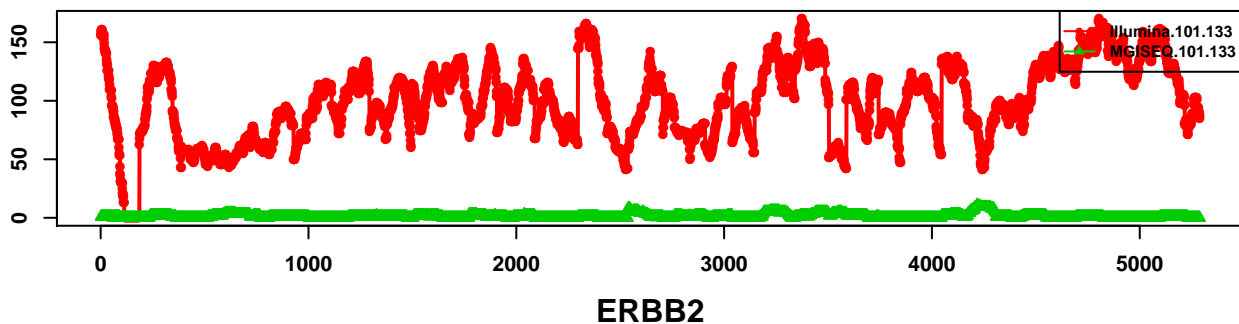

Sequencing Depth

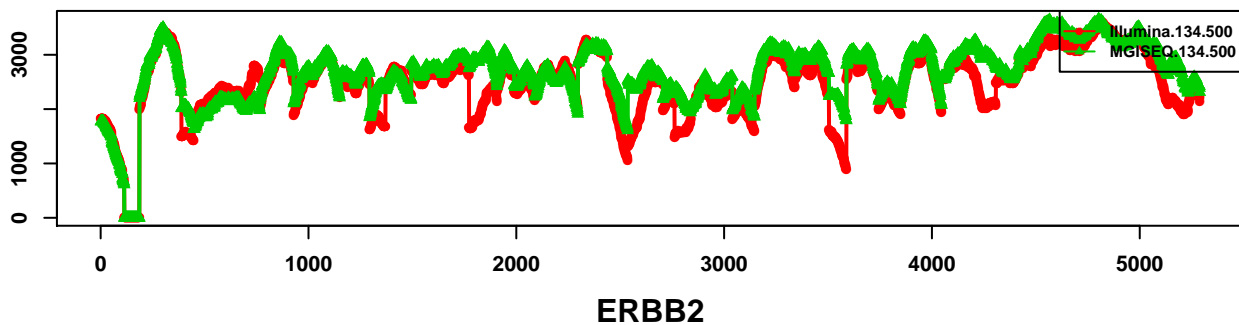

Supplement: Supplementary file 8 [file Presentation8.zip › ERBB2/19PT90802P.pdf]

Sequencing Depth

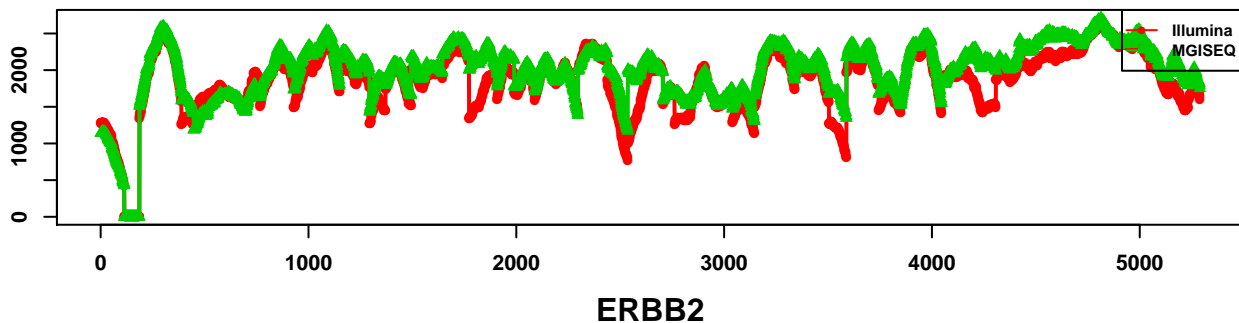

Sequencing Depth

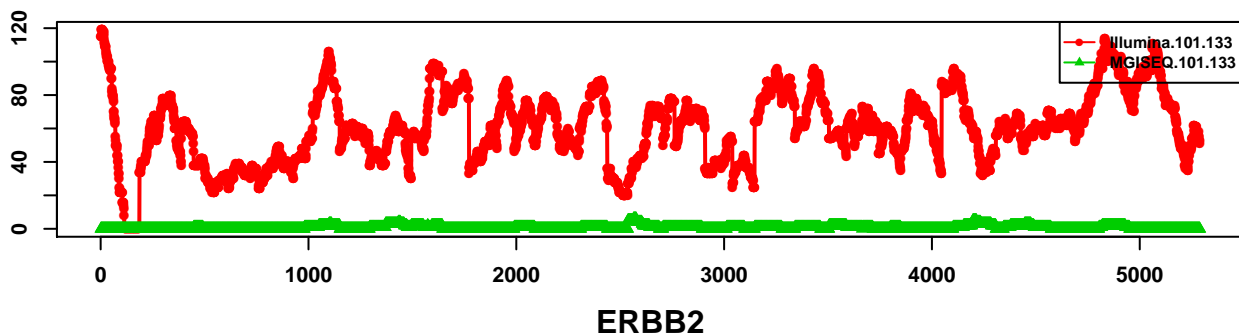

Sequencing Depth

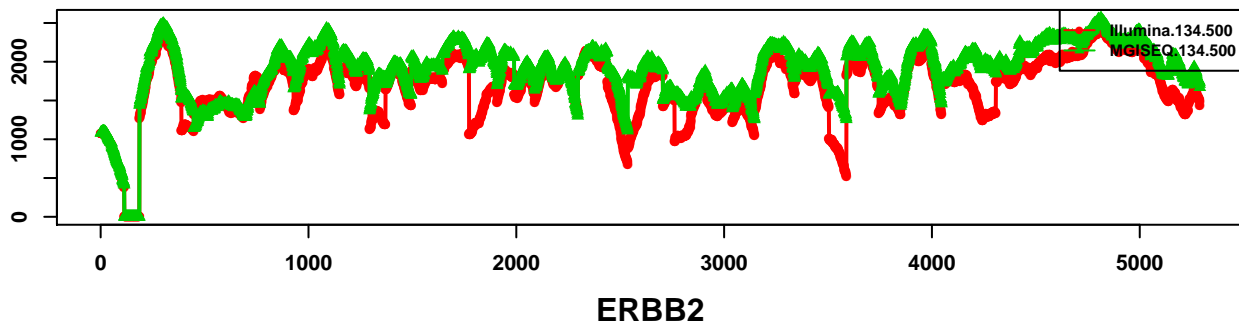

Supplement: Supplementary file 8 [file Presentation8.zip › ERBB2/19N01684P.pdf]
