# Supplementary figures and images for: Evaluation of the MGISEQ-2000 Sequencing Platform for Illumina Target Capture Sequencing Libraries (part 6 of 6)
Source: Front Genet. 2021 Oct 27;12:730519. doi: 10.3389/fgene.2021.730519 (PMC8578046; doi:10.3389/fgene.2021.730519)

Sequencing Depth

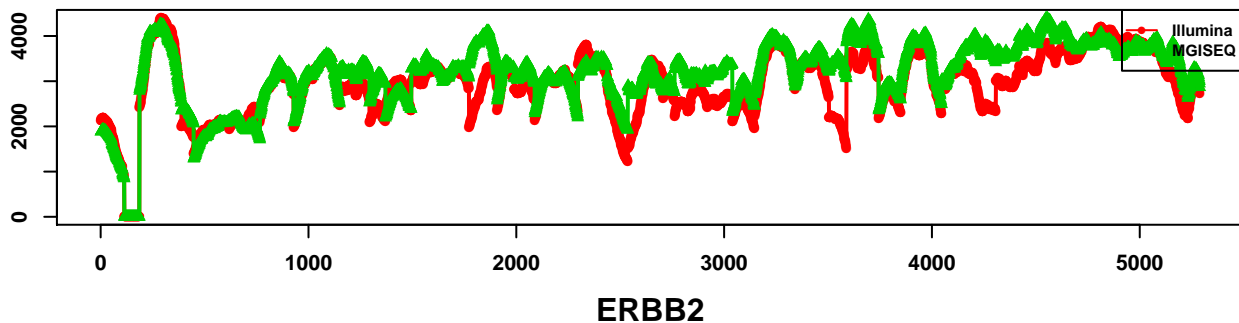

Sequencing Depth

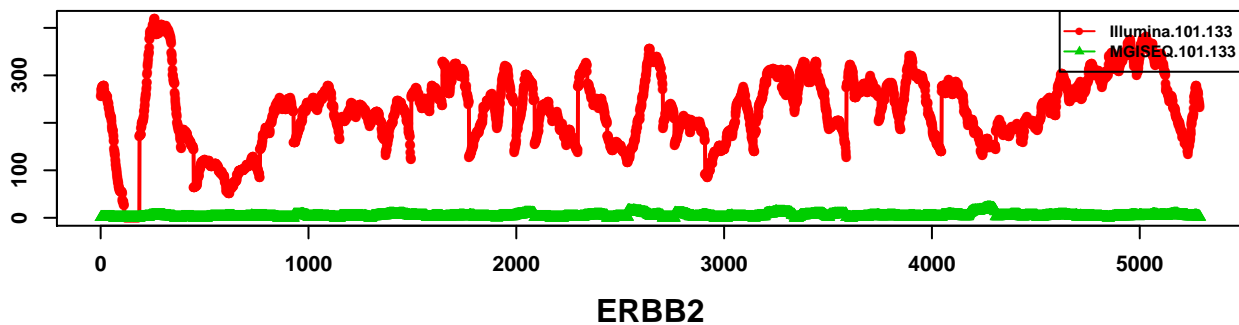

Sequencing Depth

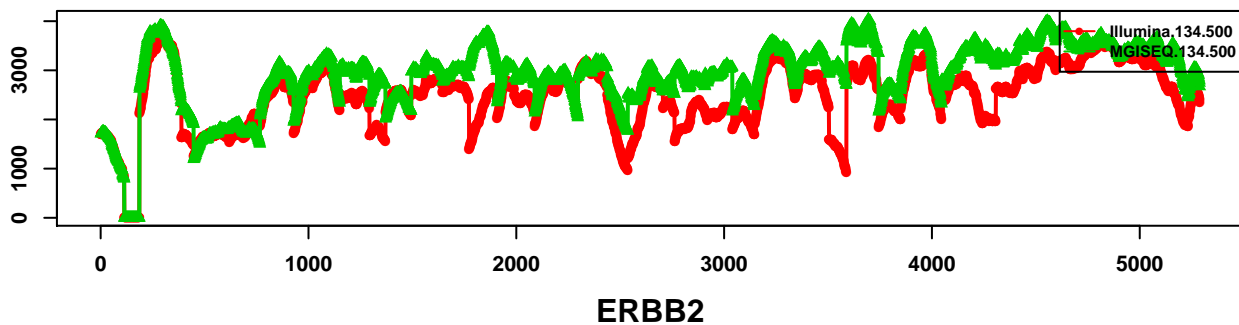

Supplement: Supplementary file 9 [file Presentation6.zip › ERBB2/19N01384P.pdf]

Sequencing Depth

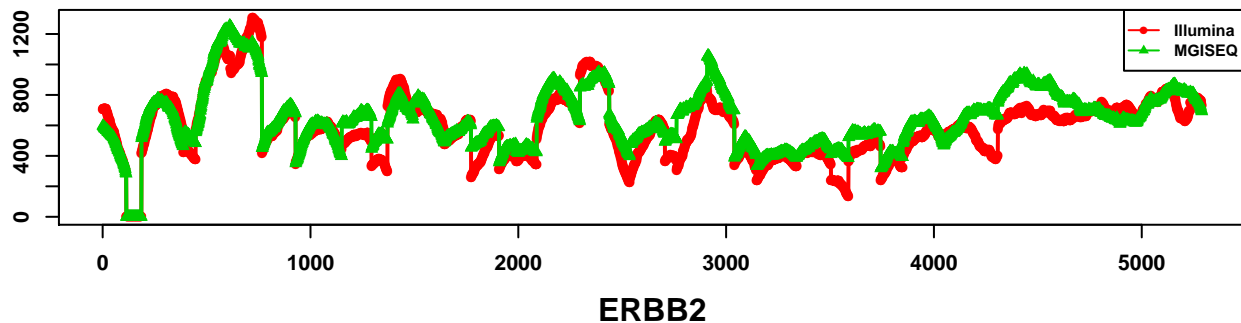

Sequencing Depth

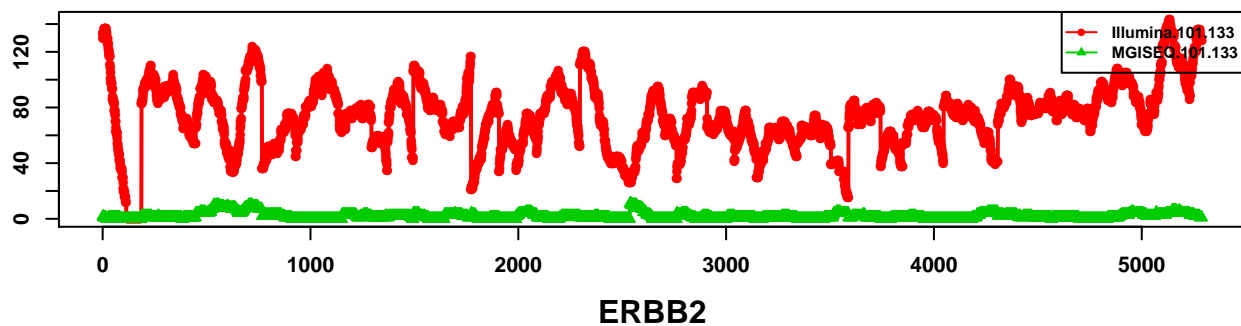

Sequencing Depth

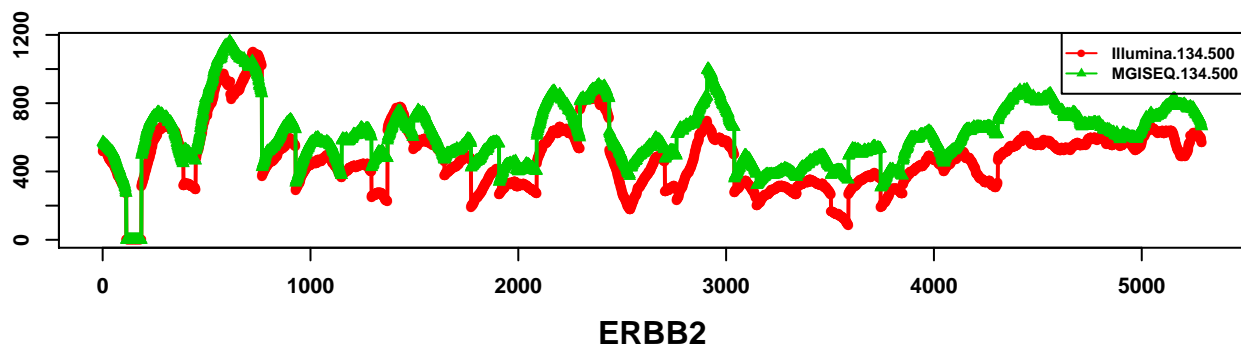

Supplement: Supplementary file 9 [file Presentation6.zip › ERBB2/19N01652F.pdf]

Sequencing Depth

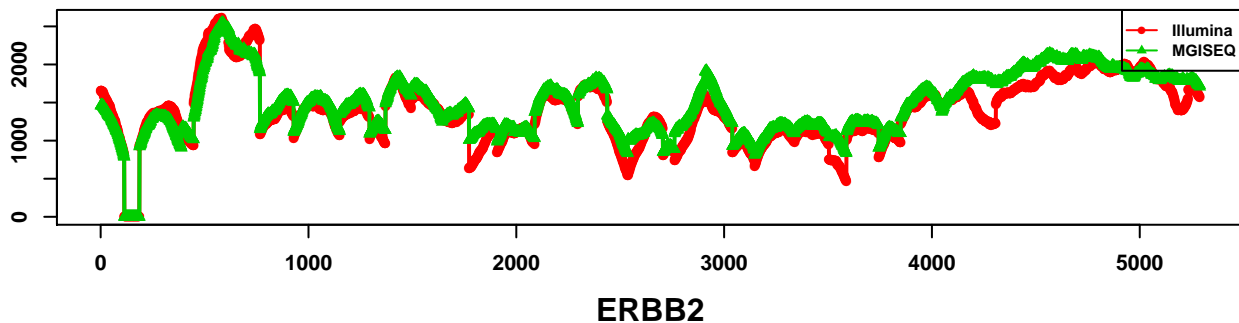

Sequencing Depth

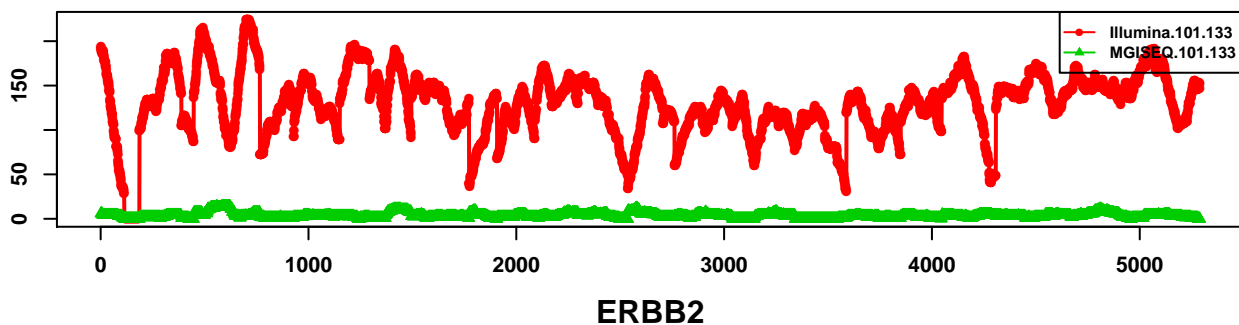

Sequencing Depth

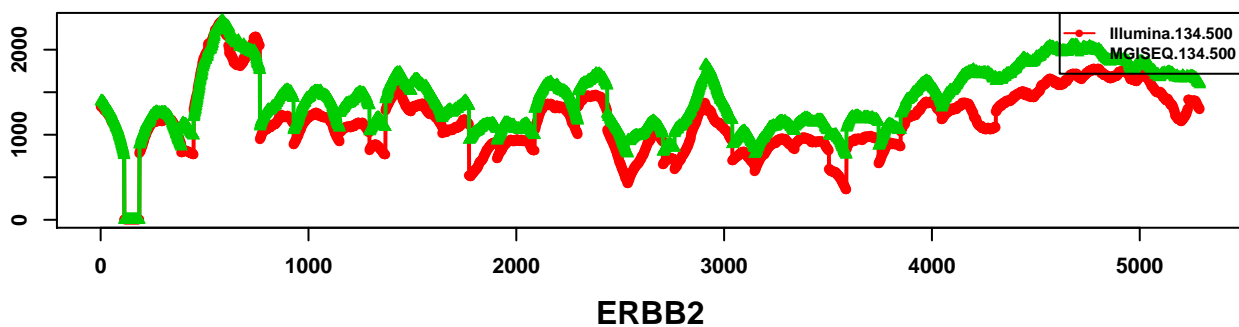

Supplement: Supplementary file 9 [file Presentation6.zip › ERBB2/19HE22006F.pdf]

Sequencing Depth

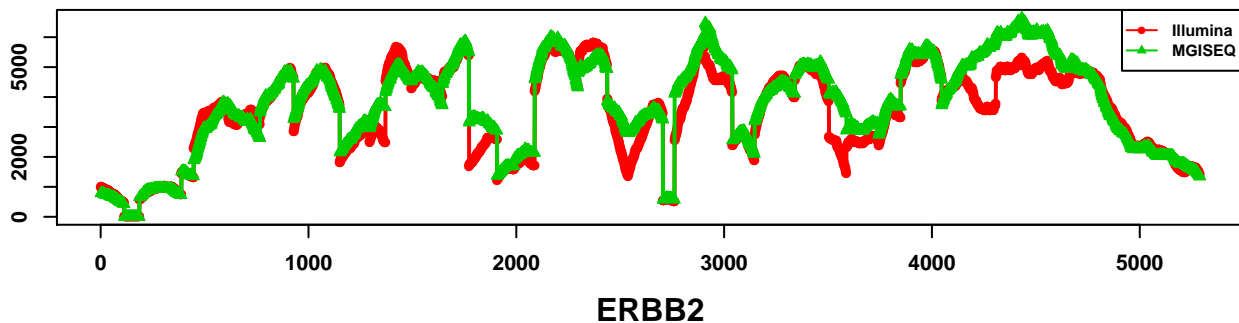

Sequencing Depth

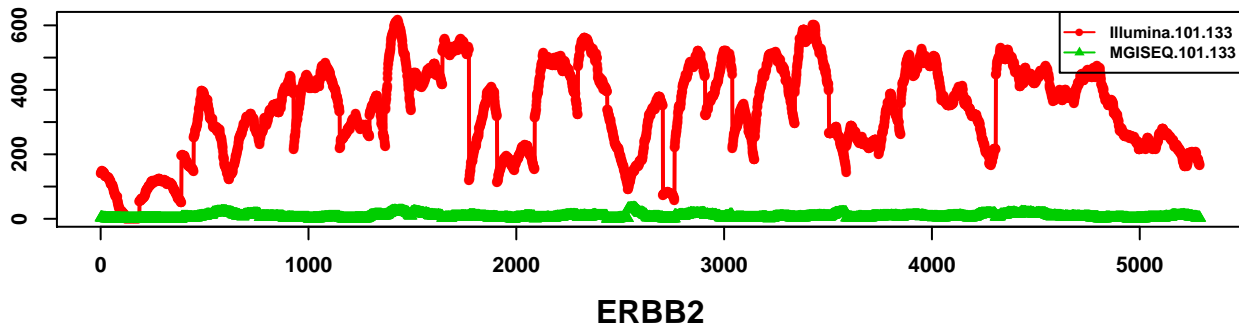

Sequencing Depth

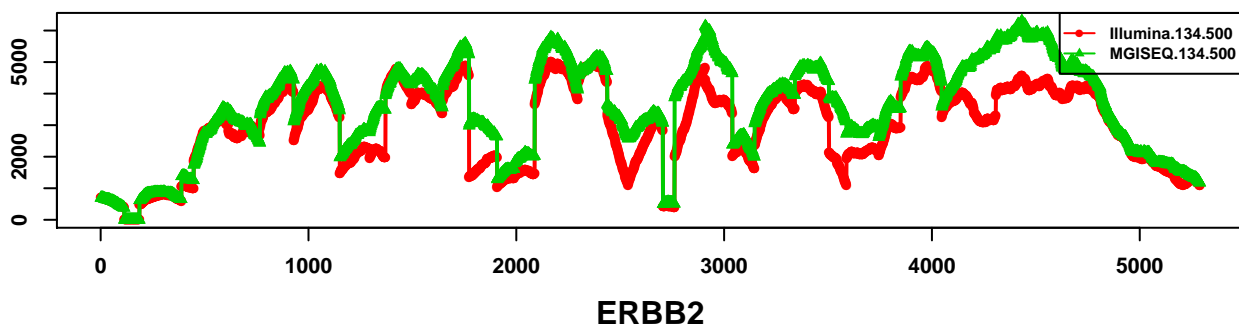

Supplement: Supplementary file 9 [file Presentation6.zip › ERBB2/19FC40248F.pdf]

Sequencing Depth

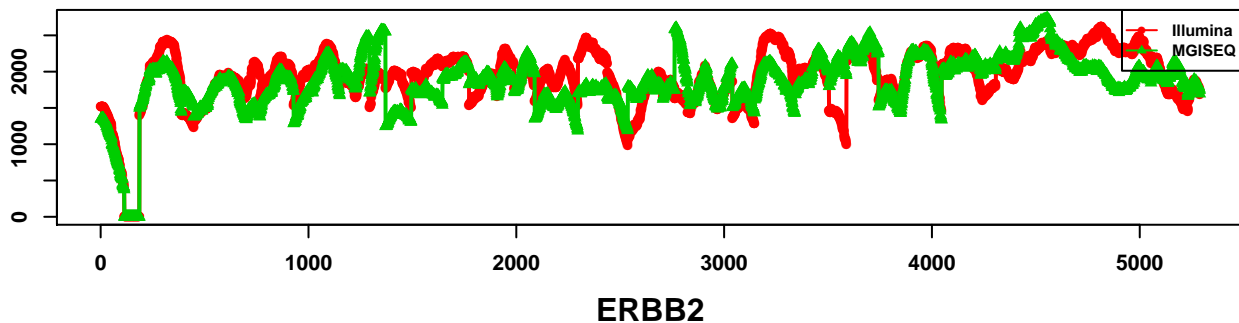

Sequencing Depth

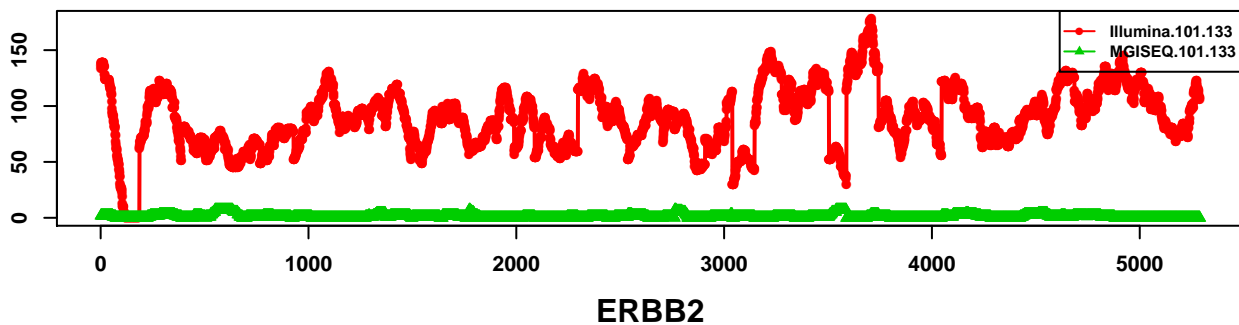

Sequencing Depth

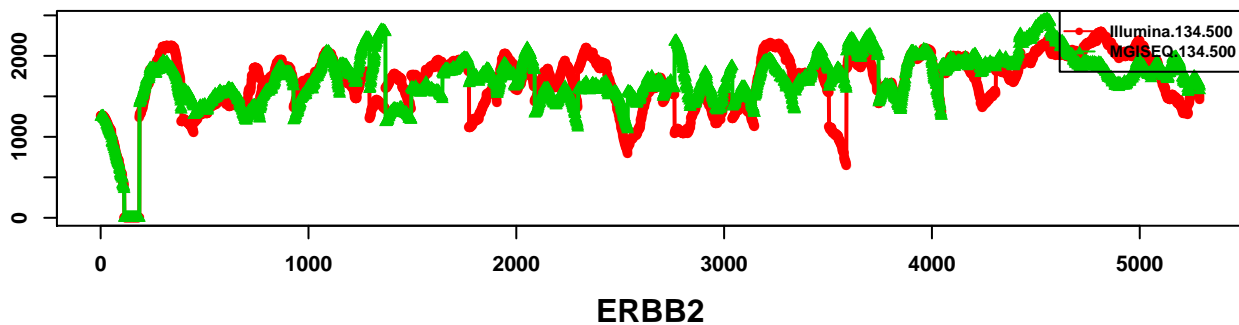

Supplement: Supplementary file 9 [file Presentation6.zip › ERBB2/19N01668P.pdf]

Sequencing Depth

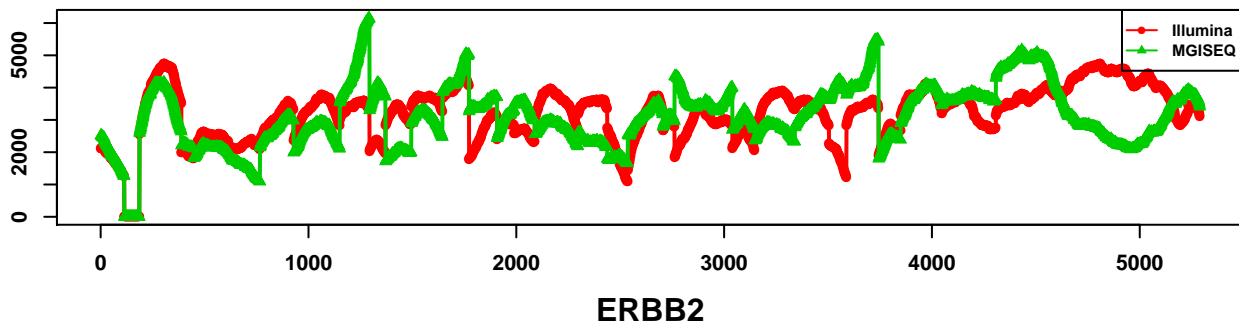

Sequencing Depth

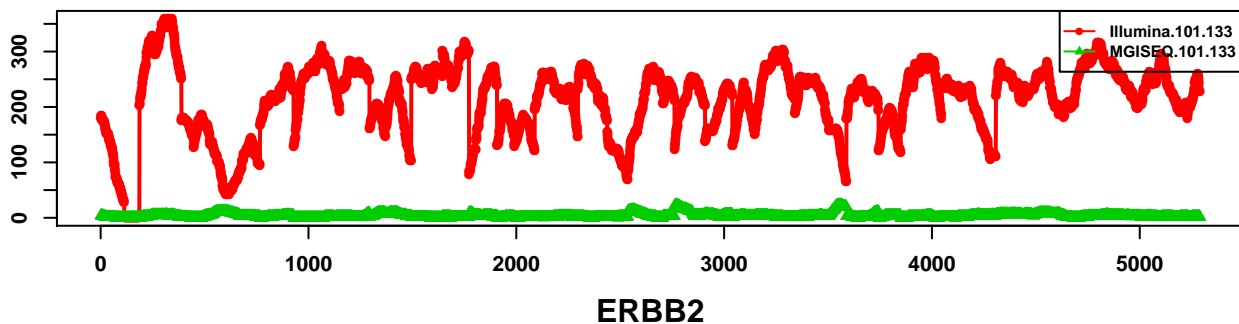

Sequencing Depth

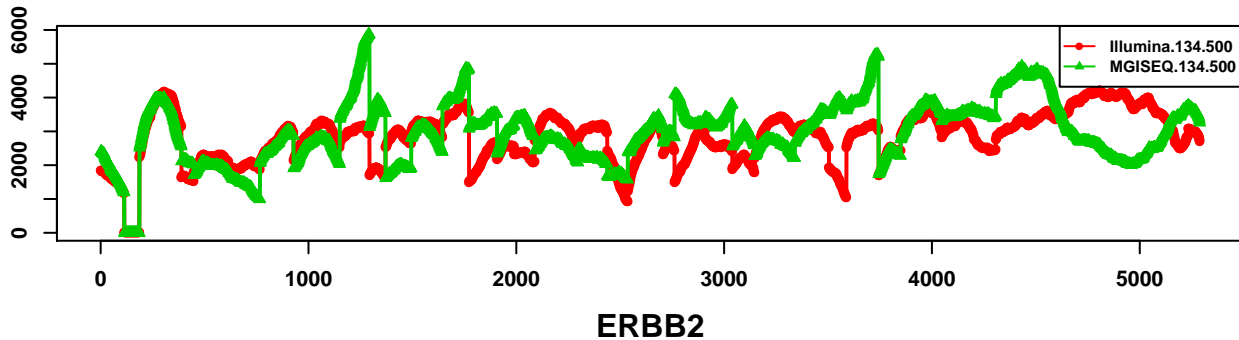

Supplement: Supplementary file 9 [file Presentation6.zip › ERBB2/19N01669T.pdf]

Sequencing Depth

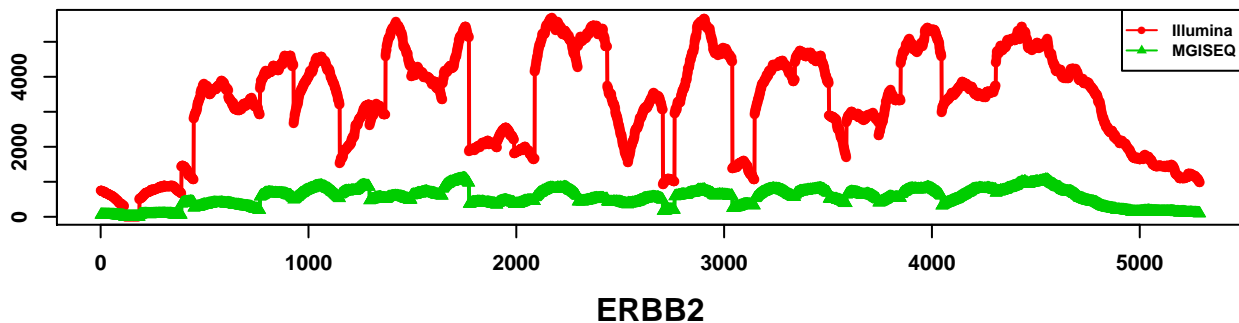

Sequencing Depth

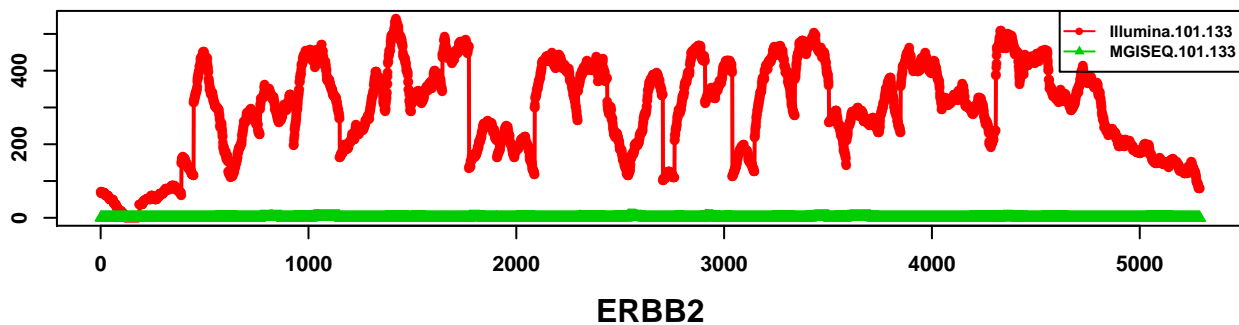

Sequencing Depth

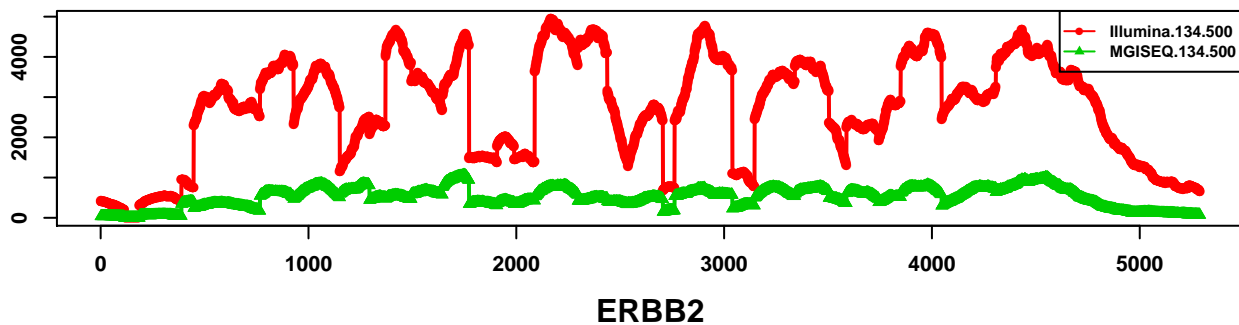

Supplement: Supplementary file 9 [file Presentation6.zip › ERBB2/19N01664F.pdf]

Sequencing Depth

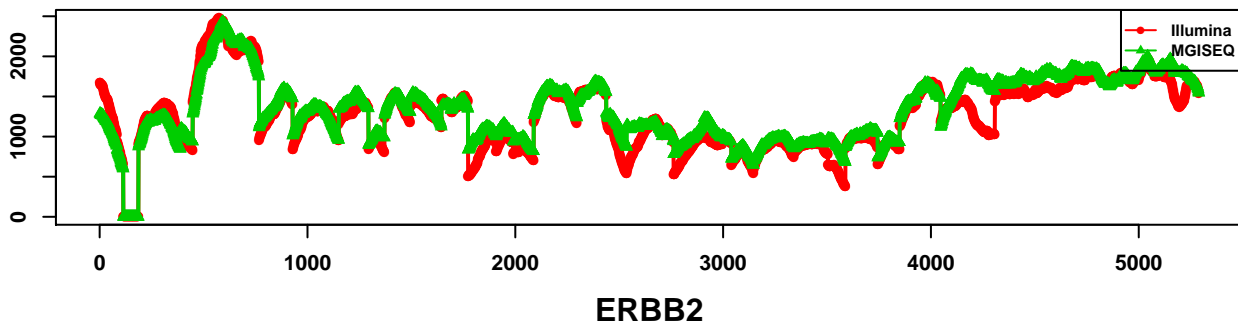

Sequencing Depth

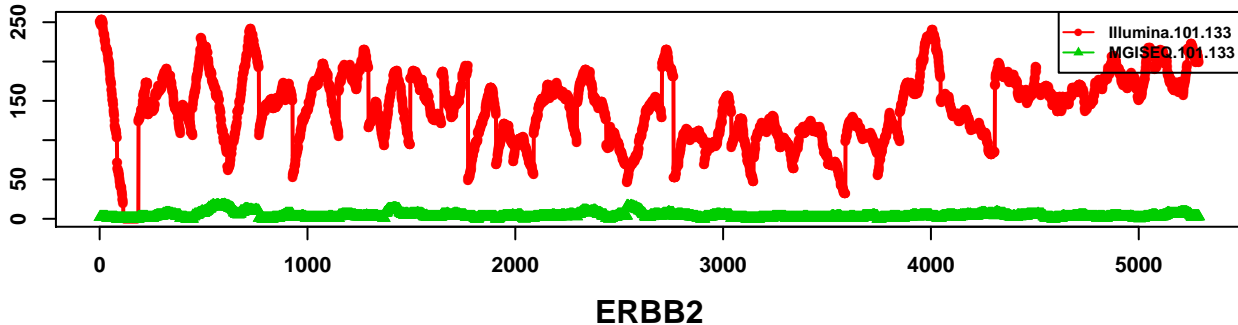

Sequencing Depth

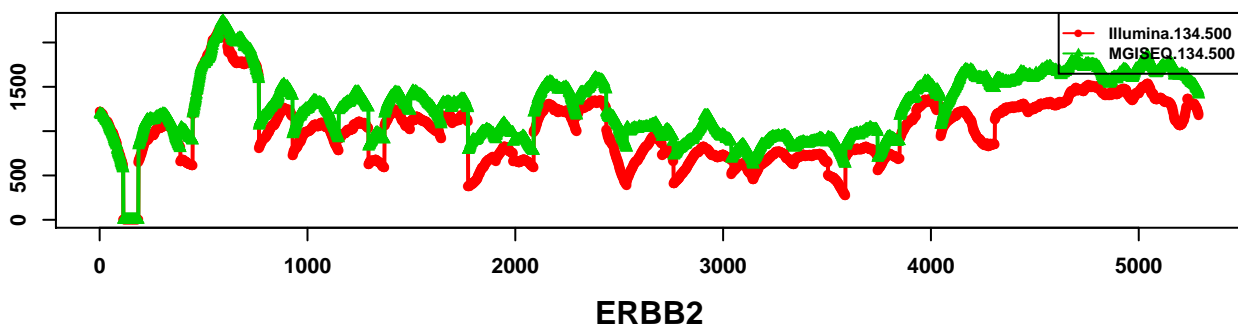

Supplement: Supplementary file 9 [file Presentation6.zip › ERBB2/19HE22067F.pdf]

Sequencing Depth

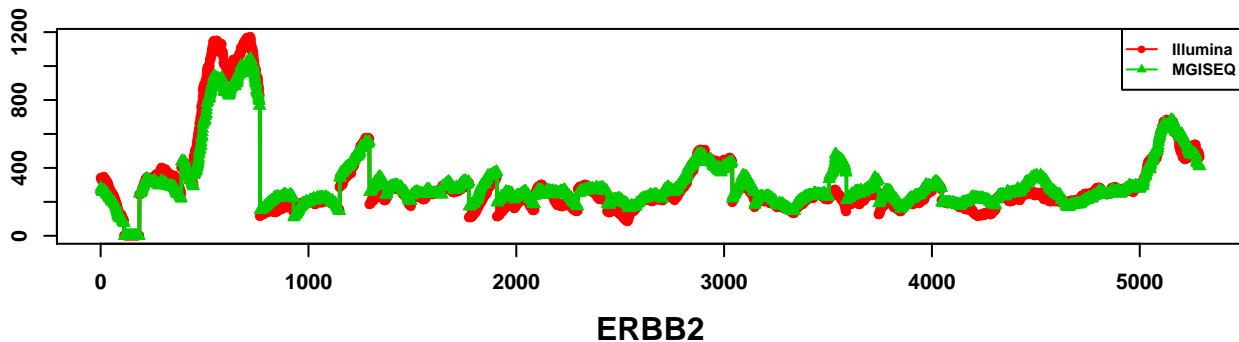

Sequencing Depth

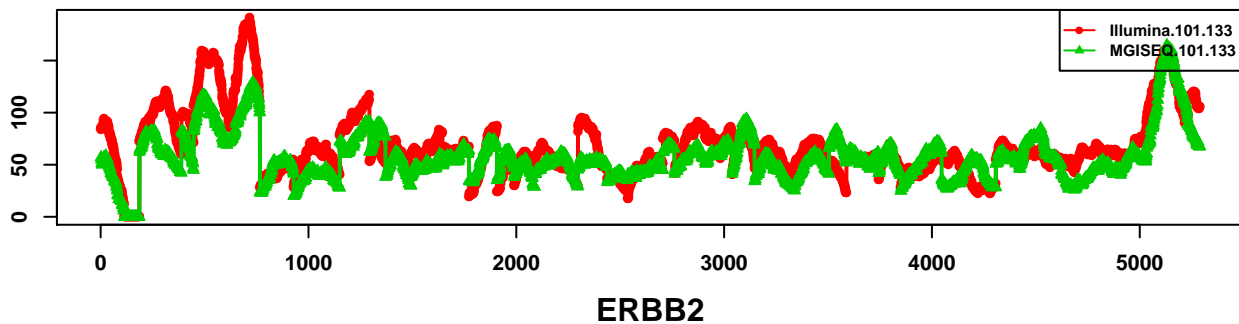

Sequencing Depth

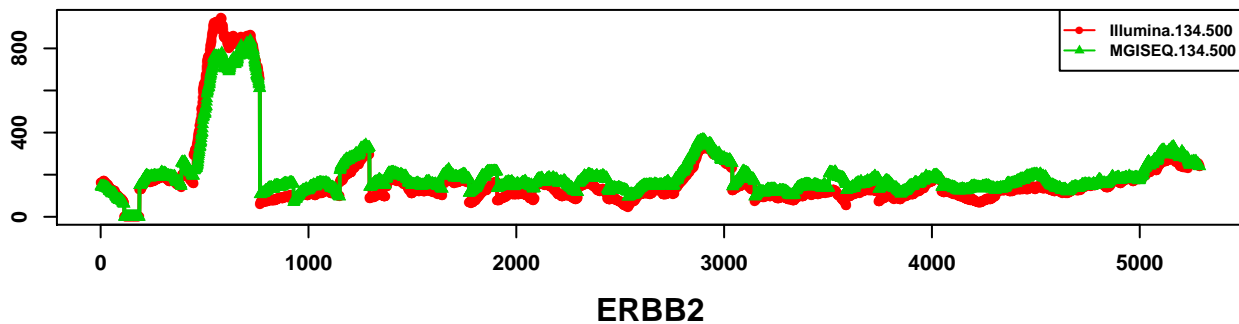

Supplement: Supplementary file 9 [file Presentation6.zip › ERBB2/19CF15713F.pdf]

Sequencing Depth

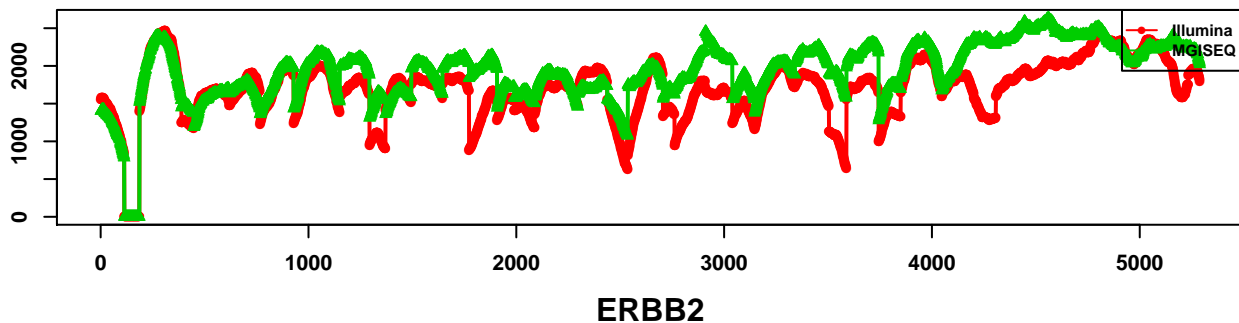

Sequencing Depth

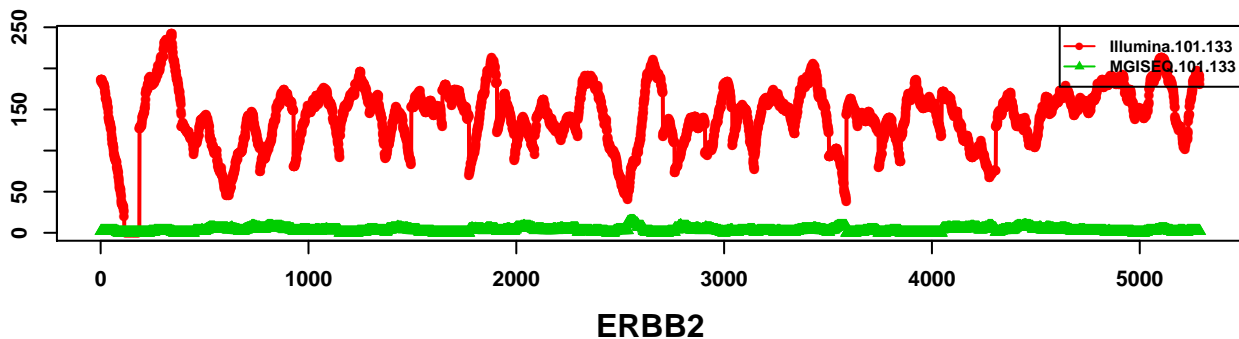

Sequencing Depth

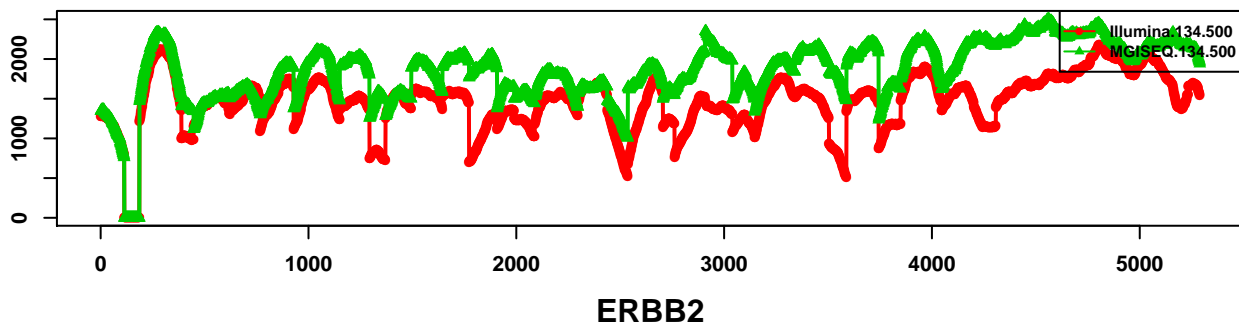

Supplement: Supplementary file 9 [file Presentation6.zip › ERBB2/19ZN12352H-R.pdf]

Sequencing Depth

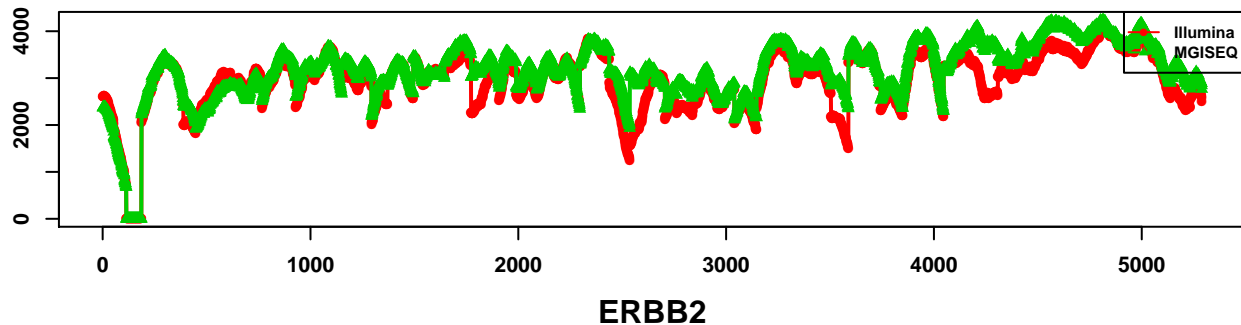

Sequencing Depth

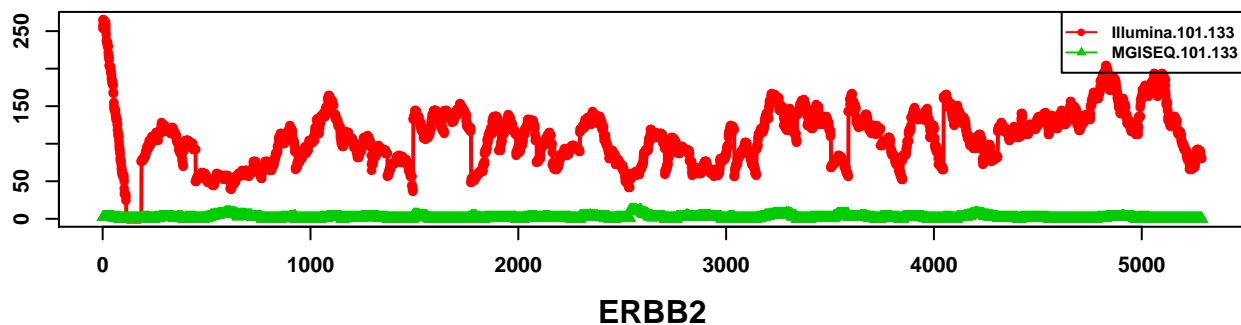

Sequencing Depth

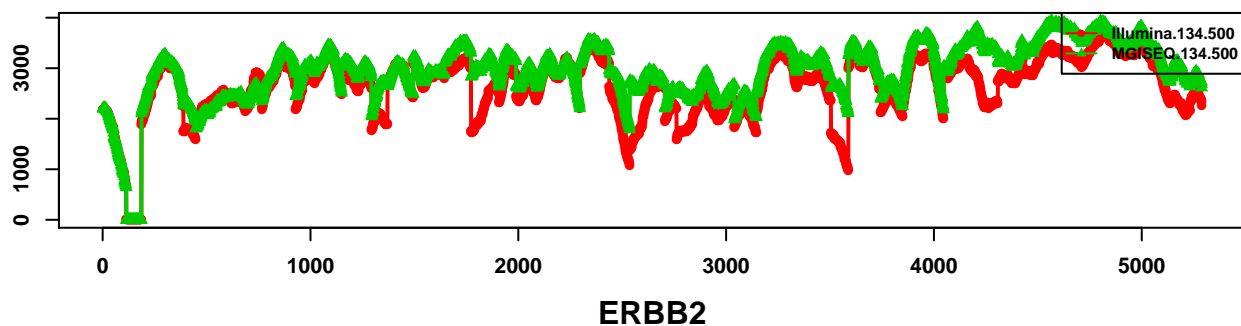

Supplement: Supplementary file 9 [file Presentation6.zip › ERBB2/19ZY39353P.pdf]

Sequencing Depth

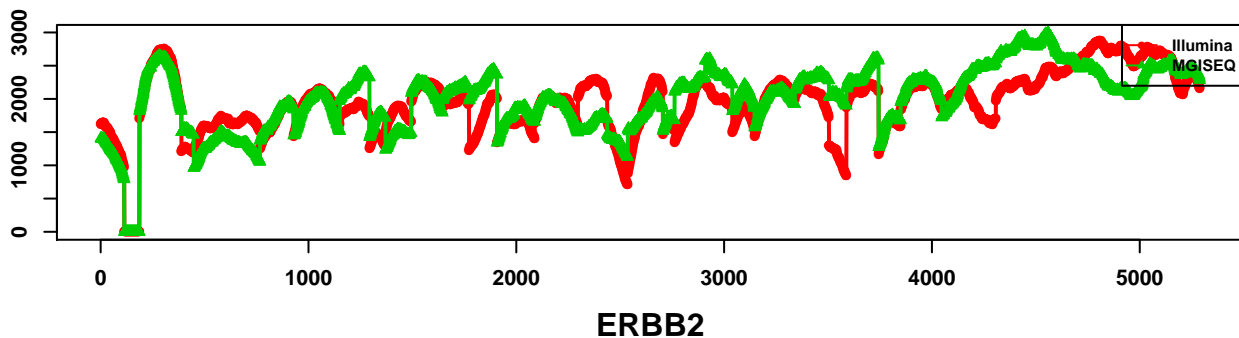

Sequencing Depth

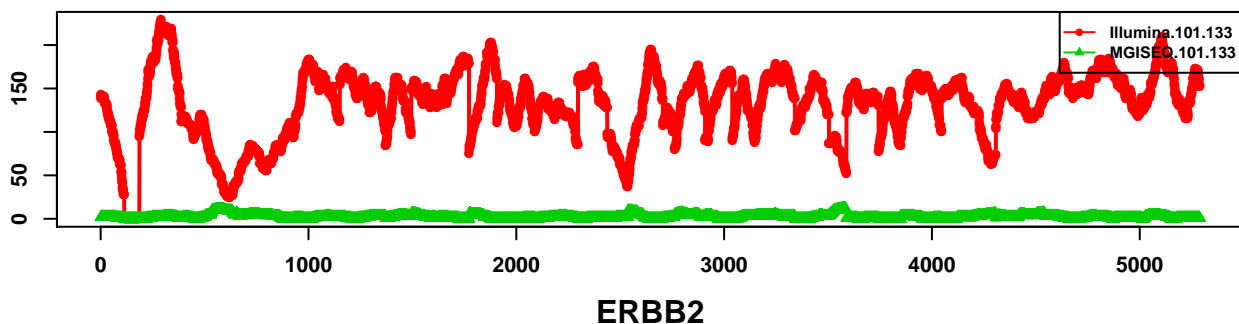

Sequencing Depth

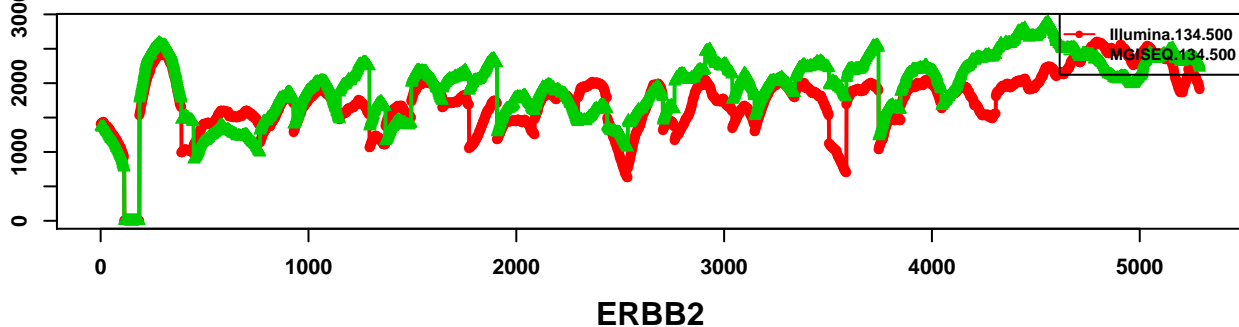

Supplement: Supplementary file 9 [file Presentation6.zip › ERBB2/19ZN12362B.pdf]

Sequencing Depth

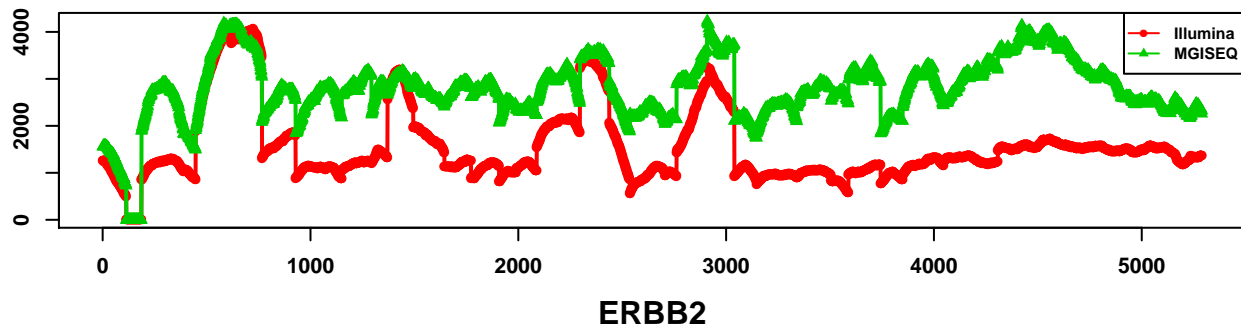

Sequencing Depth

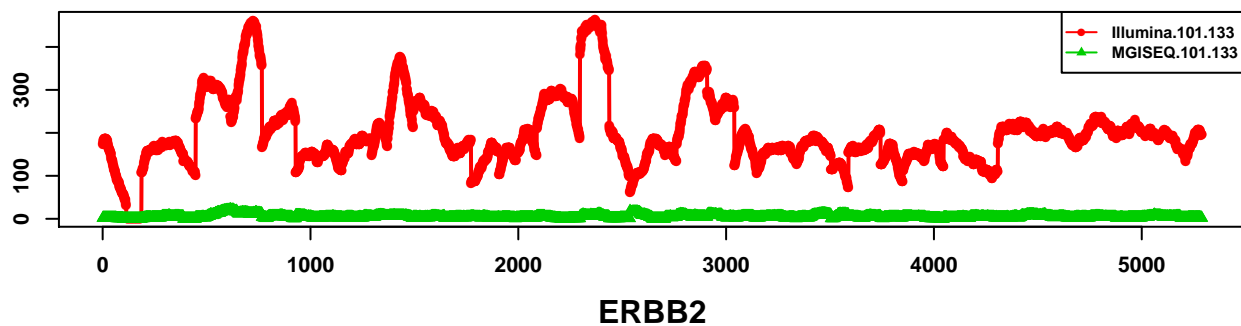

Sequencing Depth

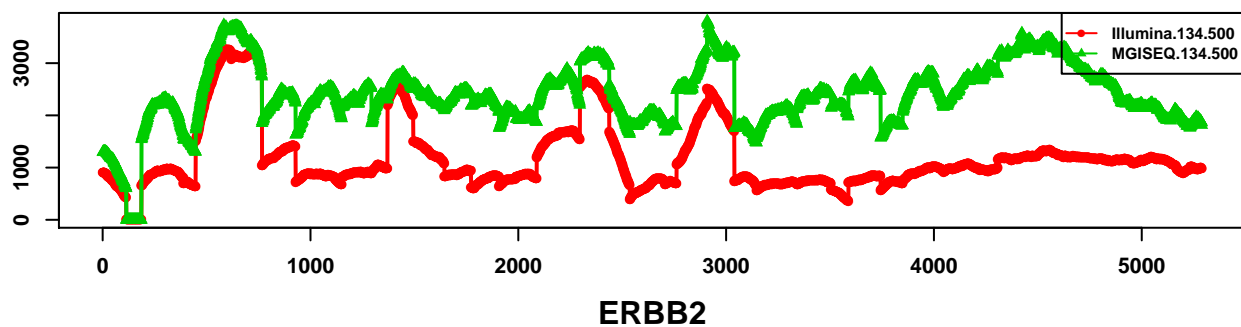

Supplement: Supplementary file 9 [file Presentation6.zip › ERBB2/19ZN12363F.pdf]

Sequencing Depth

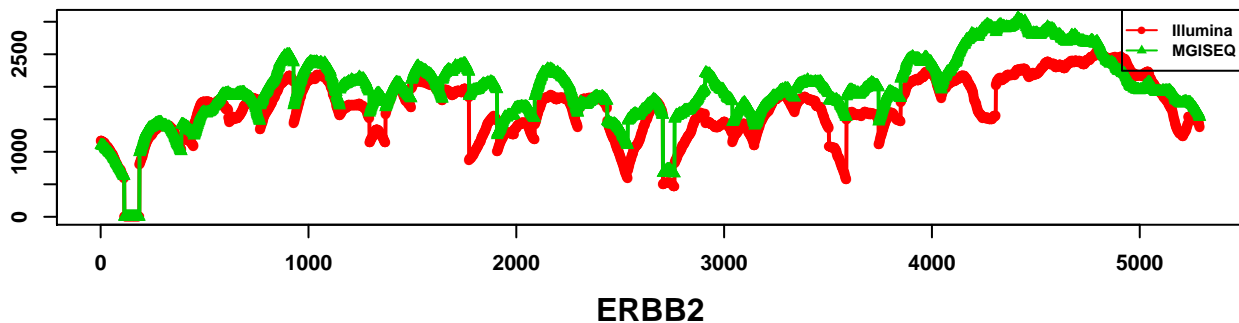

Sequencing Depth

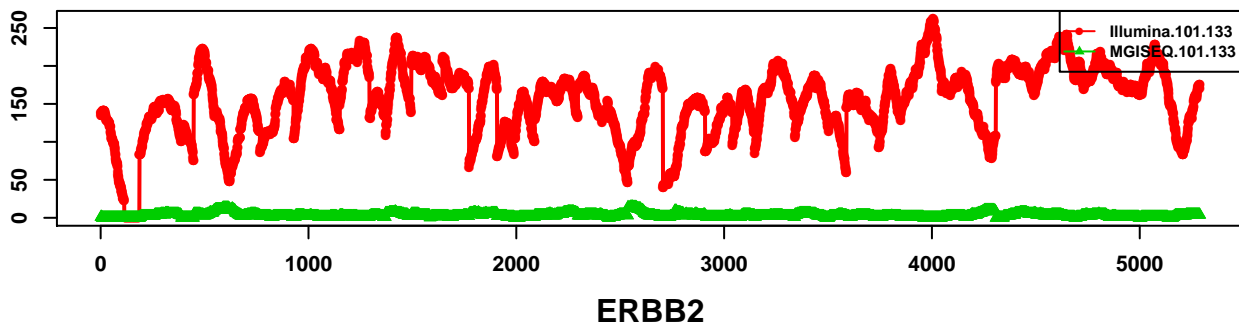

Sequencing Depth

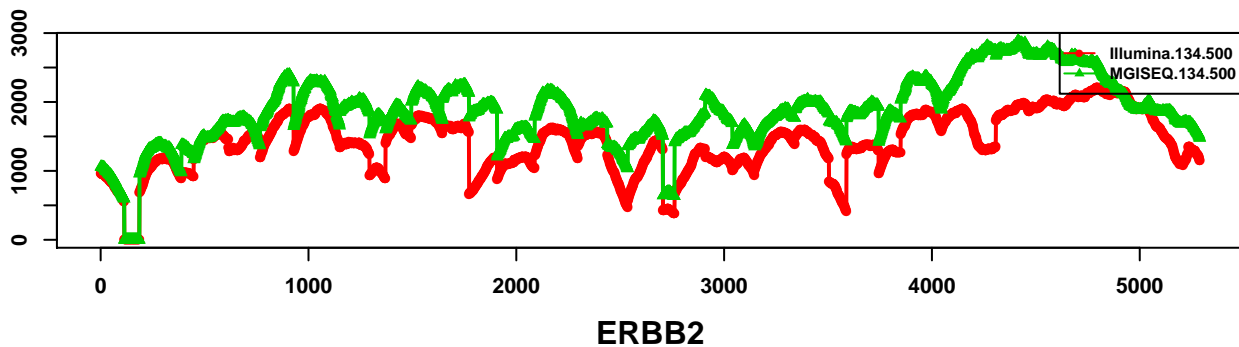

Supplement: Supplementary file 9 [file Presentation6.zip › ERBB2/19HE22134F.pdf]

Sequencing Depth

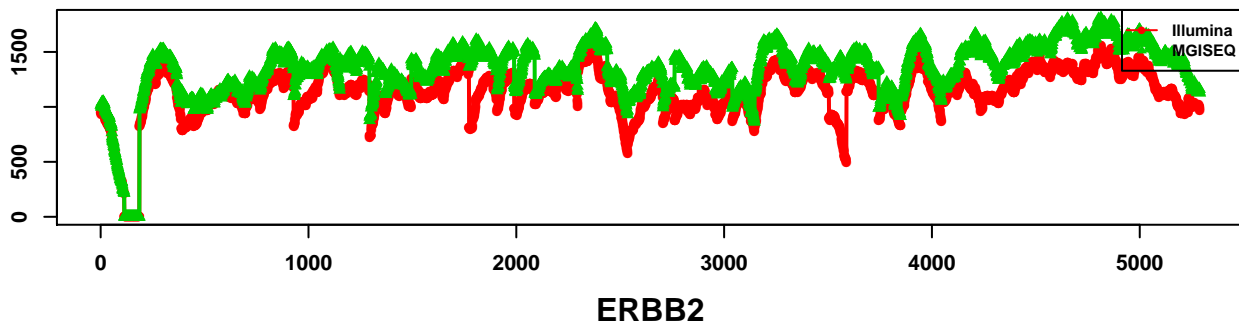

Sequencing Depth

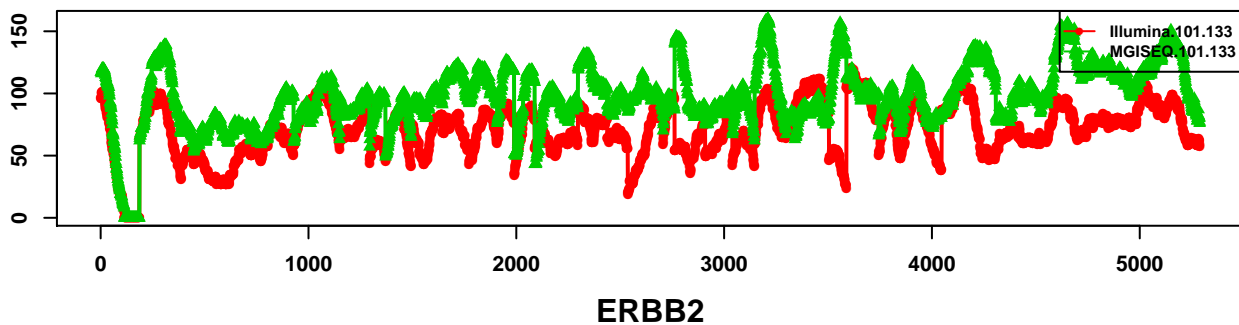

Sequencing Depth

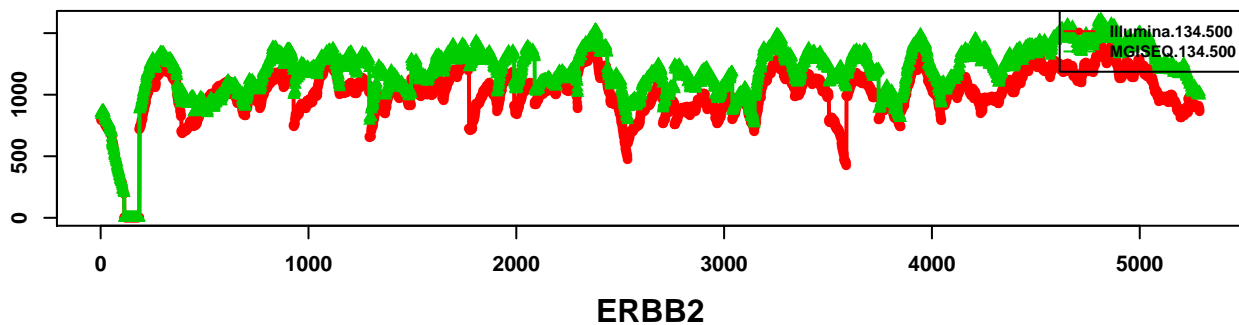

Supplement: Supplementary file 9 [file Presentation6.zip › ERBB2/19ZY39607P.pdf]

Sequencing Depth

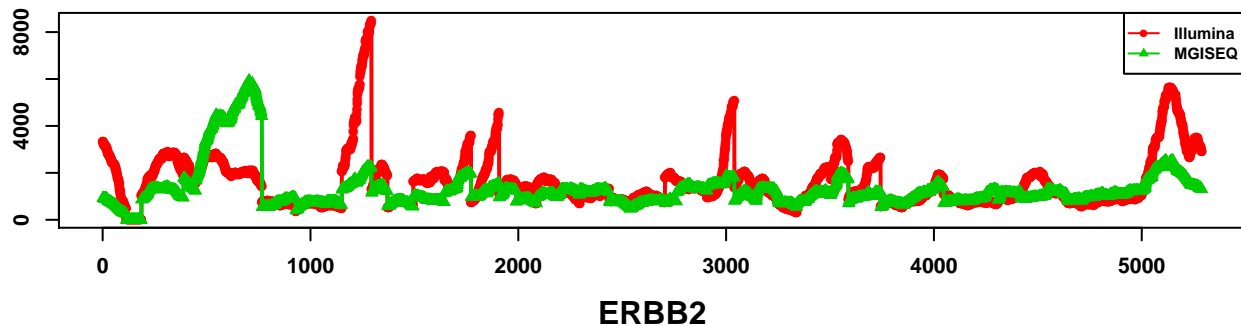

Sequencing Depth

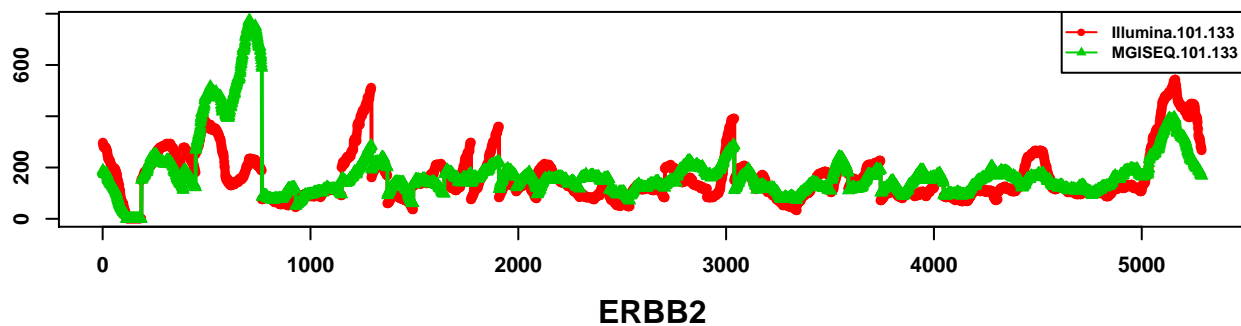

Sequencing Depth

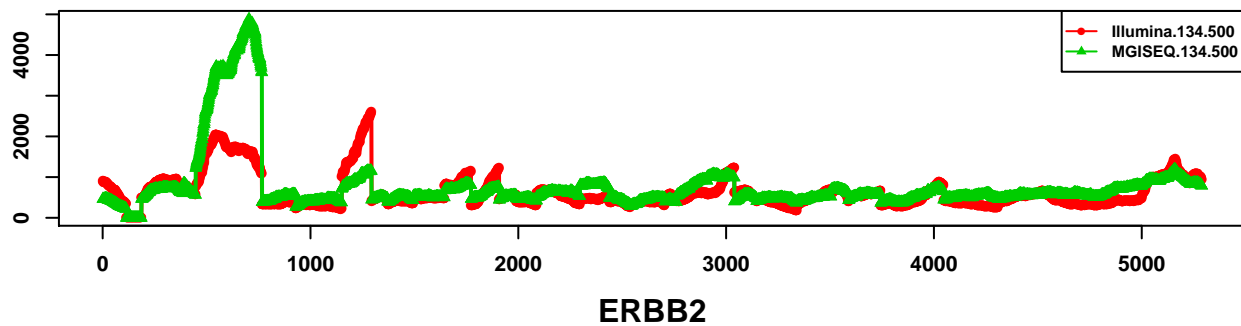

Supplement: Supplementary file 9 [file Presentation6.zip › ERBB2/19FC40415F.pdf]

Sequencing Depth

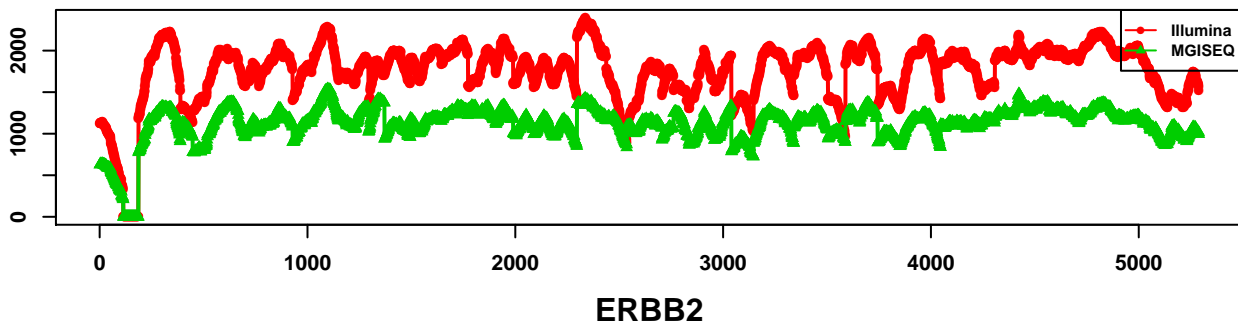

Sequencing Depth

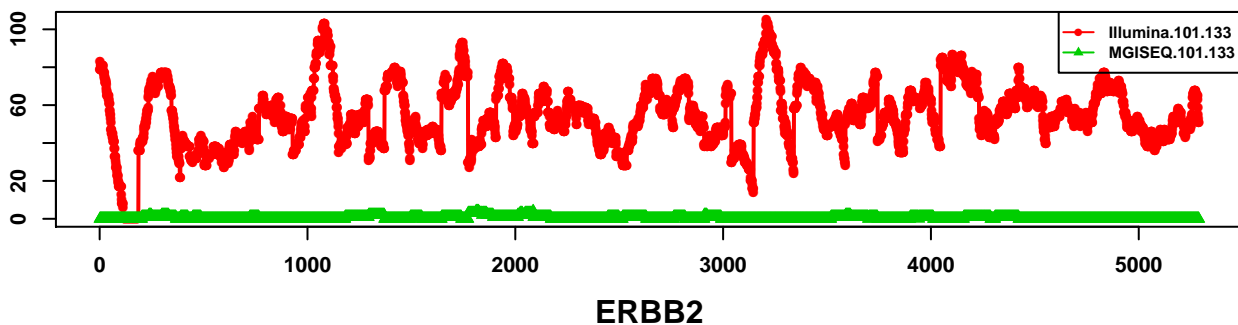

Sequencing Depth

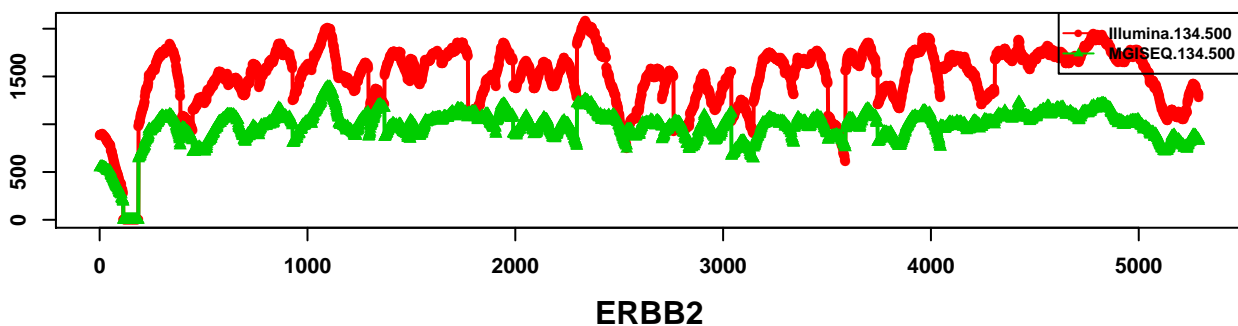

Supplement: Supplementary file 9 [file Presentation6.zip › ERBB2/19N01627-IIP.pdf]

Sequencing Depth

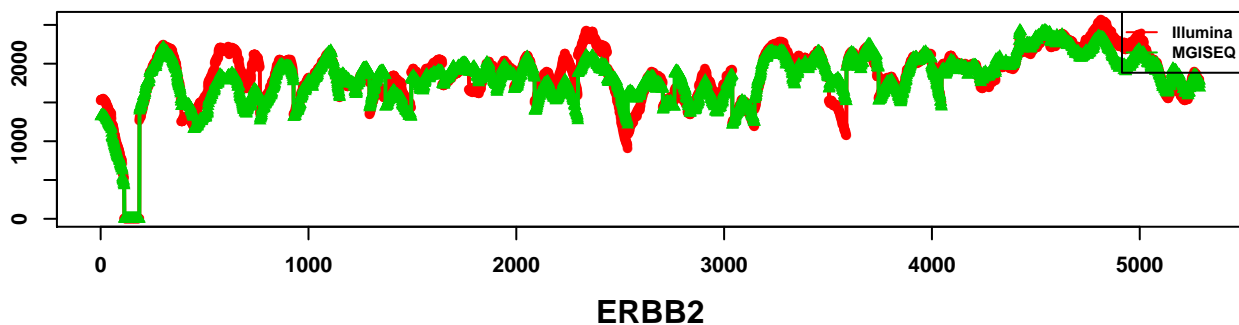

Sequencing Depth

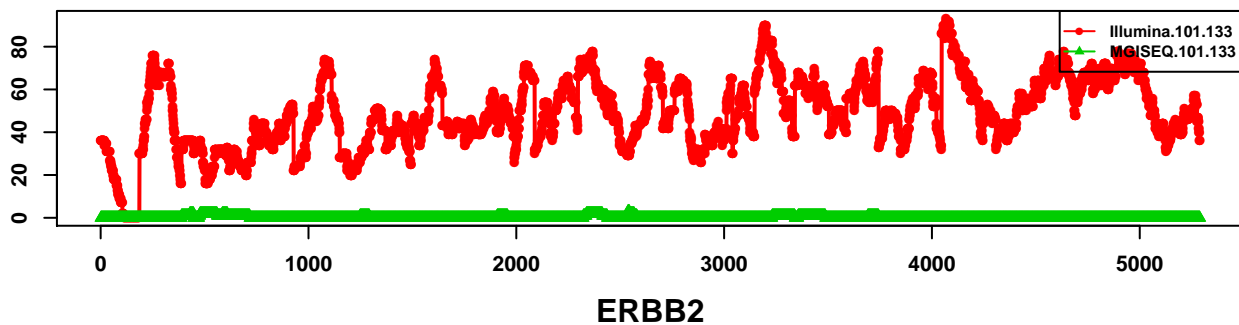

Sequencing Depth

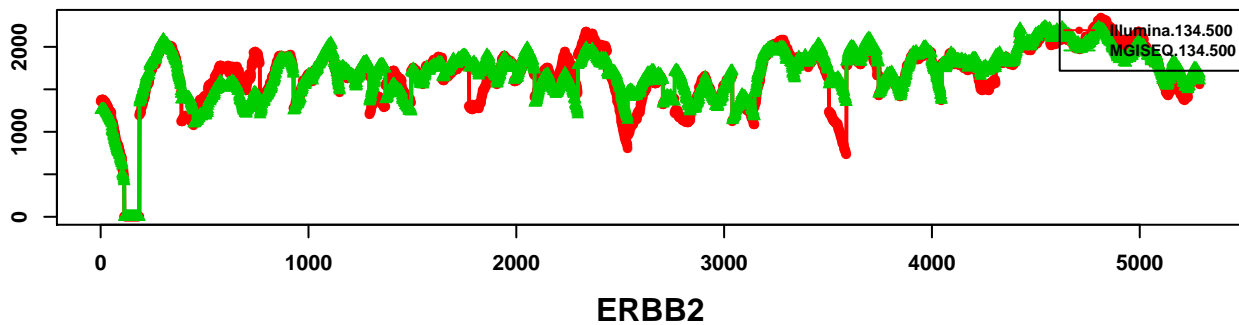

Supplement: Supplementary file 9 [file Presentation6.zip › ERBB2/19CF15528P.pdf]

Sequencing Depth

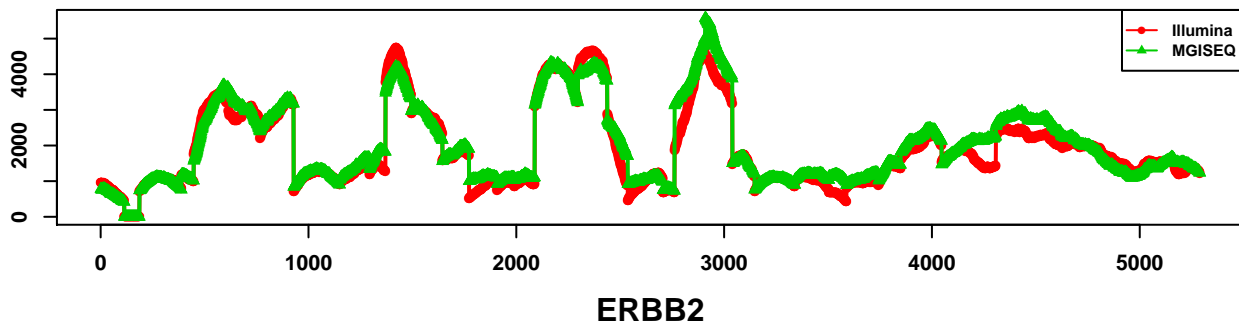

Sequencing Depth

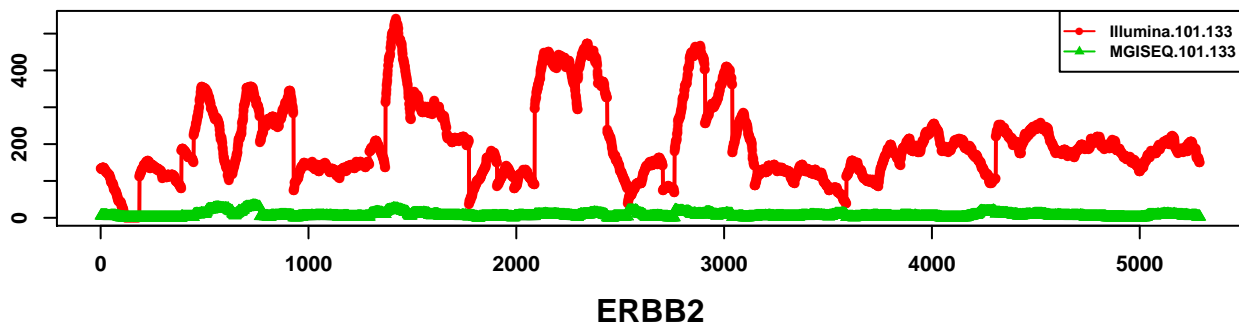

Sequencing Depth

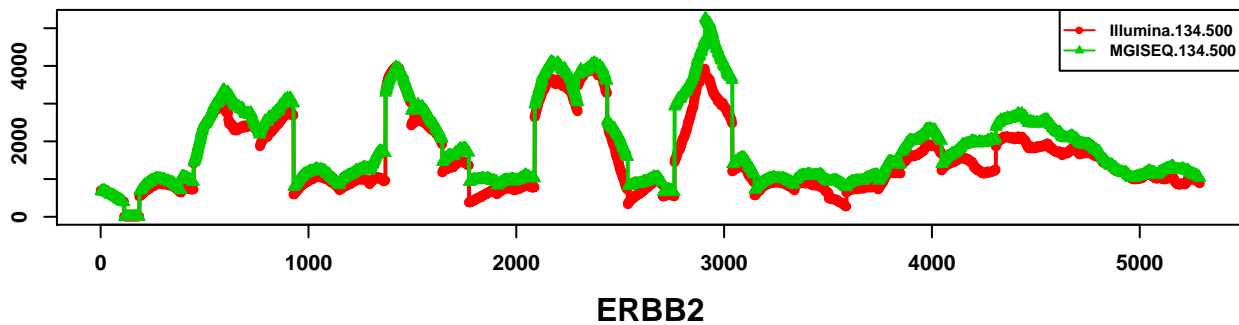

Supplement: Supplementary file 9 [file Presentation6.zip › ERBB2/19FC40245F.pdf]

Sequencing Depth

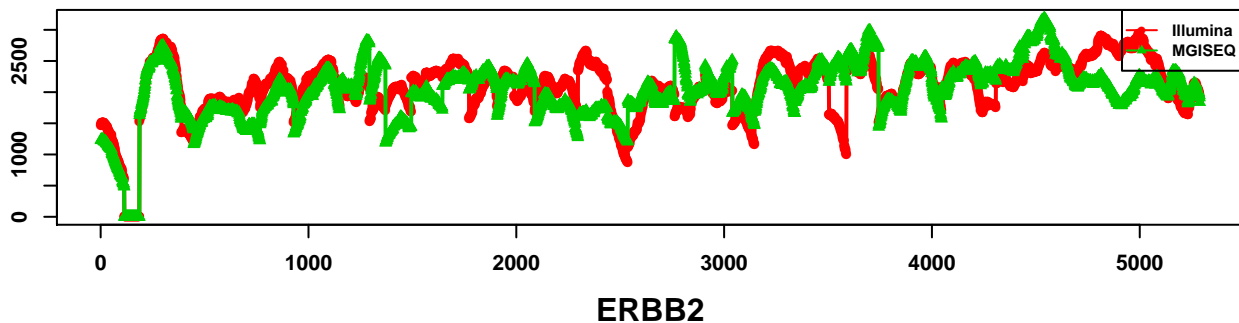

Sequencing Depth

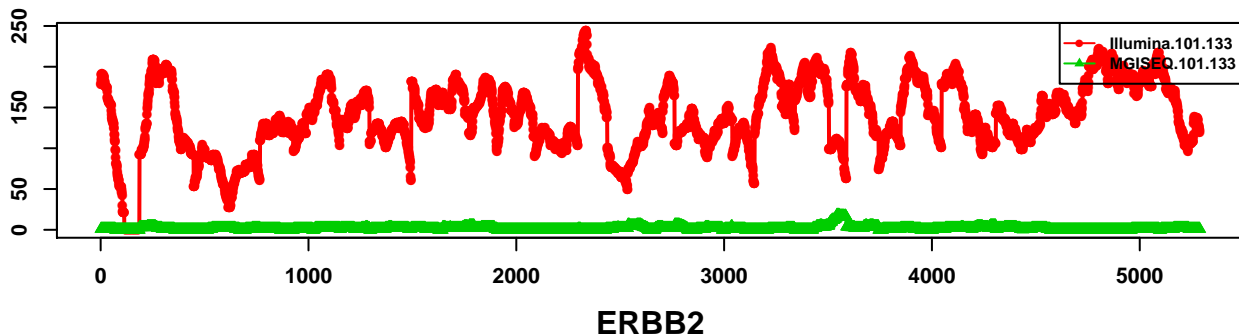

Sequencing Depth

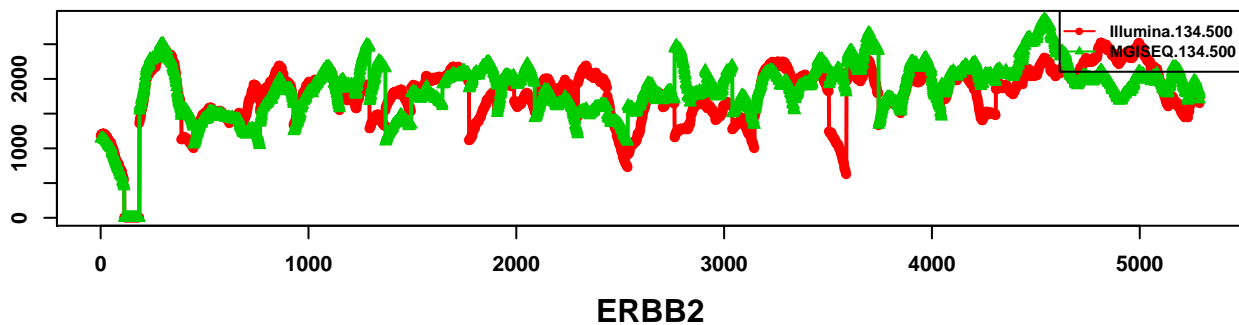

Supplement: Supplementary file 9 [file Presentation6.zip › ERBB2/19CF15529P.pdf]

Sequencing Depth

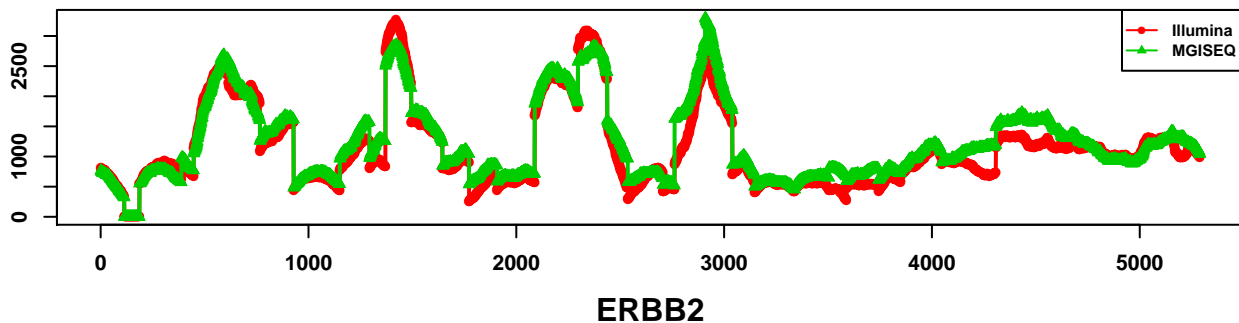

Sequencing Depth

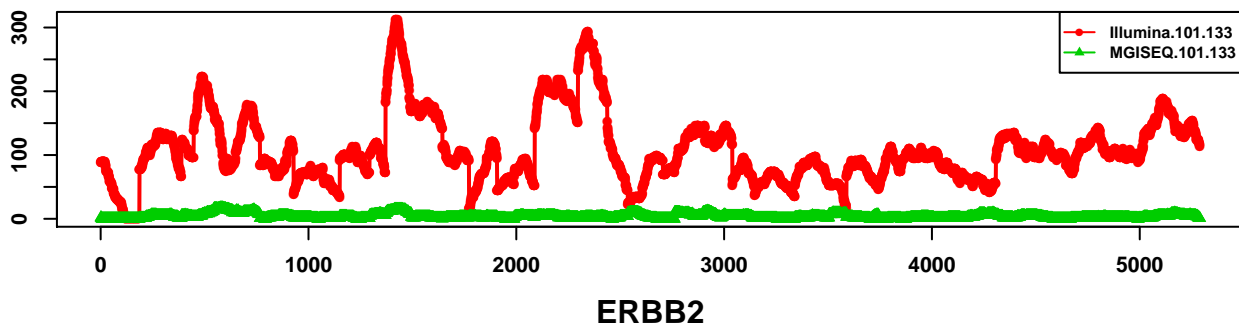

Sequencing Depth

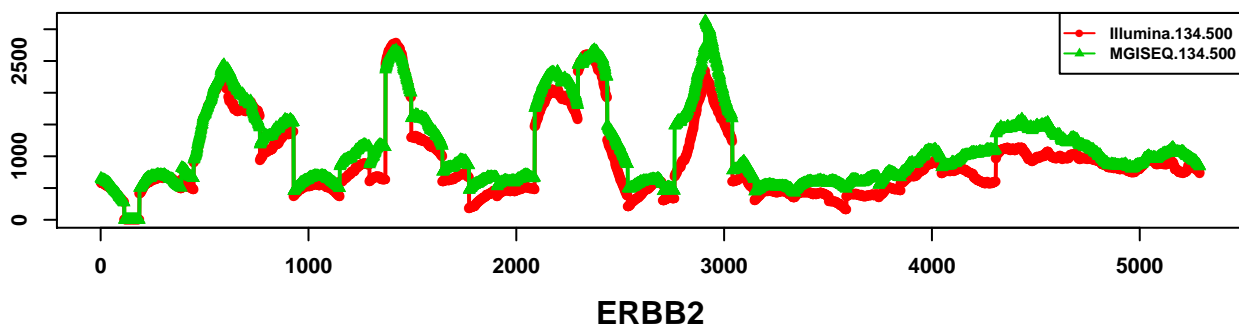

Supplement: Supplementary file 9 [file Presentation6.zip › ERBB2/19ZN12358F.pdf]

Sequencing Depth

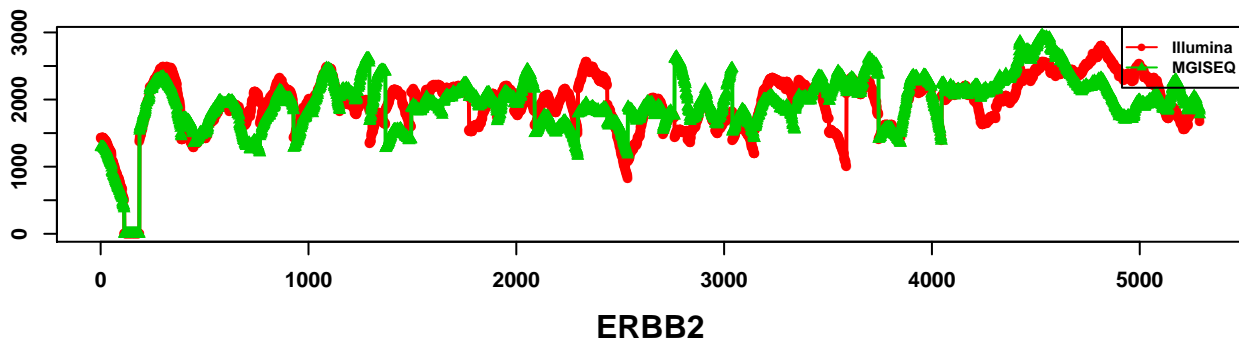

Sequencing Depth

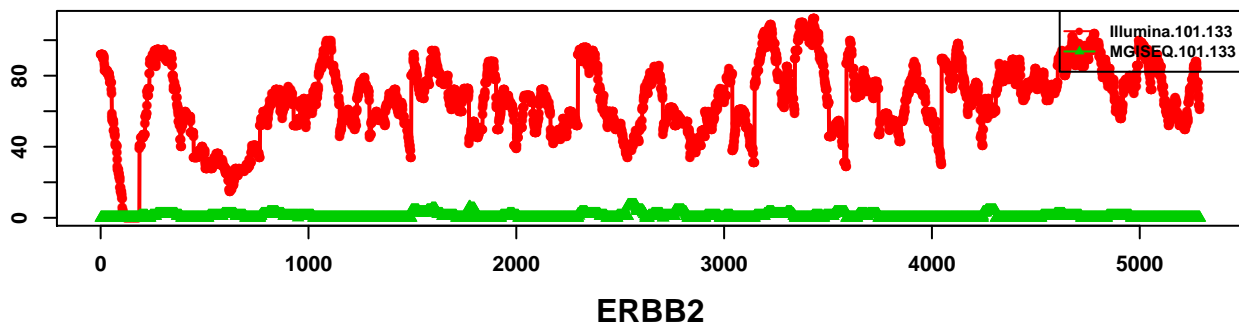

Sequencing Depth

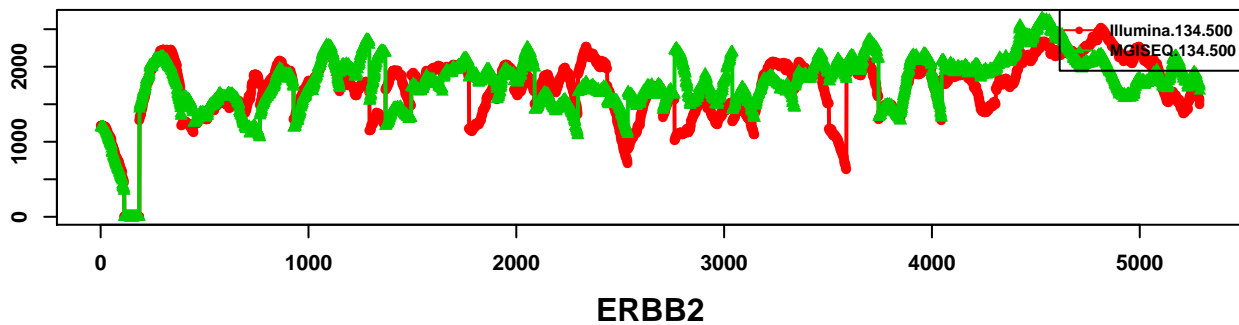

Supplement: Supplementary file 9 [file Presentation6.zip › ERBB2/19N01665P.pdf]

Sequencing Depth

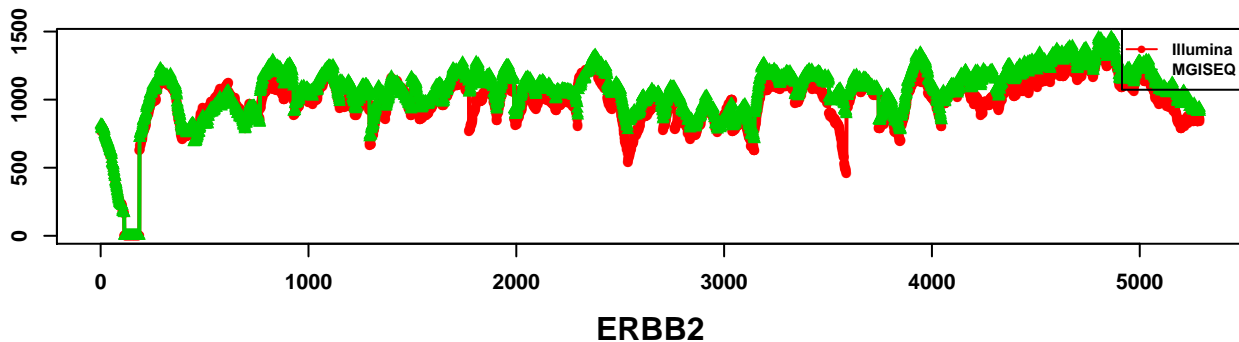

Sequencing Depth

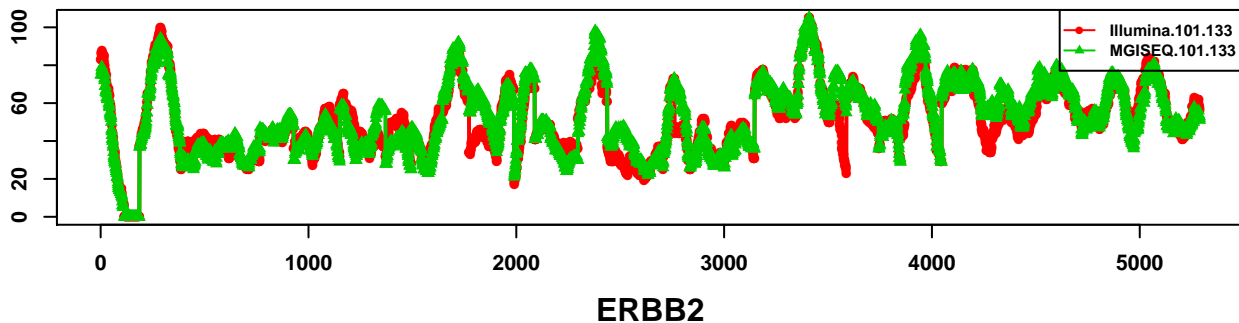

Sequencing Depth

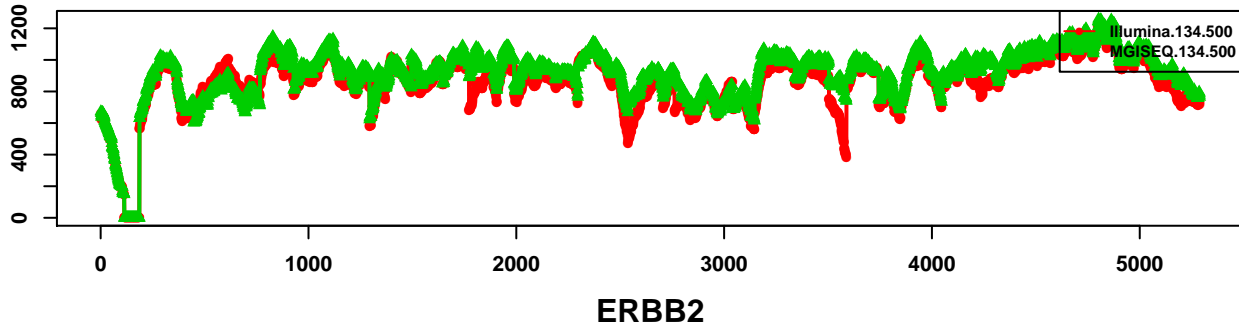

Supplement: Supplementary file 9 [file Presentation6.zip › ERBB2/19ZN12548P.pdf]

Sequencing Depth

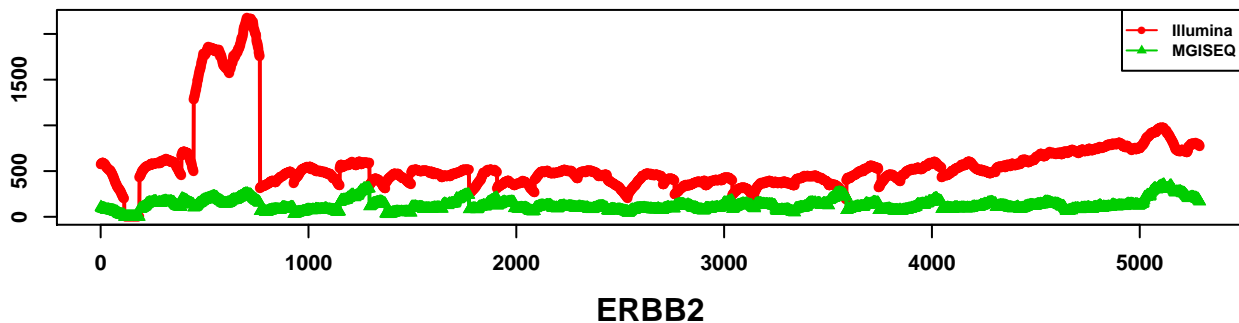

Sequencing Depth

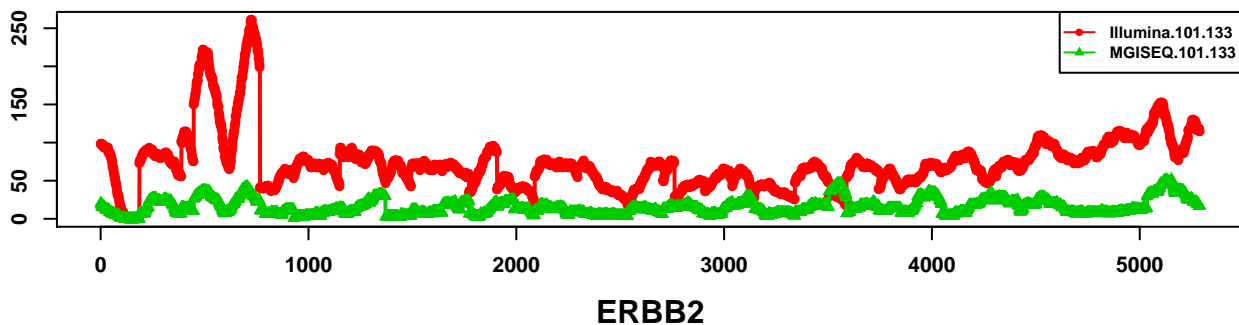

Sequencing Depth

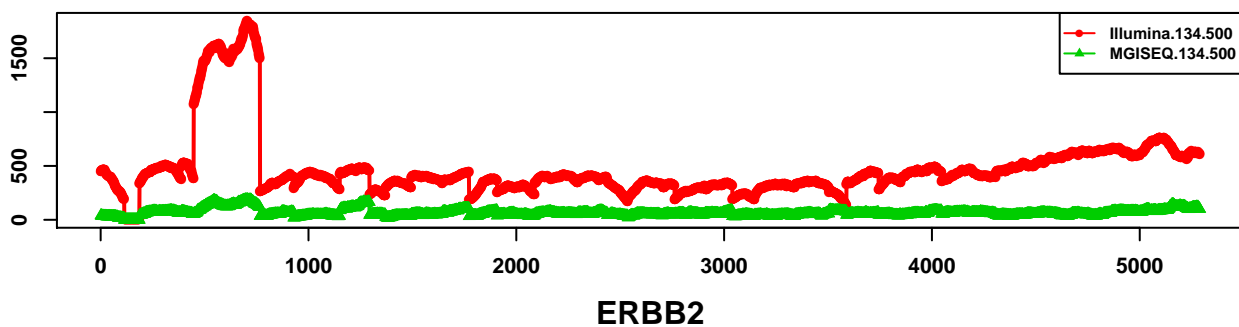

Supplement: Supplementary file 9 [file Presentation6.zip › ERBB2/19FC40438F.pdf]

Sequencing Depth

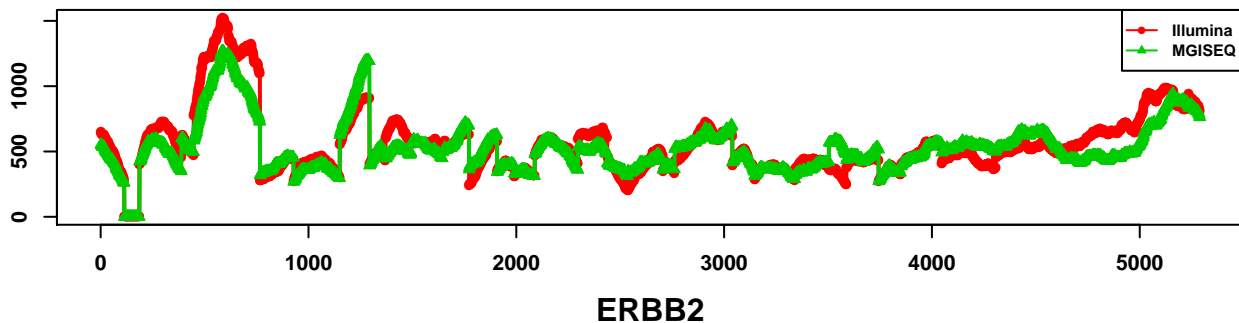

Sequencing Depth

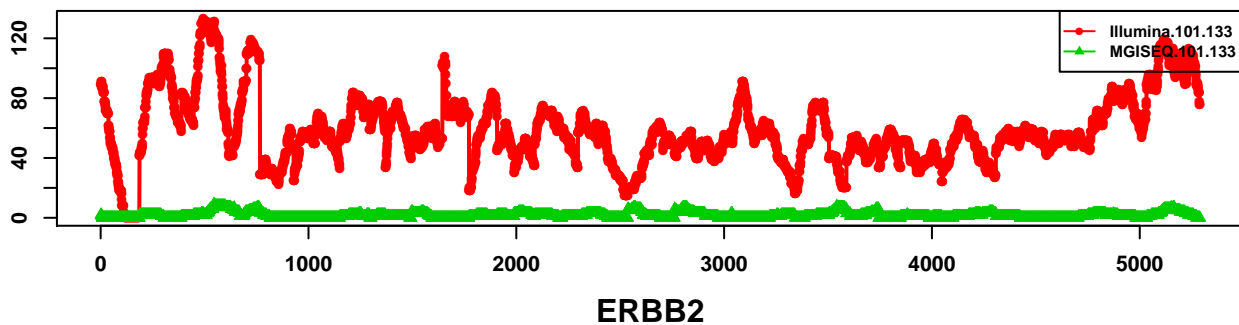

Sequencing Depth

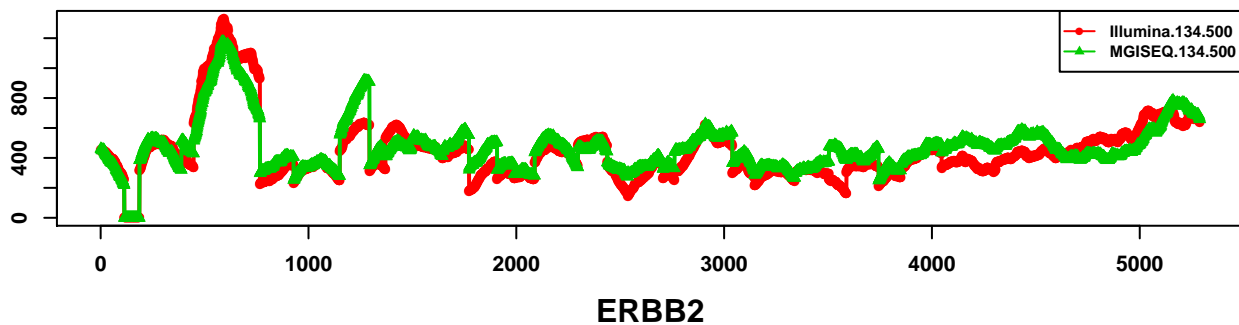

Supplement: Supplementary file 9 [file Presentation6.zip › ERBB2/19HS86178F.pdf]

Sequencing Depth

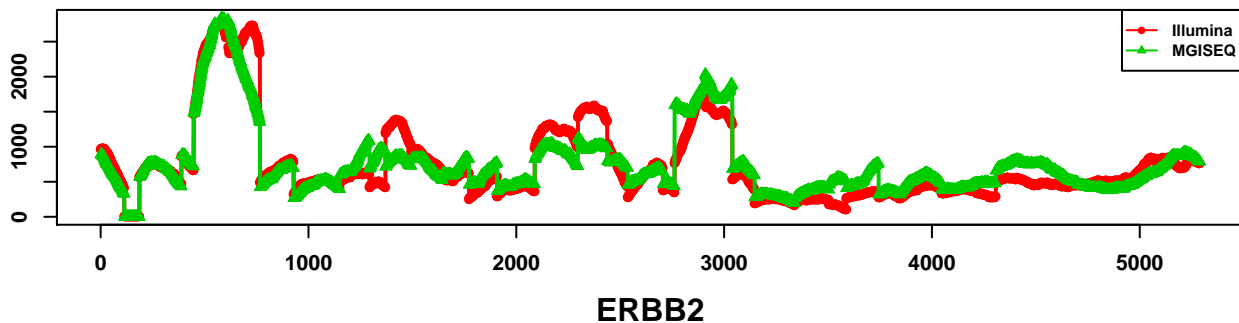

Sequencing Depth

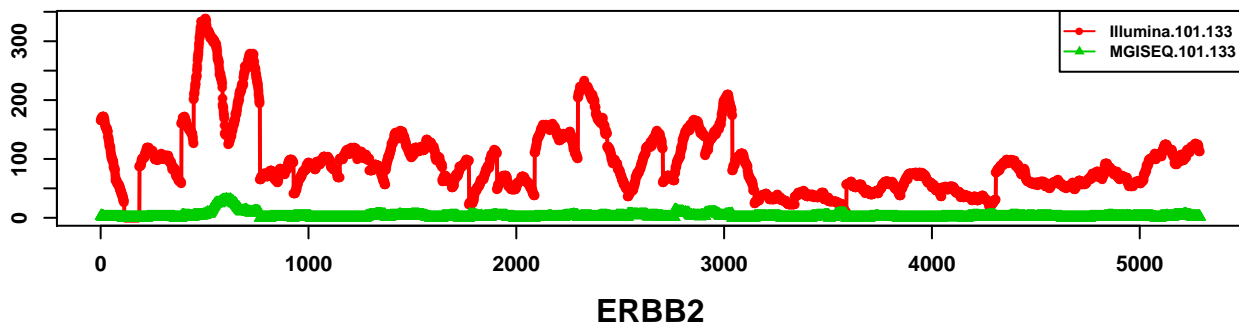

Sequencing Depth

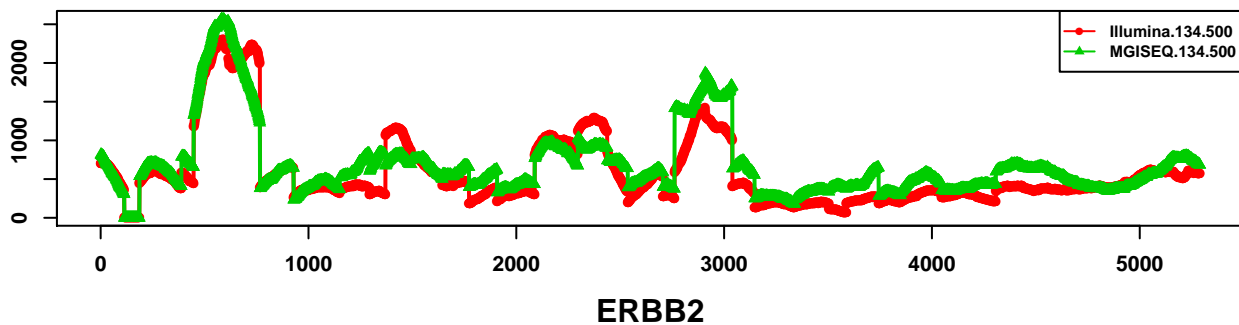

Supplement: Supplementary file 9 [file Presentation6.zip › ERBB2/19N01673F.pdf]

Sequencing Depth

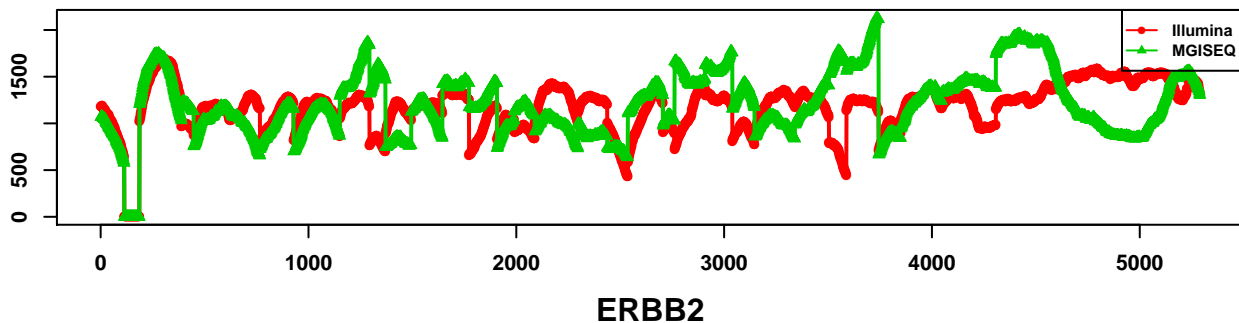

Sequencing Depth

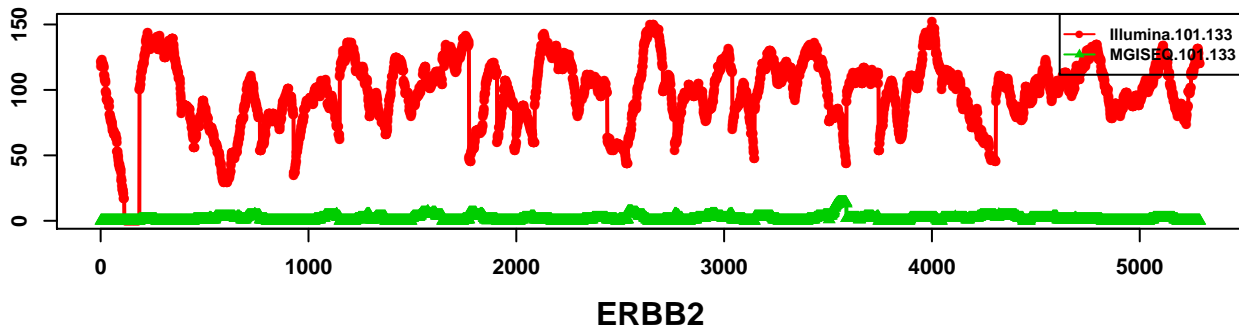

Sequencing Depth

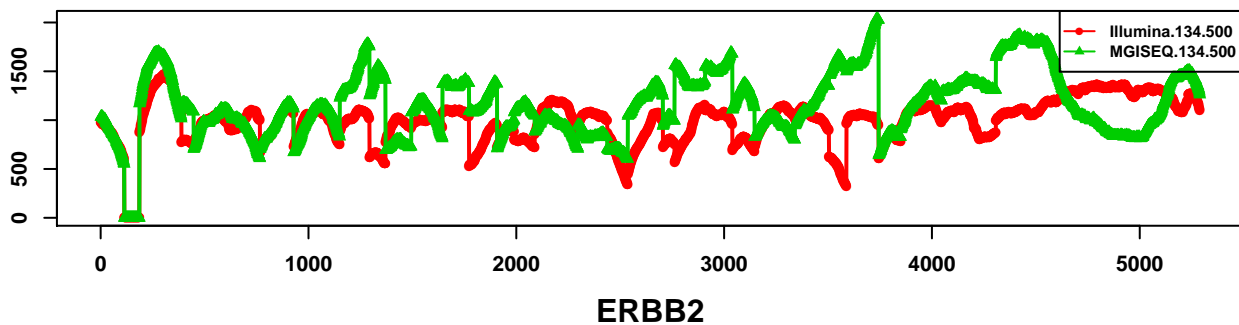

Supplement: Supplementary file 9 [file Presentation6.zip › ERBB2/19HE22162F.pdf]

Sequencing Depth

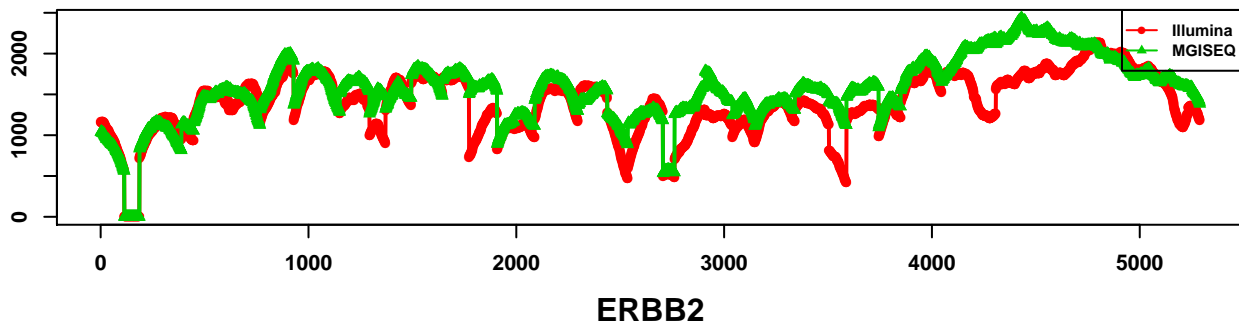

Sequencing Depth

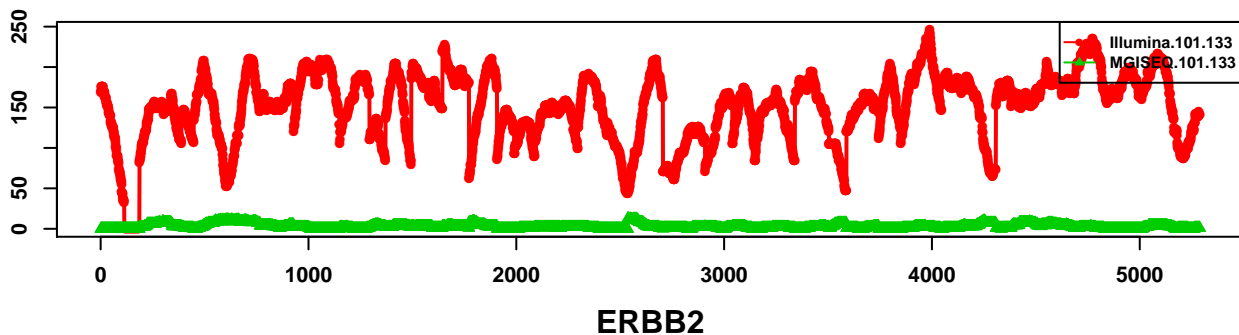

Sequencing Depth

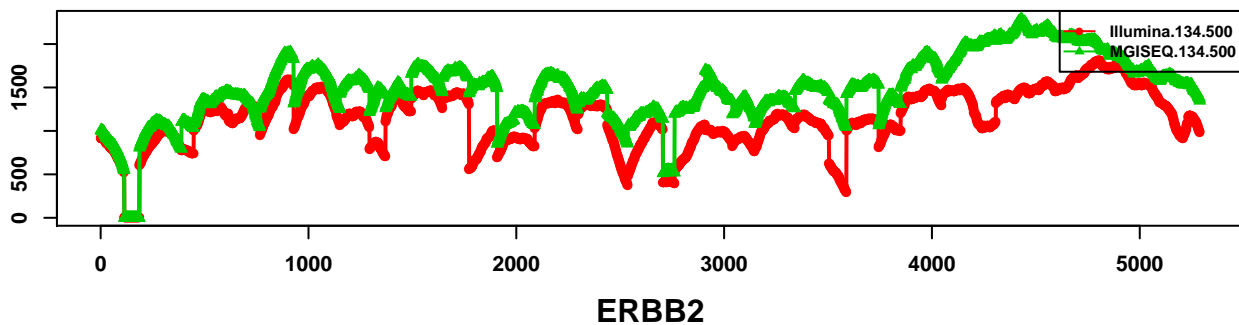

Supplement: Supplementary file 9 [file Presentation6.zip › ERBB2/19HE22135F.pdf]

Sequencing Depth

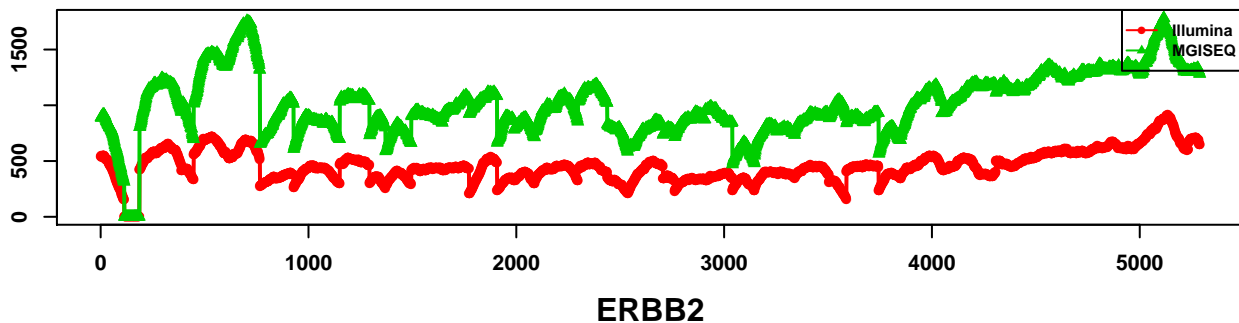

Sequencing Depth

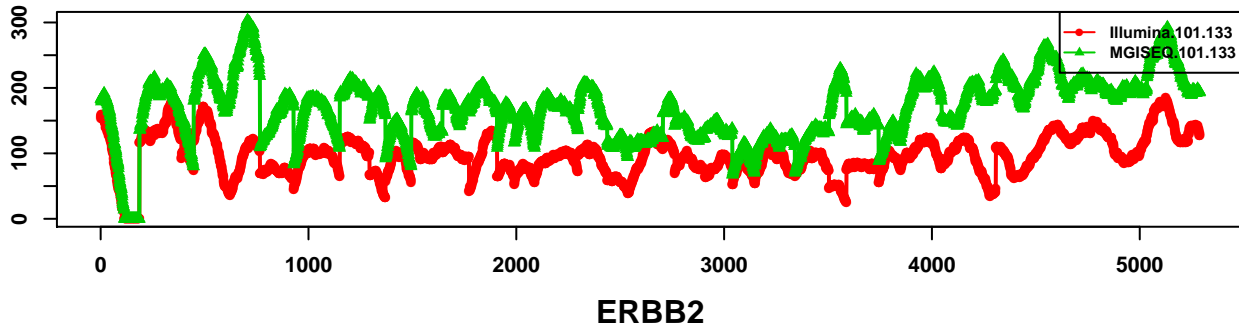

Sequencing Depth

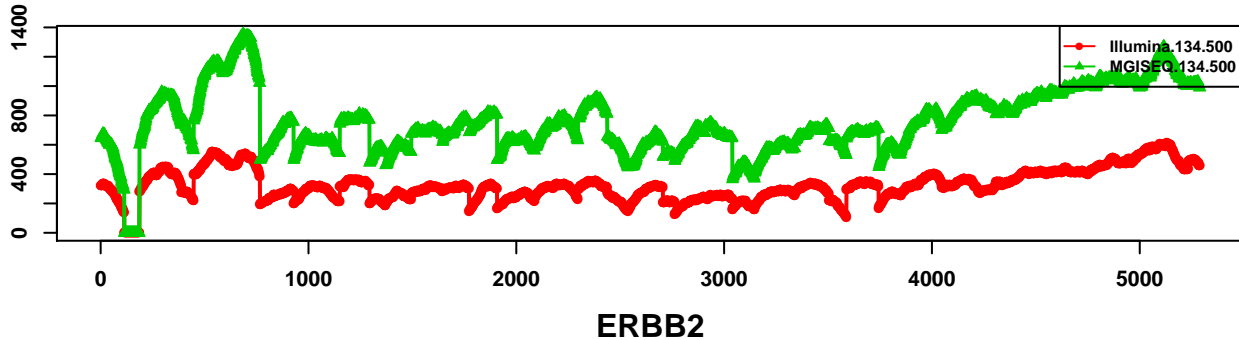

Supplement: Supplementary file 9 [file Presentation6.zip › ERBB2/19JS48274F.pdf]

Sequencing Depth

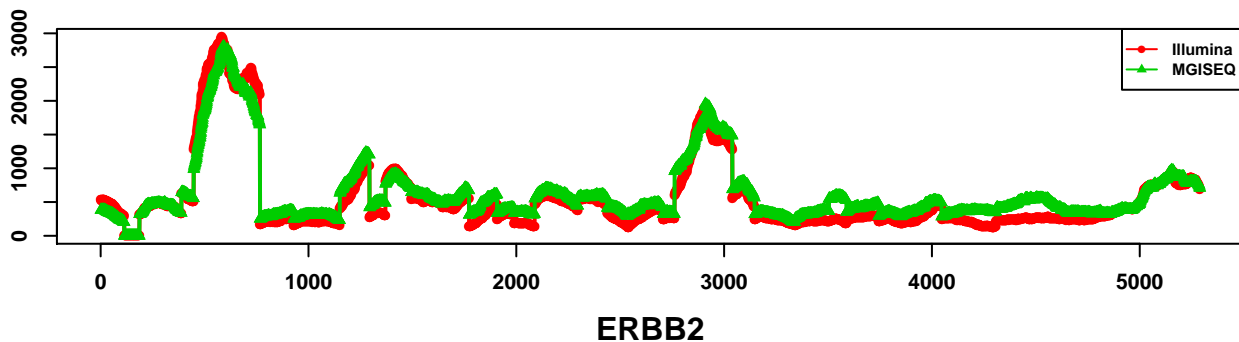

Sequencing Depth

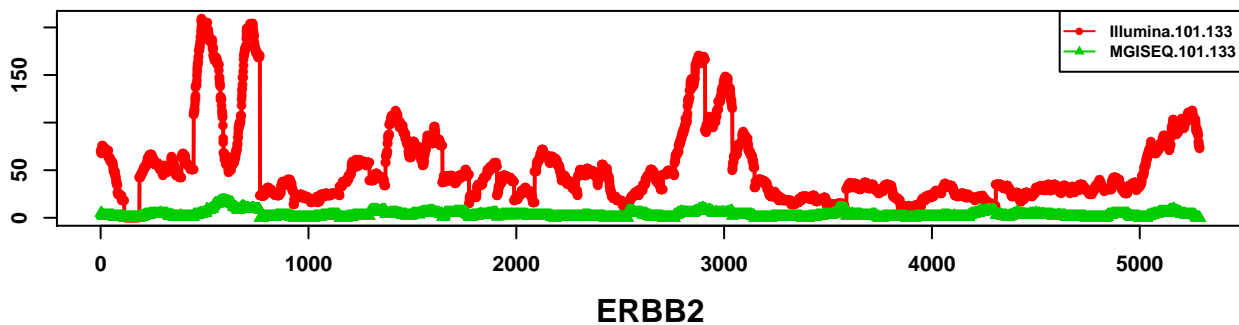

Sequencing Depth

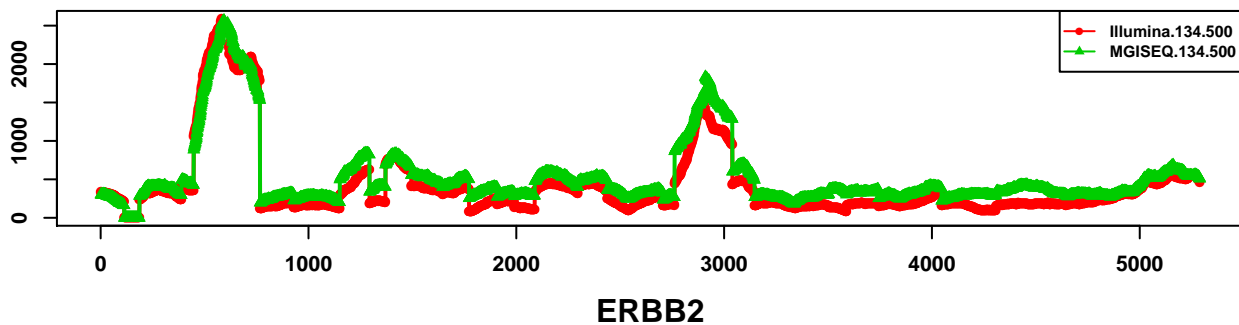

Supplement: Supplementary file 9 [file Presentation6.zip › ERBB2/19FC40249F.pdf]

Sequencing Depth

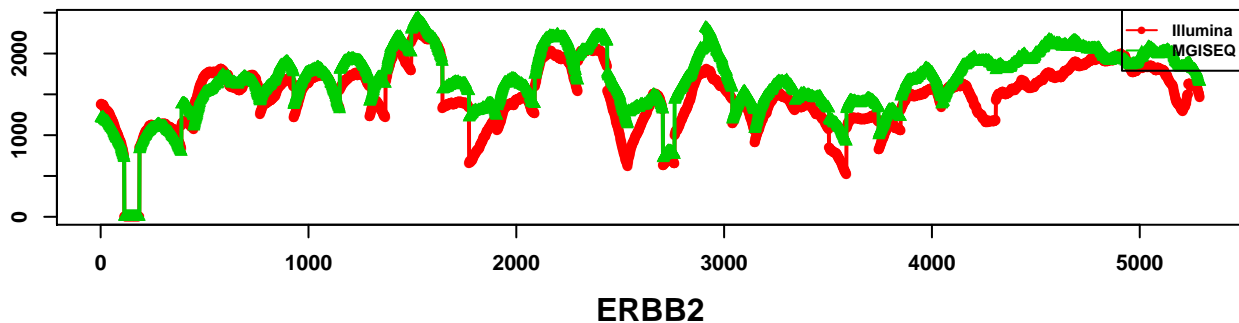

Sequencing Depth

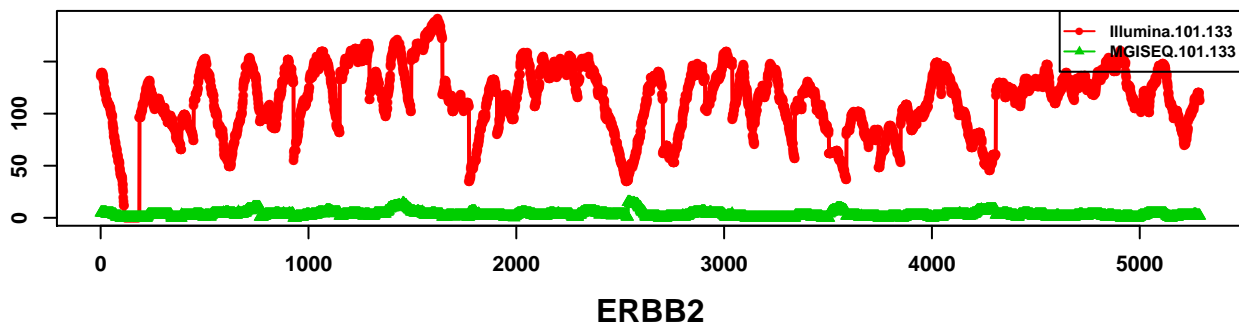

Sequencing Depth

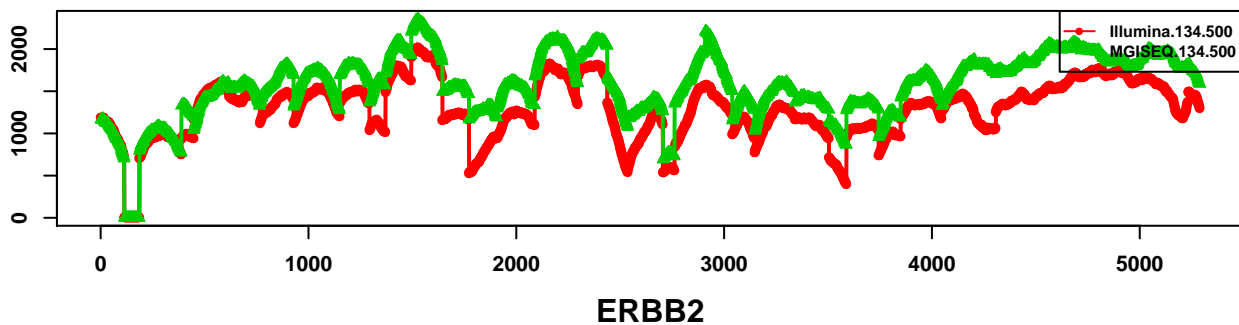

Supplement: Supplementary file 9 [file Presentation6.zip › ERBB2/19HE22103F.pdf]

Sequencing Depth

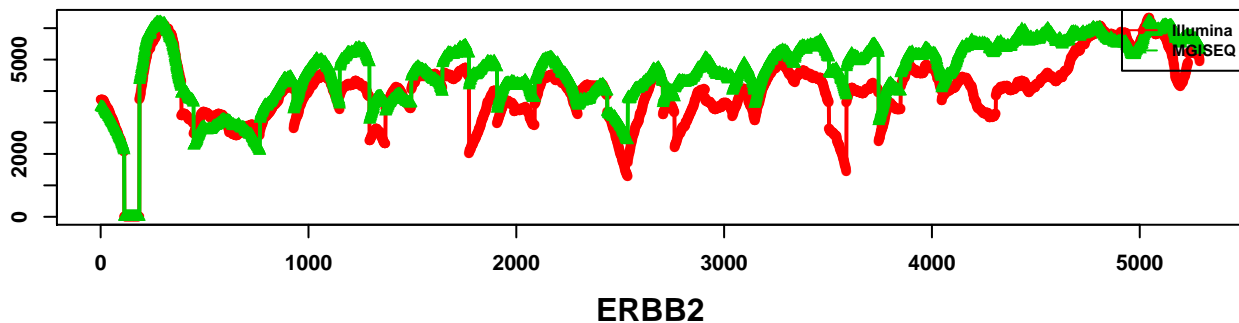

Sequencing Depth

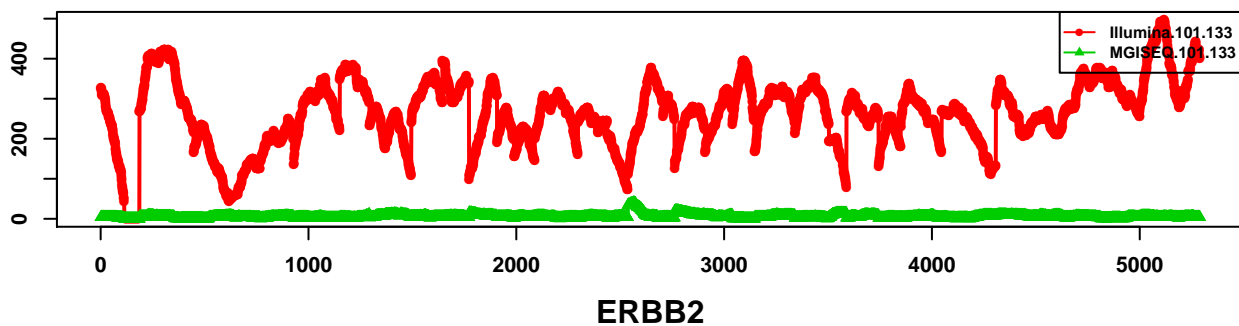

Sequencing Depth

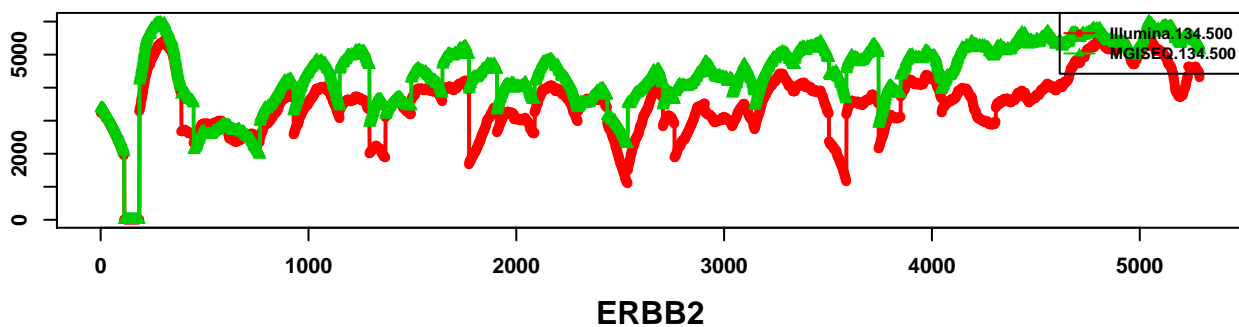

Supplement: Supplementary file 9 [file Presentation6.zip › ERBB2/19GY94041T.pdf]

Sequencing Depth

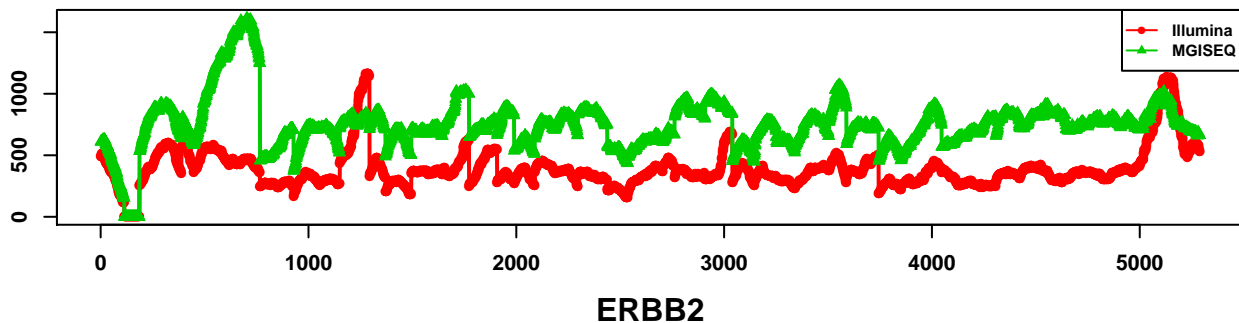

Sequencing Depth

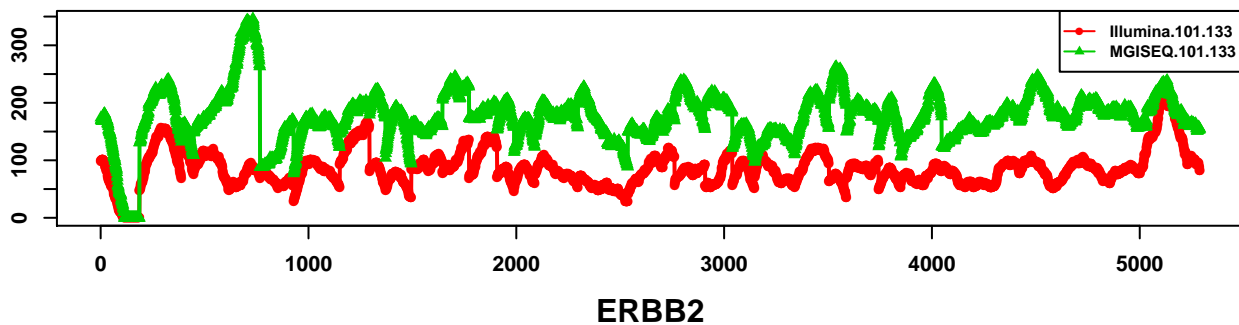

Sequencing Depth

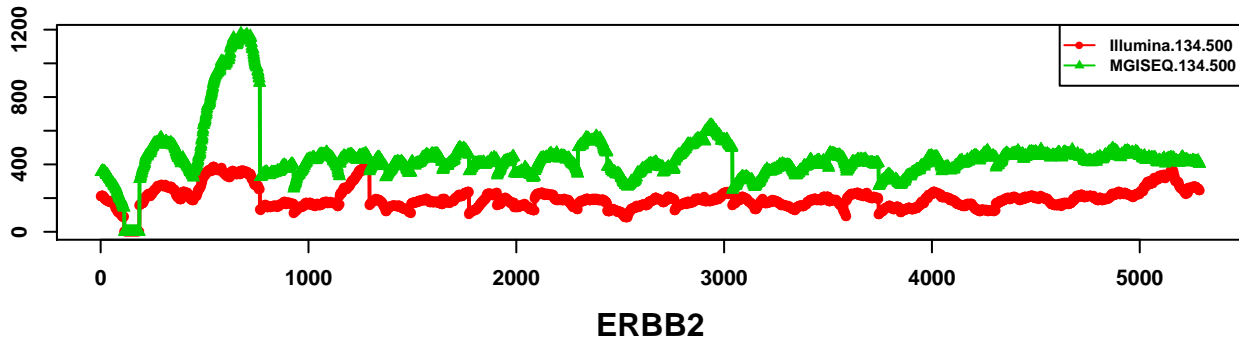

Supplement: Supplementary file 9 [file Presentation6.zip › ERBB2/19CF15765F.pdf]

Sequencing Depth

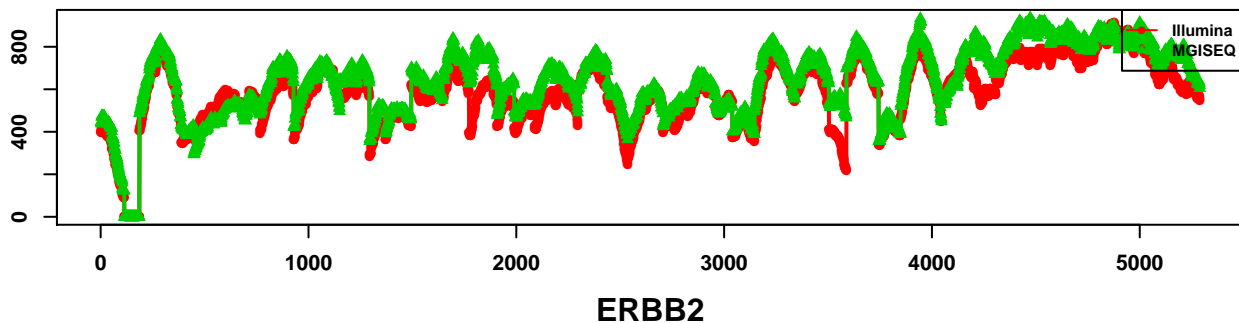

Sequencing Depth

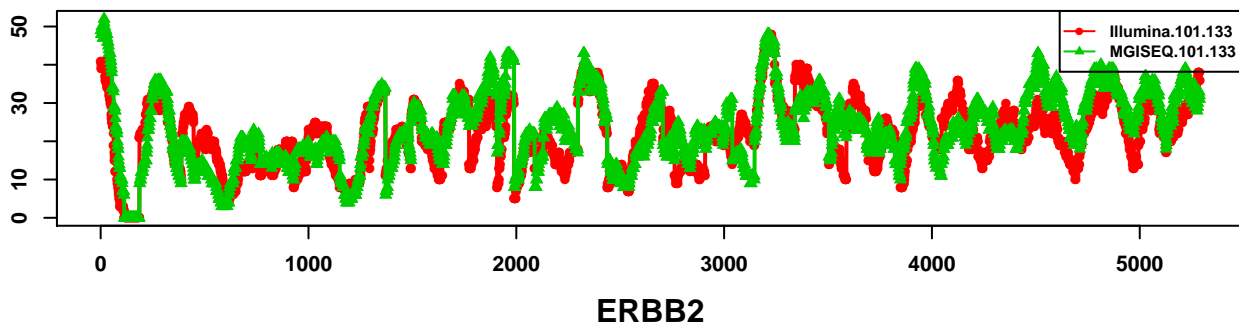

Sequencing Depth

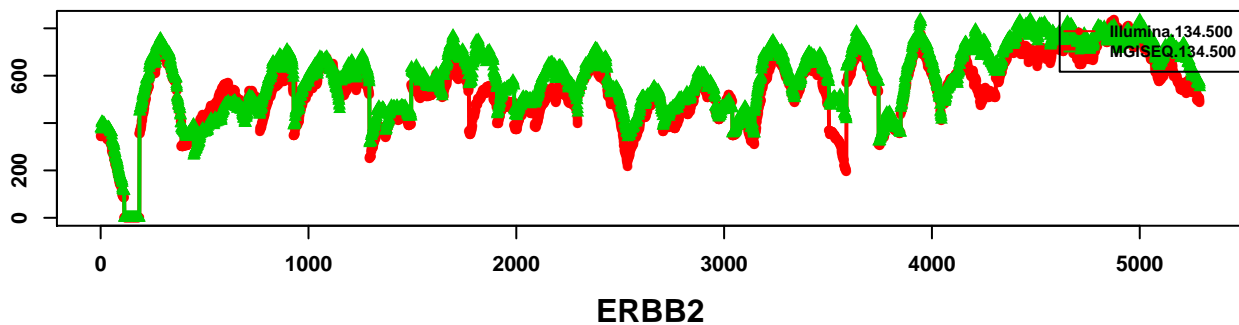

Supplement: Supplementary file 9 [file Presentation6.zip › ERBB2/M1901246P.pdf]

Sequencing Depth

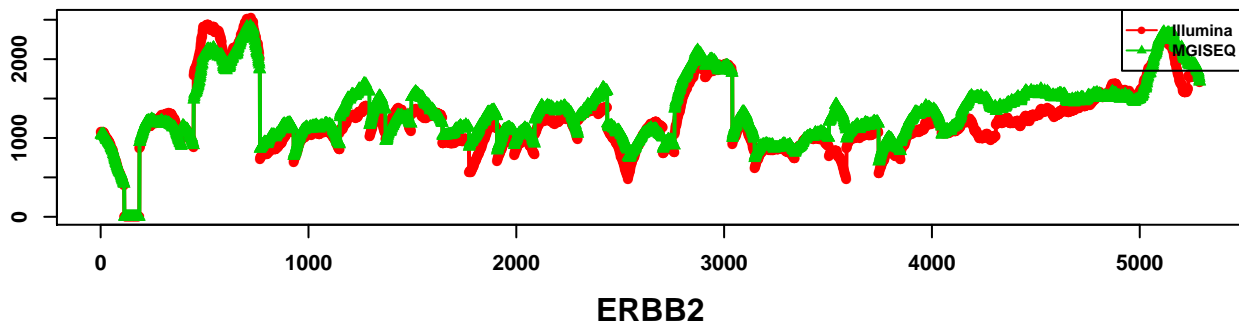

Sequencing Depth

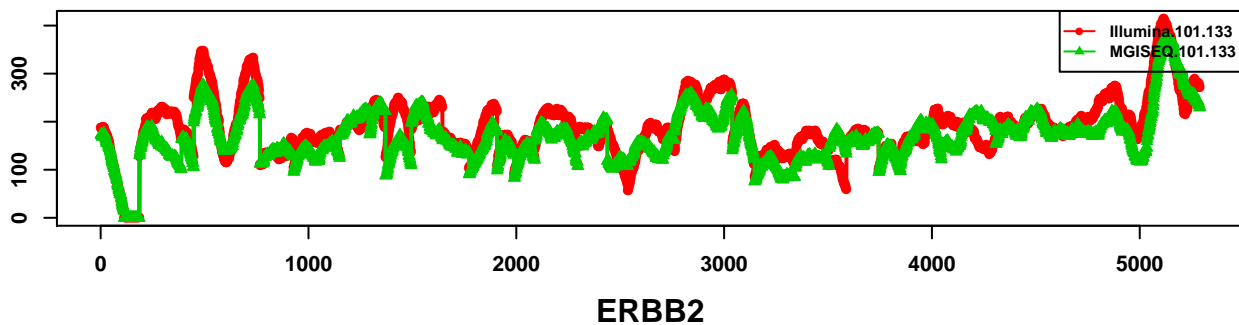

Sequencing Depth

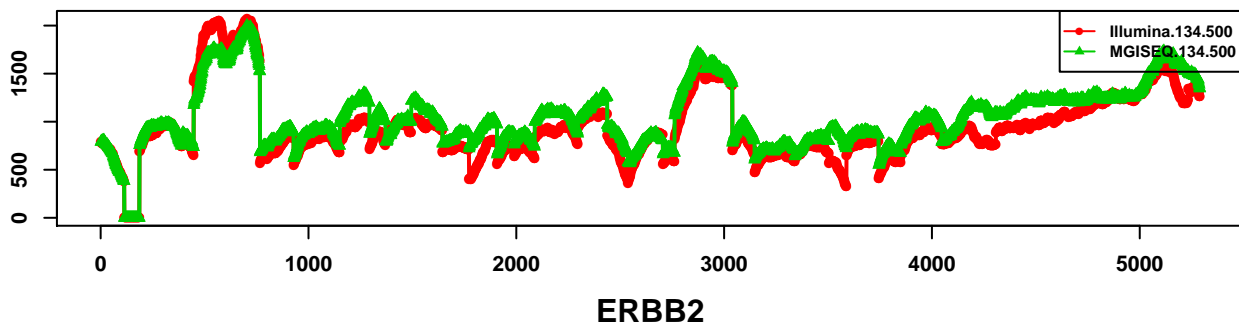

Supplement: Supplementary file 9 [file Presentation6.zip › ERBB2/19Q06298F.pdf]

Sequencing Depth

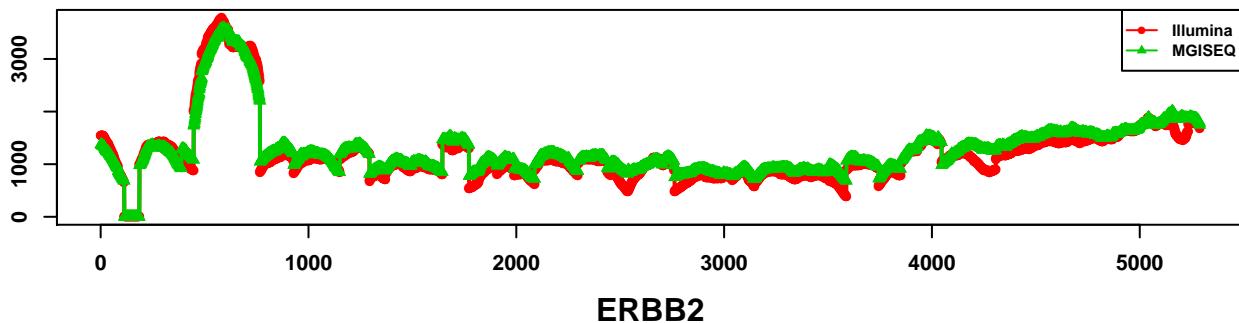

Sequencing Depth

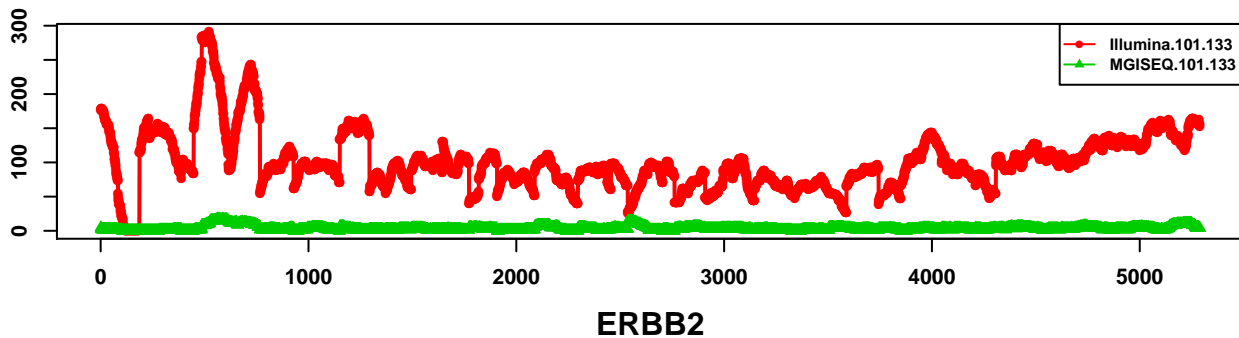

Sequencing Depth

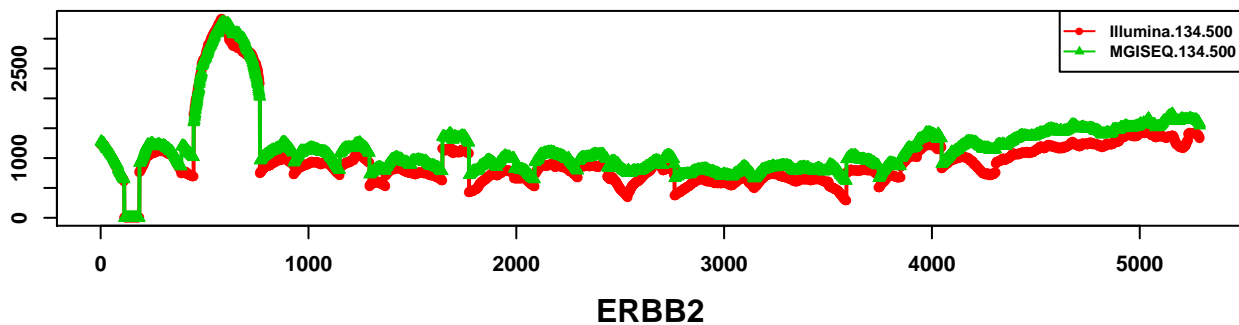

Supplement: Supplementary file 9 [file Presentation6.zip › ERBB2/19HE22050F.pdf]

Sequencing Depth

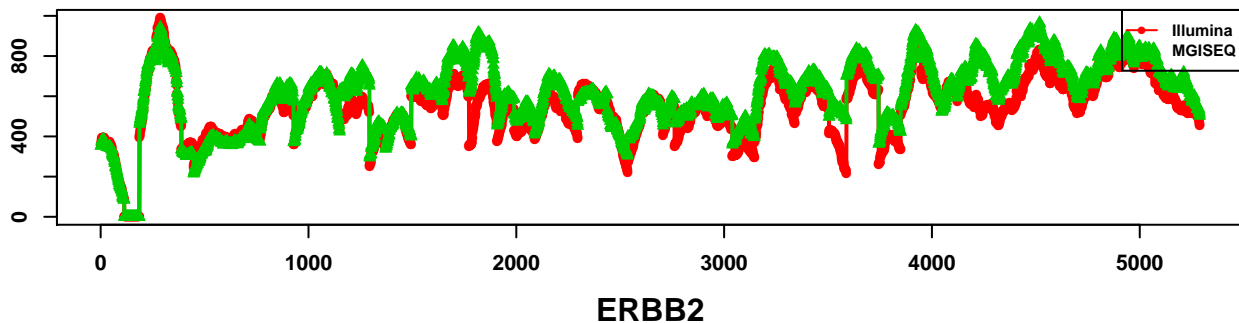

Sequencing Depth

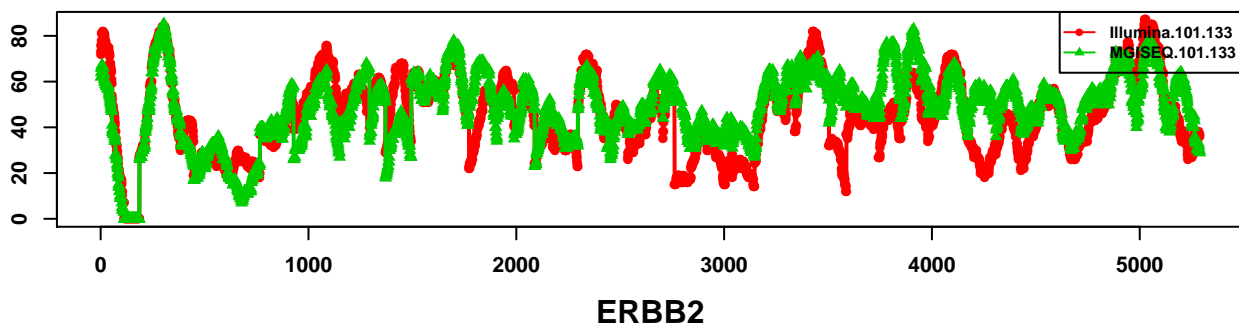

Sequencing Depth

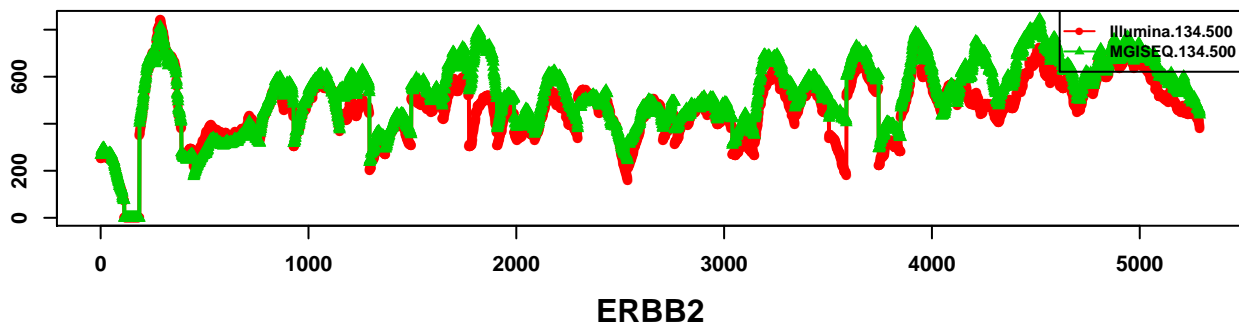

Supplement: Supplementary file 9 [file Presentation6.zip › ERBB2/ZK190805-CF.pdf]

Sequencing Depth

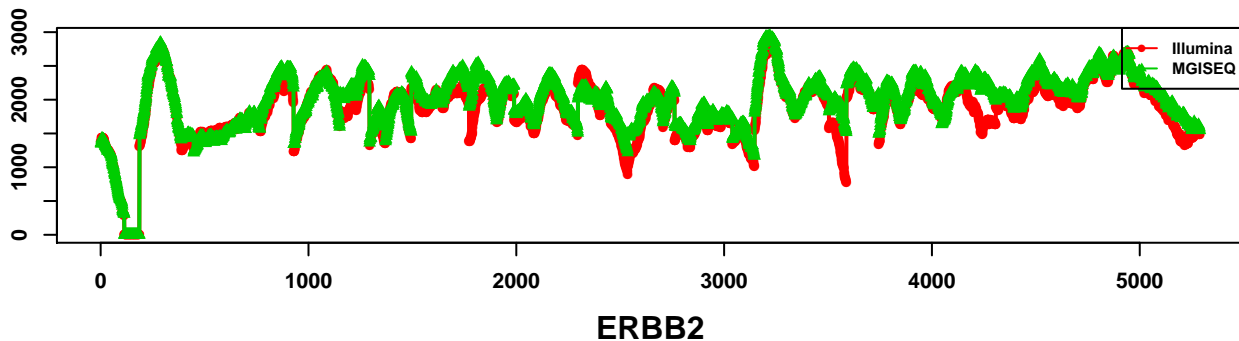

Sequencing Depth

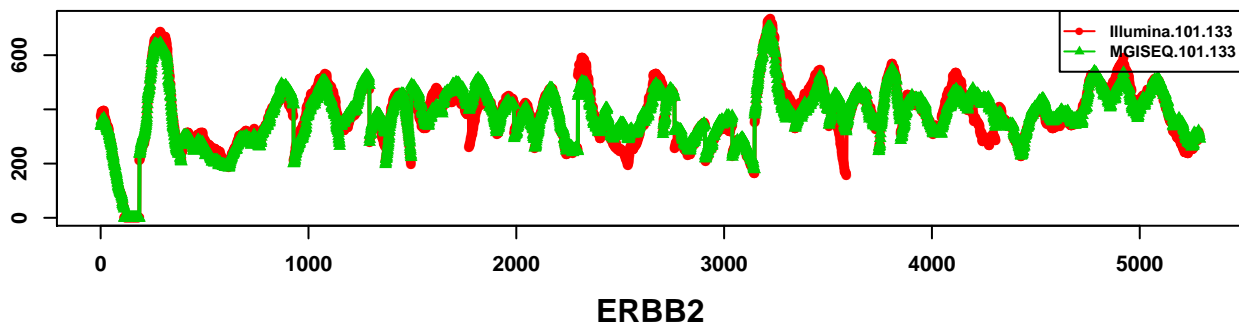

Sequencing Depth

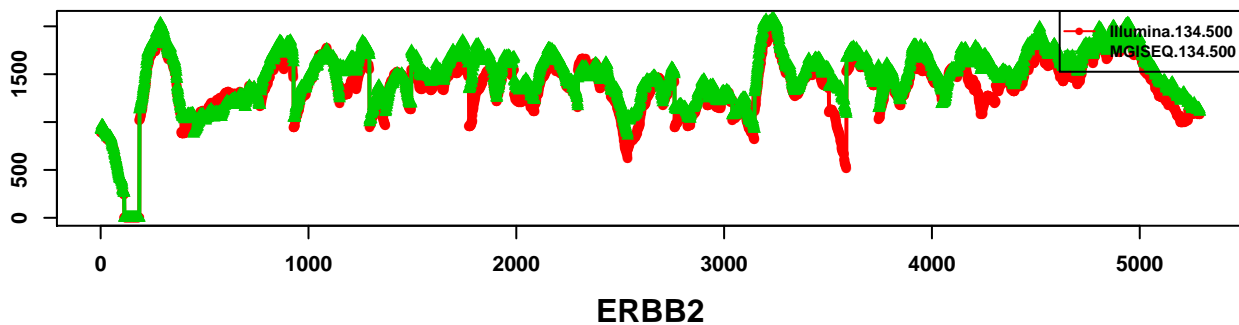

Supplement: Supplementary file 9 [file Presentation6.zip › ERBB2/19YT53595P.pdf]

Sequencing Depth

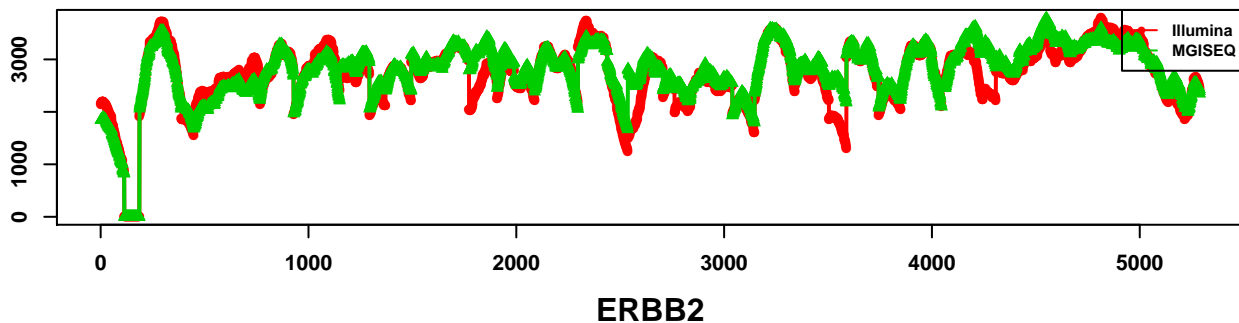

Sequencing Depth

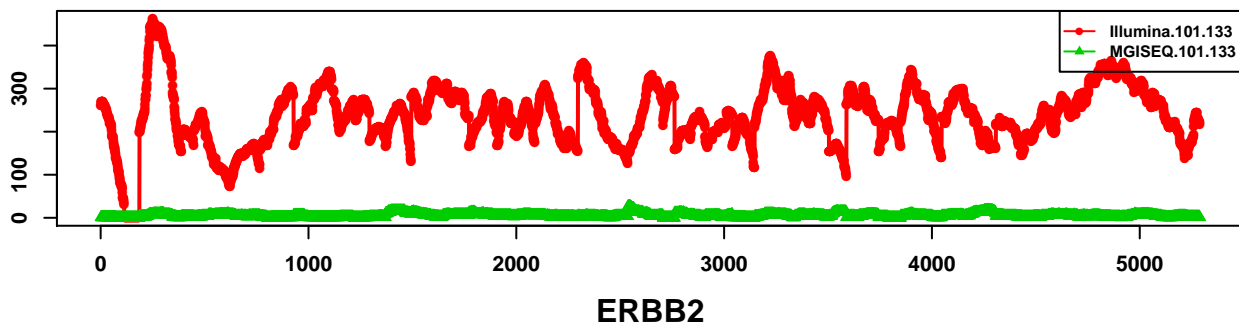

Sequencing Depth

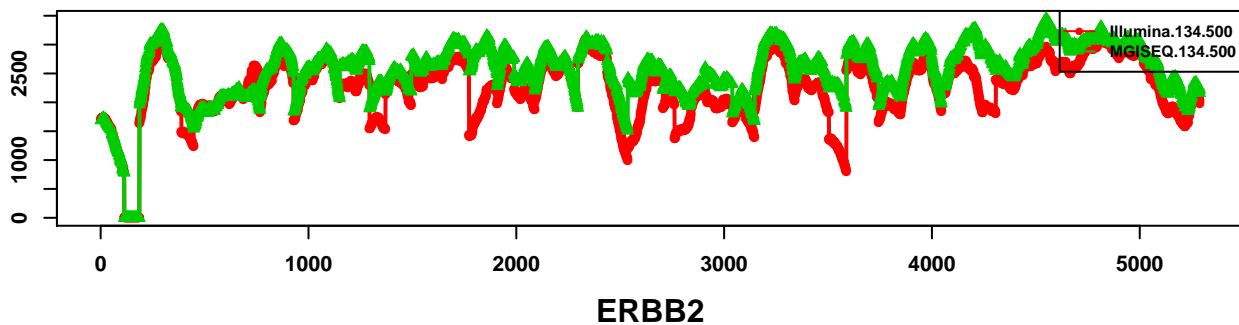

Supplement: Supplementary file 9 [file Presentation6.zip › ERBB2/19ZN13103P.pdf]

Sequencing Depth

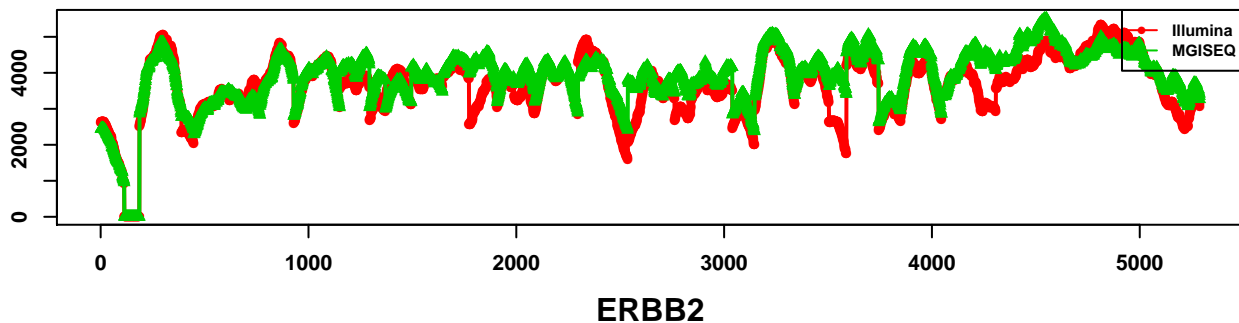

Sequencing Depth

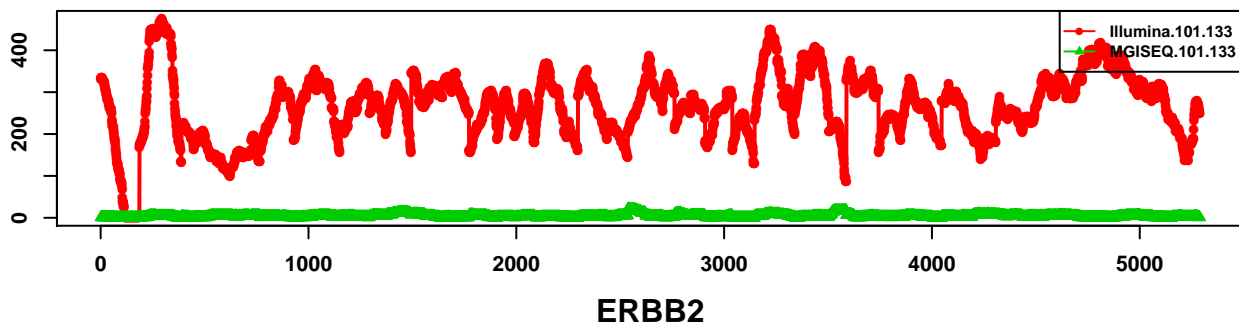

Sequencing Depth

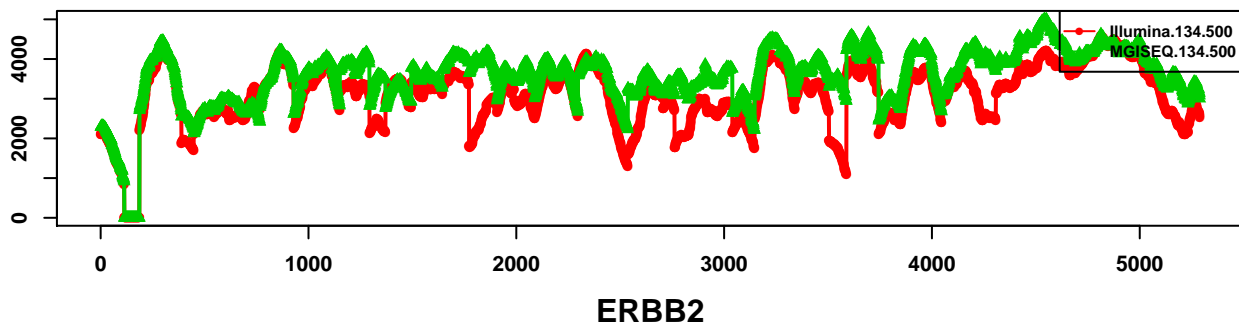

Supplement: Supplementary file 9 [file Presentation6.zip › ERBB2/19N01653P.pdf]

Sequencing Depth

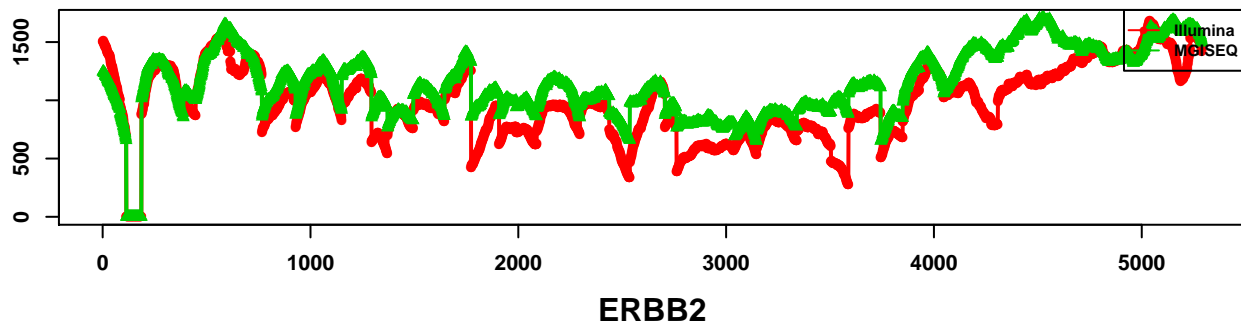

Sequencing Depth

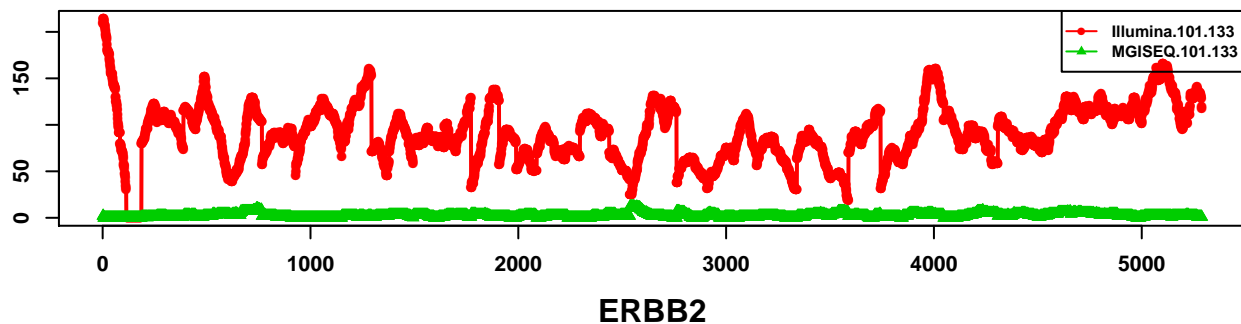

Sequencing Depth

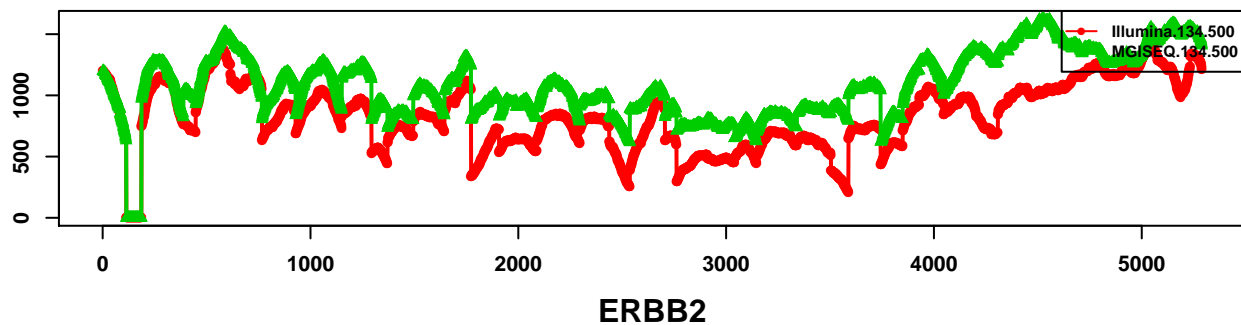

Supplement: Supplementary file 9 [file Presentation6.zip › ERBB2/19HE21980F.pdf]

Sequencing Depth

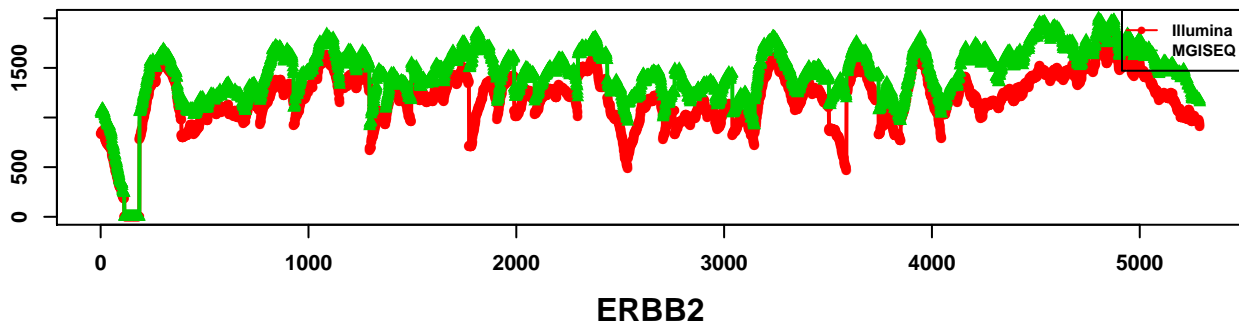

Sequencing Depth

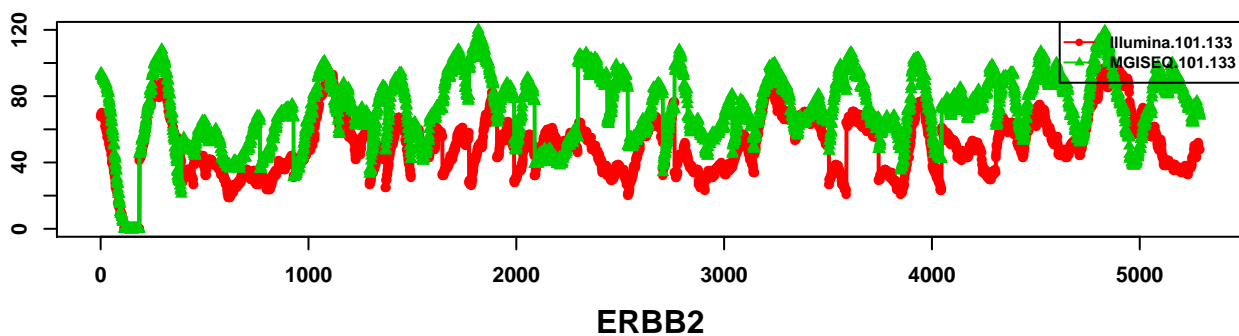

Sequencing Depth

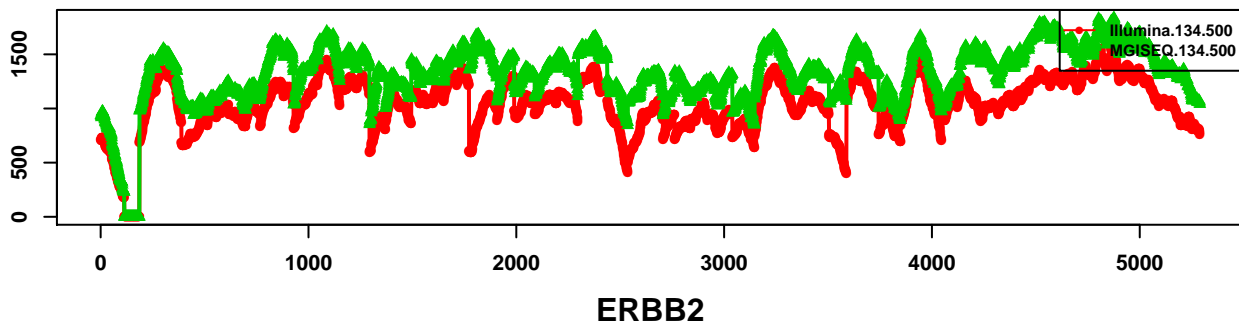

Supplement: Supplementary file 9 [file Presentation6.zip › ERBB2/FZ19-04447P.pdf]

Sequencing Depth

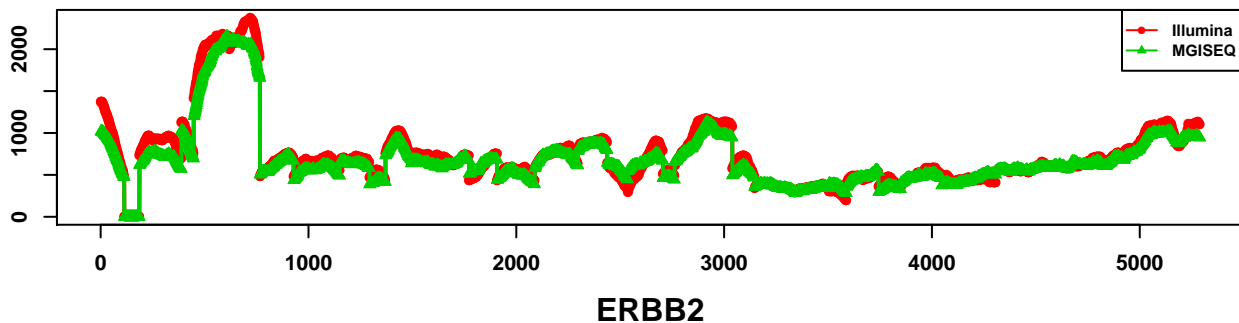

Sequencing Depth

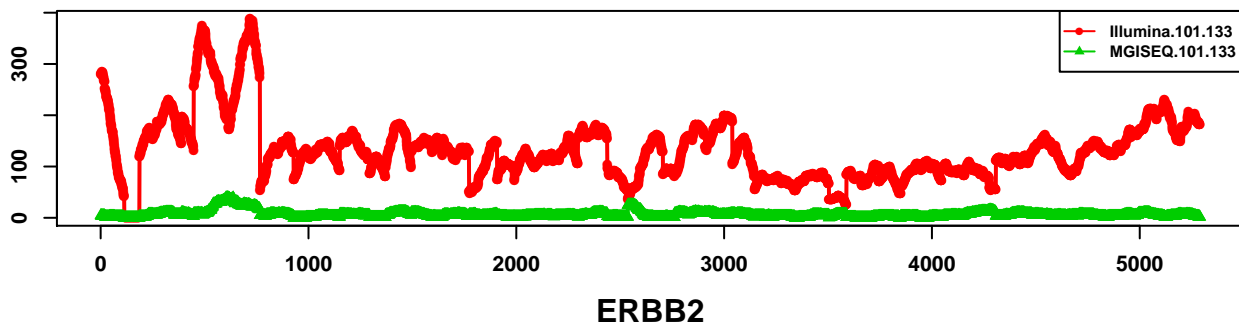

Sequencing Depth

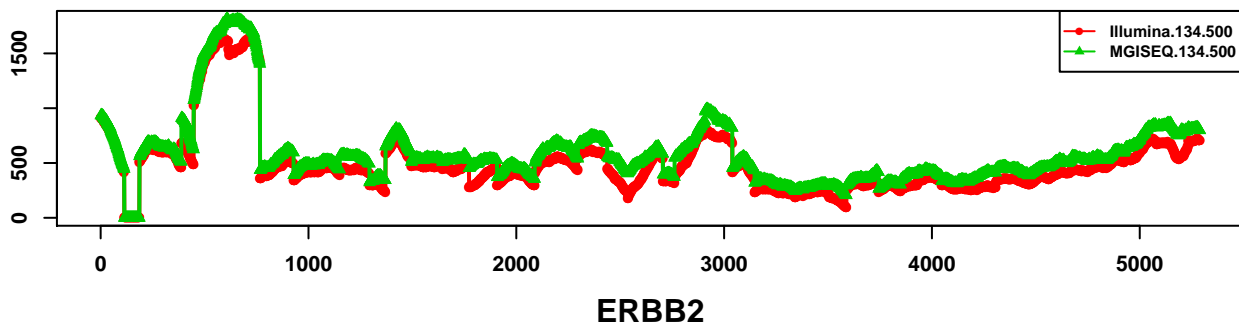

Supplement: Supplementary file 9 [file Presentation6.zip › ERBB2/19N01686F.pdf]

Sequencing Depth

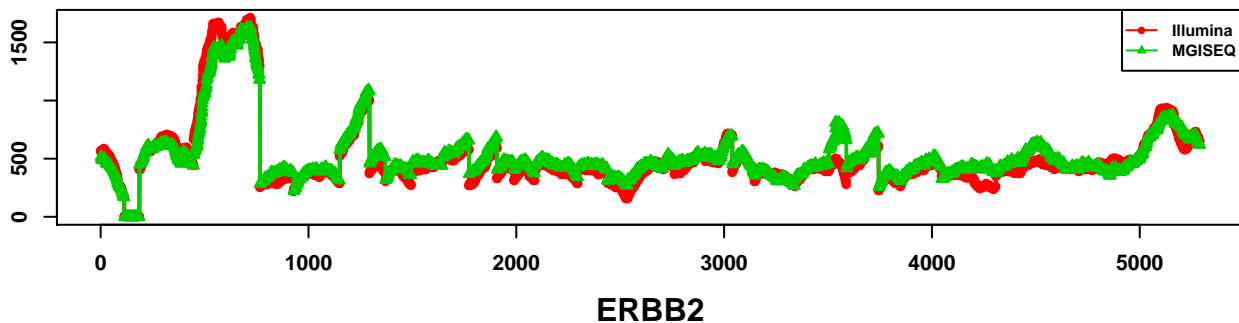

Sequencing Depth

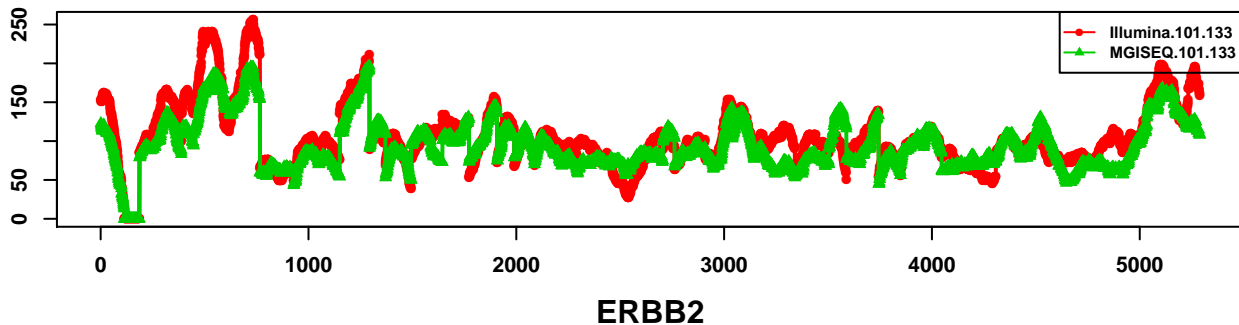

Sequencing Depth

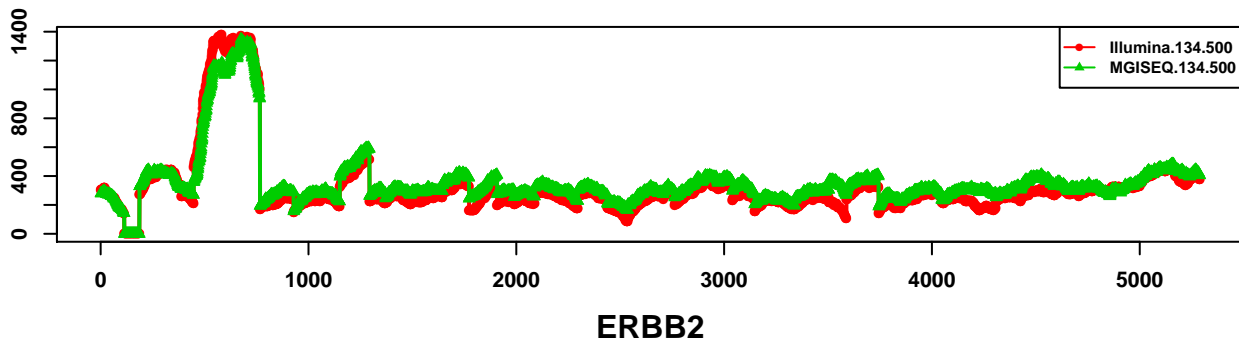

Supplement: Supplementary file 9 [file Presentation6.zip › ERBB2/19HS86145F.pdf]

Sequencing Depth

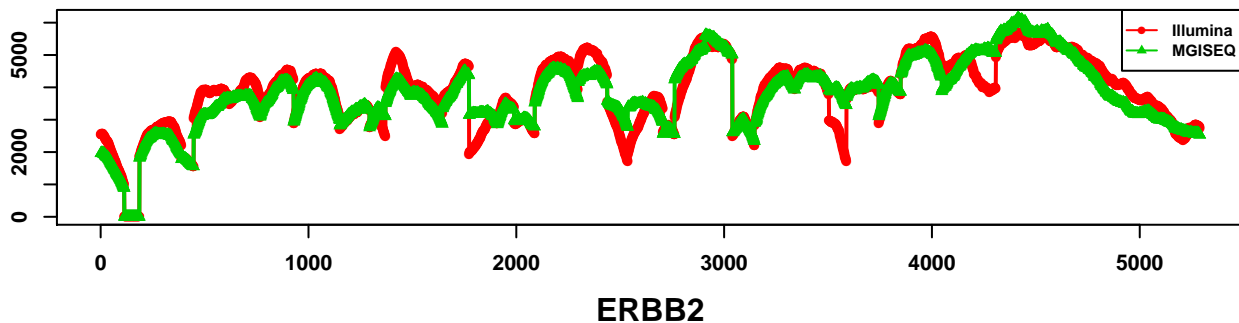

Sequencing Depth

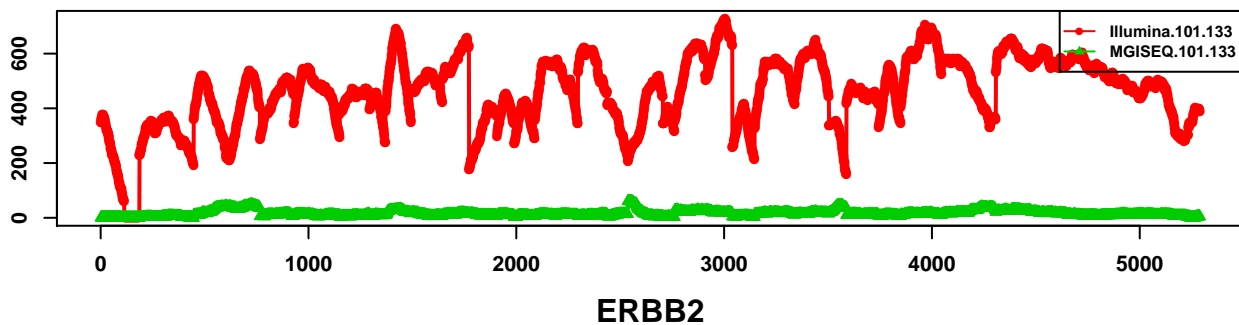

Sequencing Depth

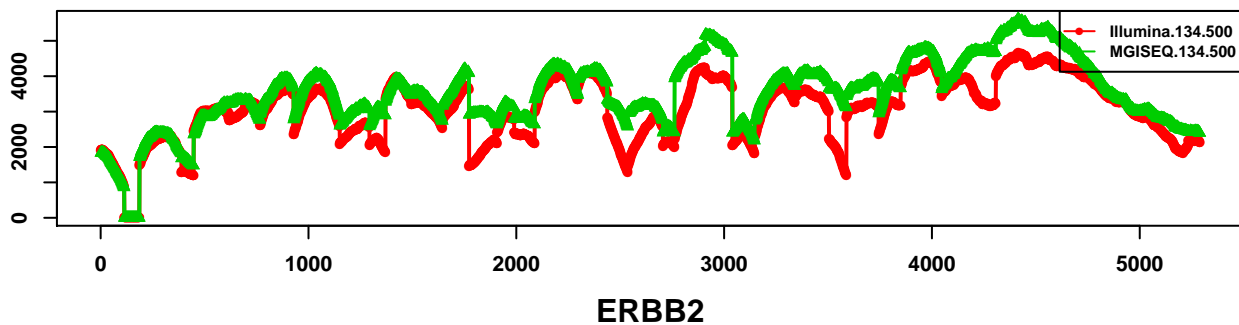

Supplement: Supplementary file 9 [file Presentation6.zip › ERBB2/19WN60001F.pdf]

Sequencing Depth

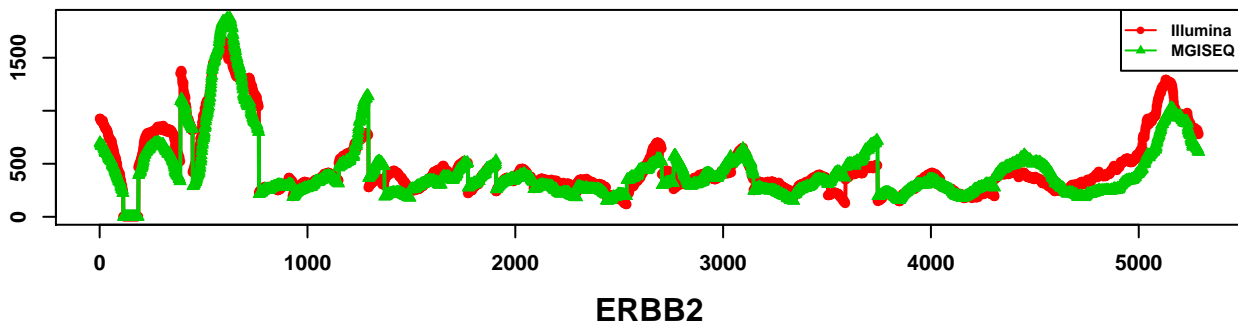

Sequencing Depth

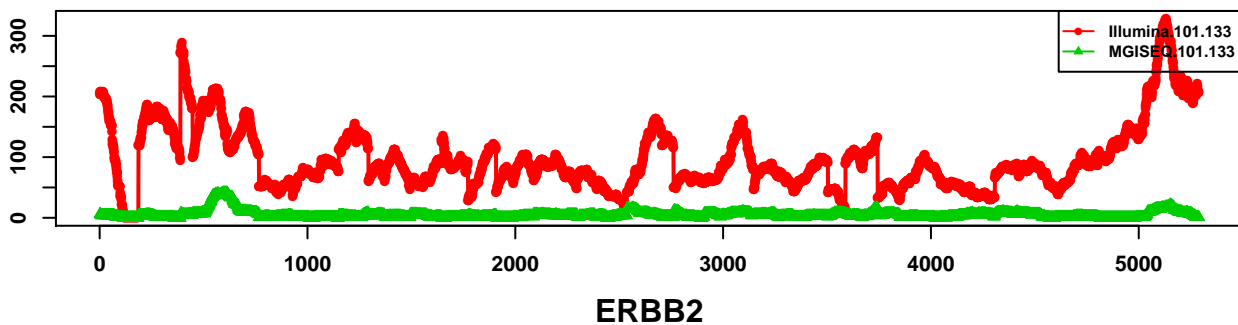

Sequencing Depth

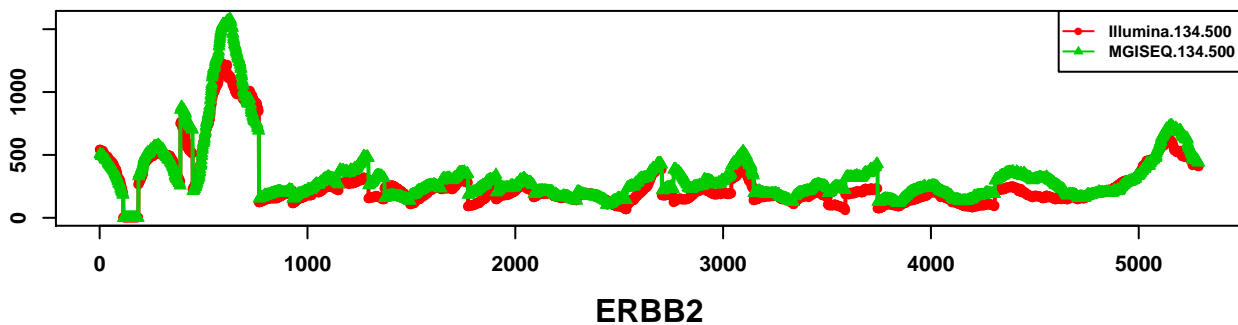

Supplement: Supplementary file 9 [file Presentation6.zip › ERBB2/19ZN13098F.pdf]

Sequencing Depth

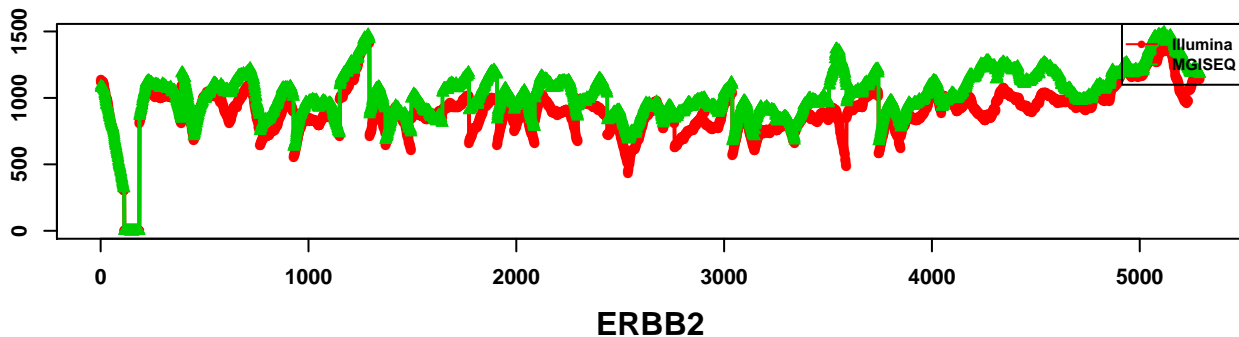

Sequencing Depth

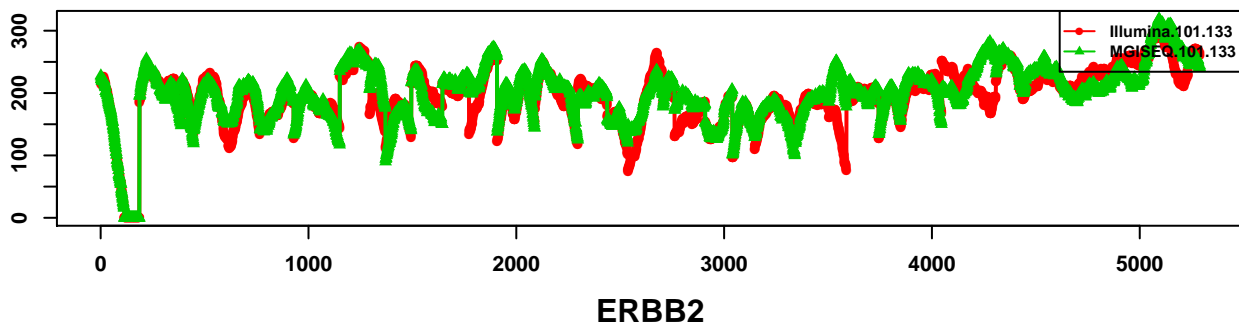

Sequencing Depth

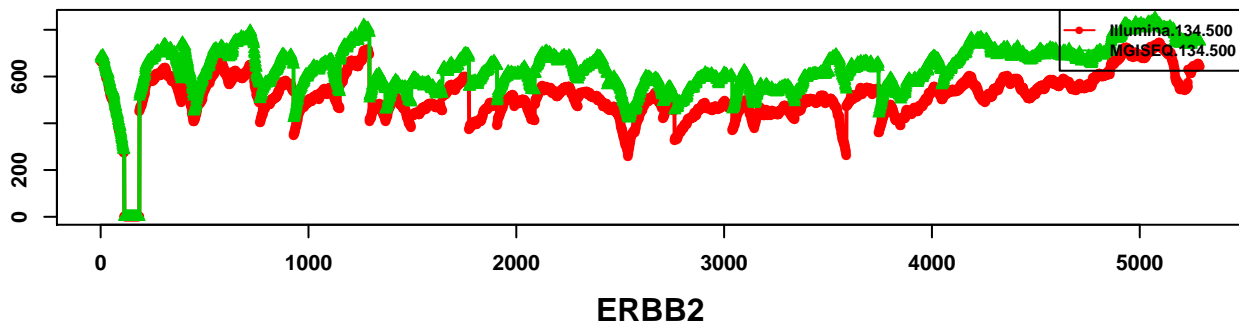

Supplement: Supplementary file 9 [file Presentation6.zip › ERBB2/19N02330F.pdf]

Sequencing Depth

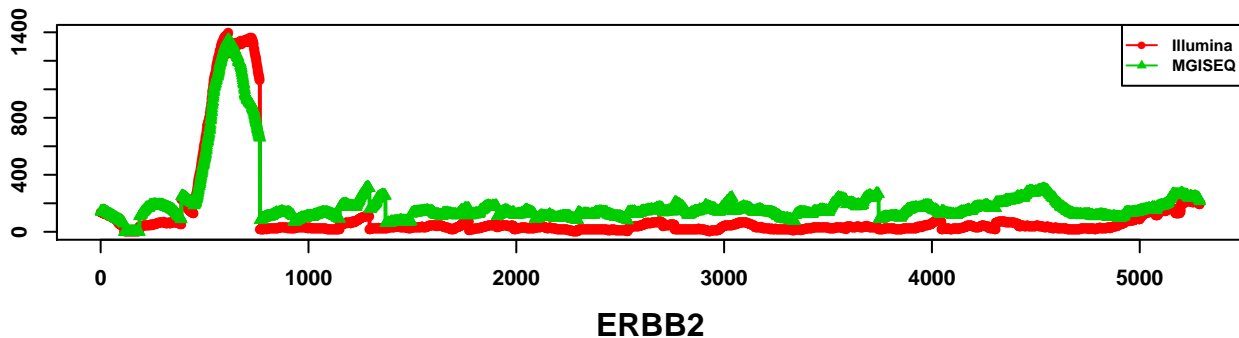

Sequencing Depth

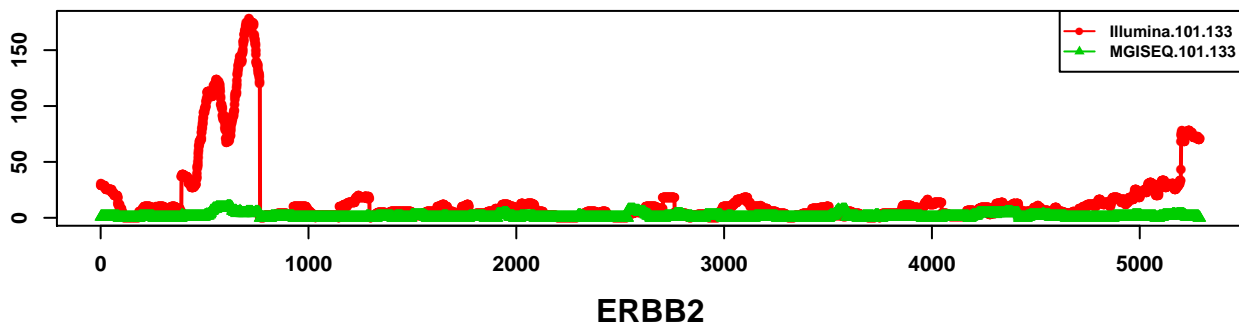

Sequencing Depth

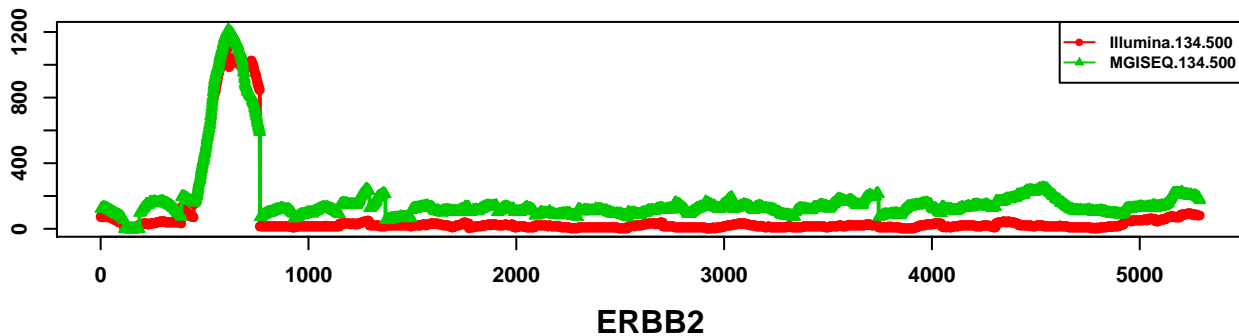

Supplement: Supplementary file 9 [file Presentation6.zip › ERBB2/19ZN12365F.pdf]

Sequencing Depth

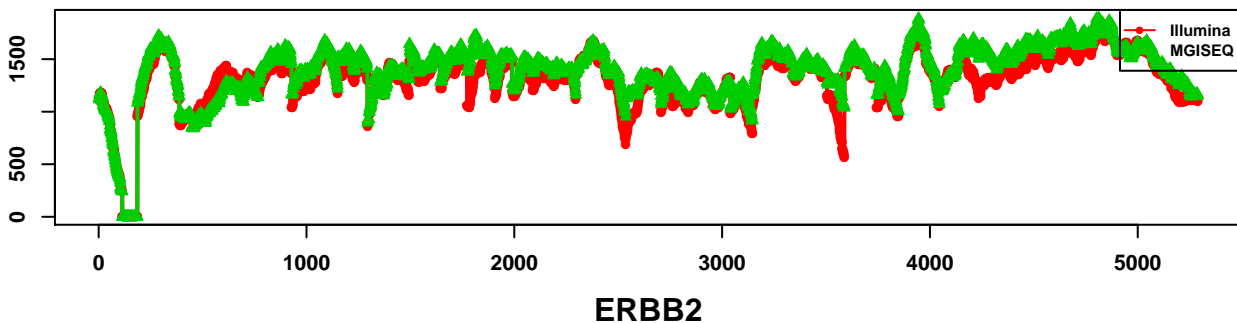

Sequencing Depth

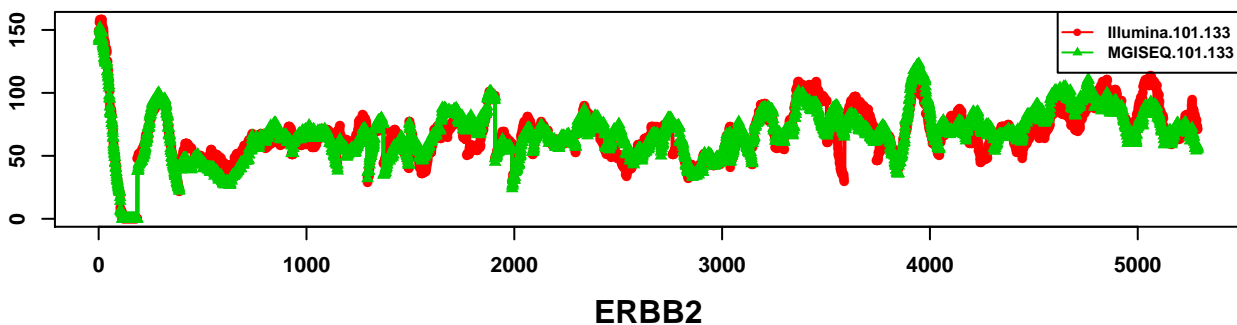

Sequencing Depth

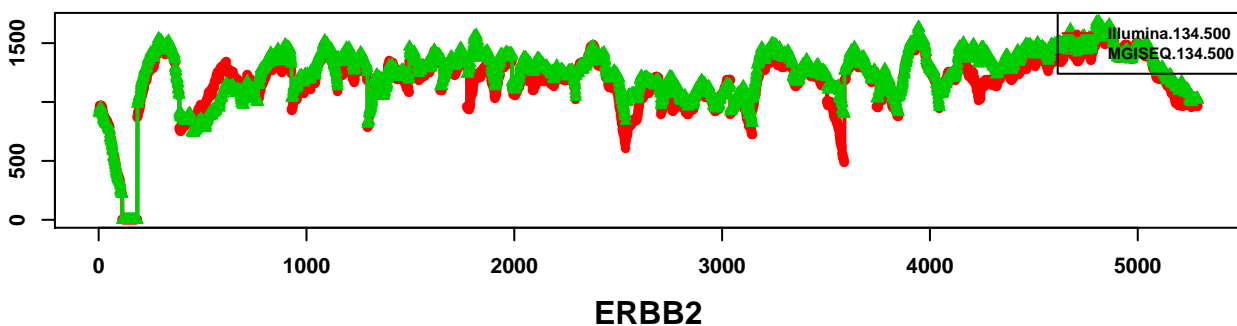

Supplement: Supplementary file 9 [file Presentation6.zip › ERBB2/19ZN13489P.pdf]

Sequencing Depth

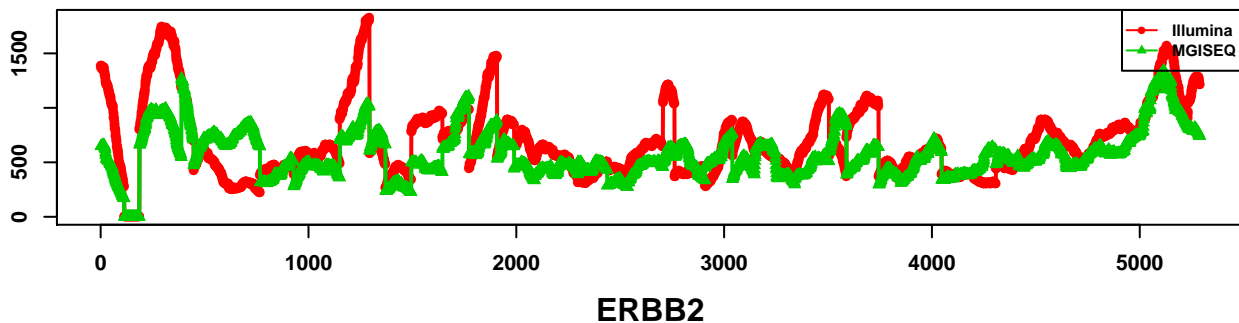

Sequencing Depth

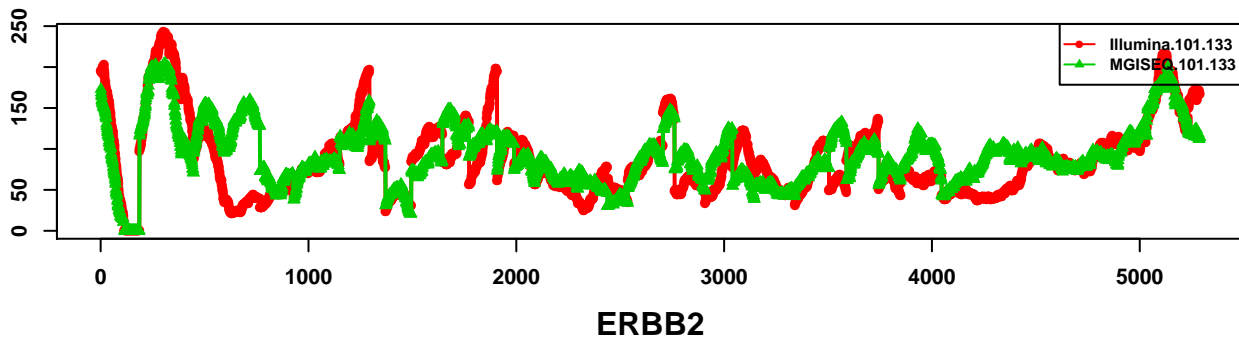

Sequencing Depth

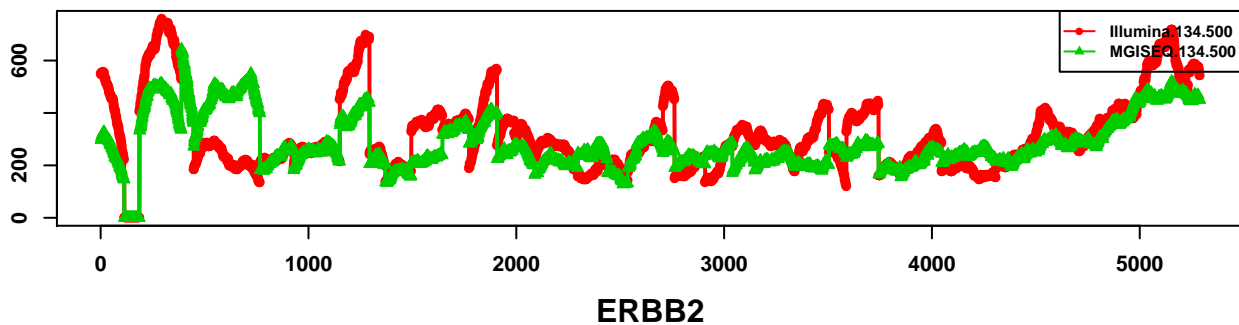

Supplement: Supplementary file 9 [file Presentation6.zip › ERBB2/19ZN12575F.pdf]

Sequencing Depth

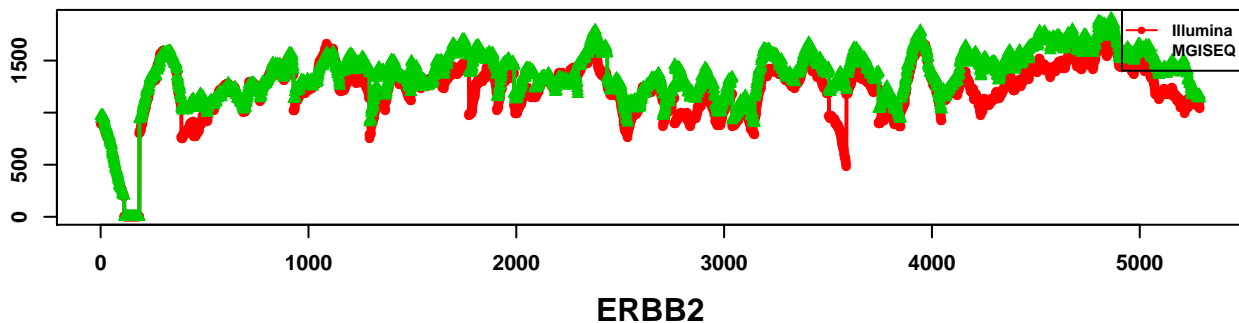

Sequencing Depth

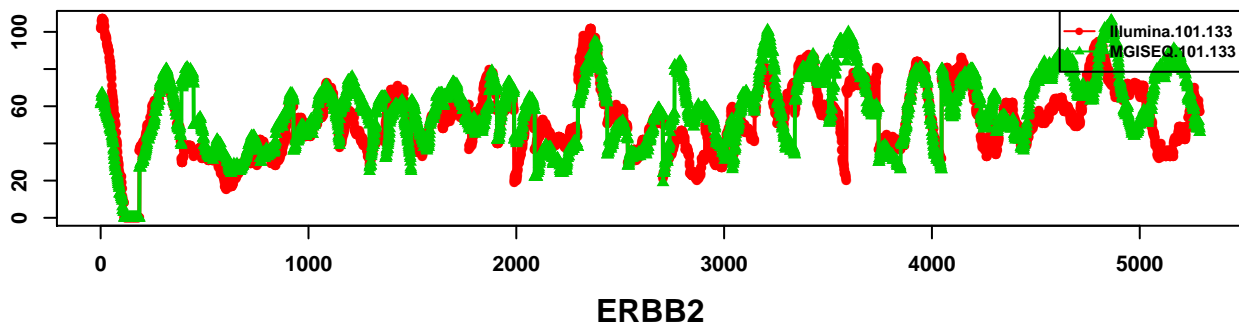

Sequencing Depth

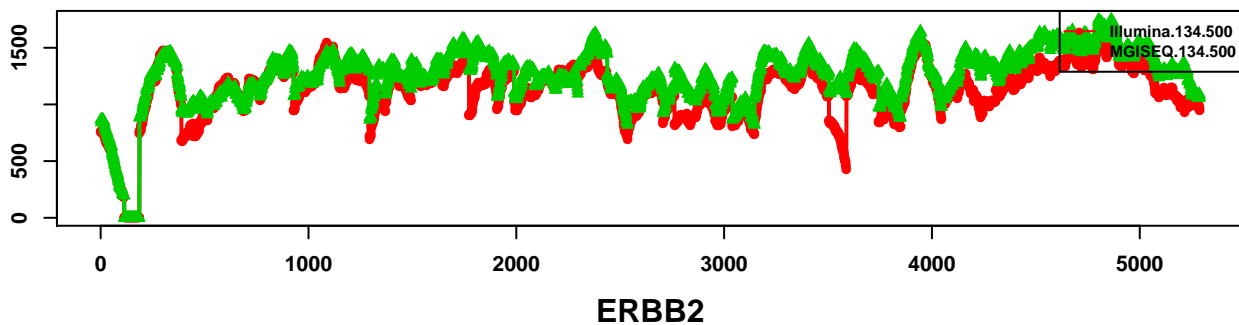

Supplement: Supplementary file 9 [file Presentation6.zip › ERBB2/19JS48265P.pdf]

Sequencing Depth

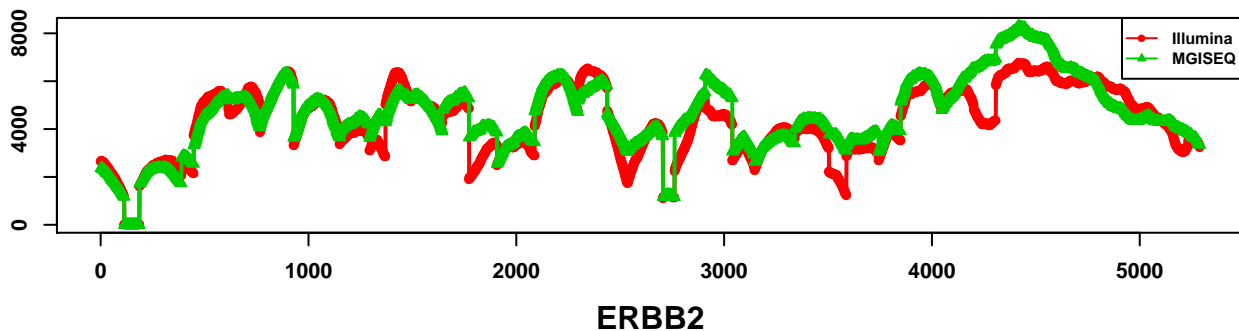

Sequencing Depth

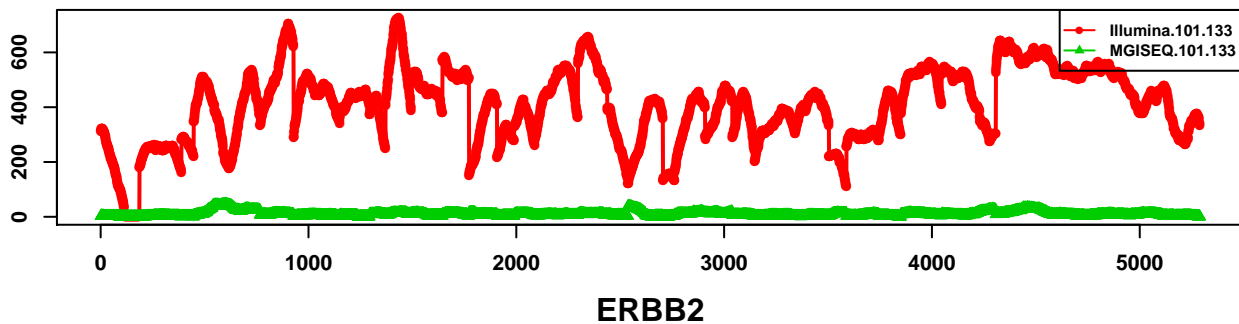

Sequencing Depth

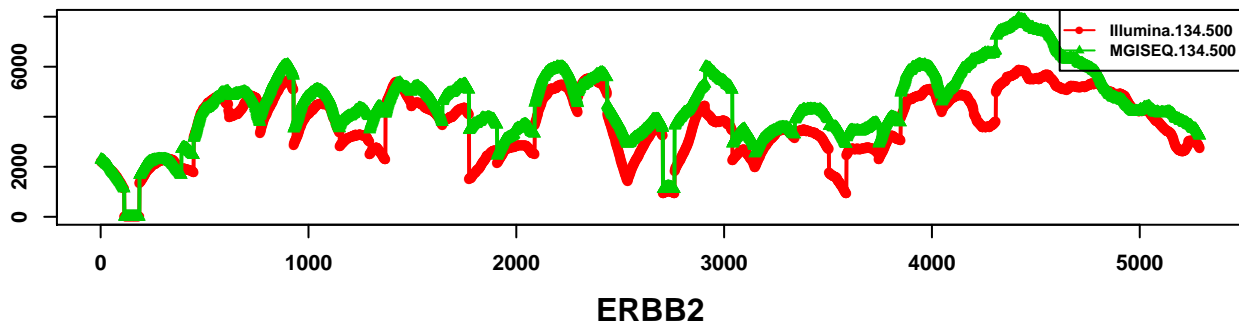

Supplement: Supplementary file 9 [file Presentation6.zip › ERBB2/19N01658F.pdf]

Sequencing Depth

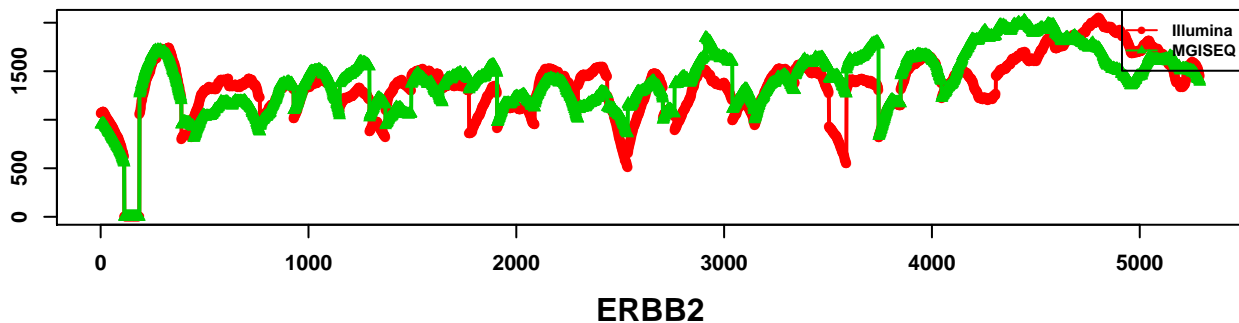

Sequencing Depth

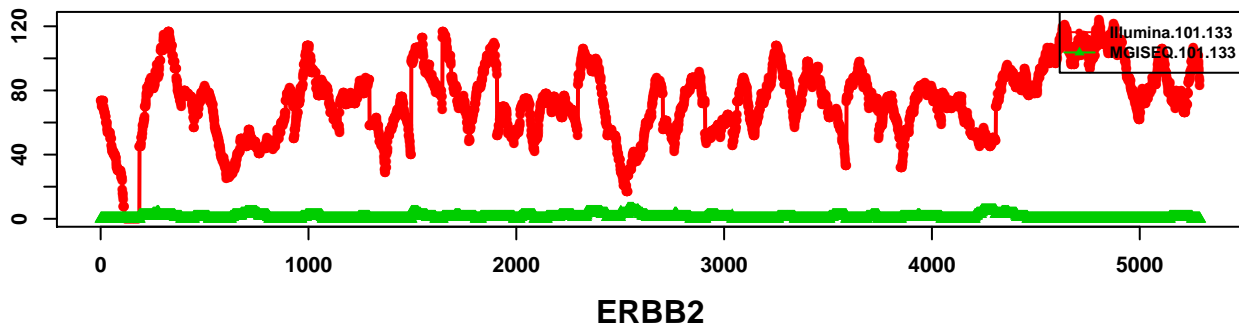

Sequencing Depth

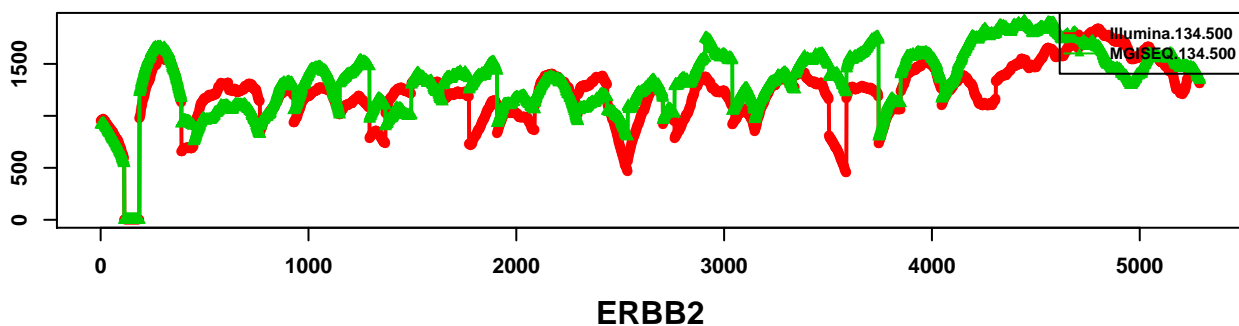

Supplement: Supplementary file 9 [file Presentation6.zip › ERBB2/ZK0603-G.pdf]

Sequencing Depth

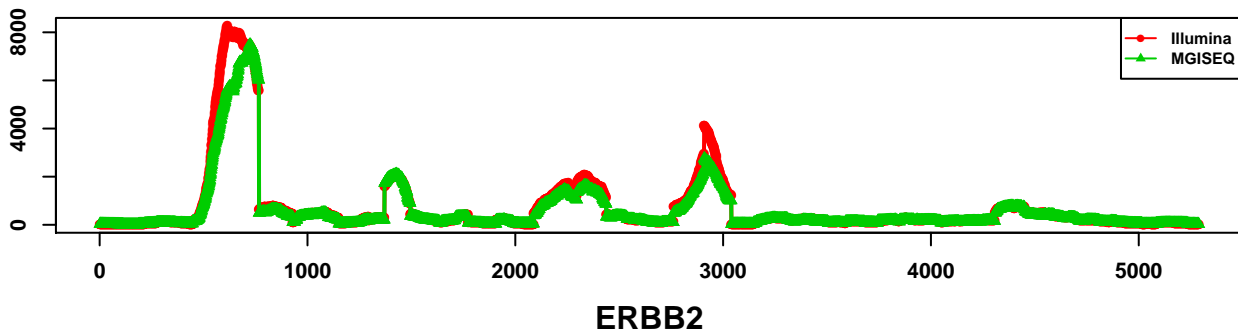

Sequencing Depth

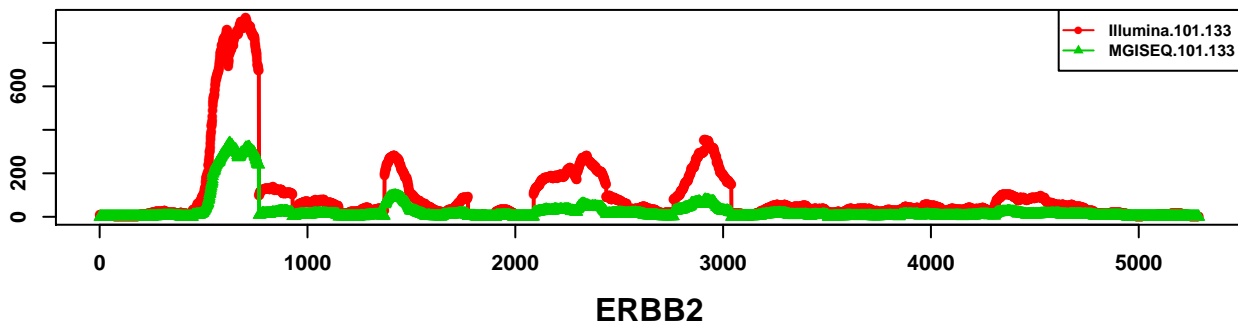

Sequencing Depth

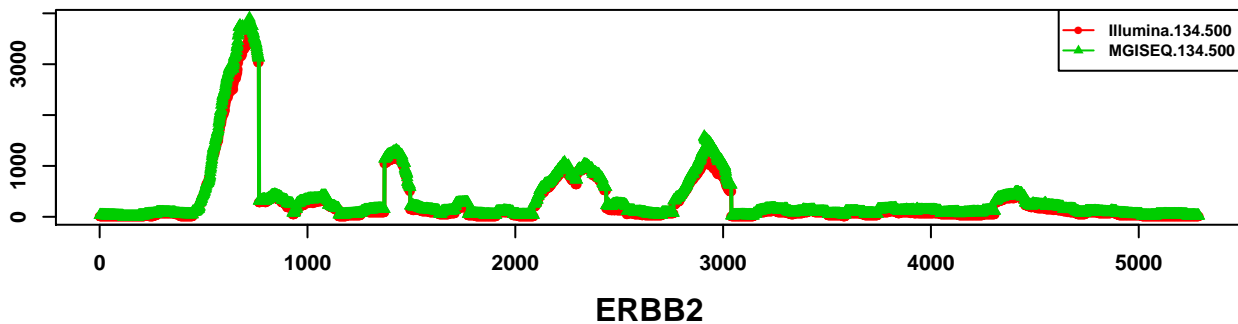

Supplement: Supplementary file 9 [file Presentation6.zip › ERBB2/19ZN13099T.pdf]

Sequencing Depth

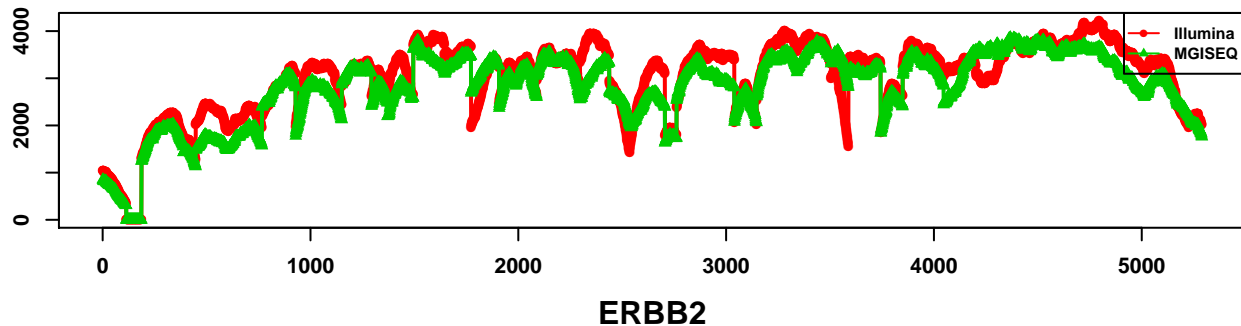

Sequencing Depth

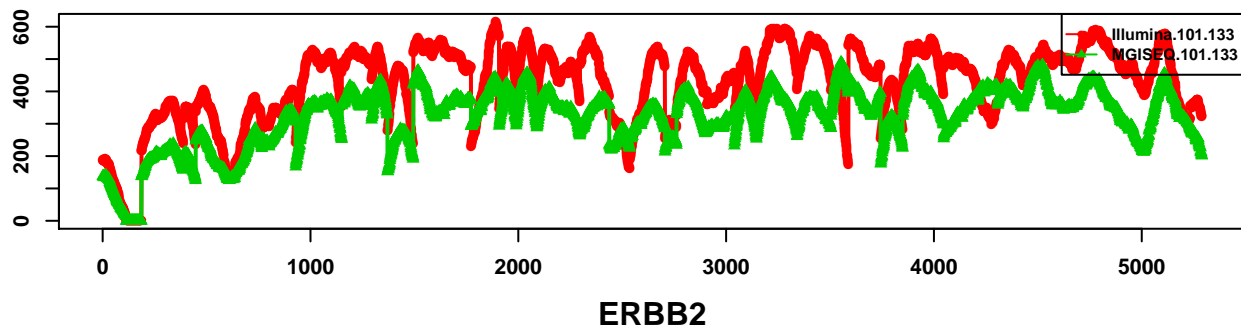

Sequencing Depth

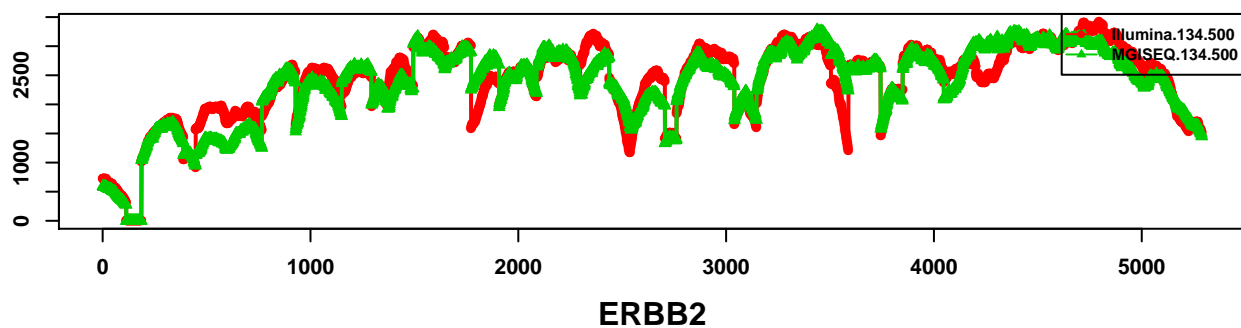

Supplement: Supplementary file 9 [file Presentation6.zip › ERBB2/19N02331T.pdf]

Sequencing Depth

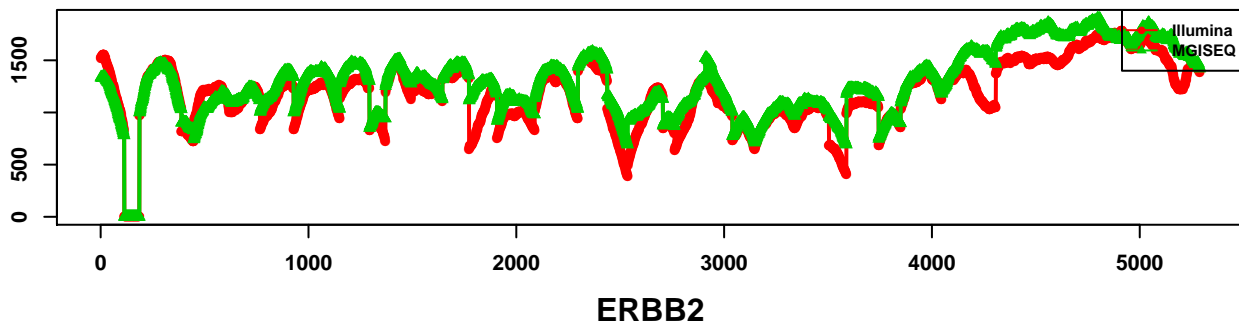

Sequencing Depth

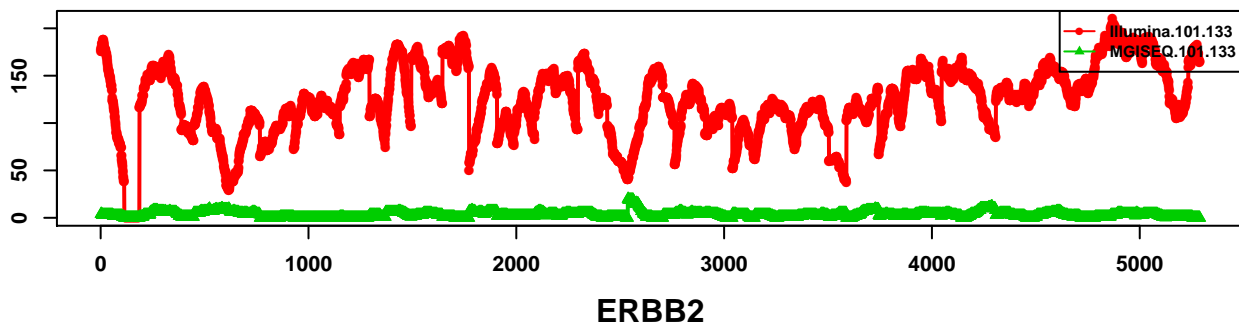

Sequencing Depth

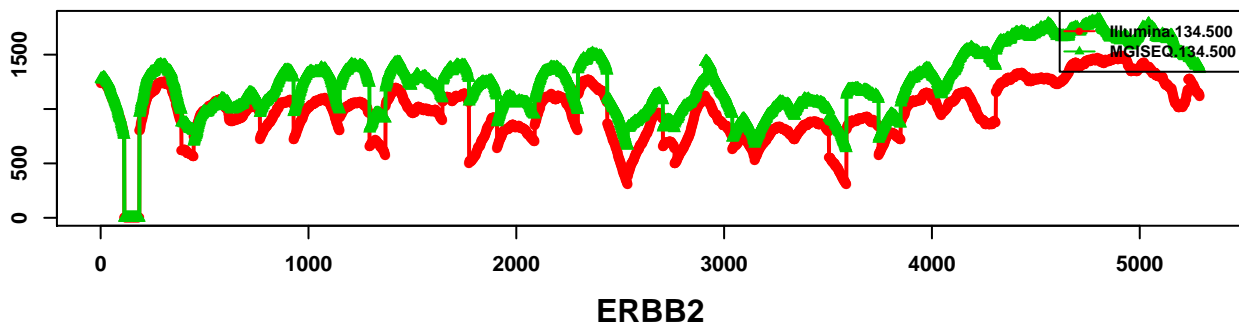

Supplement: Supplementary file 9 [file Presentation6.zip › ERBB2/19HE22108F.pdf]

Sequencing Depth

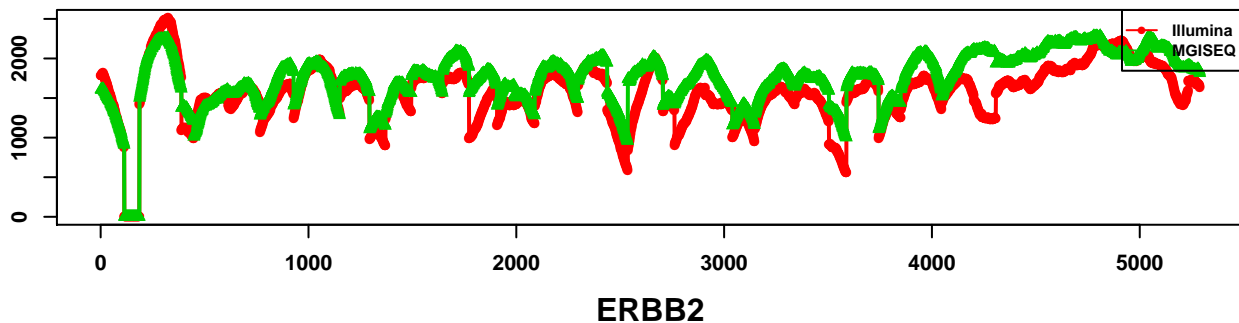

Sequencing Depth

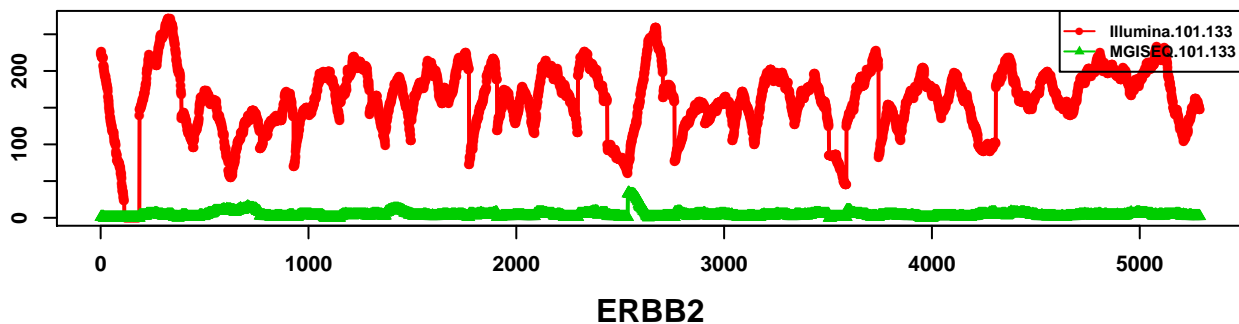

Sequencing Depth

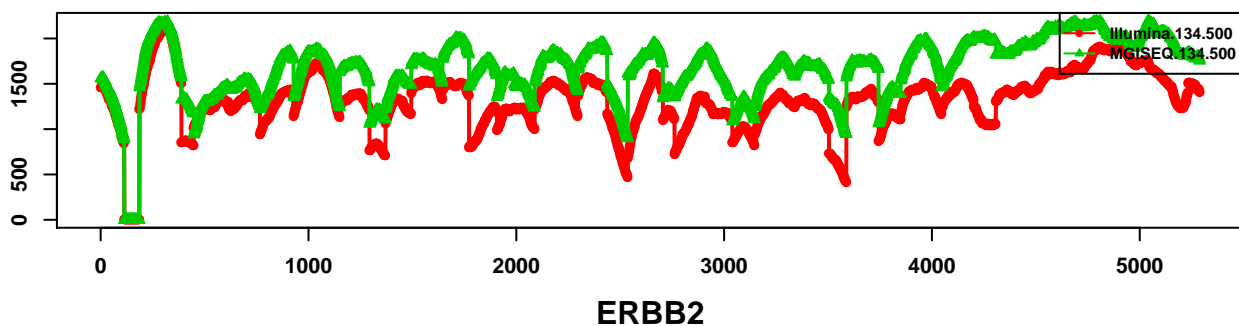

Supplement: Supplementary file 9 [file Presentation6.zip › ERBB2/19ZN11292F.pdf]

Sequencing Depth

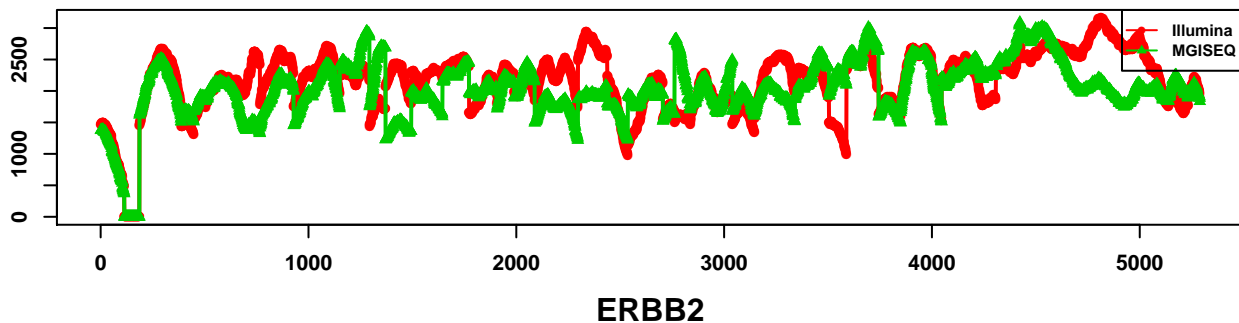

Sequencing Depth

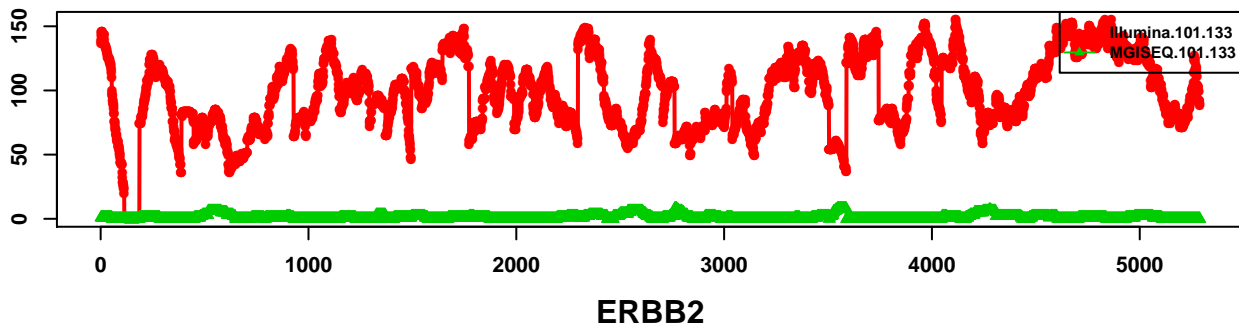

Sequencing Depth

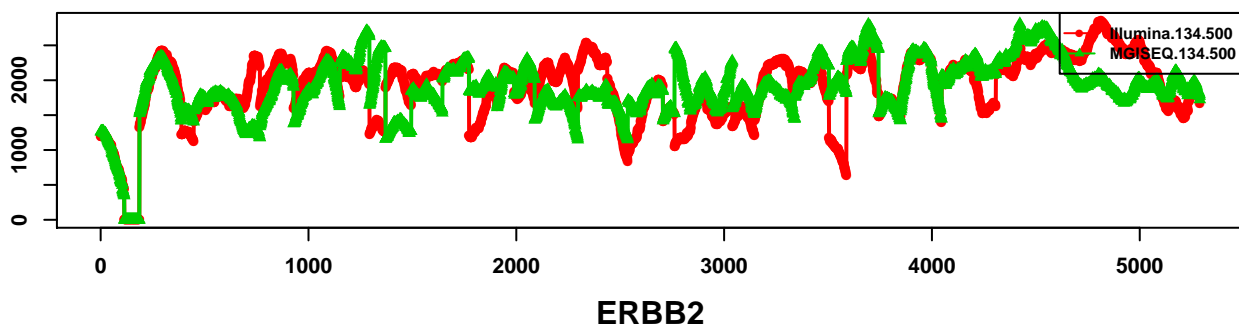

Supplement: Supplementary file 9 [file Presentation6.zip › ERBB2/19HS86165P.pdf]

Sequencing Depth

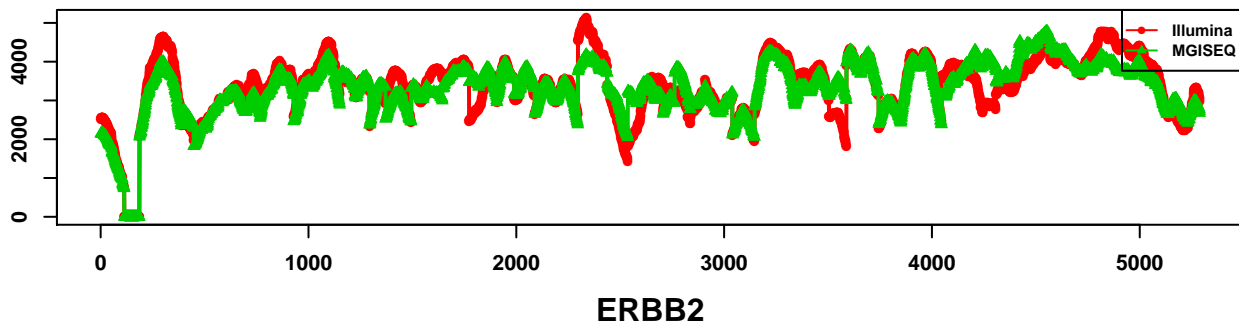

Sequencing Depth

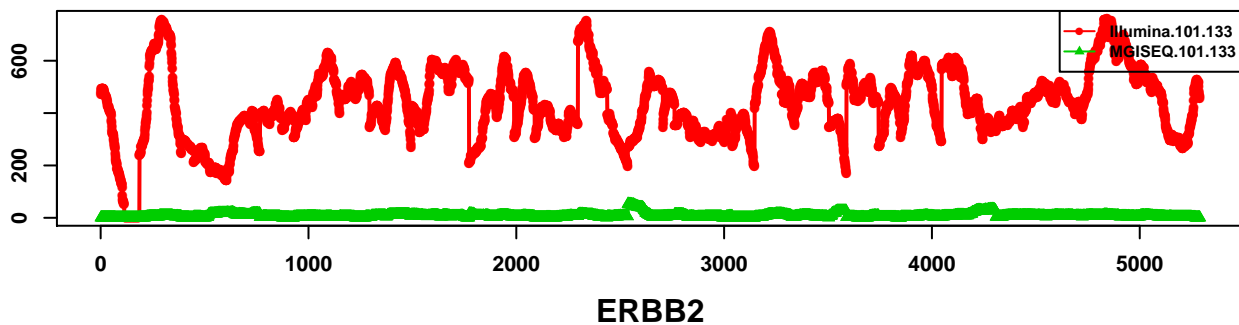

Sequencing Depth

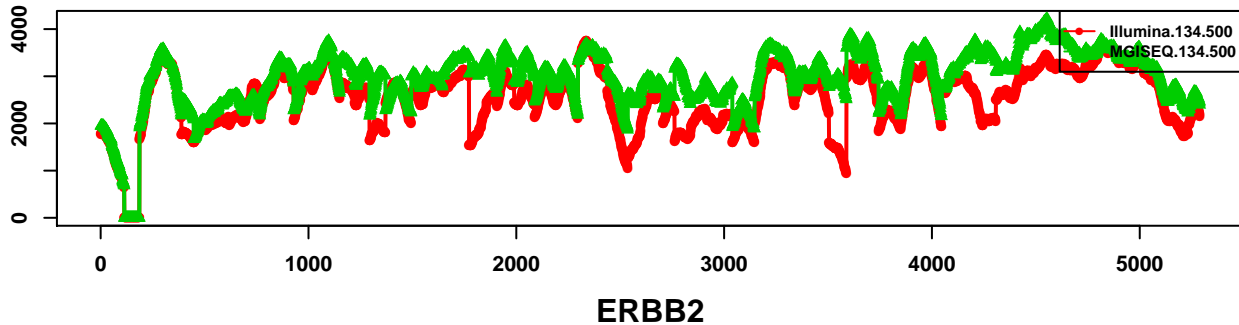

Supplement: Supplementary file 9 [file Presentation6.zip › ERBB2/FZ19-03014P.pdf]

Sequencing Depth

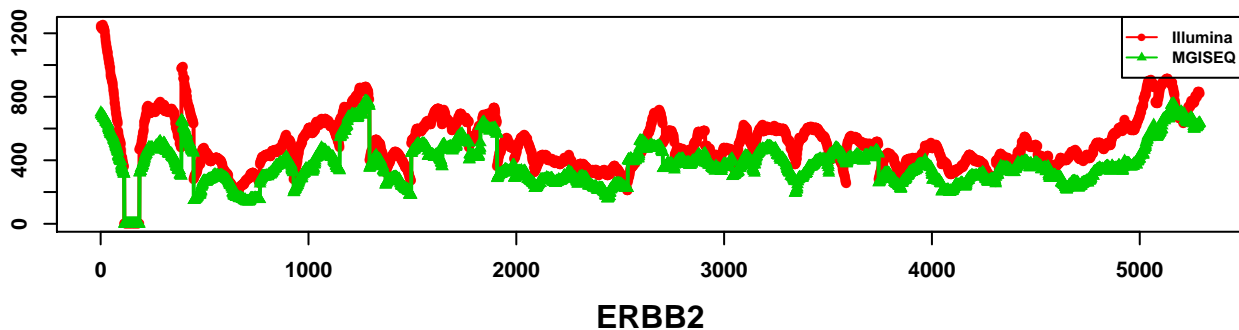

Sequencing Depth

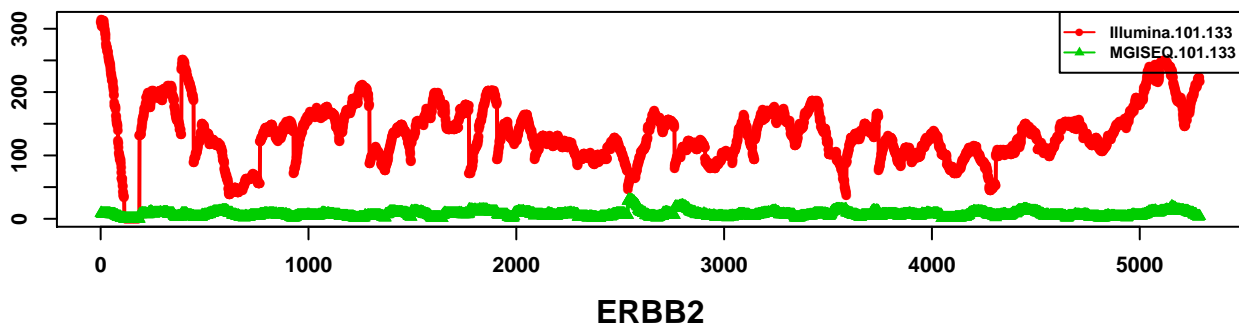

Sequencing Depth

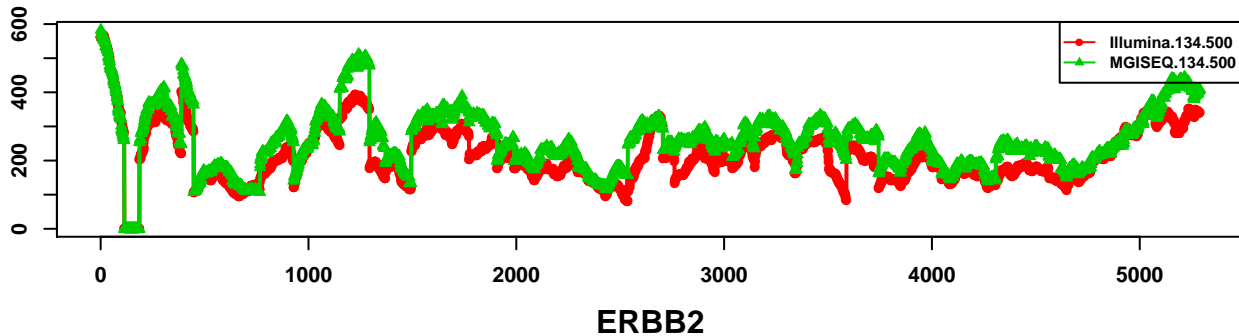

Supplement: Supplementary file 9 [file Presentation6.zip › ERBB2/19HE21737-IIF.pdf]

Sequencing Depth

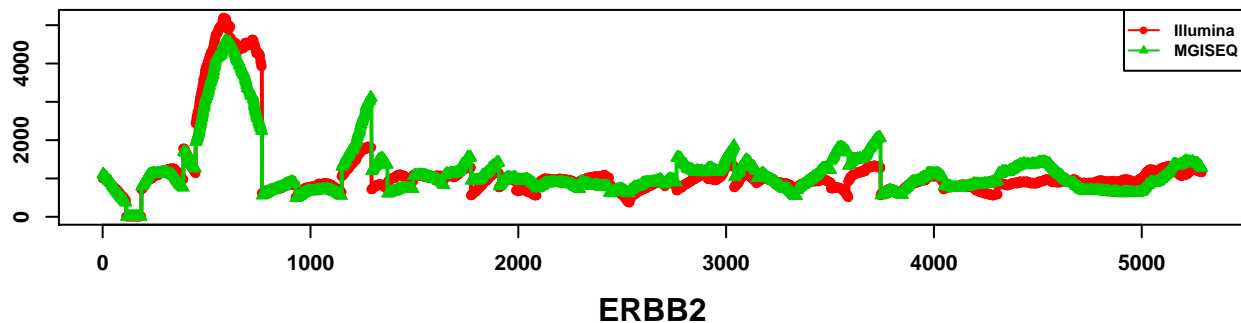

Sequencing Depth

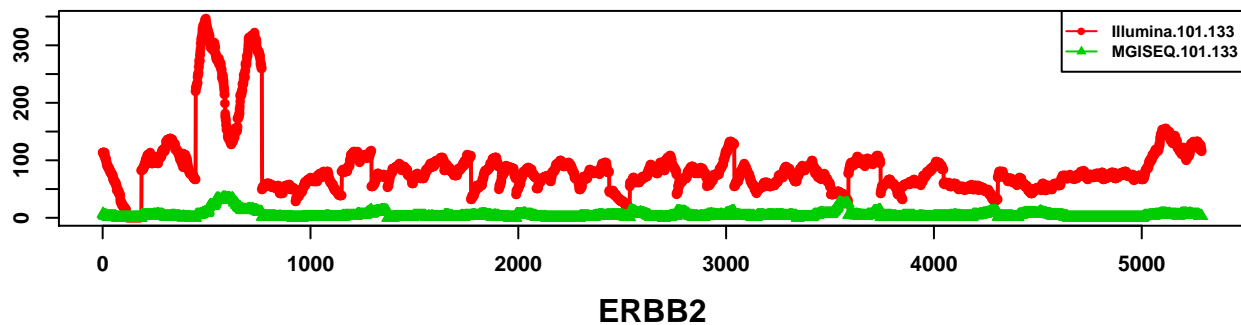

Sequencing Depth

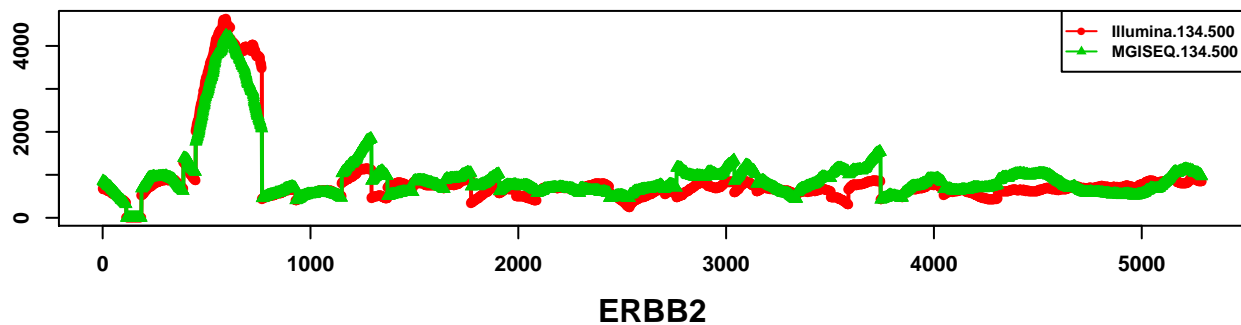

Supplement: Supplementary file 9 [file Presentation6.zip › ERBB2/19ZN13094F.pdf]

Sequencing Depth

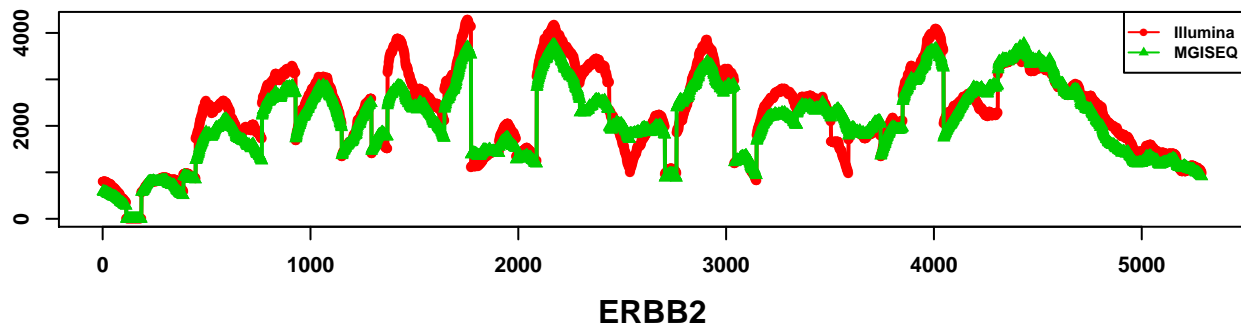

Sequencing Depth

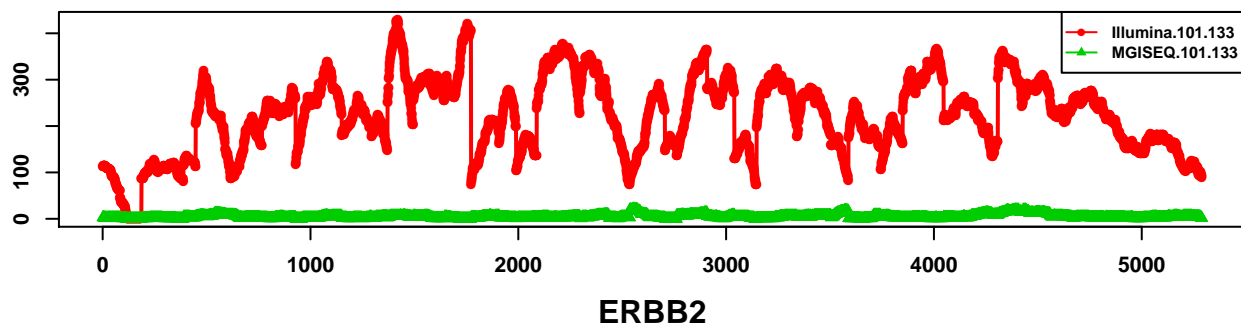

Sequencing Depth

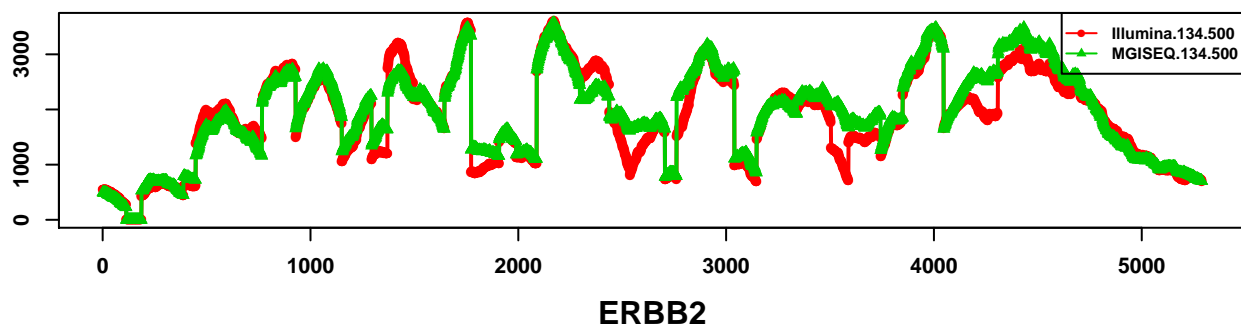

Supplement: Supplementary file 9 [file Presentation6.zip › ERBB2/19CY96044F.pdf]

Sequencing Depth

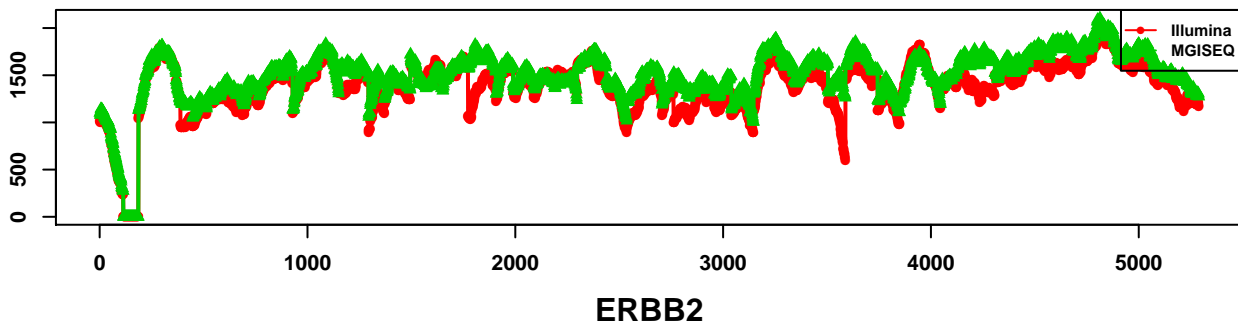

Sequencing Depth

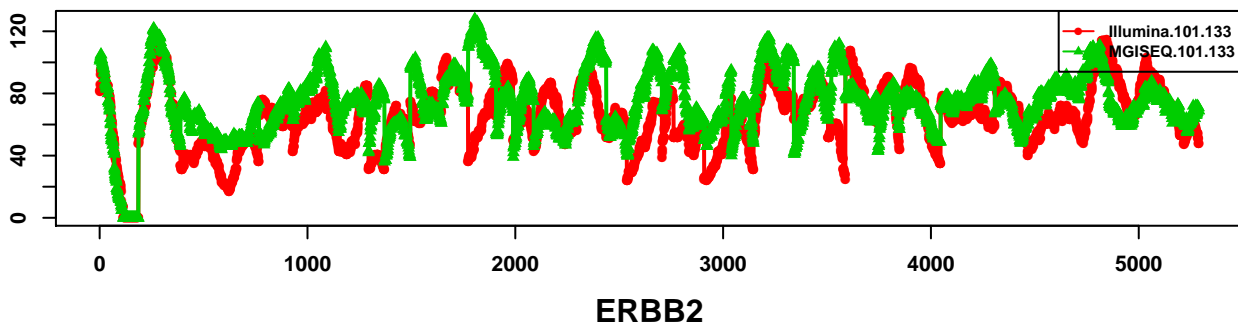

Sequencing Depth

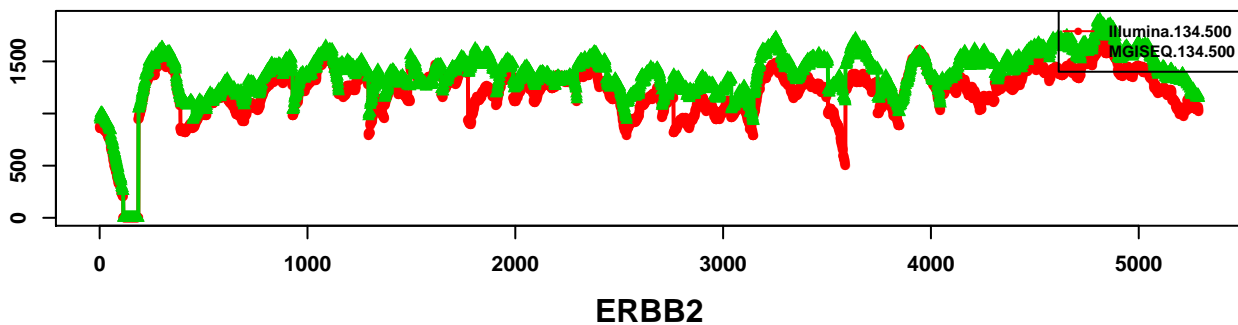

Supplement: Supplementary file 9 [file Presentation6.zip › ERBB2/19YT53879P.pdf]

Sequencing Depth

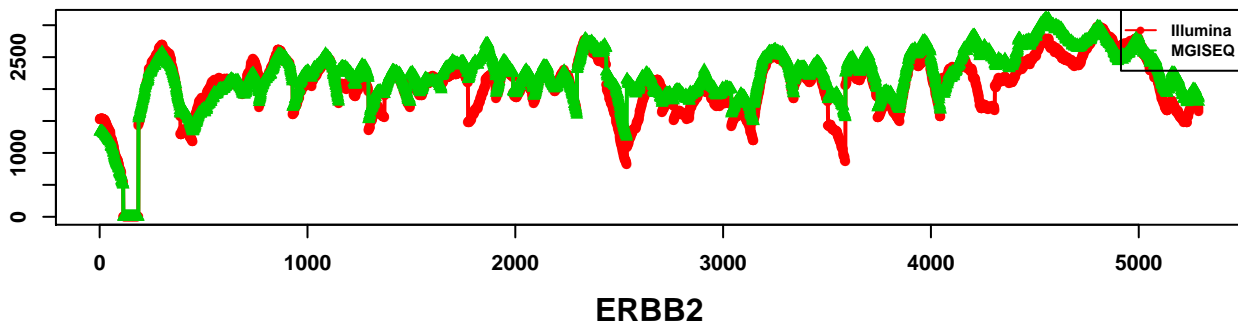

Sequencing Depth

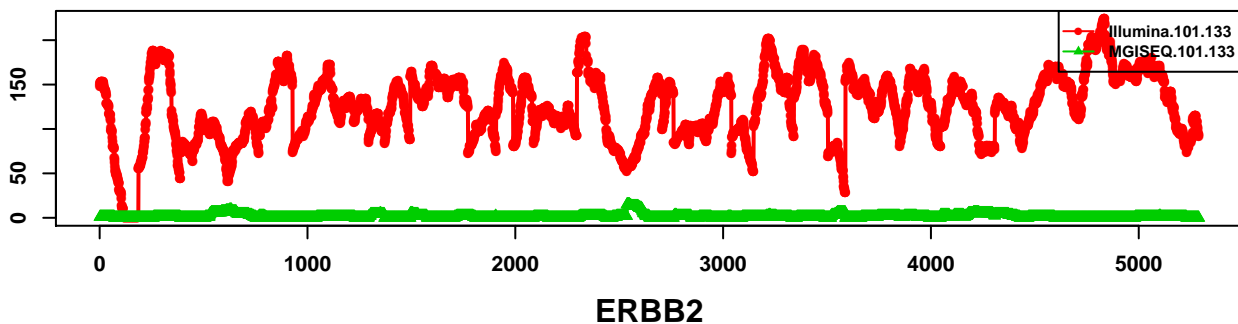

Sequencing Depth

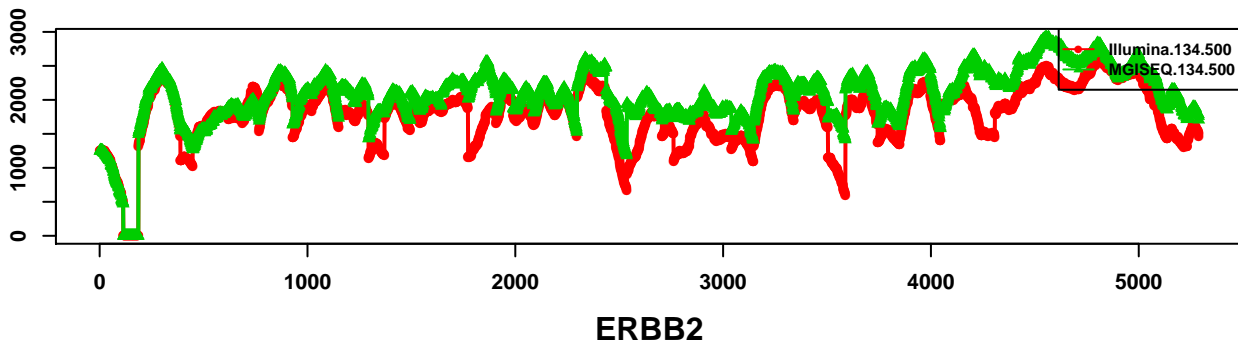

Supplement: Supplementary file 9 [file Presentation6.zip › ERBB2/19ZN13104P.pdf]

Sequencing Depth

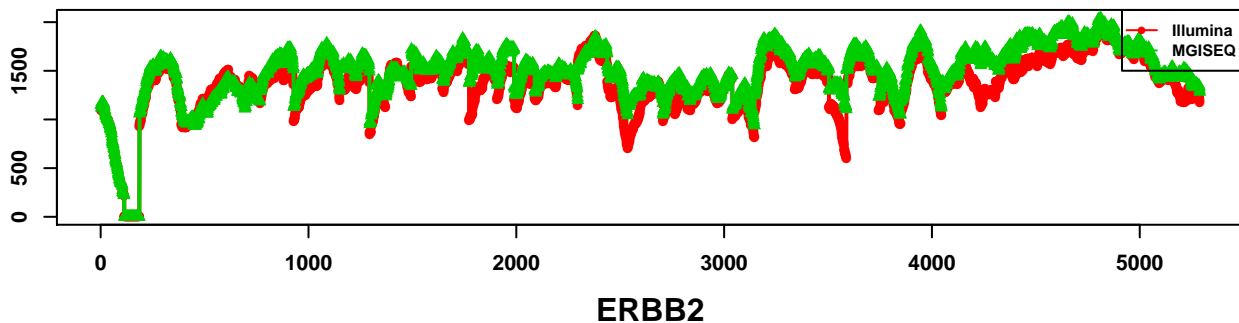

Sequencing Depth

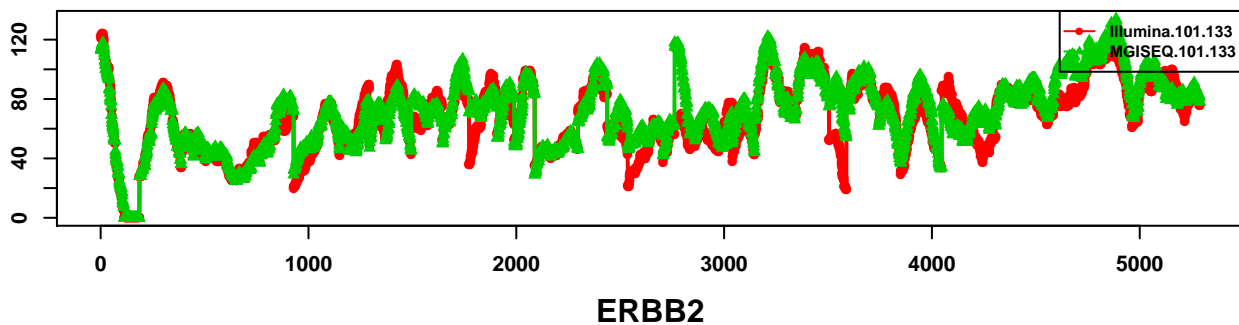

Sequencing Depth

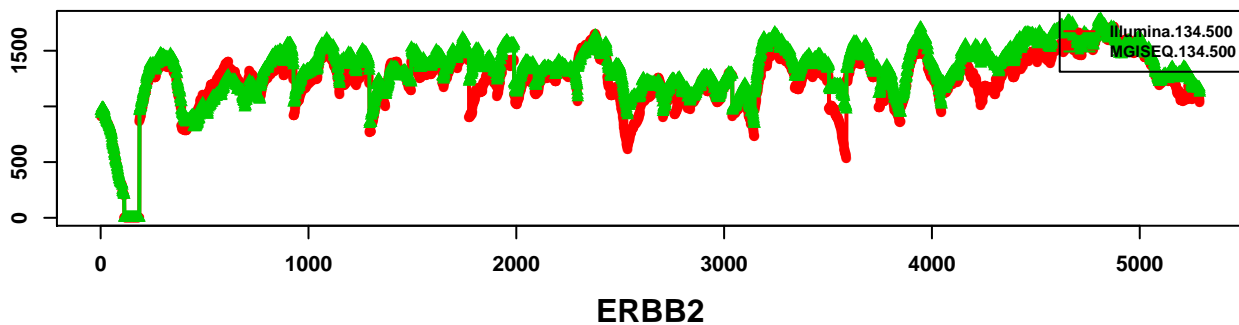

Supplement: Supplementary file 9 [file Presentation6.zip › ERBB2/19ZQ13135P.pdf]

Sequencing Depth

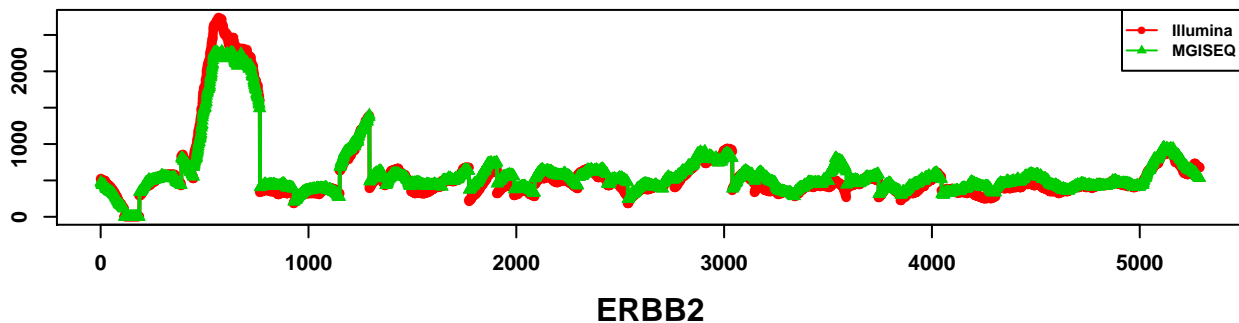

Sequencing Depth

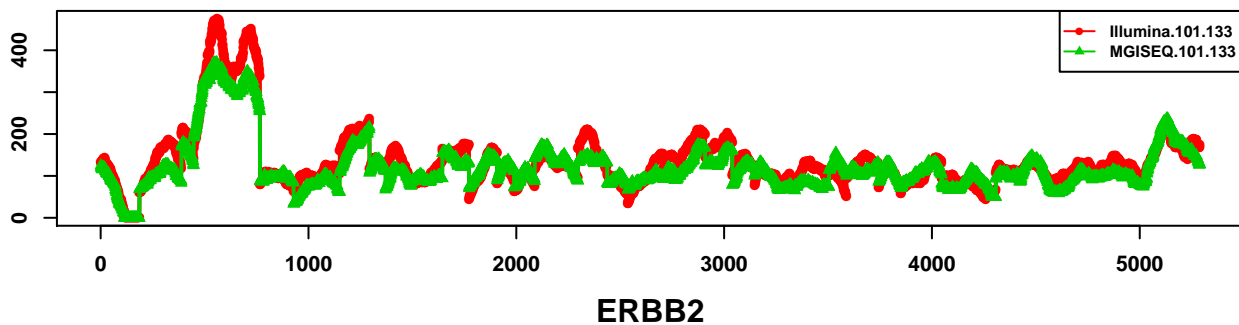

Sequencing Depth

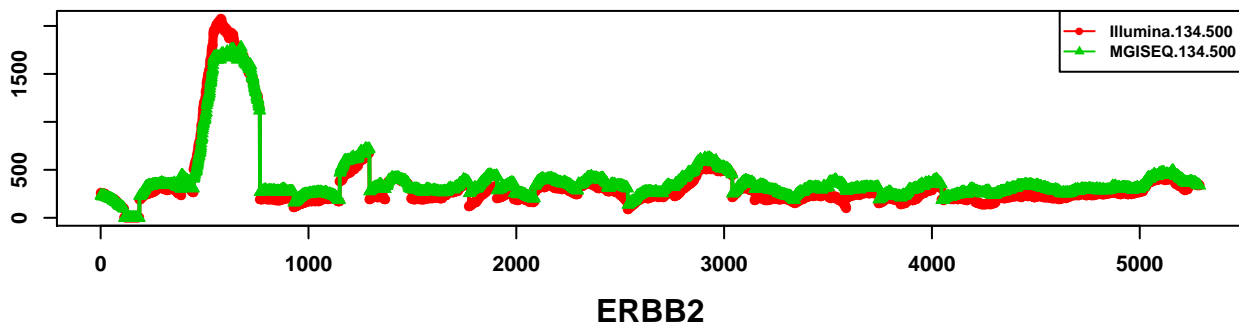

Supplement: Supplementary file 9 [file Presentation6.zip › ERBB2/19CF15689F.pdf]

Sequencing Depth

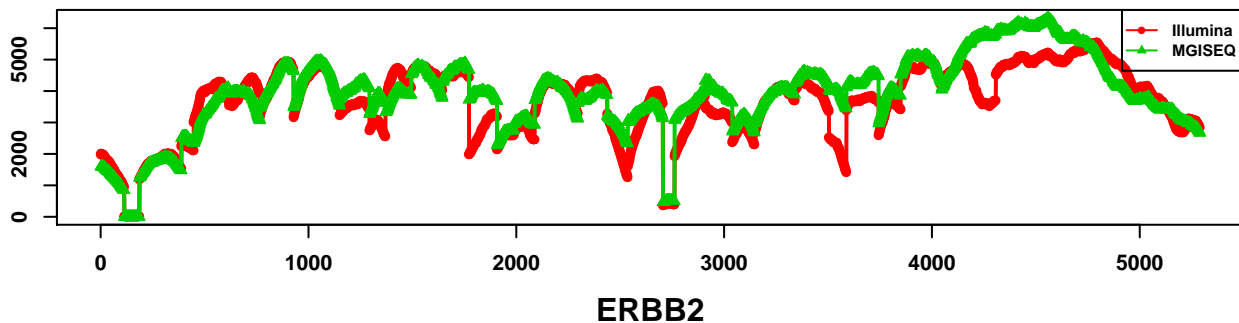

Sequencing Depth

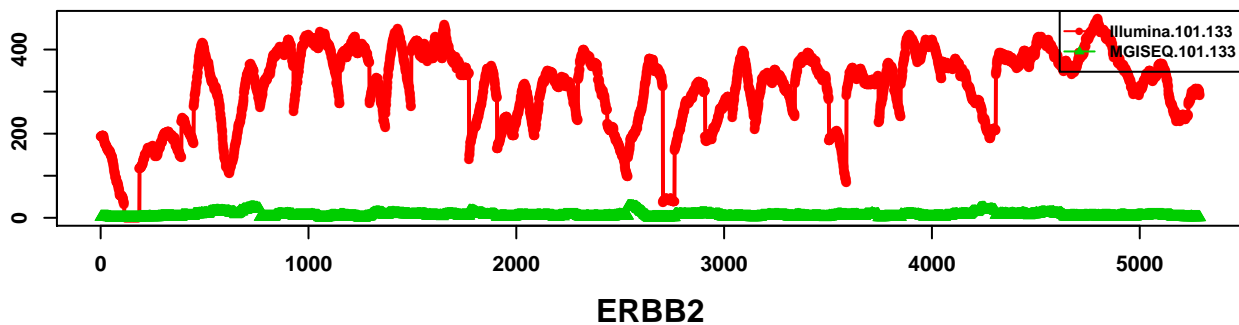

Sequencing Depth

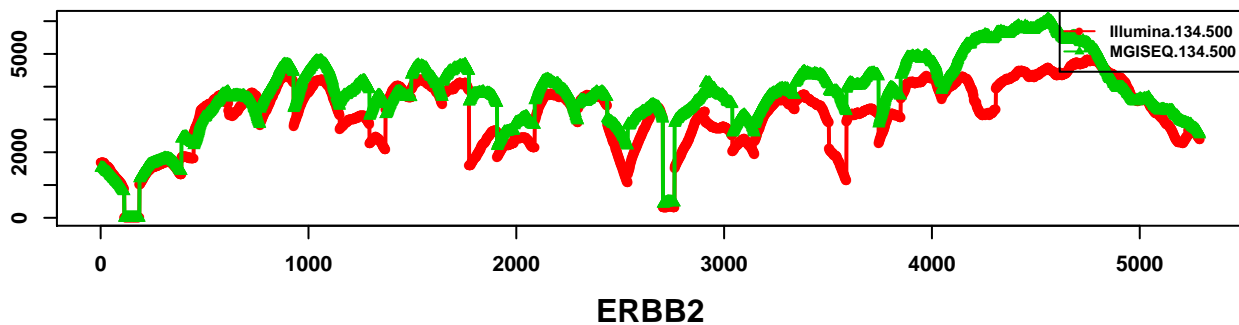

Supplement: Supplementary file 9 [file Presentation6.zip › ERBB2/19HE22145F.pdf]

Sequencing Depth

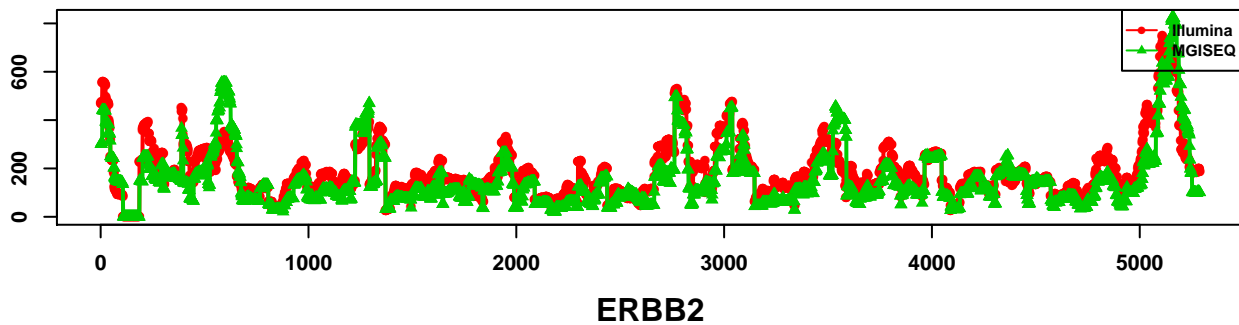

Sequencing Depth

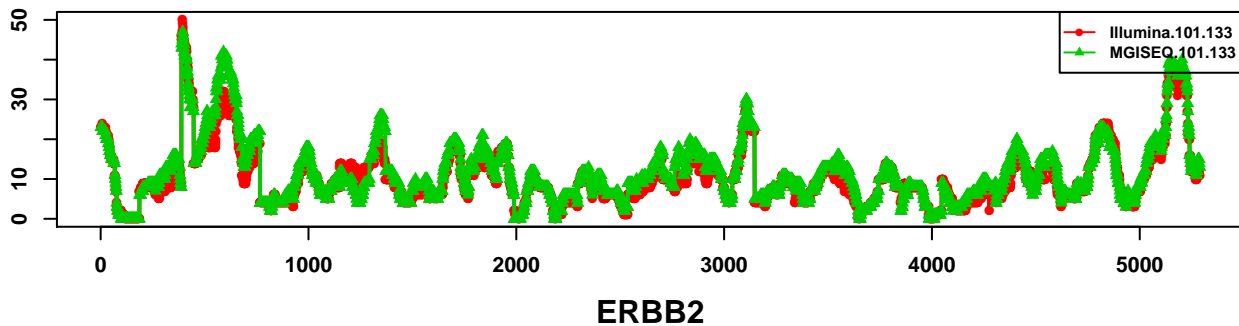

Sequencing Depth

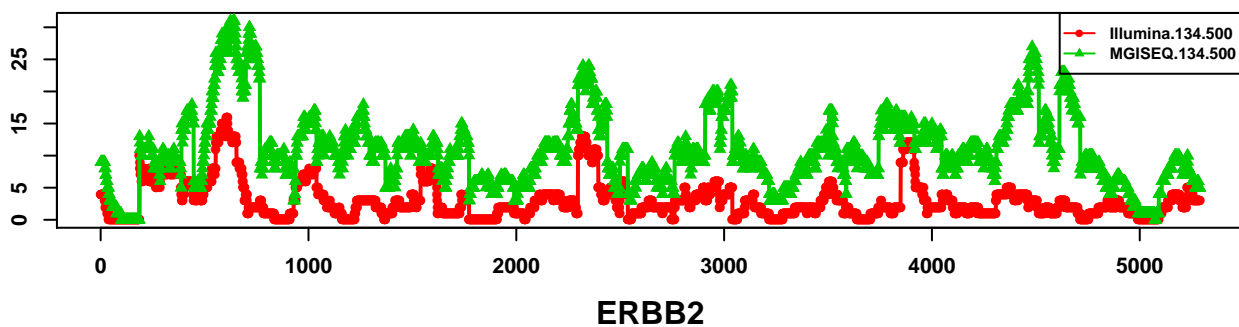

Supplement: Supplementary file 9 [file Presentation6.zip › ERBB2/19N01984F.pdf]

Sequencing Depth

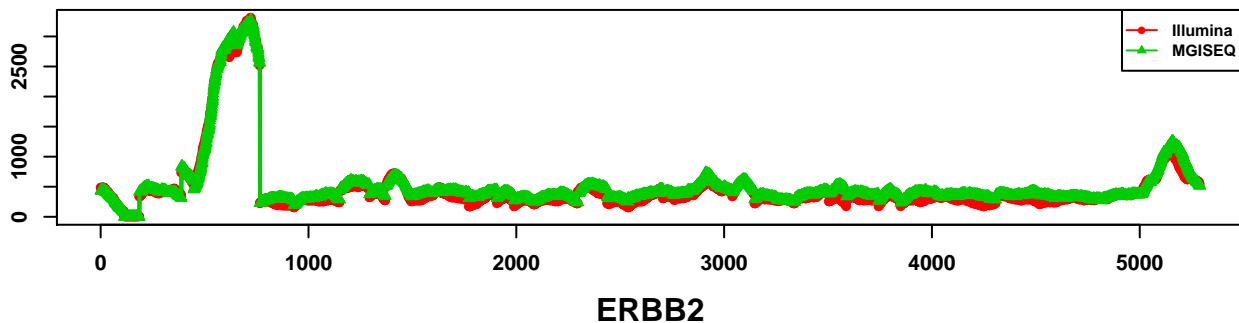

Sequencing Depth

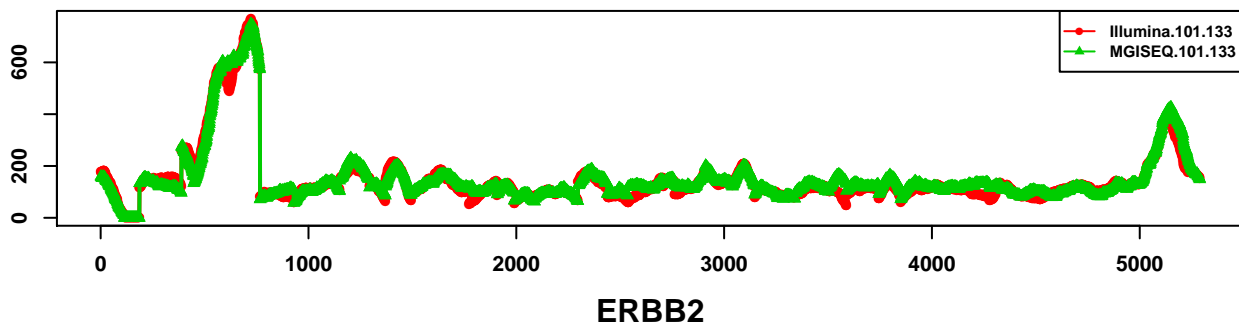

Sequencing Depth

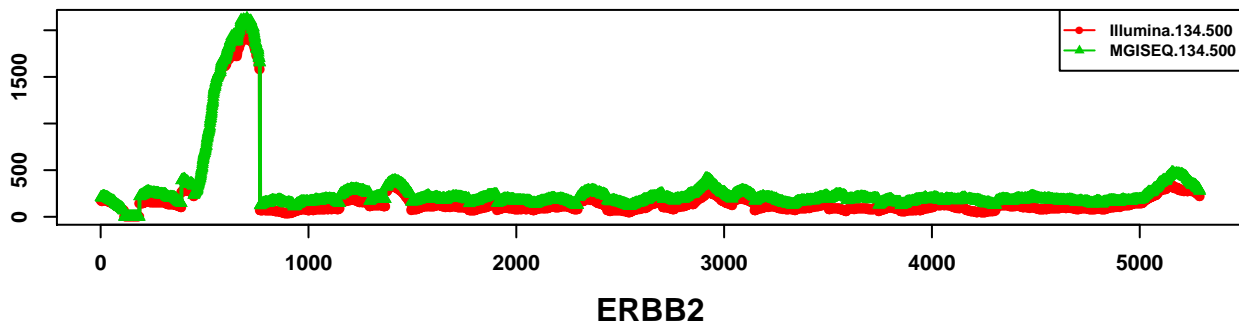

Supplement: Supplementary file 9 [file Presentation6.zip › ERBB2/19ZN13493T.pdf]

Sequencing Depth

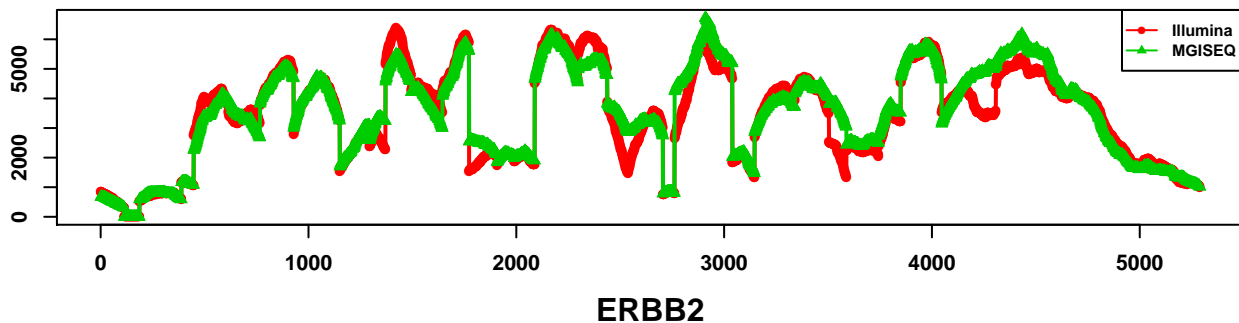

Sequencing Depth

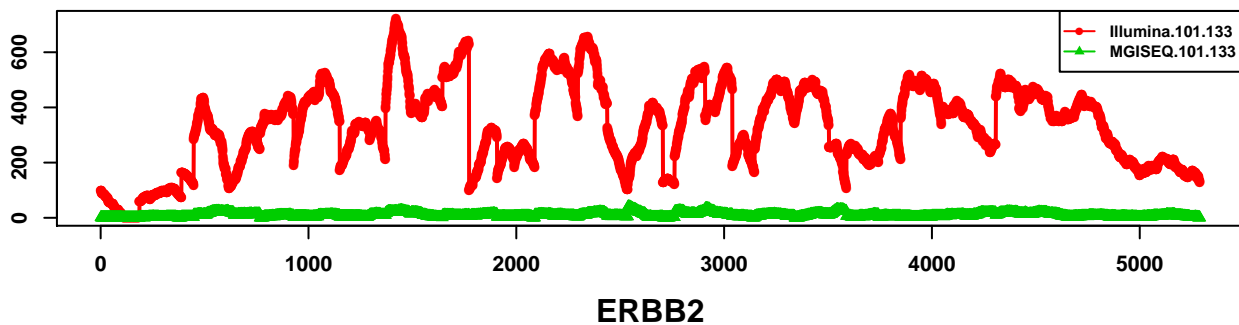

Sequencing Depth

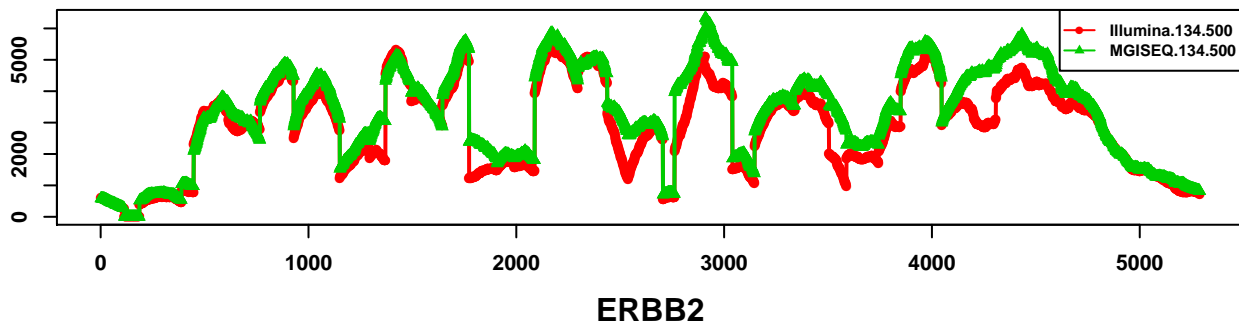

Supplement: Supplementary file 9 [file Presentation6.zip › ERBB2/19HS86164F.pdf]

Sequencing Depth

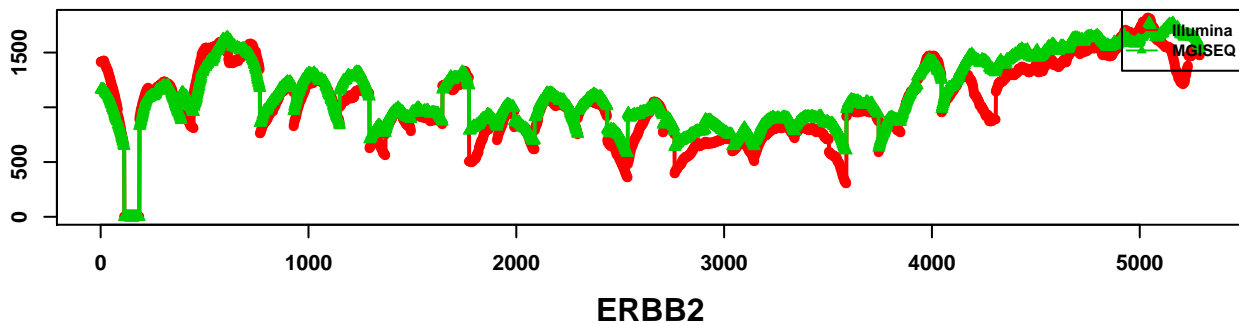

Sequencing Depth

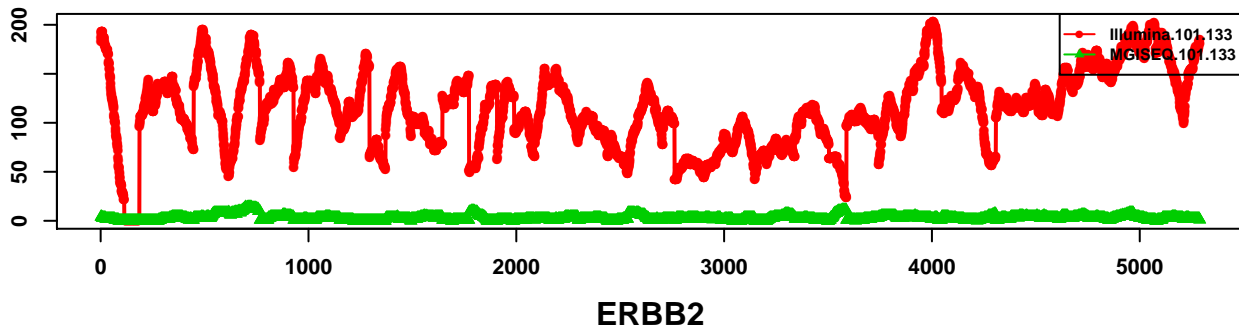

Sequencing Depth

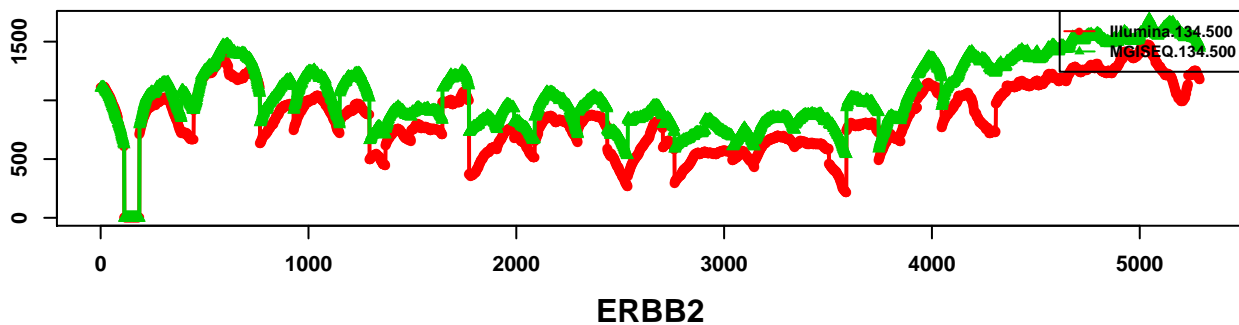

Supplement: Supplementary file 9 [file Presentation6.zip › ERBB2/19HE22001F.pdf]

Sequencing Depth

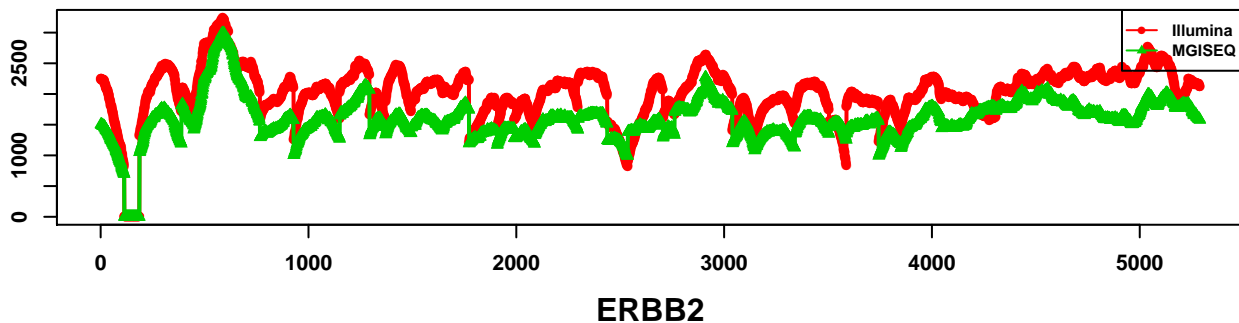

Sequencing Depth

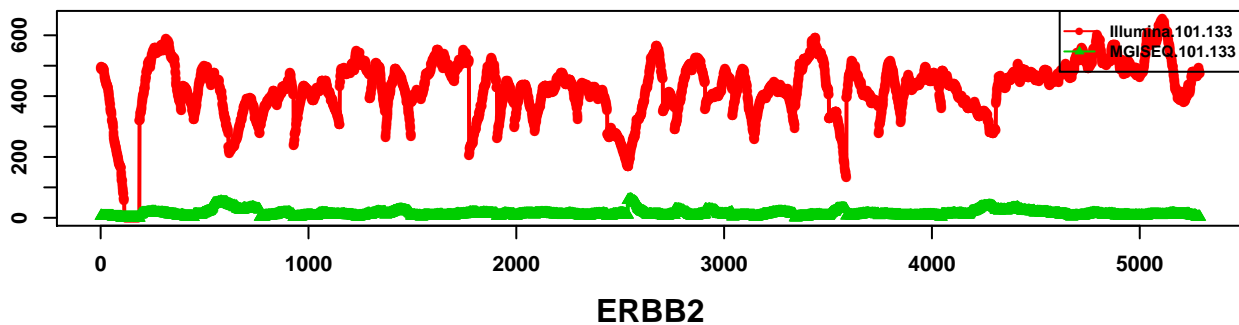

Sequencing Depth

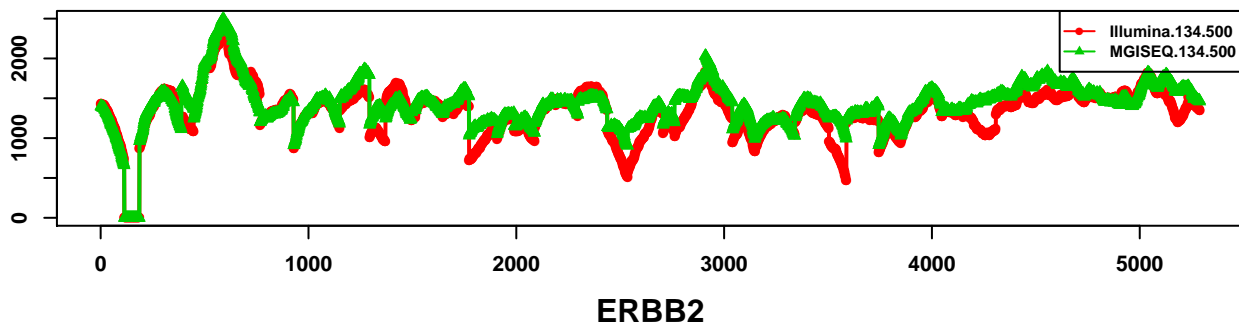

Supplement: Supplementary file 9 [file Presentation6.zip › ERBB2/19ZN12313F.pdf]

Sequencing Depth

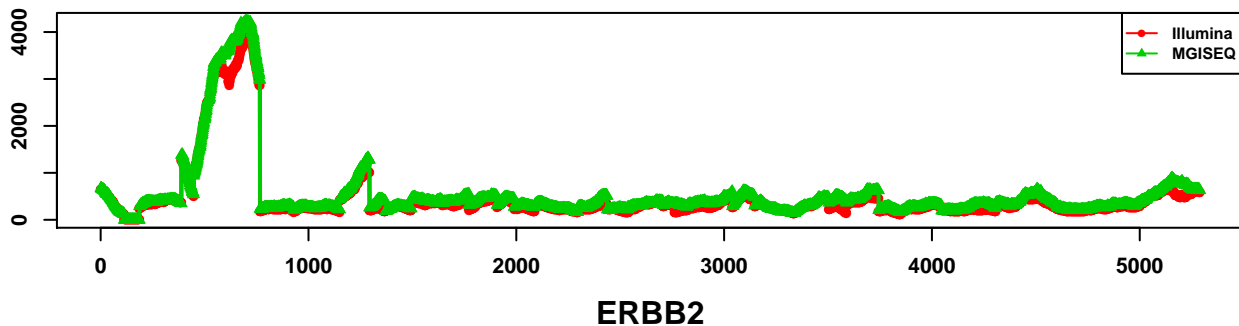

Sequencing Depth

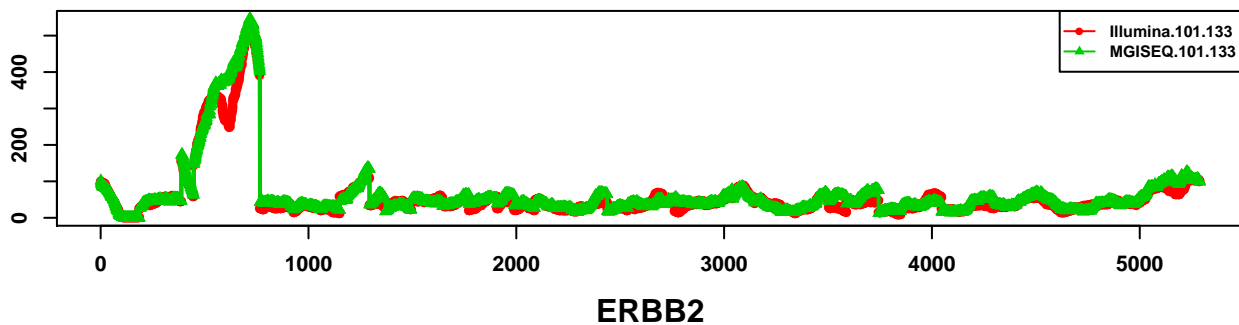

Sequencing Depth

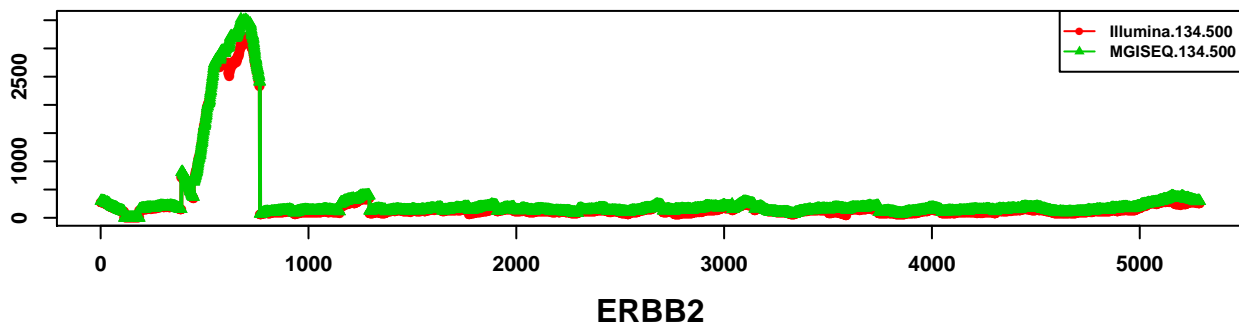

Supplement: Supplementary file 9 [file Presentation6.zip › ERBB2/19N01985F.pdf]

Sequencing Depth

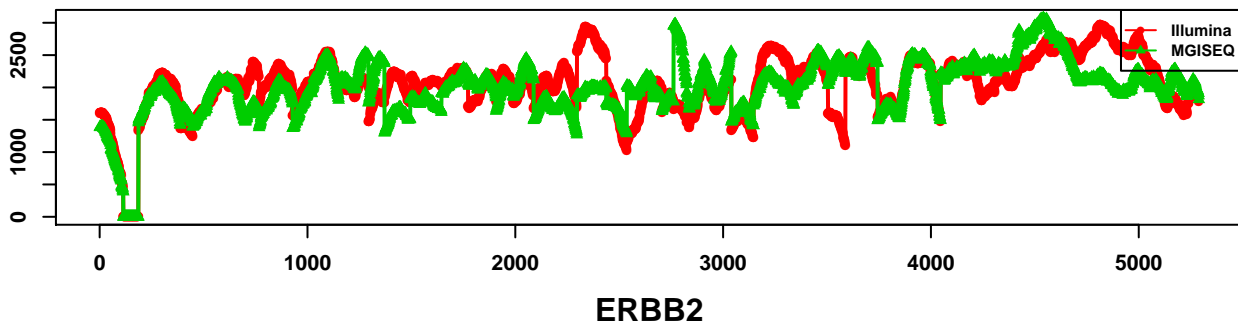

Sequencing Depth

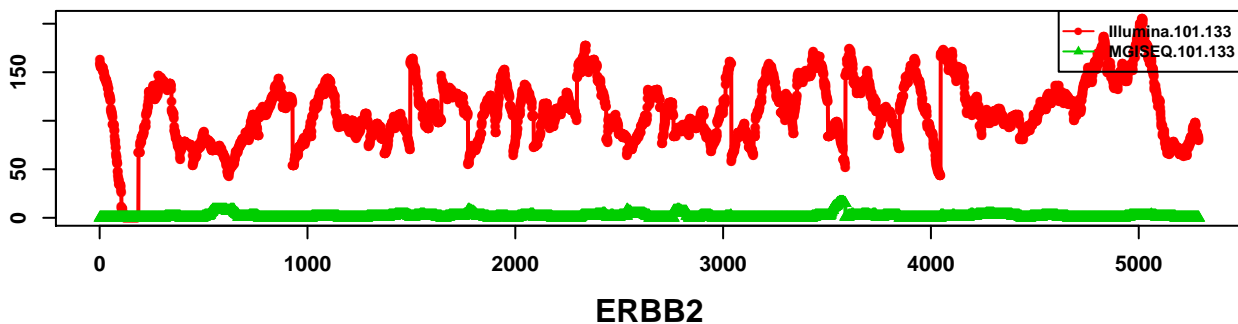

Sequencing Depth

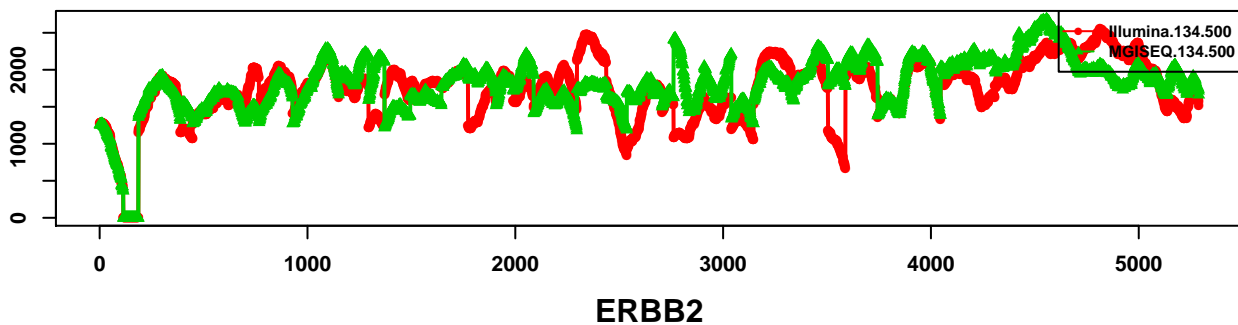

Supplement: Supplementary file 9 [file Presentation6.zip › ERBB2/19ZN12368P.pdf]
