# Supplementary material for: Evaluation of the MGISEQ-2000 Sequencing Platform for Illumina Target Capture Sequencing Libraries
Source: Front Genet. 2021 Oct 27;12:730519. doi: 10.3389/fgene.2021.730519 (PMC8578046; doi:10.3389/fgene.2021.730519)
Supplement: Supplementary file 6 [file Presentation5.zip › EGFR/19HE21977F.pdf]

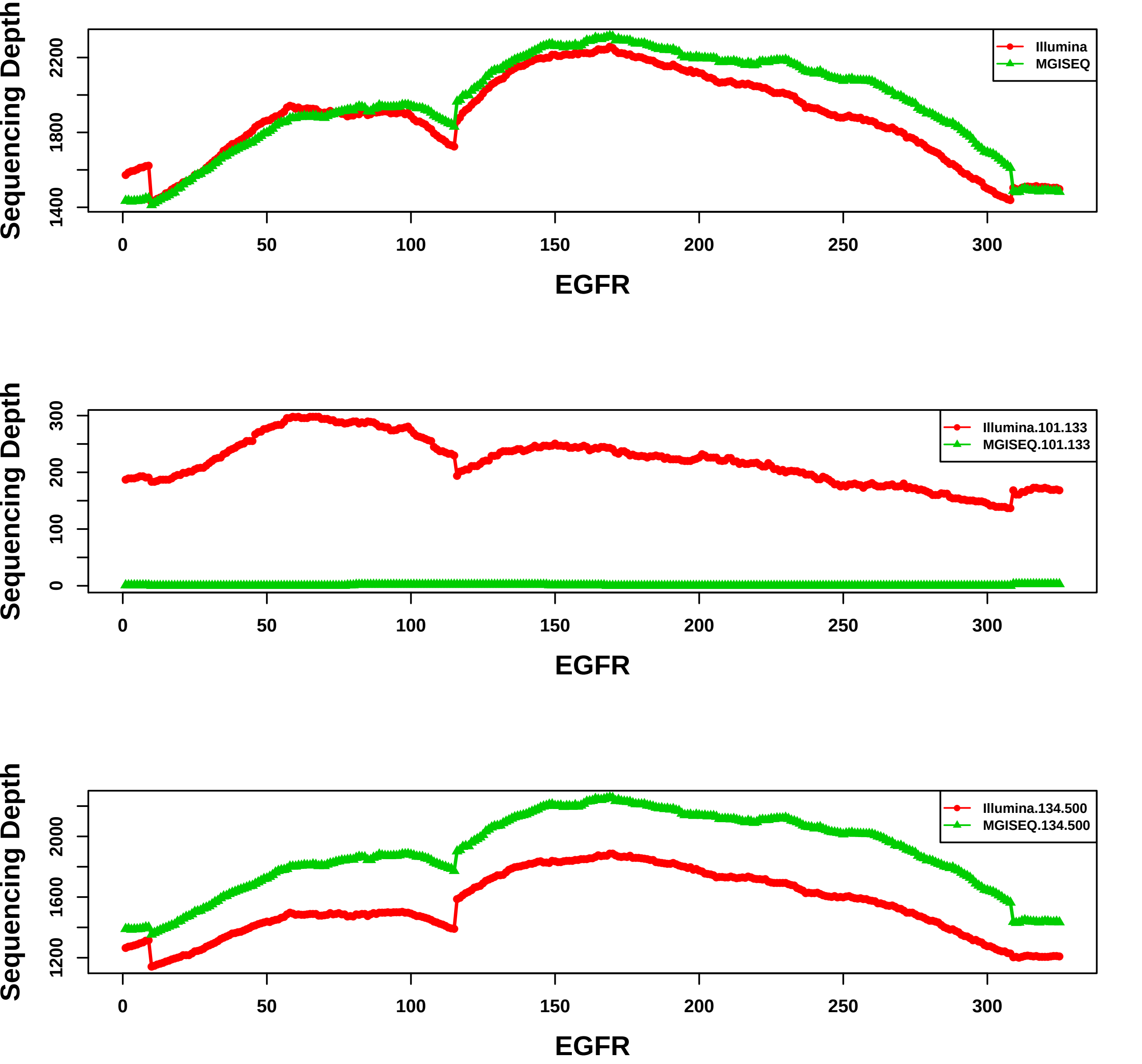
Sequencing Depth
1400
1800
2200
0
50
100
150
200
250
300
EGFR
Illumina
MGISEQ
Sequencing Depth
0
100
200
300
0
50
100
150
200
250
300
EGFR
Illumina.101.133
MGISEQ.101.133
Sequencing Depth
1200
1600
2000
0
50
100
150
200
250
300
EGFR
Illumina.134.500
MGISEQ.134.500
