# Supplementary material for: Evaluation of the MGISEQ-2000 Sequencing Platform for Illumina Target Capture Sequencing Libraries
Source: Front Genet. 2021 Oct 27;12:730519. doi: 10.3389/fgene.2021.730519 (PMC8578046; doi:10.3389/fgene.2021.730519)
Supplement: Supplementary file 6 [file Presentation5.zip › EGFR/M1900799-I-IIP.pdf]

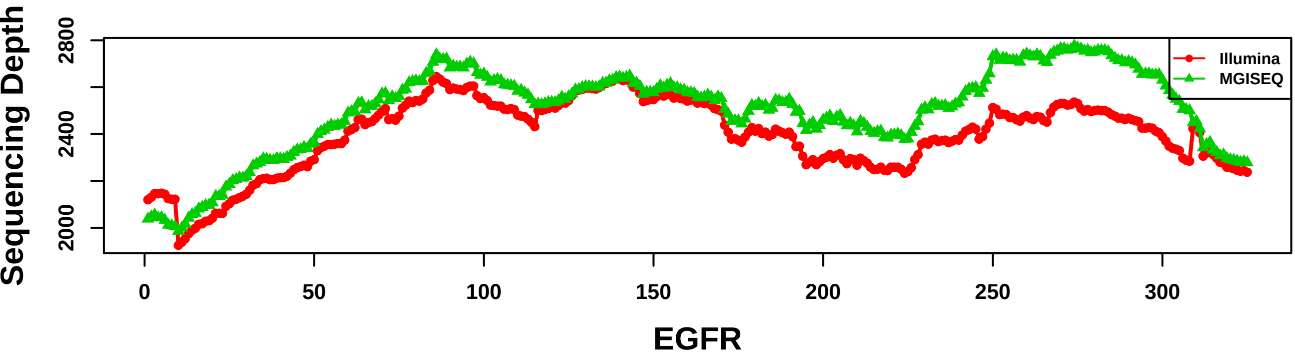
Sequencing Depth
2000
2400
2800
0
50
100
150
200
250
300
EGFR
Illumina
MGISEQ

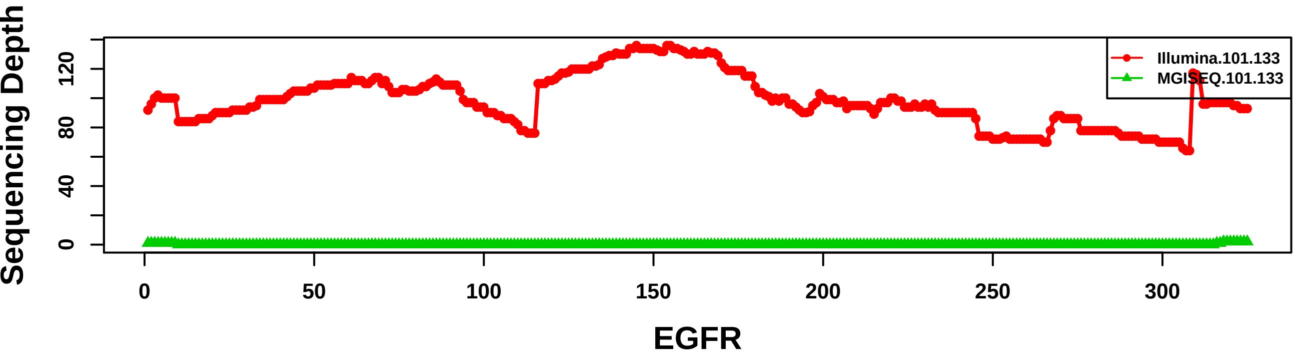
Sequencing Depth
0
40
80
120
0
50
100
150
200
250
300
EGFR
Illumina.101.133
MGISEQ.101.133

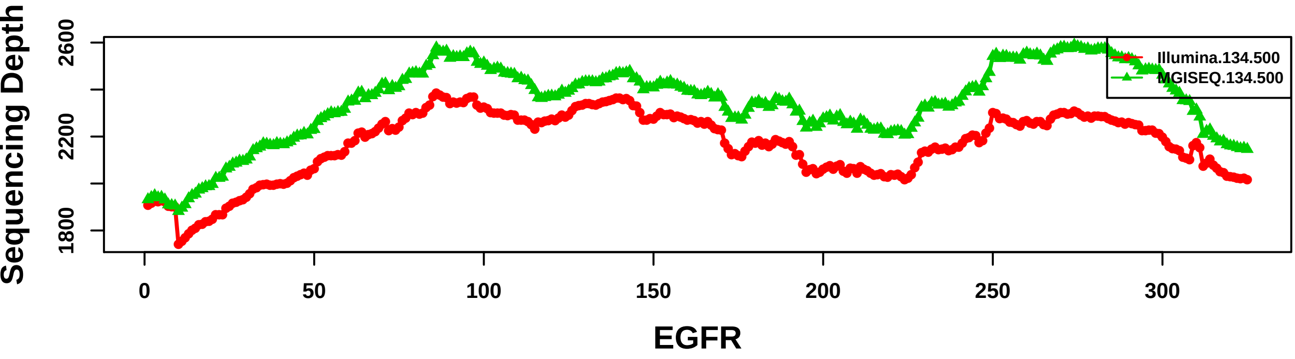
Sequencing Depth
1800
2200
2600
0
50
100
150
200
250
300
EGFR
Illumina.134.500
MGISEQ.134.500
