# Supplementary material for: Evaluation of the MGISEQ-2000 Sequencing Platform for Illumina Target Capture Sequencing Libraries
Source: Front Genet. 2021 Oct 27;12:730519. doi: 10.3389/fgene.2021.730519 (PMC8578046; doi:10.3389/fgene.2021.730519)
Supplement: Supplementary file 6 [file Presentation5.zip › EGFR/19N01651F.pdf]

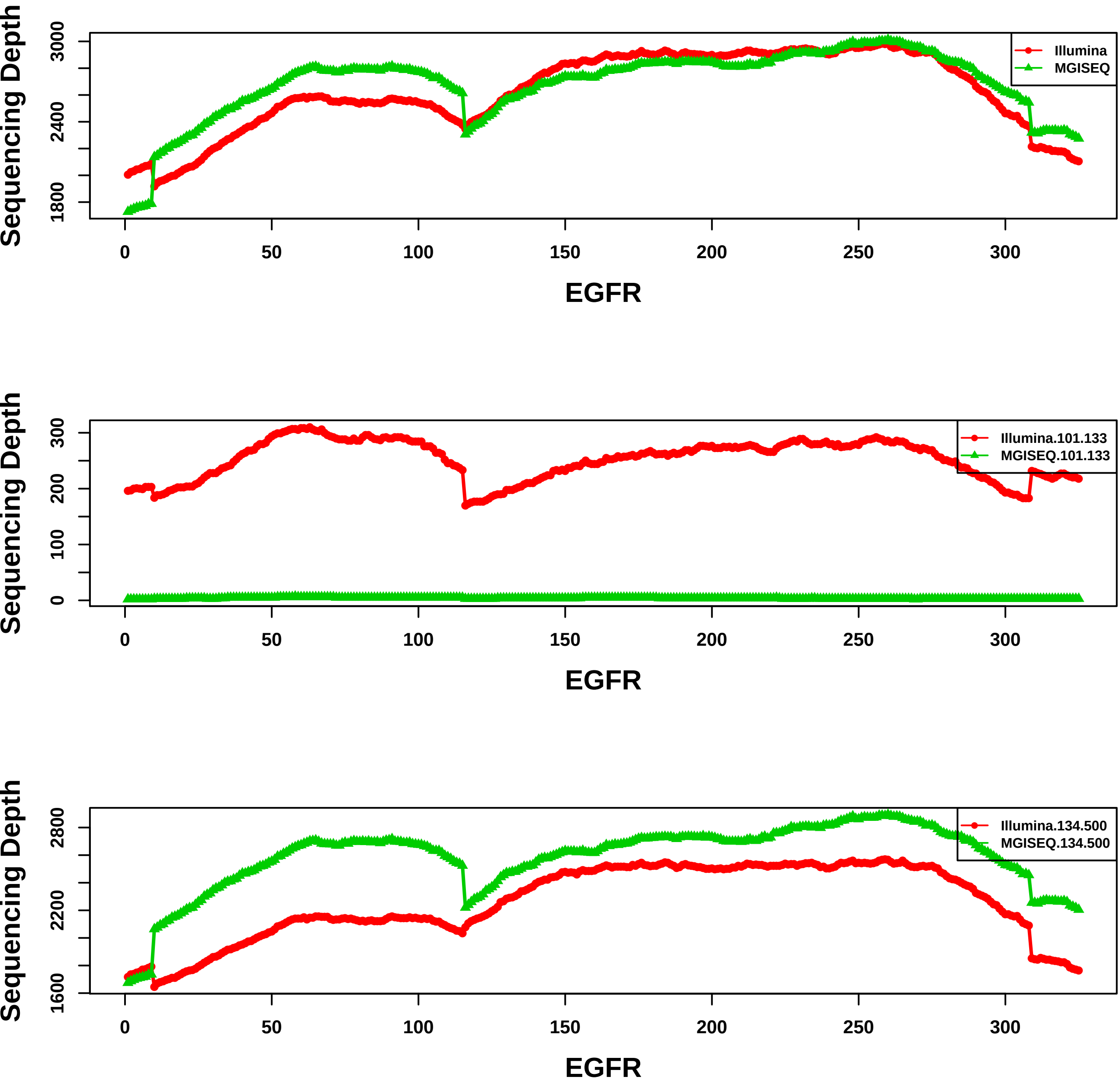
Sequencing Depth
3000
2400
1800
0
50
100
150
200
250
300
EGFR
Illumina
MGISEQ
Sequencing Depth
300
200
100
0
EGFR
Illumina.101.133
MGISEQ.101.133
Sequencing Depth
2800
2200
1600
EGFR
Illumina.134.500
MGISEQ.134.500
